# The 2024 Europe report of the *Lancet* Countdown on health and climate change: unprecedented warming demands unprecedented action

**DOI:** 10.1016/S2468-2667(24)00055-0

**Published:** 2024-05-12

**Authors:** Kim R van Daalen, Cathryn Tonne, Jan C Semenza, Joacim Rocklöv, Anil Markandya, Niheer Dasandi, Slava Jankin, Hicham Achebak, Joan Ballester, Hannah Bechara, Thessa M Beck, Max W Callaghan, Bruno M Carvalho, Jonathan Chambers, Marta Cirah Pradas, Orin Courtenay, Shouro Dasgupta, Matthew J Eckelman, Zia Farooq, Peter Fransson, Elisa Gallo, Olga Gasparyan, Nube Gonzalez-Reviriego, Ian Hamilton, Risto Hänninen, Charles Hatfield, Kehan He, Aleksandra Kazmierczak, Vladimir Kendrovski, Harry Kennard, Gregor Kiesewetter, Rostislav Kouznetsov, Hedi Katre Kriit, Alba Llabrés-Brustenga, Simon J Lloyd, Martín Lotto Batista, Carla Maia, Jaime Martinez-Urtaza, Zhifu Mi, Carles Milà, Jan C Minx, Mark Nieuwenhuijsen, Julia Palamarchuk, Dafni Kalatzi Pantera, Marcos Quijal-Zamorano, Peter Rafaj, Elizabeth J Z Robinson, Nacho Sánchez-Valdivia, Daniel Scamman, Oliver Schmoll, Maquins Odhiambo Sewe, Jodi D Sherman, Pratik Singh, Elena Sirotkina, Henrik Sjödin, Mikhail Sofiev, Balakrishnan Solaraju-Murali, Marco Springmann, Marina Treskova, Joaquin Triñanes, Eline Vanuytrecht, Fabian Wagner, Maria Walawender, Laura Warnecke, Ran Zhang, Marina Romanello, Josep M Antó, Maria Nilsson, Rachel Lowe

**Affiliations:** a Barcelona Supercomputing Center (BSC), Barcelona, Spain; b British Heart Foundation Cardiovascular Epidemiology Unit, Department of Public Health and Primary Care, University of Cambridge, Cambridge, UK; c Barcelona Institute for Global Health (ISGlobal), Barcelona, Spain; d Universitat Pompeu Fabra (UPF), Barcelona, Spain; e CIBER Epidemiología y Salud Pública (CIBERESP), Barcelona, Spain; f Heidelberg Institute of Global Health, Heidelberg University, Heidelberg, Germany; g Interdisciplinary Center of Scientific Computing, Heidelberg University, Heidelberg, Germany; h Heidelberg Institute for Geoinformation Technology (HeiGIT), Heidelberg University, Heidelberg, Germany; i Department of Public Health and Clinical Medicine, Umeå University, Umeå, Sweden; j Department of Epidemiology and Global Health, Umeå University, Umeå, Sweden; k BC3 Basque Centre for Climate Change, Bilbao, Spain; l School of Government, University of Birmingham, Birmingham, UK; m Institut National de la Santé et de la Recherche Médicale (Inserm), Paris, France; n Data Science Lab, Hertie School, Berlin, Germany; o Mercator Research Institute on Global Commons and Climate Change (MCC), Berlin, Germany; p Energy Efficiency Group, Institute for Environmental Sciences (ISE), University of Geneva, Geneva, Switzerland; q The Zeeman Institute and School of Life Sciences, University of Warwick, Coventry, UK; r Centro Euro-Mediterraneo sui Cambiamenti Climatici (CMCC), Venice, Italy; s Grantham Research Institute on Climate Change and the Environment, London School of Economics and Political Sciences, London, UK; t Department of Civil and Environmental Engineering, Northeastern University, Boston, MA, USA; u Department of Political Science, Florida State University, Tallahassee, FL, USA; v European Centre for Medium-Range Weather Forecast (ECMWF), Bonn, Germany; w Energy Institute, University College London, London, UK; x The Bartlett School of Sustainable Construction, University College London, London, UK; y Institute for Sustainable Resources, University College London, London, UK; z Institute for Global Health, University College London, London, UK; aa Finnish Meteorological Institute (FMI), Helsinki, Finland; ab European Environment Agency (EEA), Copenhagen, Denmark; ac European Centre for Environment and Health, WHO Regional Office for Europe, Bonn, Germany; ad Center on Global Energy Policy, Columbia University, New York, NY, USA; ae Pollution Management Research Group, Energy, Climate, and Environment Program, International Institute for Applied Systems Analysis, Laxenburg, Austria; af Medical School of Hannover, Hannover, Germany; ag Global Health and Tropical Medicine (GHTM), Associate Laboratory in Translation and Innovation Towards Global Health (LA-REAL), Instituto de Higiene e Medicina Tropical (IHMT), Universidade Nova de Lisboa, UNL, Lisboa, Portugal; ah Department of Genetics and Microbiology, Universitat Autònoma de Barcelona, Barcelona, Spain; ai Yale University School of Medicine, Yale University, New Haven, CT, USA; aj Department of Political Science, The University of North Carolina, Chapel Hill, NC, USA; ak Centre for Climate Change and Planetary Health, London School of Hygiene and Tropical Medicine (LSHTM), London, UK; al Environmental Change Institute, University of Oxford, Oxford, UK; am Department of Electronics and Computer Science, Universidade de Santiago de Compostela, Santiago, Spain; an University of Mannheim, Mannheim, Germany; ao Catalan Institution for Research and Advanced Studies (ICREA), Barcelona, Spain

## Executive summary

Record-breaking temperatures were recorded across the globe in 2023. Without climate action, adverse climate-related health impacts are expected to worsen worldwide, affecting billions of people. Temperatures in Europe are warming at twice the rate of the global average, threatening the health of populations across the continent and leading to unnecessary loss of life. The *Lancet* Countdown in Europe was established in 2021, to assess the health profile of climate change aiming to stimulate European social and political will to implement rapid health-responsive climate mitigation and adaptation actions. In 2022, the collaboration published its indicator report, tracking progress on health and climate change via 33 indicators and across five domains.

This new report tracks 42 indicators highlighting the negative impacts of climate change on human health, the delayed climate action of European countries, and the missed opportunities to protect or improve health with health-responsive climate action. The methods behind indicators presented in the 2022 report have been improved, and nine new indicators have been added, covering leishmaniasis, ticks, food security, health-care emissions, production and consumption-based emissions, clean energy investment, and scientific, political, and media engagement with climate and health. Considering that negative climate-related health impacts and the responsibility for climate change are not equal at the regional and global levels, this report also endeavours to reflect on aspects of inequality and justice by highlighting at-risk groups within Europe and Europe's responsibility for the climate crisis.

### Climate change is not a far-in-the-future scenario

Our report highlights the multidimensional impacts of climate change on health and health determinants in Europe that are already happening. While ensuring global temperature increases do not exceed 1·5°C will avert some of the worst climate health impacts, the world is already edging closer to this temperature increase and is failing to adequately cut emissions.

Heat-related deaths are estimated to have risen across most of Europe, with an average increase of 17·2 deaths per 100 000 inhabitants between the periods of 2003–12 and 2013–22 (indicator 1.1.4). Risky hours for physical activity (due to heat stress risk) have been spreading beyond the hottest parts of the day over the period 1990–2022 for both medium (eg, cycling or football) and strenuous (eg, rugby, or mountain-biking) activities (indicator 1.1.3), which might result in people reducing their overall physical activity and thereby increasing their risk of non-communicable diseases. Heat exposure can further undermine people's health by impacting the social and economic determinants of health. For example, labour supply was substantially lower during 2016–20 compared with a 1965–94 baseline (indicator 4.1.2). Climate suitability for various climate-sensitive pathogens and disease vectors has increased in Europe (eg, *Vibrio*, West Nile virus, dengue, chikungunya, Zika, malaria, leishmaniasis, and ticks; indicator 1.3). During 2011–20, substantially more regions were predicted to be suitable for leishmaniasis (68%) compared with 2001–10 (55%), with a northward expansion in suitable areas beyond the historical endemic zone (indicator 1.3.5). The relative increase in outbreak risk was 256% for West Nile virus from 1951–60 (outbreak risk 0·05) to 2013–22 (0·01; indicator 1.3.2), and 40·9% for dengue from 1951–60 (estimated reproduction number [R_0_] 0·09) to 2013–22 (R_0_ 0·14; indicator 1.3.3). Furthermore, the number of months suitable for *Ixodes ricinus* ticks (the vector for Lyme disease and tick-borne encephalitis) increased by 0·68 months in western Asia and 0·58 in eastern Europe. Climate change is also driving changes in the intensity and frequency of extreme climatic events. Positive trends in wildfire danger were observed across Europe during 1980–2022 (indicator 1.2.1), although no trends were detected for wildfire-particulate matter (fine particulate matter with a diameter ≤2·5 μm [PM_2·5_]) emission exposure between 2003 and 2022 (indicator 1.2.1), which might reflect effective wildfire preparedness and management. Western, southern, and eastern Europe experienced substantial increases in extreme drought conditions from 2000–09 to 2010–19 (indicator 1.2.2). Moreover, in 2021, climate change resulted in almost 12 million additional people affected by moderate or severe food insecurity in Europe (indicator 1.5.1).

### Deepening health inequities in a warming world

These interconnected health impacts tend to be unevenly distributed among populations due to differences in exposure, sensitivity, and adaptive capacity—often reflecting intersecting patterns of socioeconomic development, marginalisation, and historical and ongoing patterns of inequity. Populations most affected tend to be those least responsible and less likely to be recognised or prioritised. Southern Europe tends to be more affected by heat-related illnesses, wildfires, food insecurity, drought, and leishmaniasis, whereas northern Europe is equally or more impacted by *Vibrio* and ticks (section 1). Within countries, ethnic minoritised and Indigenous people, low-income communities, migrants and displaced people, sexual and gender minoritised people, and women experiencing pregnancy and childbirth tend to be more severely affected by climate-related health impacts.

This report shows that heat-related mortality was twice as high in women compared with men (indicator 1.1.4), low-income households had a substantially higher probability of people experiencing food insecurity (indicator 1.5.1), deaths attributable to an imbalanced diet were higher among women (indicator 3.4.2), and exposure to wildfire-PM_2.5_ was higher in highly deprived areas. Poorly designed adaptation strategies, such as nature-based solutions (indicator 2.2.2) or mechanisms to improve thermal comfort (indicator 2.2.3) that do not adequately consider equity, can perpetuate environmental and health inequities. As not all indicators can incorporate analyses on different population groups, our report offers only a glimpse of the much larger picture and emphasises the importance of more robust research to delve deeper into the unequal impacts of climate change on health to inform health protection measures for all populations.

Despite climate change exacerbating existing inequalities, indicators on governance and politics show little engagement with aspects of equality, equity, or justice in climate and health research, policy, and media (section 5). Furthermore, environmental equity, including addressing disproportionate socio-spatial distributions of climate change exposure and health risks, is not an explicit goal within existing EU policies.

### Taking responsibility and accelerating action

Many European countries remain major historical and current contributors to greenhouse gas emissions. While European countries have benefited from the economic growth that these emissions enabled, other countries—that have emitted the least—are most affected by current and future climate change. Climate change is a social and environmental justice problem. In 2021, emissions from fossil fuel combustion were 5·4 tonnes of CO_2_ per person in Europe—six times that of Africa and almost three times that of Central and South America (indicator 3.1.1). The pace at which European countries are moving towards net-zero emissions remains woefully inadequate, with Europe's current trajectory consistent with achieving carbon neutrality only by 2100 (indicator 3.1.1). Importantly, with Europe's consumption of goods and services produced in other parts of the world, European countries continue to drive environmental pressures (eg, greenhouse gas emissions and local air pollution) and their related adverse climate and health impacts elsewhere in the world (indicator 3.2.1). Despite several European countries taking action to reduce health-care emissions, the health-care sector was estimated to have contributed 330 megatonnes of CO_2_-equivalent emissions in 2020 (indicator 3.5). Furthermore, coal use increased to 13% of Europe's total energy supply in 2021 (indicator 3.1.2), and 29 of 53 countries are still providing net subsidies for fossil fuels (indicator 4.2.1).

The absence of bold action risks further exacerbating the impacts of climate change that are already happening and misses opportunities to bring considerable near-term health co-benefits, such as reduced premature mortality due to a reduction in ambient fine particles (indicator 3.2.1); increased physical activity from more active transport; and reduced morbidity and mortality by shifting towards less-polluting, less-processed, resource-efficient, and healthy plant-based diets (indicator 3.4).

Limiting warming to less than 1·5°C to avert further detrimental health impacts requires governments across Europe to strengthen their response. Therefore, political and governance structures across Europe should engage with the health dimensions of climate change. However, while scientific (indicator 5.1) and corporate sector (indicator 5.4) engagement continued to grow in 2022, there were low levels of media (indicator 5.5), political (indicator 5.3), and individual engagement (indicator 5.2) with the climate–health nexus. Given that health framing could strengthen public and political support for climate action and the need for societies in Europe to adapt to the health impacts of climate change, fostering climate-health awareness across political actors and institutions is essential to further stimulate action.

### A fair and healthy environmental transition

To meet the recommendations of the latest Intergovernmental Panel on Climate Change report of net-zero by 2040, emissions from Europe's energy systems should fall by three times the current rate. This decrease will need to happen even faster if fair-share emissions, which take Europe's historical emissions and population into account, are used to allocate reductions globally. When justice is considered, climate action does not only guarantee a fair and healthy environmental transition, but also reduces inequities in key health impact pathways, including air pollution, physical activity from active transport, and healthy diets between and within countries. Recognising the impacts of climate change within and beyond Europe and Europe's role in creating the climate crisis, Europe should commit to a fair and healthy environmental transition, which includes taking global responsibility and supporting the most affected communities.

## Introduction

After a century of fossil fuels being burnt worldwide, Europe is facing unprecedented warming and escalating extreme climatic events, highlighted by record-breaking heat, droughts, and floods in 2022 and 2023. Without swift and drastic action, climate change will continue to accelerate further, accompanied by detrimental impacts on human health and wellbeing worldwide.^1^ These impacts are not felt equally across the world, nor across European populations.^2^

Politically, some progress has been made in Europe with the adoption of the European Climate Law,^3^ EU Adaptation Strategy, and Budapest declaration as the outcome of the Seventh Ministerial Conference on Environment and Health.^4^ Furthermore, the 28th UN Framework Convention on Climate Change (COP28) considered health for the first time in official programming, and 149 countries (including within the EU) endorsed a declaration on climate change and health.^5^ However, the new Euro 7 Emissions Standards and the Industrial Emissions Directive are still inadequate to target emissions and pollution, and Europe remains one of the major historical and current contributors of greenhouse gas emissions,^6^ while outsourcing many negative environmental pressures related to EU consumption elsewhere.^7^ Furthermore, on the basis of the latest Intergovernmental Panel on Climate Change (IPCC) synthesis report, Europe should increase ambition to reach climate neutrality as close as possible to 2040 (instead of the current 2050 targets) to keep global warming within safe limits, which would deliver simultaneous improvements in air quality. Reaching climate neutrality earlier would deliver health co-benefits in addition to averting further climate change.^8^ Importantly, when appropriately considering equity and justice, global temperature increase should be kept to less than 1°C relative to pre-industrial times instead of 1·5–2°C limits.^9^ Yet, the combined pledges in nationally determined contributions are putting the world on track for around 2·5°C of warming.^10^

This report is the second report tracking progress on health and climate change in Europe. The collaboration tracks 42 indicators across five domains (panel) drawing on the transdisciplinary expertise of 69 contributors spanning 42 academic and UN institutions. Nine new indicators have been added since the 2022 report (appendix 4 pp 8–10).^11^, ^12^ Most of the pre-existing indicators^11^ have been improved by enhancing the geographical coverage (eg, from EU-27 to EEA-38) or resolution (eg, from the country level to Nomenclature of territorial units for statistics [NUTS]2 or the gridded level), expanding temporal coverage, or strengthening methodology. Where possible, indicators included the 53 countries of the WHO European region plus Liechtenstein and Kosovo (defined under the UN Security Council Resolution 1244) with a detailed description of the geographical definition of Europe and European subregions can be found in appendix 4 (pp 4–7).

Panel **Indicators of the 2024 Europe Report of the Lancet Countdown**

**Climate change impacts, exposures, and vulnerabilities**

1.1. Heat and health
  1.1.1. Vulnerability to heat exposure
  1.1.2. Exposure of at-risk populations to heatwaves
  1.1.3. Physical activity-related heat stress risk
  1.1.4. Heat-related mortality
1.2. Extreme events and health
  1.2.1. Wildfire smoke
  1.2.2. Drought
1.3. Climate-sensitive infectious diseases
  1.3.1. Climatic suitability for *Vibrio*
  1.3.2. Climatic suitability for West Nile virus
  1.3.3. Climatic suitability for dengue, chikungunya, and Zika
  1.3.4. Climatic suitability for malaria
  1.3.5. Climatic suitability for leishmaniasis*
  1.3.6. Climatic suitability for ticks*
1.4. Allergens
  1.4.1. Allergenic trees
1.5. Food and water
  1.5.1. Food security and undernutrition*

**Adaptation, planning, and resilience for health**

2.1. Adaptation, planning, and assessment
  2.1.1. National vulnerability and adaptation assessments
  2.1.2. National adaptation plans for health
  2.1.3. City-level climate change risks assessments
2.2. Adaptation delivery and implementation
  2.2.1. Climate information for health
  2.2.2. Green space
  2.2.3. Air conditioning benefits and harms

**Mitigation actions and health co-benefits**

3.1. Energy system and health
  3.1.1. Carbon intensity of the energy system
  3.1.2. Coal phase-out
  3.1.3. Renewable and zero-carbon emission electricity
3.2. Air pollution and health co-benefits
  3.2.1. Premature mortality attributable to ambient fine particles
  3.2.2. Production-based and consumption-based attribution of CO_2_ and PM_2·5_ emissions*
3.3. Sustainable and healthy transport
3.4. Food, agriculture, and health
  3.4.1. Lifecycle emissions from food demand, production, and trade
  3.4.2. Sustainable diets
3.5. Health-care sector emissions and harms*

**Economics and finance**

4.1. Health-linked economic impacts and mitigation of climate change
  4.1.1. Economic losses due to weather-related extreme events
  4.1.2. Change in labour supply
  4.1.3. Impact of heat on economic activity
  4.1.4. Monetised value of unhealthy diets
4.2. Economics of the transition to zero-carbon economies
  4.2.1. Net value of fossil fuel subsidies and carbon prices
  4.2.2. Clean energy investment*

**Public and political engagement**

5.1. Scientific engagement with health and climate change
  5.1.1. Coverage of health and climate change in scientific articles
  5.1.2. Coverage of the health impacts of anthropogenic climate change*
5.2. Individual engagement with health and climate change on social media
5.3. Political engagement with health and climate change
  5.3.1. Engagement with health and climate change in the European Parliament
  5.3.2. Political engagement with health and climate change on social media*
5.4. Corporate sector engagement with health and climate change
5.5. Media engagement with health and climate change*

Wherever possible and appropriate, indicators consider aspects of inequality and justice by analysing or disaggregating results by, for example, sex, age, or socioeconomic indices (eg, deprivation index), or focusing on specific at-risk groups (eg, older people and outdoor workers).

## Section 1: climate change impacts, exposures, and vulnerabilities

The health impacts of climate change are increasingly manifested in populations in Europe; both from the direct consequences of changes in temperature, precipitation, and extreme events, and from indirect consequences of the alterations in environmental and social systems upon which health depends. Most of the impacts tracked in this report disproportionally affect the most marginalised and disadvantaged populations in every country.

This section includes 14 indicators tracking the impacts, exposures, and vulnerabilities from rising temperatures, extreme weather and climatic events, climate-sensitive infectious diseases, allergens, and food insecurity. Following the IPCC definition, vulnerability is a combination of exposure to the hazard, susceptibility (sensitivity), and adaptive capacity.^13^ Three new indicators have been added, including food security and climatic suitability for tick-borne disease and leishmaniasis.

### 1.1: health and heat

#### Indicator 1.1.1: vulnerability to heat exposure

Increased exposure to high temperatures in Europe leads to a range of negative health impacts, with older people, those with pre-existing chronic conditions, urban populations, people working outdoors (often disproportionately migrants), those socially deprived, (pregnant) women, and newborn babies being more at risk.^14^ This indicator derives a heat vulnerability index by combining demographic and medical data: the percentage of the population older than 65 years, the percentage of the population living in urban areas, and the prevalence of diseases associated with increased heat vulnerability (appendix 4 pp 17–19).

Heat vulnerability increased by 9% from 1990–2022 in Europe (from 37·9% to 41·2%). The highest absolute vulnerability was observed in western Europe. However, the highest relative increase in vulnerability (1990–2022) was observed in western Asia (11·6%) and southern Europe (11%), and the lowest in northern and western Europe (both around 5%).

#### Indicator 1.1.2: exposure of at-risk populations to heatwaves

**Figure 1:**
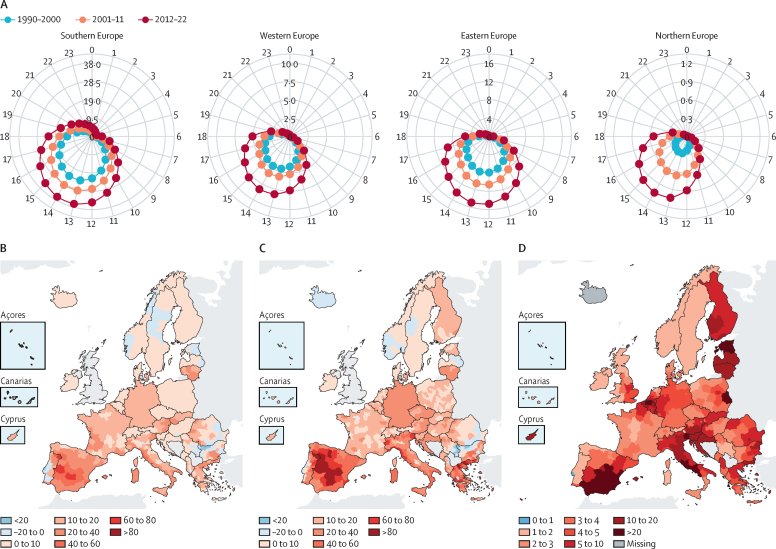
Heat and health in Europe (A) Mean annual risky hours per person for physical-activity-related heat stress (activities of medium intensity) per European subregion by time of the day for three time periods (1990–2000, 2001–11, and 2012–22). The outer grey circle shows the time of the day on a 24-hour clock, with inner grey circles showing the number of risky hours. (B) Change in heat-related mortality rate expressed as the number of deaths per 100 000 inhabitants between 2003–12 and 2013–22 for men and (C) for women. (D) Changes in the likelihood of extreme heat-related mortality episodes due to anthropogenic warming, expressed as a ratio between the probability in the recent 2003–22 period and the pre-industrial period (1850–1900).

During the summer of 2023, Europe faced record breaking temperatures, with extreme heatwaves impacting the southern half of the continent with some areas seeing temperatures above 45°C.^15^ This indicator shows that there was a 97% relative increase in the total number of person-days of heatwave exposure in the last decade (2012–21) compared with the previous decade (2000–09), increasing from 650 million to a total of 1·28 billion person-days (appendix 4 pp 20–23). This rise in person-days encompasses both an increase in at-risk populations (older people and children), and an increase in heatwave frequency, where the number of heatwave days increased by 41%. Results vary across European subregions and countries (figure 1A–B), with an increase of more than 10 days in central and southern Spain and large increases in Greece and eastern European countries.

#### Indicator 1.1.3: physical activity related heat stress risk

Regular physical activity is a key component of a healthy sustainable lifestyle,^16^ but exercising in hot weather poses a risk of heat-related illnesses, such as heat exhaustion or exertional heat stroke.^17^ Faced with heat, physical activity might be suppressed, or delayed until cooler times of the day (in people with time flexibility).^18^ This indicator assesses the evolving diurnal patterns during which there is heat stress risk when undergoing physical activity, unless risk-reducing actions are taken (figure 1A).^17^ Risky hours have been expanding into hours beyond the hottest part of the day over time for both medium and strenuous activities (appendix 4 pp 24–31). Comparing 2012–22 to 1990–2000, the mean annual risky hours per person for moderate intensity activities (eg, cycling, football, and tennis) falling outside the hottest 4 hours of the day increased in eastern (by 107%), northern (382%), southern (94%), and western Europe (101%).

#### Indicator 1.1.4: heat-related mortality

In 2022, warming since the latter half of the 19th century was almost 1°C higher in Europe than the corresponding global increase,^19^ with the 2022 summer estimated to have resulted in over 60 000 heat-related premature deaths.^20^, ^21^ With ongoing global warming, climate projections for Europe suggest a progressive reduction in cold-related deaths, and a simultaneous increase in heat-related deaths, with a 2021 study indicating that heat-related deaths have started to exceed reductions in cold-related deaths around 2010.^22^

The first part of this indicator uses weekly European Centre for Medium Range Weather Forecasts (ERA5-Land) temperatures^23^ and Eurostat mortality data^24^ to compute the change in the heat-related mortality rate between 2003–12 and 2013–22. Heat-related deaths are estimated to have increased in 771 (94%) of the 823 regions monitored (appendix 4 pp 32–35). The overall mean increase was estimated to be 17·2 deaths per 100 000 inhabitants (95% CI 10·3–24·9) rising from 50·8 (29·6–72·6) in 2003–12 to 68·0 (39·9–97·5) in 2013–22. The effects are not equally distributed: increase in heat-related mortality was almost twice as high in women at 21·5 (12·1–29·8), rising from 67·0 (36·6–93·7) to 88·4 (48·7–123·4) compared with men at 13·8 (9·9–17·7) that increased from 42·1 (28·0–55·9) to 55·9 (37·9–73·6) deaths per 100 000 inhabitants (figure 1B–C). Country-level increases ranged from 39·9 (28·0–52·8) deaths per 100 000 inhabitants in Spain to 1·0 (–6·9 to 5·9) in Iceland.

The second part of the indicator uses the annual maxima of weekly heat-related mortality, and then applies an extreme event attribution framework^25^ to calculate the changes in the likelihood of extreme heat-related mortality episodes occurring due to anthropogenic warming (appendix 4 pp 36–39). The model output is expressed as a ratio between the probability of occurrence of extreme heat-related deaths in a model driven by temperature over a recent period (2003–22 or 1981–2000) and a pre-industrial period (1850–1900). Using 2003–22 as the recent period, the indicator shows regional probability ratios greater than one in every country (ie, anthropogenic warming has contributed to the increase in the likelihood of extreme heat-related mortality episodes; figure 1D), with a median value equal to 4·1 (95% CI 0·99–773·24); 4·50 (0·99–368 296) in women and 3·62 (0·99–618·6) in men. When using 1981–2000 as the recent period, probability ratios were slightly lower, with a median value of 2·10 (0·99–12·91; appendix 4 pp 36–39). In 95% of the 232 administrative units assessed, the probability ratio for 2003–22 exceeded one, illustrating statistical significance at a 90% confidence level (or in 60% of administrative units at 95% confidence). The indicator also highlights geographical differences: in 2003–22, there was a probability ratio of 5·36 (1·15–infinity) in southern Europe, 4·03 (1·00–1177·12) in western Europe, 3·34 (1·00–167·03) in eastern Europe, and 3·09 (0·92–151·70) in northern Europe.

### 1.2: extreme events and health

#### Indicator 1.2.1: wildfire smoke

Exposure to wildfire smoke is associated with an increased risk of mortality and morbidity.^26^, ^27^ While European fire control and management have improved since pre-industrial times, fire hazards from anthropogenic climate change and epidemiological and demographic trends threaten to increase the health burden from forest fire smoke.^28^ This indicator tracks climate-driven change in wildfire danger (Fire Weather Index), estimates changes in the annual population-weighted exposure to wildfire-PM_2.5_, and estimates deaths attributable to wildfire smoke (appendix 4 pp 40–48).^29^

**Figure 2:**
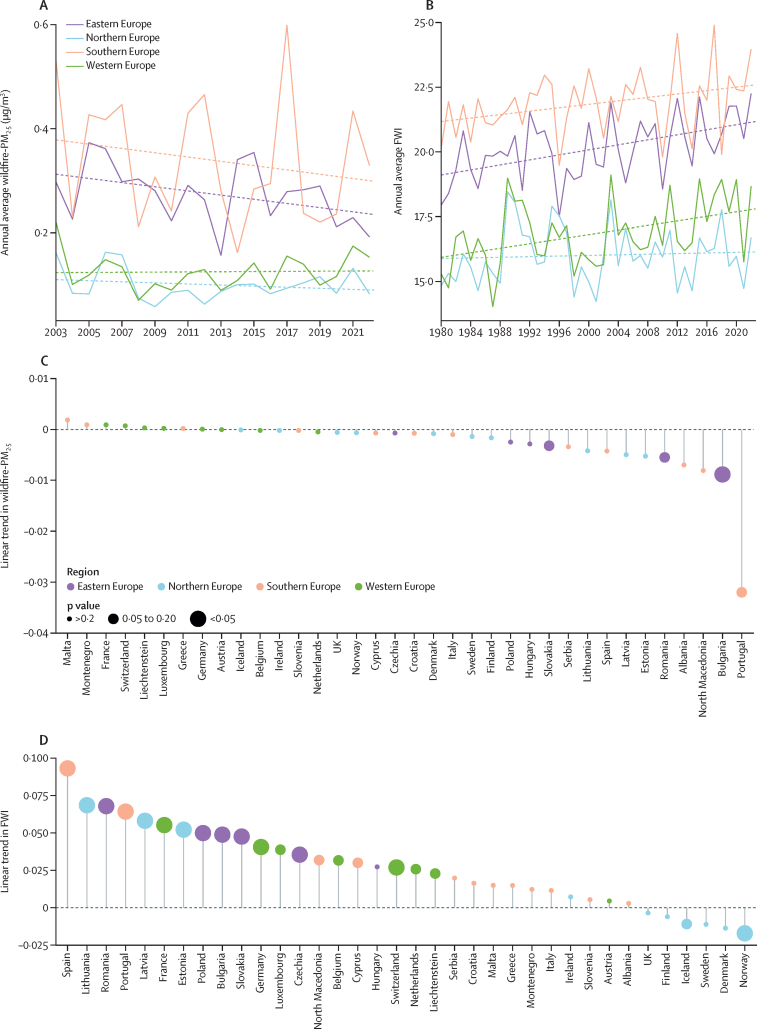
Wildfire danger and smoke (A) Annual average population-weighted wildfire-PM_2·5_ exposure (2003–22) and (B) wildfire danger according to the Canadian forest FWI (1980–2022) by European subregion, including a linear trend (dashed) during 2003–22. None of the wildfire-PM_2·5_ trends were statistically significant (p>0·05), while the wildfire danger FWI trends for eastern, southern, and western Europe were significant (p≤0·02). (C) Linear trends in annual average population-weighted wildfire-PM_2.5_ (2003–22), and (D) fire risk according to FWI (1980–2022) at country level. Dots indicate the statistical significance of the trend coefficient and colour the European subregion. FWI=Fire Weather Index.

While clear positive trends in wildfire danger were observed in eastern, southern, and western Europe during 1980–2022, wildfire-PM_2·5_ exposure trends did not show any clear positive or negative patterns during 2003–22 (figure 2A–B). In 2022, wildfire-PM_2·5_-related European-wide estimates of deaths were 737 (95% CI 501–988). The most affected countries in terms of wildfire smoke (figure 2C), wildfire danger (figure 2D), and attributable mortality were in southern and eastern Europe. Throughout Europe, results show greater wildfire smoke exposure and risk in highly deprived NUTS2 areas compared with medium or low deprived areas (appendix 4 pp 40–48).

The difference in trends in wildfire smoke compared with wildfire danger especially evident in countries with large fire danger increases, such as Spain, Portugal, and Bulgaria (figure 2B) might reflect effective wildfire preparedness, adaptation, and management.^30^, ^31^

#### Indicator 1.2.2: drought

Droughts and water scarcity are increasingly common in Europe.^32^ While most of Europe is considered to have adequate water sources, in some areas of Europe, the increase in severity and frequency of droughts can lead to long-term public health problems derived from water scarcity, which can be exacerbated by overexploitation of available water sources.^33^ This indicator examines both the change in drought frequency using the Standardized Precipitation Evapotranspiration Index and water scarcity in European river sub-basins, using the Water Exploitation Index plus (representing the ratio between the seasonal water demand and water resources—ie, incorporating water availability to people; appendix 4 pp 49–56), from 2000–09 to 2010–19.

A substantial increase in moderate (8%), severe (60%), and extreme (48%) summer drought conditions was observed in western Europe when comparing 2010–19 to 2000–09. Southern and eastern Europe had worsening drought conditions, while northern Europe saw decreases in moderate (–1%), severe (–18%), and extreme (–63%) droughts. Among the regions affected by drought, over 50% of southern Europe (particularly the Iberian Peninsula) has also been affected by water scarcity. Despite increased extreme drought events, water scarcity decreased has in recent years under these drought conditions, which might partly be due to the region's familiarity with drought and associated improved resilience.^34^, ^35^ Western and eastern Europe on the other hand faced increased water scarcity, partly due to increasing drought conditions. For instance, during the extreme drought episodes in 2010–19, over 40% of the river sub-basins in western Europe had water scarcity, compared with almost none in the previous decade. These findings emphasise the heightened need to protect crucial freshwater resources, which are essential for human health, ecological balance, and the functioning of economies and societies, to minimise water scarcity.^36^

### 1.3: climate-sensitive infectious diseases

#### Indicator 1.3.1: climatic suitability for Vibrio

Rises in sea surface temperatures have led to a higher percentage of Europe's coastline alongside brackish water to become ecologically suitable for pathogenic *Vibrio* spp, which favour *Vibrio* disease transmission.^37^ When ingesting contaminated food or experiencing direct wound contact, *Vibrio* bacteria can lead to skin, ear, and gastrointestinal issues, and more severe health outcomes, such as necrotising fasciitis. Many domestically acquired infections occurring in northern European countries have been associated with swimming and bathing,^38^, ^39^ particularly during heat waves (eg, in 1994, 1997, 2003, 2006, and 2010).^37^ This indicator uses a validated climatic suitability prediction model for *Vibrio* spp. Updates since the 2022 *Lancet* Countdown report^11^ include the estimated changes in coastlines at risk, the population at risk, and the disease burden figures (appendix 4 pp 57–60).

A total of 21 countries showed suitable areas for *Vibrio* spp in Europe in 2022, 2 188 cumulative days of exposure risk, which is the third highest ever recorded. The length of the coastline affected in these countries grew to 28 263 km, showing a consistent increase from 1982 to 2022 with a mean expansion of 136 new km of coastline suitable per year. Some of the countries with the greatest expansion in suitable coastline area were located around the Baltic Sea (a hotspot for *Vibrio* infections), with Sweden showing a relative change of 51%, and 59% in Finland, compared with 1982–2010. Other countries with an expansion of suitability include, among others, Belgium (207%), the Russian Federation (169%), and the Netherlands (131%). In 2022, the NUTS2 region of Istanbul (TR10 subregion) resulted in a population at risk of approximately 16 million and Zuid-Holland (NL33) with 3·6 million. The total population living in coastal areas of *Vibrio* suitability in Europe reached a record 150 million people in 2022 and was estimated to have resulted in 63 720 infections.

#### Indicator 1.3.2: climatic suitability for West Nile virus

West Nile virus is a climate-sensitive zoonotic pathogen which spreads from birds to humans via mosquitoes.^40^, ^41^ In Europe, the pathogen has become endemoepidemic with a large increase in the intensity, frequency, and geographical expansion of West Nile virus outbreaks co-occurring with more suitable climate conditions.^42^, ^43^ In 2022, the number of locally acquired human West Nile virus cases reported was 1340, including 104 deaths.^44^, ^45^ High temperatures, induced by climate change, accelerate capacity for West Nile virus vectors, such as *Culex* mosquitoes, consequently exacerbating virus transmission. Increasingly dry conditions also create adaptive responses in animals and humans leading to more contact between birds and mosquitoes around water sources.^46^, ^47^

**Figure 3:**
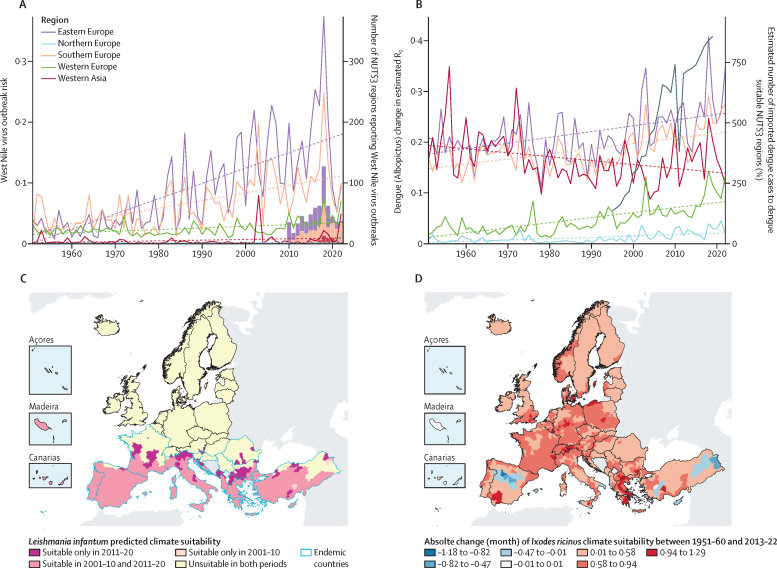
Climatic suitability for West Nile virus, dengue, *Leishmania infantum*, and *Ixodes ricinus* ticks in Europe (A) West Nile virus outbreak risk by European subregion between 1950–2022, calculated at the NUTS3 level. Bars represent the number of NUTS3 regions reporting West Nile virus transmission for each subregion (2010–22). (B) Estimated reproduction number (R_0_) for dengue by European subregion over 1951–2020. The black line shows the estimated number of yearly dengue cases imported from dengue-endemic regions to transmission suitable NUTS3 regions in Europe (1995–2019). (C) Climatic suitability for *Leishmania infantum* by NUTS3 regions. Pink-shaded areas represent suitability change between 2001–10 and 2011–20. Blue borders represent countries that are currently considered endemic for leishmaniasis. (D) Absolute change in the mean number of months with optimal climatic conditions for *Ixodes ricinus* nymph feeding activity comparing 1951–60 and 2013–22.

By using a supervised machine learning classifier on data of West Nile virus presence or absence (response) with climatic (temperature and precipitation) and socioeconomic predictors,^48^, ^49^ a steady and increasing trend of West Nile virus outbreak risk was estimated between 1951 and 2022, primarily driven by climate factors (appendix 4 pp 61–64). The relative increase in West Nile virus outbreak risk in 2013–2022 compared with a 1951–60 baseline was 256% (figure 3A), with the highest relative risk increases seen in eastern Europe (516%) and southern Europe (203%). The absolute outbreak risk for West Nile virus was highest in eastern, southern, and western Europe in 2013–22, while highest in southern and eastern Europe in 1951–60.

#### Indicator 1.3.3: climatic suitability for dengue, chikungunya, and Zika viruses

Increased human mobility combined with rising climatic suitability contributes to a surge in European arboviral disease emergence.^11^, ^50^, ^51^ The occurrence of sporadic autochthonous dengue outbreaks in Spain, Italy, and France has exposed Europe's susceptibility to these arboviruses.^52^ In 2022 alone, a total of 65 autochthonous dengue cases (nine separate transmission instances) were reported in France,^53^, ^54^ surpassing all annual cases recorded during 2010–21. Insufficient preparedness could exacerbate the adverse health consequences associated with dengue outbreaks.^55^

The first part of this indicator uses a mechanistic model to estimate the basic reproduction rate (R_0_) and length of transmission season for dengue, chikungunya, and Zika viruses by combining information on temperature, rainfall, daylight, mosquito abundance, and human population density (appendix 4 pp 65–70).^56^, ^57^ The relative increase in dengue outbreak risk was 55·94% in Europe when comparing 2013–22 with 1951–60, with the greatest absolute increase observed in southern Europe (6·88%), followed by eastern Europe (6·65%; figure 3B). The absolute risk of dengue outbreaks by *Aedes albopictus* in northern Europe increased by 1·7% and in western Europe by 6·10%, and decreased by 2·89% in western Asia, between the beginning and end of the last decades. Similar patterns were observed for chikungunya and Zika virus. The duration of dengue transmission season extended by 0·4 months in 2013–22, compared with the 1951–60 baseline.

The second part of this indicator estimates the annual number of people infected with dengue moving from dengue-endemic regions into locations in Europe where conditions are suitable for dengue transmission (appendix 4 pp 71–73). Total imported cases have increased by 176·8% across Europe in 2009–19 compared with 1995–2004. The highest relative increase in imported cases is observed in northern Europe at 194·17% followed by southern Europe at 173·73%.

#### Indicator 1.3.4: climatic suitability for malaria

Although malaria was eradicated 50 years ago in Europe,^58^ there have been sporadic local transmission events and cases reported by travellers,^59^ with around 4856 malaria cases reported in 2021 (99·7% of which were travel related).^60^ Climate change is expected to increase risk for local malaria transmission by enhancing favourable environmental conditions for the mosquito vector. Using a threshold-based model that incorporates accumulated precipitation, relative humidity, temperature, and suitable land cover classes (ie, rice fields, permanently irrigated land, and sport and leisure facilities), the first part of this indicator estimates the number of months with suitable conditions for *Plasmodium vivax* transmission, the formerly endemic malaria pathogen in Europe (appendix 4 pp 74–82).^61^, ^62^, ^63^ The second part of the indicator uses the methodology of indicator 1.3.3 to estimate malaria importation events at NUTS3 level. While all subregions witnessed an increase in months suitable, western and eastern Europe displayed the highest absolute increases of 0·34 months and 0·22 months respectively, between 1951–60 and 2013–22. During 1951–2022, there was a consistent increase in transmission suitability in non-urban areas, particularly in regions characterised by medium levels of social deprivation. Nationally, countries such as Liechtenstein, Slovenia, and Switzerland showed the largest increases in transmission suitability. Conversely, Greece, North Macedonia, and Romania among other countries, had reductions in the length of the suitable seasons. Simultaneously, there has been a consistent rise in the number of malaria importation events from endemic regions to areas with suitable conditions over the past decade.

#### Indicator 1.3.5: climatic suitability for leishmaniasis

Leishmaniasis is a climate-sensitive zoonotic disease caused by *Leishmania* parasites and transmitted by female Phlebotomine sandflies. Cutaneous (most common and causes skin sores) and visceral (rarer, systemic, and with high fatality) leishmaniasis, caused by *Leishmania infantum*, are endemic in parts of Europe, with the estimated number of cutaneous and visceral leishmaniasis cases amounting to 1100–1900 per 100 000 in south-eastern Europe and 10 000–17 000 in western Europe.^64^ However, notification of cases is not compulsory, and under-reporting and imported cases are common.^65^, ^66^, ^67^ Sandfly species tend to be located in regions with periodic temperatures above 15°C, although optimum climatic conditions for vector activity^68^, ^69^ and parasite development^70^, ^71^ vary between species. Under future climate change, many sandfly species are expected to further expand their range in Europe; geographical extensions into northern regions and higher altitudes are already reported.^72^

A nested machine learning modelling approach was applied to predict the climatic suitability for leishmaniasis across NUTS3 regions (appendix 4 pp 83–92). An initial set of models were fitted to presence and absence data for each sandfly vector species (ie, *Phlebotomus perniciosus, P ariasi, P perfiliewi, P neglectus,* and *P tobbi*) using bioclimatic indicators, land cover, and elevation. The outputs were used as covariates together with selected bioclimatic indicators to fit further models to two decadal periods (2001–10 and 2011–20) assessing spatiotemporal changes in the climatic suitability.

The number and spatial distribution of NUTS3 regions predicted to be suitable for leishmaniasis increased considerably from 2001–10 (55% of NUTS3 regions in endemic countries) to 2011–20 (68%), with new localities identified as suitable north of the historical endemic zone (figure 3C). In non-endemic zones, four previously unsuitable NUTS3 regions in Austria and Germany are predicted to become suitable for transmission in the later decade. Increases were predominantly observed in parts of southern, western, and eastern Europe, and in western Asia, while remaining absent from northern Europe. Bulgaria, France, Italy, and North Macedonia displayed the most noticeable increases in the number of suitable NUTS3 regions in 2011–20 compared with the previous decade.

#### Indicator 1.3.6: climatic suitability for ticks

Although there are multiple tick species associated with the transmission of pathogens, *Ixodes ricinus* ticks are the dominant European vectors, including for *Borrelia burgdorferi* causing Lyme disease and tick-borne encephalitis—two of the most prevalent vector-borne illnesses in the northern hemisphere.^73^, ^74^ This indicator uses a threshold-based approach to estimate the number of months with optimal climatic conditions (ie, temperatures ranging from 10–26°C and relative humidity >45%) for *I ricinus* nymph feeding activity (appendix 4 pp 93–99).^75^ Furthermore, environmental suitability, on the basis of reported tick observations, is incorporated to establish whether ticks could be present in a specific land cover class. In total, 1455 (96%) of 1514 NUTS3 regions increased in suitability during 2013–22 compared with 1951–60 (figure 3D). Overall, eastern Europe and western Asia showed the highest suitability, particularly in rural districts and areas characterised by high social deprivation. Notably, western Asia and eastern Europe witnessed the most substantial increase in months suitable when comparing 1951–60 with 2013–22 (figure 3D), extending the period of suitable activity by 0·68 months for 1951–60 and 0·58 months for 2013–22. These findings highlight a rising trend in tick climatic suitability, amplifying the exposure to feeding ticks, and involving the potential transmission of associated pathogens.

### 1.4: allergens

#### Indicator 1.4.1: allergenic trees

Allergenic pollen are substantially affected by weather conditions,^76^ with climate change leading to systematic shifts in flowering seasons of most plants (ie, start, end, duration, and severity of season).^77^ These changes impact the severity of allergic diseases (eg, allergic rhinitis, allergic rhinoconjunctivitis, and bronchial asthma), which are estimated to be prevalent in at least 40% of the European population.^78^ This indicator monitors the seasonal timing and severity (daily pollen per m^3^) for birch and alder (1980–2022) and olive (1990–2022) by analysing the European pollen reanalysis,^79^ which calculates plant development during spring and combines it with atmospheric transport modelling and pollen monitoring data (appendix 4 pp 100–110).^80^, ^81^, ^82^, ^83^

**Figure 4:**
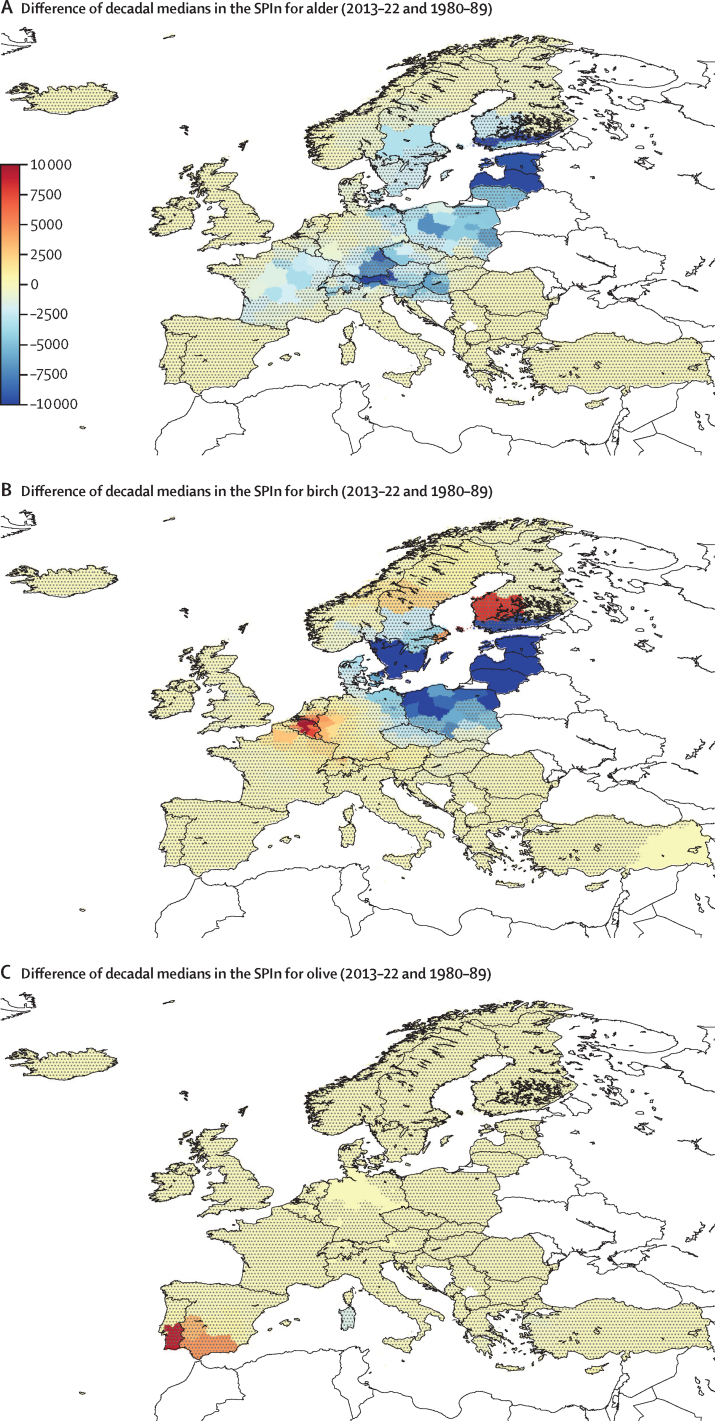
Difference between decadal medians in the SPIn in Europe Difference between decadal medians in the SPIn (pollen per day per m^3^) for (A) alder, (B) birch, and (C) olive trees in Europe at NUTS2 level, comparing 2013–22 with 1990–99. Dot-shaded areas do have not statistically significant trends (p>0·1). Dot-free areas had clinically relevant seasons that occurred less than five times between 1990–99 and 2013–22. SPIn=seasonal pollen integral.

Comparison of the decadal averages for 2013–22 with 1980–89 and 1990–99 for olive shows diverse changes in seasonal severity of birch, alder, and olive across Europe, with regional upward and downward trends, in contrast with a widespread belief of ever-increasing pollen abundance (figure 4). All three trees tend towards earlier flowering, especially in mountains (ie, Alps, Balkans, and Scandinavian ridge), where the season start over a month earlier in 2022 than 33 years ago (1990). Both the start and end of the pollen season have shifted, while the season duration remained nearly the same across most of Europe. The model suggested a small shortening of the alder season in western Europe, but lengthening in the east, whereas the birch and olive seasons remained practically the same.

### 1.5: food and water

#### Indicator 1.5.1: food security and undernutrition

In Europe, food insecurity has been linked to negative health outcomes, including a reduced ability to manage chronic disease and worsening child health.^84^ Some demographic groups are at higher risk of being food insecure, including women, older people, people with existing health conditions, and low-income households.^85^, ^86^ A move towards more plant-based diets could improve food security, reduce emissions (indicator 3.4), and increase carbon sequestration.^87^

The Food and Agriculture Organization (FAO) Food Insecurity Experience Scale (FIES) tracks eight dimensions of access to food, from not being able to eat a sufficient variety of food to not eating for a whole day.^88^ In Europe in 2021, 16·3% of those responding to the FIES survey reported eating only a few kinds of food, 14·4% reported being unable to eat healthy and nutritious food, and 10·6% reported eating less than they thought they should. New to the 2024 report, this indicator combines FIES and income data with frequency of heatwave days and drought months (SPEI-12) from ERA5-Land in 37 countries. Using a time-varying panel regression,^89^, ^90^ the indicator tracks the effects of increasing frequency of heatwaves and droughts on the prevalence of moderate or severe food insecurity (appendix 4 pp 111–13).

In 2021, nearly 60 million people had moderate or severe food insecurity in Europe. 11·9 million (95% CI 11·3–12·5 million) of these can be attributed to a higher number of heatwave days and drought months, compared with the average during 1981–2010. A higher number of heatwave days was associated with 1·12 (1·07–1·17) percentage-points higher (moderate or severe) food insecurity in 2021; while increasing frequency of droughts resulted in food insecurity being 0·47 (0·44–0·50) percentage-points higher, both compared with the 1981–2010 average. Low-income respondents have a significantly higher risk of having food insecurity compared with the median-income respondents.

### Conclusion

Climate change is contributing to worsening multidimensional health impacts across Europe, with overall increased upward trends observed across indicators. While not all indicators were able to incorporate aspects of inequality,^91^ the effects are unevenly distributed, with regional differences often reflecting sociodemographic differences and marginalisation.^2^, ^92^ Sub-regionally, southern Europe tends to be more affected by heat-related illnesses, wildfires, food insecurity, drought, and leishmaniasis, whereas northern Europe is equally or more affected by *Vibrio* and ticks. Within countries, indicators show differential impacts among socially at-risk groups; for example, differences in heat-related mortality among women and men emphasising that inequalities should be recognised in the design and roll-out of climate change adaptation strategies.

## Section 2: adaptation, planning, and resilience for health

Climate change adaptation refers to the process of preparing for or anticipating to reduce susceptibility and exposure of human populations, and the implementation of interventions to minimise the adverse outcomes when impacts do occur. Public health adaptation measures can be implemented at different scales and can include actions such as the maintenance and enhancement of crucial infrastructure,^93^ enhanced disease surveillance to track climate-sensitive diseases and inform interventions (eg, a heat-health warning system),^94^ and using outreach and public campaigns to empower communities and build resilience.^95^

Indicators in this section track climate risk assessments at international and city levels, and cross-sectoral collaboration for climate adaptation and the implementation of climate-informed surveillance and health early warning systems (HEWS). The indicators also track adaptation strategies used to prevent harmful exposure to high temperatures, such as air conditioning and more sustainable strategies including green space and other nature-based solutions (appendix 4 pp 114–17).

### 2.1: adaptation, planning, and assessment

#### Indicator 2.1.1: national vulnerability and adaptation assessments

Climate change and health vulnerability and adaptation assessments support countries in understanding health risks from current and future climate hazards, identifying gaps in current policies and programmes, evaluating which populations are most at risk, and identifying effective adaptation interventions to respond to climate change-related health risks.^96^ Using data from the 2021 WHO Health and Climate Change survey (appendix 4 pp 118–20), ten (45%) of 22 countries reported having conducted vulnerability and adaptation assessments by 2021.^96^ Only two (20%) of ten assessments reported resulted in the development of new or the revision of existing health policies or programmes, and one (10%) assessment strongly influenced the allocation of human and financial resources to address the health risks of climate change. Furthermore, only two (9%; Germany and North Macedonia) of 22 countries reported that climate change and health considerations were included in COVID-19 recovery plans.

#### Indicator 2.1.2: national adaptation plans for health

Although many countries have collaborations on health and climate change through multi-stakeholder mechanisms,^11^ only ten (45%) of 22 assessed countries have formal agreements via a memorandum of understanding between ministry of health (MoH) and any other health-determining sectors, based on the 2021 WHO Health and Climate Change survey (appendix 4 pp 118–20).^96^ Encouragingly, ten (45%) countries had an agreement between MoH and the environment sector and nine (41%) had agreements with meteorological and hydrological services, which might increase the uptake of climate information to assist decision-making for health surveillance and use of early warning systems (eg, meteorological observations and forecasts to inform about hazardous weather conditions).^97^ Temperature and precipitation can alter water quality and quantity and influence waterborne diseases. Therefore, formalising agreements between MoH, environment, and the water, sanitation, and hygiene sector is important to tackle interconnected challenges, which can be done with integrated assessments, and planning and adaptation strategies for climate change related-health risks and water-related vulnerabilities.

#### Indicator 2.1.3: city-level climate change risks assessments

With Europe having one of the world's highest densities of urban settlement, city-level adaptation and mitigation is crucial to build climate resilience. Using data from the Carbon Disclosure Project and the International Council for Local Environment Initiative,^98^ this indicator shows that in 2022, 149 (81%) of 185 responding European cities reported to have conducted a climate risk assessment, 12 (6%) reported that an assessment was in progress, and 22 (12%) reported that an assessment will be undertaken in the next 2 years (appendix 4 p 121). This indicator illustrated a slight percentage increase compared with 2021, when 150 (76%) of 197 cities conducted climate assessments. Most prominently identified climate hazards that impact health included extreme heat, heat stress, urban flooding, heavy precipitation, and air pollution, and most mentioned health issues driven by climate hazards were health-related illnesses in 141 (76%) cities, exacerbation of respiratory diseases in 88 (48%), direct physical injury and death due to extreme events in 82 (44%), mental health impacts in 69 (37%), and overwhelming of health service provision due to increased demand in 55 (30%) cities. Older people, at-risk health groups, children and youth, low-income households, outdoor workers, marginalised communities, women and girls, frontline workers, and Indigenous peoples were identified as most at risk. The absence of financial capacity was stated by 39 (21%) cities, expertise and technical capacity by 23 (12%), and political priority by 16 (9%) cities were most often mentioned to limit cities' ability to address identified climate-related health issues.

### 2.2: adaptation delivery and implementation

#### Indicator 2.2.1: climate information for health

Given the impact of weather and climatic conditions on disease, climate-informed health surveillance systems and HEWS can enhance health system capacity to prepare for increasing climate-sensitive diseases risks. Data from the WHO Health and Climate Change survey (appendix 4 pp 118–20)^96^ suggest that most of the 22 European reporting countries have health surveillance systems for specific health outcomes. However, few have health surveillance systems that incorporate climate information (ie, climate-informed surveillance systems): waterborne diseases and other water-related outcomes (four [22%] of 18 are climate-informed), vectorborne diseases (six [35%] of 17 systems), zoonoses (four [24%] of 17), airborne and respiratory illnesses (six [40%] of 15), malnutrition and foodborne diseases (two [14%] of 14), non-communicable diseases (five [36%] of 14), heat-related illnesses (ten [91%] of 11), injury and mortality from extreme events (eight [73%] of 11), mental and psychosocial health (three [30%] of ten), and impacts on health-care facilities (one [20%] of five). In contrast, a moderate number of HEWS are climate-informed, such as HEWS for heat-related illness in 12 systems, injury and mortality from extreme events in 11, waterborne diseases in ten, and vector-borne diseases in ten.

#### Indicator 2.2.2: green space

Green spaces can improve health by providing space for physical activity, reducing air and noise pollution, reducing temperatures, increasing social contacts, and relieving psychophysiological stress.^99^ Thereby, urban green space can be part of nature-based adaptation solutions (appendix 4 pp 122–29) with economic, social, and health co-benefits. However, due to spatial inequalities of blue and green spaces, disadvantaged people living in deprived areas have less access than those living in more affluent areas and tend to be disproportionately exposed to environmental hazards.^100^, ^101^

This indicator describes normalised difference vegetation index (NDVI) changes during 2000–22 at the NUTS3 level and disaggregates by levels of social deprivation at the NUTS2 level in Europe. On average, population-weighted NDVI increased by 2% during 2000–22. Some areas saw a statistically significant increase of more than 0·1 in the population-weighted greenness, particularly near the borders between Romania, Serbia and Hungary, and Albania. Reductions were seen in southern Norway and Sweden, Belgium, Iceland, Lithuania, and parts of Germany, Austria, and Slovakia. The absolute NDVI increase was larger in areas with higher social deprivation. Changes in the indicator were largely explained by population change rather than actual NDVI increase over time (appendix 4 pp 122–29).

#### Indicator 2.2.3: air conditioning benefits and harms

Rising temperatures are increasing the use of carbon-intensive active cooling systems, such as residential air conditioning. While effective to prevent health-related illnesses, air conditioning contributes to greenhouse gas emissions, power outages, air pollution, urban heat island effects, peak electricity demand, and energy poverty, which result in substantial co-harms.^102^ Furthermore, as many marginalised and low-income populations are unable to afford indoor thermal comfort, reliance on air conditioning over the use of other more accessible and sustainable cooling interventions^103^ can increase heat health-related inequalities within Europe.

This indicator tracks the proportion of European households using air conditioning and associated electricity use and CO_2_ emissions during 2000–21 (appendix 4 p 130). In 2021, air conditioning provided cooling in 16% of European households, consuming about 159 Terawatt-hours of electricity and producing 45 megatonnes (Mt) CO_2_ emissions—approximately the same as the total CO_2_ emissions of the whole of Bulgaria in 2021.^104^

Reducing cooling load and implementing sustainable cooling mechanisms (eg, passive cooling by natural ventilation, green roofing, improved shading and glazing, radiant cooling, and evaporative cooling) tailored to local contexts are important to prevent the over-reliance on carbon-intensive air conditioning, while simultaneously protecting thermal comfort and health of European populations.^102^, ^103^

### Conclusion

European health systems remain poorly adapted to climate change-related health impacts as reflected by the lack of execution of National Adaptation Plans, an absence of health sector integration with other health-determining sectors for climate adaptation, few vulnerability assessments conducted at a national level, and the few climate-informed health surveillance or HEWS in place. The EU Adaptation Strategy^105^ and the EU Biodiversity Strategy for 2030^106^ emphasise the need for cities to create biodiverse and accessible urban green spaces, and many cities have started to do so; for example Barcelona with Superblocks.^107^

However, transitioning the health sector to adequate climate change adaptation and resilience requires integration of health policy with other health-affecting sectors, better adherence and enforcement of climate accords, and increased investments in tangible adaptation solutions. Furthermore, climate adaptation measures might not benefit everyone in society to the same extent. To ensure no one is excluded, equity should be an integral part of all stages of adaptation planning, implementation, and monitoring.^108^ Adequately identifying the populations most at risk and preventing the implementation of maladaptive interventions that could inadvertently reinforce or redistribute inequity among populations should be included.^109^ While some current EU and national climate policies draw attention to at-risk groups, practical implementation of equitable adaptation solutions remains scarce.^108^

## Section 3: mitigation actions and health co-benefits

Global progress on climate change mitigation has been inadequate, with the pace of change being far from what is required to meet Paris Agreement targets,^110^ and the recent COP28 calling vaguely for a transition away from fossil fuels, as opposed to a needed phase-out. Greenhouse gas emissions from the EU-27 in 2021 were only 30% lower compared with 1990,^111^ leaving a large gap to meet intermediate 2030 targets. Worryingly, continued progress towards emission reductions is not guaranteed: European greenhouse gas emissions in 2021 were 6% higher compared with 2020.^111^

Placing health at the centre of climate change mitigation offers opportunities for large health co-benefits ancillary to emission reductions. Many health co-benefits occur in the near-term, at local scale, and their beneficial effects can offset mitigation costs in the short-term, long before the beneficial effects of climate mitigation are realised.^112^ Thus, accounting for health co-benefits provides more comprehensive, accurate estimates of net mitigation policy costs and could increase political will towards ambitious mitigation policies. Potential health co-benefits might also incentivise change in individuals' behaviours and institutional policies. Communicating the direct, individual health co-benefits has been shown to motivate households to adopt low-emission behaviours.^113^ Engagement with the health co-benefits of climate change mitigation is increasing among international organisations,^114^ suggesting that this rationale for transformation is gaining traction beyond academia.

This section includes nine indicators tracking European efforts to mitigate climate change by reducing greenhouse gas emissions, and their associated health co-benefits from the reduction of air pollution-related morality to transition towards more sustainable and healthy forms of travel and diets. Two new indicators have been added to track production-based and consumption-based emissions and health-care sector emissions.

### 3.1: energy system and health

#### Indicator 3.1.1: carbon intensity of the energy system

Energy systems remain the largest single source of greenhouse gas emissions. Using data from the International Energy Agency (IEA), this indicator shows that while Europe is making some progress towards achieving net-zero emissions, its current trajectory is consistent with achieving carbon neutrality only by 2100 (appendix 4 pp 131–32). To meet the recommendations of the latest IPCC report of net-zero by 2040, emissions from Europe's energy system are required to fall at around three times the current rate (based on the trend since 2006). This reduction will need to happen even faster if fair-share emissions—taking Europe's population and historical emissions into account—are used to allocate the reductions globally.^115^ After an 8·6% reduction in 2020, emissions from fossil fuel combustion had a substantial rebound, surging by 7·1% in 2021, to 3·4 billion tonnes (Gt) of CO_2_ per year (5·4 tCO_2_ per person)—six times higher than African per-person emissions (0·9 tCO_2_), 2·7 times higher than Central and South American emissions (2·0 tCO_2_), but 2·6 times less than US per person emissions (13·8 tCO_2_).^116^ Some countries, such as France and the UK, saw increases of around 10%, in contrast to Finland and Greece where increases were less than 1%. Despite the Russian invasion of Ukraine fuelling an energy crisis, European natural gas prices returned to pre-invasion levels in 2021,^117^ reducing the short-term pressures to shift to alternative fuel sources.

#### Indicator 3.1.2: coal phase-out

In 2021, coal use increased to 13% of Europe's total energy supply compared with 12% in 2020 (appendix 4 pp 133–34), according to IEA data.^116^ After two consecutive years of annual reductions, coal use rebounded due to increased use by Germany and Poland. This surge marked the highest growth rate in at least 40 years, underscoring a concerning trend in Europe's energy mix and is driven in part by the slowing of coal phase-out due to the Russian invasion of Ukraine. Growth in renewable energy deployment partly offset the anticipated resurgence of coal in 2022,^118^ but accelerated progress to phase out coal remains essential to meet Europe's climate targets and protect people from air pollution and its related morbidity and mortality.

#### Indicator 3.1.3: renewable and zero-carbon emission electricity

The share of electricity supplied by renewable energy has grown substantially over the past decade, but only represents 22·8% of the total energy consumption in Europe.^119^ From 2014 to 2021, the proportion of electricity supplied by renewables nearly doubled, increasing from 10% to just under 20% of total energy consumption.^116^ Recognising the need for further progress, the EU has set a target of 40% renewable energy in the overall energy mix by 2030, with plans to revise it to 45% under the REPowerEU initiative.^120^ The transition towards net-zero emissions is driven by zero carbon energy sources, including wind, solar, photovoltaic, hydro, and nuclear. Currently, these sources account for around 20% of the total energy supply in Europe.^116^ Approximately 50% of the total electricity supply is derived from energy sources with zero carbon emissions, with renewables such as solar, wind, and photovoltaic energy contributing 19% of the total (appendix 4 pp 135–36).

### 3.2: air pollution and health co-benefits

#### Indicator 3.2.1: premature mortality attributable to ambient fine particles

Exposure to fine particles (ie, PM_2·5_) is a risk factor for premature mortality, respiratory and cardiovascular disease, adverse pregnancy outcomes, cancer, diabetes, and neurological disorders.^121^ Worse European air quality is typically seen in more deprived (NUTS3) regions.^122^ This indicator tracks changes in premature mortality (ie, advanced death by any amount of time) attributable to PM_2.5_ from the combustion of coal, liquid, and gaseous fossil fuels across the residential, power generation, and transport economic sectors (appendix 4 pp 137–41).

**Figure 5:**
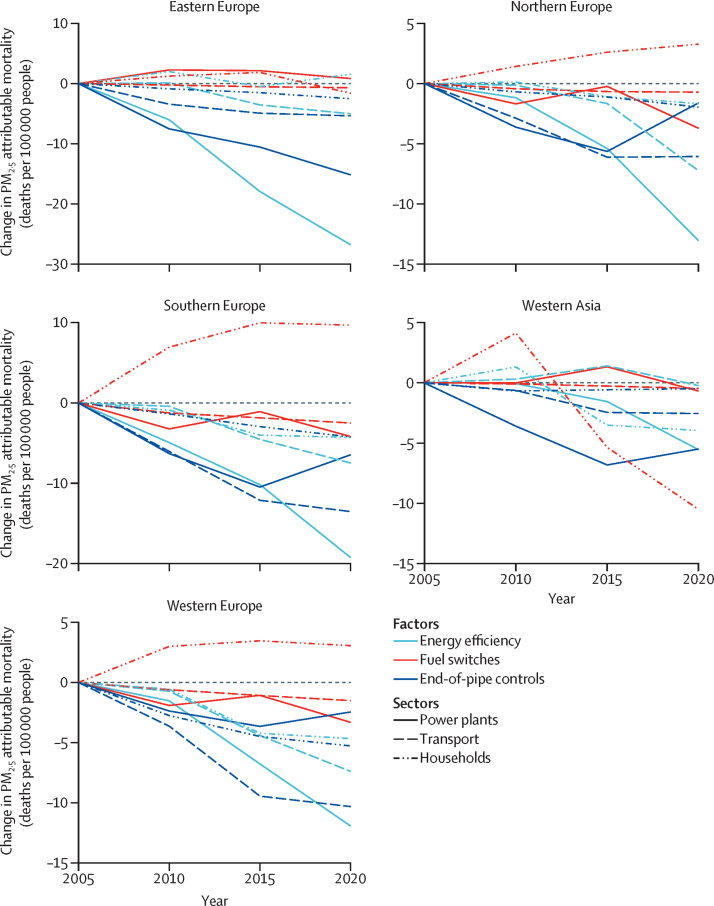
Premature mortality attributable to ambient fine particles in Europe Factors (structural changes, fuel switches, and end-of-pipe controls) contributing to mortality (annual attributable deaths per 100 000 people) due to PM_2·5_ by region and economic sector (power plants, transport, and households), calculated in 5-year steps. This indicator uses the Greenhouse Gas and Air Pollution Interactions and Synergies model to combine bottom-up emission calculations with atmospheric chemistry and dispersion coefficients using mortality data (Eurostat and UN World Population Prospects 2017), energy consumption by fuel and sector data (Eurostat and IEA energy statistics), agricultural activity data (FAOSTAT), and fertiliser use data (IFASTAT).

This indicator shows that during 2005–20, PM_2·5_ attributable deaths from fossil fuel combustion decreased by 59% in Europe overall; 74% for power; 11% for residential; and 48% for transport sectors. We also analysed the factors behind these trends (figure 5).^123^ The factors driving change varied strongly across sectors and European subregions. In all sectors, energy demand decoupled (ie, was not directly linked) from macroeconomic drivers (gross domestic product [GDP] for power, population for residential, and per-capita GDP for transport sector), which was attributed to structural changes in the economy (eg, a shift towards service-based economies) and energy efficiency improvements. This development was most evident in eastern Europe. The influence of switching to fuels with lower emissions (fuel switches) was heterogeneous across European subregions and sectors. While coal phase-down in the power sector led to decreased emissions and associated health impacts, increased use of biomass in households in northern, western, and southern Europe increased PM_2·5_ levels and associated mortality. In transport, fuel switches were mainly between gasoline and diesel, leading to moderate changes in either direction, while the effect of electrification was not prominent. Much of the ambient PM_2·5_ decrease was due to improved air pollution control technologies that decreased air pollution, but not greenhouse gas emissions. These findings highlight the need for appropriate incentives and policy measures to prevent trade-offs when tackling air pollution and greenhouse gas emissions in parallel.

#### Indicator 3.2.2: production-based and consumption-based attribution of CO_2_ and PM_2·5_ emissions

When countries report emissions or set reduction targets, production-based emissions (ie, emissions occurring only within the country or territory) are usually used. However, many European countries outsource environmental pressures (including greenhouse gas emissions, air pollution, water consumption, and ecotoxicity) and negative climate and health impacts related to European consumption of goods and services occur elsewhere.^7^ The outsourcing of environmental pressures highlights an inherent environmental and health justice problem: those where health and environment is most affected are not those driving the causal consumption. A more appropriate and just way of assessing emissions (and pollution) would be to assign emissions to the consuming territory (ie, consumption-based emissions).

This indicator uses a multi-region input–output model, to quantify consumption-based and production-based CO_2_ and PM_2·5_ emissions across all sectors (appendix 4 p 142). In 2021, consumption-based emissions exceeded production-based emissions by one percentage point for CO_2_ and 1·6 percentage points for PM_2·5_. The emissions embodied in Europe's imports accounted for 19·2% of its consumption-based CO_2_ emissions and 30·8% of its consumption-based PM_2·5_ emissions, ranking highest among all regions.

### 3.3: sustainable and healthy transport

Switching to low-emission vehicles and active transport (eg, walking, cycling, and wheeling) is essential to reduce transport emissions and create health co-benefits, such as reductions in road-traffic injuries, sedentary behaviour, and air and noise pollution.^124^, ^125^ In 2022, there was a notable increase in electric vehicle sales in Europe, with electric cars accounting for approximately 20% of the 9·5 million vehicles sold.^126^ Well-designed public and active travel infrastructure is essential to minimise socioeconomic inequities in access to sustainable transport, and to ensure health and social co-benefits are maximised across all population sub-groups.^127^

Using IEA data, this indicator reveals a substantial shift in transport mode during the COVID-19 pandemic, with a 5% increase in car usage observed from 2019 to 2020 (appendix 4 pp 143–44). This shift led to a decline in train and bus use, with car trips comprising 87·2% of all journeys in 2020 compared with 82·5% in 2019, most probably reflecting the perceived safety of private vehicles during the pandemic. The European Commission has responded with a plan aimed at enhancing multimodality by improving and expanding public transport systems while concurrently developing cycling and walking infrastructure.^128^

### 3.4: food, agriculture, and health

#### Indicator 3.4.1: lifecycle emissions from food demand, production, and trade

Feeding growing human populations while remaining within safe environmental limits, securing future population health, and facilitating fair and equitable livelihoods requires most European countries to radically reduce animal-based food consumption and shift towards less-polluting, less-processed, resource-efficient, and healthy plant-based diets—adapted to contextual factors and cultural values.^129^, ^130^ Using FAO data with lifecycle-emission estimates, this indicator estimates that European food-related emissions was reduced by only 1% (16 MtCO_2_-equivalent [eq]) between 2010 and 2020, with the greatest reductions in southern Europe (–9%, 40 MtCO_2_-eq) and eastern Europe (–0·4%, 3 MtCO_2_-eq), and increases in western and northern Europe (appendix 4 pp 145–46). In total, European food demand accounted for 2·5 tCO_2_-eq per person (total 1·85 GtCO_2_-eq) in 2022; with animal-sourced food predominantly responsible and representing 66–70% more per-person emissions from food demand than low-income and middle-income countries. Sweden's food demand-related emissions (28 MtCO_2_-eq) exceeded their territorial emissions (18 MtCO_2_-eq), then Romania with 69% and Switzerland with 58% of their territorial emissions.

#### Indicator 3.4.2: sustainable diets

Adopting healthy diets with low environmental effects is an important mitigation strategy that can deliver substantial health co-benefits. Energy-dense but nutrient-poor diets have caused increasing trends in non-communicable diseases, while co-existing with undernutrition.^131^ Using food consumption estimates with epidemiological models, this indicator estimates that in 2020, 2·48 (95% CI 2·59–2·36) million deaths were attributable to imbalanced, non-sustainable diets (ie, diets with increased dietary health risks, such as those with too much red meat or too few fruits and vegetables) in Europe (appendix 4 pp 147–54). The number of deaths attributable was similar among women (1·27 million [1·21–1·32]) and men (1·21 million [1·14–1·27]). Most deaths were attributable to diet composition; 274 000 (11%) eating too much red and processed meat, or 296 000 (12%) eating too few legumes, 308 000 (12%) fruits, 272 000 (11%) vegetables, or 220 000 (9%) consuming too few nuts. Eastern Europe had the greatest burden of diet-related deaths (1·40 million; 4·6 deaths per 1000 people). As unhealthy eating patterns and diet-related health issues have a socioeconomic gradient,^131^ transforming food systems and diets requires equitably addressing the (structural) components that prevent access to healthy sustainable foods. Such solutions can include dedicated food policies that support communities in eating sustainably and healthily, dietary guideline changes, community-based health promotion programmes, and affordable prices.

### 3.5: health-care sector emissions and harms

Health systems are a substantial source of greenhouse gas emissions and air pollution. The global health-care sector was estimated to contribute to around 4·6% of global greenhouse gas emissions in 2020,^114^ with countries such as the Netherlands estimating that about 4–8% of their carbon footprint is due to the health-care sector.^132^, ^133^ The largest health-care emissions are related to the supply chain, including medical product manufacturing, transport, use and disposal, and energy.^134^ Following the duty of doing no harm, health-care institutions should lead the way towards decarbonisation.^135^ For example, in 2020, the UK's National Health Service became the first national health system to commit to carbon net-zero. One year later at the COP26 Health Programme, a further 50 countries committed to create low-carbon, sustainable, and climate-resilient health systems, with 14 countries setting targets of net-zero emissions by 2050.^136^

**Figure 6:**
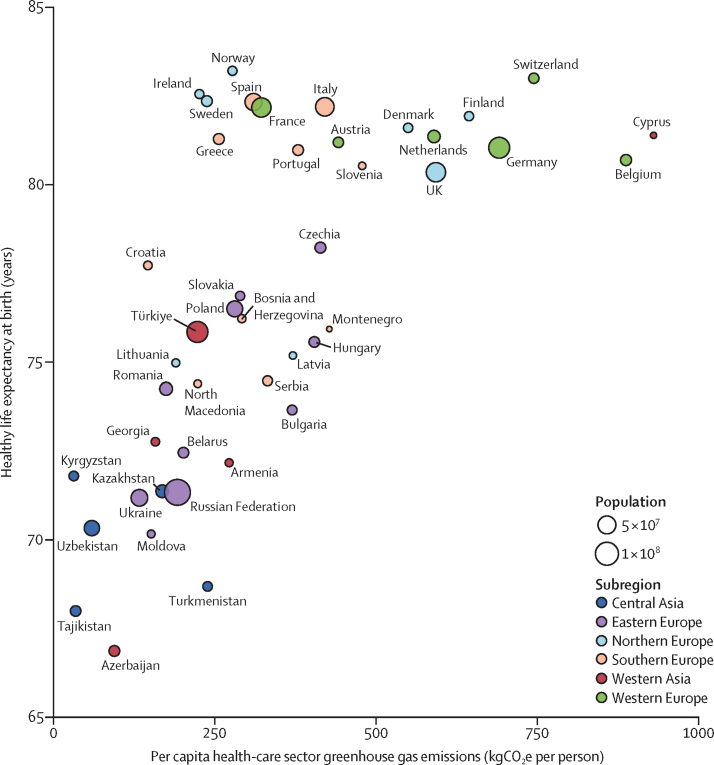
Health-care sector emissions in Europe National greenhouse gas emissions per person (kg CO_2_e per person) from the health-care sector against the healthy life expectancy at birth in 2020 (World Bank) by European subregions. Point size is defined by the size of the population.

This indicator monitors both direct and indirect health-care sector emissions using a multi-region input–output model combined with national health-care expenditure data from WHO (appendix 4 p 155). The indicator further estimates the disability-adjusted life years lost due to emissions of PM_2.5_ and ozone precursors related to health-care activities. In 2020, it was estimated that the health-care sector of the WHO European region contributed approximately 330 MtCO_2_e (356 kgCO_2_e per person) in greenhouse gas emissions. Despite several European countries taking action to reduce their health-care emissions, there was a 3% per capita increase compared with 2010. Of the 52 health systems analysed, Malta had the highest emissions per person (3380 kgCO_2_e per person)—more than ten times the emissions per person from Kyrgyzstan (31·4 kgCO_2_e per person). However, high-quality health care (using life expectancy as a proxy) can be achieved with lower per capita emissions (figure 6). Regionally, air pollution related to European health care is estimated to result in a total of 540 000 disability-adjusted life years in 2020, predominantly caused by health care-associated emissions from the Russian Federation (14%) and Germany (13%).

### Conclusion

The solutions to the climate crisis can bring considerable near-term health co-benefits. However, careful design of mitigation measures is essential to minimise the possible adverse health impacts, such as increased exposure to indoor air pollution and mould from decreased ventilation because of building energy efficiency measures, or increased road traffic injury among cyclists resulting from shifts to active travel modes lacking safe infrastructure. If equity and justice are considered, climate action can not only offer a fair and healthy environmental transition, but also reduce inequities in key health impact pathways, including air pollution, physical activity from active transport, and healthy diets, between and within countries. Alongside the economic and social co-benefits of mitigation (eg, job creation in the green economy; improved access to clean, affordable, and secure energy; and lower energy poverty), the sizable health co-benefits that can be realised in Europe and beyond provide strong support for a just transition to net-zero.^136^

## Section 4: economics and finance

The economic costs of climate change are expected to be substantial, but uncertain, with some emission scenarios pointing to high economic costs, including increased health-care costs and loss of labour productivity due to heat stress.^137^, ^138^ Actions to shift to low-carbon economies are likely to have immediate economic, social, and health benefits that outweigh the costs of inaction.^139^, ^140^, ^141^ For example, transforming land and food systems to focus on healthy sustainable diets, productive and regenerative agriculture, and protecting and restoring nature is estimated to cost about US$350 billion a year up to 2030, while the gains from these investments are estimated to amount up to $5·7 trillion with avoided health costs, more efficient agriculture, and the creation of carbon markets.^142^ This section explores health-linked economic impacts of climate change and the economic dimension of the transition to zero-carbon economies.

### Indicator 4.1: health-linked economic impacts and mitigation of climate change

#### Indicator 4.1.1: economic losses due to weather-related extreme events

Due to climate change, the intensity, frequency, timing, duration, and spatial extent of extreme weather and climatic events are changing. The direct impacts of these extreme events on human health (eg, injury or death) are further compounded by disruption of infrastructure, public service provision, and impacts on the socioeconomic determinants of health, particularly in at-risk regions. This indicator uses Swiss Re data to track economic damages (insured and uninsured), for example to infrastructure and vehicles, resulting from exposure to weather-related extreme events during 2010–22 (appendix 4 pp 156–58).

In 2022, economic losses due to weather-related extreme events were estimated to be €18·7 billion. These losses represented 0·08% of Europe's GDP, and 44·2% (€8·2 billion) were uninsured. The average annual losses in Europe during 2018–22 decreased slightly to €24·2 billion from €26·1 billion for 2010–14, while the percentage of uninsured losses decreased to 59·6% from 66·5%. In northern Europe, an average of only 24·2% of losses were uninsured for 2018–22, while an average of 77·5% of losses were uninsured in southern Europe and 80·4% in eastern Europe.

#### Indicator 4.1.2: change in labour supply

Increasing heat stress due to climate change is directly harming the health of workers, especially those employed in outdoor sectors, such as agriculture, mining, and construction, but also indoor workers without access to cooling.^143^, ^144^, ^145^ There is clear evidence that heat stress negatively affects labour supply, productivity, and capacity in most countries across the globe,^146^, ^147^ which could further affect health outcomes by reduced GDP, incomes, and public-health expenditure. This indicator tracks the impact of temperature on labour supply (number of working hours) for highly exposed outdoor occupations (ie, agriculture, forestry, mining and quarrying, and construction) by combining NUTS2 labour supply data with ERA-5 Land temperature and precipitation data (appendix 4 p 159).

**Figure 7:**
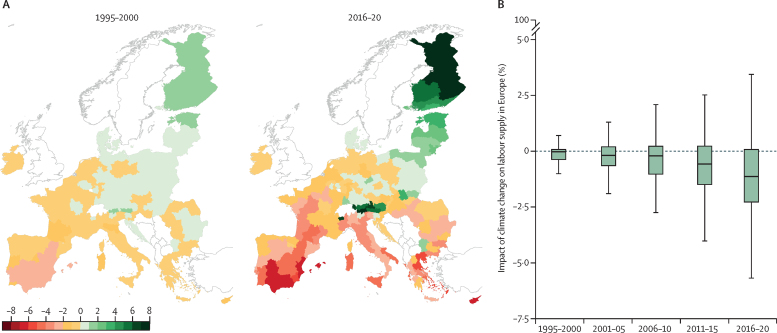
Economic impacts of climate change in Europe (A) Change in high-exposure labour supply (%) in Europe due to temperature change; counterfactual analysis for each time-period compared with the long-term mean of 1965–94. (B) Percentage change in the number of working hours (weighted by total number of working hours in 2020) due to change in temperature compared with the baseline period of 1965–94.

The association between temperature and labour supply is non-linear, with the number of productive working hours in the high-exposure sectors peaking at an annual mean temperature of 9·9°C.^11^ The non-linear relationship suggests that a temperature increase beyond the optimum has already reduced labour supply in warmer areas of Europe, whereas in relatively colder European regions, labour supply benefited from warming. Compared with 1965–94, the average number of working hours per person per year in 1995–2000 was 0·22% lower (equivalent to 4 hours per person per year) than it would have been if temperatures had not increased from this baseline average. During 2016–20, labour supply in high-exposure sectors was 1·05% lower (just under 17 hours per worker per year) due to temperature change compared with 1965–94. The highest percentage declines in working hours are estimated to be in Andalusia and the Balearic Islands in Spain, Cyprus, and the South Aegean region in Greece. Cooler regions, such as Salzburg (Austria), South Tyrol (Italy), and north and east Finland have had gains in labour supply (figure 7A–B). Adaptation measures, including appropriately designed early warning systems and labour protections, are needed to reduce the negative health and labour impacts linked to increased heat stress.^147^

#### Indicator 4.1.3: impact of heat on economic activity

In most European countries, economic activity is adversely affected by increasing temperatures, which subsequently affects human wellbeing due to unemployment, reduced incomes, increased mental stress, and overall economic pressures.^146^, ^148^, ^149^, ^150^ This indicator tracks the impact of temperature anomalies (difference between current temperature and mean temperature during 1981–2010) on economic activity in Europe, measured by real GDP per capita growth at the NUTS2 level (appendix 4 pp 160–61). In 2020, GDP per capita growth in southern Europe was 0·98% (95% CI 0·97–1·00) lower due to positive temperature anomalies compared with 1981–2010 average temperatures, but only 0·106% (0·10–0·11) lower in 2001. Furthermore, the negative impacts of temperature anomalies in southern Europe have increased over time. There was no statistically significant relationship between temperature anomalies and economic activity for northern Europe.

#### Indicator 4.1.4: monetised value of unhealthy diets

By placing an economic value on mortality (ie, using the Value of a Statistical Life) related to the consumption of imbalanced diets (indicator 3.4.2), this indicator estimates that the monetised value of imbalanced diets amounted to €9·2 trillion in 2020 (appendix 4 pp 162–163). The monetised value of diet-related health was highest in eastern Europe (€3·9 trillion), followed by southern Europe (€1·5 trillion), western Europe (€2·6 trillion), and northern Europe (€1·2 trillion). For 2010–20, the value of imbalanced diets increased by more than a third (35%), with the greatest increase observed for western Europe (37%), followed by eastern Europe (35%) and southern and northern Europe (34% each).

### Indicator 4.2: economics of the transition to zero-carbon economies

#### Indicator 4.2.1: net value of fossil fuel subsidies and carbon prices

Placing adequate carbon prices (capturing externalities of greenhouse gas emissions) can internalise the costs of climate change in economic decision making and set economic incentives for transitioning to a decarbonised economy. However, many European governments continue to subsidise fossil fuels and lack carbon border adjustment mechanisms, increasing levels of health-harming emissions.^151^ This indicator estimates the economy-wide average net carbon revenues and prices by subtracting fossil fuel subsidies from carbon price revenues using data from the IEA, Organisation for Economic Co-operation and Development, the World Bank, and WHO (appendix 4 pp 164–65).

In 2020, 32 of the 53 WHO European Region countries analysed had a carbon pricing mechanisms in place: 29 of the countries had net-negative carbon prices (ie, they were providing net subsidies for fossil fuels), while only 14 countries had net-positive carbon prices (discouraging fossil fuel use). The average net-carbon price in Europe increased from –€15·7 per tonne in 2019 to –€11·4 tonne in 2020, and total net fossil fuel subsidies decreased from €90·6 billion to €61·6 billion. However, reduced subsidies reflect, to some extent, the economic slowdown caused by the COVID-19 pandemic. The median value of subsidies in countries with a net-negative carbon price was €0·70 billion. In each of the 14 countries, the net subsidies to fossil fuels exceeded €1 billion in 2020, and in each of eight countries, net subsidies exceeded 10% of national health expenditure. Progress towards phasing out fossil fuel subsidies varied considerably across Europe, with net subsidies declining in 33 countries, but increasing in ten others between 2010 and 2020.

#### Indicator 4.2.2: clean energy investment

Clean energy investment is essential for mitigating climate change and reducing the health impacts of air pollution. This indicator monitors energy investment in Europe using data from the IEA,^152^ and compares the investment in clean energy (including renewable energy; energy efficiency; electricity networks; nuclear energy; low-emission fuels; and carbon, capture, utilisation, and storage) and fossil fuels (appendix 4 pp 166–67). Clean energy investment exceeded fossil fuel investment in Europe by 261% in 2022 (€404 million compared with €112 million). Investment was 16% higher than in 2021, and 66% higher than in 2015. Fossil fuel investment grew more slowly in 2022 by 6·3% and has mostly stagnated since 2015 (2·6% increase). Energy efficiency accounted for 31% of all European energy investment in 2022, up slightly from 29% in 2021. To be on track for net-zero emissions by 2050, global clean energy investment is required to nearly triple by 2030 and fossil fuel investment needs to be reduced to less than half its current value.^152^, ^153^ As one of the major historical and current greenhouse gas emitters, Europe should continue to play a key part in delivering this transition.

### Conclusion

There is high variability in the annual economic losses from extreme weather events, with little change in the long-run average over the past two decades. Heat stress is causing an increasing loss of hours worked in the most recent period (2016–20 *vs* 1965–94 base) compared with previous periods (1995–2000 *vs* 1965–94 base), with losses predominantly in southern Europe. Simultaneously, per capita growth in GDP in southern Europe has been lower due to higher temperatures in 2020 compared with the 1981–2010 average. The monetised value associated with lives lost from imbalanced diets amounted to €9·2 trillion in 2020 and was highest in eastern Europe.

Despite these impacts, information on carbon markets and trends points to slow progress in introducing carbon pricing and removing fossil fuel subsidies. While around 60% of European countries had some pricing mechanisms, only just over a quarter had net-positive carbon prices and the average net carbon price is decreasing slowly. The growth in investments in clean energy in Europe are encouraging but need to be ramped up considerably to contribute to the global goal of tripling by 2030.

## Section 5: public and political engagement

Implementation of mitigation and adaptation policies that address the health dimensions of climate change relies on a political environment in which different actors and institutions across society engage with climate and health. This section tracks seven indicators assessing climate and health engagement in the scientific community, and among individuals on X (formally known as Twitter), governments and politicians at the EU and national levels, the corporate sector, and media outlets.^154^ Three new indicators have been added focusing on anthropogenic climate change in areas where health impact studies have been done and engagement of politicians and media outlets in climate and health.

### Indicator 5.1: scientific engagement with health and climate change

#### Indicator 5.1.1: coverage of health and climate change in scientific articles

By informing relevant multi-sectoral actors in society, scientific evidence helps understand how climate change interacts with health across population groups, shapes mitigation and adaptation strategies, and evaluates policy efficiency.^155^, ^156^ This indicator tracks academic publications on climate change and health in Europe over time, using a machine-learning classifier^157^ applied to the open-access bibliographic database OpenAlex (appendix 4 pp 168–69).

**Figure 8:**
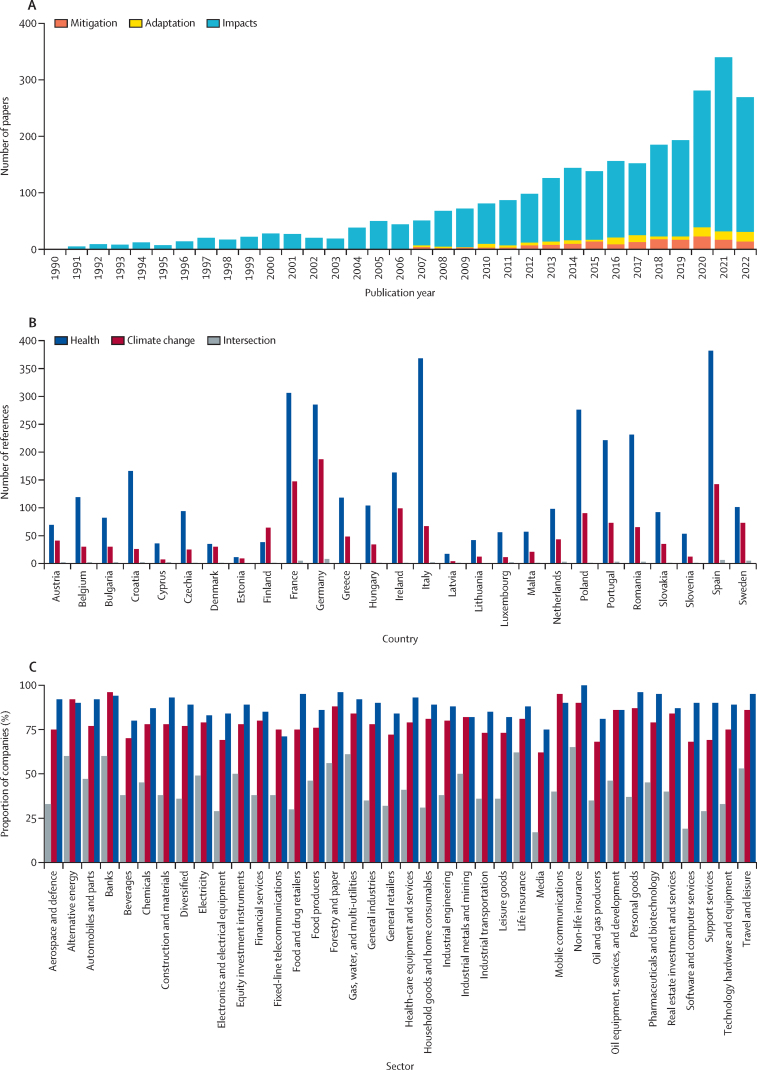
Engagement with climate change and health in science, politics, and the corporate sector in Europe (A) Numbers of scientific publications on the nexus of climate change and health between 1990 and 2022, grouped by publications focusing on mitigation, adaptation, or impact. (B) Total number of references to health, climate change, and their intersection by country in the European Parliament between 2014 and 2022. (C) The proportion of companies by sector that mention health, climate change, and their intersection in the companies' Global Compact Communication on Progress reports in 2022. Sectors with less than ten data points are excluded from the plot.

The scientific literature on health and climate in Europe has rapidly expanded since the early 2000s (figure 8A). While there were (slightly) fewer studies published in 2022 than in 2020 and 2021, the 340 publications identified represent a 32% increase in 2022 since 2019. Southern Europe was the most studied in 2022, while eastern Europe had the largest increase in studies: from 22 studies in 2017 to 76 in 2022 (+245%). Studies focused heavily on health impacts. Of 31 (9%) of 340 studies, 15 (4%) focused on mitigation and 16 (5%) on adaptation, which was a slight decline compared with 39 (11%) of 346 studies in 2020. Only four (2·1%) of 340 studies of the 2022 climate-health literature included a reference to equality, equity, or justice.

#### Indicator 5.1.2: coverage of the health impacts of anthropogenic climate change

This new indicator uses indicator 5.1.1 output, then extracts locations from the documented studies and tests whether the observed trends in climatic variables (ie, temperature and precipitation) were consistent with climate models that simulate the climate system with anthropogenic forcing (ie, trends attributable to climate change). Of the 6276 articles on the different health impacts of climate change in Europe during 1990–2022, 4134 (66%) studies were identified where long-term changes in climatic factors were attributed to anthropogenic climate change. 1261 (31%) publications were found in southern Europe and 1234 (30%) in northern Europe (appendix 4 pp 170–72).

1262 (31%) of 4134 studies were related to infectious diseases, 1061 (26%) on mortality and morbidity, and 737 (18%) cardiorespiratory diseases, with some studies referencing multiple health themes. 222 (5%) were linked food security, 255 (5%) on mental health, 85 (2%) on water security, and 87 (2%) were on direct injury or death associated with climate change. Across subregions, different health outcomes were most studied. Mental health constituted 150 (12·2%) of 1234 publications in northern Europe, but 15 (2·5%) of 610 in eastern Europe. The proportion of studies examining cardio-respiratory disease was consistent (around 16%) across subregions.

#### Indicator 5.2: individual engagement with health and climate change on social media

Public opinion has a considerable influence on government response to climate change.^158^ Recent evidence suggests that a health framing of climate change can bolster public support for mitigation policies and enhance people's intentions to advocate for solutions.^159^ However, little is known about European citizen engagements with health and climate change. Considering several studies show that X (Twitter) data can be used to examine public engagement with climate change (particularly due to its widespread use in Europe),^160^, ^161^, ^162^ this indicator monitored individual climate and health engagement by identifying geographical locations of media posts and applying a multilingual keyword list to estimate the media posts that contain both climate change and health-related keywords (appendix 4 pp 173–186), thereby improving on the methodology used in 2022.^11^

All geolocated English and non-English language media posts from some of the largest European cities in 2022 were extracted (appendix 4 pp 173–86). From 2 490 601 English language media posts and 6 156 957 non-English language multilingual posts, 146 578 (5·9%) English language posts and 478 910 (7·8%) non-English language posts contained a climate change keyword. However, only 10 037 (0·4%) English-language posts and 30 944 (0·5%) non-English language posts engaging with climate change and health were identified. Overall, these findings suggest that while there is substantial online engagement with climate change, there is still low engagement with the climate–health nexus. Furthermore, only 0·05% of all posts engaging in climate change and health referenced issues related to equality, equity, and justice.

### Indicator 5.3: political engagement with health and climate change

#### Indicator 5.3.1: engagement with health and climate change in the European Parliament

The legislative and budgetary powers of the European Parliament and its role in providing guidelines to member states on their environmental and health policies^163^, ^164^ makes it a key actor in shaping EU climate change policies. This indicator tracks political engagement with health and climate change at an EU-27 level, by assessing references to climate-related and health-related terms in legislators' speeches in the European Parliament between 2014 and 2022 (appendix 4 pp 187–218). In total, 264 058 speeches were assessed.

While there were over 800 references of climate change in legislators' speeches in 2022 and over 1400 references to health, there are only ten (0·1%) references to the intersection of health and climate change in the European Parliament in 2022. This is a decrease from 30 references in 2021, following several years of increasing engagement. Similarly to 2021, the highest engagement with the health dimensions of climate change comes from German legislators, followed by Spanish, French, and Swedish legislators (figure 8B). Of the speeches referencing the climate-health intersection, only two included inequality related terminology.

#### Indicator 5.3.2: political engagement with health and climate change on social media

Social media has become a crucial communication tool for governments and politicians with the public, particularly on key policy issues, such as climate change and health.^165^ This indicator tracks government engagement with climate change and health during 2018–22 using the official X (Twitter) handles of 49 national European governments, heads of government, and key ministries and departments (194 handles in total; appendix 4 pp 219–33). Engagement was tracked with a list of English climate-related and health-related keywords, which were translated into the relevant language for non-English media posts.

While there was substantial online engagement by governments with issues related to health (8%) and climate change (1%) individually of a total 703 792 government posts, there was little engagement with the intersection of the two (only 0·05%) during 2018–22. Illustratively, Germany had the highest engagement with health issues, with 14% of 52 831 German government posts referencing a health keyword—while Germany and Spain had the highest online engagement with climate change, with around 3% of government posts referencing climate change. In contrast, only 0·57% Tweets in both Germany and Spain mention climate change and health (68 402 government posts in total), which was the highest national-level engagement across the whole of Europe. Most governments in Europe made no reference to the health dimensions of climate change and health in their media posts, and none include references to inequality (eg, only one reference in 2022).

### Indicator 5.4: corporate sector engagement with health and climate change

Moving away from fossil fuel dependence requires engagement and actions by the corporate sector.^166^ While the UN Global Compact has been criticised for enabling so-called greenwashing (ie, making unsubstantiated claims that deceive shareholders and stakeholders into believing a company's products and services are environmentally friendly), it remains the largest global voluntary initiative promoting corporate social and environmental responsible commitments.^167^ Over 20 000 companies globally have signed up to the Compact, each submitting an annual report on progress (the Global Compact Communication on Progress [GCCOP]) towards a set of ten social and environmental principles.^168^ This indicator applies a keyword search to 25 272 GCCOP English and non-English reports submitted by 6820 companies in EEA countries and the UK between 2011 and 2022 to identify companies reporting on the climate-health nexus.^169^ Furthermore, as there is a growing awareness of the gendered impacts of climate change on human health,^92^, ^170^ an additional search was done for references to gender or sex (appendix 4 pp 234–40).

Engagement with health in the annual reports is high and somewhat consistent across 2011–22 with more than 75% of corporations referencing health. Since 2018, there has been an increase in the proportion of corporations referencing climate change, with 2672 (76%) of 3511 corporations mentioning climate change in 2022 (figure 8C) compared with just over 951 (54%) of 1757 in 2014. At the same time, an increase in engagement with the health dimensions of climate change can be observed, with 2523 (37%) of corporations referencing the climate change–health intersection in 2022 compared with only 1228 (18%) in 2019. Sectors mostly engaging with the climate-health intersection was the non-life Insurance sector with 20 (65%) of 31 companies; ten (62%) of 16 were from the life insurance sector; and 23 (61%) of 38 were from the gas, water, and multiutilities sector. Overall, references to inequality increased substantially during 2011–22, from 6% to 25% of companies that reference the climate–health nexus. Likewise, a steady increase in climate–health–gender engagement was found, particularly during 2015–17. In 2022, 18% referenced gendered impacts, almost double the proportion compared with 2011.

### Indicator 5.5: media engagement with health and climate change

The media plays a fundamental part in influencing public perceptions, government agendas, and facilitating links between policy makers and the public on climate change issues.^171^ This influence is particularly linked to how the media frames policy issues such as climate change.^172^ To track media engagement across Europe, the online communication of 169 media outlets from 28 EEA countries and the UK was examined in this indicator. As Europeans increasingly consume news from social media platforms,^173^ X (Twitter) data were used to estimate the proportion of posts from these media outlets referring to the climate and health nexus in 2022 (appendix 4 pp 241–55).

In total, 3 727 118 multilingual posts of these outlets were extracted with 547 786 posts containing at least one of the selected keywords from the list of climate change in any language. The climate change and health nexus was identified by focusing on climate change posts that mention health-related terms, which is 44 766 (8·2%). Media engagement with health and climate change shows low, stable levels throughout the period across most countries in our sample. The notable exceptions are Hungary and Malta that showed increasing engagement in the second half of the year, and moderately high levels of engagement throughout the year in Türkiye. Media outlets in these countries reported on the climate change and health nexus in 241 (1%) of 22 983 total posts for Hungary, 110 (0·39%) of 28 448 for Malta, and 17 649 (4%) of 406 680 total posts for Türkiye. Additionally, inequality-related terms were mentioned in 87 (0·19%) of 44 766 multilingual posts of the climate change and health nexus from all media outlets.

### Conclusion

Limiting warming to below 1·5°C to avert some of the worst health impacts requires governments across Europe to improve their responses. It is crucial that political and governance structures across Europe engage with the health dimensions of climate change. Yet, the indicators presented in this section provide a mixed picture of engagement across societal actors. Scientific and corporate sector engagement has continued to grow in 2022. In contrast, low levels of media, political, and individual engagement with climate and health remain. Such low engagement might suggest low levels of awareness of the health impacts of climate change and the health co-benefits of mitigation actions. Across all actors analysed in this section, there was limited engagement with equality, equity, or justice. Given the potential for health framing to strengthen public and political support for mitigation and adaptation, fostering awareness of the relationship between health and climate change across societal actors is essential to promote action.

## Conclusion of the 2024 Europe report of the *Lancet* Countdown on health and climate change

This first update of the comprehensive assessment on climate change and health in Europe emphasises that climate change is already negatively affecting the health of European populations, and that in the absence of appropriate climate action, these impacts will continue to increase in the foreseeable future.

Indicators suggest that the negative health impacts of climate change have been increasing compared with baseline levels (section 1), with most impacts exceeding previously reported levels.^11^ Rising temperatures increased heat-related mortality, reduced labour supply, and increased periods of risky hours for physical activity. Exposure to extreme events, such as heatwaves, wildfire, and droughts has increased in most European subregions, resulting in food insecurity and various negative health impacts. The climatic suitability for a wide range of climate-sensitive infectious diseases and their vectors (eg, leishmaniasis, West Nile virus, dengue, malaria, and *Ixodes ricinus* ticks) continues to increase rapidly across Europe. Climate change also resulted in economic losses (eg, reduced GDP per capita growth and damage due to extreme events; section 4).

These health threats and vulnerabilities were experienced across different European subregions and population groups (section 1) with southern Europe more affected by heat-related illnesses, wildfires, drought, food insecurity, and leishmaniasis, and northern Europe more affected by *Vibrio* and ticks. Differential impacts are also seen within countries among different groups; for example, with women at higher risk of heat-related mortality, highly deprived areas being more exposed to wildfire smoke, and older people more susceptible to heat exposure.

Due to challenges in quantitatively incorporating inequalities, inequities, and injustices within our indicators (primarily due to their reliance on publicly available population data and the absence of disaggregated assessments of the climate health burden),^91^ the indicators presented offer only a glimpse of the much larger picture. However, they underscore the crucial importance of incorporating considerations of inequality into climate change strategies and highlight the necessity for more robust research to delve into the unequal impacts of climate change on health.^91^

Since the 2022 report,^11^ there have been some encouraging trends in adaptation (section 2) and mitigation (section 3) in some parts of Europe. Nevertheless, adaptation remains too often neglected while competing with other political issues for financial resources. With the current trajectory estimating that carbon neutrality will be reached as late as 2100, the road to net-zero energy systems remains woefully inadequate. To be on track for net-zero emissions by 2050, global clean energy investment should nearly triple by 2030 and fossil fuel investment should reduce to less than half its current value.^152^ Importantly, European countries continue to drive environmental pressures and negative climate and health impacts elsewhere by their consumption of goods and services produced in other parts of the world. Thus, it is crucial for Europe to accelerate climate action—requiring political will and engagement across societal actors and engagement with the health dimensions of climate change. Yet, there is little media, political, and individual climate and health engagement, with not enough attention paid to the associated inequalities (section 5).

Climate change is not a far-in-the future theoretical scenario: it is here, and it kills.^8^ Climate change impacts are likely to worsen within and beyond Europe, affecting the wellbeing of billions of people. Recognising the impacts of climate change within and beyond Europe and its role in creating the climate crisis, Europe should commit to a fair and healthy environmental transition, which includes taking global responsibility and supporting the most affected communities.

## Declaration of interests

VK and OS are staff members of the WHO Regional Office for Europe. The authors alone are responsible for the views expressed in this publication and they do not necessarily represent the decisions or policies of WHO. The designations employed and the presentation of the material in this publication do not imply the expression of any opinion whatsoever on the part of WHO concerning the legal status of any country, territory, city, or area or of its authorities, or concerning the delimitation of its frontiers or boundaries. Dotted and dashed lines on maps represent approximate border lines for which there might not yet be full agreement. AK and EV are staff members of the European Environment Agency. The views expressed in this article are solely those of the authors and its content does not necessarily represent the views or position of the European Environment Agency. All other authors declare no competing interests.

## Supplementary Materials

French translation of the abstract

German translation of the abstract

Spanish translation of the abstract

## Footnotes

* These indicators are new to the 2024 Europe Report of the *Lancet* Countdown.
