# Correction to *Lancet Public Health* 2024; 9: e495–522

**DOI:** 10.1016/S2468-2667(24)00129-4

**Published:** 2024-06-26

*van Daalen KR, Tonne C, Semenza JC, et al. The 2024 Europe report of the* Lancet *Countdown on health and climate change: unprecedented warming demands unprecedented action.* Lancet Public Health 2024; **9:** e495–522—The author's name should read Josep M Antó, and an affiliation for Nacho Sanchez-Valdivia was removed. In the introduction, the number of academic and UN institutions should read 42. The labels in figure 1B–D and 3C–D have been changed to: Açores. The legend has been adjusted to: “…in the recent 2003–22 period and the pre-industrial period (1850–1900)”. The title for figure 2 has been changed to: Wildfire danger and smoke. The legend now includes date ranges for categories 2A–D. For Indicator 1.3.3, R_0_ is defined as reproduction number and a reference to figure 3B has been added. For the figure 3B y-axis label and legend, relative change in imports of dengue cases has been changed to estimated number of dengue cases. The labels and legend for figure 4 have been changed with the corrected abbreviation for seasonal pollen integral (SPIn). In figure 6, a datapoint has been relabelled to Russian Federation. For Indicator 5.4, the number of corporations referencing the climate change–health intersection for 2022, should read “1284 (37%) of 3511 corporations and for 2019, should read 416 (18%) of 2309 corporations.“ The declaration of interests section now includes details for AK and EV. In the acknowledgments section, the funder name has been amended to EU H2020 EXHAUSTION. An updated appendix has been linked to the Article. These corrections have been made as of June 26, 2024.

