# Supplementary material for: The 2024 Europe report of the Lancet Countdown on health and climate change: unprecedented warming demands unprecedented action
Source: Lancet Public Health. 2024 May 12;9(7):e495–522. doi: 10.1016/S2468-2667(24)00055-0 (PMC11209670; doi:10.1016/S2468-2667(24)00055-0)
Supplement: French translation of the abstract [file mmc1.pdf]

THE LANCET
Public Health

Supplementary appendix 1

This translation in French was submitted by the authors and we reproduce it as supplied. It has not been peer reviewed. *The Lancet*'s editorial processes have only been applied to the original in English, which should serve as reference for this manuscript.

Cette traduction en français a été proposée par les auteurs et nous l'avons reproduite telle quelle. Elle n'a pas été examinée par des pairs. Les processus éditoriaux du *Lancet* n'ont été appliqués qu'à l'original en anglais et c'est cette version qui doit servir de référence pour ce manuscrit.

Supplement to: van Daalen KR, Tonne C, Semenza JC, et al. The 2024 Europe report of the *Lancet* Countdown on health and climate change: unprecedented warming demands unprecedented action. *Lancet Public Health* 2024; published online May 12. https://doi.org/10.1016/S2468-2667(24)00055-0.

LANCET COUNTDOWN: HEALTH AND CLIMATE CHANGE IN EUROPE

Le rapport européen 2024 du Lancet Countdown sur la santé et les changements climatiques: un réchauffement inédit exige une action sans precedent

Kim R van Daalen, Cathryn Tonne, Jan C Semenza, Joacim Rocklöv, Anil Markandya, Niheer Dasandi, Slava Jankin, Hicham Achebak, Joan Ballester, Hannah Bechara, Thessa M Beck, Max W Callaghan, Bruno M Carvalho, Jonathan Chambers, Marta Cirah Pradas, Orin Courtenay, Shouro Dasgupta, Matthew J Eckelman, Zia Farooq, Peter Fransson, Elisa Gallo, Olga Gasparyan, Nube Gonzalez-Reviriego, Ian Hamilton, Risto Hänninen, Charles Hatfield, Kehan He, Aleksandra Kazmierczak, Vladimir Kendrovski, Harry Kennard, Gregor Kiesewetter, Rostislav Kouznetsov, Hedi Katre Kriit, Alba Llabrés-Brustenga, Simon J Lloyd, Martín Lotto Batista, Carla Maia, Jaime Martinez-Urtaza, Zhifu Mi, Carles Milà, Jan C Minx, Mark Nieuwenhuijsen, Julia Palamarchuk, Dafni Kalatzi Pantera, Marcos Quijal-Zamorano, Peter Rafaj, Elizabeth J Z Robinson, Nacho Sánchez-Valdivia, Daniel Scamman, Oliver Schmoll, Maquins Odhiambo Sewe, Jodi D Sherman, Pratik Singh, Elena Sirotkina, Henrik Sjödin, Mikhail Sofiev, Balakrishnan Solaraju-Murali, Marco Springmann, Marina Treskova, Joaquin Triñanes, Eline Vanuytrecht, Fabian Wagner, Maria Walawender, Laura Warnecke, Ran Zhang, Marina Romanello, Josep M Antò, Maria Nilsson, Rachel Lowe

Synthèse

Des températures record ont été enregistrées dans le monde entier en 2023. Sans action climatique, les effets négatifs du climat sur la santé vont s'aggraver partout sur la planète, touchant des milliards de personnes. Les températures en Europe se réchauffent deux fois plus vite que la moyenne mondiale, menaçant la santé des populations de tout le continent et entraînant des pertes humaines inutiles. Le rapport du Lancet Countdown en Europe a été créé en 2021 pour évaluer le profil santé des changements climatiques. Son objectif est de stimuler la volonté sociale et politique européenne de mettre en œuvre rapidement des mesures d'atténuation et d'adaptation aux changements climatiques répondant aux besoins de la santé. En 2022, cette collaboration a publié son premier rapport d'indicateurs, qui suit les progrès réalisés en matière de santé et des changements climatiques au moyen de 33 indicateurs, et dans cinq domaines.

Ce nouveau rapport suit 42 indicateurs, qui mettent en évidence les impacts négatifs des changements climatiques sur la santé humaine, le retard pris par les pays européens dans l'action climatique et les occasions manquées de protéger ou d'améliorer la santé grâce à une action climatique adaptée. Les méthodes utilisées pour définir les indicateurs présentés dans le rapport de 2022 ont été améliorées et neuf nouveaux indicateurs ont été rajoutés, couvrant la leishmaniose, les tiques, la sécurité alimentaire, les émissions liées au secteur de la santé, les émissions liées à la production et à la consommation, les investissements dans les énergies propres et les initiatives scientifique, politique et médiatique en faveur du climat et de la santé. Les impacts négatifs sur la santé liés au climat et la responsabilité des changements climatiques sont inégaux au niveau régional et mondial, ainsi le présent rapport s'efforce également de réfléchir aux questions d'inégalité et de justice en mettant en lumière les groupes à risque en Europe et la responsabilité de l'Europe dans la crise climatique.

Les changements climatiques ne sont pas un scénario lointain

Notre rapport met en lumière les conséquences multidimensionnelles déjà en cours des changements climatiques sur la santé et sur les déterminants de la santé en Europe. Si le fait de veiller à ce que la température n'augmente pas de plus de 1,5 °C permettra d'éviter certains des pires impacts climatiques sur la santé, la planète est déjà proche de ce chiffre et ne parvient pas à réduire adéquatement ses émissions.

On estime que les décès liés à la chaleur ont augmenté dans la plupart des pays d'Europe, avec une augmentation moyenne de 17,2 décès pour 100 000 habitants entre les périodes 2003–2012 et 2013–2022 (indicateur 1.1.4). Les heures à risque pour l'activité physique (en raison du risque de stress thermique) se sont étendues au-delà des moments les plus chauds de la journée au cours de la période 1990–2022, pour les activités intermédiaires (par exemple le

cyclisme ou le football) comme pour les activités difficiles (par exemple le rugby ou le VTT) (indicateur 1.1.3), ce qui pourrait amener la population à réduire son activité physique globale et, par conséquent, à augmenter son risque de maladies non transmissibles. L'exposition à la chaleur peut nuire davantage à la santé des personnes en influant sur les déterminants sociaux et économiques de la santé. Par exemple, l'offre de travail a été considérablement plus faible en 2016-2020 par rapport au niveau de référence de 1965-1994 (indicateur 4.1.2). L'adéquation climatique de divers pathogènes et vecteurs de maladies sensibles a augmenté en Europe (par exemple : Vibrio, virus du Nil occidental, dengue, chikungunya, Zika, paludisme, leishmaniose et tiques ; indicateur 1.3). Au cours de la période 2011–2020, un nombre nettement plus élevé de régions ont été jugées propices à l'apparition de la leishmaniose (68 %) par rapport à 2001–2010 (55 %), avec une expansion vers le nord des zones propices au-delà de la zone endémique historique (indicateur 1.3.5). L'augmentation relative du risque d'épidémie était de 256 % pour le virus du Nil occidental de 1951–1960 (0,05 de risque d'épidémie) à 2013–2022 (0,01 de risque d'épidémie ; indicateur 1.3.2), et de 40,9 % pour la dengue de 1951–1960 (R0 0·09 estimé) à 2013–2022 (R0 0·14 estimé ; indicateur 1.3.3). De plus, le nombre de mois propices aux tiques Ixodes ricinus (vecteur de la maladie de Lyme et de l'encéphalite transmise par les tiques) a augmenté de 0,68 mois en Asie occidentale et de 0,58 mois en Europe orientale. Les changements climatiques entraînent également des changements dans l'intensité et la fréquence des phénomènes météorologiques extrêmes. Des tendances croissantes en matière de risque de feu de forêt ont été observées en Europe entre 1980 et 2022 (indicateur 1.2.1), bien qu'aucune tendance n'ait été détectée pour l'exposition aux émissions de matières particulaires issues de feux de forêt (de diamètre ≤ 2·5·µm ; PM25) entre 2003 et 2022 (indicateur 1.2.1), ce qui pourrait refléter une préparation et une gestion efficaces des incendies de forêt. L'Europe de l'Ouest, du Sud et de l'Est ont connu des augmentations substantielles des conditions de sécheresse extrême de 2000-2009 à 2010-2019 (indicateur 1.2.2). En outre, en 2021, les changements climatiques ont entraîné une augmentation de près de 12 millions de personnes touchées par une insécurité alimentaire modérée ou grave en Europe (indicateur 1.5.1).

Des inégalités de santé qui s'aggravent dans un monde qui se réchauffe

Ces impacts interconnectés sur la santé tendent à être répartis de manière inégale entre les populations en raison des différences d'exposition, de sensibilité et de capacité d'adaptation, reflétant souvent des schémas croisés de développement socioéconomique, de marginalisation et d'inégalités historiques et persistantes. Les populations les plus touchées tendent à être les moins responsables et les moins susceptibles de voir leurs besoins reconnus ou traités en priorité. L'Europe du Sud a tendance à être plus touchée par les maladies liées à la chaleur, les incendies de forêt, l'insécurité alimentaire, la sécheresse et la leishmaniose, alors que l'Europe du Nord est touchée de manière égale ou plus élevée par les vibrions et les tiques (section 1). Au sein des pays, les minorités ethniques et les autochtones, les groupes à faible revenu, les personnes migrantes et déplacées, les minorités sexuelles et de genre et les femmes qui passent par la grossesse et l'accouchement ont tendance à être plus durement touchées par les impacts sanitaires liés au climat.

Ce rapport montre que la mortalité liée à la chaleur est deux fois plus élevée chez les femmes que chez les hommes (indicateur 1.1.4), que les ménages à faible revenu ont une probabilité nettement plus élevée de souffrir d'insécurité alimentaire (indicateur 1.5.1), que les décès attribuables à un régime alimentaire déséquilibré sont plus élevés chez les femmes (indicateur 3.4.2) et que l'exposition à des particules PM2.5 liées aux feux de forêt- est plus élevée dans les zones très défavorisées. Des stratégies d'adaptation mal conçues, telles que les solutions fondées sur la nature (indicateur 2.2.2) ou les mécanismes d'amélioration du confort thermique (indicateur 2.2.3) qui ne tiennent pas suffisamment compte de l'équité, peuvent perpétuer les inégalités en matière d'environnement et de santé. Tous les indicateurs ne peuvent intégrer des analyses sur plusieurs groupes de population, ainsi notre rapport n'offre que l'aperçu d'une situation très générale. Il souligne l'importance d'une recherche plus approfondie pour mieux

comprendre les impacts inégaux des changements climatiques sur la santé et mieux choisir les mesures de protection de la santé pour toutes les populations.

Bien que les changements climatiques exacerbent les inégalités existantes, les indicateurs sur la gouvernance et la politique montrent peu d'implication envers l'égalité, l'équité ou la justice dans la recherche, les politiques et les médias traitant de climat et de santé (section 5). En outre, l'équité environnementale, y compris la lutte contre les distributions socio spatiales disproportionnées de l'exposition aux changements climatiques et de ses risques pour la santé, n'est pas un objectif explicite dans les politiques européennes existantes.

Se responsabiliser et accélérer l'action

De nombreux pays européens restent des contributeurs historiques et actuels majeurs aux émissions de gaz à effet de serre. Alors que les pays européens ont bénéficié de la croissance économique que ces émissions ont permise, ce sont d'autres pays (ceux qui ont le moins émis) qui sont les plus touchés par les changements climatiques actuels et futurs. Le changement climatique est un problème de justice sociale et environnementale. En 2021, les émissions provenant de la combustion de combustibles fossiles étaient de 5,4 tonnes de CO2 par personne en Europe, soit six fois plus qu'en Afrique et près de trois fois plus qu'en Amérique centrale et en Amérique du Sud (indicateur 3.1.1). Les pays européens se dirigent vers des émissions nettes nulles à un rythme qui demeure beaucoup trop lent, la trajectoire actuelle de l'Europe suggérant la réalisation de la neutralité carbone seulement d'ici 2100 (indicateur 3.1.1). Il est notable que du fait de la consommation européenne de biens et de services produits dans d'autres parties du monde, les pays européens continuent d'exercer des pressions sur l'environnement (par exemple des émissions de gaz à effet de serre et de la pollution atmosphérique locale) et de causer des effets néfastes sur le climat et la santé ailleurs dans le monde (indicateur 3.2.1). Bien que plusieurs pays européens aient pris des mesures pour réduire les émissions des soins de santé, on estime que le secteur de la santé a contribué à hauteur de 330 mégatonnes (Mt) d'équivalent CO2 (éq) en 2020 (indicateur 3.5). En outre, la consommation de charbon a augmenté pour atteindre 13 % de l'approvisionnement énergétique total de l'Europe en 2021 (indicateur 3.1.2), et 29 des 53 pays continuent de fournir des subventions nettes pour les combustibles fossiles (indicateur 4.2.1).
L'absence de mesures ambitieuses risque d'encore aggraver les effets des changements climatiques déjà présents et de laisser passer des occasions d'apporter des avantages considérables à court terme pour la santé. De tels avantages sont par exemple la réduction de la mortalité prématurée due à une réduction des particules fines ambiantes (indicateur 3.2.1) ; l'augmentation de l'activité physique grâce à un transport plus actif ; la réduction de la morbidité et de la mortalité grâce à des régimes alimentaires moins polluants, moins transformés, économes en ressources et sains à base de plantes (indicateur 3.4).

Limiter le réchauffement à moins de 1,5 °C afin d'éviter d'autres effets néfastes sur la santé exige des gouvernements de toute l'Europe qu'ils renforcent leur réponse. Par conséquent, les structures politiques et de gouvernance de toute l'Europe devraient s'intéresser aux dimensions sanitaires des changements climatiques. Toutefois, alors que l'implication scientifique (indicateur 5.1) et l'implication du secteur des entreprises (indicateur 5.4) ont continué de croître en 2022, les niveaux d'implication médiatique (indicateur 5.5), politique (indicateur 5.3) et individuel (indicateur 5.2) dans le lien climat-santé ont été faibles. Mettre en valeur l'aspect santé pourrait renforcer le soutien public et politique en faveur de l'action pour le climat et la nécessité pour les sociétés européennes de s'adapter aux effets des changements climatiques sur la santé, il est donc essentiel de sensibiliser les acteurs politiques et les institutions au lien climat-santé pour encourager davantage à agir.

Une transition environnementale juste et saine

Pour satisfaire aux recommandations du dernier rapport du Groupe d'experts intergouvernemental sur l'évolution du climat (GIEC) sur le zéro net d'ici 2040, les émissions des systèmes énergétiques européens devraient chuter à environ trois fois leur taux actuel. Cette

diminution devra être encore plus rapide si les émissions équitables, qui tiennent compte des émissions historiques et de la population de l'Europe, sont utilisées pour allouer les réductions à l'échelle mondiale. Lorsque la justice est prise en compte, l'action climatique ne garantit pas seulement une transition environnementale équitable et saine, mais réduit également les inéquités entre les pays et à l'intérieur de ceux-ci en ce qui concerne les principales voies d'impact sur la santé, notamment la pollution de l'air, l'activité physique liée au transport actif et les régimes alimentaires sains. Suite à la reconnaissance des impacts des changements climatiques à l'intérieur et à l'extérieur de l'Europe et du rôle de l'Europe dans la création de la crise climatique, l'Europe devrait s'engager en faveur d'une transition environnementale juste et saine, ce qui implique de prendre des responsabilités au niveau mondial et de soutenir les communautés les plus touchées.
