# Supplementary material for: The 2024 Europe report of the Lancet Countdown on health and climate change: unprecedented warming demands unprecedented action
Source: Lancet Public Health. 2024 May 12;9(7):e495–522. doi: 10.1016/S2468-2667(24)00055-0 (PMC11209670; doi:10.1016/S2468-2667(24)00055-0)
Supplement: German translation of the abstract [file mmc2.pdf]

THE LANCET
Public Health

Supplementary appendix 2

This translation in German was submitted by the authors and we reproduce it as supplied. It has not been peer reviewed. *The Lancet*'s editorial processes have only been applied to the original in English, which should serve as reference for this manuscript.

Diese Übersetzung in deutscher Sprache wurde von den Autoren eingereicht und wir reproduzieren sie wie vorgelegt. Die Übersetzung wurde nicht von Experten begutachtet. Die redaktionellen Prozesse von The Lancet wurden nur auf das Original in englischer Sprache angewendet, das als Referenz für dieses Manuskript dienen soll.

Supplement to: van Daalen KR, Tonne C, Semenza JC, et al. The 2024 Europe report of the *Lancet* Countdown on health and climate change: unprecedented warming demands unprecedented action. *Lancet Public Health* 2024; published online May 12. https://doi.org/10.1016/S2468-2667(24)00055-0.

Der europäische Lancet Countdown Bericht zu Klimawandel und Gesundheit 2024: Europa heizt sich stärker auf als je zuvor – ambitionierte Maßnahmen dringend erforderlich

Kim R van Daalen, Cathryn Tonne, Jan C Semenza, Joacim Rocklöv, Anil Markandya, Niheer Dasandi, Slava Jankin, Hicham Achebak, Joan Ballester, Hannah Bechara, Thessa M Beck, Max W Callaghan, Bruno M Carvalho, Jonathan Chambers, Marta Cirah Pradas, Orin Courtenay, Shouro Dasgupta, Matthew J Eckelman, Zia Farooq, Peter Fransson, Elisa Gallo, Olga Gasparyan, Nube Gonzalez-Reviriego, Ian Hamilton, Risto Hänninen, Charles Hatfield, Kehan He, Aleksandra Kazmierczak, Vladimir Kendrovski, Harry Kennard, Gregor Kiesewetter, Rostislav Kouznetsov, Hedi Katre Kriit, Alba Llabrés-Brustenga, Simon J Lloyd, Martín Lotto Batista, Carla Maia, Jaime Martinez-Urtaza, Zhifu Mi, Carles Milà, Jan C Minx, Mark Nieuwenhuijsen, Julia Palamarchuk, Dafni Kalatzi Pantera, Marcos Quijal-Zamorano, Peter Rafaj, Elizabeth J Z Robinson, Nacho Sánchez-Valdivia, Daniel Scamman, Oliver Schmoll, Maquins Odhiambo Sewe, Jodi D Sherman, Pratik Singh, Elena Sirotkina, Henrik Sjödin, Mikhail Sofiev, Balakrishnan Solaraju-Murali, Marco Springmann, Marina Treskova, Joaquin Triñanes, Eline Vanuytrecht, Fabian Wagner, Maria Walawender, Laura Warnecke, Ran Zhang, Marina Romanello, Josep M Antò, Maria Nilsson, Rachel Lowe

Zusammenfassung

Im Jahr 2023 wurden weltweit Rekordtemperaturen gemessen. Ohne ambitionierte Klimaschutzmaßnahmen werden sich die globalen negativen Auswirkungen des Klimawandels auf die Gesundheit weiter verschärfen und Milliarden von Menschen treffen. Die Temperaturen in Europa steigen doppelt so schnell wie im globalen Durchschnitt – das bedroht die Gesundheit der Bevölkerung auf dem europäischen Kontinent und führt zu vermeidbaren Todesfällen. Der Lancet Countdown Europe wurde 2021 ins Leben gerufen. Die interdisziplinäre, internationale Forschungskooperation untersucht die Auswirkungen des Klimawandel auf unsere Gesundheit sowie und soll den gesellschaftlichen und politischen Willen in Europa fördern, gesundheitsbezogene Maßnahmen für Klimaschutz und Klimaanpassung rasch umzusetzen. Im Jahr 2022 veröffentlichte die Kooperation ihren ersten Indikatorenbericht zu den Fortschritten mit Blick auf Gesundheit und Klimawandel anhand von 33 Indikatoren in fünf Bereichen.

Der neue Bericht analysiert dies anhand von 42 Indikatoren: Diese beschreiben die negativen Auswirkungen des Klimawandels auf die menschliche Gesundheit, die verspäteten Klimamaßnahmen europäischer Länder und die verpassten Gelegenheiten zum Schutz oder zur Verbesserung der Gesundheit durch gesundheitsfördernde Klimamaßnahmen. Die den Indikatoren des Berichts aus dem Jahr 2022 zugrunde liegenden Methoden wurden verbessert. So wurden neun weitere Indikatoren ergänzt: Leishmaniose, Zecken, Ernährungssicherheit, Emissionen im Gesundheitssektor, produktions- und verbrauchsbedingte Emissionen, Investitionen in saubere Energie sowie die Auseinandersetzung von Wissenschaft, Politik und Medien mit den Themen Klima und Gesundheit . Da die negativen Gesundheitsfolgen des Klimawandels und die Verantwortung für den Klimawandel regional und global ungleich verteilt sind, versucht dieser Bericht auch, Aspekte der Ungleichheit und Gerechtigkeit zu beleuchten, indem er die Risikogruppen in Europa und die Verantwortung Europas für die Klimakrise hervorhebt.

Der Klimawandel ist kein fernes Zukunftsszenario

Unser Bericht zeigt die mehrdimensionalen Auswirkungen des Klimawandels auf die Gesundheit und Gesundheitsdeterminanten in Europa auf, die bereits heute zu beobachten sind. Eine Begrenzung des Temperaturanstiegs auf 1,5 °C kann einige der schlimmsten gesundheitlichen Auswirkungen des Klimawandels abwenden. Die Welt nähert sich aber bereits diesem Niveau und schafft es nicht, die Emissionen ausreichend zu senken.

Schätzungen zufolge hat die Zahl hitzebedingter Todesfälle in den meisten europäischen Ländern

zugenommen. Im Zeitraum von 2013 - 2022 stiegen die Todesfälle im Vergleich zum Zeitraum von 2003 – 2012 dabei im Schnitt um 17,2 Todesfälle pro 100.000 Einwohner (Indikator 1.1.4). Die Risikozeiten für körperliche Aktivitäten (aufgrund des Risikos von Hitzestress) haben sich in den Jahren 1990–2022 sowohl für moderate (z. B. Radfahren oder Fußball) als auch für anstrengende (z. B. Rugby oder Mountainbiking) Aktivitäten über die heißesten Tagesstunden hinaus ausgedehnt (Indikator 1.1.3). Folglich führt dies dazu, dass Menschen ihre körperlichen Aktivitäten insgesamt reduzieren und damit ihr Risiko für nicht übertragbare Krankheiten erhöhen. Hitzebelastung kann die Gesundheit der Menschen zusätzlich beeinträchtigen, da sie sich auf die sozialen und wirtschaftlichen Determinanten der Gesundheit auswirkt. Beispielsweise war das Arbeitskräfteangebot im Zeitraum 2016–2020 im Vergleich zur zum Zeitraum 1965–1994 deutlich geringer (Indikator 4.1.2). Die Klimatauglichkeit verschiedener klimaempfindlicher Krankheitserreger und -überträger hat in Europa zugenommen (z. B. Vibrio, West-Nil-Virus, Dengue, Chikungunya, Zika, Malaria, Leishmaniose und Zecken; Indikator 1.3). Für den Zeitraum 2011–2020 wurden deutlich mehr Regionen als geeignet für die Übertragung von Leishmaniose eingestuft (68 % – gegenüber 2001–2010 mit 55 %), wobei sich diese Gebiete über die bisherige endemische Zone hinaus nach Norden ausweiten (Indikator 1.3.5). Der relative Anstieg des Ausbruchsrisikos betrug 256 % für das West-Nil-Virus von 1951–1960 (Ausbruchsrisiko 0,05) bis 2013–2022 (Ausbruchsrisiko 0,01; Indikator 1.3.2) und 40,9 % für Dengue von 1951–1960 (geschätztes R0 0,09) bis 2013–2022 (geschätztes R0 0,14; Indikator 1.3.3). Darüber hinaus stieg die Zahl der geeigneten Monate für Zecken des Typs Ixodes ricinus (Überträger der Lyme-Borreliose und der durch Zecken übertragenen Enzephalitis) in Westasien um 0,68 Monate und in Osteuropa um 0,58 Monate. Der Klimawandel führt auch zu veränderten Intensitäten und Häufigkeiten von extremen Klimaereignissen. Im Zeitraum 1980–2022 wurden in Europa steigende Trends für Waldbrandgefahr beobachtet (Indikator 1.2.1), wenngleich für die Belastung durch Feinstaub aufgrund von Waldbränden (mit einem Durchmesser von ≤2–5 µm; PM2,5) zwischen 2003 und 2022 dieser Trend nicht beobachtbar ist (Indikator 1.2.1). Dies könnte auf eine wirksame Vorbereitung auf Waldbrände und deren Bekämpfung zurückzuführen sein. In West-, Süd- und Osteuropa haben extreme Dürreperioden zwischen 2000–2009 und 2010–2019 erheblich zugenommen (Indikator 1.2.2). Darüber hinaus waren 2021 fast 12 Millionen mehr Menschen in Europa aufgrund des Klimawandels von mäßiger oder schwerer Ernährungsunsicherheit betroffen (Indikator 1.5.1).

Verschärfung gesundheitlicher Ungerechtigkeiten in einer zunehmend wärmeren Welt

Die miteinander verknüpften Gesundheitsauswirkungen sind in der Regel aufgrund von Unterschieden hinsichtlich Exposition, Empfindlichkeit und Anpassungsfähigkeit ungleich über die Bevölkerung verteilt. Dies spiegelt häufig die sich überschneidenden Muster der sozioökonomischen Entwicklung, der Marginalisierung und der historischen – und anhaltenden – Muster der Ungerechtigkeit wider. Am stärksten sind tendenziell die Bevölkerungsgruppen betroffen, die am wenigsten Verantwortung tragen und die mit geringerer Wahrscheinlichkeit anerkannt oder vorrangig behandelt werden. In Südeuropa treten generell mehr hitzebedingte Krankheiten, Waldbrände, Ernährungsunsicherheit, Dürre und Leishmaniose auf, während Nordeuropa gleichermaßen oder stärker durch Vibrio und Zecken gefährdet ist (Abschnitt 1). Innerhalb der Länder sind ethnische Minderheiten und indigene Völker, einkommensschwache Gemeinschaften, Migrant:innen und Vertriebene, Minderheiten in Bezug auf Sexualität und soziales Geschlecht (gender) sowie Frauen, die schwanger sind oder ein Kind zur Welt bringen, meist stärker von klimabedingten Gesundheitsauswirkungen betroffen.

Der Bericht zeigt, dass die hitzebedingte Sterblichkeit bei Frauen doppelt so hoch war wie bei Männern (Indikator 1.1.4), dass in einkommensschwachen Haushalten die Wahrscheinlichkeit, dass Menschen unter Ernährungsunsicherheit litten, wesentlich höher war (Indikator 1.5.1), dass die Zahl der Todesfälle, die auf eine unausgewogene Ernährung zurückzuführen waren, bei Frauen

höher war (Indikator 3.4.2) und dass die Exposition gegenüber Feinstaub durch Waldbrände (PM2,5) in stark benachteiligten Gebieten höher war. Schlecht konzipierte Anpassungsstrategien wie naturbasierte Lösungen (Indikator 2.2.2) oder Mechanismen zur Verbesserung und Aufrechthaltung des thermischen Komforts (Indikator 2.2.3), die Aspekte der Gerechtigkeit nicht angemessen berücksichtigen, können ökologische und gesundheitliche Ungerechtigkeiten fortbestehen lassen. Da nicht alle Indikatoren weiterführende Analysen zu verschiedenen Bevölkerungsgruppen enthalten, bietet unser Bericht nur einen Einblick in das Gesamtbild und unterstreicht, wie wichtig fundierte Forschung ist, um die ungleichen Auswirkungen des Klimawandels auf die Gesundheit eingehender zu untersuchen und Maßnahmen zum Gesundheitsschutz für alle Bevölkerungsgruppen zu konzipieren.

Obwohl der Klimawandel bestehende Ungerechtigkeiten verschärft, zeigen die Indikatoren zu Governance und politischer Steuerung, dass Aspekte der Gleichheit, Gleichberechtigung oder Gerechtigkeit in Klima- und Gesundheitsforschung, Politik und Medien unzureichend berücksichtigt werden (Abschnitt 5). Darüber hinaus ist Umweltgerechtigkeit, einschließlich der Bewältigung unverhältnismäßiger sozialräumlicher Verteilungen von Klimaexposition und Gesundheitsrisiken, kein ausdrückliches Ziel der bestehenden EU-Politik.

Verantwortung übernehmen und Maßnahmen beschleunigen

Viele europäische Länder sind nach wie vor (sowohl in der Vergangenheit als auch heute) erhebliche Verursacher von Treibhausgasemissionen. Während europäische Länder von dem durch diese Emissionen ermöglichten Wirtschaftswachstum profitiert haben, sind andere Länder – die am wenigsten emittiert haben – am stärksten von den derzeitigen und künftigen Auswirkungen des Klimawandels betroffen. Der Klimawandel ist eine Frage der sozialen und ökologischen Gerechtigkeit. Im Jahr 2021 lagen die Emissionen aus der Verbrennung fossiler Brennstoffe in Europa bei 5,4 Tonnen CO2 pro Person – und damit sechsmal so hoch wie in Afrika und fast dreimal so hoch wie in Mittel- und Südamerika (Indikator 3.1.1). Die Geschwindigkeit, mit der sich die Länder Europas in Richtung Netto-Null-Emissionen bewegen, reicht nach wie vor bei Weitem nicht aus. Mit seinem derzeitigen Kurs erreicht Europa Kohlenstoffneutralität erst im Jahr 2100 (Indikator 3.1.1). Durch den europäischen Konsum von Waren und Dienstleistungen, die in anderen Teilen der Welt produziert werden, tragen die europäischen Länder weiterhin zu Umweltbelastungen (z. B. Treibhausgasemissionen und lokale Luftverschmutzung) und den damit verbundenen negativen Auswirkungen auf Klima und Gesundheit in anderen Teilen der Welt bei (Indikator 3.2.1). Obwohl mehrere Länder in Europa Maßnahmen zur Verringerung der Emissionen im Gesundheitswesen ergriffen haben, hat der Gesundheitssektor im Jahr 2020 schätzungsweise 330 Megatonnen (Mt) CO2-Äquivalent (eq) an Emissionen verursacht (Indikator 3.5). Darüber hinaus stieg der Anteil von Kohle an der gesamten Energieversorgung Europas bis 2021 auf 13 % (Indikator 3.1.2), und 29 von 53 Ländern gewähren weiterhin Nettosubventionen für fossile Brennstoffe (Indikator 4.2.1).

Wenn keine entschlossenen Maßnahmen eingeleitet werden, besteht die Gefahr, dass sich die bereits eingetretenen Auswirkungen des Klimawandels noch weiter verschärfen. Es werden Chancen verpasst, kurzfristig beträchtliche gesundheitliche Mehrgewinne (Co-Benefits) zu erzielen, z. B. eine geringere vorzeitige Sterblichkeit durch eine Reduzierung der Feinstaubbelastung in der Luft (Indikator 3.2.1), mehr körperliche Aktivität durch ein mehr aktive Mobilität und eine geringere Erkrankungs- und Sterblichkeitsrate durch die Umstellung auf eine gesunde, pflanzenbasierte Ernährung, die weniger verarbeitete Produkte enthält und zudem ressourceneffizient und umweltschonender ist. (Indikator 3.4).

Um die Erderwärmung auf weniger als 1,5 °C zu begrenzen und weitere gesundheitsschädliche Folgen abzuwenden, müssen die Regierungen in Europa ihre Maßnahmen verstärken. Die europäischen politischen Strukturen sollten daher die gesundheitlichen Aspekte des Klimawandels einbeziehen. Während jedoch das Engagement der Wissenschaft (Indikator 5.1)

und des Unternehmenssektors (Indikator 5.4) im Jahr 2022 weiter zugenommen hat, war das Interesse der Medien (Indikator 5.5), der Politik (Indikator 5.3) sowie von Einzelpersonen (Indikator 5.2) an den Zusammenhängen zwischen Klimawandel und Gesundheit gering. Anbetracht der Tatsache, dass Gesundheitsargumente das Potenzial haben, die öffentliche und politische Unterstützung für Klimaschutzmaßnahmen zu stärken, und die Gesellschaften in Europa sich an die gesundheitlichen Auswirkungen des Klimawandels anpassen müssen, ist die Förderung des Bewusstseins für die Zusammenhänge von Klimawandel und Gesundheit bei allen politischen Akteuren und Institutionen von entscheidender Bedeutung, um weitere Handlungsimpulse zu geben.

Ein fairer und gesunder ökologischer Wandel

Um die Empfehlungen des neuesten Berichts des Zwischenstaatlichen Ausschusses für Klimaänderungen (IPCC, „Weltklimarat“) zu erfüllen und bis 2040 Netto-Null-Emissionen zu erreichen, müssten die Emissionen der europäischen Energiesysteme etwa dreimal so schnell sinken wie heute. Dies muss sogar noch schneller passieren, wenn die Reduktion der Emissionen vor dem Hintergrund der historischen Entwicklung derselben sowie der Bevölkerung Europas global gerecht verteilt werden soll. Wenn Gerechtigkeit berücksichtigt wird, gewährleisten Klimamaßnahmen nicht nur einen fairen und gesunden ökologischen Wandel, sondern vermindern auch die Ungerechtigkeiten zwischen und innerhalb der Länder in Bezug auf zentrale gesundheitliche Auswirkungen wie Luftverschmutzung, körperliche Aktivität durch aktive Mobilität und gesunde Ernährung. In Kenntnis der Auswirkungen des Klimawandels innerhalb und außerhalb Europas und der Rolle Europas bei der Entstehung der Klimakrise sollte sich Europa zu einem fairen und gesunden ökologischen Wandel verpflichten, der die Übernahme globaler Verantwortung und die Unterstützung der am stärksten betroffenen Gemeinschaften einschließt.
