# Supplementary material for: The 2024 Europe report of the Lancet Countdown on health and climate change: unprecedented warming demands unprecedented action
Source: Lancet Public Health. 2024 May 12;9(7):e495–522. doi: 10.1016/S2468-2667(24)00055-0 (PMC11209670; doi:10.1016/S2468-2667(24)00055-0)
Supplement: Spanish translation of the abstract [file mmc3.pdf]

THE LANCET
Public Health

Supplementary appendix 3

This translation in Spanish was submitted by the authors and we reproduce it as supplied. It has not been peer reviewed. *The Lancet*'s editorial processes have only been applied to the original in English, which should serve as reference for this manuscript.

Los autores nos proporcionaron esta traducción al español y la reproducimos tal como nos fue entregada. No la hemos revisado. Los procesos editoriales de *The Lancet* se han aplicado únicamente al original en inglés, que debe servir de referencia para este manuscrito.

Supplement to: van Daalen KR, Tonne C, Semenza JC, et al. The 2024 Europe report of the *Lancet* Countdown on health and climate change: unprecedented warming demands unprecedented action. *Lancet Public Health* 2024; published online May 12. https://doi.org/10.1016/S2468-2667(24)00055-0.

Informe del 2024 del “*Lancet* Countdown” sobre salud y cambio climático en Europa: el calentamiento sin precedentes exige medidas sin precedentes

Kim R van Daalen, Cathryn Tonne, Jan C Semenza, Joacim Rocklöv, Anil Markandya, Niheer Dasandi, Slava Jankin, Hicham Achebak, Joan Ballester, Hannah Bechara, Thessa M Beck, Max W Callaghan, Bruno M Carvalho, Jonathan Chambers, Marta Cirah Pradas, Orin Courtenay, Shouro Dasgupta, Matthew J Eckelman, Zia Farooq, Peter Fransson, Elisa Gallo, Olga Gasparyan, Nube Gonzalez-Reviriego, Ian Hamilton, Risto Hänninen, Charles Hatfield, Kehan He, Aleksandra Kazmierczak, Vladimir Kendrovski, Harry Kennard, Gregor Kiesewetter, Rostislav Kouznetsov, Hedi Katre Kriit, Alba Llabrés-Brustenga, Simon J Lloyd, Martín Lotto Batista, Carla Maia, Jaime Martinez-Urtaza, Zhifu Mi, Carles Milà, Jan C Minx, Mark Nieuwenhuijsen, Julia Palamarchuk, Dafni Kalatzi Pantera, Marcos Quijal-Zamorano, Peter Rafaj, Elizabeth J Z Robinson, Nacho Sánchez-Valdivia, Daniel Scamman, Oliver Schmoll, Maquins Odhiambo Sewe, Jodi D Sherman, Pratik Singh, Elena Sirotkina, Henrik Sjödin, Mikhail Sofiev, Balakrishnan Solaraju-Murali, Marco Springmann, Marina Treskova, Joaquin Triñanes, Eline Vanuytrecht, Fabian Wagner, Maria Walawender, Laura Warnecke, Ran Zhang, Marina Romanello, Josep M Antò, Maria Nilsson, Rachel Lowe

Resumen ejecutivo

En 2023 se registraron temperaturas récord en todo el planeta. Sin medidas drásticas, se prevé que los efectos adversos para la salud relacionados con el clima empeoren en todo el mundo y afecten a miles de millones de personas. Las temperaturas en Europa están subiendo el doble de la media mundial, amenazando la salud de las poblaciones de todo el continente y provocando pérdidas innecesarias de vidas. El “*Lancet* Countdown” en Europa se creó en 2021 para evaluar el perfil de salud del cambio climático, con el objetivo de estimular la voluntad social y política europea de implementar rápidamente medidas de mitigación y adaptación al cambio climático que tengan en cuenta la salud. En 2022, la colaboración publicó su primer informe, en el cuál se monitorizaba los avances en salud y clima a través de 33 indicadores en cinco ámbitos.

Este nuevo informe monitoriza 42 indicadores, destacando los efectos negativos del cambio climático en la salud humana, el retraso de la acción climática de los países europeos y las oportunidades perdidas para desarrollar medidas que protejan y mejoren la salud. Se han mejorado los métodos que sustentan los indicadores presentados en el informe de 2022 y se han añadido 9 nuevos indicadores, que abarcan la leishmaniasis, enfermedades transmitidas por las garrapatas, la seguridad alimentaria, las emisiones de la atención sanitaria, las emisiones basadas en la producción y el consumo, la inversión en energías limpias y el compromiso científico, político y mediático con el clima y la salud. Teniendo en cuenta que las repercusiones negativas para la salud relacionadas con el clima y la responsabilidad por el cambio climático no son iguales a escala regional y mundial, este informe también pretende reflexionar sobre diversos aspectos relacionados con la desigualdad y la justicia, destacando los grupos de riesgo dentro de Europa, así como la responsabilidad de Europa en la crisis climática.

El cambio climático no es un escenario hipotético de un futuro lejano

Nuestro informe destaca los efectos multidimensionales del cambio climático sobre la salud y los factores determinantes de la salud en Europa que ya se están produciendo. Aunque garantizar que el aumento de la temperatura no supere los 1,5 °C evitará algunas de las peores consecuencias del clima en la salud, el mundo ya se está acercando a este aumento de la temperatura y no está reduciendo adecuadamente las emisiones.

Se estima que las muertes relacionadas con el calor han aumentado en la mayor parte de Europa, con un incremento medio de 17,2 muertes por cada 100 000 habitantes entre los periodos 2003-2012 y 2013-2022 (indicador 1.1.4). Las horas de riesgo para la actividad física (debido al riesgo de estrés térmico) se han ido extendiendo más allá de las horas más calurosas del día durante el periodo 1990-2022

tanto para actividades de intensidad media (por ejemplo, ciclismo o fútbol) como para actividades extenuantes (por ejemplo, rugby o ciclismo de montaña) (indicador 1.1.3), lo que podría dar lugar a que las personas reduzcan su actividad física habitual y, por tanto, su riesgo de enfermedades no transmisibles aumente. La exposición al calor puede minar aún más la salud de las personas al incidir en los determinantes sociales y económicos de la salud. Por ejemplo, la oferta de mano de obra fue sustancialmente inferior durante 2016-2020 en comparación con el periodo de referencia de 1965-1994 (indicador 4.1.2). La idoneidad climática para diversos patógenos y vectores de enfermedades sensibles al clima ha aumentado en Europa (por ejemplo, Vibrio, virus del Nilo Occidental, dengue, chikungunya, Zika, malaria, leishmaniasis y garrapatas; indicador 1.3). Durante 2011-2020, se consideró que un número sustancialmente mayor de regiones serían aptas para la leishmaniasis (68 %) en comparación con 2001-2010 (55 %), con una expansión de dichas zonas hacia el norte, más allá de la zona históricamente endémica (indicador 1.3.5). El aumento relativo del riesgo de aparición de brotes fue del 256 % para el virus del Nilo Occidental entre 1951-1960 (riesgo de brote 0,05) y 2013-2022 (riesgo de brote 0,01; indicador 1.3.2), y del 40,9 % para el dengue entre 1951-1960 (R0 estimado 0,09) y 2013-2022 (R0 estimado 0,14; indicador 1.3.3). Además, el número de meses adecuados para las garrapatas Ixodes ricinus (el vector de la enfermedad de Lyme y la encefalitis transmitida por garrapatas), aumentó en 0,68 meses en Asia occidental y en 0,58 en Europa oriental. El cambio climático también está provocando cambios en la intensidad y frecuencia de los fenómenos climáticos extremos. Se observaron tendencias positivas en el peligro de incendios forestales en toda Europa durante 1980-2022 (indicador 1.2.1), aunque no se detectaron aumentos en los niveles de partículas en suspensión debidas a los incendios forestales (con diámetro ≤2,5 μm; PM2,5) entre 2003 y 2022 (indicador 1.2.1), lo que podría reflejar una preparación y gestión eficaces de dichos incendios. En Europa occidental, meridional y oriental se experimentaron aumentos sustanciales en las condiciones de sequía extrema entre 2000-2009 y 2010-2019 (indicador 1.2.2). Además, en 2021 el cambio climático provocó casi 12 millones más de personas afectadas por inseguridad alimentaria moderada o grave en Europa (indicador 1.5.1).

Agravamiento de las desigualdades sanitarias en un mundo cada vez más cálido

Estos impactos interrelacionados sobre la salud tienden a distribuirse de forma desigual entre las poblaciones debido a diferencias en la exposición, la sensibilidad y la capacidad de adaptación, que a menudo reflejan patrones interconectados de desarrollo socioeconómico y marginación, así como patrones históricos y actuales de desigualdad. Las poblaciones más afectadas suelen ser las menos responsables y las que tienen menos probabilidades de ser reconocidas o de que se les dé prioridad. El sur de Europa tiende a verse más afectado por las enfermedades relacionadas con el calor, los incendios forestales, la inseguridad alimentaria, la sequía y la leishmaniasis, mientras que el norte de Europa se ve igual o más afectado por Vibrio y las garrapatas (sección 1). Dentro de los países, las minorías étnicas y las poblaciones indígenas, las comunidades con bajos ingresos, los emigrantes y desplazados, las minorías sexuales y de género, y las mujeres embarazadas y parturientas tienden a verse más gravemente afectadas por los efectos del clima sobre la salud.

Este informe muestra que la mortalidad relacionada con el calor fue dos veces mayor en las mujeres que en los hombres (indicador 1.1.4), los hogares con bajos ingresos tuvieron una probabilidad sustancialmente mayor de sufrir inseguridad alimentaria (indicador 1.5.1), las muertes atribuibles a una dieta desequilibrada fueron mayores entre las mujeres (indicador 3.4.2) y la exposición a PM2,5 procedente de incendios forestales fue mayor en las zonas muy desfavorecidas. Las estrategias de adaptación mal diseñadas, tales como las soluciones basadas en la naturaleza (indicador 2.2.2) o los mecanismos para mejorar el confort térmico (indicador 2.2.3) que no tienen debidamente en cuenta la equidad, pueden perpetuar las desigualdadesmedioambientales y en la salud. Dado que no todos los indicadores permiten analizar las diferencias entre grupos de población, nuestro informe ofrece solo un atisbo de un panorama de desigualdades mucho más amplio y pone de manifiesto la importancia de realizar investigaciones más sólidas para

profundizar en los efectos desiguales del cambio climático sobre la salud, con el fin de fundamentar las medidas de protección sanitaria para todas las poblaciones.

A pesar de que el cambio climático agrava las desigualdades existentes, los indicadores sobre gobernanza y política muestran un escaso compromiso con los aspectos de igualdad, equidad o justicia en la investigación, la política y los medios de comunicación sobre el clima y la salud (sección 5). Además, la equidad medioambiental, que incluye abordar las distribuciones socio-geogràficas desproporcionadas de la exposición al cambio climático y de los riesgos para la salud, no es un objetivo explícito de las políticas actuales de la UE.

Asumir la responsabilidad y acelerar la acción

Muchos países europeos siguen siendo grandes contribuyentes históricos y actuales a las emisiones de gases de efecto invernadero. Mientras que los países europeos se han beneficiado del crecimiento económico que han permitido estas emisiones, otros países (los que menos han emitido) son los más afectados por el cambio climático actual y futuro. El cambio climático es un problema de justicia social y medioambiental. En 2021, las emisiones procedentes de la combustión de combustibles fósiles fueron de 5,4 toneladas de CO2 por persona en Europa, seis veces más que en África y casi tres veces más que en América Central y del Sur (indicador 3.1.1). El ritmo al que los países europeos avanzan hacia unas emisiones netas cero sigue siendo lamentablemente inadecuado y, con la trayectoria actual de Europa, el logro de la neutralidad de carbono solo se alcanzará en 2100 (indicador 3.1.1). Es importante destacar que, a través del consumo europeo de bienes y servicios producidos en otras partes del mundo, los países europeos siguen incrementando las presiones medioambientales (por ejemplo, las emisiones de gases de efecto invernadero y la contaminación atmosférica local) y sus correspondientes efectos adversos sobre el clima y la salud, en otras partes del mundo (indicador 3.2.1). A pesar de que varios países europeos han tomado medidas para reducir las emisiones del sector sanitario, se calcula que este sector habrá contribuido con 330 megatoneladas (Mt) de CO_2 equivalente (eq) en 2020 (indicador 3.5). Además, el uso del carbón aumentó hasta el 13 % del suministro energético total de Europa en 2021 (indicador 3.1.2), y 29 de 53 países siguen concediendo subvenciones netas a los combustibles fósiles (indicador 4.2.1).

Si no se toman medidas enérgicas, se corre el riesgo de exacerbar aún más los efectos del cambio climático que ya se están produciendo, y se pierden oportunidades de obtener considerables beneficios colaterales para la salud a corto plazo, como la reducción de la mortalidad prematura debida a la disminución de las partículas finas en el ambiente (indicador 3.2.1); el aumento de la actividad física gracias a un transporte más activo; y la reducción de la morbilidad y la mortalidad mediante la adopción de dietas basadas en vegetales, menos contaminantes, menos procesadas, más eficientes en el uso de los recursos y más saludables (indicador 3.4).

Limitar el calentamiento a menos de 1,5 °C para evitar más efectos perjudiciales para la salud exige que los gobiernos de toda Europa refuercen su respuesta. Por ello, las estructuras políticas y de gobernanza de toda Europa deben abordar las dimensiones sanitarias del cambio climático. Sin embargo, mientras que el compromiso científico (indicador 5.1) y del sector empresarial (indicador 5.4) siguió creciendo en 2022, los niveles de compromiso mediático (indicador 5.5), político (indicador 5.3) e individual (indicador 5.2) con el nexo clima-salud resultaron bajos. Teniendo en cuenta que el marco sanitario podría reforzar el apoyo público y político a la acción por el clima, así como la necesidad de que las sociedades europeas se adapten a los efectos del cambio climático sobre la salud, es esencial fomentar la concienciación sobre la salud climática entre los actores políticos y las instituciones para seguir estimulando la acción.

Una transición medioambiental justa y saludable

Para cumplir las recomendaciones del último informe del Grupo Intergubernamental de Expertos sobre el Cambio Climático (IPCC) de llegar a cero emisiones netas en 2040, las emisiones de los sistemas energéticos europeos

deberían reducirse a un ritmo aproximadamente tres veces superior al actual. Esta disminución deberá producirse aún más rápido si se utiliza el reparto justo de emisiones, que tiene en cuenta las emisiones históricas y la población de Europa, para asignar las reducciones a nivel mundial. Cuando se tiene en cuenta la justicia, la acción climática no solo garantiza una transición medioambiental justa y saludable, sino que también reduce las desigualdades en la salud, entre países y dentro de ellos, debidas a factores como la contaminación atmosférica, la actividad física derivada del transporte activo y las dietas saludables. Reconociendo las repercusiones del cambio climático dentro y fuera de Europa y el papel de ésta en la creación de la crisis climática, Europa debería comprometerse a una transición medioambiental justa y saludable, que incluya asumir una responsabilidad global y apoyar a las comunidades más afectadas.
