# Supplementary material for: The 2024 Europe report of the Lancet Countdown on health and climate change: unprecedented warming demands unprecedented action
Source: Lancet Public Health. 2024 May 12;9(7):e495–522. doi: 10.1016/S2468-2667(24)00055-0 (PMC11209670; doi:10.1016/S2468-2667(24)00055-0)
Supplement: Supplementary appendix 4 [file mmc4.pdf]

THE LANCET
Public Health

Supplementary appendix 4

This online publication has been corrected. The corrected version first appeared at thelancet.com/public-health on June 26, 2024

This appendix formed part of the original submission and has been peer reviewed. We post it as supplied by the authors.

Supplement to: van Daalen KR, Tonne C, Semenza JC, et al. The 2024 Europe report of the *Lancet* Countdown on health and climate change: unprecedented warming demands unprecedented action. *Lancet Public Health* 2024; published online May 12. https://doi.org/10.1016/S2468-2667(24)00055-0.

The 2024 Europe Report of the *Lancet* Countdown on Health and Climate Change: unprecedented warming demands unprecedented action

Appendix

Table of contents

Geographic definition of Europe 4

Summary of data utilised in each indicator 8

Overview with headline findings 11

Section 1: Climate change impacts, exposures, and vulnerabilities 17

1.1: Heat and health 17

Indicator 1.1.1: Vulnerability to heat exposure 17

Indicator 1.1.2: Exposure of vulnerable populations to heatwaves 20

Indicator 1.1.3: Physical activity related heat stress risk 24

Indicator 1.1.4: Heat-related mortality 32

1.2: Extreme events and Health 40

Indicator 1.2.1: Wildfire smoke 40

Indicator 1.2.2: Drought 49

1.3: Climate-sensitive infectious diseases 57

Indicator 1.3.1: Climatic suitability for *non-cholerae* Vibrio 57

Indicator 1.3.2: Climatic suitability for West Nile virus 61

Indicator 1.3.3: Climatic suitability for dengue (chikungunya, Zika) 65

Indicator 1.3.4: Climatic suitability for Malaria 74

Indicator 1.3.5: Climatic suitability for leishmaniasis 83

Indicator 1.3.6: Climatic suitability for ticks 93

1.4: Allergens 100

Indicator 1.4.1: Allergenic trees 100

1.5: Food and water 111

Indicator 1.5.1: Food security 111

Section 2: Adaptation, planning and resilience for health 114

Panel 2.1: Nature-based solutions 114

2.1: Adaptation planning and assessment 118

Indicator 2.1.1: National vulnerability and adaptation assessments 118

Indicator 2.1.2: National adaptation plans for health 118

Indicator 2.1.3: City-level climate change risks assessments 121

2.2: Enabling conditions, adaptation delivery and implementation 122

Indicator 2.2.1: Climate information services for health 118

Indicator 2.2.2: Green space 122

Indicator 2.2.3: Air conditioning benefits and harms 130

Section 3: Mitigation actions and health co-benefits 131

3.1: Energy system and health 131

Indicator 3.1.1: Carbon intensity of the energy system 131

Indicator 3.1.2: Coal phase-out 133

Indicator 3.1.3: Renewable and zero-carbon emission electricity 135

3.2: Air pollution and health co-benefits 137

Indicator 3.2.1: Premature mortality attributable to ambient fine particles 137
Indicator 3.2.2: Production-based and consumption-based attribution of CO_2 and $PM_{2.5}$ emissions 142
3.3: Sustainable and healthy transport 143
3.4: Food, agriculture and health 145
Indicator 3.4.1: Life cycle emissions from food demand, production and trade 145
Indicator 3.4.2: Sustainable diets 147
3.5: Health sector emissions and harms 155
Section 4: Economics and finance 156
4.1: The health linked economic impacts of climate change and its mitigation 156
Indicator 4.1.1: Economic losses due to climate-related extreme events 156
Indicator 4.1.2: Change in labour supply 159
Indicator 4.1.3: Impact of heat on economic activity 160
Indicator 4.1.4: Monetised value of unhealthy diets 162
4.2: The economics of the transition to zero-carbon economies 164
Indicator 4.2.1: Net value of fossil fuel subsidies and carbon prices 164
Indicator 4.2.2: Clean energy investment 166
Section 5: Politics and governance 168
5.1: Scientific engagement with health and climate change 168
Indicator 5.1.1: Coverage of health and climate Change in scientific articles 168
Indicator 5.1.2: Coverage of the health impacts of anthropogenic climate change in scientific articles 170
5.2: Individual engagement with health and climate change on social media 173
5.3: Political engagement with health and climate change 187
Indicator 5.3.1: Engagement with health and climate change in the European Parliament 187
Indicator 5.3.2: Political engagement with health and climate change on social media 219
5.4: Corporate sector engagement with health and climate change 234
5.5: Media engagement with health and climate change 241
References 256

Geographic definition of Europe

In this European *Lancet* Countdown indicator report, we aimed to include all 53 World Health Organisation (WHO) European region countries (**see table 2**) plus Liechtenstein and Kosovo (under UNSC resolution 1244), where possible. However, due to data availability constrains, in practise, we include predominantly all 38 European Environment Agency (EEA) member states and cooperating countries (**see table 1**) plus the United Kingdom of Great Britain and Northern Ireland, or the 27 European Union countries plus the United Kingdom of Great Britain and Northern Ireland across the different indicators. As data availability varies depending on the indicator, we provide a description of the geographic coverage of Europe utilised for each individual indicator in the relevant appendix sections. **Table 3** and **table 4** provide an overview of the member countries that are part of the European Union (EU) and the European Trade Association (EFTA).

Some of the indicators report outcomes by European sub-regions (Eastern Europe, Northern Europe, Southern Europe, Western Europe, Central Asia and Western Asia). Unless stated otherwise, these follow the classification as provided by the UN geoscheme (**table 5**).

Table 1 European Environment Agency (EEA) member and cooperating countries[1]

**Member countries**			
Austria	Belgium	Bulgaria	Croatia
Cyprus	Czechia	Denmark	Estonia
Finland	France	Germany	Greece
Hungary	Iceland	Ireland	Italy
Latvia	Liechtenstein	Lithuania	Luxembourg
Malta	Netherlands (Kingdom of the)$^{\pm}$	Norway	Poland
Portugal	Romania	Slovakia	Slovenia
Spain	Sweden	Switzerland	Türkiye
**Cooperating countries**			
Albania	Bosnia and Herzegovina	Kosovo (UNSCR 1244)*	Montenegro
North Macedonia	Serbia		

** Designation is without prejudice to position on status and is in line with United Nations Security Council resolution (UNSCR) 1244 and the ICJ Opinion on the Kosovo Declaration of independence.*

$^{\pm}$ Unless stated otherwise, this report only includes the country of the Netherlands and excludes the Caribbean part of the Netherlands (Kingdom of the). Therefore, the main report refers to "the Netherlands" instead of "the Netherlands (Kingdom of the)".

Table 2 World Health Organisation (WHO) European region member countries[2]

Albania	Andorra	Armenia	Austria	Azerbaijan
Belarus	Belgium	Bosnia and Herzegovina	Bulgaria	Croatia
Cyprus	Czechia	Denmark	Estonia	Finland
France	Georgia	Germany	Greece	Hungary
Iceland	Ireland	Israel	Italy	Kazakhstan
Kyrgyzstan	Latvia	Lithuania	Luxembourg	Malta
Monaco	Montenegro	Netherlands (Kingdom of the)	North Macedonia	Norway
Poland	Portugal	Republic of Moldova	Romania	Russian Federation
San Marino	Serbia	Slovakia	Slovenia	Spain
Sweden	Switzerland	Tajikistan	Türkiye	Turkmenistan
Ukraine	United Kingdom of Great Britain and Northern Ireland	Uzbekistan		

Table 3 European Union (EU) member and candidate countries[3,4]

**Member countries**			
Austria	Belgium	Bulgaria	Croatia
Cyprus	Czechia	Denmark	Estonia
Finland	France	Germany	Greece
Hungary	Ireland	Italy	Latvia
Lithuania	Luxembourg	Malta	Netherlands (Kingdom of the)
Poland	Portugal	Romania	Slovakia
Slovenia	Spain	Sweden	
**Candidate countries**			
Albania	North Macedonia	Montenegro	Serbia
Türkiye			
**Potential candidate countries**			
Bosnia and Herzegovina	Kosovo (UNSCR 1244)*	Iceland	

Table 4 European Free Trade Association (EFTA) countries[5]

**Member countries**			
Iceland	Liechtenstein	Norway	Switzerland

Table 5 Sub-regions of Europe following the UN geoscheme

**Eastern Europe**			
Belarus	Bulgaria	Czechia	Hungary
Poland	Republic of Moldova	Romania	Russian Federation
Slovakia	Ukraine		
**Northern Europe**			
Denmark	Estonia	Finland	Iceland
Ireland	Latvia	Lithuania	Norway
Sweden	United Kingdom of Great Britain and Northern Ireland		
**Southern Europe**			
Albania	Andorra	Bosnia and Herzegovina	Croatia
Greece	Italy	Kosovo (UNSCR 1244)*	Malta
Montenegro	North Macedonia	Portugal	San Marino
Serbia	Slovenia	Spain	
**Western Europe**			
Austria	Belgium	France	Germany
Liechtenstein	Luxembourg	Monaco	Netherlands (Kingdom of the)
Switzerland			
**Central Asia**			
Kazakhstan	Kyrgyzstan	Tajikistan	Turkmenistan
Uzbekistan			
**Western Asia**			
Armenia	Azerbaijan	Cyprus	Georgia
Israel	Türkiye		

Figure 1 European subregions in the Lancet Countdown in Europe.

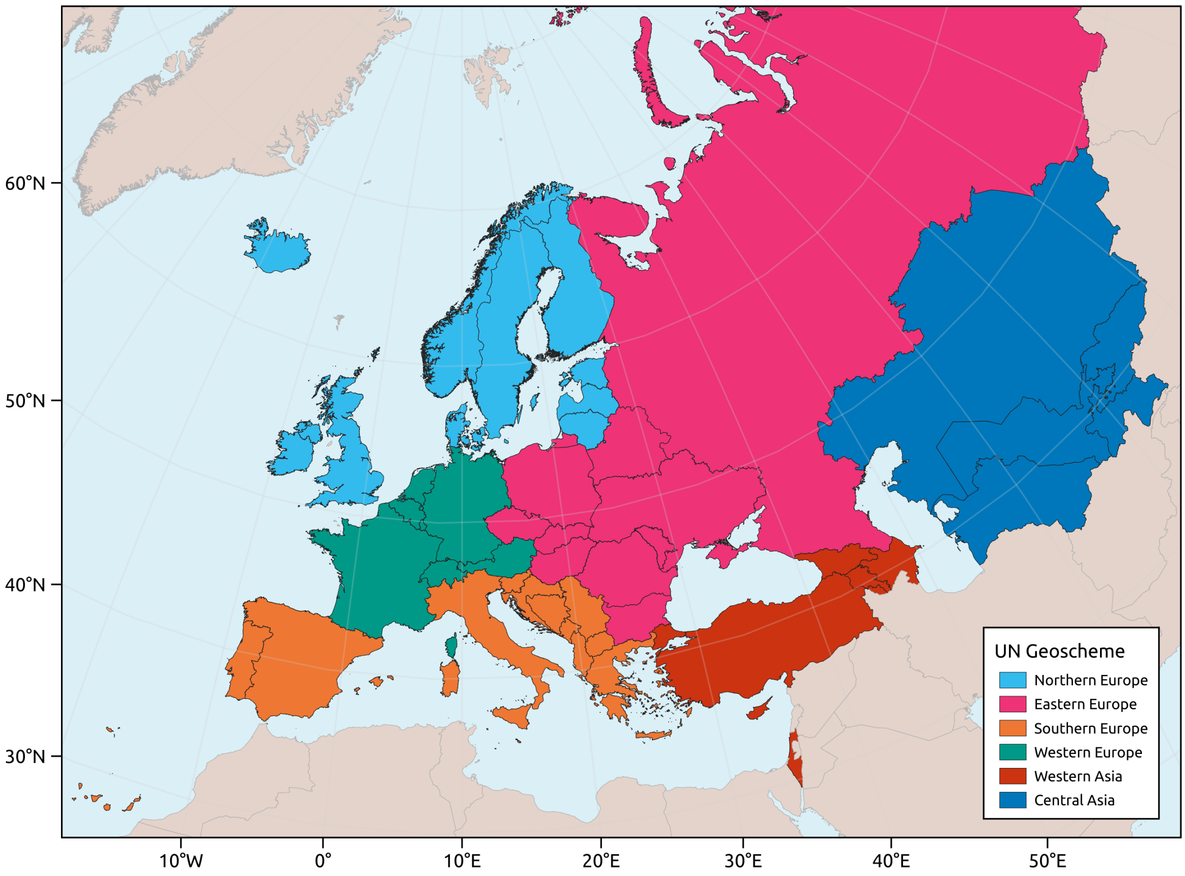

Summary of data inputs for each indicator

Table 5 Data inputs for each indicator

Indicator	Data inputs
**1. Climate change impacts, exposures, and vulnerabilities**	
1.1 Heat and health	
1.1.1 Vulnerability to heat exposure	- Population estimates, GBD<br>- DALYs and deaths, GBD<br>- Urban population, UNDP Urbanization Prospects
1.1.2 Exposure of vulnerable populations to heatwaves	- Climatic data, ERA-5 Land reanalysis<br>- Population data, NASA SEDAC GPWv4 & Eurostat demographic census data
1.1.3 Physical activity related heat stress risk	- Climatic data, ERA-5 Land reanalysis<br>- Population data, Eurostat GEOSTAT
1.1.4 Heat-related mortality	- Climatic data, ERA-5 Land reanalysis<br>- Population data, Eurostat<br>- Mortality data, Eurostat
1.2 Extreme events and Health	
1.2.1 Wildfire smoke	- Population data, Eurostat GEOSTAT<br>- Geographic data, Eurostat GISCO<br>- Mortality, Eurostat<br>- Fire smoke dispersion forecasts, IS4FIRES & SILAM models based on Thermal Anomalies and Fire MOD14/MYD14<br>- Fire weather index
1.2.2 Drought	- Climatic data, ERA-5 Land reanalysis<br>- Water scarcity data, WEI+ by EEA
1.3 Climate-sensitive infectious diseases	
1.3.1 Climate suitability *non-cholerae* Vibrio	- Population data, Eurostat GEOSTAT<br>- Sea surface temperature data, GHRSST Level 4 MUR Global Foundation Sea Surface Temperature Analysis (v4.1)<br>- Sea surface salinity data, Ocean Physical Reanalysis and Analysis EU Copernicus Marine Service
1.3.2 Climatic suitability for West Nile virus	- Climatic data, ERA-5 Land reanalysis<br>- WNV infections data, ECDC
1.3.3 Climatic suitability for dengue (chikungunya, Zika)	- Climatic data, ERA-5 Land reanalysis & C3S<br>- Population data, Hybrid gridded data combining NASA SEDAC GPWv4 with ISIMIP Histsoc and World Population Prospects demographic modelling data
1.3.4 Climate suitability for malaria	- Climatic data, ERA-5 Land reanalysis<br>- Land cover, CORINE<br>- Altitude data, JISAO
1.3.5 Climatic suitability for Leishmaniasis **[new]**	- Leishmaniasis data, ECDC<br>- Vector data, ECDC<br>- Climatic data, ERA-5 Land reanalysis<br>- Land cover, CORINE<br>- Elevation data, WorldClim (SRTM)
1.3.4 Climatic suitability for ticks **[new]**	- *Ixodes rinicus* presence, GBIF<br>- Temperature data, ERA-5 Land reanalysis<br>- Land cover, CORINE
1.4 Allergens	
1.4.1 Allergenic trees	- Climatic data, ERA-5 Land reanalysis<br>- Land cover data, ECOCLIMAP, EFI and GLC-SHARE<br>- Pollen data, European Aeroallergen Network
1.5 Food and water	
1.5.1 Food security **[new]**	- Temperature & SPEI, ERA-5 Land reanalysis<br>- Food security data, FAO FIES
**2. Adaptation, planning and resilience for health**	
2.1 Adaptation planning and assessment	

2.1.1 National assessments of climate change impacts, vulnerability and adaptation for health	- Questionnaire data, WHO Health and Climate Change Survey
2.1.2 National adaptation plans for health	- Questionnaire data, WHO Health and Climate Change Survey
2.2. Enabling conditions, adaptation delivery and implementation	
2.2.1 Climate information services for health	- Questionnaire data, WHO Health and Climate Change Survey
2.2.2 Green space	- Population data, Eurostat<br>- NDVI data, MOD13Q1 from Terra MODIS<br>- Deprivation data, Eurostat
2.2.3 Air conditioning benefits and harms	- Airconditioning data, IEA
Panel 3: Nature-based solutions	- Urban Nature Atlas.
**Section 3. Mitigation actions and health co-benefits**	
3.1. Energy system and health	
3.1.1 Carbon intensity of the energy system	- CO_2 Emission from Fuel Combustion dataset, IEA
3.1.2 Coal phase-out	- World Extended Energy Balances dataset, IEA
3.1.3 Renewable and zero-carbon emission electricity	- World Extended Energy Balances dataset, IEA
3.2 Air pollution and health co-benefits	
3.2.1 Premature mortality attributable to ambient fine particles	- Energy consumption, Eurostat & IEA<br>- Agricultural activity, FAOSTAT<br>- Fertiliser use, IFASTAT<br>- Municipal waste, GAINS internal calculations<br>- Mortality data, Eurostat & UN World Population Prospects
3.2.2 Production-based and consumption-based attribution of CO_2 and $PM_{2.5}$ emissions **[new]**	- Multi-region environmentally extended input-output tables: EXIOBASE<br>- National total production-based CO_2 emissions: the Global Carbon Project 2021<br>- Change ratio of countries' GDPs<br>- Change ratio of countries' exports: WTOSTAT, World Trade Organization<br>- $PM_{2.5}$ emission inventory: GAINS<br>- $PM_{2.5}$ emission inventory: EDGAR database<br>- Population data: World Bank
3.3 Sustainable and healthy transport	- Fuel use, World Extended Energy Balances dataset, IEA<br>- Population data, UN World Population Prospects<br>- Model split of passenger transport, Eurostat
3.4. Food, agriculture and health	
3.4.1 Life cycle emissions from food demand, production and trade	- Food demand, FAO's Food Balance Sheets<br>- Life-cycle emissions, Poore & Nemecek 2018
3.4.2 Sustainable diets	- Population data, GBD 2019<br>- Mortality data, GBD 2019<br>- Food consumption, FAO's Food Balance Sheets<br>- Health estimates, meta-analysis of prospective cohort studies
***3.5 Health sector emissions and harms* [new]**	- Environmentally extended multi-region input-output tables: WIOD 2013<br>- Per capita health expenditure, WHO's Global Health Expenditure Database<br>- Market exchange rates, UN Statistics Division<br>- Consumer price indices, World Bank<br>- Healthy life expectancy at birth, WHO's Global Health Observatory
**Section 4: Economics and finance**	
4.1. The health linked economic impacts of climate change and its mitigation	
4.1.1 Economic losses due to climate-related extreme events	- Swiss Re Institute sigma catastrophe database<br>- IMF World Economic Outlook
4.1.2 Change in labour supply	- Climatic data, ERA-5 Land reanalysis<br>- Labour data, Eurostat Regional Database from the EU Labour Force Survey
4.1.3 Impact of heat on economic activity	- Climatic data, ERA-5 Land reanalysis<br>- GDP growth data, Eurostat Regional Database from the EU Labour Force Survey
4.1.4 Monetised value of unhealthy diets	- Values of statistical life, OECD

	- Diet-related health impacts from indicator 3.4.2
4.2. The economics of the transition to zero-carbon economies	
4.2.1 Net value of fossil fuel subsidies and carbon prices	- Fossil fuel subsidies, IEA, OECD<br>- Carbon pricing, World Bank Carbon Pricing Dashboard<br>- CO2 emissions from fuel, IEA<br>- Health expenditure, WHO,<br>- US Dollar GDP deflator index, IMF World Economic Outlook database
4.2.2 Clean energy investment **[new]**	- World Energy Investment 2023, IEA
**Section 5: Politics and governance**	
5.1 Scientific engagement with health and climate change	
5.1.1 Coverage of health and climate Change in scientific articles	- Publication data, Scientific databases, Scopus, Medline, Web of Science Core Collection
5.1.2 Coverage of studies on the health impacts of anthropogenic climate change in scientific articles **[new]**	- Publication data, Scientific databases, Scopus, Medline, Web of Science Core Collection<br>- Temperature data, HADCRUT4<br>- Precipitation data, GPCC
5.2 Individual engagement with health and climate change on social media	- Twitter data, Twitter Developer API for the Academic purposes
5.3 Political engagement with health and climate change	
5.3.1 Engagement with health and climate change in the European Parliament	- EU parliament debates transcripts
5.3.2 Political engagement with health and climate change on social media **[new]**	- Twitter data, Open-source Python Library Tweepy (uses Twitter API V2)
5.4 Corporate sector engagement with health and climate change	- UN GCCOP reports
5.5 Media engagement with health and climate change **[new]**	- 169 media outlets (Table 5.6)

ABBREVIATIONS:

CORINE; Copernicus Land Monitoring Service, **C3S**; Copernicus Climate Change Service, **CO_2**; Carbon Dioxide, **DALY**; Disability-Adjusted Life Years, **ECDC**; European Centre for Disease Prevention and Control, **EDGAR**; Emission Database for Global Atmospheric Research, **EEA**; European Environment Agency, **EFI**; European Forest Institute, **EU**; European Union, Eurostat; European Statistical Office, **FAO**; Food and Agriculture Organization, **FAOSTAT**; Food and Agriculture Organization Corporate Statistical Database, **FIES**; Food Insecurity Experience Scale, **GAINS**; Greenhouse Gas-Air Pollution Interactions and Synergies, **GBD**; Global Burden of Disease, **GBIF**; Global Biodiversity Information Facility, **GCCOP**; Global Compact Communication Progress; **GDP**; Gross Domestic Product, **GISCO**; Geographical Information System, **GLC**; Global Land Cover, **GPW**; Gridded Population of the World, **GHRSST**; Group for High-Resolution Sea Surface Temperature, **GPCC**; Global Precipitation Climatology Centre, **IEA**; International Energy Agency, **IFASTAT**; International Fertilizer Association Statistics, **IMF**; International Monetary Fund, **ISIMIP**; Inter-Sectoral Impact Model Intercomparison Project, **IS4FIRES**; Integrated Monitoring and Modelling System for Wildland Fires, **JISAO**; Joint Institute for The Study of Atmosphere and Ocean, **MODIS**; Moderate Resolution Imaging Spectroradiometer, **MUR**: Multiscale Ultrahigh Resolution, **NASA**; National Aeronautics and Space Administration; **NVDI**; Normalised Vegetation Index, **OECD**; Organisation for Economic Co-operation and Development, **$PM_{2.5}$**; Particulate matter $\leq$ 2.5 µm**,** **SEDAC**; Socioeconomic Data and Applications Center, **SILAM**; System for Integrated Modelling of Atmospheric Composition, **SRTM**; Shutter Radar Topography Mission, **TCD**; Tree Cover Density, **UN**; United Nations, **UNDP**; United Nations Development Programme, **WEI+**; Water Exploitation Index plus, **WHO**; World Health Organization, **WNV**; West-Nile Virus

Overview with headline findings

In the following section we provide an overview with headline findings for each individual indicator included in the 2024 *Lancet* Countdown in Europe report.

Section 1: Climate change impacts, exposures, and vulnerabilities

1.1: Heat and health

Indicator 1.1.1: Vulnerability to heat exposure

Vulnerability to heat exposure has increased by 9% across all regions in Europe (1990-2020). Western Asia followed by southern Europe regions registered the greatest relative increase compared to 1990.

Indicator 1.1.2: Exposure of vulnerable populations to heatwaves

There was a 97% relative increase in the total number of person-days of heatwave exposure in the last decade (2012-2021) compared to the previous decade (2000-2009), increasing from 650 million to a total of 1.28 billion person-days.

Indicator 1.1.3: Physical activity related heat stress risk

Heat stress risk during physical activity has increased beyond the hottest part of the day. Comparing 2010-2020 to 1990-2000, the number of risky hours outside the hottest part of the day increased in all European regions. Southern Europe was the most affected region: in 2022, risky hours per person were 182 within the hottest part of the day and 358 outside the hottest part of the day.

Indicator 1.1.4: Heat-related mortality

Heat attributable mortality rate increased 17.2 deaths per 100 000 million inhabitants in Europe during the last decade (2013-2022) compared to the previous decade (2003-2012). Increase in heat-related mortality was almost twice as high in women compared to men.

Anthropogenic warming has caused a fourfold increase in the probability of extreme heat-related mortality episodes observed during 2003-2022, with geographical and sex differences.

1.2: Extreme events and Health

Indicator 1.2.1: Wildfire smoke

Wildfire danger has been increasing in most of Europe while trends in wildfire smoke ($PM_{2.5}$) exposure remain inconclusive. The most affected countries in terms of wildfire danger, wildfire smoke, and attributable mortality were in southern and eastern Europe.

Indicator 1.2.2: Drought

A substantial increase in moderate, severe and extreme summer drought conditions was observed in western Europe when comparing 2010-2019 with 2000-2009.

1.3: Climate-sensitive infectious diseases

Indicator 1.3.1: Climatic suitability for *non-cholerae* Vibrio

A total of 21 countries in Europe showed suitable conditions for Vibrio in 2022 with an accumulated number of days per country of 2188, the 3rd highest number in record, and 28263 km of coast (the 3rd highest since records began), showing a consistent increase over the period with an extension of 136 new km per year in Europe.

Indicator 1.3.2: Climatic suitability for West Nile virus

The risk of West Nile virus outbreaks is steadily increasing across Europe. The relative increase in WNV outbreak risk in 2013-2022 compared to 1951-1960 baseline was 256%, with the highest relative risk increase seen in eastern Europe (516%) and southern Europe (203%).

Indicator 1.3.3: Climatic suitability for dengue (chikungunya, Zika)

The transmission suitability R_0*, has increased by 55.9% in the current decade 2013-2022 compared to the 1951-1960 baseline with the greatest absolute increase observed in southern Europe (6.88%), followed by eastern Europe (6.65%). The absolute risk in northern and western Europe increased by 1.7% and 6.10% respectively, compared to baseline of 1951-1960.*

Total dengue cases imported in locations in Europe where conditions are suitable for dengue transmission have increased by 176.8% across Europe in 2009-2019 compared to 1995-2004.

Indicator 1.3.4: Climatic suitability for Malaria

The climatic suitability for P. vivax transmission has increased by 0.34 months in western Europe and 0.22 months in eastern Europe between 1951-1960 and 2013-2022. Simultaneously, there has been a consistent rise in the number of malaria importation events to areas with suitable conditions.

Indicator 1.3.5: Climatic suitability for leishmaniasis

Climatic suitability for leishmaniasis has increased in Europe in the last two decades (2001-2020), with more noticeable changes in southern and eastern European countries. New localities have been identified as suitable northward of the historical endemic zone.

Indicator 1.3.6: Climatic suitability for ticks

Eastern Europe and western Asia exhibited the highest suitability for Ixodes ricinus feeding activity, particularly in rural areas and areas with high social deprivation levels. In total, 1455 (96%) of 1514 NUTS3 regions monitored increased in suitability during 2013-2022 compared to 1951-1960.

1.4: Allergens

Indicator 1.4.1: Allergenic trees

Birch, alder and olive tend towards earlier flowering, especially in mountains (Alps, Balkans, Scandinavian ridge), where the season currently (2022) starts over a month earlier than 33 years ago (1990). Furthermore, the season duration remained nearly the same in length across most of Europe, despite the start and end of the pollen season shifting.

1.5: Food and water

Indicator 1.5.1: Food security

Nearly 60 million people had moderate or severe food insecurity in Europe in 2021. Around 11.9 million of these can be attributed to a higher number of heatwave days and droughts months compared with the average during 1981-2010.

Section 2: Adaptation, planning and resilience for health

2.1: Adaptation planning and assessment

Indicator 2.1.1: National vulnerability and adaptation assessments

10 of 22 (45%) reporting countries reported conducting Vulnerability & Adaptation assessments by 2020. Only two (20%) and one (10%) assessment(s) were reported to strongly result in the development of new or revision of existing health policies or programmes, or to strongly influence the allocation of human and financial resources to address the health risks of climate change, respectively.

Indicator 2.1.2: National adaptation plans for health

10 (45%) of 22 reporting countries had an agreement between the Ministry of Health and the environment sector and 9 (41%) with meteorological/hydrological services.

Indicator 2.1.3: City-level climate change risks assessments

In 2022 149 (81%) of 185 responding European cities reported to have conducted a climate risk assessment, 12 (6%) reported that an assessment was in progress, and 22 (12%) that an assessment will be undertaken in the next two years. Elderly, vulnerable health groups, children and youth, low-income households, outdoor workers, marginalised communities, women and girls, frontline workers, and Indigenous peoples were identified as most vulnerable to climate hazards.

2.2: Enabling conditions, adaptation delivery and implementation

Indicator 2.2.1: Climate information services for health

Whilst most European countries have health surveillance systems for specific health outcomes, few incorporate climate information (i.e., climate-informed surveillance systems). A moderate number of health early warning systems (HEWS) are climate informed.

Indicator 2.2.2: Green space

On average, population-weighted NDVI increased by 2% during 2000-2022 in Europe. In some areas there was a significant increase of more than 0.1 in the population-weighted greenness, particularly near the borders between Albania, Hungary, Romania and Serbia.

Indicator 2.2.3: Air conditioning benefits and harms

In 2021, air conditioning provided cooling in 16% of European households, consuming about 159 Terawatt-hour (TWh) of electricity and producing 45 megatonnes (Mt) of CO_2 emissions – approximately the same as the total CO_2 emissions of the whole of Bulgaria in 2021.

Section 3: Mitigation actions and health co-benefits

3.1: Energy system and health

Indicator 3.1.1: Carbon intensity of the energy system

While the carbon intensity of the energy system in Europe continues to decrease, the pace of change is far too slow, with the current trajectory consistent with achieving net-zero by 2100 rather than the legally binding target of 2050.

Indicator 3.1.2: Coal phase-out

The contribution of coal to Europe's total energy supply increased by 1% from 2020 to 2021, driven by a slowdown of coal phase out due to the Russian invasion of Ukraine.

Indicator 3.1.3: Renewable and zero-carbon emission electricity

The transition towards zero-carbon emission energy sources is underway; however, these sources account for only around 20% of the total energy supply in Europe.

3.2: Air pollution and health co-benefits

Indicator 3.2.1: Premature mortality attributable to ambient fine particles

Air pollution related deaths caused by emissions from power, transport and household sectors have decreased by 59% between 2005 and 2020. Most of this decrease is due to structural changes and technical controls, the contribution from fuel switches is smaller and partly negative.

Indicator 3.2.2: Production-based and consumption-based attribution of CO_2 and $PM_{2.5}$ emissions

In 2021, Europe's consumption-based CO_2 and $PM_{2.5}$ emissions exceeded its production-based ones by 1.0 percentage point and 1.6 percentage point, respectively. The emissions embodied in Europe's imports accounted for 19.2% of its consumption-based CO_2 emissions and 30.8% of its consumption-based $PM_{2.5}$ emissions, ranking highest among all regions.

3.3: Sustainable and healthy transport

There was a substantial shift in transport mode during the COVID-19 pandemic, with a 5% increase in care usage observed from 2019 to 2020. Most probably reflecting the perceived safety of private vehicles during the pandemic.

3.4: Food, agriculture and health

Indicator 3.4.1: Life cycle emissions from food demand, production and trade

From 2010 to 2020, there was little progress in reducing emissions associated with European food demand, which in 2022 amounted to 1.85 GtCO2-eq. Animal-sourced food was predominantly responsible.

Indicator 3.4.2: Sustainable diets

About 2.5 million deaths in Europe were attributable to imbalanced diets in 2020, representing almost a third (30%) of all deaths amongst adults. Between 2010 and 2020, the proportion of diet-related deaths only decreased by 1 percentage points.

3.5: Health sector emissions and harms

In 2020, it was estimated that the healthcare sector of the WHO European region contributed approximately 330 $MtCO_2e$ (356 $kgCO_2e$ per person) in emissions. Air pollution related to healthcare was estimated to result in a total of 540 thousand DALYs.

Section 4: Economics and finance

4.1: The health linked economic impacts of climate change and its mitigation

Indicator 4.1.1: Economic losses due to climate-related extreme events

In 2022, economic losses due to weather-related extreme events in Europe was €18.7 billion, representing 0.08% of Europe's GDP of which 44.2% (€8.2 billion) was uninsured.

Indicator 4.1.2: Change in labour supply

Labour supply in high-exposure sectors was 1.05% (~17 hours per worker per year) lower in 2016-2020 compared to 1965-1994 due to global warming. In comparison, in 1995-2000 labour supply was only 0.22% lower (~4 hours per worker per year).

Indicator 4.1.3: Impact of heat on economic activity

In 2020, GDP per capita growth in Southern Europe was estimated to be 0.98% (95% CI: -0.97, -1.00) lower than it would otherwise have been if there had been no warming since 1981-2010.

Indicator 4.1.4: Monetised value of unhealthy diets

The monetised value of imbalanced diets amounted to €9·2 trillion in 2020, which was highest in eastern Europe followed by southern Europe.

4.2: The economics of the transition to zero-carbon economies

Indicator 4.2.1: Net value of fossil fuel subsidies and carbon prices

Net fossil fuel subsidies of €61.6 billion were recorded in Europe in 2020, corresponding to an average net negative carbon price of -11.4 €/t. Only 14 countries had net-positive carbon prices, while 29 had net-negative prices.

Indicator 4.2.2: Clean energy investment

Clean energy investment exceeded fossil fuel investment in Europe by 261% in 2022, and was16% higher than in 2021 and 66% higher than in 2015.

Section 5: Politics and governance

5.1: Scientific engagement with health and climate change

Indicator 5.1.1: Coverage of health and climate change in scientific articles

In 2022, 340 studies on the nexus between climate and health were identified in Europe, with 91% focused on the impacts, compared to 4% on mitigation and 5% on adaptation actions.

Indicator 5.1.2: Coverage of studies on the health impacts of anthropogenic climate change in scientific articles

Of the 6,276 articles on of the impact climate change on health in Europe during 1990-2022, 66% (4,134) studies where identified where long-term changes in climatic factors can be attributed to anthropogenic climate change.

5.2: Individual engagement with health and climate change on social media

In 2022, only 0.4% (10,037/2,490,601) English-language Tweets and 0.5% (30,944/6,156,957) non-English language Tweets (both geolocated in some of the largest European cities) engaged with climate change and health. Of these only 0.05% referenced issues related to equality, equity, and justice.

5.3: Political engagement with health and climate change

Indicator 5.3.1: Engagement with health and climate change in the European Parliament

Whilst there were over 800 references to climate change in legislators' speeches and over 1,400 references to health in 264058 speeches assessed, there were only 10 (0.1%) references to the intersection of health and climate change in the European Parliament in 2022. Of these, only two included inequality-related terminology.

Indicator 5.3.2: Political engagement with health and climate change on social media

During 2018-2022 there was very limited online engagement by governments with the intersection of health and climate change, with only 0.05% of 703,792 government tweets mentioning the intersection.

5.4: Corporate sector engagement with health and climate change

A substantial increase in engagement with the health dimensions of climate change was observed over 2011-2022, with 37% of corporations referencing the climate change-health intersection in 2022 compared to only 18% in 2019. References to gender or inequality in relation to the climate change-health intersection likewise increased.

5.5: Media engagement with health and climate change

Throughout 2022, media engagement with health and climate change was low across countries (8.2%, 44,766/3,727,118 multilingual Tweets), with hardly any engaging with inequality (0.19%).

Section 1: Climate Change Impacts, Exposures, and Vulnerability

1.1: Heat and Health

Indicator 1.1.1: Vulnerability to Extremes of Heat

Geographic Coverage of Europe

For this indicator, European Environment Agency (EEA) member and cooperating countries plus the United Kingdom of Great Britain and Northern Ireland were included.

Data

1. Institute for Health Metrics and Evaluation (IHME). Global Fertility, Mortality, Migration, and Population Forecasts 2017-2100. Seattle, United States of America: Institute for Health Metrics and Evaluation (IHME), 2020.
2. Global Burden of Disease Collaborative Network. Global Burden of Disease Study 2019 (GBD 2019) Results. Seattle, United States: Institute for Health Metrics and Evaluation (IHME), 2020.Available from http://ghdx.healthdata.org/gbd-results-tool.
3. United Nations, Department of Economic and Social Affairs, Population Division (2018). World Urbanization Prospects: The 2018 Revision, Online Edition. Annual Percentage of Population at Mid-Year Residing in Urban Areas by region, subregion and country, 1950-2050.

Methods

This indicator displays an index derived by taking mean of proportion of the population over 65 years (1);[6] the prevalence of cardiovascular, diabetes and chronic respiratory diseases among population over 65years GBD study 2019 estimates (2)[7] and the proportion of the population living in urban areas (3)[8] as a measure of exposure to urban heat island. The index ranges between 0 and 100 and is a measure of potential vulnerability of a country to heat exposure. Aggregated trends by European regions are presented for the period 1990 to 2019.Percentage change in vulnerability was also computed taking 1990 as the baseline year. The country level estimates are provided in the appendix.

Caveats

There is no consistent and universally accepted standard for distinguishing urban from rural areas, in part because of the wide variety of situations across countries. Most countries use an urban classification related to the size or characteristics of settlements. This indicator does not include the existence of heat early warning systems, or prevalence of cooling devices. Neither does it include the prevalence of green areas in cities.

Future Form of the Indicator

GBD and urbanization estimates now are revised annually; the indicator will be updated every year.

Findings & Additional Analysis

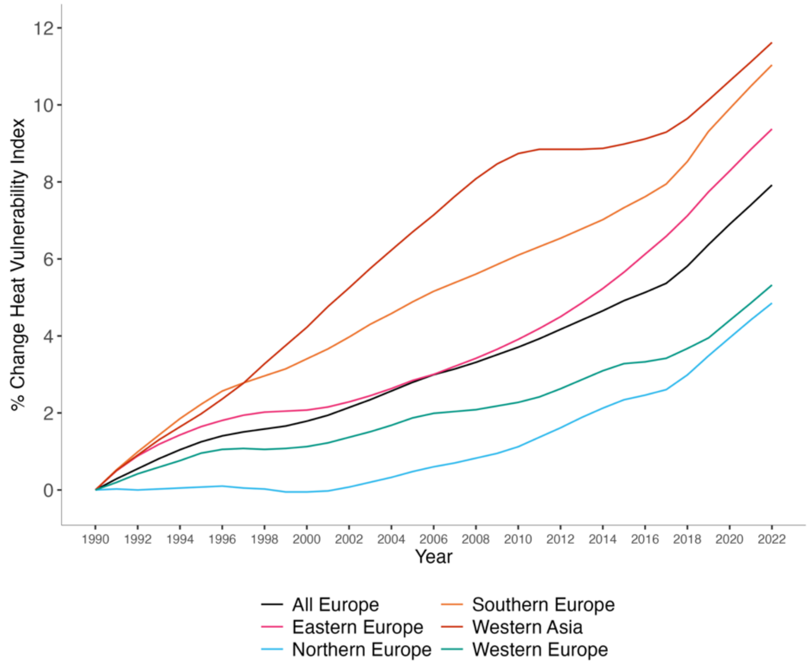

Figure 1.1 Percentage changes in vulnerability to heat by European regions 1990-2022 with 1990 baseline.

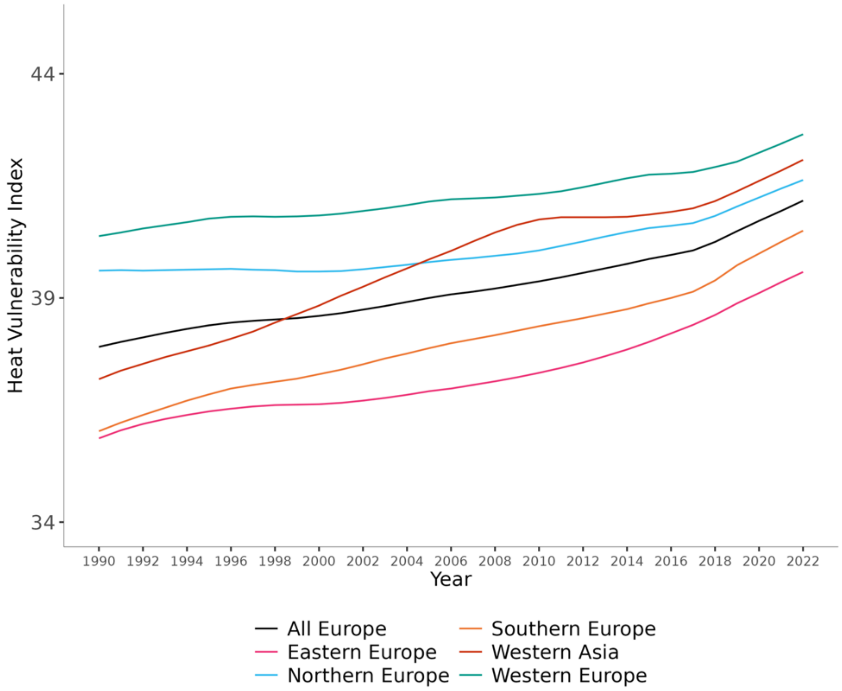

Figure 1.2 Trends in vulnerability to heat by European regions 1990-2022.

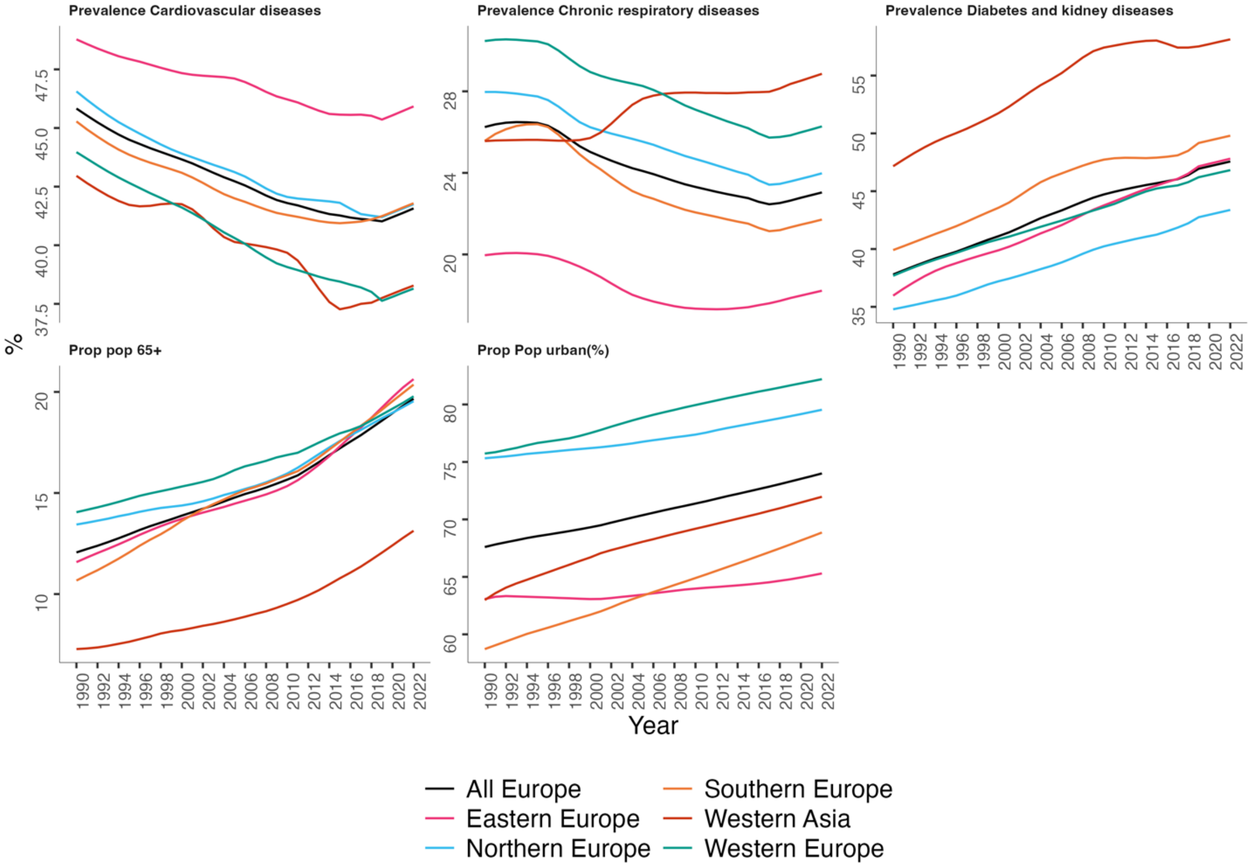

Figure 1.3 Trends cardiovascular diseases prevalence, proportion of population in urban areas and proportion of the population 65 years and above used in computation of heat vulnerability index for the period 1990-2022.

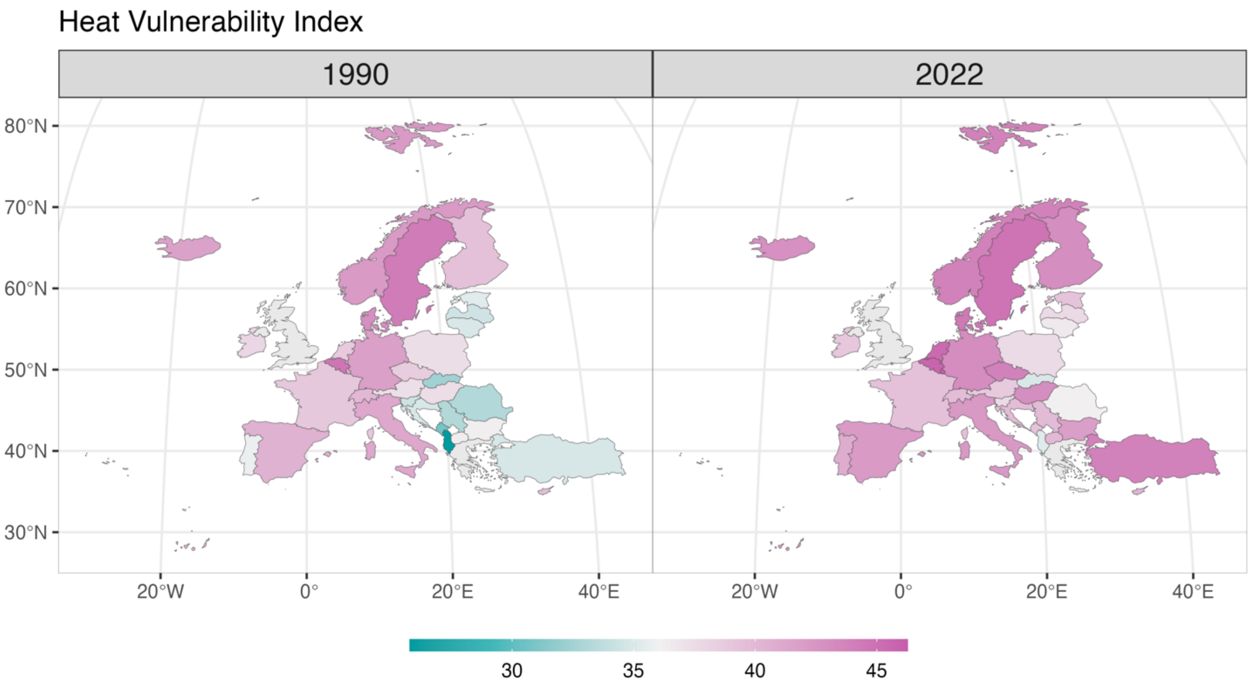

Figure 1.4 Map contrasting the heat vulnerability between 1990 and 2022 for countries in Europe.

Indicator 1.1.2: Exposure of vulnerable populations to heatwaves

Geographic Coverage of Europe

For this indicator we included the European Environment Agency (EEA) member and cooperating countries plus the United Kingdom of Great Britain and Northern Ireland.

Data

1. For climate data, ERA5 Land reanalysis data at 0.1˚ resolution was used. For each grid cell, the number of days of heatwave per year were calculated.
2. To derive vulnerable population counts, spatiotemporal demographic data was derived from NASA SEDAC GPWv4 and Eurostat data. The spatial distribution of demographic age bands for 2010 was obtained from GPWv4. Eurostat demographic census data keyed to NUTS2 regions (2016 definition) was projected onto the grid and used to adjust demographic fractions per grid cell for the years 2000 - 2021 relative to the 2010 baseline. The number of infants was estimated as the difference between total births and infant mortality rate. Missing values demographic, birth, and infant death rate data obtained from Eurostat were filling using the most recent available year preceding year, or the next available year (forward fill first then back fill).

Methods

Heatwaves are defined as periods of 2 or more days where the both the minimum and maximum temperatures are above the 95th percentile of the local climatology (defined on 1986-2005 baseline). The vulnerable population is defined as those above the age of 65 and infants between 0 and 1 years old. Previous research has identified these groups as being particularly vulnerable to heatwave impacts on health. Exposures were calculated by multiplying the number of heatwave days by vulnerable population count, producing an indicator of 'person-days' that reflects both the changes in frequency and duration of heatwaves.[9]

Caveats

In order to estimate the time evolution of demographics, data from diverse sources was combined in order to obtain estimates of both the spatial and temporal characteristics. This has been subject to limited validation.

Future Form of the Indicator

Future versions of the indicator aim to also use Eurostat gridded population data. However, this data is only available for a selection of years, therefore it was decided for this first version to use this hybrid dataset in order to show the time series change and decade changes.

Additional analysis

Figure 1.5 shows the percentage change in heatwave exposure between the decade 2000-2009 and 2010-2019. These increases are broadly distributed across the region with particular hot spots in central and eastern Spain and in central Europe.

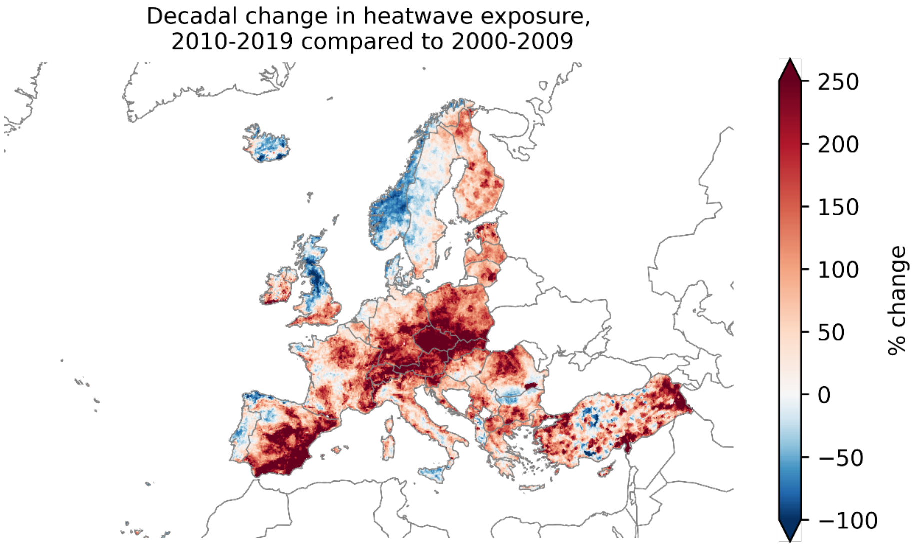

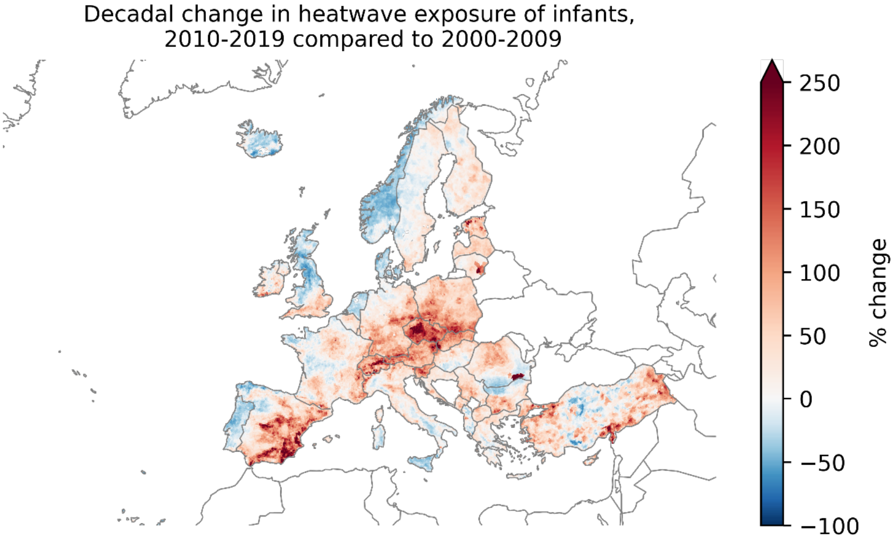

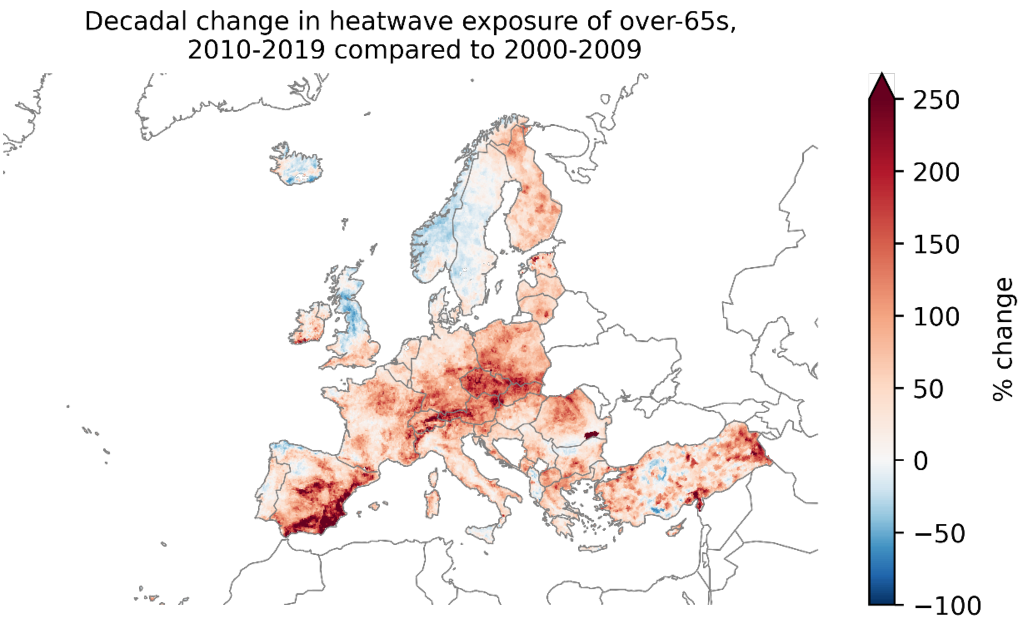

Figure 1.5 Decadal change in heatwave exposures of vulnerable people: (top) over 65 and infants combined, (middle) infants, (bottom) over-65s, comparing the mean exposure by grid cell in 2010-2019 to 2000-2009.

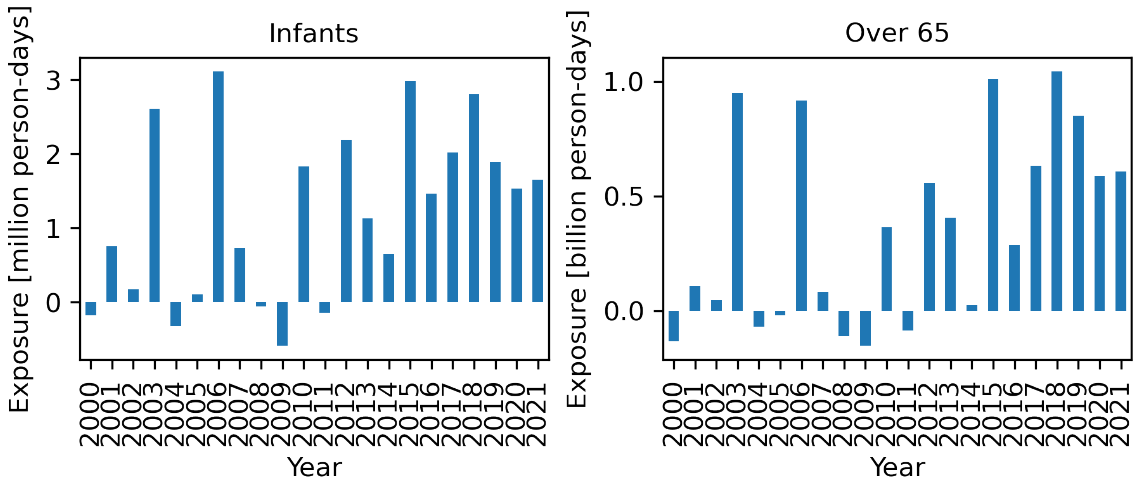

Figure 1.6 Exposure to change in heatwaves (relative to 1986-2005 baseline) for infants and over 65s.

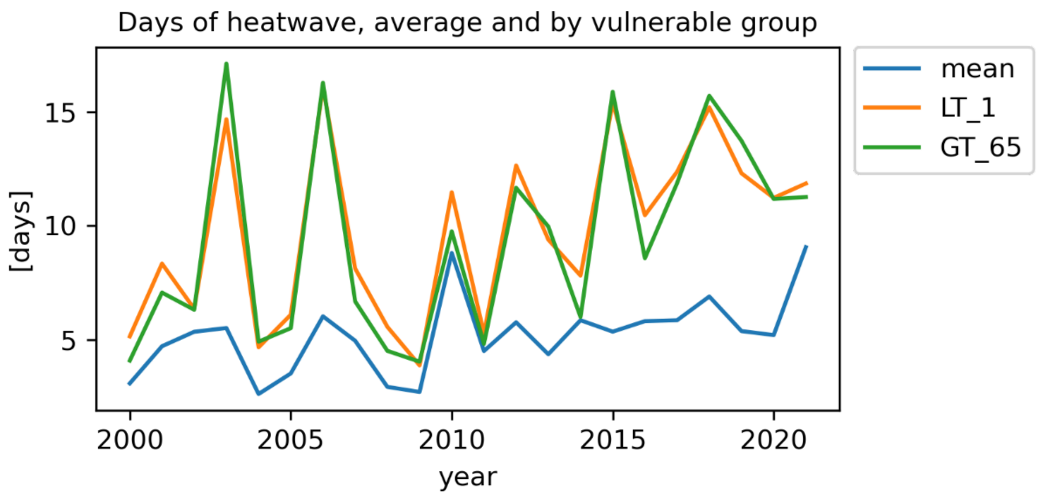

Figure 1.7 Population-change normalised number of heatwave days experienced by infants (LT_1) and over 65s (GT_65), compared to mean of study region.

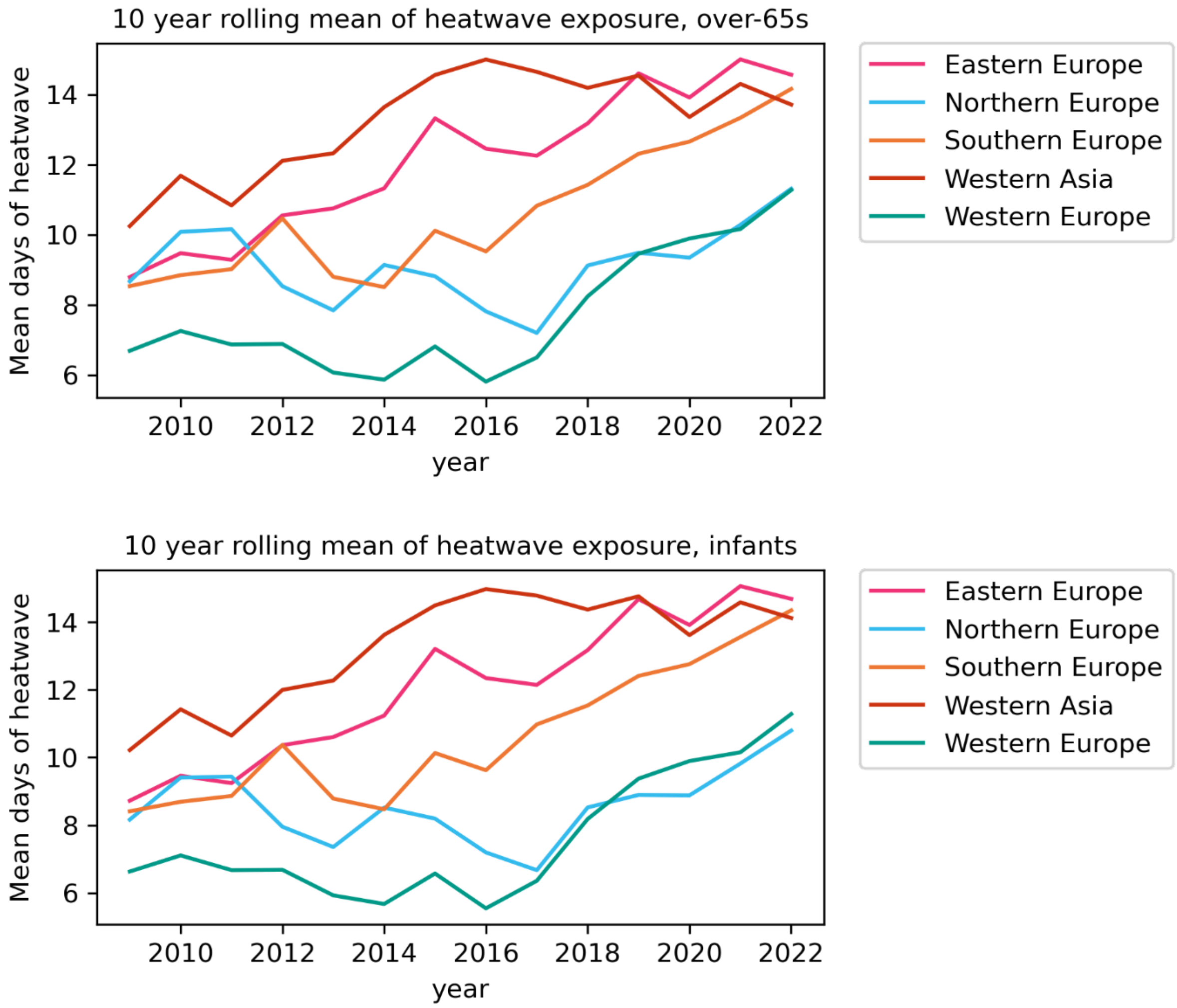

Figure 1.8 10 year rolling averages of vulnerable population normalised heatwave exposure in a) over 65s and b) infants, divided by European region.

Indicator 1.1.3: Physical activity-related heat stress risk

Geographic Coverage of Europe

The indicator covers the following countries, classified using the UN Geoscheme:

- Eastern: Bulgaria, Czechia, Hungary, Poland, Romania, and Slovakia
- Northern: Denmark, Estonia, Finland, Ireland, Iceland, Lithuania, Latvia, Norway, Sweden, and United Kingdom of Great Britain and Northern Ireland
- Southern: Albania, Greece, Spain, Croatia, Italy, Montenegro, North Macedonia, Malta, Portugal, Serbia, and Slovenia
- Western: Austria, Belgium, Switzerland, Germany, France, Liechtenstein, Luxembourg, and Netherlands (Kingdom of the)

Data

1. *Climate/weather data:*
   Variables: hourly 2m temperature; hourly 2m dew point temperature
   Source: ERA5-Land, available for download[10]
   Coverage: 9km grid for Europe from 1990 to 2022.
2. *Population data:*
   Variable: population counts
   Source: GEOSTAT, available for download[11]
   Coverage: 1km grid for the year 2018
   Notes: As for the first iteration of the indicator, the population was fixed to 2018 levels throughout the analysis.
3. *National and regional borders for Europe:*
   Data: National borders, and broad geographic regions (i.e. North, Central, South, West) for Europe
   Source: Eurostat, available for download[12]

Methods

This indicator shows heat stress risk during physical activity, by time of day (intraday patterns) and time of year (intraseasonal patterns), as "risky hours per person per year", based on the Sports Medicine Australia (SMA) Extreme Heat Policy.[13,14] Although developed for use in Australia, the functions underlying the policy were considered to be the most fit-for-purpose of available functions because: no European-specific alternatives exist;[13,15] the policy was recently developed by leading scientists in the field based on the best available empirical evidence; and, the functions rest on human physiology which may reasonably be assumed to be similar across high income countries (personal communication: Professor Ollie Jay, University of Sydney, e-mail, Jan 7 2022). Additionally, the same functions were judged to be the most suitable for global application in the global-level Lancet Countdown.[16]

An "hour at risk" is defined as an hour during which "heat" – represented by a combination of average 2-meter temperature and relative humidity during that hour – renders physical activity of a given intensity "risky". In the

SMA Policy,[13] risk is stratified into four levels (low, moderate, high and extreme) for each of five categories of physical activity, which are grouped according to intensity while accounting for typical clothing and equipment. Risk levels are defined in terms of actions required to avoid heat stress as follows; low risk: maintain hydration and wear light-weight clothing if needed; moderate risk: increase frequency and duration of rest breaks; high risk: use active cooling during rest breaks and/or before and after activity (e.g. drinking cold liquids; water dousing with a sponge or spray bottle); extreme risk: stop activity and seek shelter. The categories of activity include, for example: Category 1 – leisurely walking; Category 2 – brisk walking, athletics, golf; Category 3 – cycling, football, basketball, tennis; Category 4 – trekking, rugby, horse riding; Category 5: mountain biking, field hockey.

For the indicator, risk was quantified as the sum of low, moderate, high, and extreme risks for Category 3 and Category 5 activities; we refer to these as "medium intensity activities" and "strenuous activities", respectively.

The indicator covers the period from 1990 to 2022 but holds population levels constant at 2018 levels over the entire period. Thus, it specifically assesses temporal and spatial changes in risk that are attributable to natural and anthropogenic climate patterns and trends.

Risk functions of the following form, which define the thresholds between given levels of risk for a given category of physical activity, were derived from figures in the SMA guidelines using image processing.[17]

$$\boldsymbol{f(x) = a + bx + cx^2 + dx^3 + ex^4}$$

where $\boldsymbol{x}$ is 2-meter temperature in a given hour, $\boldsymbol{f(x)}$ is 2-meter relative humidity in a given hour, and $\boldsymbol{a, b, c}$ and $\boldsymbol{d}$ are coefficients that are specific to each activity category and risk transition (e.g. moderate to high). The coefficients are specified in Table.

Climate data were obtained for Europe from the ERA5-Land 9km^2 grid for 2-meter temperature and 2-meter dew point temperature for each hour for the years 1990 to 2022.[10] These data were then combined to calculate hourly 2-meter relative humidity.[18] The hourly temperature and relative humidity data were used to drive the risk functions to calculate the level of heat stress risk during each hour in each 9km^2 grid cell.

Population data were obtained from the GEOSTAT 1km^2 population estimates for 2018 (Population was fixed at these levels for all years in the analysis).[11] The total population in each ERA5-Land 9km^2 grid cell was estimated based on the location of the centroids of the GEOSTAT 1km^2 cells (GEOSTAT cells that fell outside the ERA5-Land grid (which were principally on the coast) were assigned the risk level of the closest ERA5-Land grid cell with available climate data based on the Euclidian distance using the latitude and longitude coordinates.).

The calculation of intraday risky hours was performed as follows:

For each 9km^2 grid cell, the annual number of person-hours at risk by time of day (in hours) was calculated by summing the population in the cell for each level of risk, by physical activity category, for each hour of the day during that year.

These estimates were then aggregated to NUTS 2 level and by broad geographic region (i.e. North, Central, West and South Europe).[12,19] For NUTS 2 regions, grid cells were assigned to the region they had the greatest overlap with (this could lead to some misclassification but the implications for our estimates are likely to be minimal),

and the values of each cell falling in each region were summed. NUTS 2 regions were then assigned to broad geographic regions based on their centroids.

Next, risky hours per person per year by time of day was calculated by dividing the person-hours at risk by the population of the area of interest, and, total risky hours per person per year by time of day was calculated by summing risky hours for low, moderate, high, and extreme risk.

Finally, average annual number of risky hours per person by time of day was calculate for three 11-year time slices: 1990-2000, 2001-2011, 2012-2022. Additionally, risky hours were split into those falling into the four hours corresponding to the hottest part of the day in 1990-2000 (11:00 to 14:00 in Central and Northern Europe; 12:00 to 15:00 in Southern and Western Europe), and, all other hours.

For the intraseasonal calculation, the procedure was the same, except that time was divided into months rather than time of day.

Table 1.1 Risk function coefficients by activity type and risk threshold

**Coefficients by physical activity type**	**Risk threshold**		
Medium intensity activity	*Low-Moderate*	*Moderate-High*	*High-Extreme*
a	1123·0830	1054·5207	1240·9576
b	-101·9229	-89·1276	-106·4948
c	3·7725	3·1001	3·7484
d	-0·0657	-0·0509	-0·0614
e	0·0004	0·0003	0·0004
Strenuous activity	*Low-Moderate*	*Moderate-High*	*High-Extreme*
a	1268·3137	1254·2166	846·5127
b	-117·2863	-110·5991	-59·4419
c	4·3161	3·8902	1·6317
d	-0·0733	-0·0626	-0·0194
e	0·0005	0·0004	0·0001

Footnote for Table 1.1: "Risk threshold" refers to the line separating one level of risk from another; for instance, low-moderate refers to the line between low risk and moderate risk. Coefficients are rounded to four decimal places.

Inequality Context

For this indicator, it is not possible to directly assess health inequalities due to a lack of empirically-based knowledge (Here, as is typical in health research, we use "inequality" to refer to unfair, avoidable differences). The analysis is able to assess daily and seasonal patterns of when and where physical activity is risky, and is able to link this to the number of people living in a given location (in 2018). However, while uneven spatial patterns across Europe are discernible, it is not clear that such differences constitute inequalities; within spatial units, the

distribution of risks, and how this may translate into differential impacts, cannot be assessed. Given this, we limit our discussion to general issues which highlight potential areas for future research.

A recent analysis showed two main physical activity-related behavioural responses to heat: activity-depressing effects and activity-delaying effects.[20] Respectively, these refer to not engaging in the activity at all, and, shifting the activity to a cooler part of the day. Activity-depressing effects presume the prior intention to exercise. In Europe, however, an estimated one in three people are classified as physically inactive, with a greater likelihood of inactivity in women compared to men, in people aged 40 to 65 years compared to people aged 18 to 24, and in people not of high socioeconomic status.[21] In terms of differences in confronting the prospect risky hours during physical activity, then, the better-off groups may be expected to encounter greater risk and potentially the greatest reduction in activity. On the other hand, if heat makes it less likely that the inactive decide to become active, the disadvantage of the worst-off may be deepened. Thus, some negative health impacts related to engaging in activity during risky hours or due to reduced activity would be expected across all social groups, but future empirical studies are needed to determine the patterns and associated health inequalities.

Rather than reducing physical activity, some people may delay it to another part of the day. This presumes an awareness of the risk as well as a degree of flexibility. In very general terms, the health of people of higher socioeconomic status may tend to be better than those of lower status partly because they have access to greater array of health-protecting resources, including knowledge, power, money and social connections.[22] It seems reasonable to assume that these broadly useful resources may be harnessed by the best-off to better confer both risk awareness and options to defer activity. If such mechanisms were found to be operating, the resulting differences in outcomes would likely be inequalities. An additional complication, however, is that avoiding heat by delaying activity until the evening has the potential to affect sleep quality, which in turn may be linked to diabetes, obesity and cardiovascular disease.[20] That is, it may be that the better-off maintain physical activity only by opening new streams of risk.

In sum, the net-balance of negative health impacts by social group is likely to arise from a broad set of relations involving differences in heat exposure, propensity to exercise, risk awareness, access to resources (material, social, cultural), and knock-on effects. While on balance it is seems reasonable to assume that rising physical activity-related heat stress risk will make the worst-off worse off (at least relatively; probably absolutely), more empirical evidence is required before attempting to quantify these in this indicator.

Caveats

The first caveat is that the exposure-risk functions were originally developed for use in Australia. They were however considered to be the best applicable option. See the first paragraph of "Methods" for further detail. We note that, given European populations – particularly those in Northern region – may be less accustomed to high temperatures than the Australian population, our estimates may tend to be conservative.

A second caveat is that the figures from which the risk functions were derived only consider temperatures in the range 26°C to 44°C.[13] Thus it was necessary to extrapolate them based on the following justifications. For temperatures above 44°C, the underlying figures show that almost all hours (an exception being for medium

intensity activities when relative humidity is close to 0%; the latter is unlikely) with high temperatures would be classified as extreme risk for both of categories of physical activity considered in the indicator; thus, extrapolation at high temperatures is straightforward. For temperatures below 26°C, the figures show most hours would be low risk. However, at high levels of relative humidity, and depending on the intensity of exercise, risk may be moderate or high, or - very rarely - extreme. This means extrapolation is less certain at the low end of temperatures. In this regard, it was suggested by an author of the original guideline (personal communication: Professor Ollie Jay, University of Sydney, e-mail, Jan 10, 2022) that below 26°C risk would generally (except at very high levels of exertion) be expected to be quite low irrespective of relative humidity as dry heat loss becomes increasingly pronounced and is less dependent on sweat evaporation. Given this, it was suggested it would be reasonable to extrapolate the functions down to around 23°C and then assume that risk is "low" beyond this. Our extrapolated functions adhere to this recommendation.

A third caveat is that population is fixed at 2018 levels when calculating the indicator for all years. This was partly a pragmatically-based decision given the available data and resources, and 2018 was chosen as it offered the greatest spatial coverage of available options. The implication is that the indicator specifically assesses how risk has changed due to warming since 1990. This will have some influence on the spatial pattern of risk trends due to differences in population growth rates across regions, including those due to urbanization. Additionally, because of population growth since 1990, the magnitude of the change in person-hours of risk over time will tend to be lower than it would be if a moving population were used.

A fourth caveat is that the analysis by hour of the day is done using UTC (Coordinated Universal Time) for all countries. While this does not affect the total risky hours it has some influence on their intradaily distribution. However, for the analysis across Europe, this is likely to have only a small effect on the results, and the hottest parts of the day were determined empirically for UTC.

Future Form of the Indicator

Three main aspects of development should be considered. Firstly, the introduction of a moving population so as to allow the assessment of how population changes are influencing risk patterns. Secondly, the incorporation of applicable new research that allows risk stratification by population group (e.g. age, gender). Thirdly, if the latter is possible, assessing which between-group difference represent inequalities in risk and expected impacts.

Additional analysis

The results in the main text are complemented by, firstly, by some additional numbers from the figure in the main text; secondly, the same intraday analysis but for strenuous (Category 5) activities; and, thirdly, analyses of intraseasonal patterns for medium intensity and strenuous activities.

For intraday patterns of risky hours per person per year for medium intensity activity – as shown in the main figure – the absolute change between 1990-2000 and 2002-2012 in the number of hours in the hottest part of the day, in Eastern, Northern, Southern, and Western Europe was, respectively: 31 to 58; 1 to 4; 91 to 141; and, 18 to

32. The corresponding changes for hours outside the hottest part of the day were, respectively: 36 to 74; 1 to 4; 129 to 255; and 20 to 39.

Figure 1.9 shows the evolving intraday time pattern of risky hours for strenuous activities for three 11-year time slices covering 1990-2000, 2001-2011, and 2012-2022. Comparing 2012-2022 to 1990-2000, the proportion of risky hours per person falling inside the hottest part of the day rose relatively by 72%, 168%, 51%, and 59%, in Eastern, Northern, Southern, and Western Europe, respectively (The corresponding absolute rises were: 51 to 88; 3 to 8; 141 to 196, and 32 to 51). For risky hours outside the hottest part of the day, the relatively changes were, respectively: 94%, 239%, 94%, and 81% (with corresponding absolute changes of: 65 to 126; 2 to 8; 222 to 385; and, 37 to 67). In 2022, the total risky hours per person in Eastern, Northern, Southern, and Western Europe were 208, 23, 769, and 137, respectively, of which 56%, 52%, 68%, and 58% occurred outside the hottest part of the of the day.

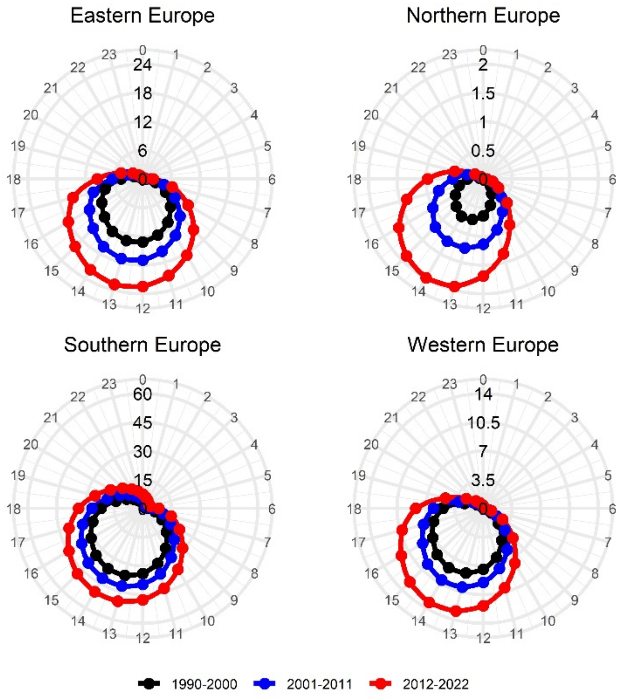

Figure 1.9 Mean annual risky hours per person, for physical activity-related heat stress, for strenuous activities, by time of day for three 11 year time slices, by European region. In each plot, the outer circle shows time of day (UTC) using a 24 hour clock; the inner circles show number of risky hours (note that the scale differs in each plot). (Footnote: The hottest part of the day is 11:00-14:00 for Eastern Europe and 12:00-15:00 in Northern, Southern and Western Europe.)

Figure 1.10 and **Figure 1.11** show the evolving intraseasonal pattern of risky hours for medium intensity and strenuous activities, respectively, for the same three 11 year time slices as above. In both figures, it is evident that risky hours are expanding into a wider range of months. **Table 1.2** shows this as the proportion of all risky hours that fall in a given month, in 1990-2000 and 2002-2022.

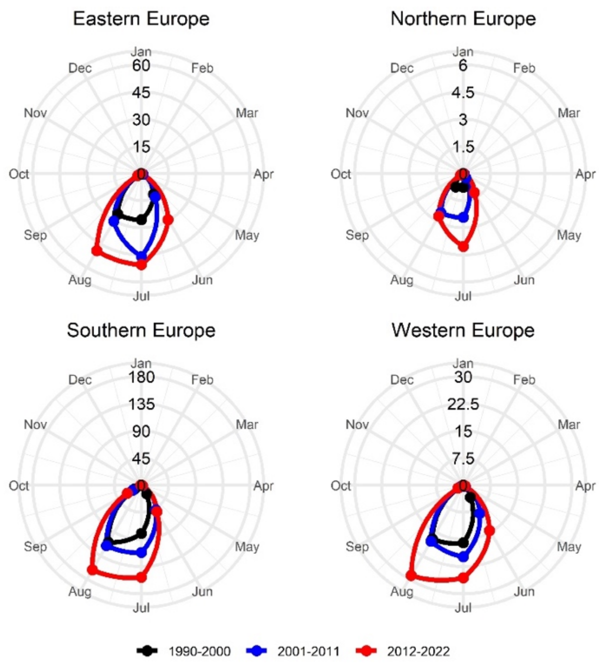

Figure 1.10 Mean annual risky hours per person for physical activity-related heat stress risk during moderate intensity activity, by month of year for three 11-year time slices, by European region. In each plot, the outer circle shows the month; the inner circles show number of risky hours (note that the scale differs in each plot).

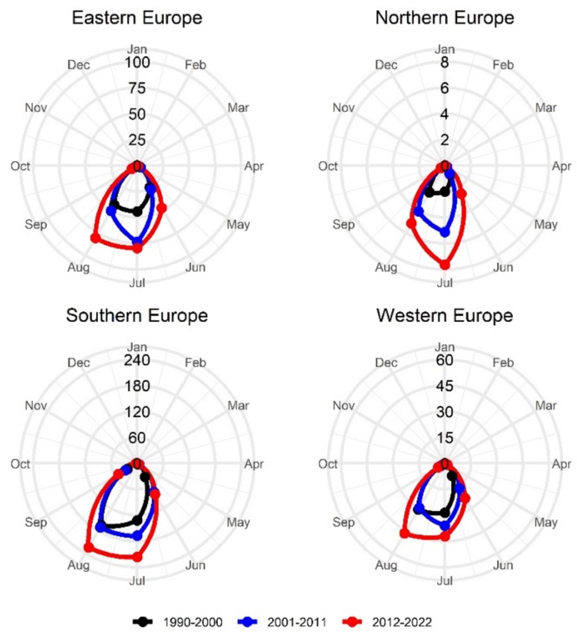

Figure 1.11 Mean annual risky hours per person for physical activity-related heat stress during strenuous activity, by month of year for three 11 year time slices, by European region. In each plot, the outer circle shows the month; the inner circles show number of risky hours (note that the scale differs in each plot).

Table 1.2 Proportion of total risky hours per person per year falling in a given month in 1990-2000 and 2012-2022, for medium intensity and strenuous activities.

**Eastern Europe**				
**Month**	**Medium intensity (Category 3)**		**Strenuous (Category 5)**	
	**1990-2000**	**2012-2022**	**1990-2000**	**2012-2022**
March	0.0%	0.0%	0.0%	0.0%
April	0.0%	0.0%	0.0%	0.0%
May	0.9%	0.1%	1.1%	0.4%
June	19.9%	22.4%	20.9%	22.0%
July	38.7%	38.2%	38.0%	37.2%
August	39.2%	37.4%	37.9%	37.6%
September	1.2%	1.8%	1.9%	2.7%
October	0.2%	0.0%	0.2%	0.0%
November	0.0%	0.0%	0.0%	0.0%
**Northern Europe**				
**Month**	**Medium intensity (Category 3)**		**Strenuous (Category 5)**	
	**1990-2000**	**2012-2022**	**1990-2000**	**2012-2022**
March	0.0%	0.0%	0.0%	0.0%
April	0.0%	0.0%	0.0%	0.0%
May	0.0%	0.0%	0.2%	0.1%
June	15.1%	14.9%	15.1%	16.0%
July	40.2%	49.5%	38.3%	48.7%
August	44.7%	33.4%	46.0%	32.8%
September	0.0%	2.1%	0.3%	2.4%
October	0.0%	0.0%	0.0%	0.0%
November	0.0%	0.0%	0.0%	0.0%
**Southern Europe**				
**Month**	**Medium intensity (Category 3)**		**Strenuous (Category 5)**	
	**1990-2000**	**2012-2022**	**1990-2000**	**2012-2022**
March	0.0%	0.0%	0.0%	0.0%
April	0.0%	0.0%	0.0%	0.0%
May	0.3%	0.4%	0.5%	0.8%
June	7.7%	12.9%	9.9%	14.2%
July	36.3%	38.5%	36.3%	37.3%
August	49.9%	41.0%	45.7%	38.6%
September	5.6%	6.9%	7.2%	8.6%
October	0.2%	0.3%	0.4%	0.5%
November	0.0%	0.0%	0.0%	0.0%
**Western Europe**				
**Month**	**Medium intensity (Category 3)**		**Strenuous (Category 5)**	
	**1990-2000**	**2002-2022**	**1990-2000**	**2002-2022**
March	0.0%	0.0%	0.0%	0.0%
April	0.0%	0.0%	0.0%	0.0%
May	0.1%	0.7%	0.6%	1.4%
June	10.5%	20.4%	12.0%	19.7%
July	42.8%	36.0%	41.4%	35.7%
August	46.0%	40.5%	44.6%	39.5%
September	0.6%	2.4%	1.4%	3.8%
October	0.0%	0.0%	0.0%	0.0%
November	0.0%	0.0%	0.0%	0.0%

Indicator 1.1.4: Heat-related mortality

Part A (first part of the indicator)

Geographic Coverage of Europe

The indicator included 823 contiguous regions representing over 543 million Europeans in 35 countries, namely Albania (12 regions), Austria (35), Belgium (44), Bulgaria (28), Switzerland (26), Cyprus (1), Czechia (14), Germany (16), Denmark (11), Estonia (5), Greece (52), Spain (59), Finland (19), France (96), Croatia (1), Hungary (20), Ireland (1), Iceland (2), Italy (103), Liechtenstein (1), Lithuania (10), Luxembourg (1), Latvia (6), Montenegro (1), Malta (1), the Netherlands (Kingdom of the) (40), Norway (11), Poland (73), Portugal (25), Romania (42), Serbia (25), Sweden (21), Slovenia (1), Slovakia (8) and the United Kingdom of Great Britain and Northern Ireland (12).

Data

1. *Mortality data*

We obtained weekly counts of all-cause mortality by sex and age groups from Eurostat.[23] Missing data in the dataset was complemented by contacting the corresponding National agencies for statistics. The final dataset included 45,184,044 counts of death (22,000,519 for women and 21,913,050 for men) between January 2015 and November 2022 from 823 contiguous regions in 35 European countries.

2. *Temperature data*

We transformed the hourly gridded 2-meter temperature data from the high-resolution ERA5-Land reanalysis (ECMWF 2022),[24] freely available in near real time at a 9-kilometre grid resolution, into weekly regional averages of daily mean 2-meter temperature.

3. *Population data*

Regional population estimates were obtained from Eurostat.[25] Population estimates were used to compute heat related mortality rates.

Methods

The statistical analysis was stratified by sex. First, a quasi-Poisson regression model was used to characterise the temperature-mortality relationship in each European region. Specifically, the model equation included a natural cubic spline of time with 8 degrees of freedom per year to control for the seasonal and long-term trends, and a cross-basis function from a Distributed Lag Non-Linear Model to estimate the exposure-lag-response association between weekly temperatures and mortality counts.[26] The exposure-response function of the cross-basis was modelled with a natural cubic spline with three internal knots at the 10th, 50th and 90th percentiles of local weekly temperature distribution,[27] and the lag-response function with integer lag values of 0, 1, 2 and 3 weeks.[28]

In the second stage, we used a multivariate multilevel meta-regression analysis[29] to pool the location-specific coefficients obtained in the first step. The meta-regression included (i) country random effects and the location-specific (ii) temperature average, and (iii) temperature interquartile range as meta-predictors. We derived the best linear unbiased predictions of the temperature-mortality relationship in each region from the meta-regression, which were then used to calculate the number of deaths attributable to heat.[30] Heat weeks were defined as those with average temperatures above the local minimum mortality temperature. Heat attributable mortality rate (per 100 000 inhabitants) was computed separately for the periods 2003-2012 and 2013-2022, and given that mortality counts were not available in some countries for some of these years (see section *Data* above), the attributable mortality was estimated by using the mean annual cycle of mortality counts in each region.[31] Finally, we computed the difference in heat attributable mortality rate between 2013-2022 and 2003-2012.

The code used to construct this indicator and create the visualizations included in this publication is available at the following repository: https://github.com/BallesterJoan/europe_summer_2022_heat.

Inequality Context

There are multiple individual-level factors that increase vulnerability to heat, such advanced age and pre-existing co-morbidities (i.e., cardiovascular and respiratory diseases),[32] sex (being women),[33,34] social isolation[35] or low educational level,[34,36] among many others. However, because of data limitations, this indicator was only stratified by sex.

On the other hand, given that the indicator is available for sub-national regions, it highlights both between-country and within-country inequalities in the impact of heat on mortality. These spatial inequalities respond to differences in community-level factors that have not, however, been analysed in this indicator.

Caveats

The standard approach in timeseries studies of the health impacts of ambient temperatures is to calculate epidemiological models between daily temperatures and counts of death.[27] A recent study used daily mortality data in 147 contiguous European regions to compare temperature attributable mortality estimates obtained from daily and weekly data models.[28] Results showed that annual heat attributable mortality incidences are systematically underestimated in the weekly data model. These biases are however constant over time, and therefore, the difference in heat-attributable mortality rate between 2013-2022 and 2003-2012 from the weekly data model is not biased. The study also uses relatively large areas in some of the analysed countries which may lead to exposure misclassification.

Future Form of the Indicator

EARLY-ADAPT (Feb'2021 - Jan'2026)[37] project is currently expanding (i.e. more countries and smaller regions) and updating (retrospectively and forward in time) the database of 147 contiguous European regions used in Martínez-Solanas and colleagues.[27] In future years, estimates from daily and weekly data models could be calibrated to bias-correct any estimate (i.e. any year, region and country) obtained from weekly data models

Findings & Additional Analysis

In order to validate that the spatial distribution of the indicator was not sensitive to the population ageing, we introduced in the meta-regression (see Methods above) the percentage of people aged 80+ as a new meta-predictor and recalculated the indicator. This sensitivity analysis showed that the spatial distribution of the heat attributable mortality rate does not depend on the population ageing (see **Figure 1.13**).

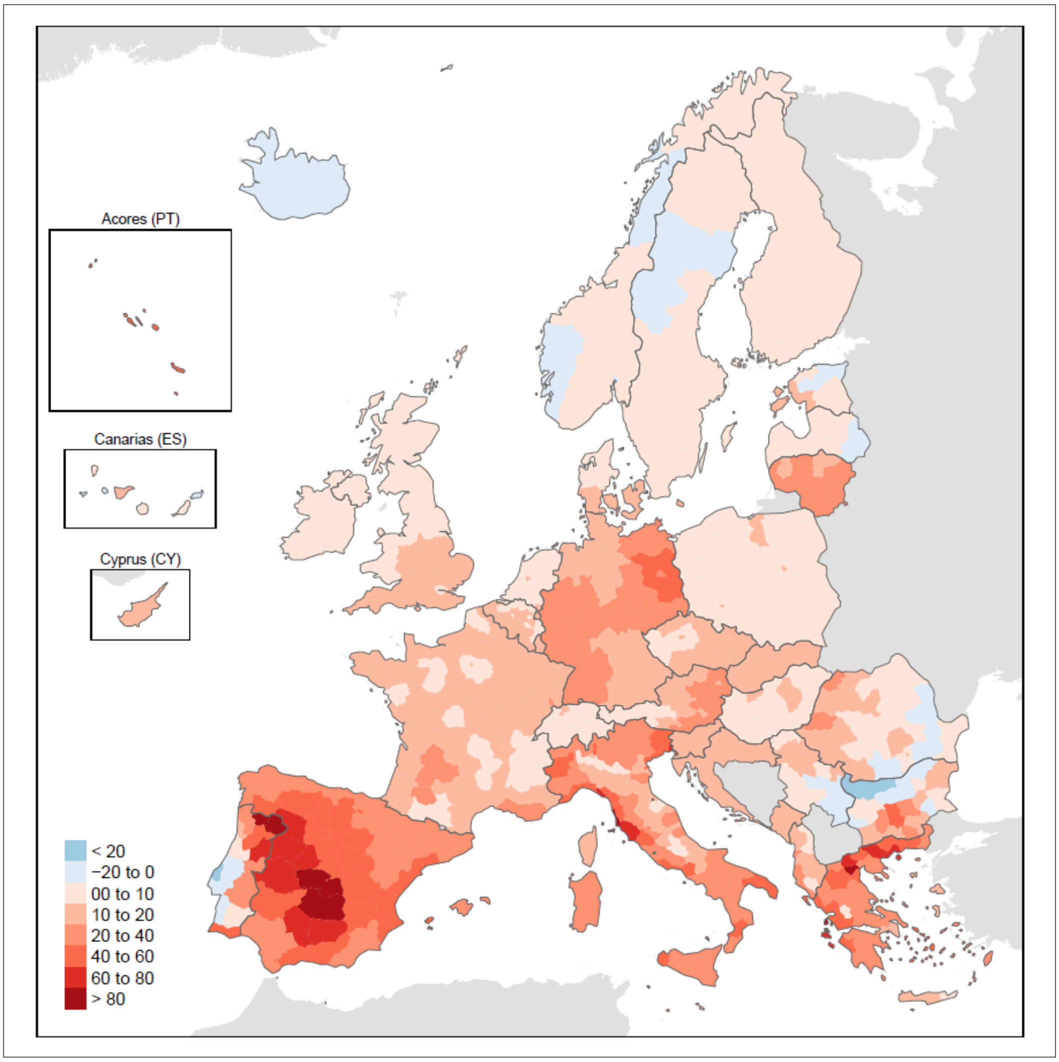

Figure 1.12 Change in heat attributable mortality rate between 2003-2012 and 2013-2022 for the general population. The mortality rate is expressed as the number of deaths per 100 000 inhabitants.

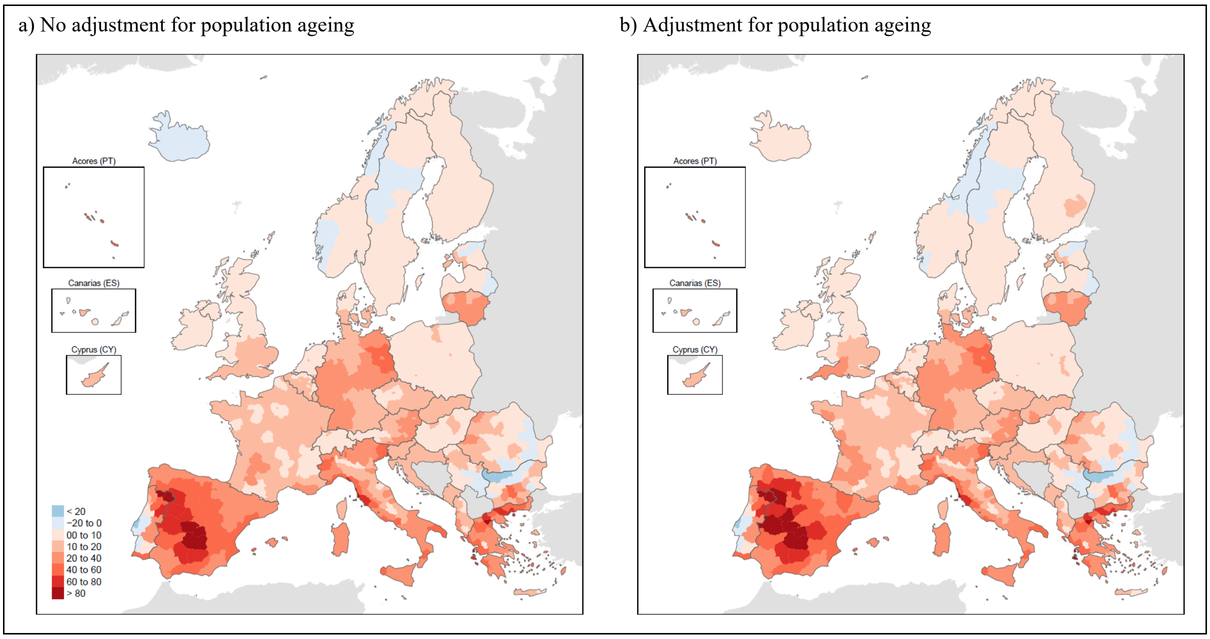

Figure 1.13 Heat attributable mortality rate before and after adjusting for population ageing

The mortality rate is expressed as the number of deaths per 100 000 inhabitants.

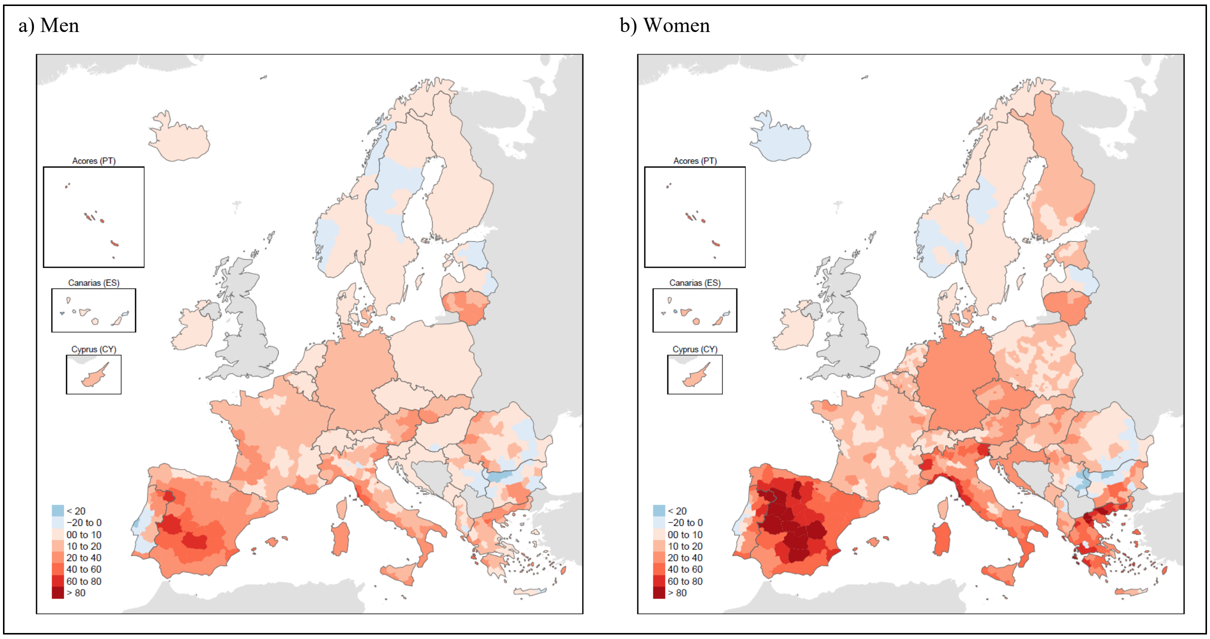

Figure 1.14 Heat attributable mortality rate by sex. The mortality rate is expressed as the number of deaths per 100 000 inhabitants.

Indicator 1.1.4: Heat-related mortality

Part B (second part of the indicator)

Geographic Coverage of Europe

For this indicator, we included the European Environment Agency (EEA) member and cooperating countries plus the United Kingdom of Great Britain and Northern Ireland, excluding Türkiye, Bosnia and Herzegovina, Kosovo (under UNSC resolution 1244) and North Macedonia as data was not available for these countries.

Data

We obtained weekly counts of all-cause mortality by sex from Eurostat.[38] Missing data was complemented by contacting the corresponding national agencies for statistics. The final dataset included 45,184,044 counts of death (22,000,519 for women and 21,913,050 for men) between January 2015 and November 2022 from 823 contiguous regions representing over 543 million Europeans in 35 countries, namely Albania (12 regions), Austria (35), Belgium (44), Bulgaria (28), Switzerland (26), Cyprus (1), Czechia (14), Germany (16), Denmark (11), Estonia (5), Greece (52), Spain (59), Finland (19), France (96), Croatia (1), Hungary (20), Ireland (1), Iceland (2), Italy (103), Liechtenstein (1), Lithuania (10), Luxembourg (1), Latvia (6), Montenegro (1), Malta (1), the Netherlands (Kingdom of the) (40), Norway (11), Poland (73), Portugal (25), Romania (42), Serbia (25), Sweden (21), Slovenia (1), Slovakia (8) and the United Kingdom of Great Britain and Northern Ireland (12). On average, each region represented a population of 660,000 Europeans. Data by sex was not available in the United Kingdom of Great Britain and Northern Ireland, and only at the country level in Germany.

Temperature data

We transformed the hourly gridded 2-meter temperature data from the high-resolution ERA5-Land reanalysis[10] into weekly regional averages of daily mean 2-meter temperature. We obtained annual values of global mean surface temperature relative to 1950-1980 from NOAA.[39]

Methods

Epidemiological models

The calculation of the temperature-related mortality was done in two steps, following the methodology used elsewhere.[40] In the first stage, we used quasi-Poisson regression models, which allow for overdispersed counts of deaths, to calculate the location-specific temperature-lag-mortality relation in each European region. The models included (i) an intercept, (ii) a natural cubic spline of time with 8 degrees of freedom per year to control for the seasonal and long-term trends, and (iii) a cross-basis function to estimate the exposure-lag-response association between weekly temperatures (*temp*) and mortality counts (*mort*) in the period 2015-2019:

$$log(\ E(mort)\ ) = intercept + ns(\ time,\ 8df\ per\ year\ ) + crossbasis(\ temp;\ 0,1,2,3\ weeks\ )$$

The lag-response function of the cross-basis was modelled with integer lag values of 0, 1, 2, and 3 weeks, and the exposure-response function with a natural cubic spline with three internal knots at the 10th, 50th, and 90th percentiles of the location-specific weekly temperature distribution.

In the second stage, we used a multivariate multilevel meta-regression analysis to pool the location-specific coefficients obtained in the first step.[29] The meta-regression included (i) country random effects and the location-specific (ii) temperature average, (iii) temperature inter-quartile range, and (iv) percentage of people aged 80+ years as meta-predictors. We derived the best linear unbiased predictions of the temperature-mortality relationship in each region from the meta-regression[41] to obtain the location-specific minimum mortality temperature, and to transform the regional temperature and mortality time-series into the weekly heat-related mortality numbers over the years 1981-2022.[30] Given that mortality counts were not available for most of these years, the heat-related mortality was estimated by using the mean annual cycle of regional mortality time-series.[31] Heat-related mortality was calculated for the weeks with average temperatures above the location-specific minimum mortality temperature. To perform the extreme event attribution analysis (see the section *Extreme event attribution analysis* below), regional heat-related mortality numbers were aggregated to 232 larger regions (generally from NUTS3 to NUTS2, but to NUTS0 in Iceland, Norway and Sweden; see the section *Caveats* below). Similarly, we computed 1000 Monte Carlo simulations of the regional heat-related mortality numbers, and separately aggregated the numbers in each simulation.

Extreme event attribution analysis

After estimating the regional heat-related mortality numbers, the indicator applied the extreme event attribution framework[42] to calculate the increase in the likelihood of the annual maxima of these weekly time-series in recent factual periods compared with a counterfactual pre-industrial climate. Towards this aim, we fitted a non-stationary Generalized Extreme Value (GEV) distribution to the annual maxima of the weekly regional time series, referred to as "*block maximum approach*" in extreme value theory. For each region, we obtained a GEV distribution with time-varying location parameter, and calculated the probability ratio for each year by shifting the GEV distribution with the annual value of the smoothed Global Mean Surface Temperature (GMST). For any given recent factual period (here, 1981-2000 and 2003-2022), we calculated the median of the probability ratios over the years of the period. We applied a bootstrapping routine generating 1000 GEV parameters (location, location trend, shape, and scale) to obtain the confidence intervals of the GEV distributions, and extracted the fraction of probability ratios greater than one to calculate the significance level of the results.

The code used to construct this indicator and create the visualizations included in this publication is available at the following repository: https://github.com/tmb-esr/LCE24_attribution_mortality.

Inequality Context

There are multiple factors of increased vulnerability to heat,[40] such as the elderly with pre-existing cardiovascular and respiratory diseases,[42] women,[33,34] and socially isolated,[43] or less educated individuals,[34,36] among many others

Caveats

We used weekly temperature and mortality data in the epidemiological models, which is expected to underestimate the day-to-day variability of the time-series, and possibly, their lagged short-term associations. We also expect that the use of daily data would improve the fitting of the GEV distribution, given that the annual maxima are more extreme when they are calculated from daily time-series. The choice of weekly data was however motivated by the need to use format-homogeneous regional counts of death covering almost all the European countries over a common period of time.

Another limitation of the indicator is the relatively short period of temperature data considered for the fitting of the GEV distribution, i.e. 1981-2022. The indicator might therefore be sensitive to the (non-)occurrence of unprecedented, high-impact, low-probability events. We restricted the period to the last 42 years and aggregated the heat-related mortality numbers to the NUTS2 level to improve the fitting of the GEV distribution. In general, the GEV fitting was found to be more challenging when applied to annual heat-related mortality maxima than for annual temperature maxima, because of (i) the non-linear relationship between temperatures and mortality numbers, and (ii) the existence of a cut-off point at the minimum mortality temperature, below which heat-related mortality numbers are by definition equal to zero.

Future Form of the Indicator

The indicator here proposed for Europe can be generalised (i) globally, (ii) to less or non-extreme events (e.g. summer heat-related mortality), (iii) to any health outcome (e.g. morbidity), and (iv) to any climate variable or compound event. The framework here proposed can thus be calculated from a climate-only perspective for any climate variable (e.g. highest precipitation episode), compound event (e.g. hot-humid), or climate impact (e.g. hospital admissions). Moreover, the concept "*episode*" can be easily adapted to represent the most extreme day, week, month or season of the year.

The Lancet Countdown initiative encourages the use of open-access data, which motivated the use of the weekly mortality database from Eurostat. Beyond this general framework, it is possible to use restricted-access data from ongoing international initiatives, such as EARLY-ADAPT,[44] which is currently creating a European database for regions and cities with daily mortality, morbidity and occupational records spanning over several decades. The use of this daily database would (i) improve the fitting of the GEV distribution, (ii) generalise the framework to other health outcomes, and (iii) allow to include the role of adaptation in the analyses.

Additional analysis

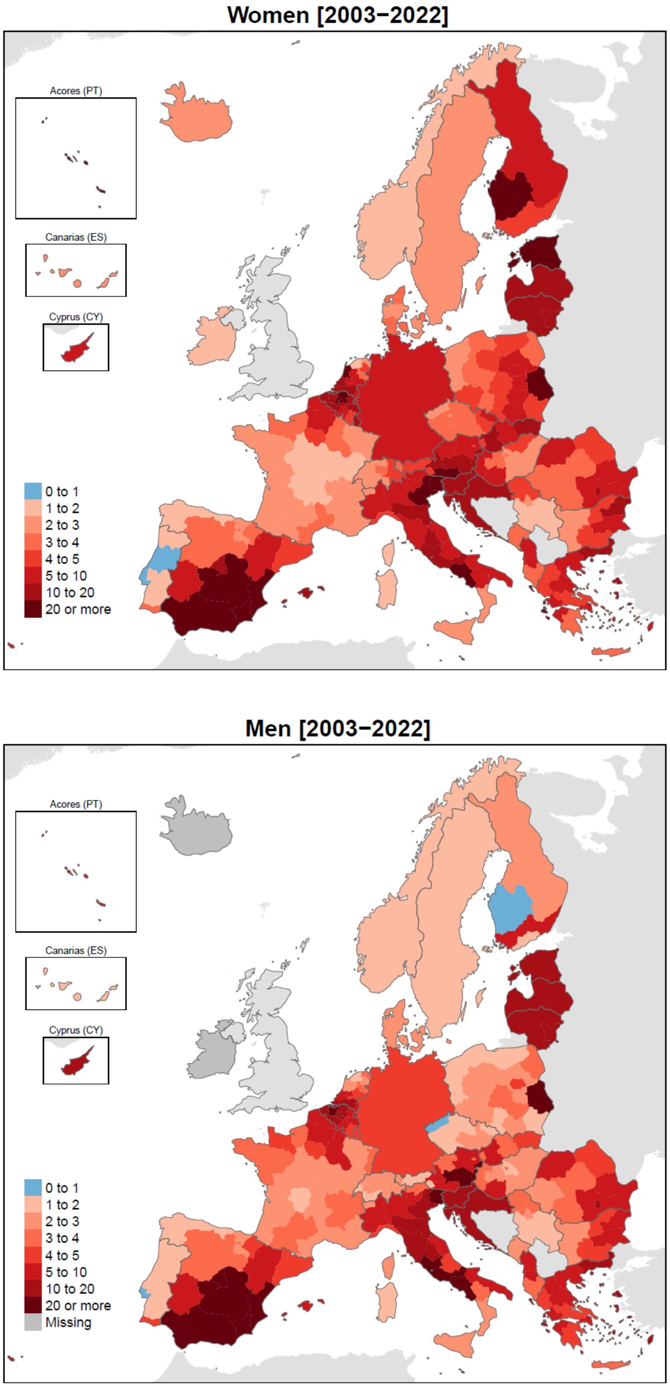

Figure 1.15. Same as Figure in main text, but for women (up) and men (down).

1.2: Extreme events and Health

Indicator 1.2.1: f smoke

Geographic coverage of Europe

For this indicator, European Environment Agency (EEA) member and cooperating countries plus the United Kingdom of Great Britain and Northern Ireland were included subject to exposure, population, and mortality data availability (see below). We grouped the included countries in four European regions (Western/Southern/Northern/Eastern) according to the UN Geoscheme classification except for Cyprus, which was moved to the "Southern Europe" group since it was the only country we analysed which was considered to be in the "Western Asia" group in the UN classification.

Data

The input data for this indicator is as follows:

1. GEOSTAT population grids for Europe in polygon format. The spatial resolution of the dataset is 1km, and the available years are 2006, 2011, 2018.
2. Eurostat GISCO NUTS boundaries for Europe at a scale 1:1M. The 2021 edition was used.
3. Eurostat regional weekly total death counts for the study period 2003-2022 at the NUTS 2 level for all countries except for Croatia, Germany, Ireland, and Slovenia, whose mortality data was only available at the NUTS 1 level.
4. Daily $PM_{2.5}$ – all-cause mortality exposure-response linear function (RR: 1.0065; 95% CI: 1.0044–1.0086 for a 10µg/m^3 $PM_{2.5}$ increase) extracted from a meta-analysis by Orellano *et al.* 2020.[45]
5. Finnish Meteorological Institute fire smoke dispersion forecasts ($PM_{2.5}$ concentrations) derived from the Integrated System for wild-land Fires (IS4FIRES) and the System for Integrated modelling of Atmospheric composition (SILAM) models. The spatial resolution of the product is 0.1° (~10km) and the temporal resolution is 1h. Daily averages were used in the production of the indicator. A complete description of the fire system can be found in Sofiev *et al.* 2009,[46] Soares *et al.* 2015,[47] and Hänninen *et al.* 2022.[48] Briefly, the system is based on MODIS fire radiative power products MOD14/MYD14, which are available globally with 1-9 km^2 spatial resolution depending on the particular overpass and the instrument viewing angle. The released radiative energy is converted to smoke emissions using empirical emission factors. Emissions are then used by the SILAM atmospheric composition model for computing plume dispersion, chemical transformations, and deposition. Outputs from this model have been previously used to estimate the health burden of wildfire smoke in Europe (Kollanus et al., 2017).[49]
6. Canadian Forest fire Weather Index (FWI) computed from ERA5 climate reanalysis data for the period 1980-2022 with a 0.5° spatial resolution. Briefly, FWI is a unitless fire danger index that estimates potential fire intensity based on fuel availability (i.e. drought and moisture conditions) and fire spread (i.e. wind conditions).

Methods

Two sub-indicators were constructed: 1) annual average wildfire-$PM_{2.5}$ exposure and wildfire risk (exposure sub-indicator) and 2) yearly attributable mortality to wildfire-$PM_{2.5}$ exposure (health impacts sub-indicator). Each of these indicators was calculated at the NUTS2, country, European regions; for areas that met the following criteria:

- Exposure sub-indicators: To be included in the NUTS 2021 classification, to have gridded population data for at least a subset of the study period, and to have exposure data available. Following these criteria, overseas territories, Atlantic Islands (Canary Islands, Madeira, Azores), and several European countries/regions (Bosnia and Herzegovina, Kosovo [under UNSC resolution] 1244), Türkiye) were excluded from the sub-indicator.
- Health impacts sub-indicator: The same criteria for the exposure indicators applied, with the additional requirement of having weekly mortality time series data for at least a subset of the study period. Following this criterium, North Macedonia was further excluded from the sub-indicator.

The workflow to construct the indicators was as follows:

1. GEOSTAT gridded population data (2006, 2011, 2018) in polygon format (1km spatial resolution) were transformed into a regular raster matching the SILAM grid (0.1° spatial resolution). Aggregation was performed via addition of GEOSTAT population counts whose polygon centroids fell within the larger SILAM wildfire-$PM_{2.5}$ cell. No population estimates were available for a set of countries (Albania, Croatia, Cyprus, Montenegro, North Macedonia and Serbia) at the 2006 edition, which were imputed with data from 2011. Population data for the rest of the years was obtained via linear interpolation.
2. NUTS2 codes were assigned to each of the SILAM wildfire-$PM_{2.5}$ grid cells with a spatial join via intersection. If a given cell intersected with more than one NUTS2 unit, the one with the largest overlap was taken. Country codes and regional indicators were derived from the NUTS2 codes.
3. Daily wildfire-$PM_{2.5}$ from SILAM, NUTS2 codes and population counts spatially-aligned grids were stacked and transformed into tabular format for further analysis.
4. EUROSTAT weekly mortality data were disaggregated into daily counts (i.e., the temporal resolution of the exposure and the exposure-response function) by assigning an equal number of deaths to each day of the week, i.e. by dividing the weekly mortality counts by 7.
5. Daily population-weighted wildfire-$PM_{2.5}$ at NUTS2 level was calculated by first computing population weights adding up to 1 within each NUTS2 unit, and then computing a weighted average of the exposures. Daily NUTS2 population-weighted $PM_{2.5}$ exposure datasets were aggregated to years via averaging. Yearly averages of population-weighted $PM_{2.5}$ at the country, regional, and European-wide level were computed using the same approach.
6. To compute yearly attributable mortality, the following steps were taken following a comparative Health Impact Assessment (HIA) framework:
   a) The point estimate Relative Risk (RR) (RR: 1.0065 per 10μg/m³ $PM_{2.5}$ increase) was scaled to the exposure in each daily wildfire-$PM_{2.5}$ cell by applying the following transformation where *i, j* and *t* indices refer to the grid cell, NUTS and day identifiers, respectively; and $PM_{2.5}$ refers to the actual wildfire-$PM_{2.5}$ for a grid cell-day:

$$\mathrm{RR_{ijt}} = \exp\left(\ln(1.0065) * \frac{\mathrm{PM2.5}_{ijt}}{10}\right)$$

b) Daily Population Attributable Fractions (PAF) were computed at the NUTS 2 level using standard HIA methods[50] for a null counterfactual exposure of 0μg/m³ and the scaled RR from the previous step. Population weights p_{ijt} were computed as per step 5:

$$\mathrm{PAF_{jt}} = \frac{\sum_{i=1}^{n} \mathrm{p_{ijt}RR_{ijt}} - 1}{\sum_{i=1}^{n} \mathrm{p_{ijt}RR_{ijt}}}$$

c) Daily NUTS2 PAF data were merged with daily mortality counts at the same temporal and spatial scale and were multiplied to obtain the daily attributable mortality to wildfire-$PM_{2.5}$ exposure at the NUTS2 level. We summed the counts to form yearly aggregates for the NUTS 2 regions with complete data for a given year. Estimates of yearly attributable deaths at the NUTS2 level were further added at the country, regional and Europe-wide level.

d) Confidence Intervals (CI) of the yearly attributable deaths were calculated by propagating the uncertainty of the exposure-response function via Monte Carlo simulation (Khomenko *et al.*, 2021).[51] Briefly, we assumed the natural logarithm of the RR to follow a normal distribution with mean equal to the natural logarithm of the point estimate and Standard Deviation (SD) computed from the CI (RR CI: 1.0044–1.0086 per 10μg/m³ $PM_{2.5}$ increase) as:

$$\mathrm{SD} = \frac{\ln(\mathrm{upper}) - \ln\,(\mathrm{lower})}{\mathrm{qnorm}(0.975) * 2}$$

Then, 200 realisations of that distribution were simulated and exponentiated to obtain 200 realisations of the RR. With them, steps 6a-c were repeated to have 200 estimates of the yearly attributable mortality at the NUTS 2 and country level. 95% CI were computed by taking the percentiles 0.025 and 0.975 of the attributable mortality distribution per year and spatial unit.

7. Annual average population-weighted FWI at NUTS2 level was calculated by first obtaining annual averages from the daily FWI grids. The resulting annual grids at the 0.5º resolution were resampled at the SILAM wildfire-$PM_{2.5}$ 0.1º resolution using bilinear interpolation. Once the FWI, NUTS2 and population grids were spatially aligned, we stacked, transformed them into tabular format, and computed population weights adding up to 1 within each NUTS2 area. With these, we computed a population-weighted annual average of the FWI. Annual FWI averages at the country, regional and European-wide level were computed using the same approach.

As an additional analysis included in the visualizations of the indicator, linear trends in regional and national annual average population-weighted wildfire-$PM_{2.5}$ exposure and FWI were computed by fitting a linear model per spatial unit with the yearly exposure/risk as a dependent variable and the year as an independent variable. Slope coefficients of the linear regressions were used as trend estimates. Statistical significance of the trends was assessed via p-values of a t-test for the slope coefficients under a null hypothesis of $\beta = 0$.

Two sensitivity analyses were run:

1. Annual average wildfire-$PM_{2.5}$ exposure and FWI risk by European region without population weighting, i.e. a purely spatial mean giving all pixels within each region the same weight, was computed to assess whether proximity of population and fire events played a role in the observed trends. Similar to the main

results, this analysis showed non-significant trends in wildfire-$PM_{2.5}$ while fire risk according to the FWI increased in all regions except Northern Europe (Figure A3). This suggests that the location of wildfires and population within region do not play a major role in the observed trends.

2. An alternative exposure-response function was used to examine the potential increased toxicity of wildfire smoke compared to total $PM_{2.5}$ mass concentration.[52] The chosen estimate came from a global multi-city time series study which examined the association between wildfire-$PM_{2.5}$ and all-cause mortality[53] we used the global pooled relative risk (lag0) reported therein: 1.021 (95% CI 1.018–1.024) for 10 µg/m3 increase of wildfire-PM2.5. When using this estimate, the number of annual attributable deaths more than tripled (Table A2).

The R code used to construct this indicator and create the visualizations included in this publication is available at the following repository: https://github.com/carlesmila/LCDE2024_wildfires. The SILAM model can be found here: http://github.com/fmidev/silam-model.

Inequality context

We analysed the distribution of average (2003-2022) population-weighted wildfire-$PM_{2.5}$ and wildfire risk according to the Forest fire Weather Index (FWI) at the NUTS2 level by deprivation level (Figure A4). The source of the deprivation data was the dataset *Severe material deprivation rates* published by EUROSTAT and was only available for a subset of the areas we analysed. Results showed greater wildfire smoke exposure and wildfire risk in highly deprived NUTS2 areas compared to medium and low-deprived areas (Figure A4).

Caveats

The list of caveats for the indicator is as follows:

1. GEOSTAT population grids included a limited set of years, and therefore we had to estimate population for years where gridded population data was not available using linear interpolation. Furthermore, no population estimates were available for a set of countries (Albania, Croatia, Cyprus, Montenegro, North Macedonia and Serbia) at the 2006 edition, which had to be imputed with data from 2011.
2. NUTS 2 regions were used in our analyses, except for Croatia, Germany, Ireland and Slovenia, which were analysed at the NUTS 1 level for the health impact sub-indicator due to limited mortality data availability.
3. The temporal resolution of the Eurostat mortality time series (weekly) was different than the temporal resolution of the exposure and the exposure-response function (daily). As a result, an equal number of deaths across the 7 weekdays had to be assumed to be able to estimate the health effects. Furthermore, the temporal availability of the weekly death count data varied widely across countries.
4. There is still currently no widely accepted exposure-response function specific to wildfire-$PM_{2.5}$. The epidemiologic body of literature linking wildfire smoke and health effects is still limited, particularly for European populations; and heterogeneous, with varying exposure assessment methods between studies. The exposure-response function used in the indicator assumes a similar toxicity and exposure range of wildfire $PM_{2.5}$ and $PM_{2.5}$ from other sources, which evidence suggests may not be true.[52]
5. Regarding the fire emission system, to-date, MODIS active fire counts and fire radiative energy products are arguably the best source of fire information worldwide. However, as every low-orbit satellite, MODIS

suffers from omission errors. These have two causes: (i) cloud obscuration, including the fire obscuration by own smoke plumes, (ii) limited sensitivity of the instrument causing omission of small fires.[54] In Europe, during the local fire season, the omission error can be close to ~20-30%, as suggested by Sofiev, in preparation. The clear-sky detection limit depends on the viewing angle and time of the day, such that at night fires down to 4 MW can be detected. During the day the detection limit at the edge of the viewing area can be as large as 40 MW.

Future form of the indicator

In order to overcome the limitations of the exposure-response function, new epidemiologic studies and meta-analyses will provide more robust estimates of the association, as well as possible effect modifiers. Once they become available, exposure-response functions will be modified accordingly. Subject to resources, European-specific exposure-response functions will be estimated in an epidemiological study, including weekly exposure-response functions that match the temporal resolution of the mortality data, as well as exposure-response functions for population subgroups that may be more vulnerable, such as the elderly or outdoor workers.

In order to rectify the fire omission problem, several options will be explored. Firstly, more satellites will be used, e.g. VIIRS and SLSTR, producing similar products but providing the data at different overpass times. Secondly, utilization of geostationary instruments, such as SEVIRI for Southern Europe, will be explored. Thirdly, the emerging technology of the fire data assimilation and fusion will allow for breakthrough improvements, essentially merging together the fire models and (incomplete) satellite observations.

Additional analysis

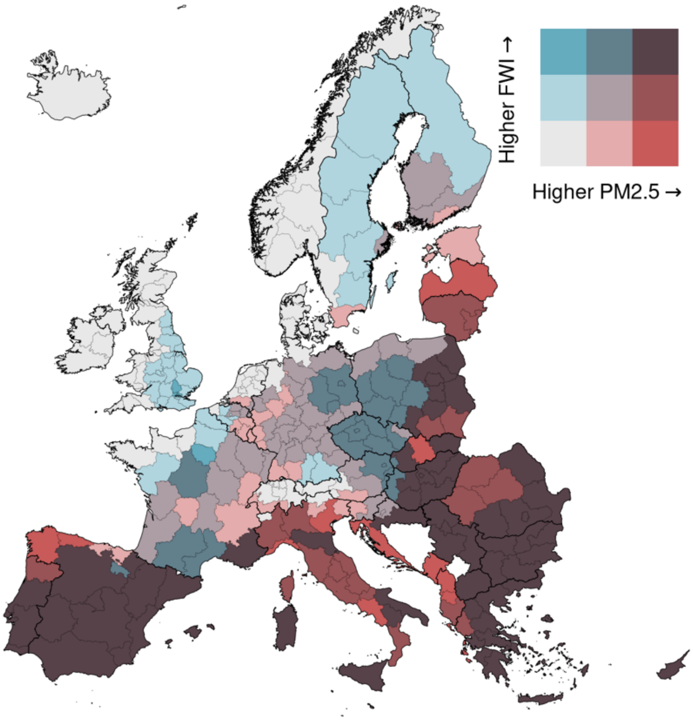

Figure 1.16 Bivariate choropleth map of average (2003-2022) population-weighted wildfire-$PM_{2.5}$ and wildfire risk according to the Forest fire Weather Index (FWI) at the NUTS2 level. Each of the two indicators is divided into tertiles for colour assignment. No data are available for Bosnia and Herzegovina and Kosovo (under UNSC resolution 1244) due to a lack of population data.

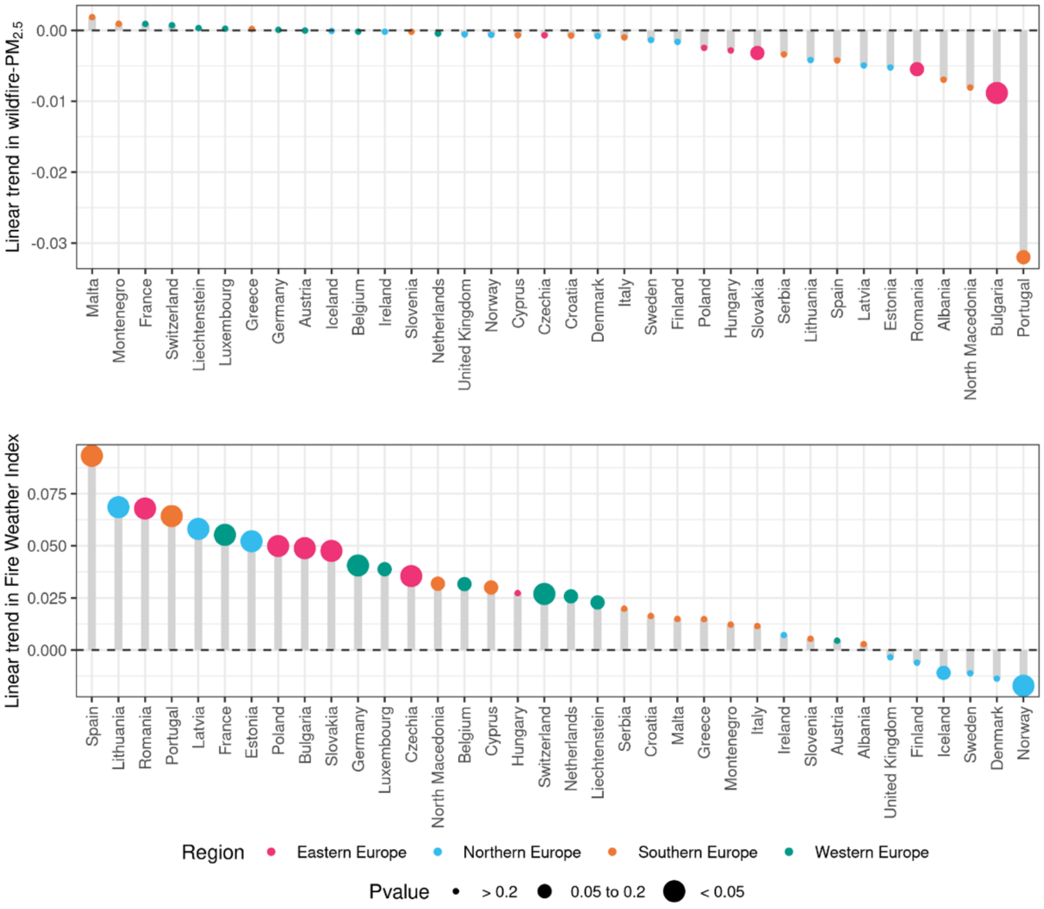

Figure 1.17 Linear trends in annual average population-weighted wildfire-$PM_{2.5}$ (2003-2022, top) and fire risk (1980-2022, bottom) according to the Forest fire Weather Index (FWI) at the country level. The dot size indicates the statistical significance of the trend coefficient and the colour the region each country belongs to.

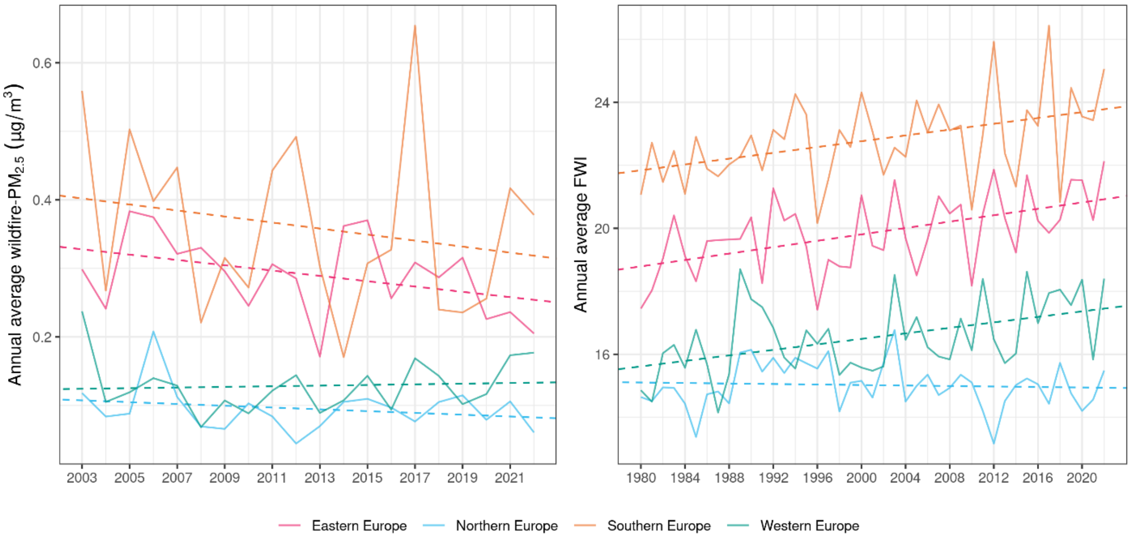

Figure 1.18 Annual average wildfire-$PM_{2.5}$ exposure (left) and wildfire risk (right) according to the Forest fire Weather Index (FWI) by European region (bold) and linear trend (dashed) without population weighting, i.e. pure spatial averaging within region. None of the wildfire-$PM_{2.5}$ trends were statistically significant (pvalues > 0.05), while FWI trends for Eastern, Southern, and Western Europe had pvalues < 0.01.

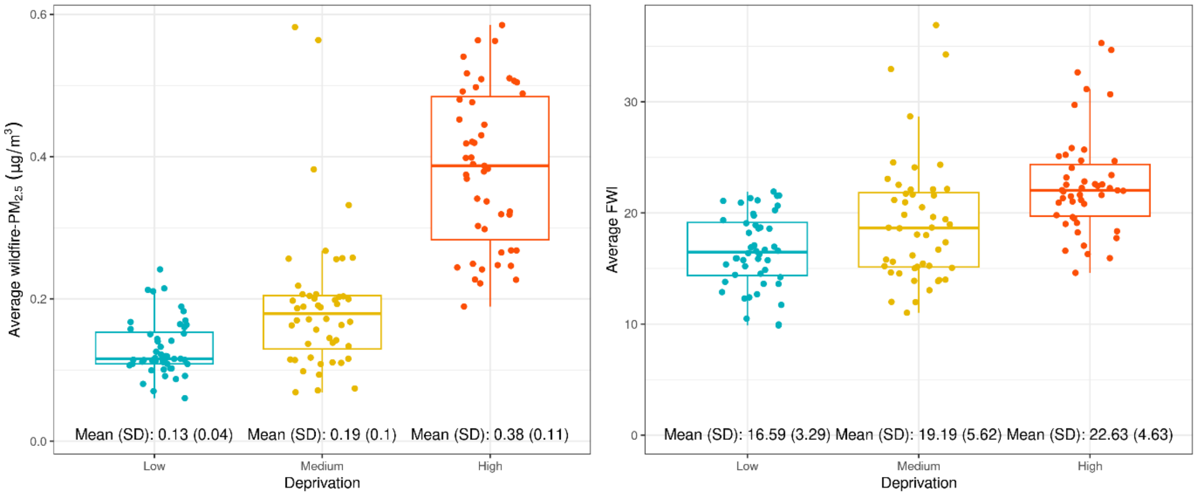

Figure 1.19 Distribution of average (2003-2022) population-weighted wildfire-$PM_{2.5}$ and wildfire risk according to the Forest fire Weather Index (FWI) at the NUTS2 level. Only the subset of NUTS2 areas with available deprivation data were included in the visualization.

Table 1.3 Trends in annual population-weighted average wildfire-$PM_{2.5}$ exposure and wildfire risk according to the Forest fire Weather Index (FWI) by European region as shown in the main figure of the report. Trend coefficients correspond to the estimate of a linear regression where the annual exposure/risk is the dependent variable and the year in the independent variable.

Metric	Region	Trend coefficient (95% CI)	pvalue
Wildfire-$PM_{2.5}$	Eastern Europe	-0.004 (-0.008, 0)	0.07
Wildfire-$PM_{2.5}$	Northern Europe	-0.001 (-0.004, 0.001)	0.4
Wildfire-$PM_{2.5}$	Southern Europe	-0.004 (-0.014, 0.006)	0.39
Wildfire-$PM_{2.5}$	Western Europe	0 (-0.003, 0.003)	0.9
FWI	Eastern Europe	0.049 (0.023, 0.074)	0
FWI	Northern Europe	0.006 (-0.021, 0.033)	0.66
FWI	Southern Europe	0.033 (0.005, 0.061)	0.02
FWI	Western Europe	0.044 (0.015, 0.072)	0

Table 1.4 Annual European-wide estimated attributable deaths to wildfire-$PM_{2.5}$ according to the two exposure response functions used in the indicator. Note that the number of countries included in the estimate varies between years due to availability of weekly mortality data.

Year	Main analysis[1]	Sensitivity analysis[2]	Number of included countries
2003	563.5 (383, 755.1)	1805.5 (1551, 2075.4)	22
2004	285.6 (194, 382.8)	915.9 (787, 1052.8)	22
2005	502.4 (341, 673.1)	1607.1 (1382, 1846.2)	22
2006	537.5 (366, 719.6)	1711.1 (1472, 1963.6)	24
2007	441.4 (300, 590.7)	1402.9 (1208, 1609.5)	25
2008	251.3 (171, 336.8)	805.4 (692, 925.6)	25
2009	355.5 (242, 476.5)	1140.1 (980, 1310.4)	25
2010	307.9 (209, 412.5)	985.9 (847, 1132.8)	25
2011	466.1 (317, 623.8)	1481.1 (1275, 1699)	25
2012	546.3 (371, 732.3)	1752.2 (1506, 2014)	27
2013	390 (265, 522.6)	1248.3 (1073, 1434.3)	28
2014	417.3 (283, 559.3)	1339.8 (1151, 1540.6)	28
2015	751.7 (511, 1007.4)	2409 (2070, 2768.4)	34
2016	590.8 (401, 791.7)	1892.4 (1626, 2174.6)	34
2017	989.1 (672, 1325.5)	3168.1 (2723, 3640.3)	34
2018	641.7 (436, 860)	2056.4 (1767, 2363.2)	34
2019	606.6 (412, 812.9)	1944.5 (1671, 2234.8)	34
2020[3]	596.6 (405, 799.7)	1912.8 (1644, 2198.4)	34
2021[3]	929.9 (632, 1245.1)	2961.8 (2549, 3399.2)	32
2022	737.2 (501, 988)	2363.6 (2031, 2716.6)	31

[1] The main analysis used a daily total mass $PM_{2.5}$ – all-cause mortality exposure-response linear function (RR: 1.0065; 95% CI: 1.0044–1.0086 for a 10μg/m^3 $PM_{2.5}$ increase) extracted from a meta-analysis by Orellano *et al.* 2020.

[2] The sensitivity analysis used a daily wildfire-$PM_{2.5}$ – all-cause mortality exposure-response linear function (RR: 1.021; 95% CI: 1.018–1.024 for a 10μg/m^3 wildfire-$PM_{2.5}$ increase) extracted from multi-city global study by Chen *et al.* 2021.

[3] Estimates for years 2020 and 2021 should be considered with caution since there was an excess mortality due to the COVID-19 pandemic in many European countries.

Table 1.5 Top 20 annual attributable mortality to wildfire-$PM_{2.5}$ episodes by NUTS2 region according to the exposure-response function by Orellano et al (2020).[45] Presence in this table is subject to mortality time series data availability.

Year	NUTS2	Country	Attributable deaths (95% CI)
2021	Attiki	Greece	96.7 (66, 128.3)
2011	Norte	Portugal	52.8 (36, 70.2)
2005	Centro	Portugal	42.5 (29, 57)
2017	Norte	Portugal	40.5 (28, 54.3)
2005	Norte	Portugal	40.3 (27, 53.9)
2021	Sicilia	Italy	40 (27, 53.7)
2013	Norte	Portugal	36.1 (25, 48.3)
2007	Norte	Portugal	34.4 (24, 45.4)
2009	Norte	Portugal	33.8 (23, 45.3)
2006	Area Metropolitana de Lisboa	Portugal	31.8 (22, 42.1)
2017	Centro	Portugal	30.1 (20, 40.5)
2011	Galicia	Spain	29.9 (20, 40)
2017	Campania	Italy	28.3 (19, 37.9)
2017	Andalucia	Spain	27.6 (19, 37)
2003	Centro	Portugal	27.5 (19, 36.9)
2016	Centro	Portugal	26.6 (18, 35.7)
2017	Sicilia	Italy	26.6 (18, 35.6)
2006	Norte	Portugal	26 (18, 34.8)
2010	Norte	Portugal	26 (18, 34.8)
2013	Centro	Portugal	25.9 (18, 34.8)

Indicator 1.2.2: Drought

Geographic Coverage of Europe

The indicator covers the river sub-basin catchments present in EEA member countries and cooperating countries. It includes 32 member countries, such as the 27 European Union Member States, Iceland, Liechtenstein, Norway, Switzerland, and Türkiye. The six West Balkan countries, namely Albania, Bosnia and Herzegovina, North Macedonia, Montenegro, Serbia, and Kosovo (under UNSC resolution 1244), are considered cooperating countries. However, Italy, UK, and Türkiye were excluded from the analysis due to incomplete information on water scarcity for the study period 2000-2019. The classification of European countries as Southern, Western, Eastern, and Northern aligns with the UN Geoscheme.

Data

1. *Climate/weather data:*

The monthly means of daily maximum temperature, dew point temperature, precipitation, 10-m wind speed, incoming solar radiation, relative humidity and atmospheric pressure at surface from the ERA5-Land Reanalysis product[24] have been used to estimate drought indicator. The data cover the period from 1950-present at a spatial resolution of 0.1° globally. ERA5-Land is produced and updated by the European Centre for Medium-Range Weather Forecasts (ECMWF) within 3 months of real time.

2. *Water scarcity data:*

For the analysis of water scarcity conditions at the river sub-basin level, we utilized an advanced geo-referenced implementation of the Water Exploitation Index - WEI+ developed by the EEA, which provides valuable insights into water scarcity. The analysis incorporated data from 292 river sub-basin catchments across Europe, excluding those in Italy, the UK, and Türkiye. The dataset covers the period from 2000 to 2019, and the index is available at a seasonal resolution.[55] Updates for the WEI+ dataset are scheduled every two years.

Methods

In this assessment, we use Standardized Precipitation-Evapotranspiration (SPEI12), to assess the drought conditions, and Water Exploitation index plus (WEI+), to study the water scarcity over the European river sub-basin during the years 2000-2019.

SPEI12 is a widely used global drought indicator that combines precipitation and evapotranspiration data to assess hydrological drought conditions. SPEI12 is the standardized estimate of the climate water balance, which is defined as the difference between monthly precipitation and potential evapotranspiration (PET). The FAO-Penman Monteith equation is employed to determine PET values due to its broad applicability and higher accuracy compared to simpler methods.[56] The methodology involves aggregating and standardizing the monthly climate water balance values over a twelve-month period leading up to the target month of interest.[57] For instance, to

estimate SPEI12 for January, the monthly climate water balance values from the preceding February to January are accumulated and standardized. Likewise, SPEI12 for February is the standardized estimate of the climate water balance values from previous March to February, and so on.

Positive SPEI12 values indicate wet conditions, while negative values indicate dry conditions. For this analysis, to study the impact of different drought conditions on water scarcity, three severity levels of drought were defined based on SPEI thresholds provided by the Federal Office of Meteorology and Climatology MeteoSwiss,[58] as described in **Table 1.6**.

Table 1.6 Drought severity classification based on SPEI12 indicator.

SPEI12	Event description
-0.80 to -1.29	Moderate drought
-1.30 to -1.59	Severe drought
less than -1.60	Extreme drought

The WEI+ data used in our study was obtained from the EEA. WEI+ is a water scarcity indicator, which provides an evaluation of the pressure or stress on freshwater resources as a consequence of human activity in terms of water abstractions. It is defined as the total net water use (abstractions minus returns) divided by the available freshwater resources of a region, including upstream inflowing water. WEI+ values were calculated as quarterly averages per river sub-basin for the years 2000-2019, based on the European catchments and rivers network system (ECRINS). The four quarters correspond to winter (Q1: January, February, March), spring (Q2: April, May, June), summer (Q3: July, August, September), and autumn (Q4: October, November, December).

The WEI+ values are expressed as percentages. To distinguish water scarcity levels across Europe, we adopted the categorization provided by the EEA: values below 10% indicate low water stress, values between 10% and 20% indicate moderate stress, values between 20% and 40% imply high water stress, and values above 40% indicate severe water stress (EEA, 2021).

In this study, the aim is to analyze the frequency of different drought conditions and resulting water scarcity in European river sub-basins from 2010 to 2019, compared to the previous decade (2000-2009). The river sub-basins were categorized into four regions of Europe (Southern, Western, Eastern, and Northern), based on the countries where the majority of each river sub-basins area fell. The percentage increase or decrease in the number of river sub-basins that have experienced moderate, severe, and extreme drought conditions during the years 2010-2019, in comparison to the previous ten years, is estimated to study the drought frequency of each considered European region. The frequency of drought occurring in different seasons of the year has been analyzed by considering SPEI12-March, SPEI12-June, SPEI12-September, and SPEI12-December to study the developed winter, spring, summer, and autumn drought conditions, respectively.

Furthermore, to examine the impact of drought conditions on water scarcity in the four European regions, we estimated the percentage of regions experiencing low, moderate, high, and severe water scarcity during different drought severity levels (moderate, severe, extreme) from 2010 to 2019 and compared it with the period from 2000 to 2009. Similar to the drought frequency analysis, we associated the water scarcity of each specific season with the drought conditions that developed in the preceding 12 months of the considered season. For example, summer water scarcity was studied using SPEI12-September, autumn drought conditions using SPEI12-December, and so on. Overall, these assessments allow us to track the frequency of varied drought conditions and resulting water scarcity in European river sub-basins over the specified season, providing insights into the changes and patterns observed in the regions of interest.

The code used to construct this indicator and create the visualizations included in this publication is available at the following repository: https://earth.bsc.es/gitlab/ghr/lcde-drought.

Inequality Context

Droughts exert a significant influence on inequality within societies. The effects of drought are often felt most acutely by vulnerable populations who lack resources and social support systems to cope with its consequences.[59] However, it is important to note that due to the complex nature of drought and the limited availability of reliable data on vulnerable populations affected by drought across Europe, it is not feasible to stratify this indicator across specific subgroups. The indicator used in this study focuses on tracking the climatic impact of drought conditions on human communities by utilizing essential climate variables, including precipitation and temperature datasets, rather than relying on case or burden data.

Caveats

The outcomes presented in this study focuses on European river sub-basins and covers the period from 2000 to 2019. While this timeframe provides valuable insights into the patterns and trends of drought and water scarcity, it is important to point out that long-term trends may not be fully captured within this limited time frame. Future assessments extending the analysis to include longer periods could offer a more comprehensive understanding of drought and water scarcity patterns. Moreover, the categorization of drought severity levels and water scarcity levels is based on predetermined thresholds provided by relevant sources, such as the MeteoSwiss and EEA. These thresholds have inherent subjectivity but may not capture the full range of drought or water scarcity conditions experienced in different regions. Therefore, caution should be exercised when interpreting the severity classifications and water scarcity levels.

Future Form of the Indicator

The developed indicator only considers the percentage of regions exposed to water scarcity due to drought, without accounting for the population residing in those regions. Future development of the indicator will focus on capturing the differential impacts of drought and water scarcity on various population groups by incorporating population weights. This development will require a better understanding of the exposure factors affecting the

European population and would contribute to a more robust analysis of the impacts of drought and water scarcity on communities.

Main & additional analysis

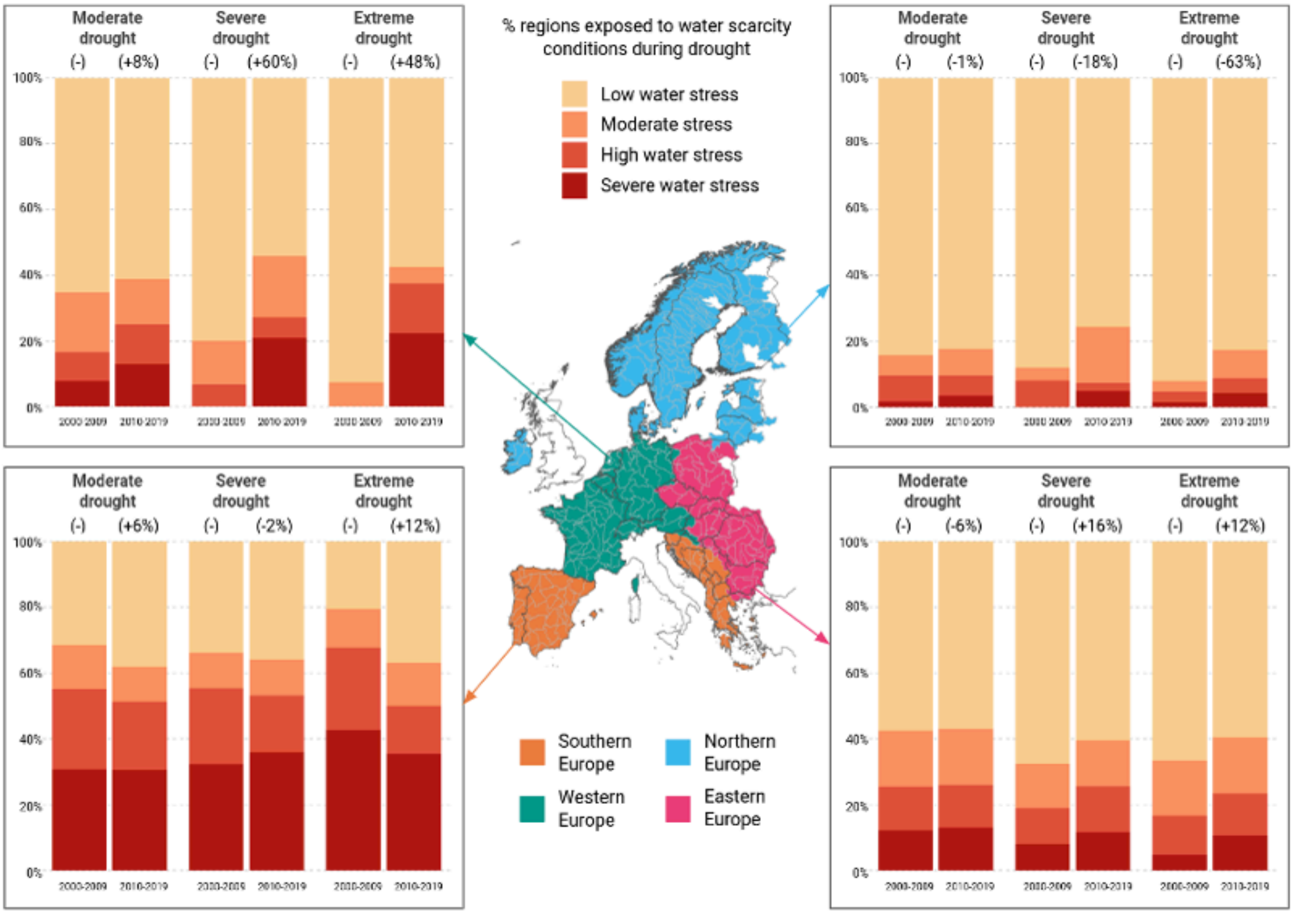

Figure 1.20 Fraction of regions exposed to water scarcity across EU regions (measured by the Water Exploitation Index plus, WEI+) during summer months (July-August-September) under varying drought severity conditions (measured by the Standardized Precipitation-Evapotranspiration Index, SPEI12-September) in 2010-2019 compared to 2000-2009. The percentage in brackets indicates the change (+/-) in the number of regions experiencing moderate, severe, or extreme summer drought conditions from 2010-2019 compared to 2000-2009.

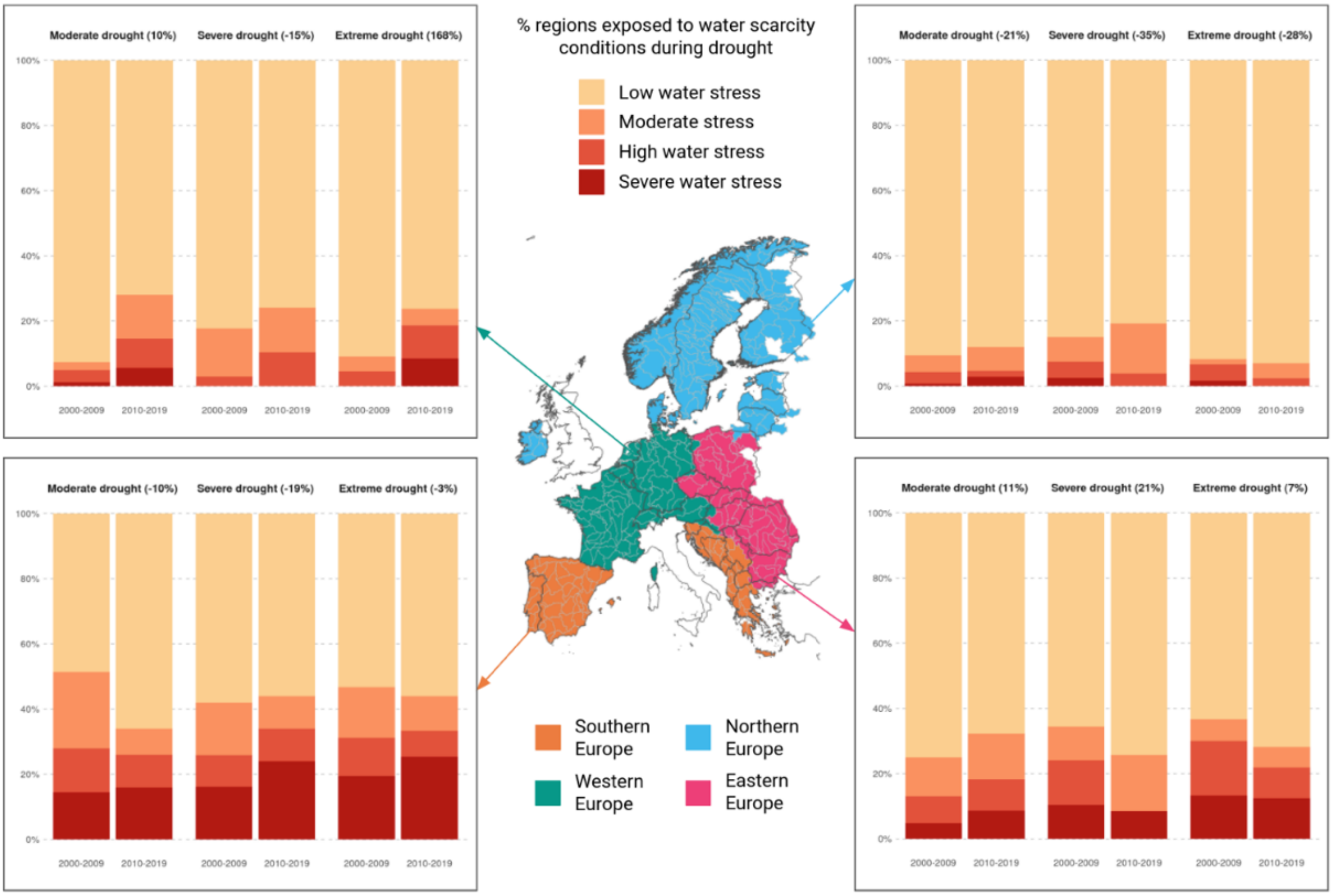

Figure 1.21 Fraction of regions exposed to water scarcity across the EU regions (measured by the WEI+) under varying drought severity conditions (measured by the SPEI12-March) during winter months (January-February-March) from 2010 to 2019 compared to previous ten years. The percentage in brackets indicates the change (+/-) in the number of regions experiencing Moderate/Severe/Extreme summer drought conditions from 2010-2019 compared to 2000-2009.

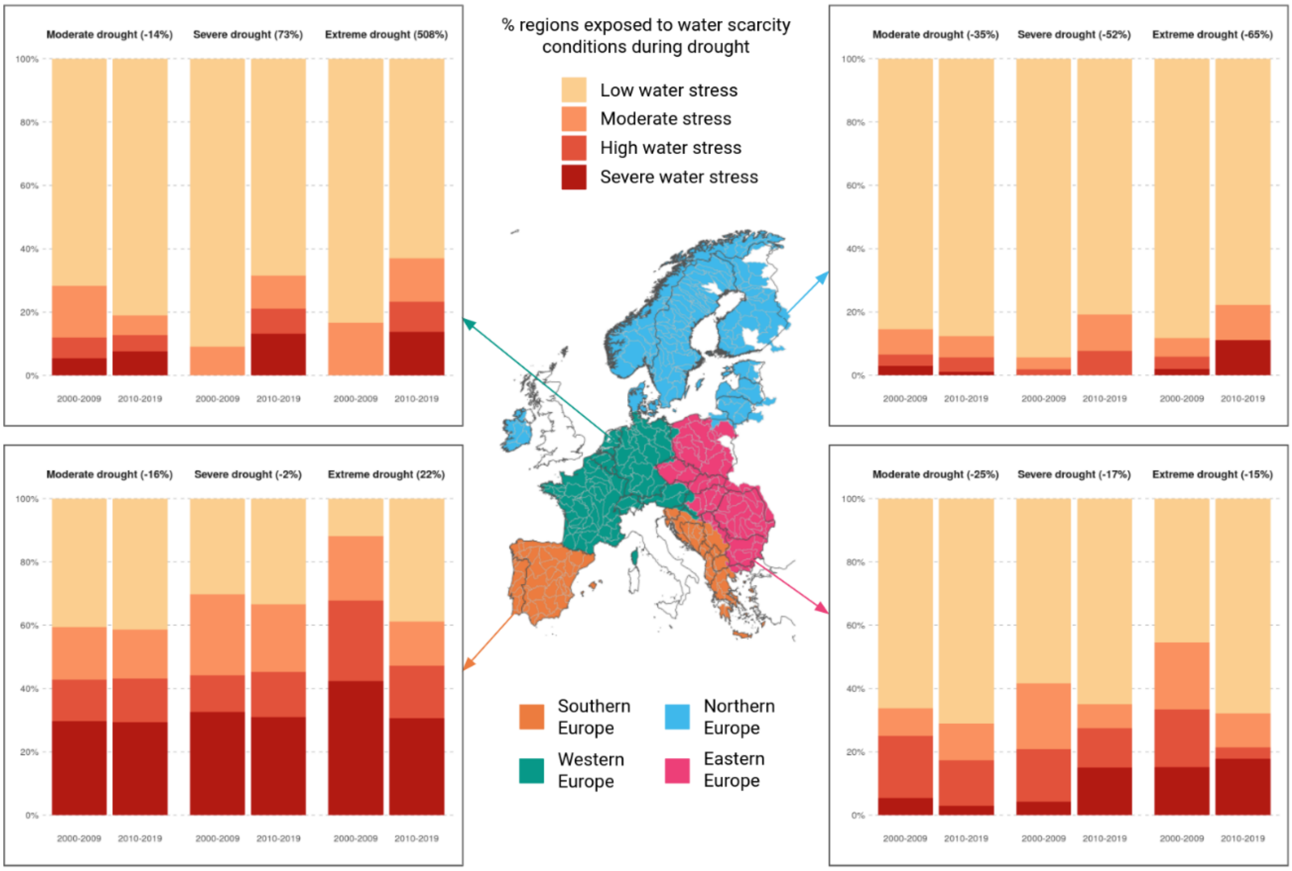

Figure 1.22 Fraction of regions exposed to water scarcity across the EU regions (measured by the WEI+) under varying drought severity conditions (measured by the SPEI12-June) during spring months (April-May-June) from 2010 to 2019 compared to previous ten years. The percentage in brackets indicates the change (+/-) in the number of regions experiencing Moderate/Severe/Extreme summer drought conditions from 2010-2019 compared to 2000-2009.

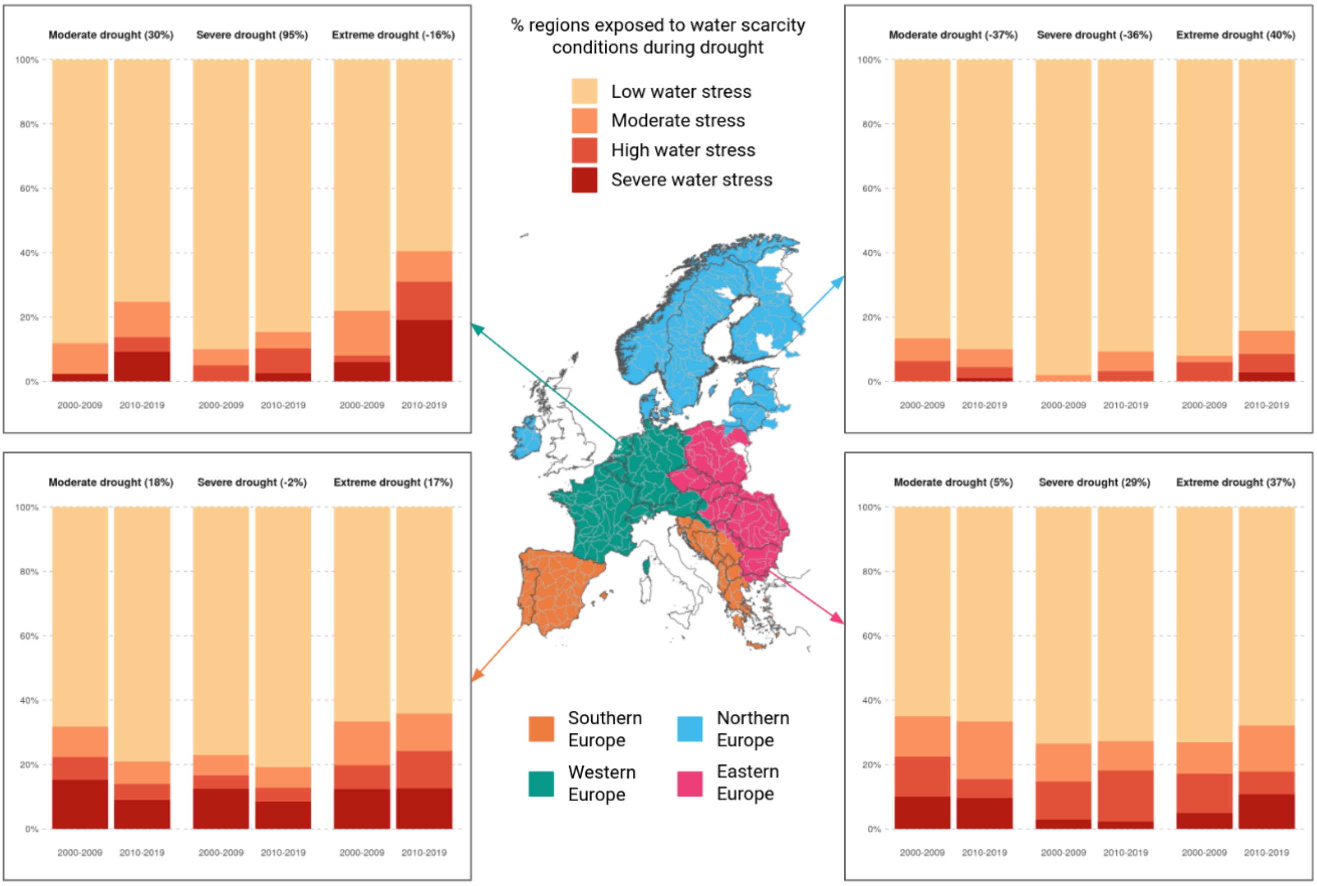

Figure 1.23 Fraction of regions exposed to water scarcity across the EU regions (measured by the WEI+) under varying drought severity conditions (measured by the SPEI12-December) during months (October-November-December) from 2010 to 2019 compared to previous ten years. The percentage in brackets indicates the change (+/-) in the number of regions experiencing Moderate/Severe/Extreme summer drought conditions from 2010-2019 compared to 2000-2009.

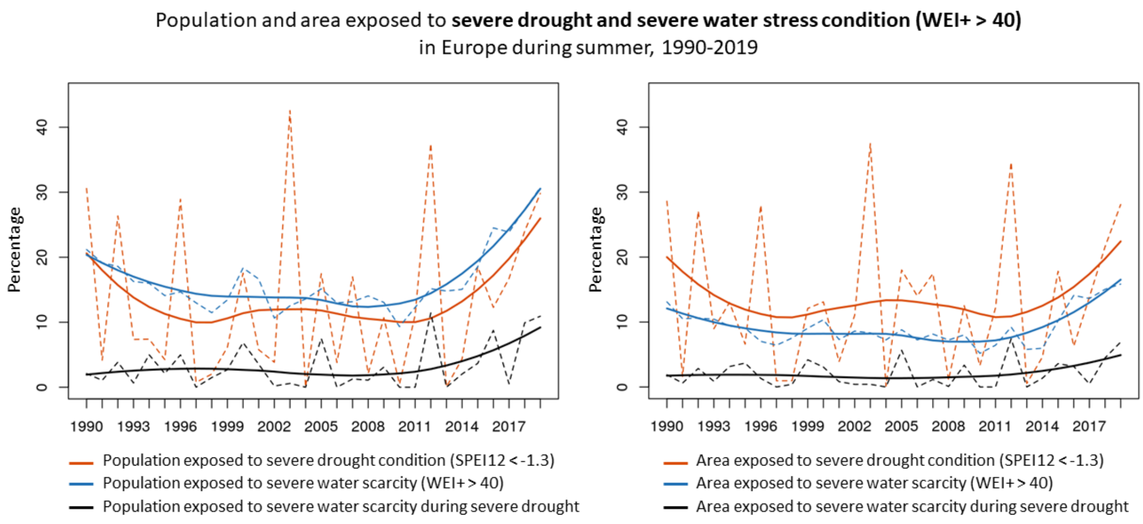

Figure 1.24 Population and area exposed to water scarcity conditions in Europe during summer months (measured by WEI+ > 40 for July-August-September) under severe drought severity condition (measured by SPEI12-September < -1.3) from 1990-2019. Dotted lines represent yearly variations in severe water scarcity and drought conditions, while thick lines correspond to the smoothed annual time series.

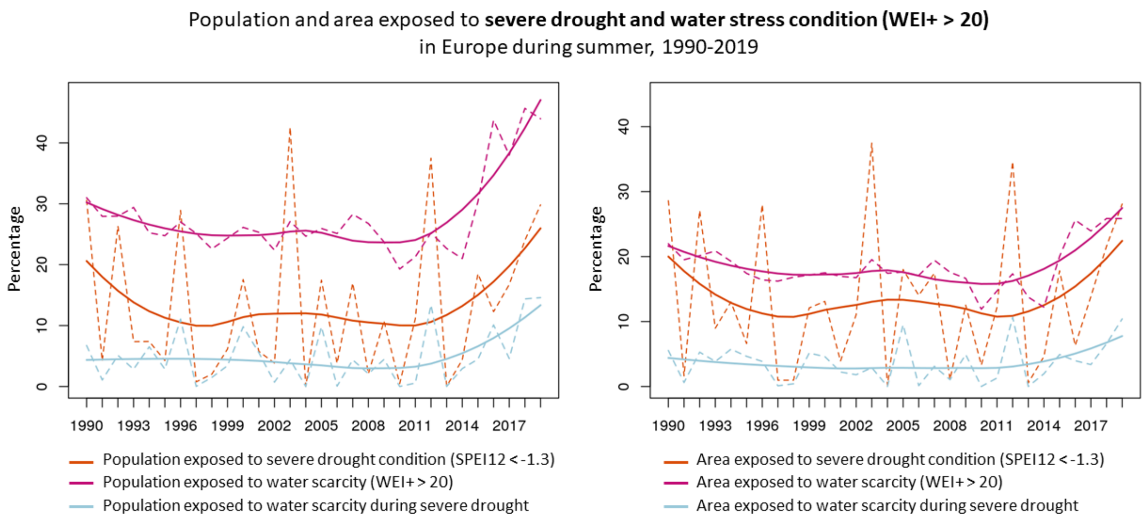

Figure 1.25 Population and area exposed to water scarcity conditions in Europe during summer months (measured by WEI+ > 20 for July-August-September) under severe drought severity condition (measured by SPEI12-September < -1.3) from 1990-2019. Dotted lines represent yearly variations in severe water scarcity and drought conditions, while thick lines correspond to the smoothed annual time series.

1.3: Climate-sensitive infectious diseases

Indicator 1.3.1: Climatic suitability for *non-cholerae* Vibrio

Geographic Coverage of Europe

All European countries listed in the current NUTS classification by EUROSTAT, which includes the 38 European Environment Agency member and cooperating countries in addition to the United Kingdom of Great Britain and Northern Ireland, Georgia, Republic of Moldova and Ukraine.

Data

- AWI-CM-1-1-HR and CNRM-CM6-1-HR sea surface temperature (SST) and sea surface salinity (SSS) from CMIP6 (2015-2100) SSP126 and SSP370experiments. Both variables are provided at monthly time steps and 25km resolution, offering an improved coastal coverage than similar products at 0.5º resolution.[60]
- Coastline length from the World Factbook data (https://www.cia.gov/the-world-factbook/). This dataset is used to estimate the trends (km/year and per country) in the length of coastline affected by Vibrio favourable conditions.
- ISIMIP2b annual global population data at 0.5º resolution for the period 2006-2100 (SSP245&SSP585) and 1850-2015 (historical) were used to estimate the population potentially affected by Vibrio infections. Our methodology takes into account a maximum distance of 100km between the pixel showing Vibrio suitability and the center of the population cell. Our simulations for each scenario use the corresponding socio-economic population dataset.[61]
- Sea surface temperature data from the Global Ocean OSTIA Sea Surface Temperature and Sea Ice Reprocessed dataset between 1982-2022.[62]
- Sea surface salinity data from the Mercator Ocean Reanalysis.[63]
- Gridded Population of the World (GPW), v4.[64]

Methods

Vibrio ecology, abundances, distributions, and patterns of infection have been proved to be strongly mediated by environmental conditions.[65,66,67,68] On the basis of previous research on the conditions favourable for the detection of *Vibrio* infections, the *Vibrio* indicator has built on thresholds of >18°C for Sea Surface Temperature (SST) and <28 PSU for Sea Surface Salinity (SSS). The *Vibrio* suitability regions were determined based on these parameters and thresholds and consequently areas showing temperatures above 18ºC and salinities below 28 psu were flagged as suitable for *Vibrio*.

For the 2024 *Vibrio* indicator projections, data from CMIP (AWI-CM-1-1-HR and CNRM-CM6-1-HR)[60] was used to estimate areas and periods of *Vibrio* suitability and population at risk. Additionally, the Inter-Sectoral Impact Model Intercomparison Project (ISIMIP) Project 2b annual global population data was employed to

compute the population at risk. The population potentially affected by exposure to *Vibrio* has been selected based on the ad-hoc distance of 100km between areas showing *Vibrio* suitability and the center of the population cell for that time period. Climate, population and socioeconomic projections were combined to generate more accurate estimates of changes in *Vibrio* suitability and provide a global estimate of the population at risk of vibriosis for 2022 compared to a 1995-2014 baseline with data coverage from 1982 to present. We applied a conservative assumption of infection rate per 100,000 population of 0.3 reported for the USA (as estimated by both COVIS-CDC and FoodNet for the USA)[69] and took in consideration the limitations of surveillance data and underreporting in the USA, scaled up the number of infections 143 times[70] to calculate a more probable incidence of disease.

Finally, we also considered the climate, population and socioeconomic projections included into the framework of the Shared Socioeconomic Pathways (SSPs)[70] to provide accurate estimates of future changes in *Vibrio* suitability and population at risk and generate projections for a low- and high-emission scenarios (SSP1-2.6. and SSP3-7.0 respectively) by the end of the century compared to the pre-industrial period.

Here suitability is reported at two levels. the length in Km of coastline that experienced suitable conditions for *Vibrio* infections and the period of suitable conditions for Vibrio in days per year. These two indicators were calculated globally (for all coastal countries) and the results summarised by country and compared to historical data since the records began in 1982.

Inequality Context

Risk factors of Vibriosis may differ among different European regions. Vibriosis is associated with two different routes of infection and can result from the contact with seawater or via consumption of seafood. Behavioural habits associated with both the use of coastal water for recreational activities and seafood consumption vary across Europe and may affect population groups with different socioeconomic status, different age groups, or with other underlying factors (liver disease, alcohol abuse) which may make these groups more frequently expose or with higher risk or prevalence of infections.). Though most of the infections are self-limiting, inequalities in access to healthcare in marginalised and lower socioeconomic populations may prevent them to received health care and result in a higher infection rate or more severe disease outcome.

Caveats

The results are derived on the basis of suitable SST and SSS conditions only, and do not include other potentially important drivers (e.g., globalisation), environmental predictors of pathogenic *Vibrio* infections (e.g., cholorphyll-*a*, turbidity) or disease case data. Nevertheless, these associations have been explored and are reported in the supporting references included above.

In the global analysis, the slope of the trendlines over the time series is mostly flat for the tropical/subtropical region and the southern Hemisphere. However, the SST-only suitability shows a strong upward trend in the southern hemisphere, indicating that on average temperature conditions are also improving growth conditions for *Vibrio* in these areas, while SSS is generally limiting. However, locally suitable SSS conditions will also occur in

these regions based on, for example, variation in local rainfall and river runoff, which can make these regions sporadically suitable for *Vibrio* infections.

Analysis

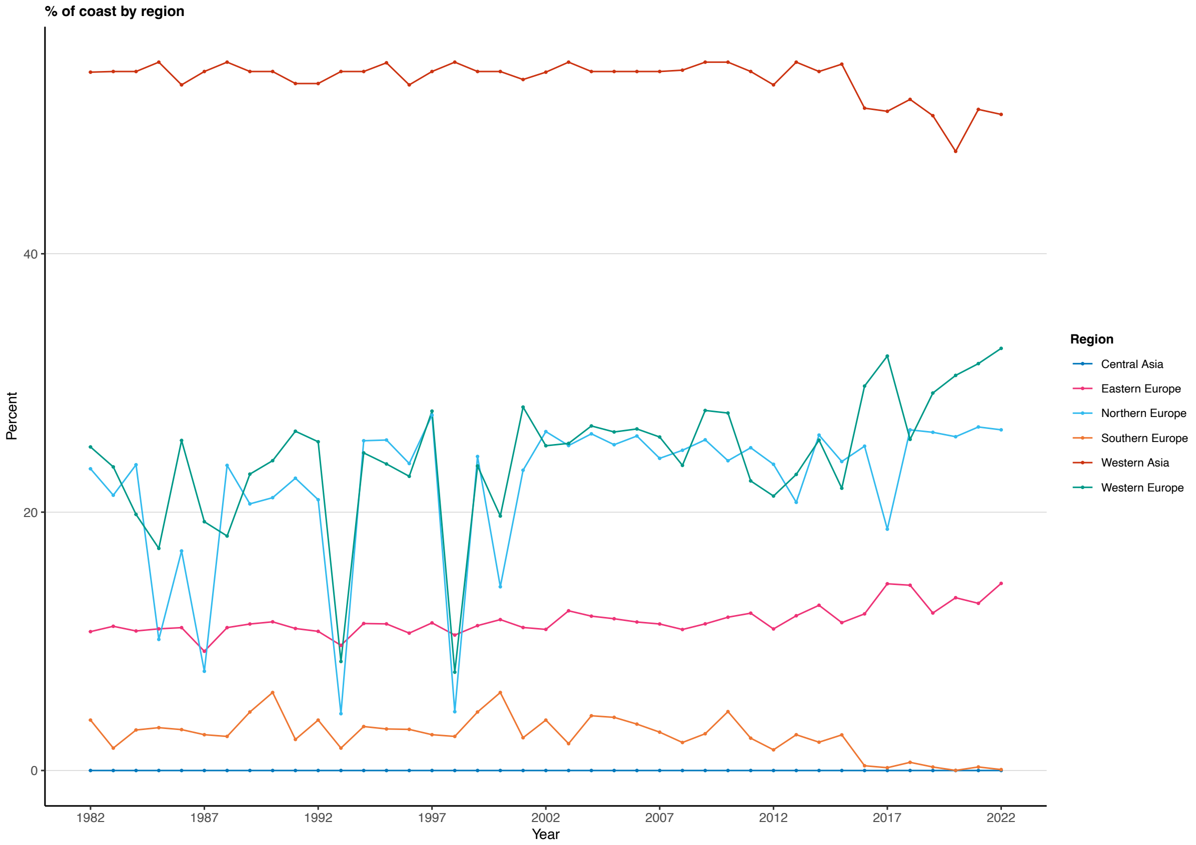

Figure 1.26 Sub-regional time series within the WHO European region illustrating the percentage of coastline showing suitable conditions for Vibrio over time. The largest variability is predominantly observed in the Western and Northern European countries.

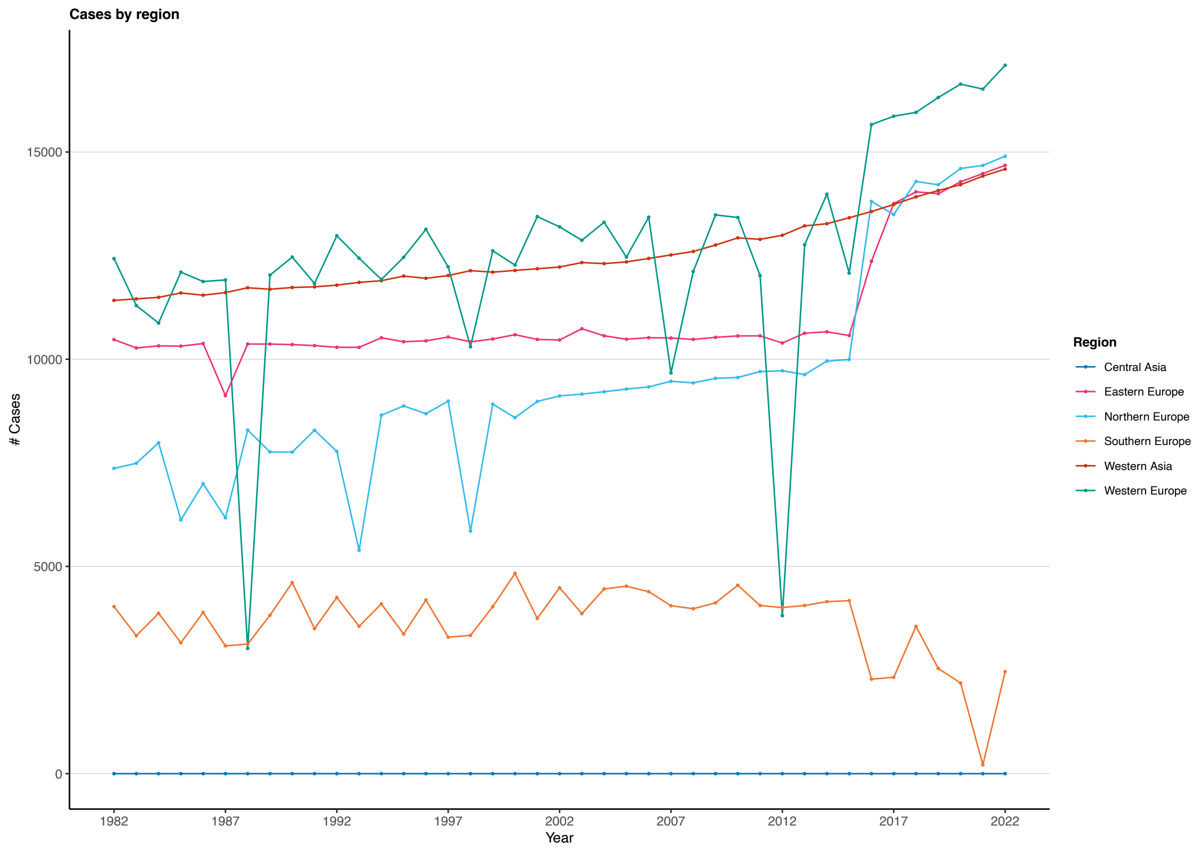

Figure 1.27 Estimated number of Vibriosis (non-Cholora) cases per sub-region within the WHO European region. The figures have been corrected for underreporting.

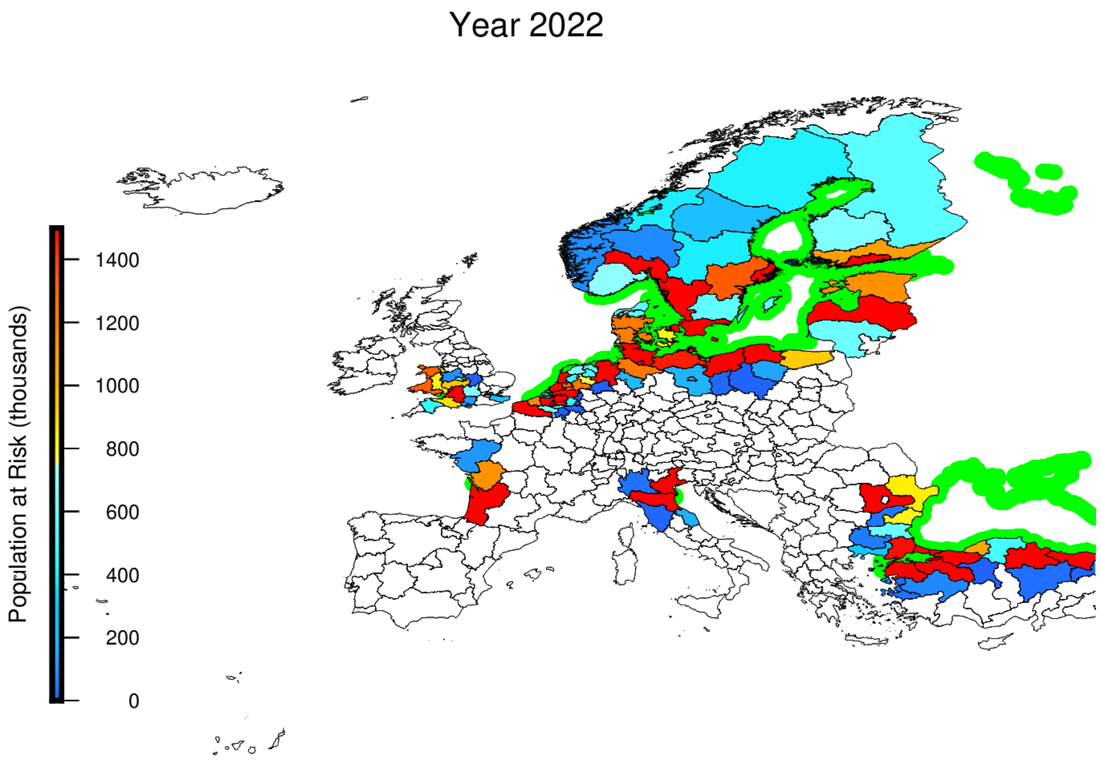

Figure 1.28 Population at risk for Year 2022 at NUTS-2 level. The areas along the coast showing suitable conditions for Vibrio are shown in green.

Indicator 1.3.2: Climatic suitability for West Nile virus

Geographic Coverage of Europe

For this indicator we included the European Environment Agency member and cooperating countries plus the United Kingdom of Great Britain and Northern Ireland - excluding Kosovo (under UNSC resolution 1244) and Liechtenstein as data was not available for these countries.

Data

For quarterly climate variables (temperatures and precipitation), ERA5 Land reanalysis data at 0.1° resolution was used.[71] Also, the 19 bioclimatic variables were extracted as predictors from the same data set.[72,73] The records WNV human infections data for the response variable were obtained from ECDC.[74] This data was available for the years 2010-2022 containing a total of 800 NUTS3 level observations of recorded human infections included in the main analysis while the remaining observation with 'unknown' NUTS3 identification were not included. The training data set was imbalanced in favour of negative classes (i.e., 96% regions with no WNV infection cases) compared to the regions with WNV transmission (4% regions with WNV human infection cases).

Methods

The European NUTS3 regions (2021 definition) spatiotemporal data consisting of climate and bioclimatic variables were used as predictors. All climatic variables, i.e., temperature, precipitation and relative humidity were averaged in four quarters. To provide covariates for the complex interactions between WNV outbreaks and climate, a set of 19 bioclimatic variables (bio01-bio19),[72] that delineate the annual tendencies were incorporated. Annual data on socioeconomic and population density variables were also added to improve the model's predictive ability.[73,75] The influence of the variables was presented by dividing regions into two groups based on transmission activity in a specific year. The transmission activity was based on the WNV human infections data. This data was obtained from the *European centre for disease prevention and control* (ECDC),[74] from 2010 to 2022. The regions with WNV human infections were attributed as positive class regions (1). The regions without any information on WNV human infections were classified as zero in the response variable.

A supervised machine learning classifier, eXtreme gradient boosting (XGBoost),[76,77] was applied to the final data set. For this, the data was split into two subsets: data from the years 2010-2019 as the training set, and the data from 1950-2009 and 2020-2022 as the testing set. For the model training and tuning, a 5-fold cross-validation approach was used. The last three years were kept in the test set firstly for consistency of reporting the relative change in risk to the previous year's report,[78] and secondly to assess the out-of-bag score of the model as well which was not possible in the previous year's report. The model output was the WNV transmission or outbreak risk as probability between 0 and 1 at the NUTS3 level. The model achieved an AUC (Area under the receiver operator characteristic curve) scores of 95% and 89% for the internal validation and out-of-bag data sets,

respectively. As there were no positive class instances available for the external validation data set, all the analyses were done without setting a classification-threshold in a departure from the usual practice to assess the performance of a machine learning algorithm.

The code used to construct this indicator and create the visualizations included in this publication is available at the following repository: https://github.com/Ziaf021/LancetEurope_WNV.

Inequality Context

When examined and stratified NUTS3 into 3 topological categories (urban, intermediate, and rural). It was found that there were more outbreaks in the regions with rural and intermediate topology as compared to the urban topology (**Figure 1.28**). The empirical trend was well captured by the model predictions as well as shown in **Figure 1.28**.

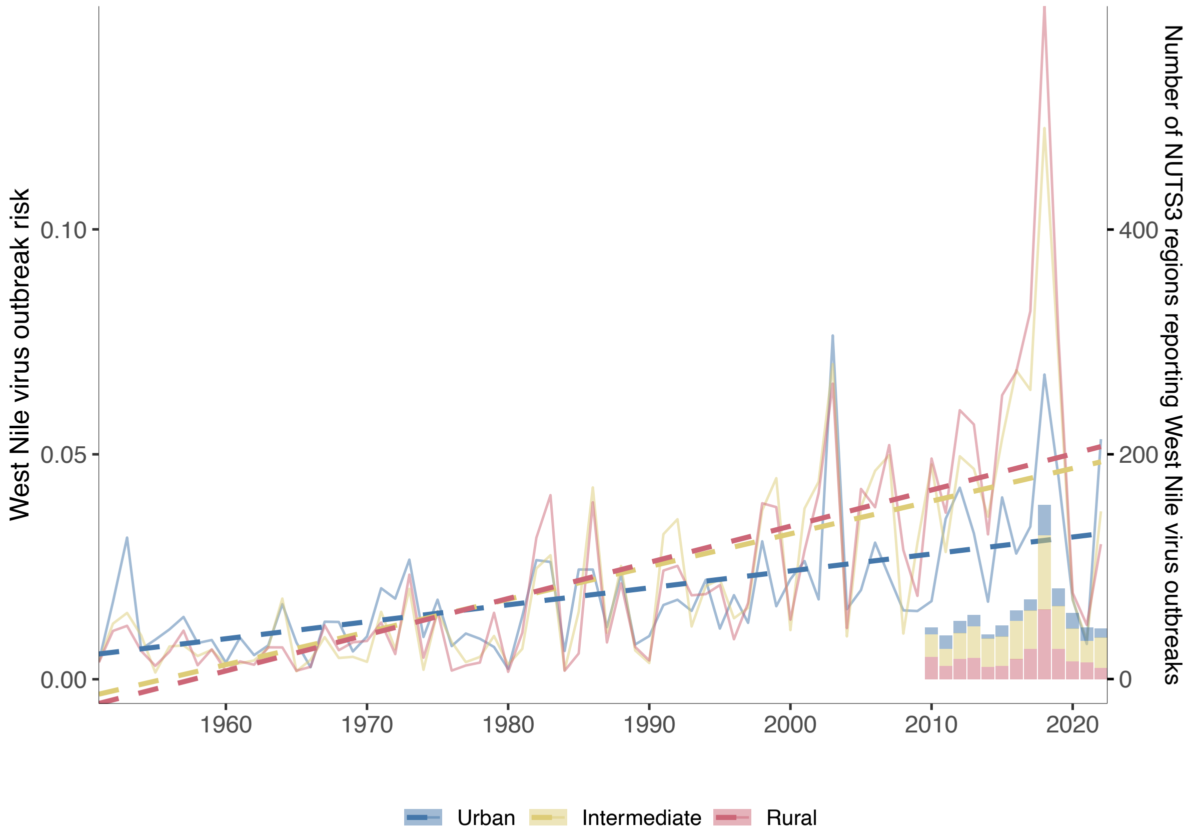

Figure 1.29 Annual West Nile virus risk trends categorized into three topological regions shown along left vertical axis. The bar plots (right vertical axis) show the corresponding number of NUTS3 reporting the virus outbreaks in each category.

Caveats

The WNV cases data related to human infections only were considered in the study. However, data on equines infections and host birds infections on the NUTS3 level could also have been incorporated upon availability. The

inclusion of the infections data for the equines and the host birds would further strengthen the model predictions though XGBoost performed reasonably well to handle the severe class-imbalance due to its flexibility of numerous hyperparameters tunning options.

Future form of the indicator

In future versions, the aim will be to improve by overcoming/ minimizing the caveats listed above, that is, by incorporating equines and equids infections data. This modification in the future indicators will likely increase the model's predictive power and robustness.

Additional analysis

Figure 1.29 shows the expected probability of WNV risk predicted by the model for each decade during the study period. Evidently, tangible increase in the outbreaks risk is observed in the regions where the virus is established. Model post-hoc analysis revealed that the average spring temperature was one of the most influential climatic variables associated with model predicted outbreak risk. A statistically significant ($p < 0.05$) correlation (R=0.68) between the most influential variable and the model predicted WNV-outbreak risk probability was found using the Pearson correlation test presented in Figure 1.30.

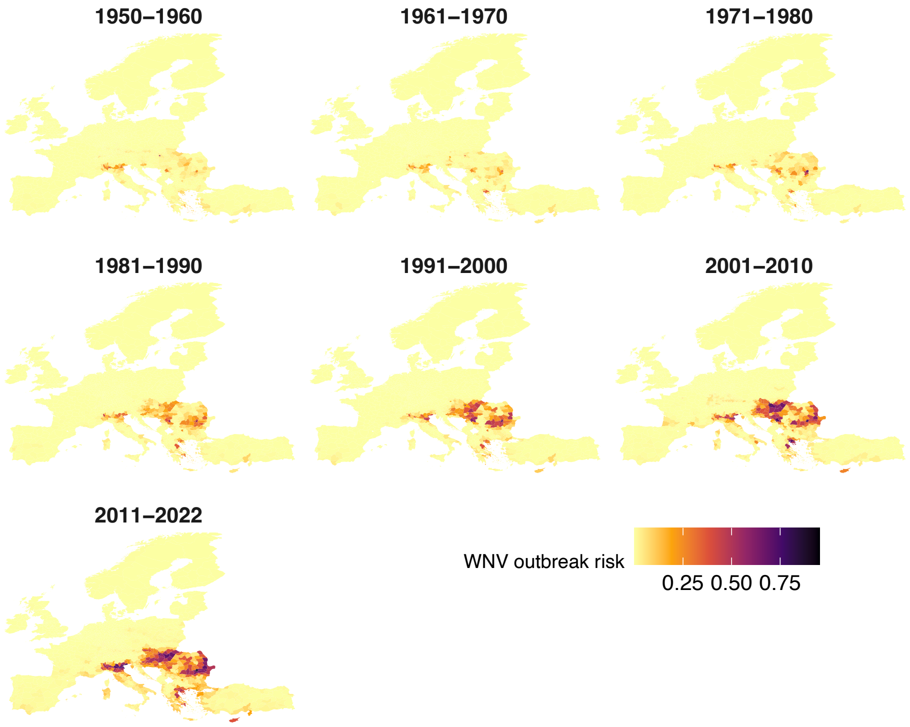

Figure 1.30 Model predicted West Nile virus (WNV)-outbreak risk for Europe at NUTS3 level from 1950 to 2022.

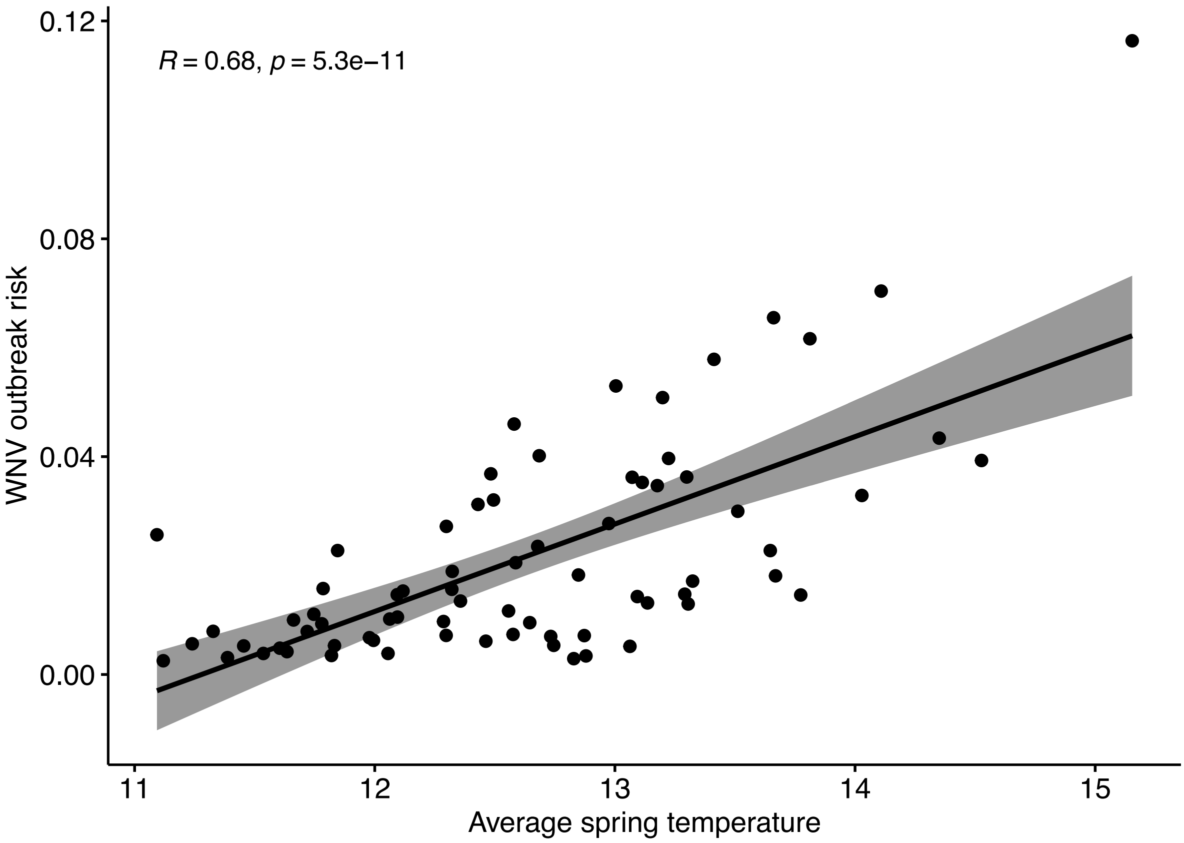

Figure 1.31 Scatter plot showing the correlation between the temperature of the 2nd quarter and the model predicted WNV risk, the correlation coefficient (R), and the p-value.

Indicator 1.3.3: Climatic Suitability to Dengue, Chikungunya and Zika

Part A

Geographic Coverage of Europe

The indicator covers European Environment Agency (EEA) member countries and cooperating countries. The geographic coverage is also extended to include the United Kingdom of Great Britain and Northern Ireland.

Data

1. ERA5-Land monthly averaged data from 1950 to 2022.[24]
2. Copernicus Climate Change Service (C3S) precipitation and temperature data.[24]
3. HYBRID gridded population data.[79]

Methods

A stage structured mechanistic model based on temperature dependent life cycle traits of *Aedes Albopictus* and *Aedes Aegypti* mosquitoes is used to estimate the mosquito abundance and subsequently basic reproduction number (R0) and length of transmission season for dengue by considering various factors such as temperature, rainfall, mosquito abundance, and human population density. The model incorporates the effects of temperature, rainfall, and other climatic factors on the traits associated with the mosquitoes' life cycles.[80] We divided the model into two stages: the immature stages (aquatic stage) and the adult stage, encompassing all phases of the mosquito life cycle. To estimate the equilibrium population of blood-feeding mosquitoes, which we refer to as mosquito abundance (M), we utilised the temperature dependent data of these traits to from previous studies[80,81,82,83] and fit them with simplest unimodal functional responses (quadratic, briere or gaussian) to avoid overfitting using the Bayesian approach.[84,85] The main emphasis of the Bayesian rule lies in the posterior distribution, which represents the probability of specific parameter values given the observed data. This distribution is obtained by combining the likelihood, which quantifies the probability of observing the data given certain parameter values, with the prior distribution, which expresses our prior beliefs about the parameters independently of the observed data. By utilizing Bayesian inference, we can effectively integrate information from related species in our analysis, particularly in cases where climate-dependent trait data is limited or scarce. This approach enables a more comprehensive and robust inference process, facilitating a broader understanding of eco - epidemiological patterns of vector transmissions.

The mosquito abundance M then was used to estimate the Vectorial Capacity (VC) which is also explained as ability of the vector to transmit the virus and the epidemic risk when the vector is exposed to the virus is considered in the vectorial capacity, which is also known as the daily reproduction rate[86] as

$$VC = \frac{a^2(T)bc(T)\exp\left\{\frac{-\mu(T)}{PDR\ (T)}\right\}}{N\ \mu(T)}\ M(T,W,S)$$

where a refers to the biting rate (per mosquito), bc denotes the vector competence, μ is the mosquito mortality rate, PDR, the parasite (in this case, viral) development rate and N is the size of the human population. T, W and S represents temperature, rainfall and sunlight respectively.

Vectorial capacity depends only on vector biology and is intrinsically related to the basic reproduction number for vector-borne diseases, R0, which is the expected number of hosts to be infected by a single infected host in a susceptible population and is formulated as

R0 = $\frac{VC\ .\ \beta}{r}$

where r is the recovery rate of infected humans (i.e. the infectious period), and is the probability of a susceptible host being infected if bitten by an infectious mosquito.

The significant factor in this approach is estimation of the mosquito-to-human ratio (M) and its relation to the basic reproduction number (R0) in vector control efforts. However, this ratio is often overlooked or estimated in a simplistic manner. To address this, we developed a model to estimate the mosquito populations of *Aedes Aegypti* and *Aedes Albopictus* separately. The original models provide results in terms of the number of *Aedes Aegypti* individuals per breeding site (X) or the number of *Aedes Albopictus* per hectare (Y).[80,82] To appropriately estimate M, which represents the mosquito population density per human population density (p), we multiplied X by a function f(p, a, c) = a * g(p, c), where a represents the number of breeding sites per human, and Y by f(p, $\frac{a}{b}$, c) = $\frac{a*g(p,c)}{b}$, where b represents the average number of breeding sites per hectare. The function g(p, c) = $\frac{p^2}{c^2+p^2}$ is an increasing sigmoidal function that captures the viability of domesticated mosquito populations in relation to human population density. By fitting the R0 data available for a subset of spatiotemporal points, we were able to determine appropriate values for a, a/b, and c, which enabled us to estimate accurate values for M.[81] Numerical calculations of vector abundance (V) and mosquito abundance will be conducted at a spatial resolution of 0.25 x 0.25 based on selected input data. Separate calculations will be performed for *Aedes Aegypti* and *Aedes Albopictus* vectors. Gridded population data based on HYDE3.2[87] will be utilised in the computation of R0. For the diseases Dengue and Zika, we used *Aedes Aegypti* vector abundance estimates to compute M. Additionally, we separately estimated M for Dengue and Chikungunya using *Aedes Albopictus* abundance estimates. Further, the annual length of transmission season (LTS) was computed by summing the number of months in a year when R0 was greater than 1. The annual average R0 values will be extracted per grid cell for Dengue (*Aedes Aegypti*), Dengue (*Aedes Albopictus*), Chikungunya (*Aedes Albopictus*), and Zika (*Aedes Aegypti*) and averaged by NUTS regions, European regions (e.g. ....) and by country. The total European R0 values represent averaged values across all countries in the European Union (EU).

The code used to construct this indicator and create the visualizations included in this publication is available at the following repository: https://github.com/Pratik697/Dengue-transmission-suitability-LCDE2024.

Inequality Context

In order to assess the inequality context, we conducted an in-depth analysis by stratifying the NUTS3 region based on its urban-rural topology. Through this stratification, we categorized the NUTS3 regions into three distinct categories: urban, intermediate, and rural. To evaluate the transmission suitability of dengue, we aggregated the NUTS3 transmission suitability values according to population for each of these topological regions. Comparing the transmission risk to the baseline values of 1951-1960, the highest relative transmission risk is observed in rural regions with a significant increase of 49.4%. In the intermediate and urban regions, the relative risk of dengue transmission compared to baseline showed a respective increase of 36.3% and 32.9%. Similar trends were also observed for transmission suitability of Chikungunya and Zika arboviral diseases. The model captures the annual changes in transmission suitability for all three topologies. Figure 1 visually represents these changes, providing insights into the evolving landscape of dengue transmission risk.

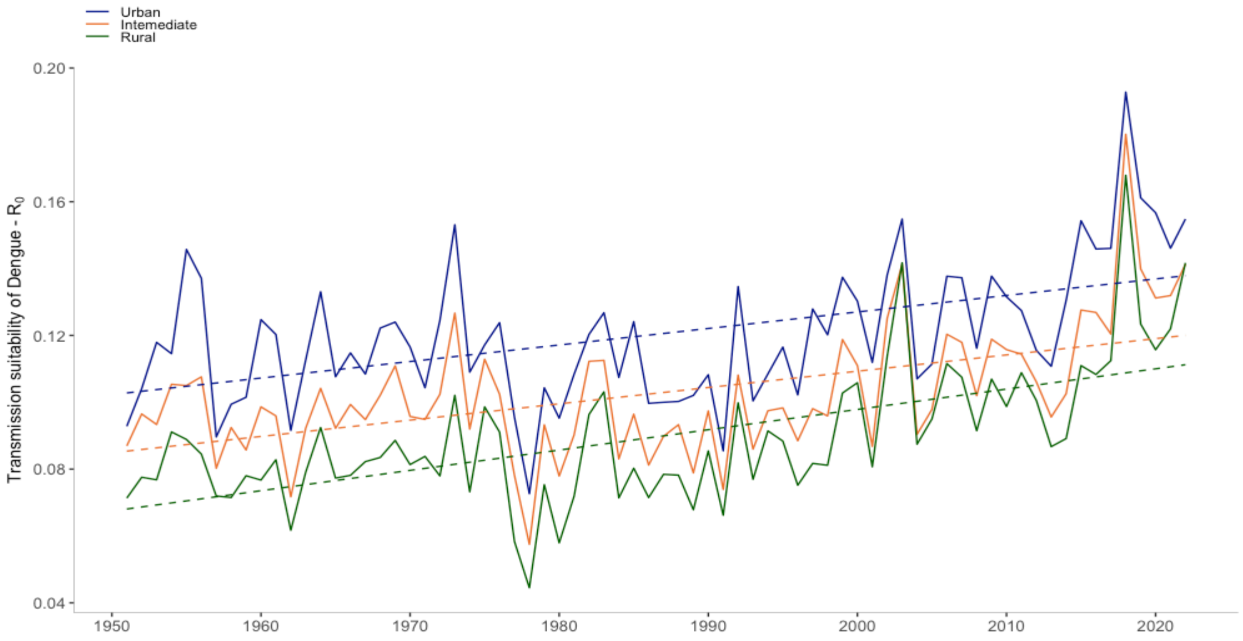

Figure 1.32 Annual change in transmission suitability of dengue in three topological region (urban, rural and intermediate).

Caveats

Key caveats and limitations of the V model and its parameterisation are fully described in Liu-Helmersson *et al.* 2014[88] and 2016[82] and Rocklöv *et al.* 2019.[86] The predicted R0 should not be confused with actual dengue cases, although it is an indicator of the potential for outbreaks.[81,89,90]

Future Form of the Indicator:

The current mechanistic model, although reliable, primarily emphasises temperature as the climatic variable in predicting disease transmission. However, there is a need to expand this model in the future to incorporate mechanistically additional climatic variables such as rainfall, daylight duration, and relative humidity based on availability of data. Additionally, we aim to establish a link between mosquito abundance, as determined by a stage-structured model, and field observations of mosquito population counts using techniques like mark-capture methods. This approach will account for uncertainties in trait responses, consider the impact of population density on trait limitations, and ultimately enable more precise estimations of disease risk.

Analysis

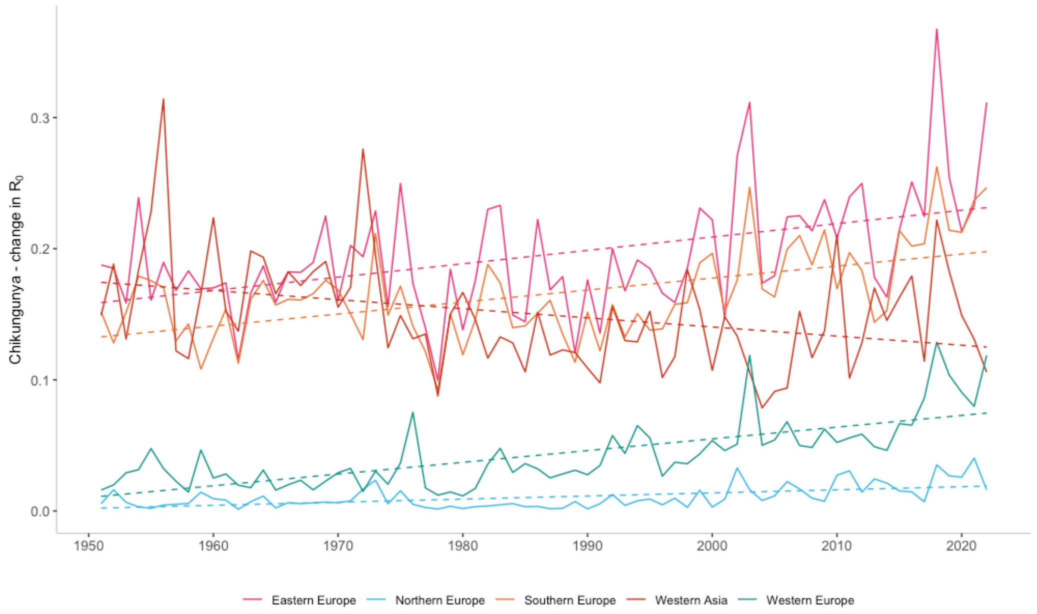

Figure 1.33 R0 for Chikungunya by European regions between 1951 and 2022.

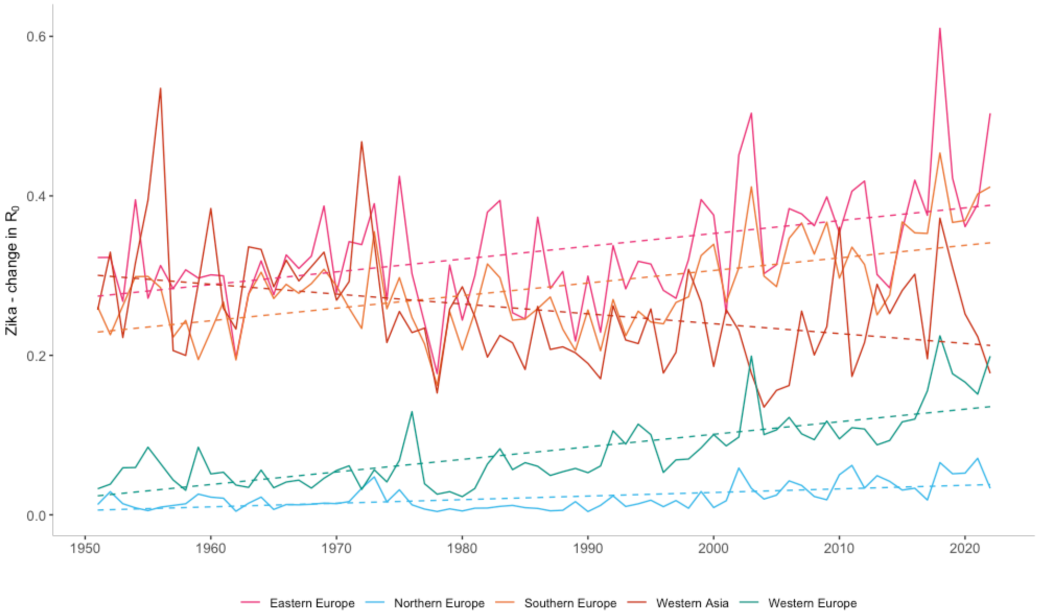

Figure 1.34 R0 for Zika by European regions between 1951 and 2022.

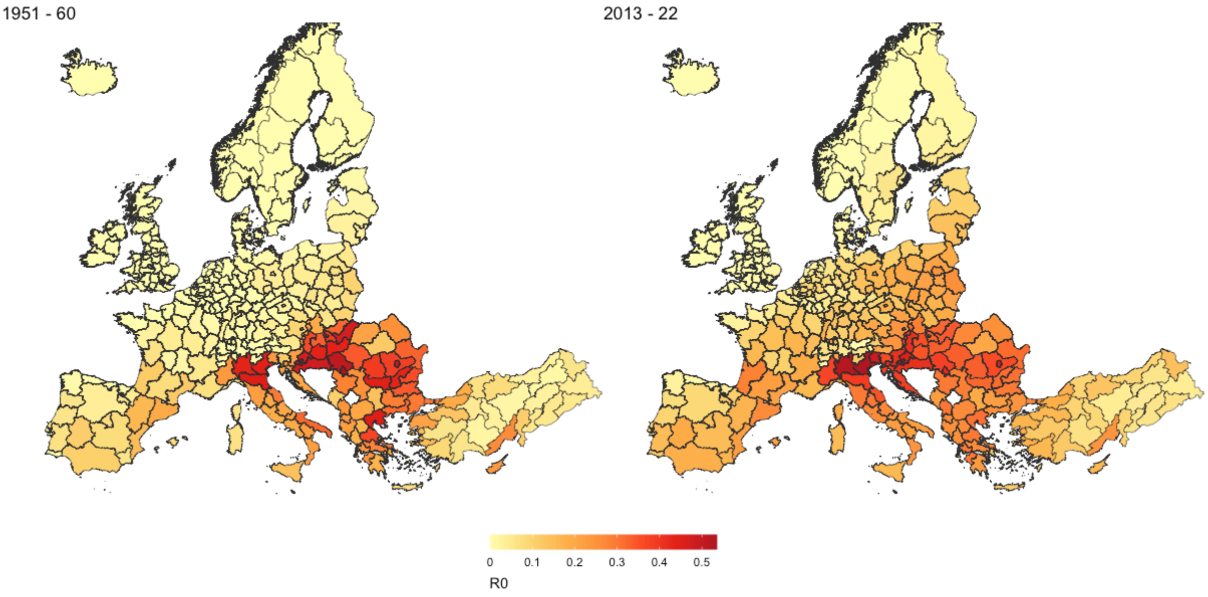

Figure 1.35 R0 by decade 1951-1960 vs 2011-2020 for Dengue by NUTS2 regions.

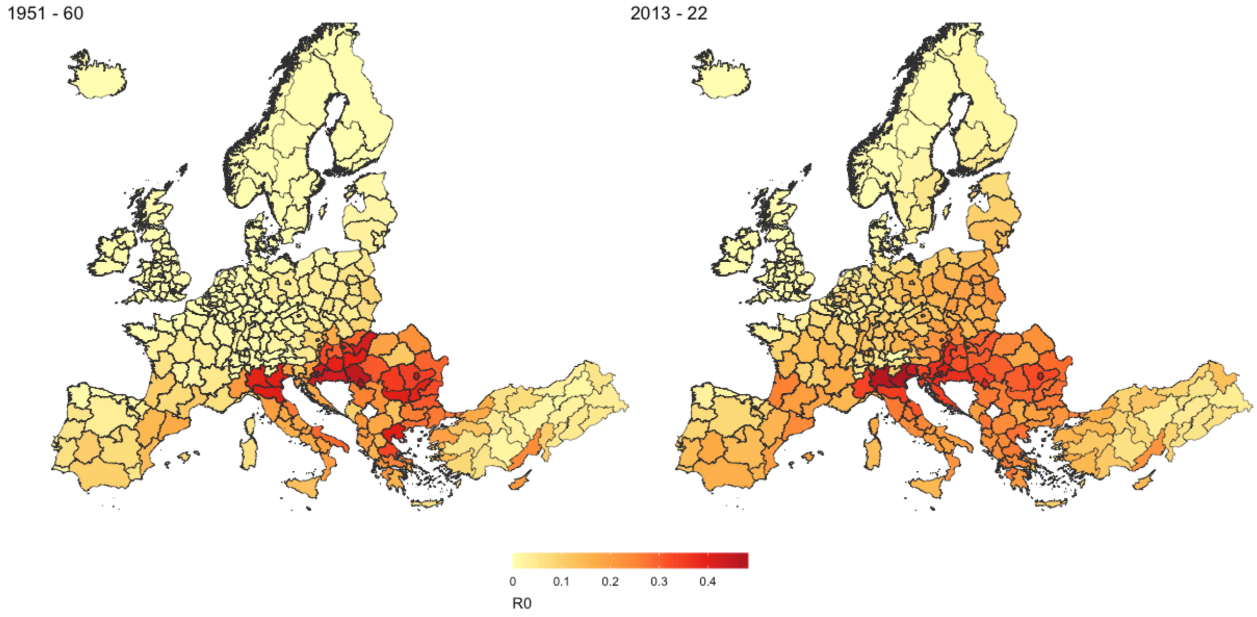

Figure 1.36 R0 by decade 1951-1960 vs 2013-2022 for Chikungunya by NUTS2 regions.

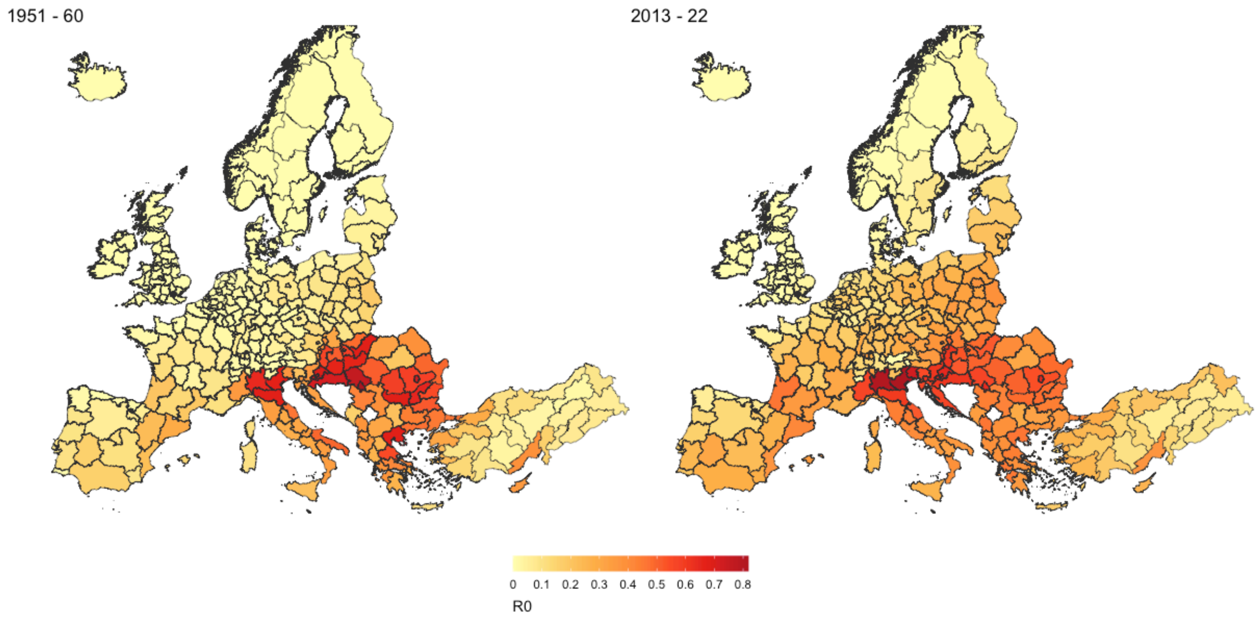

Figure 1.37 R0 by decade 1951-1960 vs 2013-2022 for Zika by NUTS2 regions.

Indicator 1.3.3: Climatic Suitability Dengue, Chikungunya and Zika

Part B (Mobility part)

Geographic coverage of Europe

For this indicator we included the European Environment Agency (EEA) member and cooperating countries plus the United Kingdom of Great Britain and Northern Ireland - excluding Kosovo (under UNSC resolution 1244) and Liechtenstein as data was not available for these countries.

Data

Refer to Methods and Data section of Indicator 1.3.3a: Climatic Suitability to Dengue, Chikungunya and Zika.

Methods

The average travel rate from source location i to destination location j is given by the radiation model

$$\langle U_{ij} \rangle = U_i \frac{n_i\, n_j}{(n_i + s_{ij})(n_i + n_j + s_{ij})}, \tag{1}$$

where n_i is the population size at the source location, n_j that at the destination location, and s_{ij} the population size that is encompassed by a circle around location i with a radius given by the distance between locations i and j.[91] With $U_i = n_i t_i u_i$, where t_i is the proportion of the population at location i that is travelling, and u_i the proportion of the population at location i that is infected, $\langle U_{ij} \rangle$ is the average travel rate of infected individuals from i to j, i.e., the number of infected individuals that travels between location i and location j per unit time. Here, we have used one year as unit time.

For every year in the range 1995 to 2019, the variables n_i, n_j, s_{ij}, t_j, and u_i was given by data or derived from data at a yearly temporal resolution (see section Data). Country level annual population data were extracted from World Bank. Country-level population sizes were used for any location outside of Europe. Within Europe, NUTS-3-level population sizes obtained from the eurostat data were used. Accordingly, for any travel into Europe, n_i was country-level population size, and n_j NUTS-3-level population size. As s_{ij} is the population size that is encompassed by a circle around location i with a radius given by the distance between locations i and j, it includes both country-level and NUTS-3-level population sizes. The average proportion of individuals u_i, at any point in time for a given year in source location i, that is infected within a phase such that viremia may set in during international travels, was derived from dengue-incidence data (individuals per 100000 per year) reported at the Global Burden of Disease.[7] Specifically, it was assumed that $u_i = \frac{v_i}{n_i}$, where the average number of infected individuals at any day within a year are given as $v_i = d_i \frac{k}{365} \frac{n_i}{100000}$. This formulation assumes that incidence rate (d_i) was uniformly distributed over any year where k represents sum of the expected infectious period (7-days) for dengue, and average time (in days) spent by tourists in Europe- a time-window plausible for viremia to set in during international visits. For European travellers travelling to endemic countries, the measure k is defined as

the minimum of the infectious period and the average visit duration of tourists to endemic countries whereas d_i should be read as the incidence rate of the travel destination (i.e. endemic countries).

The annual per country inbound duration times were obtained from the world tourism organization's (UNWTO) data. The international travel proportions together with the condition that $t_i > 0$ were estimated from UNWTO data for the years between 1995 to 2011. For the rest of the years (2012-2019), travel rates were linearly extrapolated country-wise. Note that the travel proportions don't represent the total individual travelling from location i to location j , rather they estimate total trips made from location i to location j.

To obtain a relevant measure of the mobility indicator that takes the environmental suitability for vectors and pathogens into account, the average travel rate of infected individuals from i to j was overlaid with the length of transmission season (LTS) at destination location j, denoted here by L_j. This means that the total number of infectious arrivals in any NUTS3 region were multiplied by the fraction of the year when the climatic suitability for mosquitos in that NUTS3-region was good, such that $R_0 > 1$, see equation (2). The LTS was derived in *Lancet* Countdown Europe Indicator: Climate Suitability Dengue, Chikungunya, Zika, and is measured in the unit of months per year. This overlay means, specifically, that the mobility considers (1) only NUTS-3 regions where L_j was above zero, and (2), that the monthly number of inbound infected individuals in any such NUTS-3-region j was multiplied with the corresponding $L_j > 0$.

$$M_{ij} = \langle U_{ij} \rangle L_j/12 \quad (2)$$

The resulting mobility measure is thus an estimation of the yearly number of arriving individuals, infectious at least some of the time in Europe, from source location i to any NUTS-3-region j where the environmental suitability is such that autochthonous transmission is plausible. This assumes that travels are uniformly distributed over time. From equation (2), we can derive the corresponding yearly total number of relevant infected inbound individuals to any NUTS-3-region j and write - where Ω denotes the set of dengue endemic countries and α , a scaling constant, was obtained simply by fitting the total travel-related dengue cases in our model to the ones reported by European centre for disease prevention and control (ECDC) between 2012 to 2019.

$$I_j = \alpha \sum_{i \in \Omega} M_{ij}, \quad (3)$$

Similarly, we can derive the yearly total number of relevant infected outbound individuals from location i and write-

$$O_i = \sum_{j \in \Psi} M_{ij}, \quad (5)$$

where Ψ is the set of NUTS-3 regions with environmentally suitable conditions for vectors and pathogens.
Code development and numerical computation were done in MATLAB 2020a.

Caveats

The radiation model is one of the most suitable mobility models to use as it is tractable, and it is essentially parameter free (only a scaling parameter). It has been shown to provide general and robust results.[91,92] The rate estimations are still based only on population-sizes, and do accordingly not account for travel processes dependent

on other aspects. Whereas some deviations from realizations naturally should be expected, the radiation model could still be expected to capture the important trends.

This analysis was data driven and based on data from across the world. One should note that any deficiencies in the applied data could affect the results computed and presented here. To our knowledge, however, the data applied here were of high quality.

Future form of the indicator

Future forms of the indicator could consider secondary or tertiary recursions, i.e., account for exportation within Europe from NUTS-3 regions where disease transmission arises due to primary importation pressures.

Additional analysis

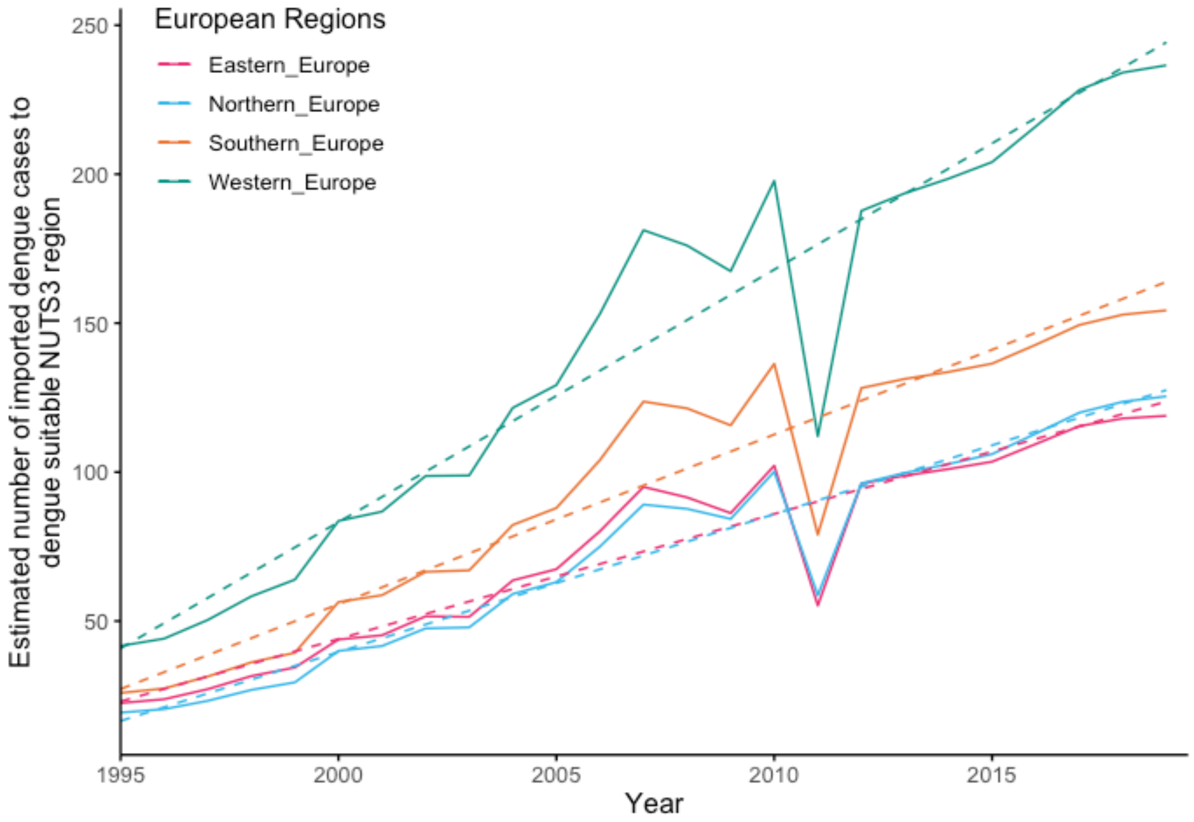

Figure Estimated numbers of total imported dengue cases to transmission suitable NUTS 3 locations of European subregions from dengue endemic-regions (1995 – 2019).

Indicator 1.3.4: Climatic suitability for Malaria

Geographic Coverage of Europe

This indicator includes the EEA-38 and the United Kingdom of Great Britain and Northern Ireland.

Data

Variable	Source	Frequency of update	Spatial resolution	Temporal range
Monthly 2-meter dew point temperature	ERA5-Land	Monthly	0.1°/ ~9 km	1950 to 2022
Monthly 2-meter temperature	ERA5-Land	Monthly	0.1°/ ~9 km	1950 to 2022
Monthly total precipitation	ERA5-Land	Monthly	0.1°/ ~9 km	1950 to 2022
Land cover	CORINE	Every 6 years	100 m	1990, 2000, 2006, 2012, and 2018
Altitude	European Environment Agency (EEA)	-	0.1°/ ~9km	-
Country level annual incidence rates	Worldbank	-	Country	2000 to 2019
Population size	Worldbank	-	Country	2000 to 2019

Methods

Europe has achieved malaria elimination in the early 1970s.[93] Although *P. vivax* has historically been the cause of autochthonous malaria cases in Europe, there have been reports of infections with *P. falciparum* in Germany and The Netherlands (Kingdom of the), without travel history.[94,95] As a result, we proposed to estimate the number of months suitable for transmission of *Plasmodium vivax* and *Plasmodium falciparum*, calculated from empirically derived thresholds of precipitation, temperature, and relative humidity.

Monthly climate information between 1951 and 2022 were obtained from the ERA5-Land repository with a 9km resolution. Land cover data were obtained from the CORINE repository, maintained by the Copernicus Land Monitoring Service, at 100m resolution (https://land.copernicus.eu/pan-european/corine-land-cover). Relative humidity was calculated with the August-Roche-Magnus equation using dew point temperature and temperature values.[96] Elevation data were obtained from the European Environment Agency (EEA) data repository at a 9km resolution (https://www.eea.europa.eu/data-and-maps/data/digital-elevation-model-of-europe).

Climatic suitability was defined as the coincidence of precipitation accumulation greater than 80 mm, average temperature between 14.5°C and 33°C for *P. vivax* and between 18°C and 32°C for *P. falciparum*, and relative humidity greater than 60%.[97,98] These combined values reflect the limits for potential transmission of each of the mentioned *Plasmodium* parasites.

The number of months with suitable climatic conditions was calculated at a 9 km resolution and later averaged to NUTS-3, NUTS-2, country, and region levels. Environmental suitability for *Anopheles* mosquitoes was defined

based on Benali et al., 2014, namely rice fields, permanently irrigated land, and sport and leisure facilities. The proportion of suitable area per ERA5-Land grid cell was included as weights in the computation of the summary values per NUTS district, country, and region (**Figure 1.38**).

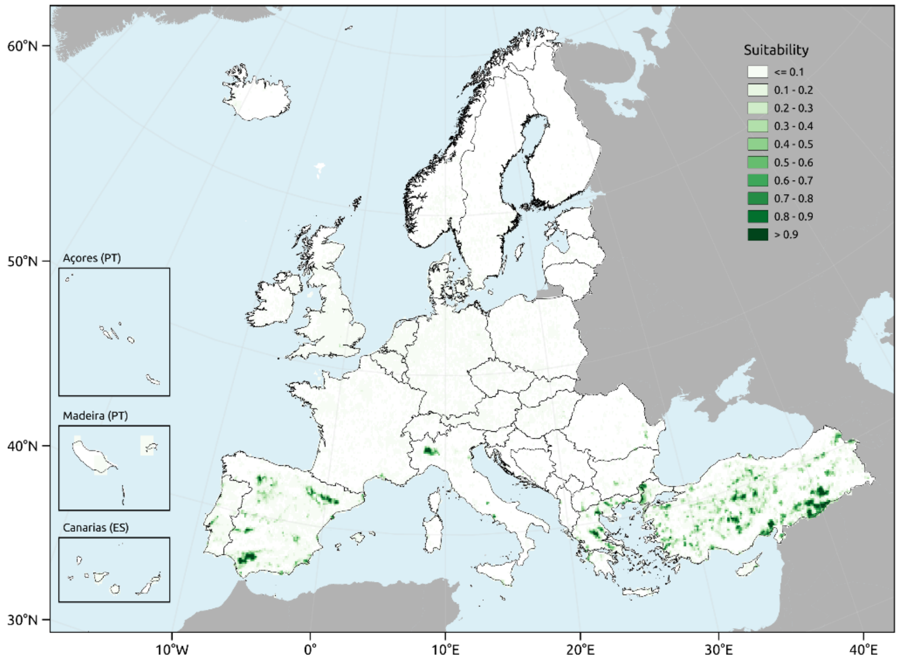

Figure 1.38 Environmental suitability weights. Weights computed using CORINE land cover classes suitable for Anopheles mosquitoes.

In addition, a radiation model,[91] based on the methodology developed in the second part of the Climatic Suitability to Dengue indicator (1.3.3), was employed to assess the risk of malaria importation from countries with endemic transmission. This model established connections between relevant source and destination locations, considering their population sizes and burdens of disease. Then, the number of months suitable for malaria transmission were used as a weighting factor in estimating the potential number of importation events for each malaria parasite at a NUTS3 level.

The code used to construct this indicator and create the visualizations included in this publication is available at the following repository: https://earth.bsc.es/gitlab/ghr/lcde-malaria.

Inequality Context

The role of climatic and environmental factors in mosquito activity and the spread of malaria parasites is well established. However, it is crucial to recognize that malaria is not solely driven by these factors but is also deeply intertwined with social inequalities. In endemic countries, the occurrence of malaria is frequently linked to low levels of urbanization, inadequate housing infrastructure, working in agriculture and residing in peri-urban and rural regions with limited access to healthcare services. Furthermore, areas characterized by lower socioeconomic conditions often exhibit a lack of knowledge regarding the disease, as well as of preventive strategies and services.[99]

Caveats

As this indicator balances accuracy and simplicity, it ignores the role of container breeding sites for *Anopheles* mosquitoes and considers only the role of precipitation for providing mosquito habitat. Additionally, this indicator reflects the state of conditions that would potentially allow malaria transmission to occur, had there not been public health efforts, socio-economic growth, and an increase in urbanisation levels. In this regard, the challenge relies on the correct communication of these results and emphasising the relevance of public health interventions that are currently preventing malaria from returning to the continent.

Additionally, this indicator considers land cover classes to be constant over time. *Anopheles* mosquitoes are susceptible to deforestation processes, as well as to changes in urbanisation, and have a high adaptation capacity.

Future Form of the Indicator

In the future, access to land cover change rates over the tracking period could help better estimate the average suitability by year. For example, replacement of forest areas with cities or crop fields would create a more realistic approximation to the suitability for disease transmission.

Additional analysis

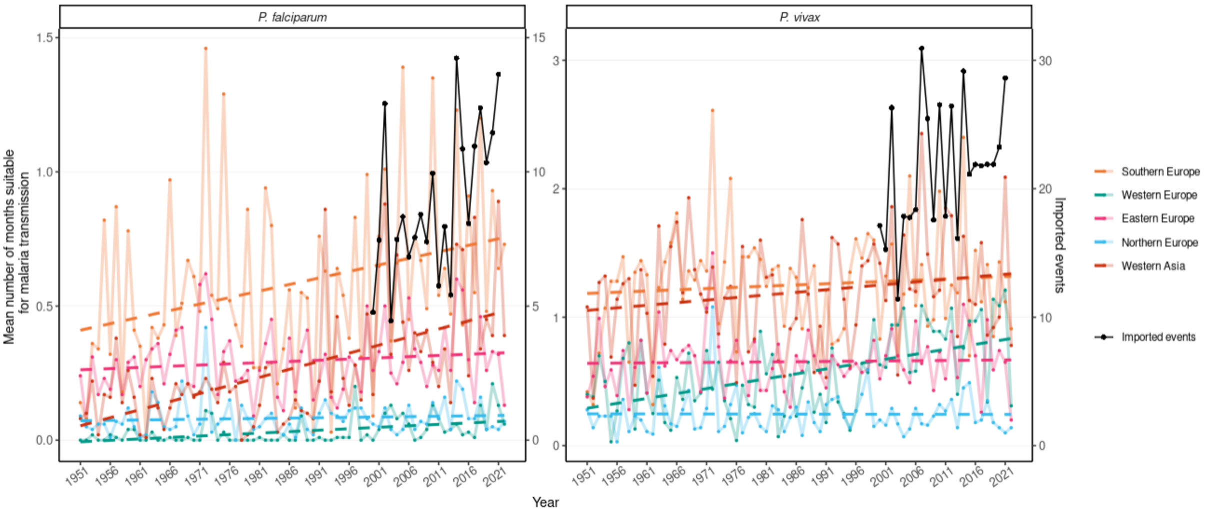

Figure 1.39 Mean number of months suitable for P. falciparum and P. vivax transmission between 1951 and 2022, grouped by European regions defined by the United Nations geo-scheme, and number of importation events from endemic regions to areas suitable for disease transmission. The trend estimation was performed using linear regression.

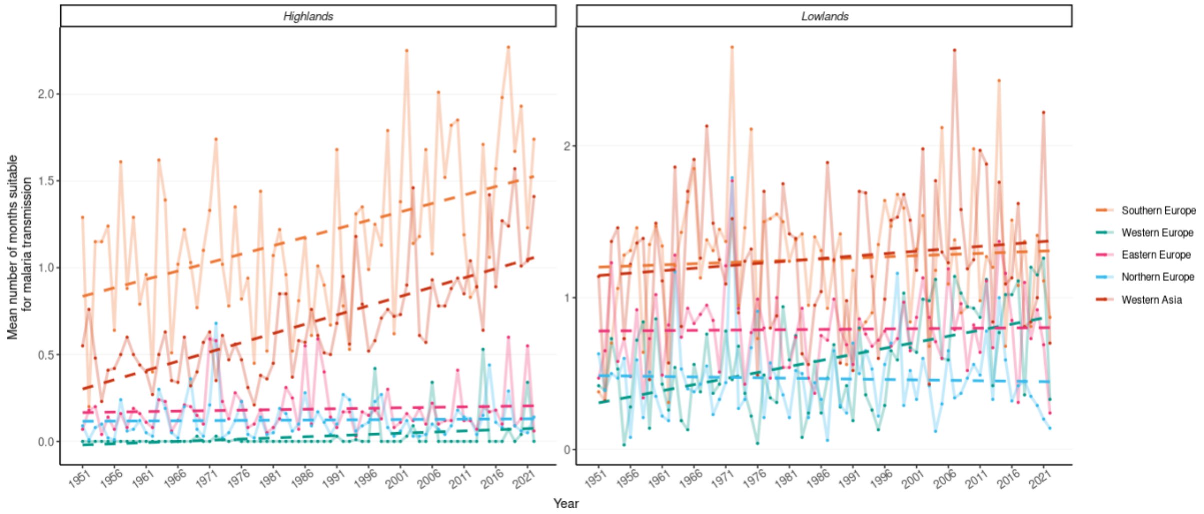

Figure 1.40 Mean number of months suitable for P. vivax transmission between 1951 and 2022, grouped by European regions defined by the United Nations geo-scheme, and stratified by high- and lowlands (highlands ≥ 700m). The trend estimation was performed using linear regression.

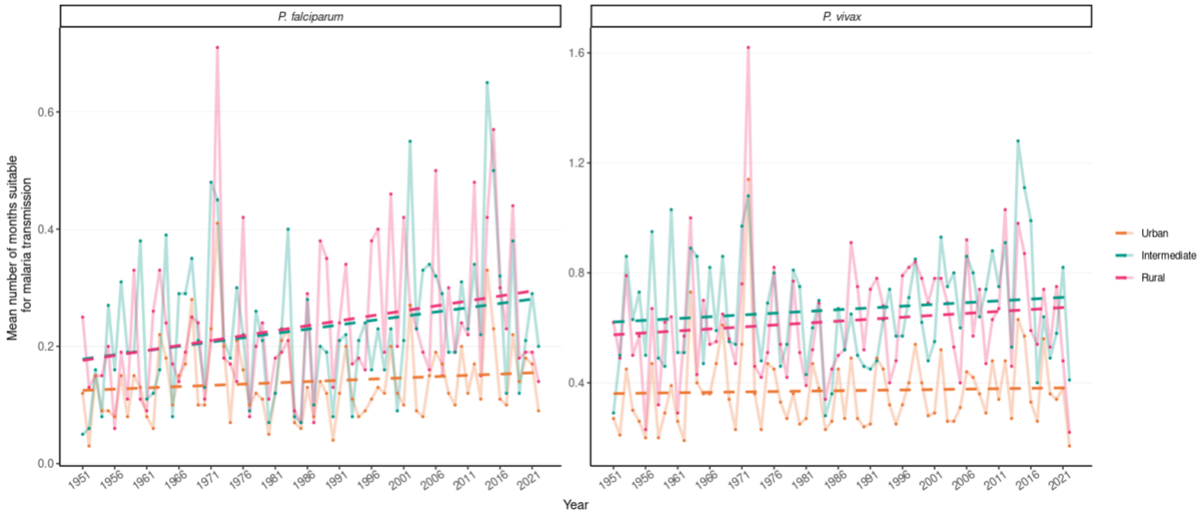

Figure 1.41 Mean number of months suitable for P. falciparum and P. vivax transmission between 1951 and 2022, grouped by urbanization level. The trend estimation was performed using linear regression.

Figure 1.42 Mean number of months suitable for P. falciparum and P. vivax transmission between 1951 and 2022, grouped by social deprivation level. The trend estimation was

performed using linear regression.

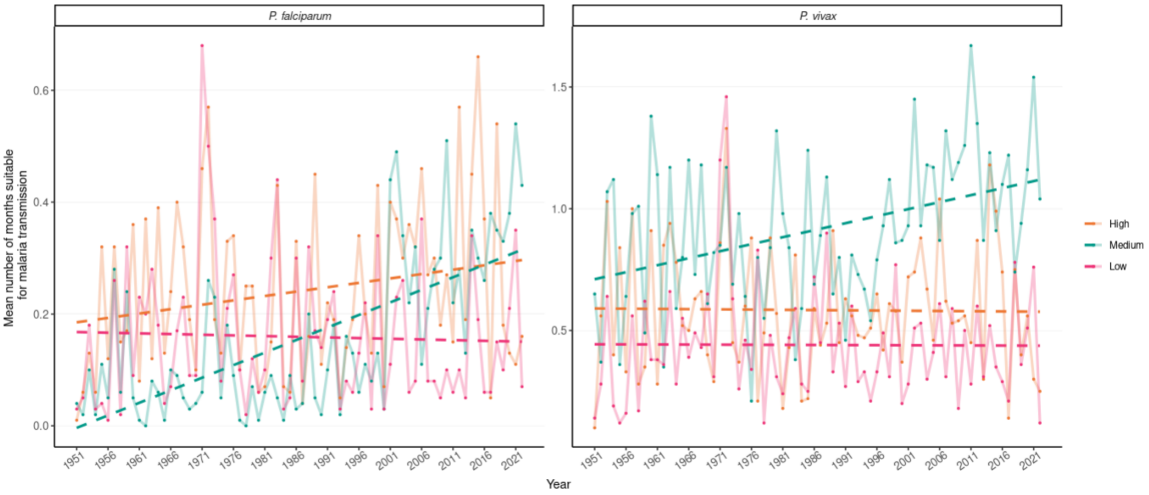

Figure 1.42 Mean number of months suitable for P. falciparum and P. vivax transmission between 1951 and 2022, grouped by social deprivation level. The trend estimation was performed using linear regression.

Table 1.7 Change in mean number of months per year suitable for P. falciparum and P. vivax transmission between 1951-1986 and 1987-2022 across European regions defined by the United Nations geo-scheme.

European region	*P. vivax*	*P. falciparum*
Eastern Europe	-2.54% (-0.03 month)	22.4% (0.12 month)
Northern Europe	64.7% (0.28 month)	318% (0.04 month)
Southern Europe	2.45% (0.002 month)	5.64% (0.02 month)
Western Europe	15.6% (0.17 month)	161% (0.24 month)
Western Asia	-2.24% (-0,006 month)	10.3% (0.008 month)

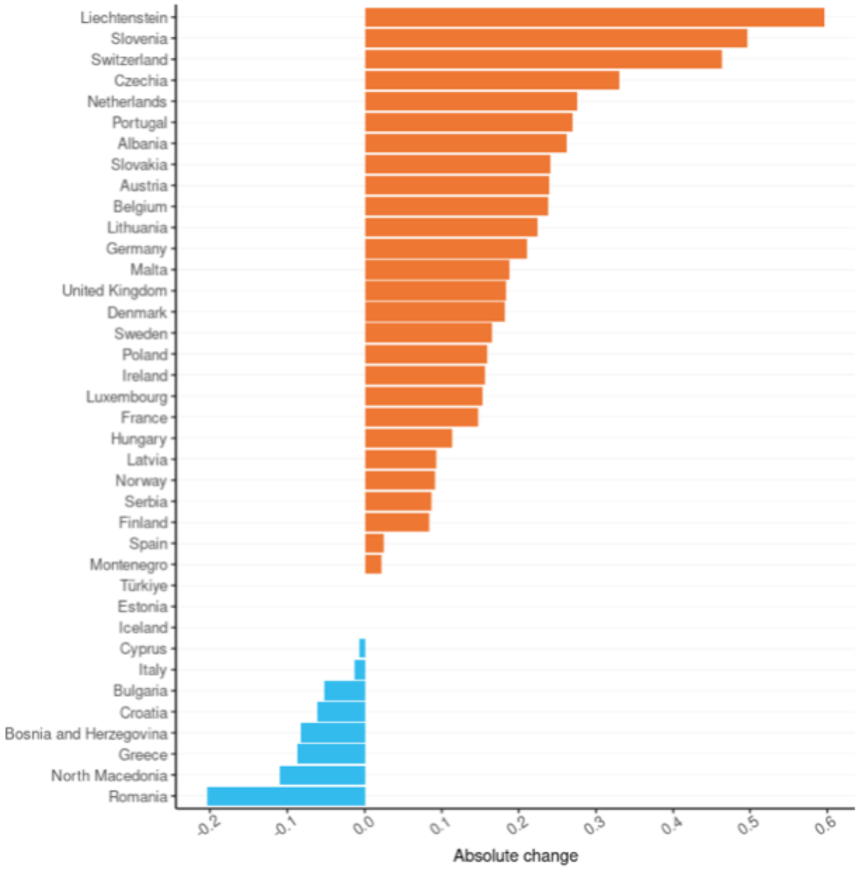

Figure 1.43 Absolute change in the mean number of months suitable for P. vivax transmission between 1951-1986 and 1987-2022 per country.

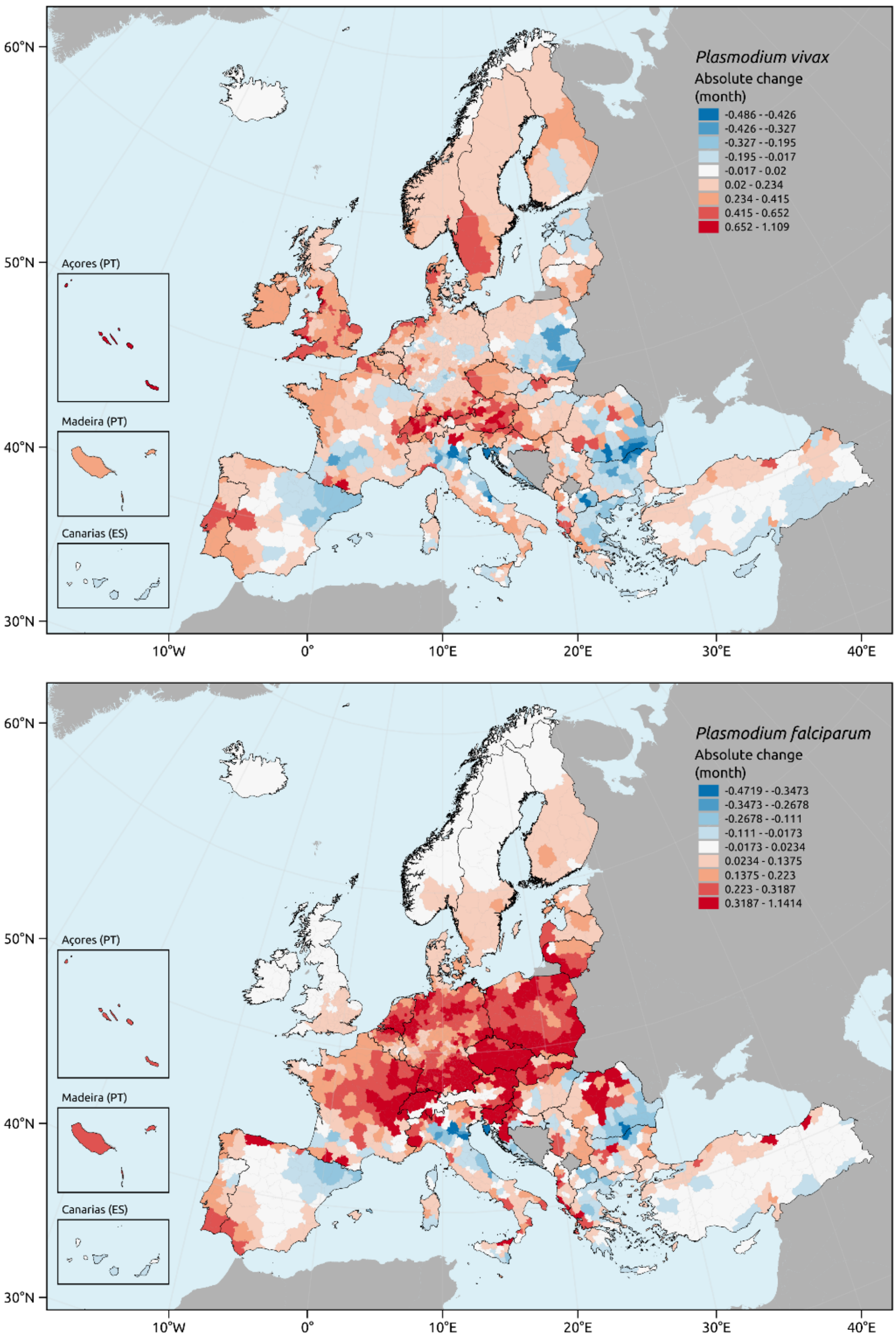

Figure 1.44 Change in mean number of months per year suitable for P. vivax (top) and P. falciparum (bottom) transmission between 1951-1986 and 1987-2022.

Table 1.8 Change in mean number of months per year suitable for P. falciparum and P. vivax transmission between 1951-1960 and 2013-2022 across European regions defined by the United Nations geo-scheme.

European region	*P. vivax*	*P. falciparum*
Eastern Europe	26.5% (0.27 month)	76% (0.22 month)
Northern Europe	75.2% (0.35 month)	462% (0.06 month)
Southern Europe	15.3% (0.09 month)	47.4% (0.11 month)
Western Europe	16.3% (0.17 month)	202% (0.34 month)
Western Asia	16.2% (0.03 month)	59% (0.04 month)

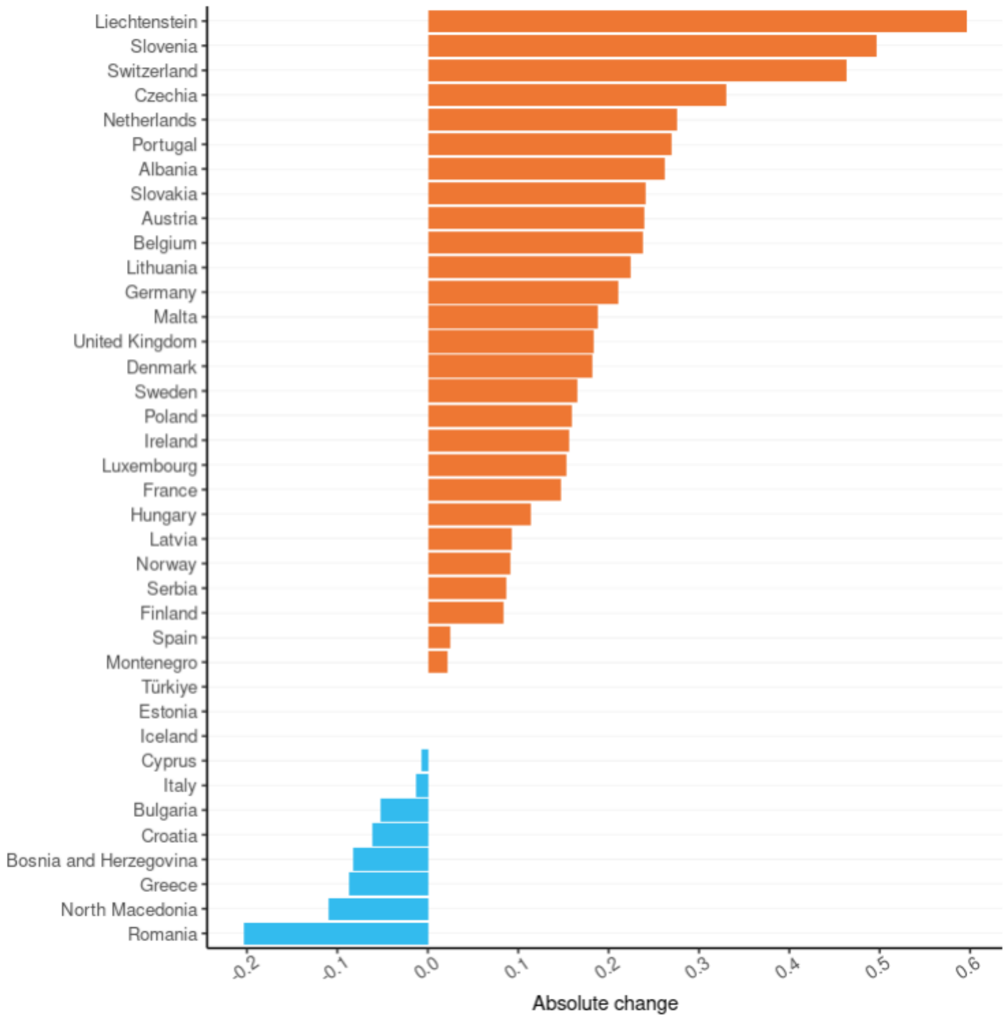

Figure 1.45 Absolute change in the mean number of months suitable for P. vivax transmission between 1951-1960 and 2013-2022 per country.

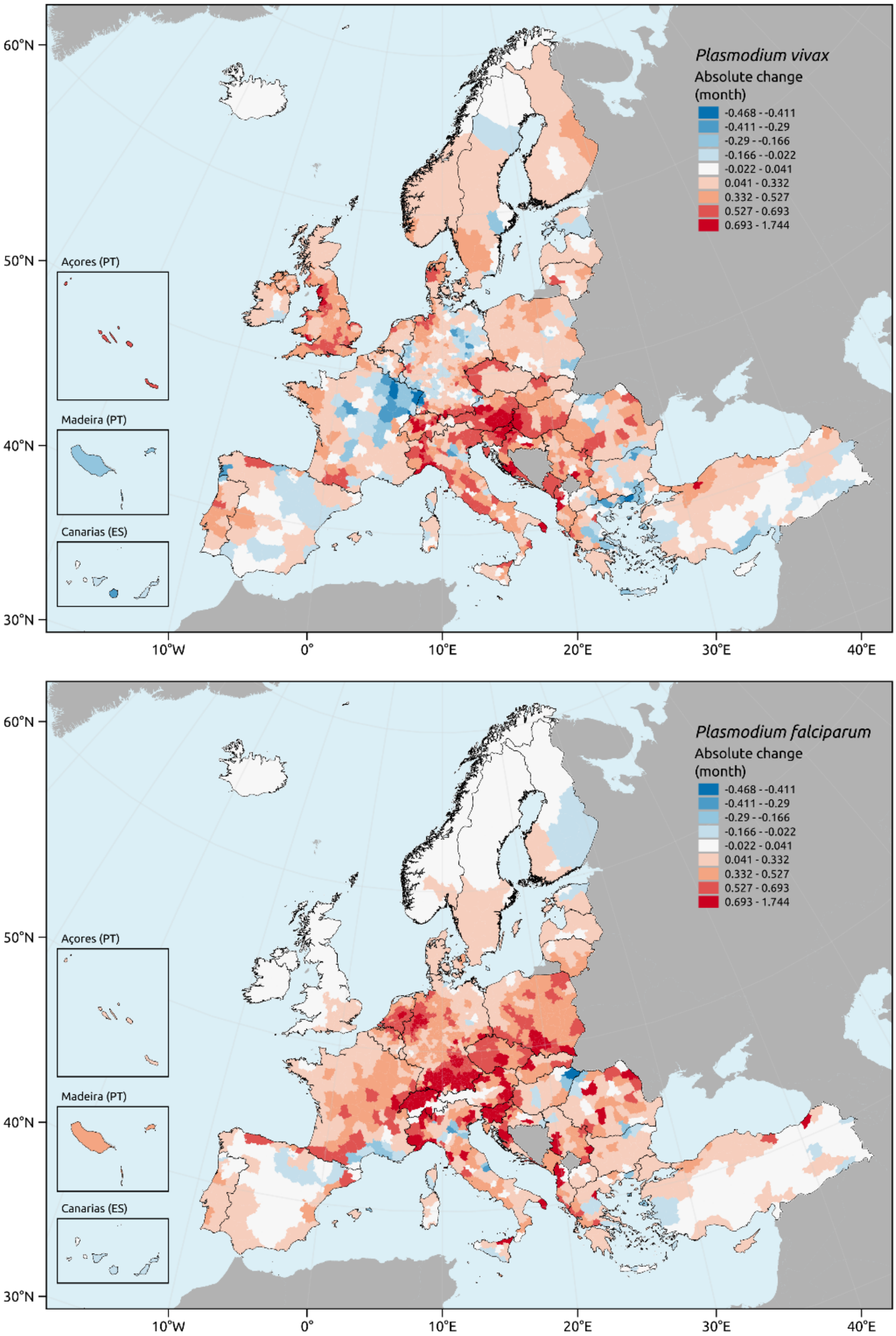

Figure 1.46 Change in mean number of months per year suitable for P. vivax (top) and P. falciparum (bottom) transmission between 1951-1960 and 2013-2022.

Indicator 1.3.5: Climatic suitability for Leishmaniasis

Geographic Coverage of Europe

All European countries listed in the current NUTS 2021 classification by EUROSTAT, which includes the 38 European Environment Agency member and cooperating countries in addition to the United Kingdom of Great Britain and Northern Ireland, Georgia, Republic of Moldova and Ukraine.

Data

Table 1.9 summarises the different data types, their original resolutions, sources, and if any transformation was applied before using them in the climatic suitability models.

Table 1.9 Summary of data features and sources.

Data type	Original spatial resolution	Transformed spatial resolution	Original temporal resolution	Transformed spatial resolution	Source
Leishmaniasis	NUTS3	-	Historical, 2009-2020	-	ECDC 2022, https://doi.org/10.2900/823484
Vectors	NUTS3	-	Historical, until 2022	-	ECDC VectorNet, https://www.ecdc.europa.eu/en/disease-vectors/surveillance-and-disease-data/phlebotomine-maps
Climate	0.1 x 0.1 degree	Averaged by NUTS3	Monthly, 2001-2020	Averaged for 2001-2010, 2011-2020	ERA5-Land, Copernicus, https://doi.org/10.24381/cds.68d2bb30
Land cover	100 x 100 meters	Percent coverage of five classes by NUTS3	2018	-	CORINE Land Cover, Copernicus, https://land.copernicus.eu/pan-european/corine-land-cover
Elevation	2.5 x 2.5 arc-minutes	Averaged by NUTS3	-	-	WorldClim (SRTM), https://www.worldclim.org/data/worldclim21.html

1. *Leishmaniasis data*

The indicator focuses on *Leishmania infantum*, the only autochthonous species causing both VL and CL in Europe. Because of the marked differences in disease reporting by European countries,[100] spatiotemporally aggregated data was used, representing presence/absence data for all NUTS3 regions for the period 2009-2020 (**Figure 1.47**). The data were obtained from a recent report published by the ECDC[101] describing the surveillance, prevention, and control measures implemented in 40 countries in Europe, northern Africa, the Middle East, Türkiye, and the Caucasus. Information was gathered through an extensive, non-systematic review of the scientific and grey literature published between 2009 and 2020, and through questionnaires addressing the public health and veterinary national authorities in the targeted countries.[101] To reduce the limitation of having both autochthonous

and imported cases in the dataset, occurrences from countries not listed as currently endemic by the WHO[102] and the ECDC[101] were removed from the dataset (records from Austria, Germany, and Hungary).

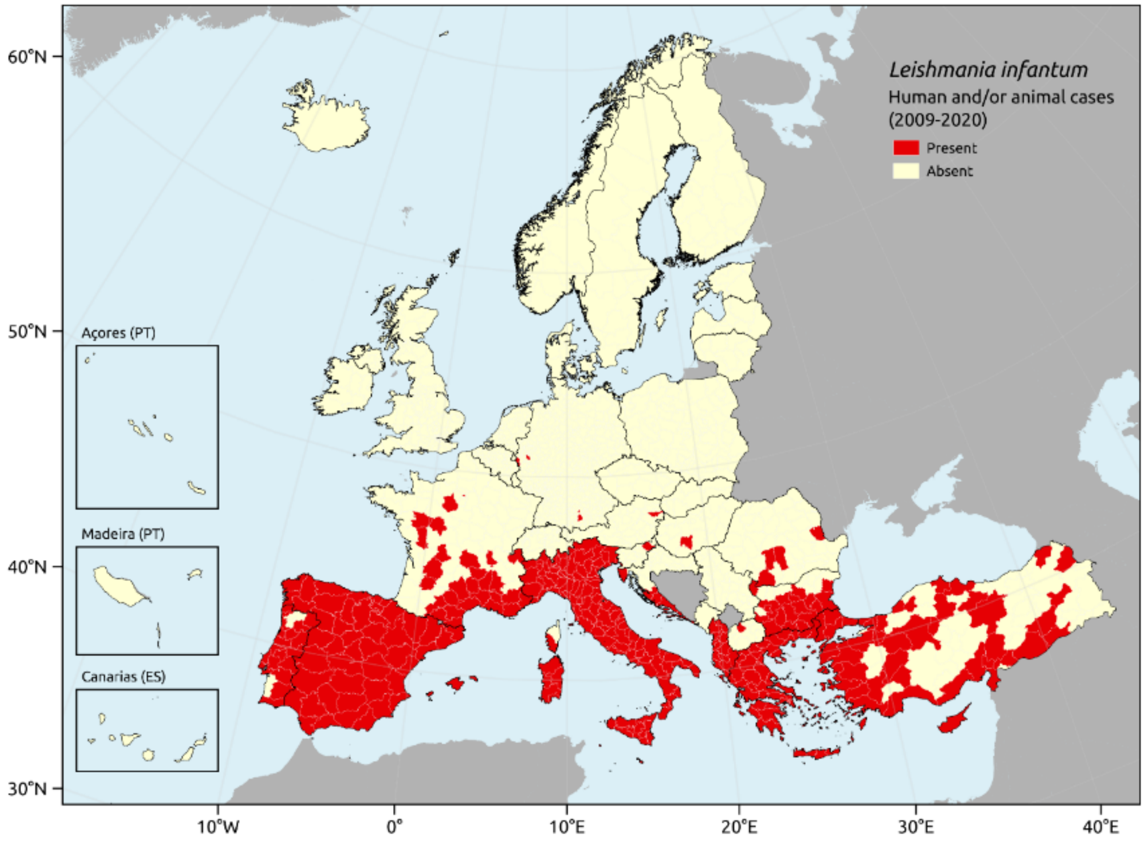

Data source: European Centre for Disease Prevention and Control (ECDC). Surveillance, prevention and control of leishmaniases in the European Union and its neighbouring countries. Stockholm: ECDC; 2022. doi: 10.2900/823484.

Figure 1.47 Geographical distribution of reported human and/or animal cases of leishmaniasis due to *Leishmania infantum*.

2. *Sand fly data*

Despite the occurrence of multiple sand fly species in Europe, only the main species related with *L. infantum* transmission in Europe were assessed, namely: *Phlebotomus perniciosus*, *P. ariasi*, *P. perfiliewi*, *P. neglectus*, and *P. tobbi*. Historical records by NUTS3 regions were obtained from ECDC's VectorNet project (**Table 1.10**). These distribution maps are based on published historical data and confirmed by experts from the respective countries. Species are classified as present, introduced, anticipated absent, or confirmed absent, which were reclassified as presence (present or introduced) or absence (anticipated or confirmed absent) by NUTS3 region (**Figure 1.48**)

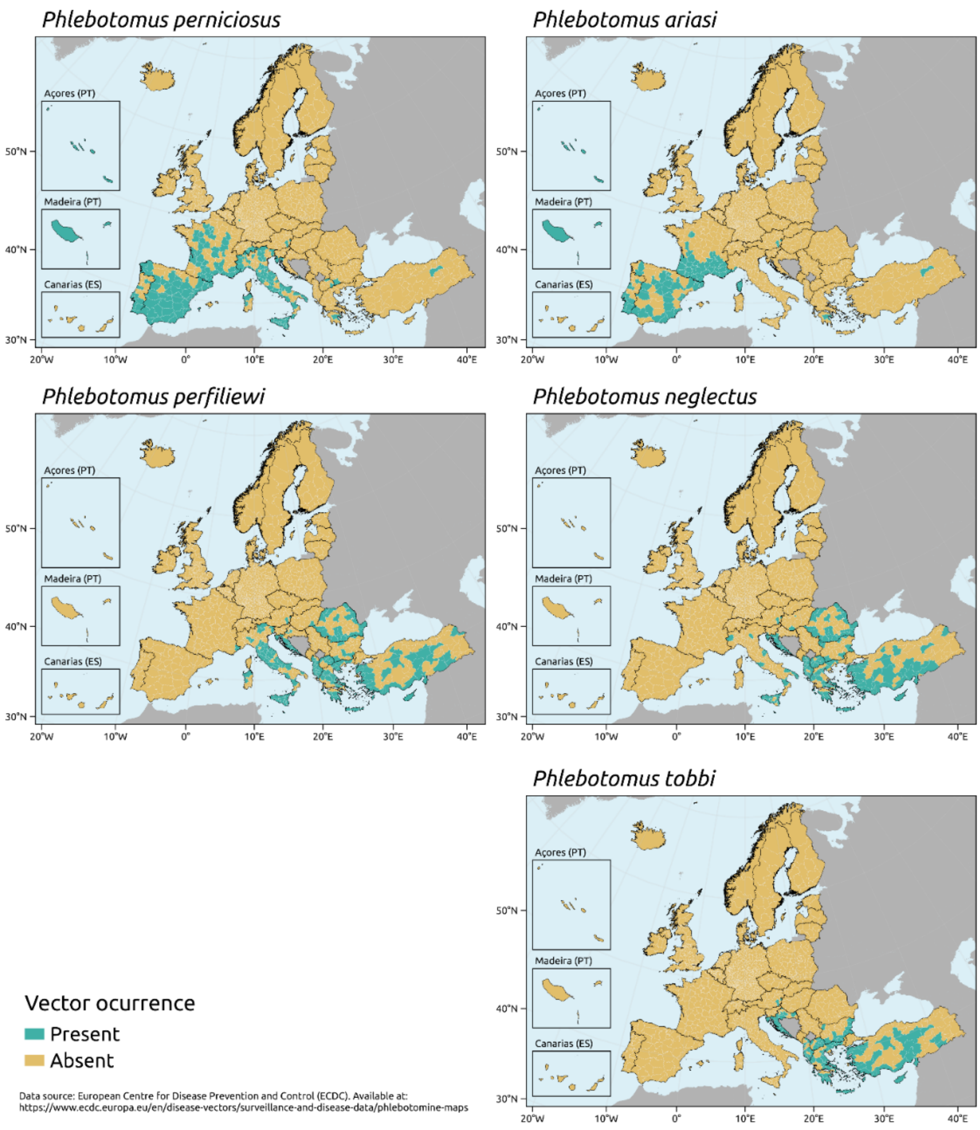

Figure 1.48 Geographical distribution of vectors of *Leishmania infantum*.

3. Climate data

A set of bioclimatic indicators was calculated from ERA5-Land reanalysis data. The 19 indicators cover bioclimatic variables for the land environment, characterising yearly averages and trends of temperature and precipitation.[103] An initial set of models was run with all bioclimatic indicators, and a final selection of indicators was obtained after checking their relative contributions to the models and removing those with high collinearity (Pearson's $r > 0.8$). The final selection had 9 variables, namely: annual mean temperature (BIO01), mean diurnal range of temperature (BIO02), isothermality (BIO03), temperature seasonality (BIO04), maximum temperature of the warmest month (BIO05), minimum temperature of coldest month (BIO06), precipitation of the wettest month (BIO13), precipitation of the driest month (BIO14), precipitation seasonality (BIO15).

To correctly match the time range of the leishmaniasis data and to assess climatic suitability in previous decades, the bioclimatic indicators were averaged over the periods 2011-2020 (for model fitting and predicting), and 2001-2010 (for predicting).

4. *Land cover and elevation data*

The percent coverage of different land cover classes was calculated by NUTS3 using data from the CORINE Land Cover project of the Copernicus land monitoring service (**Table 1.10**). The initial set of 44 land cover classes was reclassified into the five level 1 classes according to the product user manual: artificial surfaces, agricultural areas, forest and semi-natural areas, wetlands, and water bodies.

Elevation in meters above sea level was obtained from the WorldClim database (**Table 1.10**), which in turn was originated from the 90m digital elevation model by the NASA Shuttle Radar Topographic Mission (SRTM). Average values were calculated by NUTS3 regions.

Methods

A nested approach was applied to predict the climatic suitability for leishmaniasis. An initial set of models was fit for each sand fly vector species, using as covariates: bioclimatic indicators, land cover, and elevation. The outputs of the vector models (representing their climatic suitability) were then used as covariates in a second round of models, focused on predicting the climatic suitability for leishmaniasis, together with the selected bioclimatic indicators (**Figure 1.49**).

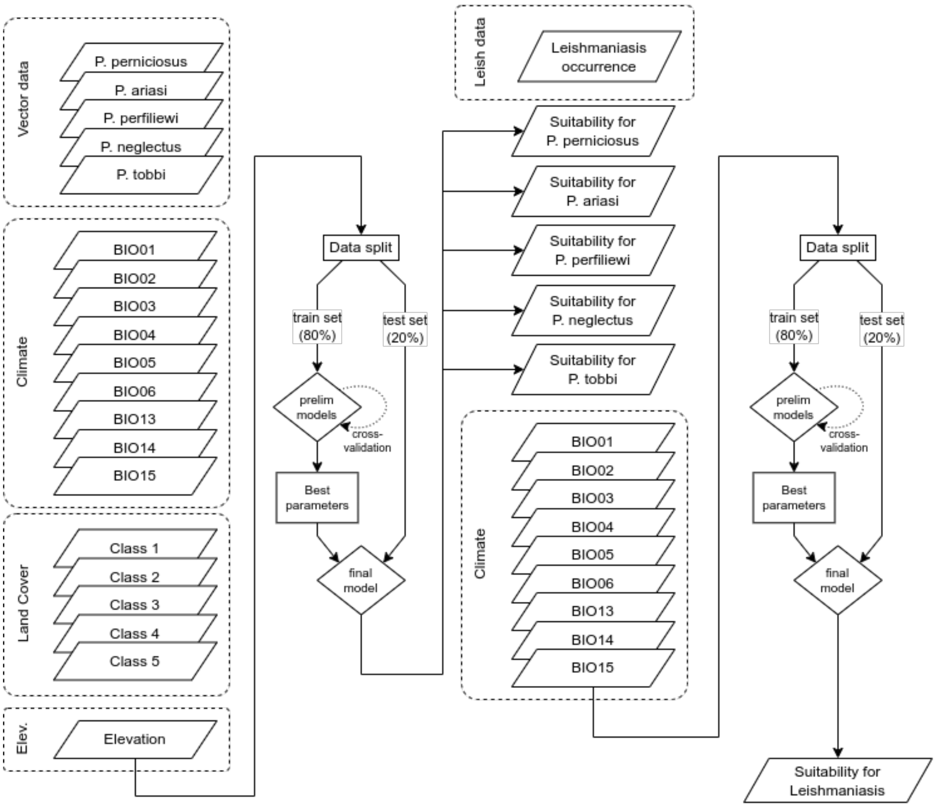

Figure 1.49 Scheme of the nested modelling framework.

The models were based on extreme gradient boosted regression (XGBoost), a machine learning algorithm that creates an ensemble of weak decision trees to form a stronger prediction model by iteratively learning from weak classifiers and adding them to a strong classifier (i.e., boosting). Gradient boosted regression is flexible in that it allows for non-linearity, both among features (i.e., interactions) and between features and predictions, collinearity between features, and non-random patterns of missing data.[104] XGBoost also includes a scale term for weighting unbalanced input datasets, and allows the use of regularisation parameters to prevent overfitting. Models were fitted using the R package *xgboost* version 1.7.5.1.[105] Model predictive performance was assessed by the area under the ROC curve method (AUC), applied to out-of-sample test sets, using the *pROC* R package.[106]

For each climatic suitability model, the input presence/absence dataset was randomly split into two sets, for model training (75%) and out-of-sample evaluation (25%). The model training set was applied in 10-fold cross-validation mode for 500 runs to estimate the best parameters for the final models. For each run of the cross-validation, multiple combinations of XGBoost parameters were tested. Final models were run using the best set of parameter values and the full training set, then further evaluated against the out-of-sample test set (**Figure 1.49**).

The code used to construct this indicator and create the visualizations included in this publication is available at the following repository: https://earth.bsc.es/gitlab/ghr/lcde-leishmaniasis.

Inequality Context

Risk factors of leishmaniases differ among European countries: VL is still primarily a paediatric disease in areas where poverty is prevalent, particularly in Eastern countries. Climate change can impact food insecurity, affecting populations of low socioeconomic status, and malnutrition is a known risk factor for development of VL, particularly in children. In Western European countries, the majority of VL cases occur in adults with co-morbidities (e.g., human immunodeficiency virus infection), in those undergoing immunosuppressive therapies, and in immunocompetent people lacking acquired immunity to the parasite.[101] Socially vulnerable populations include migrants and refugees, internally displaced and marginalised populations. Inequalities in access to healthcare, social and economic burdens and stigma associated with leishmaniasis, whilst not as pronounced as in endemic low-income countries, are to be expected in the more marginalised and lower socioeconomic populations of Europe.[101,107]

Caveats

One important limitation is not having disease data at sub-national levels on a yearly basis. Canine leishmaniasis data is also not included, and dogs are the main reservoirs of *L. infantum* and therefore considered good sentinels for human infection. This data is unlikely to be available at present, because leishmaniasis is not in the current EU list of notifiable diseases for humans and animals - hence the absence of data available from EU institutions (i.e. ECDC and EFSA). The most recent ECDC report, based on an extensive literature review and questionnaires sent to health authorities,[101] only includes subnational data for selected countries (Greece, Italy, France, Spain, Portugal and Türkiye), but they are aggregated in different multi-year periods. A consistent source of yearly disease counts is available by the WHO Global Health Observatory, but only at the national scale.[102]

Not being able to clearly distinguish between autochthonous and imported cases may limit the interpretation of climatic suitability for disease transmission. In the case of an imported human or animal case, disease transmission might have occurred under different climatic conditions. This limitation was reduced by removing the records from countries not currently considered as endemic by the WHO[102] and the ECDC.[101]

As leishmaniasis transmission is linked not just to climate, but also to specific socioeconomic profiles and human migration, not having these variables in the indicator is also a limitation.

Future Form of the Indicator

The indicator can be updated by projecting the current models into different decades, depending on the availability of the climate data. With the inclusion of climate projections, the suitability models can be further projected under climate change scenarios.

Analysis

Climatic suitability for leishmaniasis has increased in Europe in the last two decades, with more noticeable changes in countries of the Southern and Eastern European regions (**Figure 1.50**). The numbers and spatial distribution of NUTS3 regions predicted to be suitable have changed within each country, between the two decades (**Table 1.10; Figure 1.51**). Most NUTS3 regions predicted to be suitable in 2011-2020 are from countries currently endemic for leishmaniasis according to the WHO (2023) and the ECDC (2022), with some exceptions in Austria and Germany (**Figure 3C in main manuscript**).

The model outputs were further aggregated into the different categories of material deprivation. The material deprivation rate is an EU-SILC indicator that represents the inability to afford some items considered by most people to be desirable or even necessary to lead an adequate life. The indicator distinguishes between individuals who cannot afford a certain good or service, and those who do not have this good or service for another reason, e.g. because they do not want or do not need it.

The original predictions by NUTS3 were aggregated by NUTS2 regions to match the spatial resolution of the material deprivation data. Regions with higher material deprivation rates had notably higher climatic suitability for leishmaniasis, and there is an increasing trend when comparing the two periods (**Figure 1.53**). This reflects the known associations of leishmaniasis with poverty and precarious socioeconomic conditions.[101,107]

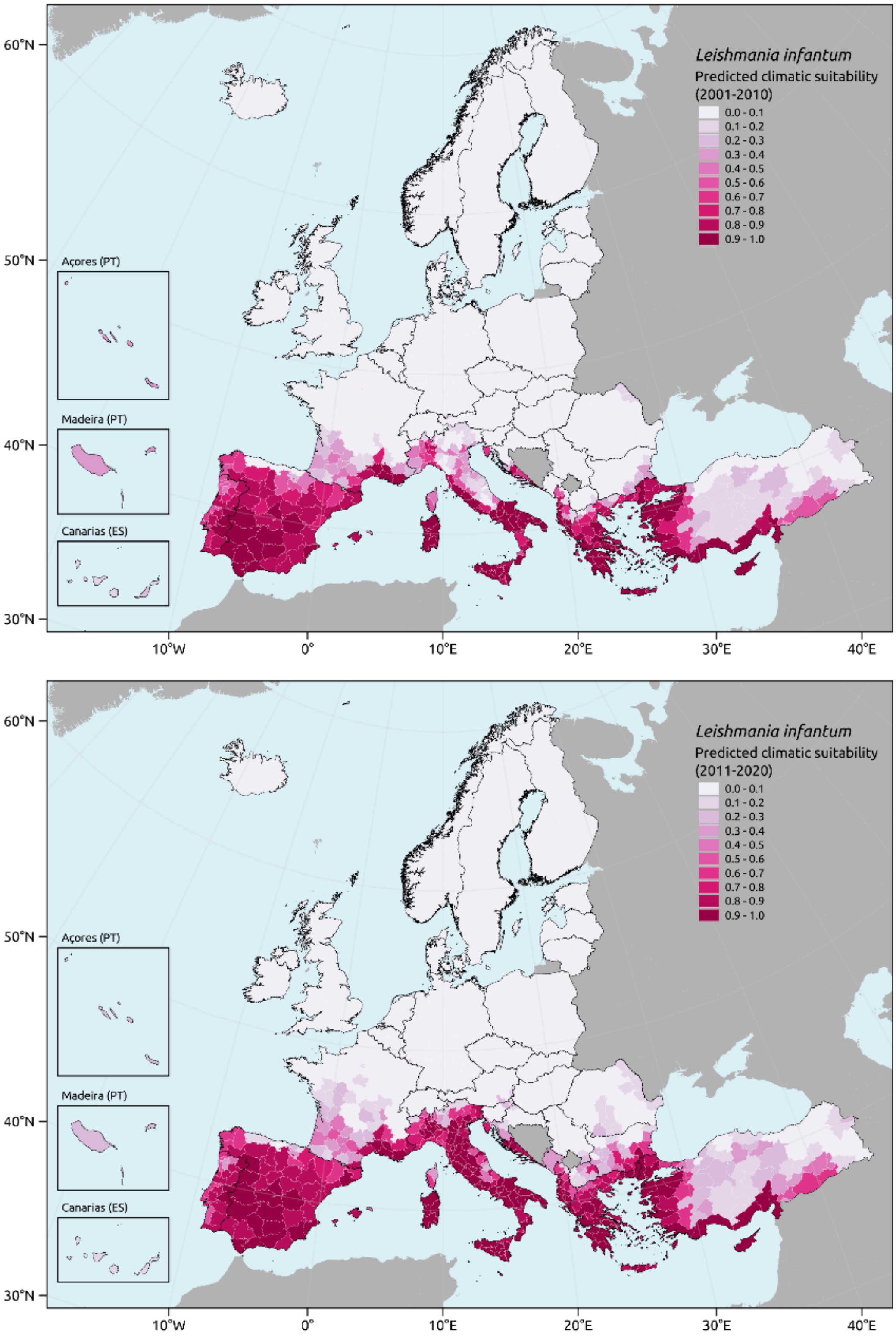

Figure 1.50 Predicted climatic suitability for leishmaniasis caused by *Leishmania infantum*. Darker colours represent higher climatic suitability by NUTS3 regions, in two decades: 2001-2010 (top), and 2011-2020 (bottom).

Table 1.10 Absolute number and percent change of NUTS3 regions suitable for leishmaniasis transmission by country in two periods: 2001-2010 and 2011-2020.

Country	NUTS3 regions suitable in 2001-2010	NUTS3 regions suitable in 2011-2020	Total number of NUTS3 regions	Percent change between periods
Albania	11	12	12	8.3
Austria	0	2	35	5.7
Bulgaria	5	17	28	42.9
Croatia	5	7	21	9.5
Cyprus	1	1	1	0.0
France	27	42	101	14.9
Germany	0	2	401	0.5
Greece	50	52	52	3.8
Italy	87	106	107	17.8
Malta	2	2	2	0.0
Montenegro	0	1	1	100.0
North Macedonia	1	6	8	62.5
Portugal	25	25	25	0.0
Romania	0	4	42	9.5
Serbia	0	1	25	4.0
Slovenia	0	3	12	25.0
Spain	54	56	59	3.4
Türkiye	52	56	81	4.9

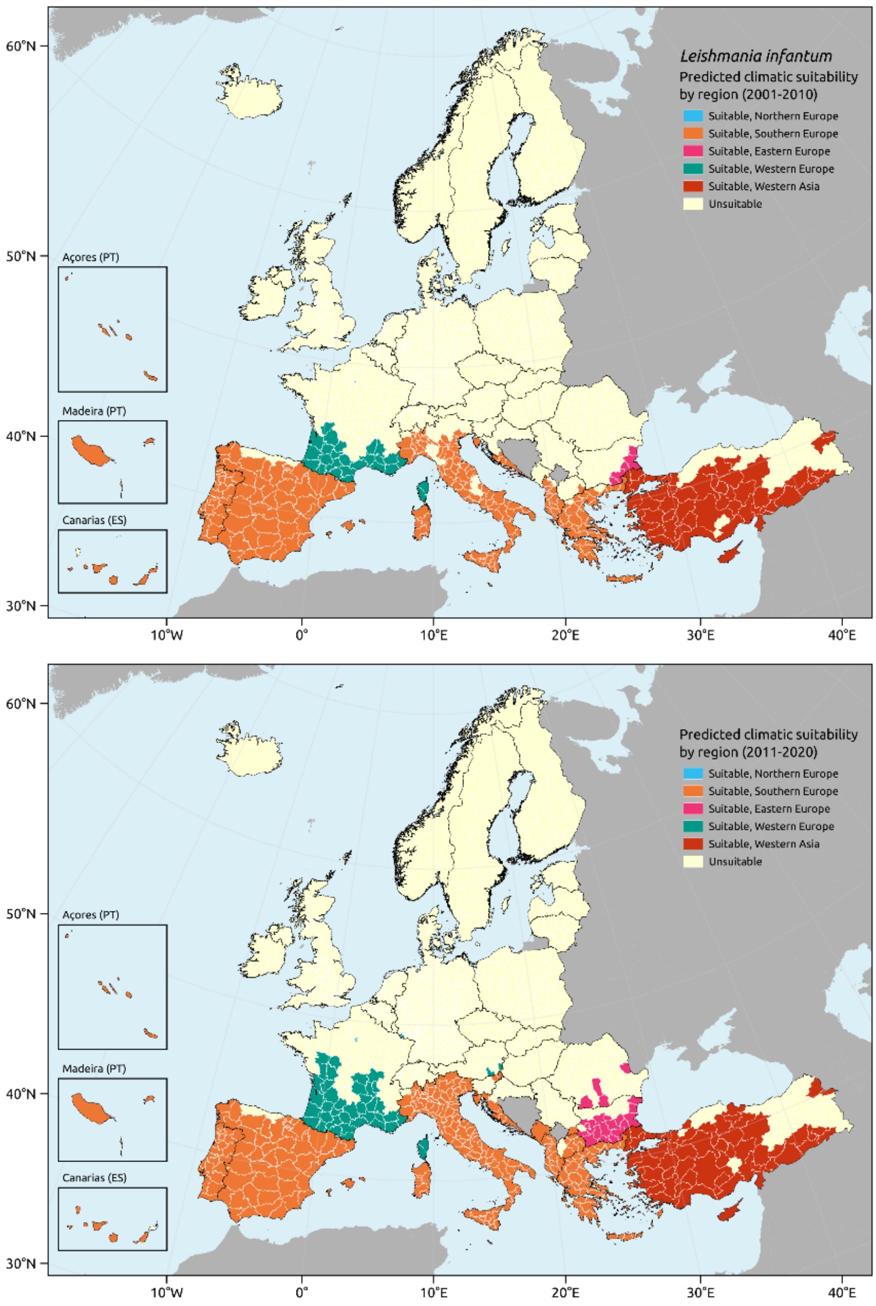

Figure 1.51 Predicted climatic suitability for leishmaniasis caused by *Leishmania infantum*. NUTS3 regions are classified as being suitable or unsuitable by European region, in two decades: 2011-2020 (top), and 2001-2010 (bottom).

The number of suitable NUTS3 regions has increased in the last decades in Southern, Western, and Eastern Europe, and in Western Asia, while remaining absent from Northern Europe (**Figure 1.52**). These numbers have increased in Italy, Spain, Türkiye, Greece, France, Albania, Croatia, Bulgaria, and North Macedonia (**Figure 1.53**). Slovenia, Serbia, Romania, Montenegro, Germany, and Austria only had suitable NUTS3 regions in the period 2011-2020 (**Figure 1.53**).

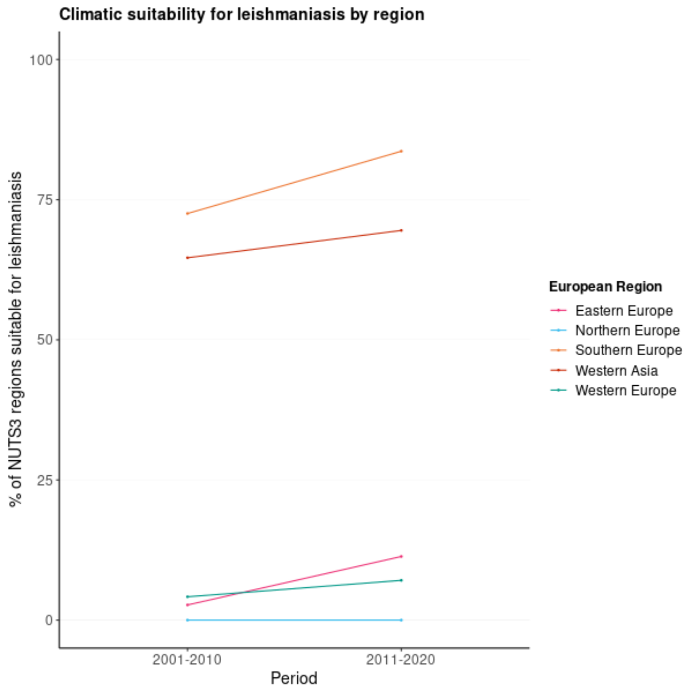

Figure 1.52 Percentage of NUTS3 regions suitable for leishmaniasis transmission by European region, in two periods: 2001-2010 and 2011-2020.

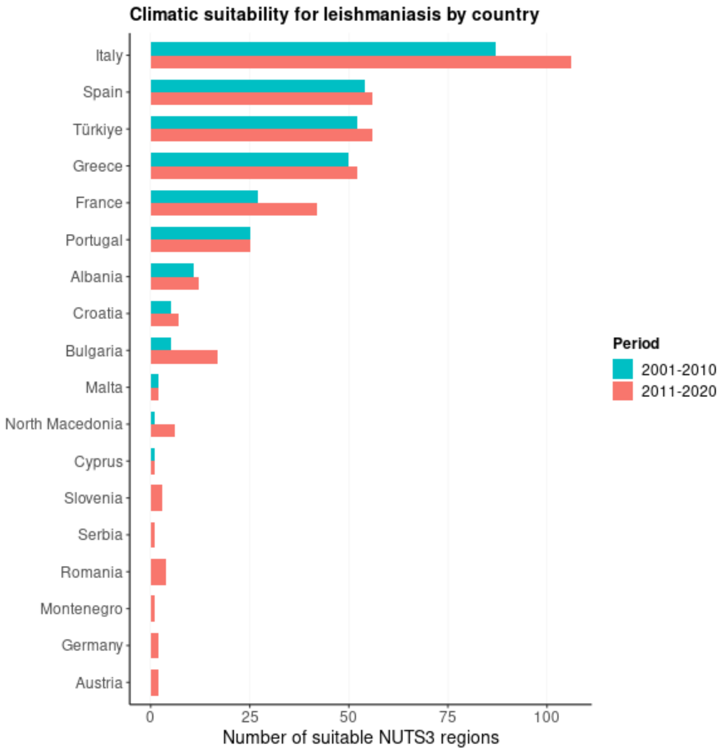

Figure 1.53 Number of NUTS3 regions suitable for leishmaniasis transmission by country, in two periods: 2001-2010 and 2011-2020

Indicator 1.3.6: Climatic suitability for ticks

Geographic Coverage of Europe

For this indicator we included the European Environment Agency (EEA) member and cooperating countries plus the United Kingdom of Great Britain and Northern Ireland.

Data

Variable	Source	Frequency of update	Spatial resolution	Temporal range
Monthly 2-meter dew point temperature	ERA5-Land	Monthly	0.1°/ ~9 km	1950 to 2022
Monthly 2-meter temperature	ERA5-Land	Monthly	0.1°/ ~9 km	1950 to 2022
Land cover	CORINE	Every 6 years	100 m	1990, 2000, 2006, 2012, and 2018
Ixodes ricinus presence	GBIF	-	Point data	2012 to 2018

Methods

Tick-borne diseases (TBDs) are the most common vector-borne illnesses in the Northern Hemisphere.[108] Among them, Lyme disease (LD) stands out as the most frequent in Europe, with Western Europe reporting 200,000 cases per year.[109] Another prevalent TBD is tick-borne encephalitis (TBE), caused by the TBE-virus from the Flaviviridae family.[110] The frequency of LD and TBE, as well as the range of their primary vector, *Ixodes ricinus* ticks, have been steadily increasing over the past few decades.[111,112,113] Moreover, there is compelling evidence suggesting the expansion of tick species of public health relevance towards higher altitudes and latitudes.[113] Ticks, being ectotherm parasites, heavily rely on environmental conditions for feeding, development, and breeding.[114] Optimal climatic conditions for nymph activity have been suggested as temperatures between 10ºC and 26ºC, coupled with relative humidity above 45%.[115]

The number of months with optimal climatic conditions for *I. ricinus* nymphs' feeding activity was estimated based on empirically derived thresholds of temperature and relative humidity. These combined values reflect the limits for potential exposure to questing *I. ricinus* ticks.

Data from the ERA5-Land repository were used, namely monthly 2-meter temperature and dew point temperature between 1951 and 2022 with a resolution of 9 km.[24] The calculation of relative humidity employed the August-Roche-Magnus equation, incorporating dew point temperature and temperature values.[96] In addition, suitable land cover classes were included as weights in the computation of summary statistics. Environmental suitability was derived from reports of *Ixodes ricinus* presence in Europe from 2012 to 2018, covering the period between the latest land cover classification and its predecessor. Land cover classes were sourced from the CORINE repository, which is maintained by the Copernicus Land Monitoring Service and had a resolution of 100m (https://land.copernicus.eu/pan-european/corine-land-cover). Based on observations published in the Global Biodiversity Information Facility (GBIF) repository, 82.4% of the ticks were found in discontinuous urban landscapes, moors and heathlands, non-irrigated croplands, as well as coniferous and broad-leaf forests (**Figure 1.54**).[116]

The code used to construct this indicator and create the visualizations included in this publication is available at the following repository: https://earth.bsc.es/gitlab/ghr/lcde-ticks.

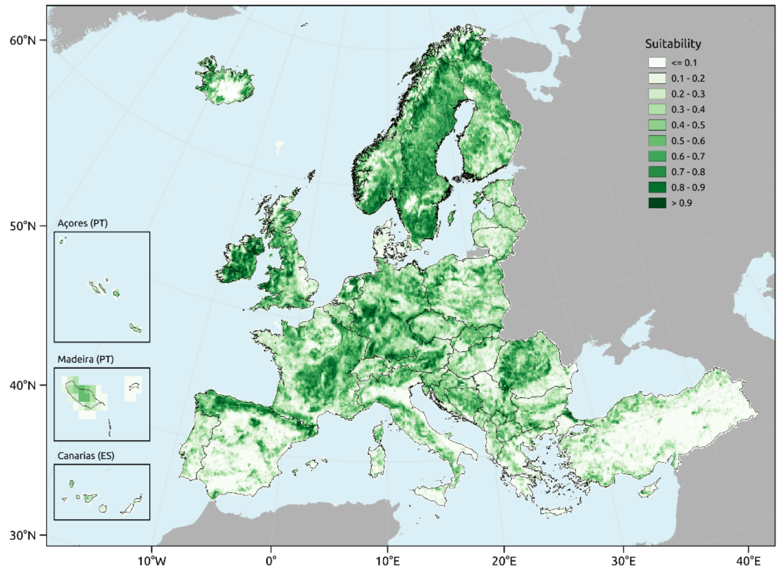

Figure 1.54 Environmental suitability for *Ixodes ricinus* ticks.

Subsequently, the number of months with suitable climatic and environmental conditions were computed at a resolution of 9 km and later averaged to NUTS-3, NUTS-2, country, and region, encompassing the EEA-38 countries.

Inequality Context

The distribution and habitats of *Ixodes ricinus* ticks are associated with the presence of social inequalities regarding the exposure to and prevention of TBDs. Occupational exposure remains a significant source of contact with these ticks in natural environments, as well as residing in proximity to forested areas. These disparities result in differential knowledge of prevention strategies and access to treatment options.

Certain populations, particularly those engaged in agriculture or residing in peri-urban and rural areas, may face challenges in accessing healthcare services. The situation can be exacerbated in the case of TBE, a disease that can be prevented through vaccination. Despite the availability of information and vaccination centres, vulnerable individuals may still encounter difficulties in obtaining necessary healthcare.

Caveats

This indicator is a simplified representation of the complex dynamics observed in *I. ricinus*. While suitable climatic conditions play a relevant role, the availability of intermediate hosts, such as rodents, birds, and roe deer, also influences tick populations. Although land suitability considers host availability indirectly, it does not explicitly include their ecological requirements. In addition, land suitability assumes a constant land use classification throughout the time series, disregarding the changes in forest distribution and agricultural areas that have occurred in Europe since the 1950s.

This indicator should be interpreted as a measure of hazard rather than risk because it did not incorporate actual cases of TBDs. Due to the complexity of TBD systems, the suitability of climatic and environmental conditions may not directly correspond to the occurrence of cases and may not align perfectly with surveillance records.

Future Form of the Indicator

Future forms of the indicator would consider NDVI thresholds as a proxy for land use. In addition, statistical models could use surveillance records of the most frequent TBDs with mandatory notification, such as TBE for validation or bias correction of the indicator.

Additional analysis

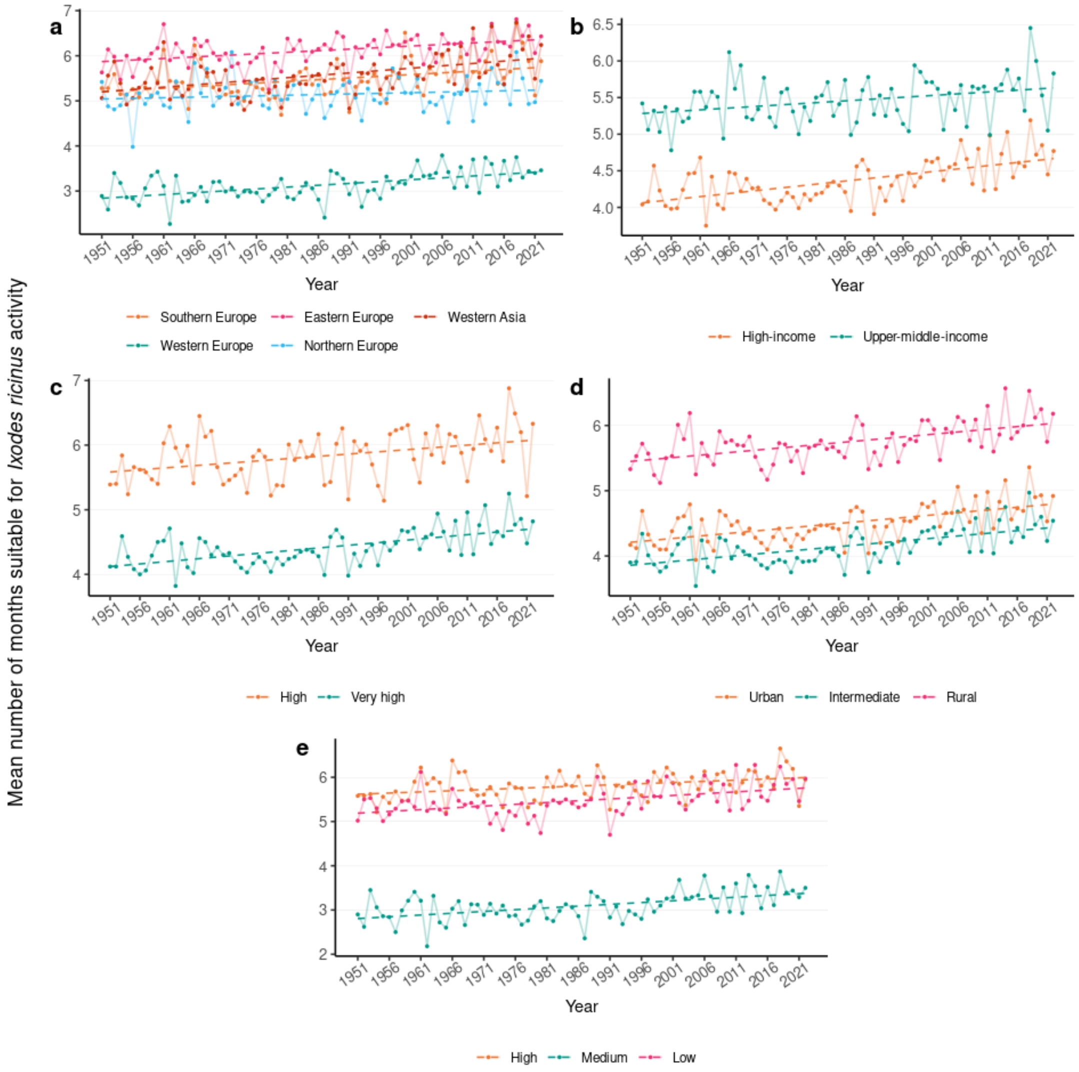

Figure 1.55 Mean number of months with optimal conditions for Ixodes ricinus nymphs' feeding activity between 1951 and 2022, grouped by a) European regions defined by the United Nations geo-scheme, b) income level, c) Human Development Index (HDI), d) level of urbanization, and e) level of social deprivation. The trend estimation was performed using linear regression.

Change in the number of months suitable for tick activity between 1951-1986 and 1987-2022.

Table 1.11 Change in mean number of months with optimal conditions for Ixodes ricinus nymphs' feeding activity between 1951-1986 and 1987-2022 across European regions defined by the United Nations geo-scheme.

European region	Percentage change (Absolute change)
Eastern Europe	4.90% (0.26 month)
Northern Europe	10.2% (0.31 month)
Southern Europe	4.64% (0.28 month)
Western Europe	7.37% (0.4 month)
Western Asia	0.79% (0.04 month)

Table 1.12 Change in mean number of months with optimal conditions for Ixodes ricinus nymphs' feeding activity between 1951-1986 and 1987-2022 between level of social deprivation.

Level of social deprivation	Percentage change (Absolute change)
High	2.86% (0.16 month)
Medium	6.22% (0.33 month)
Low	9.79% (0.29 month)

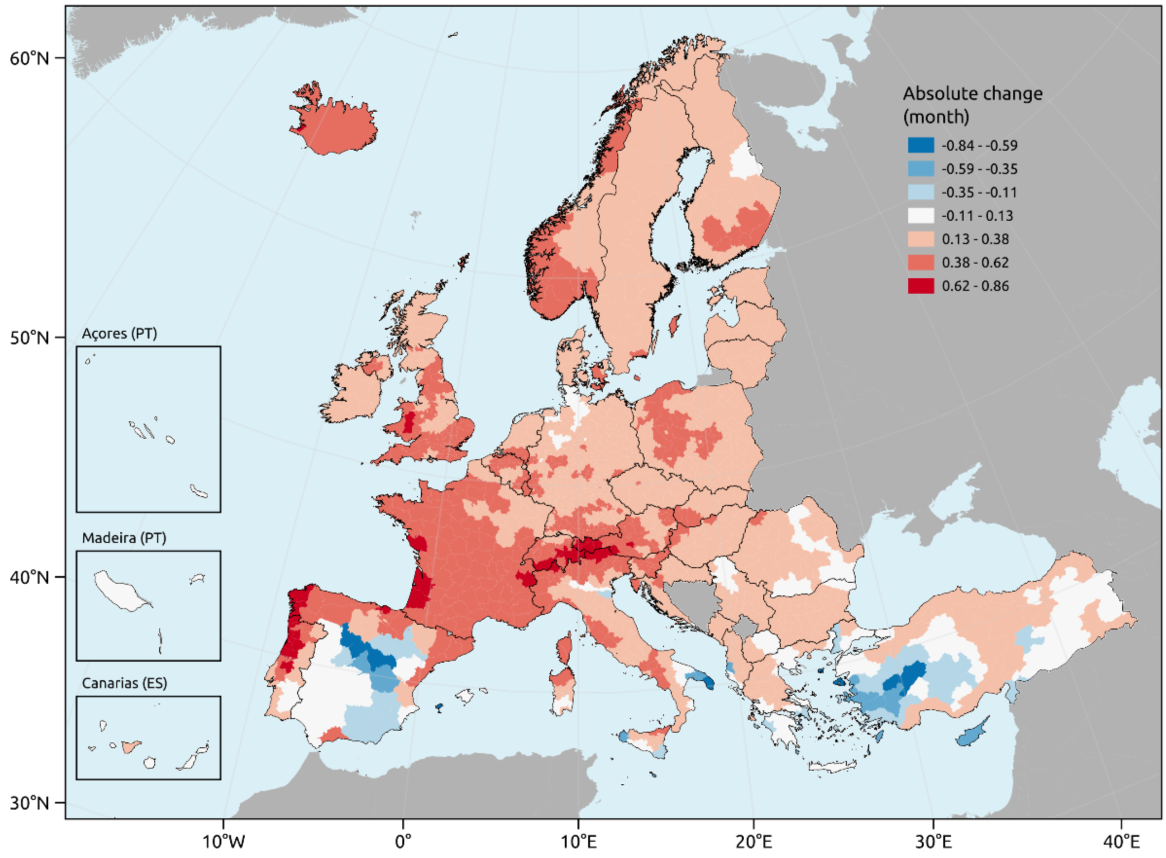

Figure 1.56 Absolute change in the mean number of months with optimal conditions for Ixodes ricinus nymphs' feeding activity between 1951-1986 and 1987-2022.

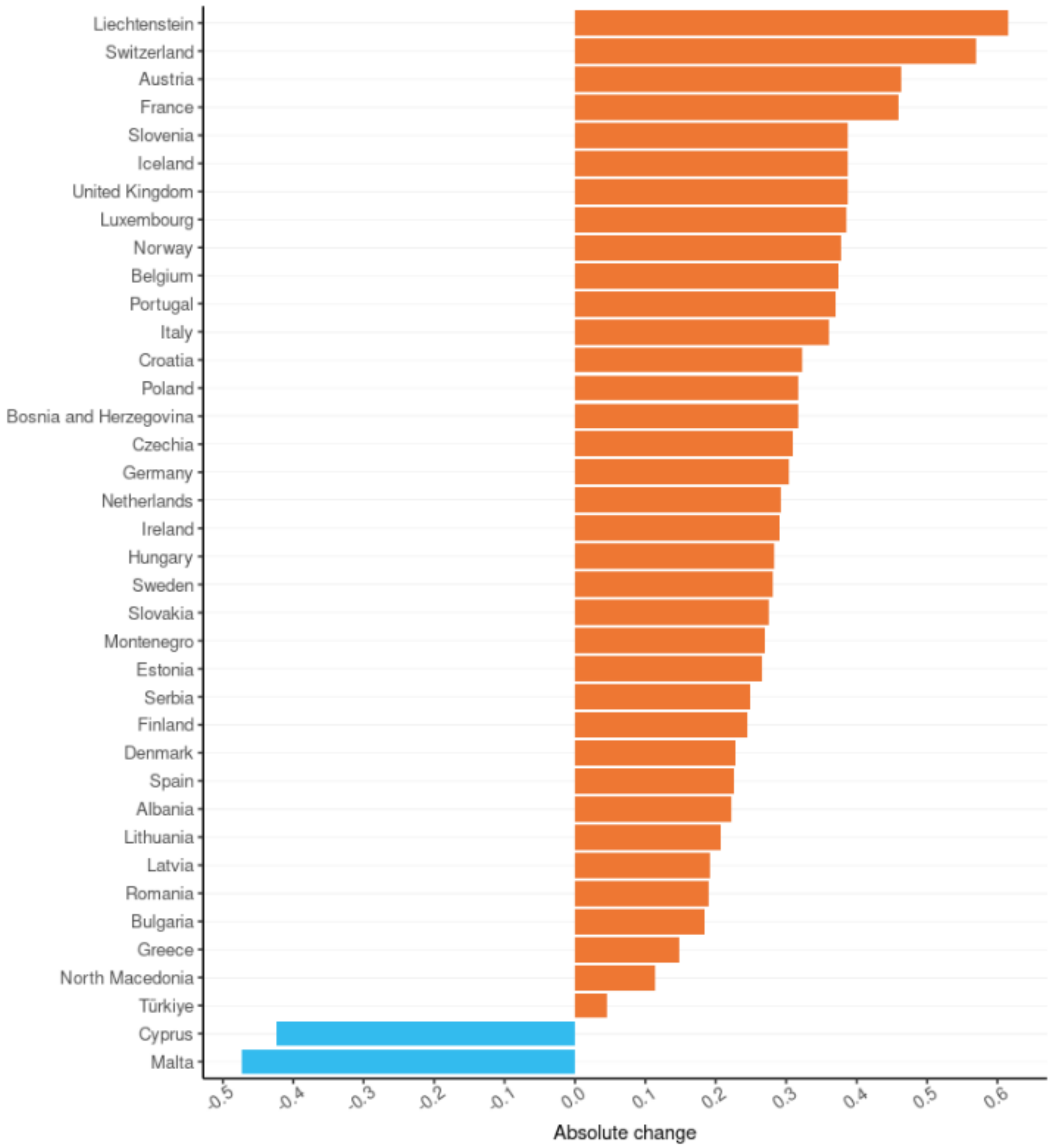

Figure 1.57 Absolute change in the in mean number of months with optimal conditions for Ixodes ricinus nymphs' feeding activity between 1951-1986 and 1987-2022 per country.

Change in the number of months suitable for tick activity between 1951-1960 and 2013-2022.

Table 1.13 Change in mean number of months with optimal conditions for Ixodes ricinus nymphs' feeding activity between 1951-1960 and 2013-2022 across European regions defined by the United Nations geo-scheme.

European region	Percentage change (Absolute change)
Eastern Europe	11% (0.58 month)
Northern Europe	14.7% (0.44 month)
Southern Europe	8.96% (0.53 month)
Western Europe	7.53% (0.37 month)
Western Asia	12.7% (0.68 month)

Table 1.14 Change in mean number of months with optimal conditions for Ixodes ricinus nymphs' feeding activity between 1951-1960 and 2013-2022 between level of social deprivation.

Level of social deprivation	Percentage change (Absolute change)
High	8.74% (0.0.49 month)
Medium	9.46% (0.50 month)
Low	15.6% (0.0.47 month)

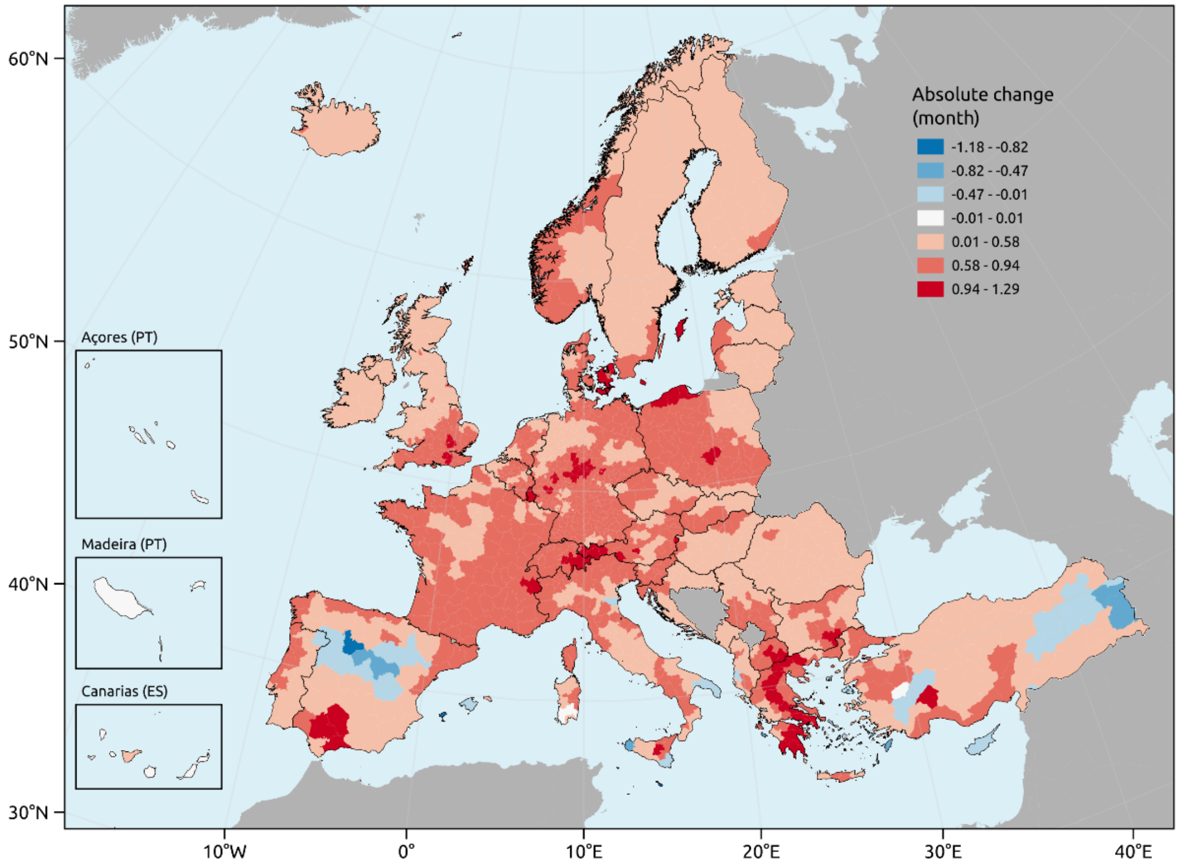

Figure 1.58 Absolute change in the mean number of months with optimal conditions for Ixodes ricinus nymphs' feeding activity between 1951-1960 and 2013-2022.

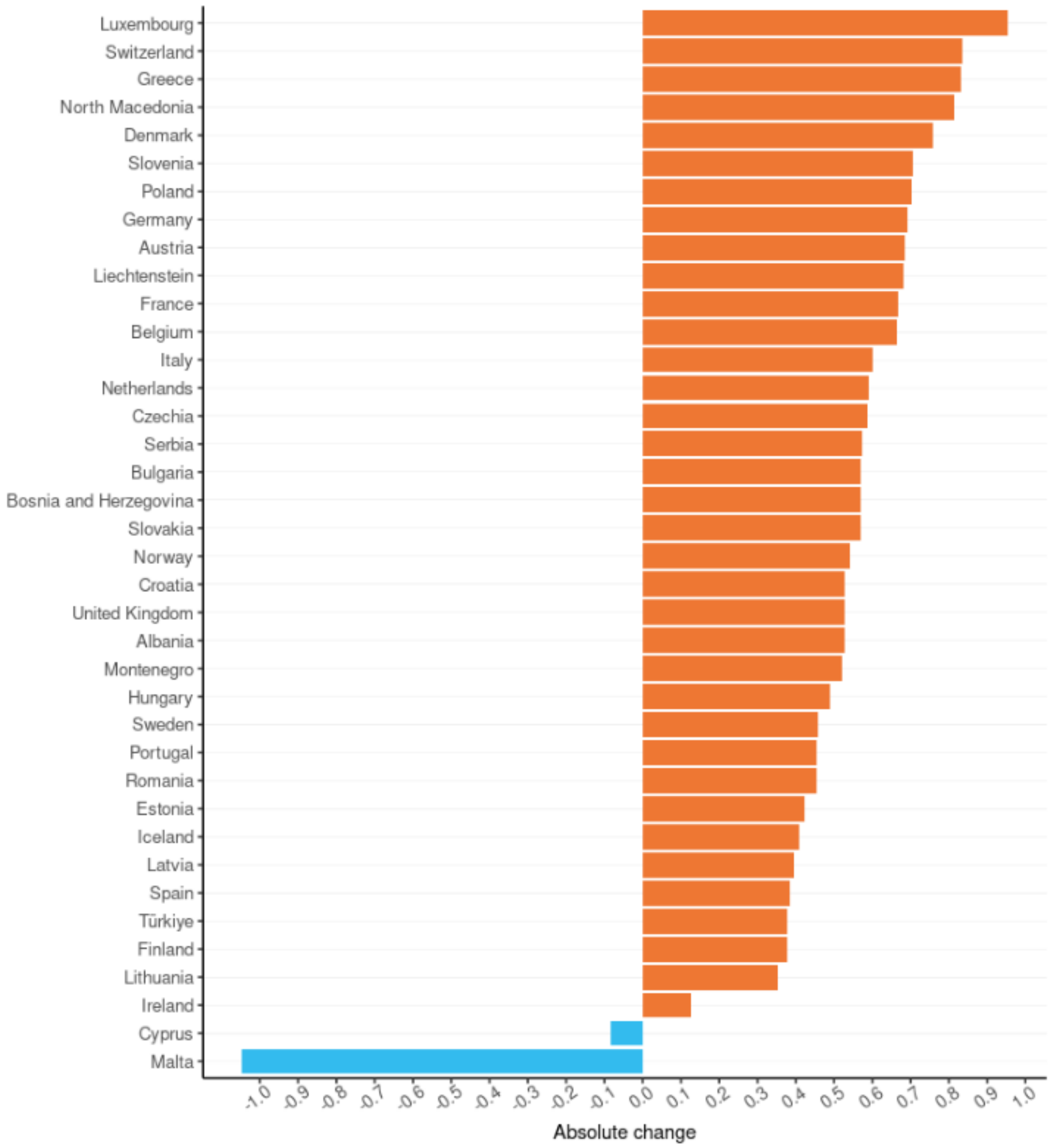

Figure 1.59 Absolute change in the in mean number of months with optimal conditions for Ixodes ricinus nymphs' feeding activity between 1951-1960 and 2013-2022 per country.

1.4: Allergens

Indicator 1.4.1: Allergenic trees

Geographic Coverage of Europe

For this indicator we considered the European Union member countries, candidate and potential candidate countries plus the United Kingdom of Great Britain and Northern Ireland and EFTA countries.

Data

The SILAM computations rely on three major data sets:

- Meteorological European Reanalysis ERA5[71] of European Centre of Medium-Range Weather Forecasting ECMWF. The period 1990-2022 was used for the indicator. It is based on computations of the ECMWF Integrated Forecasting System, IFS, with assimilation of large amount of in-situ and remote-sensing data. The ERA5 temporal resolution is one hour, and spatial resolution is about 25 km.
- The global land-use dataset ECOCLIMAP,[117] which provides 1 km global classification of land use. The land-use categories of ECOCLIMAP do not distinguish the individual tree species and do not provide temporal evolution of the land-use. Therefore, the ECOCLIMAP maps were combined with species-specific data of European Forest institute EFI[118] and Global Land Cover data GLC.[119] The final step of adaptation was an inverse problem solution with the SILAM model, which procedure was described by Prank *et al.* 2013,[120] aiming at the climatologically unbiased concentration predictions. The same map was used throughout the simulated period.
- The pollen concentrations with the season severity corrected via the data assimilation were taken from the European Pollen Reanalysis v.1.0, a joint work of participants of the European Aeroallergen Network, EAN, https://www.ean-net.org/en.html, whose data were made available for the SILAM team for the model development, evaluation, and the reanalysis computation. The number of assimilated stations varied from about-10 in early 1980s up to over a hundred in 2020s.[121,122]

Evaluation of the model predictions for 2022 was made using the data of European Aeroallergen Network provided to Copernicus Atmosphere Monitoring Service for model evaluation.

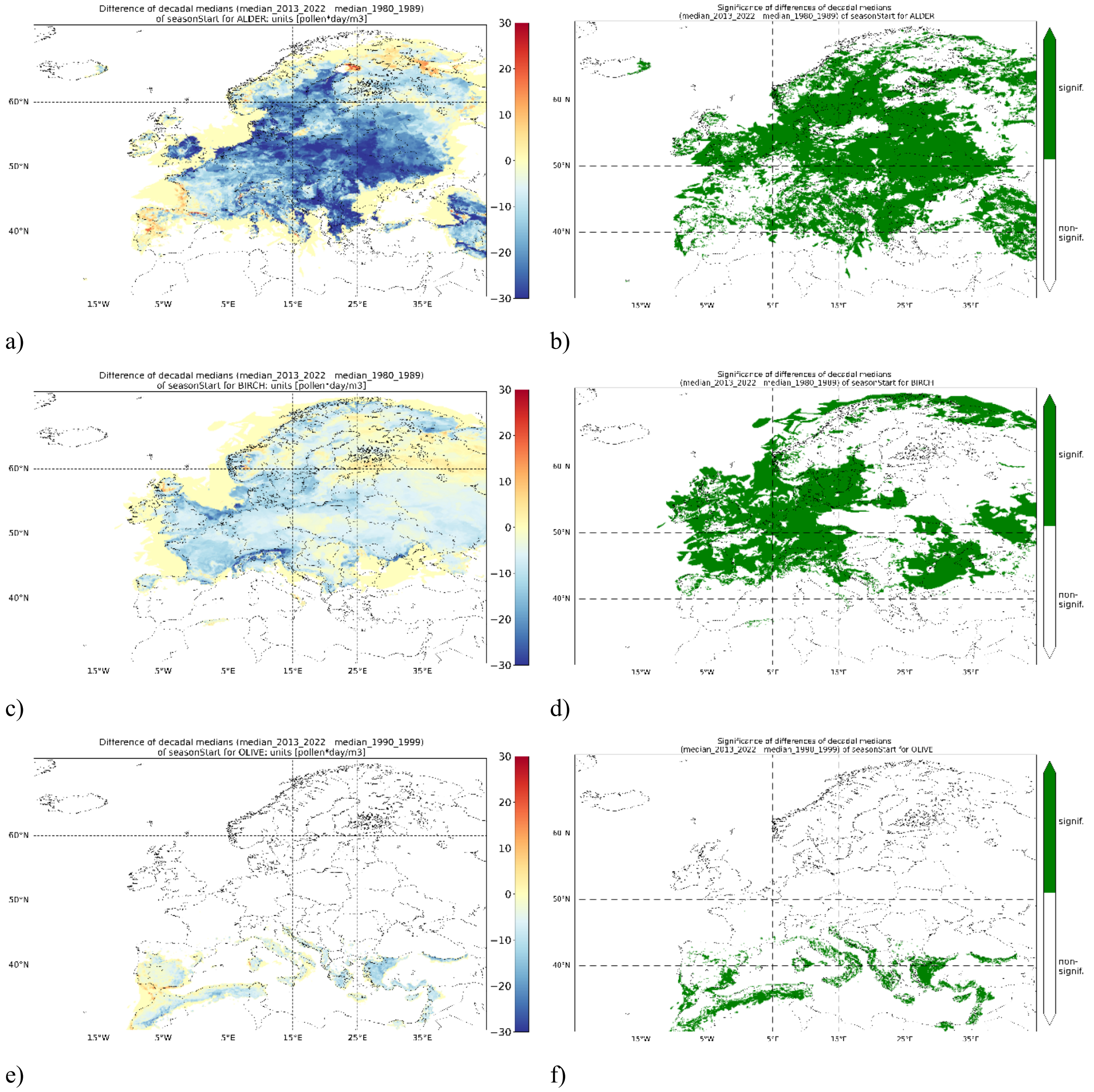

Figure 1.60 Difference of the decadal medians of start of the clinically-relevant season and their statistical significance for alder (upper panel), birch (middle panel), olive (lower panel). Left column: trends, unit: [days]; right column: significance with p-value < 0.1, i.e., trends over all green-colour areas are significant with p<0.1.

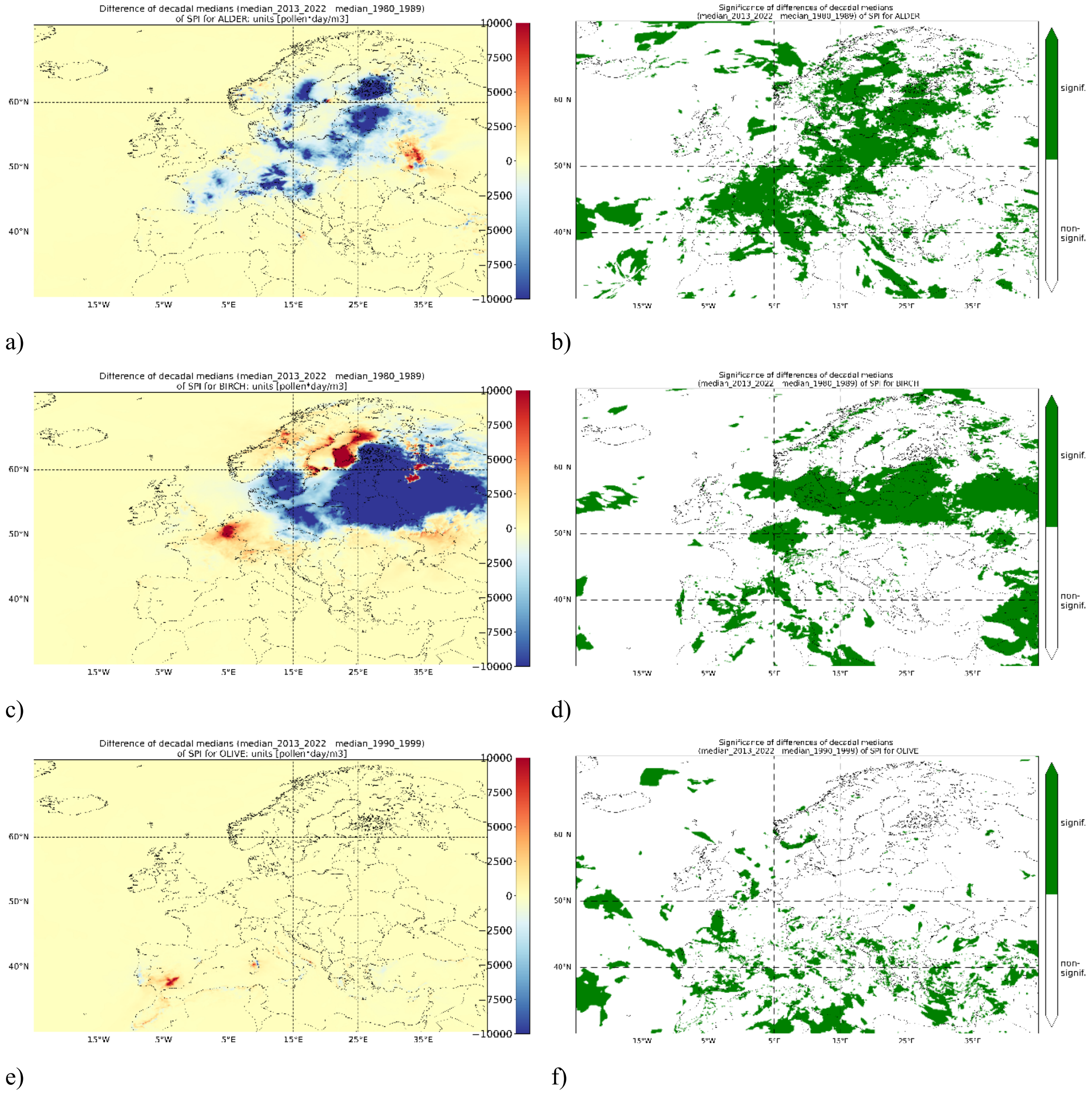

Figure 1.61 Difference of the decadal medians of the Seasonal Pollen Integral, SPI, and their statistical significance for alder (upper panel), birch (middle panel), olive (lower panel). Left column: trends, unit: [pollen*day/m3]; right column: significance with p-value < 0.1, i.e., trends over all green-colour areas are significant with p<0.1.

Methods

Changing climate leads to substantial changes in flowering season of European trees. The current indicator explores the season timing and abundance, the most-important parameters for pollen allergy sufferers.

Pollen season has many definitions, depending on the target use. The definition relevant in the current context is a so-called "Clinically-relevant" pollen season, i.e., the period when concentrations of the specific pollen are sufficiently high to cause allergy symptoms. It follows the guidelines of a Task Force of European Academy of Allergology and Clinical Immunology (EAACI).

Calculation of the clinically-relevant pollen season is performed by SILAM atmospheric composition model (Sofiev *et al.* 2015[123] and 2012[124] http://silam.fmi.fi, visited 5.3.2022; open-source code http://github.com/fmidev/silam-model visited 5.3.2022). The procedure is performed separately for each year.

The pollen season timing (start, end, and duration) is calculated from the unconstrained SILAM model runs. The flowering season timing is computed by SILAM phenological module as a period when a specific plant releases pollen into the atmosphere; the release is stored as a function of location and time all over Europe. Atmospheric dispersion of the released pollen is computed by SILAM, thus obtaining pollen concentration as a function of location and time all over Europe.

The season severity and its trends are calculated from the results of the European Pollen Reanalysis (EPR), v.1.0[121,122] which also presents the data assimilation technology and input data: meteorological reanalysis ERA5 and pollen data of the European Aeroallergen Network, EAN. Below, summarise the key elements of the methodology behind the EPR.

1. Assimilation of pollen observations in the European Pollen Reanalysis v.1.0.
    - the concentrations obtained from the initial unconstrained SILAM run are compared with observations and the error is minimised via iterative adjustment of total seasonal emission (an integral over entire season of the emission fields) of the initial run; the resulting emission correction fields are stored for all iterations;
    - an optimal iteration is selected using the L-curve approach to solution of the regularized ill-posed optimization problem; the emission correction from this iteration is stored as the outcome of the assimilation procedure
2. Final pollen concentration assessment
    - The outcome of the assimilation – the optimal emission correction of the EPR v.1.0 – is applied to the pollen productivity map and the computations of the initial setup are repeated;
    - clinically-relevant season definition of is calculated based on the computed concentrations;
3. The calculations are performed independently for each year through 1980-2022 for alder and birch and 1990-2022 for olive;
4. Trends of the clinically-relevant season start day, season end day, season duration, and total seasonal concentration integral are calculated as a decadal difference for each grid cell and NUTS2 region.

Details of the assimilation procedure are presented in the European Pollen Reanalysis methodology paper (Sofiev et al, in prep.), below we outline its main principles.

1. *Phenological season*

SILAM currently possesses phenological parameterizations for three European trees: alder, birch, and olive. For all of them, the phenological season computation is based on the concept of accumulated heat as the main trigger and the driver of flowering season.[124,125,126] For the heat accumulation, the Daily Temperature Sum model is written in the following form[124]:

(1)

$$H(D) = \sum_{d=D_0}^{D} \left(\overline{T(d)} - T_{co}\right) * U\left(\overline{T(d)} - T_{co}\right),$$

where D is day for which the heat sum is evaluated, D_0 is the day of start of heat accumulation, $\overline{T(d)}$ is daily mean temperature for the day d, T_{co} is cut-off temperature, and U is unity cut-off function:

(2)

$$U(x) = \begin{cases} 0, & x \leq 0 \\ 1, & x > 0 \end{cases}$$

Equations (1) - (2) imply that the heat accumulation rate is proportional to an excess of daily-mean temperature above the cut-off level. Same equations can be written also for hourly averaging, which for alder leads to a better agreement with the observed phenological timing than the daily model.

Phenological flowering season starts at the day D_s when heat sum exceeds a start-season threshold: $H(D_s) > H_{start}$. The season ends on the day when the accumulated heat reaches the end-season threshold: $H(D_e) > H_{end}$. For all trees, these thresholds depend on location, i.e., they are represented via maps rather than scalar values. This approach reflects the variety of the European climate zones as well as the marine-vs-continental climate gradient. The thresholds were empirically identified based on long-term SILAM simulations and pollen measurements across Europe by the European Aeroallergen Network (Sofiev *et al.* 2012,[124] 2015[127] and 2017[128]).

2. *Dispersion computations*

Release into the air and atmospheric dispersion of pollen was computed by SILAM driven by the ERA5 meteorological reanalysis of European Centre for Medium-Range Weather Forecasting (ECMFW). Spatial resolution was 0.1° lon-lat for the domain covering the whole Europe. Output included hourly-mean pollen concentrations, dry and wet deposition. Details of the computation procedure can be found in Sofiev *et al.* 2012,[124] 2015[127] and 2017.[128]

3. *Data assimilation*

The assimilation of the pollen observations followed the principles outlined by. It has been shown that lasting improvement of the model predictions require extended assimilation approaches correcting emission fields, whereas classic forms of assimilation of the model state (concentrations) lead to unsatisfactory results. The data assimilation run used the stored hourly pollen emission of the initial run as the first guess, $E_{fg}(i,j,t)$. The outcome of the assimilation was the map of correction factors $\alpha(i,j)$, constant-in-time multipliers, different for each year, for the first-guess emission fields, which turn them into the a-posteriori solution of the data assimilation problem $E_{assim}(i,j,t)$:

(3)

$$E_{assim}(i,j,t) = \alpha(i,j)E_{fg}(i,j,t)$$

The cost function J for minimization is then written as a regularized minimization problem:

(4)

$$J(\alpha) = \frac{1}{2}(y - Hx)^T R^{-1}(y - Hx) + \frac{1}{2}(\alpha - 1)^T B^{-1}(\alpha - 1)$$

Here, x is the model-predicted concentrations, y is observed concentrations, H is observation operator, R is observational covariance matrix, B is covariance matrix of emission correction α. The first term penalizes the deviation of concentrations from observations, the second one – deviation of α from the first-guess unity correction. The SILAM model operator M sets the connection between α and x:

(5)

$$x = M\alpha E_{fg}$$

Minimization of J goes iteratively, thus obtaining a series of correction factors α_n, n=1...N. Out of this set, an optimal iteration α_{opt} is selected, corresponding to a balance between the deviation from the observations and deviation from the unity emission scaling.

4. Clinically relevant season

Computations of clinically-relevant season follows the definition of Pfaar *et al.* 2016[129]: Start of season is the 1st day of 5 days – out of 7 consecutive days – each of these five days with concentration exceeding C_{daily_clin} and with a sum of these five days exceeding C_{5days_clin}. The authors identified the values of the two constants for 5 species: birch, grass, cypress, olive, and ragweed. For this indicator, only birch and olive were used (Table), whereas the constants for alder were roughly estimated using mean seasonal exposure to this pollen in comparison to birch and olive. For the trend of the season start, this is a satisfactory approximation. For actual season computations, the consensus thresholds for alder must be identified.

Table 1.15 Numerical constants for concentration thresholds, modified from Pfaar *et al.* 2016[129].

	*Alder**	*Birch*	*Olive*
C_{daily_clin}	3 pollen m^{-3}	10 pollen m^{-3}	20 pollen m^{-3}
C_{5days_clin}	30 pollen m^{-3}	100 pollen m^{-3}	200 pollen m^{-3}

Note: The Task Force of Pfaar et al did not identify the constants for Alder. The values were estimated by extrapolating the typical pollen concentrations of birch and olive and comparing them to those of alder.

5. Multi-annual computations and trend analysis

The SILAM computations have been made for 1990-2022 using the European Reanalysis ERA5.[71] Each year is computed independently, with the same amount of pollen released as a seasonal integral in the first-guess run. Upon completion of the assimilation cycle (due to a lack of measurements in 1980s, it covered only 1990-2022), the clinically-relevant season start, end, and duration, as well as the Seasonal Pollen Integral, SPI, were computed from daily pollen concentrations for each grid cell and for each NUTS region as a difference of decadal medians DIFF, Eq. (6). Based on the obtained time series of season characteristics, the decadal difference over 33 years was calculated at every grid cell/NUTS region.

(6)

$$DIFF = MEDIAN_{2013\_2022} - MEDIAN_{1990\_1999}$$

Caveats

The indicator employs the major simplification: the same vegetation distribution map is applied for all years in the first-guess initial runs.

The clinically-relevant season depends not only on the phenological season but also on absolute pollen concentrations. For trees, strong fluctuations between years are common: strong-pollination years are often followed by weak years and vice versa. However, predicting the absolute level of pollen season for each specific year is a very difficult task, and no adequate European-wide solution exists for it this-far. The first-ever regional model of Ritberga *et al.* 2017[130] covers only Northern Europe. Therefore, this indicator includes assimilation of available data as a part of the model development and adaptation to real-life conditions. This step has largely alleviated the problem of fixed source maps: correction factor for emission automatically also corrected the abundance of trees in the grid cell.

The second significant limitation is the lack of observations in early years. As a result, it has been decided to limit the assimilation to 1990-2022 for olives leaving the earlier years intact and excluding them from the trend analysis. Finally, assimilation of time series with non-perfect correlation leads to under-estimation of the total seasonal emission (the basic features of the *J* functional (4), see Sofiev et al, in prep.).

Future form of the indicator

The European Pollen Reanalysis opens several possibilities. The indicator will be developed in four directions. The inter-annual season-strength forecasting model will be expanded to the whole Europe and applied to obtain a more realistic season severity prediction for each specific year. It will be combined with the data assimilation efforts, with the overall idea to allow for inclusion of earlier years using combined model and (few) observations of 1980s.

The fixed land-use map will be switched to a series of annual maps, each adjusted to the specific forest distribution pattern obtained from satellites for each specific year. It is anticipated that such maps will not be available for the pre-satellite era, but an effort will be made to obtain reference distributions at least every 5-10 years from the in-situ observations and inventories of EFI. The final refinement will, again, be based on the EPR v.1.0 results. Better habitation maps will improve the correlation of the first-guess, thus reducing the artificial low bias of the assimilated fields.

The ERA5 archive is being extended by ECMWF towards 1950. Upon completion of this work, the indicator will be extended to cover additional 30 years in the past. With time passing, the indicator will be updated on annual basis to include the recent-most years.

Additional analysis

The obtained trends show a significant spatial variability, both in the trend absolute values and their statistical significance (**Figure 1.60; Figure 1.61**). The high variability has been shown to originate from year-to-year meteorological variability. However, over the areas with a statistically significant trends (green areas in the left column of **Figure 1.60**), the shift of the season reached 10-20 days during the last 40 years. It exceeds one month for e.g. birch season start in Alpine region. Highly systematic shift was found also for olives: in practically all olive-rich regions, the shift reached about 10 days over last 40 years. Trends for the SPI are also highly inhomogeneous in space, with regional upward and downward trends controlled by the local vegetation and land-use dynamics.

Apart from the season start time, the season duration and the season end time are of high interest for allergy sufferers. As follows from Eq. (*2*) and the definition of, the season end can be obtained following the same procedure as for the season start. This signal is less articulated in the time series, partly due to pollen resuspension, partly due to regional and long-range transport. However, it is still possible to compute its trends with reasonable statistical significance: **Figure 1.62**.

Comparing **Figure 1.60** and **Figure 1.63**, one can see that both season start and end are shifting synchronously in most of European regions, but the start is shifting slightly faster. As a result, the season duration stays almost constant (**Figure 1.64**), with practically no statistically significant trends in any region.

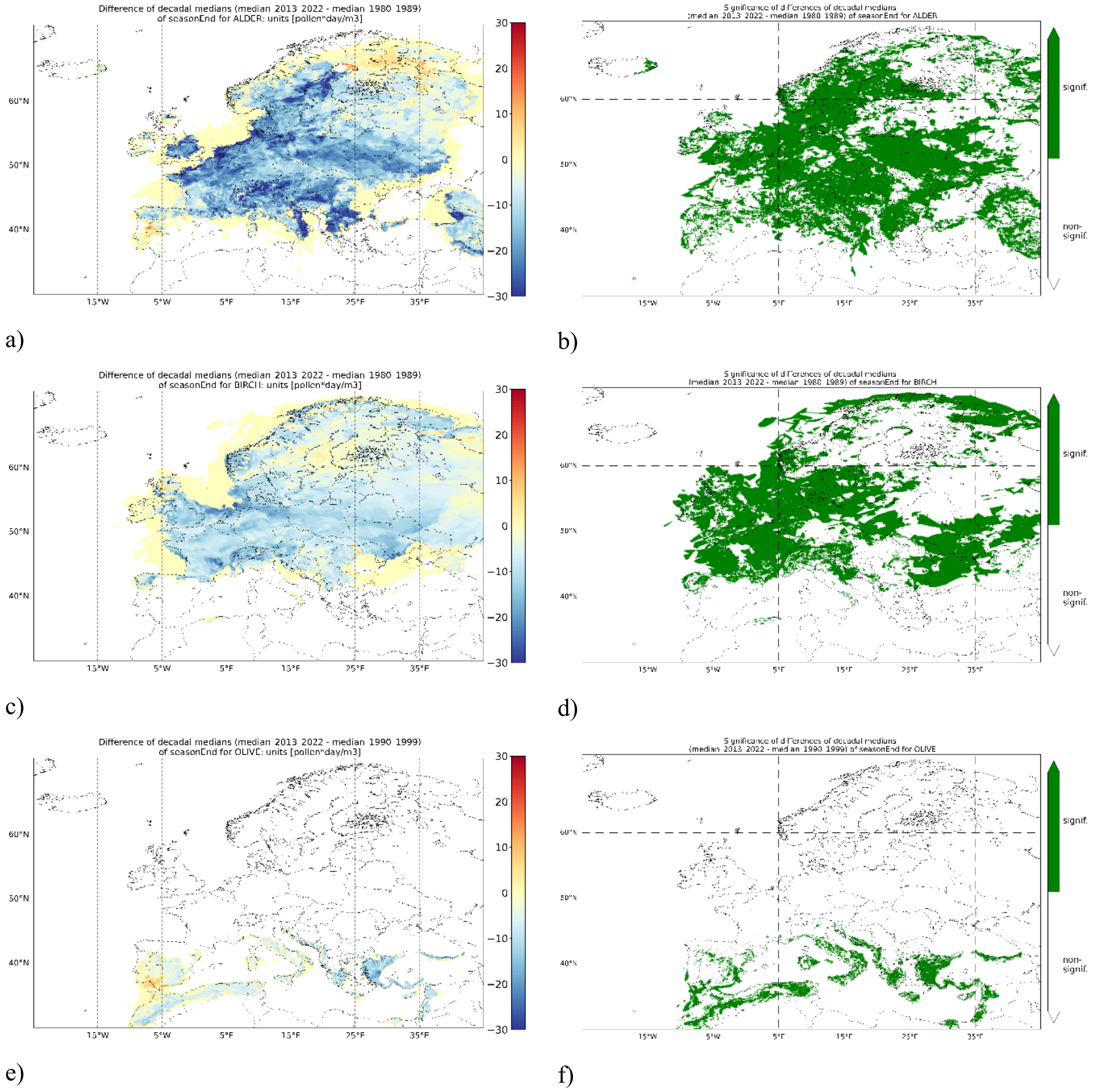

Figure 1.62 Difference of the decadal medians of the end of the clinically-relevant season, and their statistical significance for alder (upper panel), birch (middle panel), olive (lower panel). Left column: trends, unit: [days]; right column: significance with p-value < 0.1, i.e., trends over all green-colour areas are significant with p<0.1.

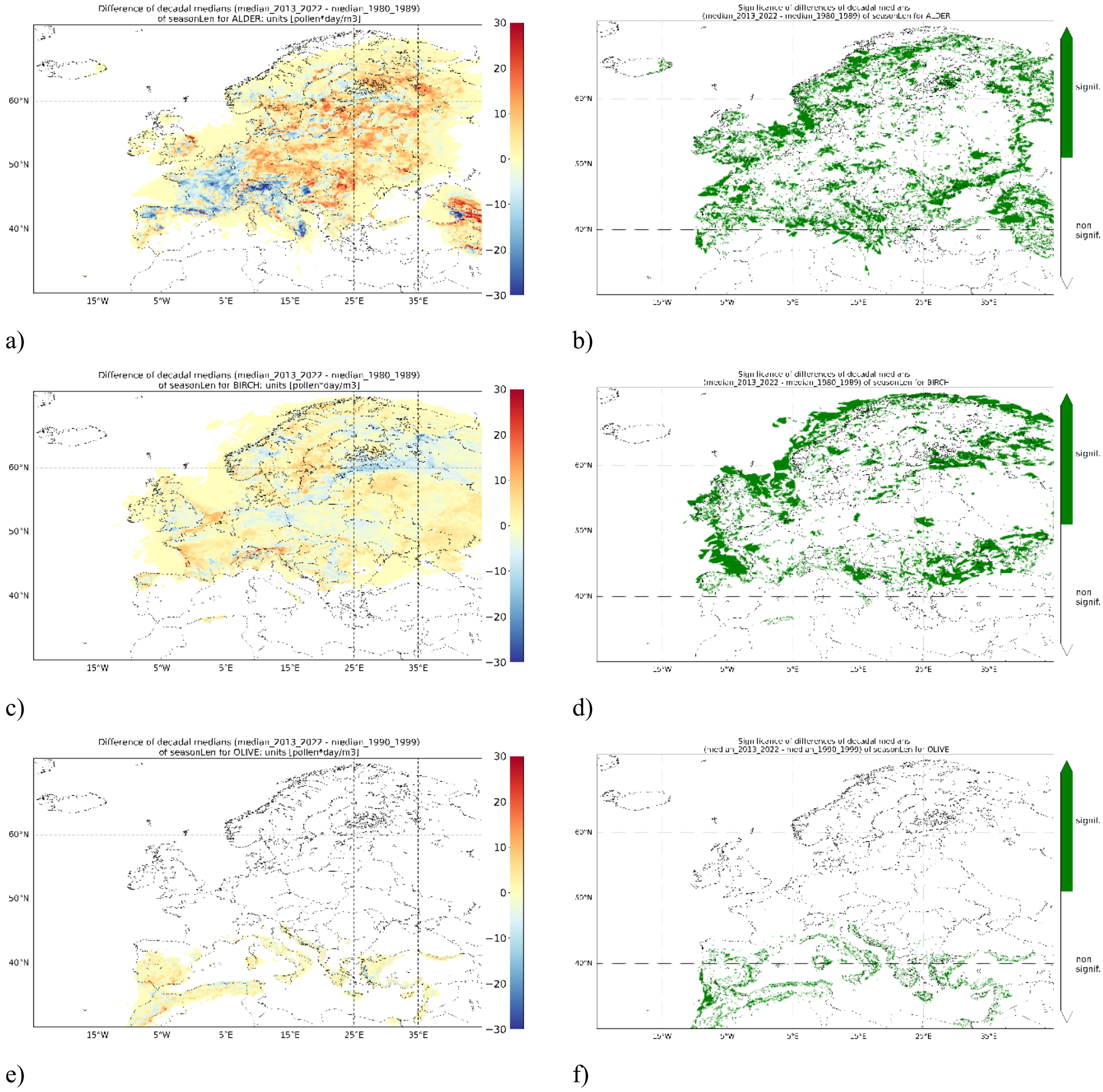

Figure 1.63 Difference of the decadal medians of duration of the clinically-relevant season, and their statistical significance for alder (upper panel), birch (middle panel), olive (lower panel). Left column: trends, unit: [days]; right column: significance with p-value < 0.1, i.e., trends over all green-colour areas are significant with p<0.1.

The SILAM pollen predictions have been extensively evaluated in a series of international projects. An example of 2022 evaluation of the first-guess birch and olive season abundance withing the Copernicus Atmosphere Monitoring Service CAMS is shown in **Figure 1.64**. That year, the birch season was significantly overestimated in Northern Europe by the first-guess run (corrected by the assimilation in the current dataset), but the model still reproduced well the season in Central Europe. Practically no bias was revealed in predictions of olive pollen concentrations.

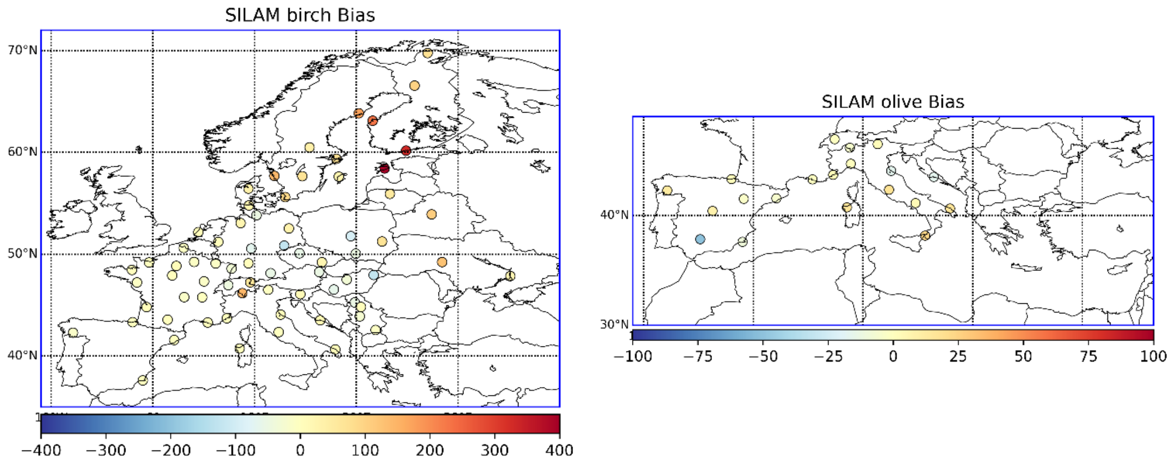

Figure 1.64 Quality of the season abundance identification in 2022. Left-hand panel: mean season bias of birch pollen concentrations; right-hand panel: mean season bias of olives pollen concentrations. [pollen*day/m3]. Observational data are provided by European Aeroallergen Network to CAMS for the model evaluation within the scope of CAMS-23 service contract.

Several studies reported local/regional trends in the pollen season timing, e.g., but no long-term European-scale trends have been published this-far due to a lack of historical pollen observations. Comparing the current trend estimates with the published local assessments, there is a broad consensus in a tendency towards the earlier season in most of Europe. Specific trends vary between the stations and species, similar to patterns calculated for this indicator.

Acknowledgements

The work has been performed within the scope of Horizon Europe project SYLVA (grant 101086109) and Academy of Finland project ALL-IMPRESS, grant 329215; the model evaluation was performed within Copernicus Atmospheric Monitoring Service, CAMS. The European Pollen Reanalysis v.1.0 is a joint development of the European Aeroallergen Network teams, who contributed to the EPR.

1.5: Food and water

Indicator 1.5.1: Food security

Geographic Coverage of Europe

For this indicator we include the 37 WHO Europe member countries included in the FAO Food Insecurity Experience Scale (FIES).

Data

1. Temperature and SPEI: ERA5-Land.[24]
2. Food insecurity: FAO Food Insecurity Experience Scale (FIES).[131] FIES "represents a significant change in approach to food insecurity measurement compared to traditional ways of assessing it indirectly through determinants such as food availability or consequences such as poor-quality diets, anthropometric failures and other signs of malnutrition". The indicator is "developed by professionals from the nutrition field," includes quantity and quality measures, making it particularly relevant for the Lancet Countdown and its focus on climate change and health. "FIES provides a tool for the nutrition and food security community to build on existing knowledge regarding relationships between the experience of food insecurity and indicators of malnutrition".[132]

Methods

The methodology of this indictor is based on Dasgupta and Robinson.[133] To track the impact of climate change and income on the incidence of food insecurity, we use a panel data regression with coefficients that vary over time. To operationalise the concept of climate change, we focus on the number of heatwave days and the frequency of droughts each region. A heatwave is defined as a period of at least two days where both the daily minimum and maximum temperatures are above the 95th percentile of the respective climatologies (Lancet Countdown 2022 - Indicator 1.1.2) in each region. The gridded 95th percentile of daily minimum and maximum temperatures, taken from the ERA5-Land hourly dataset,[24] were calculated for 1986-2005. We use the lagged number of heatwaves and frequency of droughts (measured by SPEI-12) during 2014-2021.

Increase in the number of heatwave days can affect food insecurity through multiple pathways.[134] These can variously be through the impacts of heat stress and droughts on crop yields, on agricultural labour and therefore crop production and agricultural income, on non-agricultural labour and non-agricultural income, on health and the ability to earn enough to afford food, on food prices and therefore the affordability of food, and on food supply chains and therefore the variety of food (which we can summarise as income and food supply effects). Our regression also includes twelve-month Standardized Precipitation Evapotranspiration Index (SPEI) as a measure of drought. SPEI-12 was computed using precipitation data from ERA5-Land monthly averaged dataset[24] and the SPEI package in R.[57]

We consider two dependent variables: first, the probability of moderate to severe food insecurity; and second the probability of severe food insecurity from the FAO Food Insecurity Experience Scale (FIES) from 37 WHO Europe member countries. To account for unobserved heterogeneity such as differences in food and storage policies across countries and changes in the prices of food items from year to year, our specification also includes both location and time (year) fixed-effects. The standard errors are clustered at the country-level.[135] Our panel data specification can be written as follows:

$$FIES_{it} = \beta_1(\tau_t) + \pi_{it}V_{(it)} + \gamma'(\tau_t)X_{(it)} + \alpha_{(i)} + \mu_{(it)}$$

where $FIES_{it}$ is the probability of moderate or severe food insecurity or probability of severe food insecurity, $\pi_{it}V_{(it)}$ is a vector of change in the number of heatwave days and the frequency of drought months, and $\boldsymbol{X}_{it}$ is a vector of relevant variables affecting food insecurity – income and a dummy to control for the COVID-19 pandemic in 2020. $\boldsymbol{\mu}_{it}$ is a random error term. All variables are recorded for different locations with index $i = 1, \ldots, N$ and over a number of years $t = 1, \ldots, T$. The time-varying coefficients allow us to examine whether the relationship between temperature anomaly and food insecurity has evolved over time. $\alpha_{(i)}$ and $\mu_{(it)}$ are location and survey wave fixed-effects, respectively.

In the second-step, we conduct a counterfactual analysis to explore the extent to which food insecurity may have been affected by climate change.[133] To do this we compute the cumulative impacts of increasing frequency of heatwaves and frequency of drought months above the historical norms over the period 1981–2010. The counterfactual impact of climate change on food insecurity is derived by combining the coefficients from the time-varying regression with the historical norm average and each year for which we have food security data. We consider the effects of increases in the frequency of heatwaves and frequency of drought months over compared to the baseline (1981–2010) under which frequency of heatwaves increases according to its historical trend.

Caveats

The main caveat the food insecurity indicator is the possible recall bias in the survey data and the bias that may have been induced to interviews during the pandemic being conducted by phone instead of in-person visits.

Future Form of the Indicator

In the future, we will provide disaggregated analysis by income groups.

Analysis

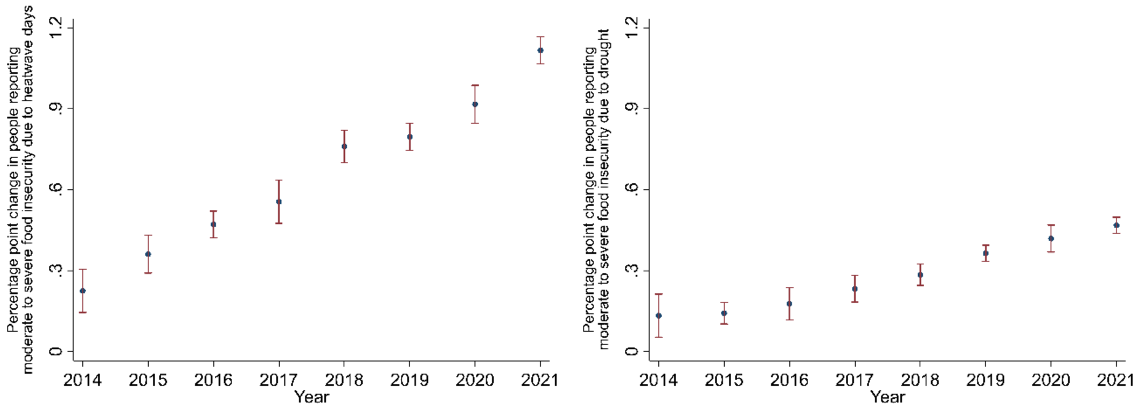

Figure 1.65 Change in the share of the population (percentage point change) reporting moderate or severe food insecurity due to heatwave days (left-panel) and frequency of drought months (right-panel).

	**Moderate or severe food insecurity**
**Low income**	0·119 (0·115, 0·123)
**High income**	-0·258 (-0·250, -0·266)
**COVID-19 dummy**	0·098 (0·095, 0·101)
**Heatwave frequency($_t$)**	
2014	0·225 (0·145, 0·305)
2015	0·361 (0·291, 0·431)
2016	0·471 (0·421, 0·521)
2017	0·555 (0·475, 0·635)
2018	0·761 (0·701, 0·821)
2019	0·796 (0·746, 0·846)
2020	0·917 (0·847, 0·987)
2021	1·117 (1·067, 1·167)
**Drought frequency($_t$)**	
2014	0·133 (0·053, 0·213)
2015	0·142 (0·102, 0·182)
2016	0·177 (0·117, 0·237)
2017	0·233 (0·183, 0·283)
2018	0·285 (0·245, 0·325)
2019	0·364 (0·334, 0·394)
2020	0·419 (0·369, 0·469)
2021	0·468 (0·438, 0·498)

Table 1.16 Relationship between frequency of heatwaves and droughts, and food insecurity during 2014-2021 using a time-varying regression. 95% confidence intervals in parentheses.

Section 2: Adaptation, planning and resilience for health

Panel 2.1 Nature-based solutions to climate change and health.

Nature-based solutions (NbS) are integrated interventions that can synergistically benefit climate change adaptation and mitigation, protect biodiversity and benefit human wellbeing, whilst reducing trade-offs between addressing these three challenges.[136]

Implementation of NbS in Europe has been rapidly scaled up, particularly within cities.[137] Analyses on NbS (n=1012 projects) in European cities using the Urban Nature Atlas database find a steady increase in newly implemented NbS over the last three decades, despite a decline in new initiatives during the COVID-19 pandemic (**figure 2.1**). Large urban parks, pocket parks, neighbourhood green spaces, and community gardens are among the most reported NbS projects. Newly registered initiatives are slightly more common in western Europe (306 projects), followed by northern Europe (281), southern Europe (245), eastern Europe (170), central Asia (6), and western Asia (4). Around 60% of urban NbS describe one or several of the following intended health impacts: gain in activities for recreation and exercise, improved physical health, and improved mental health. The limited nomenclature and reporting of health effects within the Urban Nature Atlas illustrate that health effects may be underestimated or not properly accounted for within NbS design and implementation.

NbS can affect people's health and well-being through various mediating pathways,[138,139,140] albeit not all are fully understood. NbS can have several health co benefits (**table 2.1**), however, poorly designed NbS can likewise lead to unintended negative consequences such an increase in vector-borne diseases or allergenic agents (**table 2.1**).[141] Importantly, NbS implementation that does not include equity considerations may reproduce unequal access across different population groups, further perpetuating environmental and health inequities (e.g., those living in low socioeconomic neighbourhoods,[142] or central urban areas may have less access to green-blue space[143]). Growing literature also highlights that new green infrastructure can contribute to gentrification, thus creating new social, health and racial inequities.[144] This calls for accompanying anti-displacement and inclusive green policies preventing further unequal urban development.[145,146]

If not considered in the planning stage, unintended consequences and inequities may arise, develop and accumulate over time. This can lead to offsets in the intended positive impacts of NbS and hinder acceptance of NbS implementation among decision-makers and local communities. Mitigation of unintended consequences requires a thorough and transdisciplinary approach which prioritises equity and implements preventive policies for vector control, education and empowerment of local communities, communication campaigns, as well as improved healthcare access and services.

Table 2.1 Health co-benefits and potential unintended consequences of NbS

Health co-benefits:

- Protection against spillover of zoonotic diseases through reduction of contact with reservoirs
- Space for physical exercise and recreational activities
- Improved air and water quality
- Exposure to activities reducing stress and anxiety
- Space for social interaction and cohesion, fostering a sense of belonging within a community and contributing to mental health
- Reduced exposure to UV light, (urban) heat and noise
- Prevention of allergenic sensitization in children exposed to green spaces (particularly in late-childhood)[147]

Unintended health consequences:

- Exposure to vectors of vector-borne diseases and vector-bite nuisance
- Exposure to reservoirs or intermediate hosts of zoonotic diseases
- Exposure to allergenic agents, such as pollen, grass, and mold among people prone to developing allergic disease
- Exposure to water-related health hazards associated with recreation

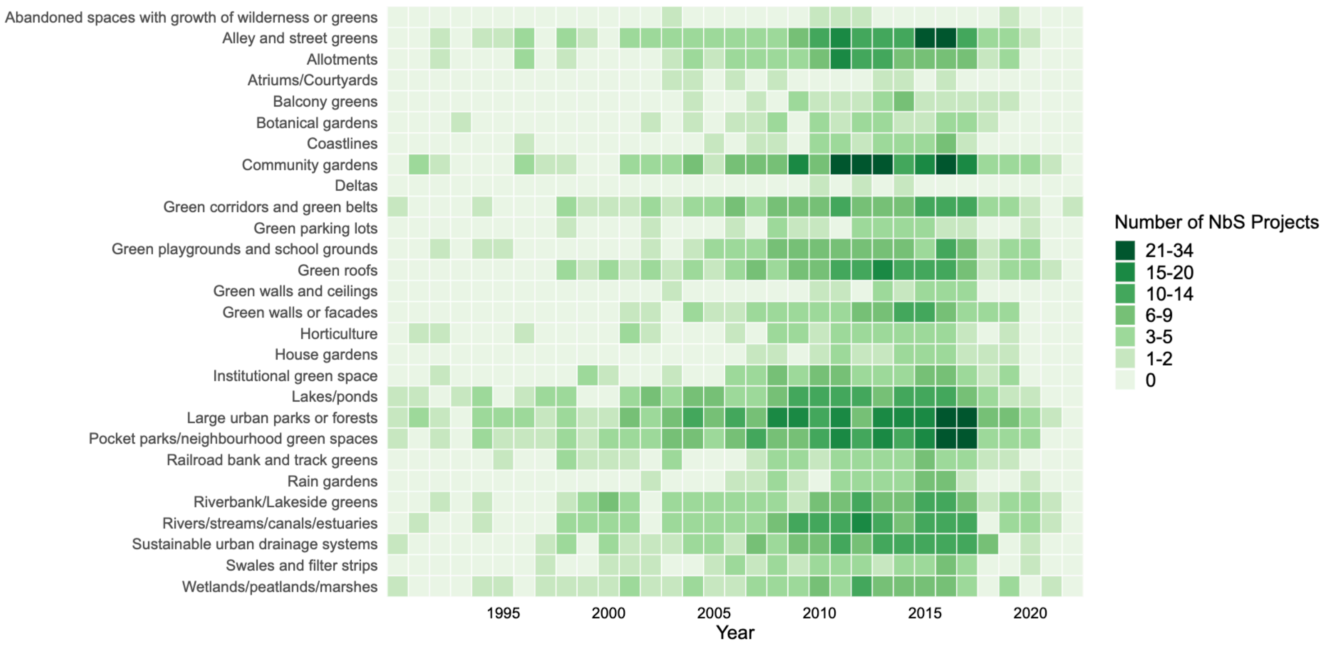

Figure 2.1 Implemented urban NbS initiatives (n=915) in the WHO European region reported to the Urban Nature Atlas. NbS are presented by year and NbS type. Note, this figure is based on NbS projects self-reported to the Urban Nature Atlas. Planned and initiated NbS are not included.

Geographic Coverage of Europe

For this panel, the 53 WHO Europe countries plus Liechtenstein and Kosovo (under UNSC resolution 1244) were included.

Data

1. Urban Nature Atlas. (May 15th, 2023). https://una.city/

The database has been constructed and maintained by Urban Nature Atlas initiative. The Urban Nature Atlas was developed in 2017 and is maintained up to the present. It contains profiles of over 1000 projects from European cities. The data is collected within the NATURVATION Horizon 2020 project and is currently maintained by PHYSI Solutions Ltd., founded in 2022 (https://www.physi.earth/about/). As of May 2021, the database was opened up to allow the submission of new projects and is updated regularly. Data collected is all submitted in a questionnaire format and undergoes quality control. Compared to other websites, the structure of the website https://una.city/ facilitates the usage of webscraping techniques for text mining. There are 1012 NbS projects on UNA within the WHO European Area. 24 of the projects are in the planning stages and for 73 it is unknown if they are completed/implemented/planned/initiated etc. For the remaining 915 projects they are either completed or actively being implemented.

Methods

Webscraping and Natural Language Processing (NLP), specifically text mining and information extraction techniques, were applied to gather the data on NbS projects in Europe from the Urban Nature Atlas hosted on the website una.city. Data was collected using the R package 'rvest' on a per-project basis using html tags and includes details such as location, management, funding sources, interventions, solutions, objectives, scale, and impacts. These details were further collated, descriptively analysed, and visualised for thematic trends and geographic and temporal patterns.

The code used to construct this indicator and create the visualizations included in this publication is available at the following repository: https://github.com/climate-and-AI-lab/NbS_Lancet/tree/main.

Caveats

The data for the Urban Nature Atlas was collected within the NATURVATION Horizon 2020 project through surveys to 100 cities selected across Europe. As of May 2021, the database was opened up to allow for the submission of new projects and is updated regularly. However, despite these recent changes, the original survey design was not a random sample and may have biased the database to a subset of European cities possibly by their population size, political significance, fame, or other factors. This may have impacted the distribution and concentration of NbS projects across Europe and further attention is necessary to account for these biases. When examining the dataset and focusing on regions where countries with listed projects are present on una.city, we observe that Western Europe consists of 5 countries, Eastern Europe has 7 countries, Southern Europe has 7 countries, Northern Europe has 7 countries, West Asia has 3 countries, and Central Asia has 2 countries. Therefore, the variation in the number of countries within each region does not seem to account for the difference in the reported number of NbS. The variation could potentially arise from factors such as disparities in national

and municipal wealth, urban programs, and funding, or even the prevalence of professionals in the urban/landscape design and development fields. These factors, among others, could contribute to the discrepancy observed in the distribution of NbS projects among different regions.

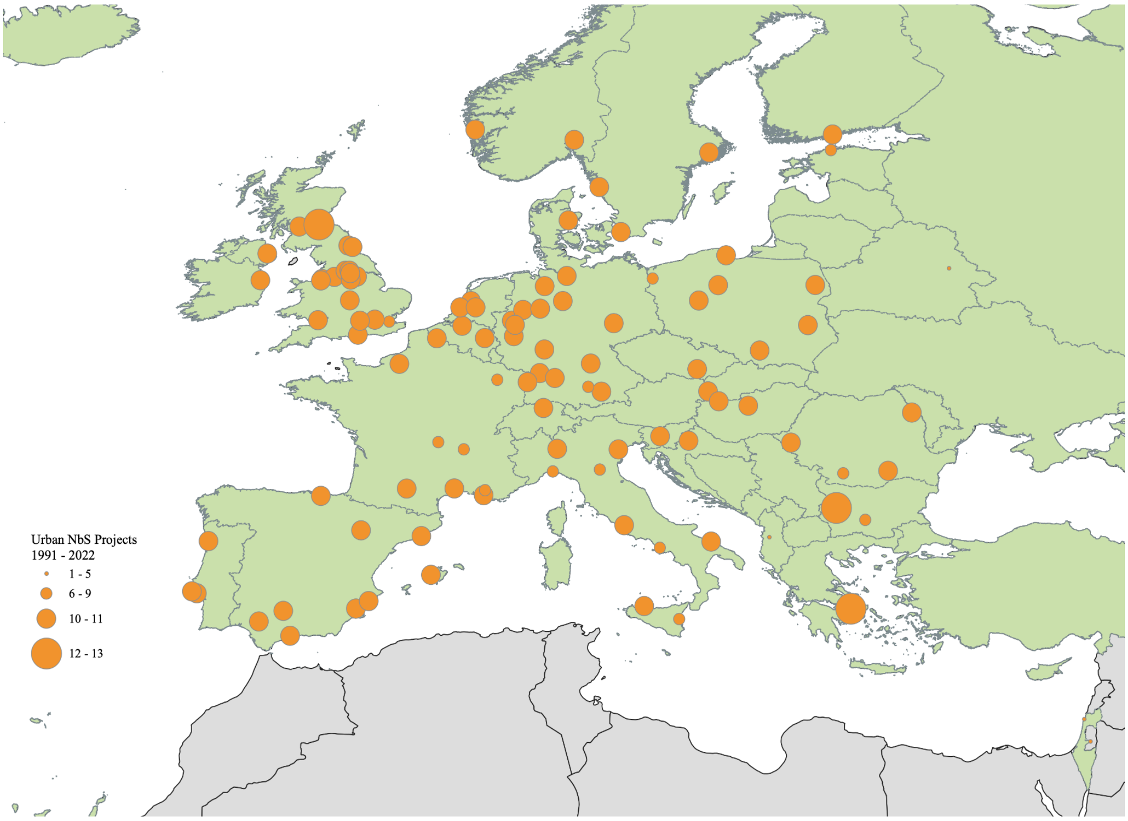

Figure 2.2 Map of NbS counts by city across the WHO European Region. Source of data: Urban Nature Atlas (https://una.city/). Note: countries shown in the figure without an orange blob indicate that they did not report the implementation of NbS in the database.

2.1: Adaptation planning and assessment

The following section describes the methods, data, caveats, geographic coverage, and future forms of the indicators for three section 2 indicators that are based on the WHO Health and Climate Change Global Survey.

Indicator 2.1.1: National vulnerability and adaptation assessments

Indicator 2.1.2: National adaptation plans for health

Indicator 2.2.1: Climate information services for health

Geographic coverage of Europe

For this indicator, we include countries that completed the WHO survey. The 2021 survey was completed by 22 member states with representation from the WHO European Region out of the 53 member states. See **Table 2.2**.

Table 2.2 The list of participating countries in 2021 global survey from the WHO European Region

List of participating countries and areas	Number of participating countries	Proportion of WHO region represented
Azerbaijan, Bulgaria, Croatia, Cyprus, Czechia, Estonia, Germany, Israel, Italy, Kazakhstan, Kyrgyzstan, Lithuania, Netherlands (Kingdom of the), North Macedonia, Poland, Portugal, Republic of Moldova, San Marino, Serbia, Slovakia, Sweden, Turkmenistan	22	42%

Source: 2021 WHO health and climate change global survey report.[148]

Data

Validation of the 2021 country reported data was undertaken in multiple steps. First, survey responses were reviewed for missing information or inconsistencies with follow-up questions directed to survey respondents. A summary of responses was shared with WHO regional focal points and key informants for review, comments and validation. Source documents including national health strategies and plans, and climate change and health vulnerability and adaptation assessments were collected. A desktop review of these source documents was conducted to compare with survey results with follow-up to survey respondents to seek clarification or additional documentation. Findings were also cross referenced with existing external publications. Data were collected detailing all the ministries, institutions and national stakeholders that provided contributions to or review of the survey responses in order to provide insight into the national consultation process of each survey submission.

Finally, all respondents reviewed and acknowledged the WHO data policy statement on the use and sharing of data collected by WHO in Member States outside the context of public health emergencies.

Of note, due to the ongoing pandemic, the standard data collection procedures were modified to reduce the reporting burden on countries that wished to participate in the Global Survey but that were facing human resource constraints due to pandemic response. In eight cases, WHO prepared pre-filled survey questionnaires with data provided by ministries of health in the previous 2018 survey cycle or using data the countries had published in the

2020/2021 WHO UNFCCC health and climate change country profile when available. These countries were requested to review, revise, and complete the hard copy questionnaires. These hard copy questionnaires were then entered into the online platform by WHO. The same data validation steps as described above were then followed. Additionally, a number of countries requested an extension of the reporting period. As such, there may be a slight increase in the total number of participating countries in the WHO health and climate change global survey report after the time of the publication of the report and an online dynamic data dashboard will reflect any updated data and findings as required with specified version time and date.

Caveats

1. The global survey is conducted every three years, and not all the same countries participate every year.
2. The survey sample may not be a representative sample of all European countries as the survey is administered on a voluntary basis.

Future form of the indicator

1. For the next iteration of the indicator, self-reported data for adaptation plans, assessments, and climate information services can be disaggregated according to social determinants of health. This disaggregation will enable public health interventions to actively identify and support the populations most vulnerable to the effects of climate change. The WHO/UNFCCC Health and Climate Change Country Profiles, developed in collaboration with national health services, are data-driven snapshots of the climate hazards and the expected health impacts of climate change countries are facing. The data from the country profiles can me marge with the survey data to track current policy responses and summarize key priorities for climate and health action by country.
2. In 2022, WHO will publish at least 12 new country profiles for the WHO European Region, and more than half are countries that are not participating in the 2021 survey which was the base for this indicator. We hope that we will extend the number of countries for the indicator for 1/3 with information of countries that report having done a climate change, health vulnerability, and adaptation assessment or report having climate change and health plans based on the data from the countries' profiles.

Additional analysis

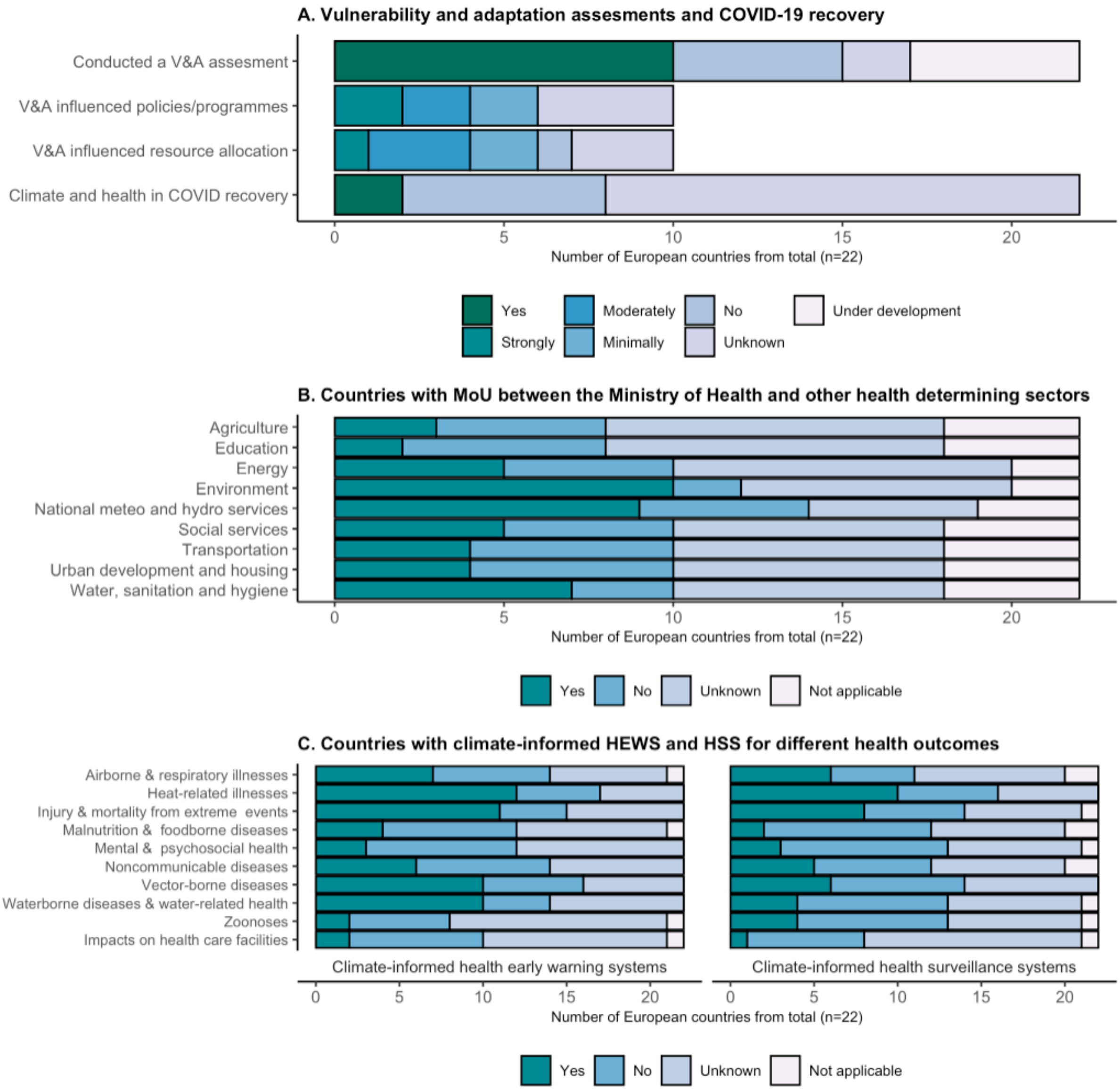

Figure 2.3 Adaptation planning, delivery, and implementation for health in Europe. The data presented in this figure is based on the 2021 WHO Health and Climate Change Survey. **(A)** Countries reporting having conducted a vulnerability and adaptation (V&A) assessment, whether V&A assessments influenced policies/programmes or resource allocation, and whether climate and health were considered in the COVID-19 recovery (indicator 2.1.1). **(B)** Countries reporting having a Memorandum of Understanding (MoU) between the Ministry of Health and other health determining sectors (indicator 2.1.2). **(C)** Countries reporting having a climate-informed health early warning system (HEWS) or health surveillance system (HSS) for different health outcomes (indicator 2.1.1).

Indicator 2.1.3: City-level climate change risks assessments

Geographic coverage of Europe

For this indicator we included cities in countries that are part of CPD Europe: Belgium, Bulgaria, Croatia, Denmark, Finland, France, Georgia, Germany, Gibraltar, Greece, Iceland, Ireland, Italy, Latvia, Lithuania, Monaco, Montenegro, Netherlands (Kingdom of the), Norway, Poland, Portugal, Romania, Russian Federation, Serbia, Slovenia, Spain, Sweden, Switzerland, Türkiye, and the United Kingdom of Great Britain and Northern Ireland.

Data

1. 2022 CPD-ICLEI Annual Cities Survey

Methods

This indicator is based on the annual Carbon Disclosure Project (CPD) and International Council for Local Environmental Initiatives (ICLEI) Cities questionnaire. Data is collected through a CDP-ICLEI Unified Reporting System, in which cities can report their responses on questions regarding city-level climate change mitigation and adaptation. The dataset is updated daily to reflect new submissions, and is publicly accessible through: https://data.cdp.net/browse

The indicator is based on the following parts of the questionnaire.

- Has a climate change risk and vulnerability assessment been undertaken for your city?
- Is your city facing risks to public health or health systems associated with climate change?
    - Health-related risk and vulnerability assessment undertaken.
    - Identify the health issues driven by the selected climate hazard(s).
    - Please identify which vulnerable populations are affected by these climate-related impacts.
    - What factors affect your jurisdiction's ability to address the selected health issues.

Caveats

Participation of cities in this survey is on voluntary basis and self-reported. Hence data captured may be subjective to different forms of bias (i.e., response bias, social desirability bias), and not geographically representable of the entire European region.

Future form of the indicator

Firstly, for further iterations of the Lancet Countdown in Europe report, we hope to combine data from the Global Convent of Mayors for Climate and Energy, and the CPD-ICLEI Annual Cities Survey to capture information on a broader set of cities within Europe. Secondly, we are further planning to perform additional analyses utilising the CPD annual survey to explore a broader set of indicators and monitor associations between city-level health vulnerabilities. Thirdly, in the next iteration of the Europe report we aim to track trends over time.

2.2: Enabling conditions, adaptation delivery and implementation

Indicator 2.2.2: Green space

Geographic Coverage of Europe

The present indicator is provided for all EEA-38 countries except for Türkiye and including the UK. All the maps excluded overseas territories and used the proposed projection, EPSG 3035 (https://epsg.io/3035).

Data

1. *NDVI:* Residential surrounding greenness will be assessed using satellite-derived NDVI. NDVI quantifies photosynthetically active vegetation by measuring the difference between near-infrared (which vegetation strongly reflects) and red light (which vegetation absorbs). NDVI values range from +1.0 to -1.0. Areas of barren rock, sand, or snow usually show very low NDVI values (for example, 0.1 or less). Sparse vegetation such as shrubs and grasslands or senescing crops may result in moderate NDVI values (approximately 0.2 to 0.5). High NDVI values (approximately 0.6 to 0.9) correspond to dense vegetation such as that found in temperate and tropical forests or crops at their peak growth stage. Negative values of NDVI (values approaching -1) correspond mainly to water and ice and snow cover.

   NDVI was derived from the Vegetation Indices (MOD13Q1) product of the Terra Moderate Resolution Imaging Spectroradiometer (MODIS) with 250 m x 250 m resolution.[149] MOD13Q1 16-day composite vegetation indices provide one NDVI value every 16 days allowing the composition of gap-free temporal series. The per-pixel compositing algorithm is described in the MOD13Q1 product user guide (Kamel Didan et al., 2015).[150] The MOD13Q1 product includes the following relevant bands, among others: a) NDVI and b) quality assessment (QA) information. More information could be found elsewhere (https://lpdaac.usgs.gov/products/mod13q1v006/). Per-pixel Quality Assessment (QA) band provides information about how reliable the acquired data is and allows for the removal of pixels with less accuracy. QA values of 0 and 1 mean a good quality level, whereas values of 2 and 3 represent pixels covered by ice, snow or clouds, which are discarded.

   Land Water Mask derived from Landsat[151] was used to filter out the water bodies from the NDVI maps. This mask is well-described satellite imagery with a refined resolution (30m) where each pixel was individually classified into water / non-water using an expert system historically in time. Water features have typically negative signal values for NDVI, so they add noise to the negative edge when conducting spatial analysis.

1. *Population:* Population gridded datasets were obtained for the available years 2006, 2011, 2018 and 2021 at 1 km square resolution from the Eurostat/GISCO.[11] Each grid cell contained the estimated total population count.

2. *NUTS 3:* NUTS 3 administrative layers were obtained for 2021 from the Eurostat/GISCO at 1M resolution.

3. *Social deprivation data:* Material social deprivation rate was obtained stratified at low/medium/high levels for a subset of NUTS 2 regions.

Methods

The two main indicators presented here: "population-weighted NDVI change between 2000 and 2022 at NUTS 3 regions" and the "distribution of NDVI change between 2000 and 2022 among deprivation groups at NUTS 2 level" among other additional analysis presented in the "Additional analysis" section were computed as follows: No population estimates were available for a set of countries (Albania, Croatia, Cyprus, Montenegro, North Macedonia and Serbia) at the 2006 edition, which were imputed with data from 2011.

Green space quantification

We used Google Earth Engine to obtain the final datasets for the years 2000 and 2022.

Briefly, all MODIS Terra product imagery available was obtained each year (one imagery every 16 days). A cleaning process was conducted to remove non-good quality pixels using the Quality Assessment band. In order to identify and remove the influence of water bodies in the analysis, we used the "occurrence" band from the Land Water Mask which defines the frequency with which water was present to mask out areas where water was present for at least 50% of the time. Consequently, areas identified as the water remained as no data in the final datasets.

Finally, all processed imagery was aggregated using the median value at the year level and exported as a 250m resolution surface.

We calculate the mean NDVI for 2000 and 2022 for each grid cell in all the population gridded datasets using spatial analysis techniques in the Python language.
Since water bodies were removed from the satellite NDVI data, some grid cells completely covered by water will remain as no data.

Data assembling

We used the population grid datasets from 2006 and 2021 as our unit of analysis. However, both datasets were incomplete and didn't include all EU countries. We used the 2011 and 2018 population grid datasets to complete the missing information for the main population dataset. Therefore, population stability might be considered in those countries. **Table 2.3** lists the data source used in each dataset.

Each population grid cell from the different datasets was linked to its belonging NUTS 3 region from 2021. Since NUTS 3 administrative areas have changed between 2006 and 2021 we used the latest region distribution to allow data comparability. As a result, we built two grid datasets as follows:

Grid dataset for 2000:

- Total population from 2006 (or 2011 when grid cells were missing, i.e. Balkans countries)
- NUTS 3 region from 2021.
- Country code.
- NDVI average for years 2000 and 2022.

Grid dataset for 2022:

- Total population from 2021 (or 2018 when grid cells were missing, i.e. UK, Balkans countries)
- NUTS 3 region from 2021.
- Country code.
- NDVI mean for years 2000 and 2022.

As explained before, some areas in NDVI surface maps are represented as no data (water bodies) we end up with some missing data associated with population grid cells. To avoid affecting the final indicator expressed as population-weighted, we accounted for these missing cells and distributed the population to be removed among all the rest of the cells within the same NUTS 3 region. Weighted distribution of the population was done at grid cell level (i.e. cells with higher population counts received more than cells with smaller population).

For each grid dataset, data was aggregated at NUTS 3 level and the population-weighted NDVI average was computed for the years 2000 and 2022. This allowed us to perform several comparisons: population and non-population weighted NDVI changes across the time (**Figure 2.2** and **2.3**), distribution of the weighted NDVI across NUTS3 and countries (**Figure 2.5**). Data was also aggregated and compared at European regions (**Figure 2.6**).

Caveats

The 2006 and 2021 population grid dataset were incomplete for some years. Therefore, we had to use the 2011 and 2018 grid datasets to replace the missing cells in the missing countries and rely on the population stability across these years. Table 1 provides detailed information on which data was used for each grid.
Bosnia and Herzegovina and Kosovo (under UNSC resolution 1244) didn't show NUTS 3 data at any time; therefore, the greenness indicator is provided at the country level for these countries.

The population-gridded dataset from 2021 consists of provisional data, as stated in the documentation ("*The Census 2021 grid dataset is provisional. 15 Member States (AT, CY, CZ, DE, EL, ES, HU, IE, IT, LI, LT, LU, PL, RO, SE) flagged their total population grid data as provisional. Final grid dataset on total population in grids will be available in 2024")* (ESMS_Census_Grid 2021.pdf). That might affect the provided population-weighted averaged values and the analysis would need to be re-calculated when the final population data is available.

Table 2.3 Description of available data by country and the population data source used for each grid dataset.

COUNTRIES FOR LCDE 2024 Report - NDVI Indicator				POPULATION GRID DATASETS	
Country name	Eurostat code	ISO 3166-1 Alpha-2 code	EEA sub-region division	2006	2021
Albania	AL	AL	Southern	2011	2018
Andorra		AN	Not EEA	2006	2018
Armenia		AM	Not EEA		
Austria	AT	AT	Western	2006	2021
Azerbaijan		AZ	Not EEA		
Belarus		BY	Not EEA		
Belgium	BE	BE	Western	2006	2021
Bosnia and Herzegovina	BA	BA	Southern	2011	2018
Bulgaria	BG	BG	Eastern	2006	2021
Croatia	HR	HR	Southern	2011	2021
Cyprus	CY	CY	Southern	2011	2021
Czechia	CZ	CZ	Eastern	2006	2021
Denmark	DK	DK	Northern	2006	2021
Estonia	EE	EE	Northern	2006	2021
Finland	FI	FI	Northern	2006	2021
France	FR	FR	Western	2006	2021
Georgia	GE	GE	Not EEA		
Germany	DE	DE	Western	2006	2021
Greece	EL	GR	Southern	2006	2021
Hungary	HU	HU	Eastern	2006	2021
Iceland	IS	IS	Northern	2006	2018
Ireland	IE	IE	Northern	2006	2021
Israel		IL	Not EEA		
Italy	IT	IT	Southern	2006	2021
Kazakhstan		KZ	Not EEA		
Kosovo (UNSCR 1244)	XK		Southern	2011	2018
Kyrgyzstan		KG	Not EEA		
Latvia	LV	LV	Northern	2006	2021
Liechtenstein	LI	LI	Western	2006	2021
Lithuania	LT	LT	Northern	2006	2021
Luxembourg	LU	LU	Western	2006	2021
Malta	MT	MT	Southern	2006	2021
Republic of Moldova	MD	MD	Not EEA		
Monaco		MC	Not EEA		
Montenegro	ME	ME	Southern	2011	2018
Netherlands (Kingdom of the)	NL	NL	Western	2006	2021
North Macedonia	MK	MK	Southern	2011	2018
Norway	NO	NO	Northern	2006	2021

Poland	PL	PL	Eastern	2006	2021
Portugal	PT	PT	Southern	2006	2021
Romania	RO	RO	Eastern	2006	2021
Russian Federation		RU	Not EEA		
San Marino		SM	Not EEA		
Serbia	RS	RS	Southern	2011	2018
Slovakia	SK	SK	Eastern	2006	2021
Slovenia	SI	SI	Southern	2006	2021
Spain	ES	ES	Southern	2006	2021
Sweden	SE	SE	Northern	2006	2021
Switzerland	CH	CH	Western	2006	2021
Tajikistan		TJ	Not EEA		
Türkiye	TR	TR	Southern		
Turkmenistan		TM	Not EEA		
Ukraine	UA	UA	Not EEA		
United Kingdom of Great Britain and Northern Ireland	UK	GB	Not EEA	2006	2018
Uzbekistan		UZ	Not EEA		

Analysis

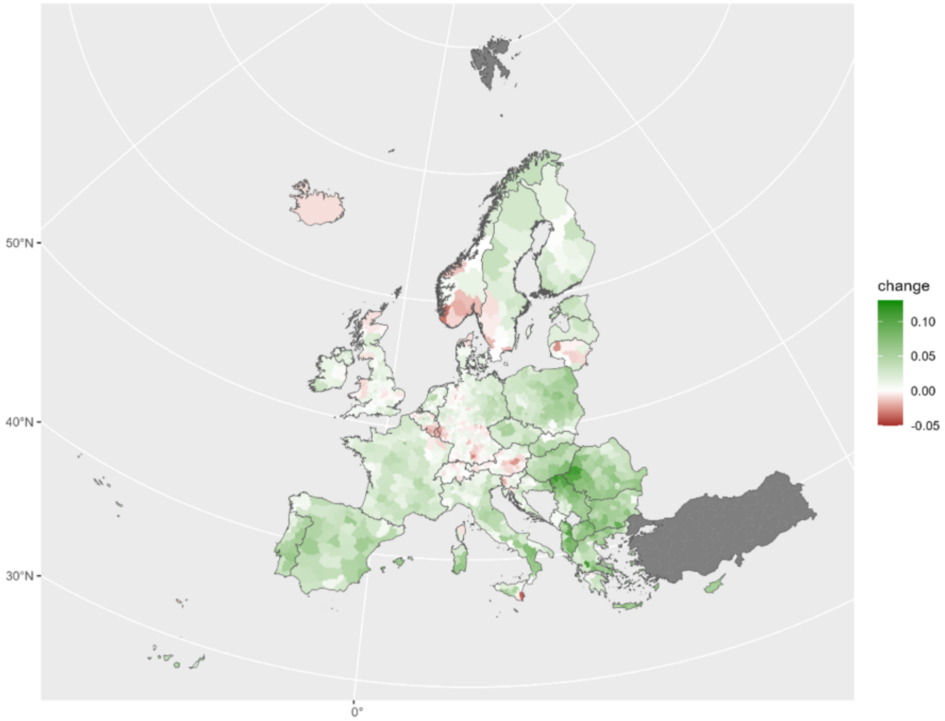

Figure 2.4 Absolute change in population-weighted NDVI (2000-2022) by NUTS3 region.

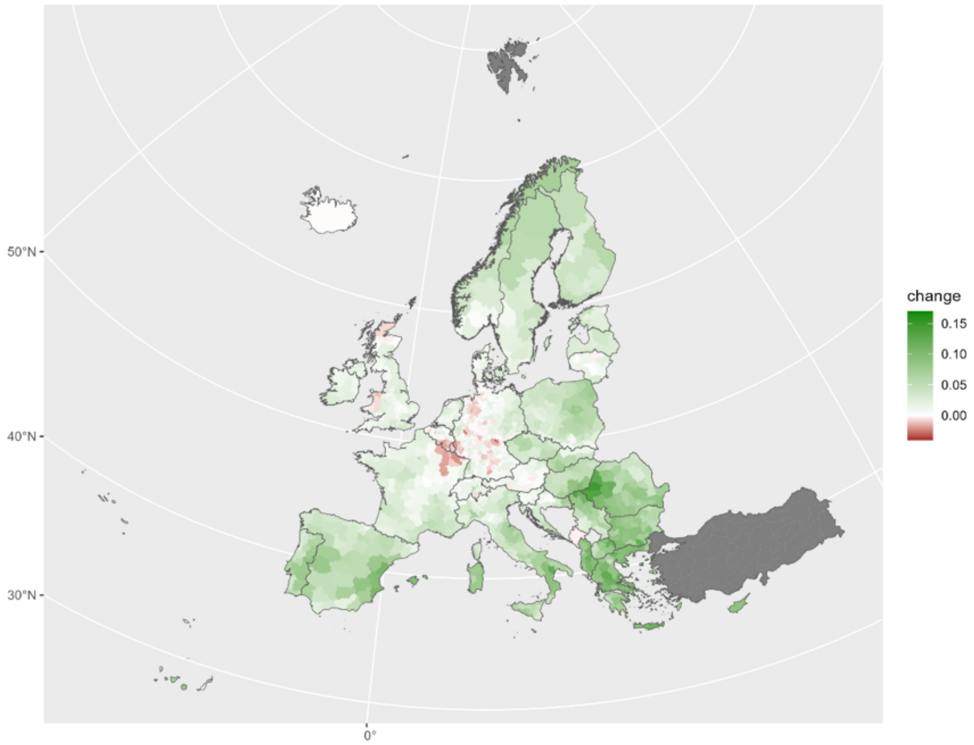

Figure 2.5 Absolute change in NDVI (non-population-weighted) between 2000 and 2022 by NUTS 3

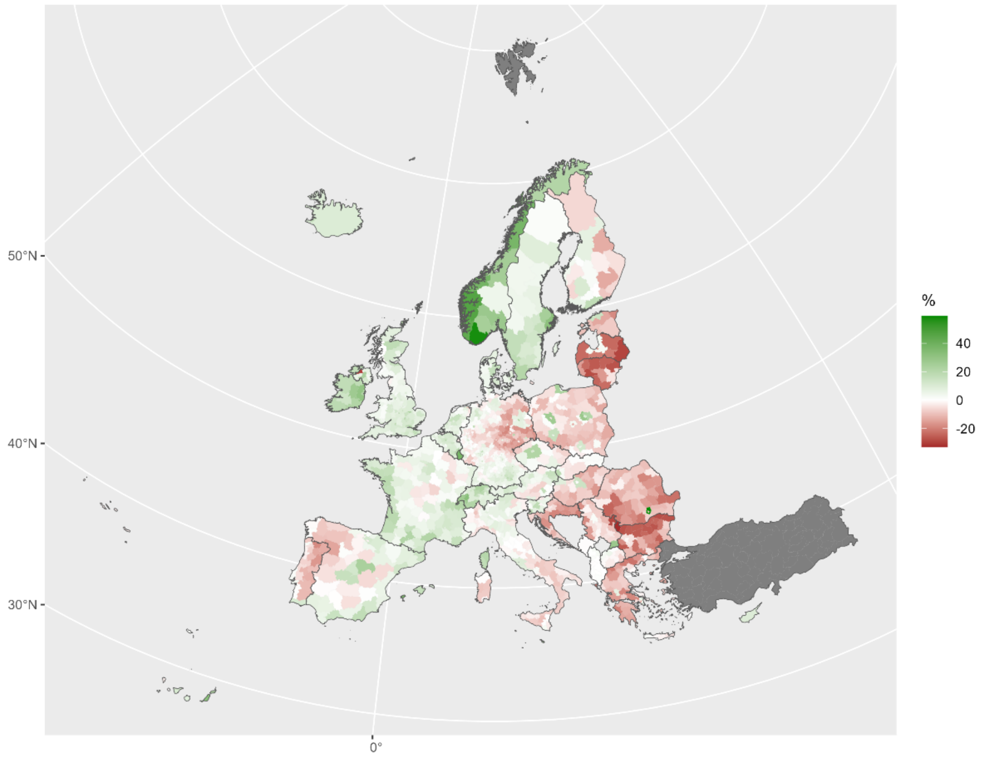

Figure 2.6 Total population percentage change between 2006 and 2021 by NUTS 3 regions

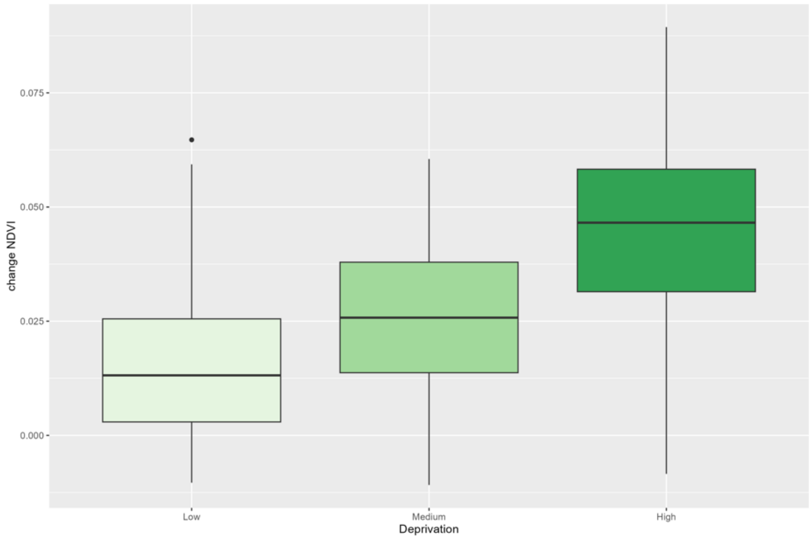

Figure 2.7 Distribution of the population-weighted NDVI change between 2000 and 2022 by social deprivation at NUTS2 level. Only NUTS2 areas with available deprivation data were included.

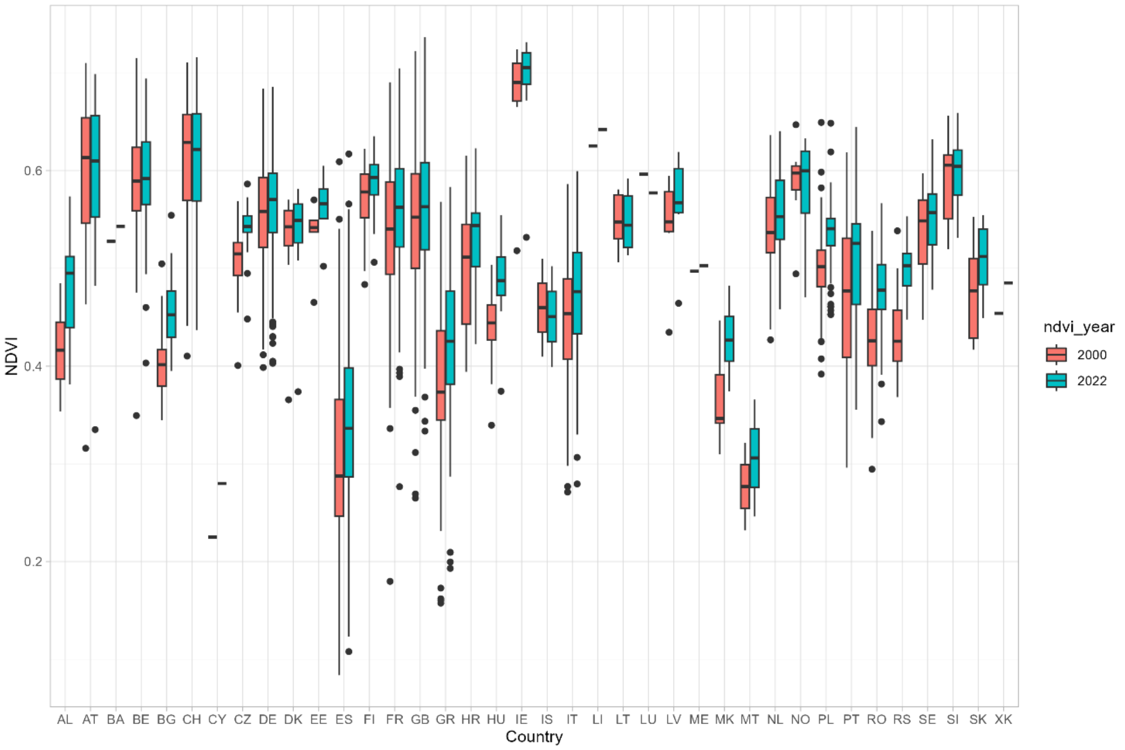

Figure 2.8 Distribution of the population-weighted NDVI for years 2000 and 2022 by NUTS 3 and country level.

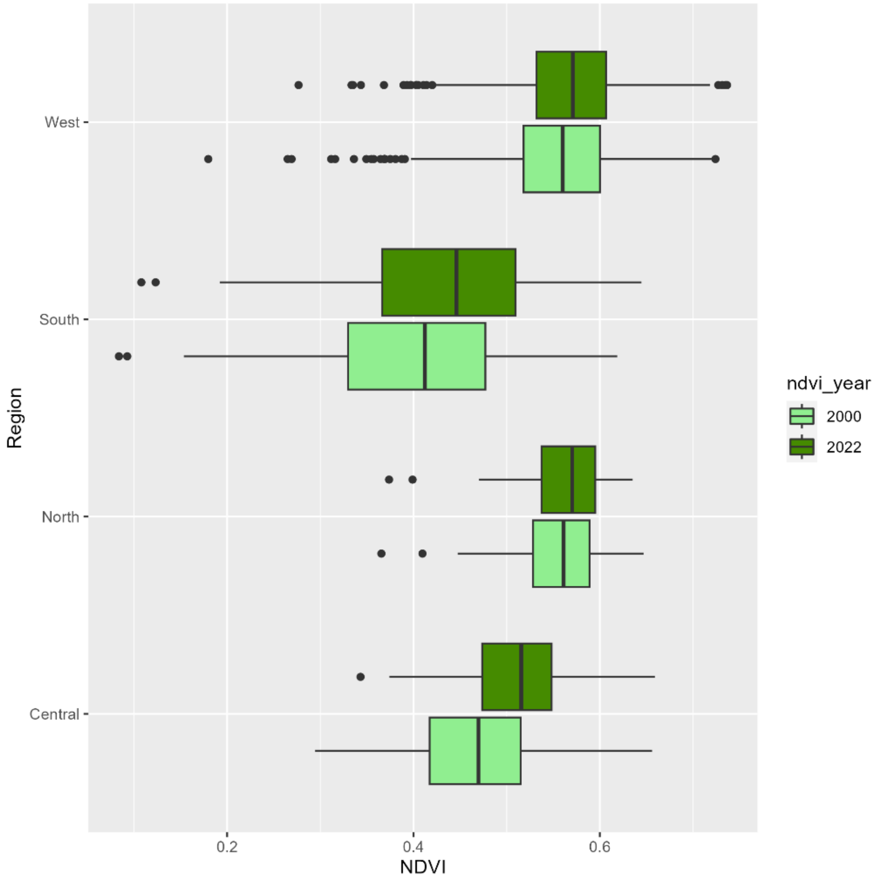

Figure 2.9 Distribution of the population-weighted NDVI for years 2000 and 2022 across the European regions.

Indicator 2.2.3: Air conditioning benefits and harms

Geographic coverage of Europe

This indicator includes: Albania, Austria, Belarus, Belgium, Bosnia and Herzegovina, Bulgaria, Croatia, Cyprus, Czechia, Denmark, Estonia, North Macedonia, Germany, Gibraltar, Greece, Holy See, Hungary, Italy, Iceland, Ireland, Israel, Kosovo (under UNSC resolution 1244), Latvia, Lithuania, Luxembourg, Malta, Republic of Moldova, Monaco, Montenegro, Netherlands (Kingdom of the), Norway, Poland, Portugal, Romania, San Marino, Serbia, Slovakia, Slovenia, Spain, Switzerland, Sweden, Türkiye, Ukraine, United Kingdom of Great Britain and Northern Ireland.

Data, methods, caveats, future form of the indicator

This indicator is based on the data and methods of **indicator 2.2.2** of the global *Lancet* Countdown 2022 Report.

Romanello, M., Di Napoli, C., Drummond, P., Green, C., Kennard, H., Lampard, P., ... & Costello, A. (2022). The 2022 report of the Lancet Countdown on health and climate change: health at the mercy of fossil fuels. *The Lancet*, *400*(10363), 1619-1654.

A full description of the methods, data, caveats and future form of the indicator can be found in the Appendix of the global *Lancet* Countdown 2022 report[16]: https://www.thelancet.com/cms/10.1016/S0140-6736(22)01540-9/attachment/d63703f8-315e-4cdb-9573-1e552e1d4913/mmc5.pdf

Section 3: Mitigation actions and health co-benefits

3.1: Energy system and health

This indicator outlines the fundamental drivers of the relationship between climate change and health, namely the use fossil fuels in the energy system. It comprises three sub-indicators.

Indicator 3.1.1: Carbon intensity of the energy system

Geographic Coverage of Europe

For this indicator, we included the European Environment Agency (EEA) member and cooperating countries plus the United Kingdom of Great Britain and Northern Ireland, for the period 1990-2021.

Data

This sub-indicator is based on the International Energy Agency (IEA) dataset, CO_2 Emissions From Fuel Combustion: CO_2 Indicators, accessed via the OECD data library.[152]

Methods

This sub-indicator contains two metrics:
1. Carbon intensity of the energy system in Europe, (1990-2021), in tCO_2/TJ; and CO_2 emissions from energy combustion by fuel, in $GtCO_2$ (1990-2021).
2. Technical definition is the tonnes of CO_2 emitted for each unit (TJ) of primary energy supplied.

The rationale for the sub-indicator choice is that carbon intensity of the energy system will provide information on the level of fossil fuel use, which has associated air pollution impacts (explored in indicator 3.2). Higher intensity values indicate a more fossil dominated system, and one that is likely to have a higher coal share. As countries pursue climate mitigation goals, the carbon intensity is likely to reduce with benefits for air pollution.

The indicator is calculated based on total CO_2 emissions from fossil fuel combustion divided by Total Energy Supply (TES). TES reflects the total amount of primary energy used in a specific country, accounting for the flow of energy imports and exports. The current rate of reduction of carbon intensity is incompatible with meeting the goals of the European Climate Law and the Paris agreement. Countries with high overall emissions are highlighted in the figure. The remainder of countries are aggregated as “Rest of Europe”. All countries in the EEA are given under the “All of Europe” region.

Inequality Context

The data included for this indicator are not available at a subnational level. Significant differences exist between the carbon emissions of different European countries but relate in complex ways to measures of inequality.

Caveats

The sub-indicator does not provide information on the share of different fossil fuels, their use in different sectors, or the absolute levels of usage. These are all important elements in understanding air pollution emissions and their impacts. Additional sub-indicators (**3.1.2** & **3.1.3**) provide additional complimentary information.

Data for 2021 are preliminary and do not include a full set of countries covered by this indicator. Albania, Bulgaria, Bosnia and Herzegovina, Cyprus, Croatia, Republic of North Macedonia, Malta, Montenegro, Romania, and Serbia are not included. It is likely that these countries do not have a significant impact on the average carbon intensity across the region.

Future Form of the Indicator

The data are updated annually by the IEA. There is currently no expectation that sub-national figures will become available.

Analysis

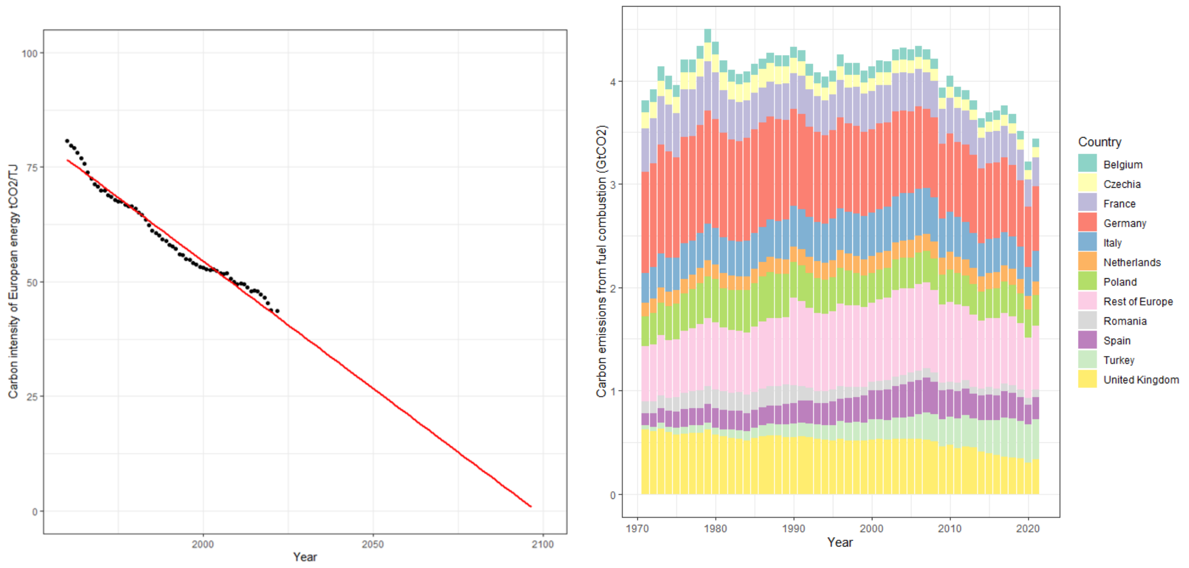

Figure 3.1. (**A**) Carbon intensity of total energy supply in Europe from 1990 to 2021. Red solid line shows extrapolation of current rate of reduction to 2100. (**B**) Carbon emissions from the combustion of fuels 1990-2020 for countries with emissions greater than $0.1GtCO_2$/year.

Indicator 3.1.2: Coal phase-out

Geographic Coverage of Europe

For this indicator, we included the European Environment Agency (EEA) member and cooperating countries plus the United Kingdom of Great Britain and Northern Ireland.

Data

This sub-indicator is based on the extended energy balances from the International Energy Agency. The specific dataset is called World Extended Energy Balances (for 2023),[153] and is sourced via the UK data service.

Methods

This sub-indicator is based on two metrics:

1. Total primary coal supply by country (in exajoules, EJ).
2. Share of electricity generation from coal (% of total generation from coal).

These metrics are important to enable tracking of changes in coal consumption at a regional and country level. As countries pursue climate mitigation goals, the use of coal is likely to reduce with resulting benefits for air pollution. The metric on primary energy coal supply is an aggregation of all coal types used across all sectors (from IEA energy balances). The data are available for all EEA member countries for the period 1990-2019.
The metric on the share of electricity generation from coal is estimated based on electricity generated from coal power plants as a percentage of total electricity generated.
Countries with high overall use of coal are highlighted in the figure. The remainder of countries are aggregated as "Rest of Europe". All countries in the EEA are given under the "All of Europe" region.

Inequality Context

The data included for this indicator are not available at a subnational level. Significant differences exist between the energy usage of different European countries but relate in complex ways to measures of inequality.

Caveats

The sub-indicator does not provide information on the share of different fossil fuels, their use in different sectors, or the absolute levels of usage. These are all important elements in understanding air pollution emissions and their impacts.

Future Form of the Indicator

The data are updated annually by the IEA. There is currently no expectation that sub-national figures will be available.

Analysis

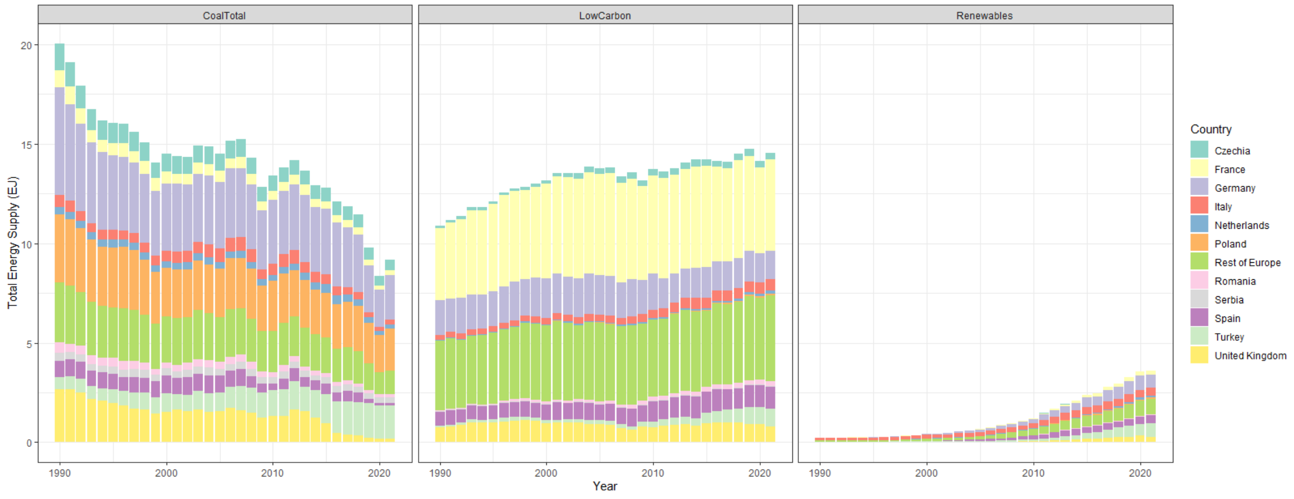

Figure 3.2 Total energy supply (EJ) in Europe between 1990-2021 provided by (A) coal (b) low-carbon sources (renewables with the addition of hydroelectricity and nuclear) and (B) renewables (wind, solar, geothermal and tidal power). Zero-carbon energy sources are those which have effectively no carbon emissions associated with their production operations.

Indicator 3.1.3: Renewable and zero-carbon emission electricity

Geographic Coverage of Europe

For this indicator, we included the European Environment Agency (EEA) member and cooperating countries plus the United Kingdom of Great Britain and Northern Ireland.

Data

This sub-indicator is based on the extended energy balances from the IEA. The specific dataset is called World Extended Energy Balances,[153] and is sourced via the UK data service.

Methods

This sub-indicator comprises two metrics:
1. Total low carbon electricity generation, (including nuclear, and all renewables); and
2. Total renewable generation (wind, solar PV and solar thermal, geothermal), as % share of total electricity generated.

The increase in the use of low carbon and renewable energy for electricity generation will push other fossil fuels, such as coal, out of the mix over time, resulting in improved air quality and associated health benefits.
The renewables indicator has been used to allow for the tracking of rapidly emergent renewable technologies. For both metrics, generation, rather than capacity, has been used as the electricity generated from these technologies is what actually displaces fossil-based generation.

Data are based on the IEA extended energy balances. The absolute level data are total gross electricity generated aggregated from the relevant technology types. The share data are estimated as the low carbon or renewable generation as a % of total generation.

The data are available for most countries, for the period 1971-2021. Only the period from 1990 has been used, due to data gaps for selected countries prior to 1990.

Inequality Context

The data included for this indicator are not available at a subnational level. Significant differences exist between the energy usage of different European countries but relate in complex ways to measures of inequality.

Caveats

This sub-indicator does not provide information on the air pollutant emissions displaced due to the increasing share of renewable energy generation.

Future Form of the Indicator

The data are updated annually by the IEA. There is currently no expectation that sub-national figures will become available.

Analysis

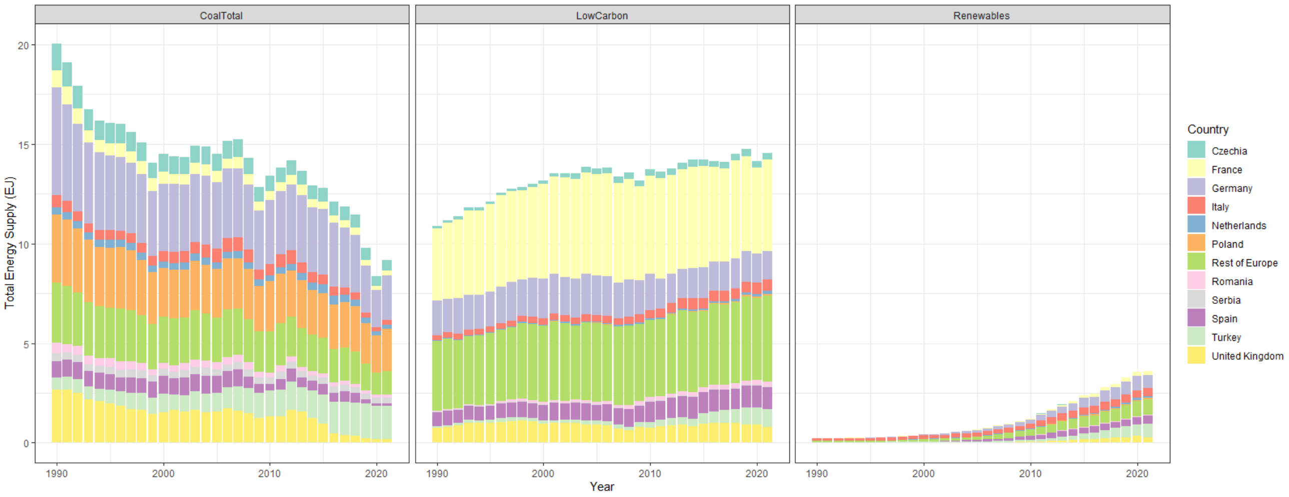

Figure 3.2 Total energy supply (EJ) in Europe between 1990-2021 provided by (A) coal (b) low-carbon sources (renewables with the addition of hydroelectricity and nuclear) and (B) renewables (wind, solar, geothermal and tidal power). Zero-carbon energy sources are those which have effectively no carbon emissions associated with their production operations.

3.2: Air pollution and health co-benefits

Indicator 3.2.1: Premature mortality attributable to ambient fine particles

Geographic Coverage of Europe

The indicator covers 45 countries: EU-27, UK, Norway, Switzerland, Iceland, West Balkan, Eastern Europe (Ukraine, Belarus, Republic of Moldova, European part of the Russian Federation), Caucasus (Armenia, Georgia, Azerbaijan). The temporal resolution available at the moment is in five-year steps from 2005 to 2020.

Data

- Energy consumption by fuel and sector, industrial production: Eurostat,[154] IEA energy statistics[155] for countries missing in Eurostat
- Other activities:
  - Agricultural activities: Food and agriculture organization FAOSTAT[156]
  - Fertilizer use: IFASTAT[157]
  - Municipal waste, other minor sources: GAINS internal calculations

Mortality data: Eurostat. Data gaps for individual countries and years were filled with UN World Population Prospects 2017 estimates

Methods

This indicator reports estimates of mortality attributable to long-term exposure to $PM_{2.5}$ originating from different fuels and sectors. It relies on calculations with the Greenhouse Gas-Air Pollution Interactions and Synergies (GAINS) model,[158] which combines bottom-up emission calculations with atmospheric chemistry and dispersion coefficients. Although GAINS covers all economic sectors, the focus for this indicator is on households, electricity generation, and transport.

Data on energy consumption and other activities have been imported into GAINS from Eurostat data and mapped to detailed sectors in GAINS. They are then merged with GAINS internal information on application of emission control technologies in each country and year, reflecting the appropriate fleet/stock composition and legislation at that time. Each technology is associated with country-specific emission factors to calculate emissions of $PM_{2.5}$ and PM precursor gases SO_2, NOx, NH_3, and non-methane VOC.

Ambient $PM_{2.5}$ concentrations are calculated from the region and sector specific emissions by applying atmospheric transfer coefficients, which are a linear approximation of full chemistry-transport models. Calculations include both primary PM as well as secondary inorganic and organic aerosols. Atmospheric transfer coefficients in GAINS-Europe are based on full year perturbation simulations with the EMEP Chemistry Transport Model[159] run at a resolution of 0.5°×0.25° and include a downscaling for low-level emission sources of primary PM (residential emissions, road traffic and non-road machinery) to a resolution of 0.125°×0.0625° or roughly 7×7km based on a full-year simulation with the CHIMERE CTM.[160]

GAINS atmospheric calculations are described in detail by Kiesewetter et al. (2015). For the Lancet Countdown indicator, contributions from individual (aggregated) source sectors such as road traffic, power plants, industry, and households, as well as from different fuel types (coal, liquids, gas, biomass, others) used in each sector, are kept separate in the calculation.

Attributable deaths are calculated through a comparative risk assessment framework. GAINS uses a linear approximation of the attributable fraction calculation,

$$AF_{csf} = \beta\ [PM]_{csf}$$

where AF_{csf} is the attributable fraction in country c from sector s and fuel f, $[PM]_{csf}$ is the population-weighted mean concentration of $PM_{2.5}$ in country c from emissions of sector s and fuel f, and β is the risk coefficient of the linearized concentration-response function. Here we use the concentration-response function reported by the systematic review by Chen and Hoek 2020[161] which found a relative risk for natural-cause mortality from long-term exposure to $PM_{2.5}$ of 1.08 per 10μgm^{-3}, thus the coefficient $\beta = 0.008/\mu gm^{-3}$. Attributable deaths ad are calculated as

$$ad_{csf} = AF_{csf}\ nd_c$$

with nd_c the total natural-cause deaths over 30 years of age in country c.

In this edition of the Lancet Countdown for Europe, we have extended the calculations to include a disaggregation of estimated trends in $PM_{2.5}$ concentrations and attributable mortality into three main contributing factors:

(i) Temporal change in energy intensity, that is, the Energy consumed per unit of economic output (GDP), which determines the size of energy demand, structure of energy services, and reflects differences in socioeconomic structures, as well as in behavioural patterns. Energy intensity is complemented by the impacts of efficiency improvements of the energy system, in other words, the efficiency at which primary energy is converted into secondary and final energy.

(ii) The evolution of the fuel mix of different energy forms affects emission intensities, comprising inter-fossil-fuel switch and changes in the fraction of non-fossil fuels in energy supply. Substitution of traditional/combustible fuels by electricity and district heating contributes to this mitigation component on the demand side of the energy system. This component does not apply to the (non-combustion) industrial process activities, and the potential impacts of the innovative techniques (e.g. direct reduced iron (DRI) with H2) would be indirectly covered in factor (i).

(iii) The changes in aggregated emission factors over time which typically follow the implementation of end-of-pipe measures and fuel quality standards. The resulting emission coefficient reflects the removal efficiency (eff) of a given abatement measure adopted at a specific rate (ΔX).

Following the methodology introduced by Rafaj *et al.,*[162] we construct four scenarios between 2005 and 2020 to derive these contributing factors:

1. A hypothetical scenario in which the energy consumption follows macroeconomic drivers that seem most appropriate for a given sector. For the power sector, we use GDP, for transport GDP per capita, and for residential combustion population as a driver. The composition of fuels consumed remains fixed to the base year consumption (no fuel shifts), and emission factors for each fuel remain constant as well (no technological improvement)

2. A hypothetical scenario in which the total energy consumption within each sector equals the real (statistical) energy consumption but the share of fuels used remains fixed to that of the base year. Also the emission factors remain fixed to base year.
3. A hypothetical scenario in which the energy consumption within each sector equals the real (statistical) energy consumption for each fuel but the emission factors for each fuel remain constant.
4. A scenario using both the real (statistical) fuel consumption within each sector and fuel type, as well as the real emission factors including changes in control technologies over time. This scenario describes our best understanding of how the emissions and concentrations from each sector changed over time.

For each scenario 1-4 and for each sector, the GAINS model was run to calculate emissions of all $PM_{2.5}$ precursors, ambient $PM_{2.5}$ concentrations, and attributable mortality. Contributions from different factors are then diagnosed as differences between different scenarios.

Differences between scenario 1 and 2 represent changes due to structural changes in the economy and energy efficiency improvements, so that the total energy demand in a given sector deviated from the trend of the relevant macroeconomic driver. Differences between scenario 2 and 3 represent changes due to fuel switches while the total energy demand is the same. Differences between scenarios 3 and 4 represent changes due to end-of-pipe emission control technologies.

The methodology described here has been used for disaggregating trends in sectoral emissions of CO_2 and air pollutants by Rafaj *et al.*[162] We extend it here to ambient $PM_{2.5}$ concentrations and attributable mortality.

Inequality Context

Although air pollution has an inequality context, particularly in the context of residential solid fuel burning, this aspect has not been further analysed in this indicator.

Caveats

- The indicator relies on model calculations which are inherently uncertain and use linear approximations for atmospheric processes which are partly non-linear. See Amann *et al.* 2012[158] for a discussion of the linearity approximations.
- Meteorological conditions vary from year to year and are one important factor for inter-annual variability of ambient $PM_{2.5}$ concentrations. The indicator does not consider this variability and reports only trends attributable to emission changes. Atmospheric coefficients in GAINS are based on CTM simulations for five different meteorological years (2006-2010) in order to represent a reasonable average of meteorological conditions.
- The spatial distribution of emissions is fixed to the base year
- Concentration-response functions used in the attributable mortality calculation are based on the relative risk reported by Chen & Hoek (2020). This systematic review and meta-analysis included studies across the globe through 2018 and was conducted in the context of the revision of the WHO Ambient Air Quality Guidelines. The resulting RR=1.08 per 10 μgm^{-3} was used in recent analysis by the European Commission[163,164] and the European Environment Agency.[165] More recent findings from the ELAPSE cohorts which are more representative of European exposure and population contexts,[166,167] have

indicated higher relative risk (RR=1.13 per 5µgm^{-3}) which would increase the attributable mortality results of this study by more than a factor of 2.

- The factor disaggregation relies on hypothetical scenarios and assumptions. Particularly the factor relating to structural changes and energy efficiency improvements is uncertain because it depends on the macroeconomic driver selected for hypothetical scenario 1. Furthermore, the magnitude of the factors depends on the sequence in which the analysis is done. Here we follow the logic and sequence of Rafaj *et al.*[162]

Future Form of the Indicator

- Atmospheric calculations in the GAINS model have been updated recently. In the next iteration of the Lancet Countdown, these will allow for improved estimation of sectoral contributions to ambient PM2.5 and related health impacts.
- The spatial domain of the GAINS-Europe model will be extended with the new atmospheric calculations to include also Central Asian countries: Kazakhstan, Turkmenistan, Uzbekistan, Tajikistan, Kyrgyzstan.
- More sectors like agriculture could be included
- While the temporal resolution can theoretically be extended and improved to cover also in-between years, the import of individual statistical years into the GAINS model is time consuming and it is yet unclear to which extent this will be possible within the Lancet Countdown for Europe. Importing statistics from earlier years poses an additional challenge, as structure and national borders changed over time.
- It is not clear yet whether the trend attribution analysis undertaken in this edition will become a regular element of the indicator or the indicator will report total mortality attributable to different emission sectors and fuels as in Lancet Countdown Europe 2022 (see also Additional Analysis below).

Additional analysis

Figure 3.3 shows the indicator results for the whole domain: (a) total mortality attributable to $PM_{2.5}$ concentrations caused by each sector, and (b) the disaggregation of factor contributions to the trends.

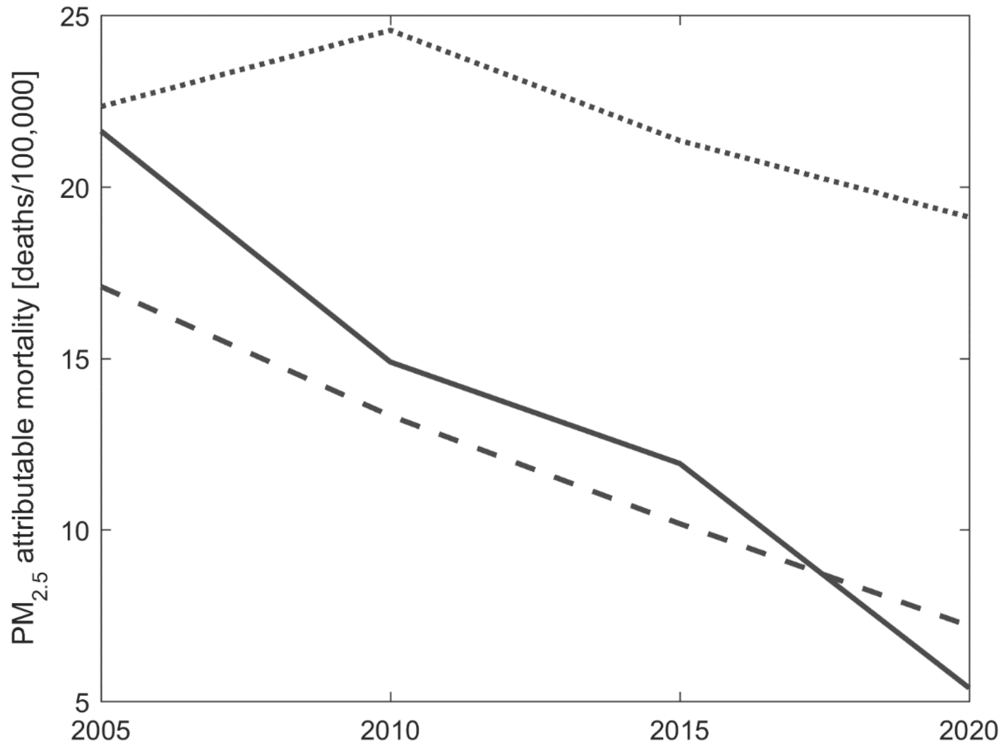

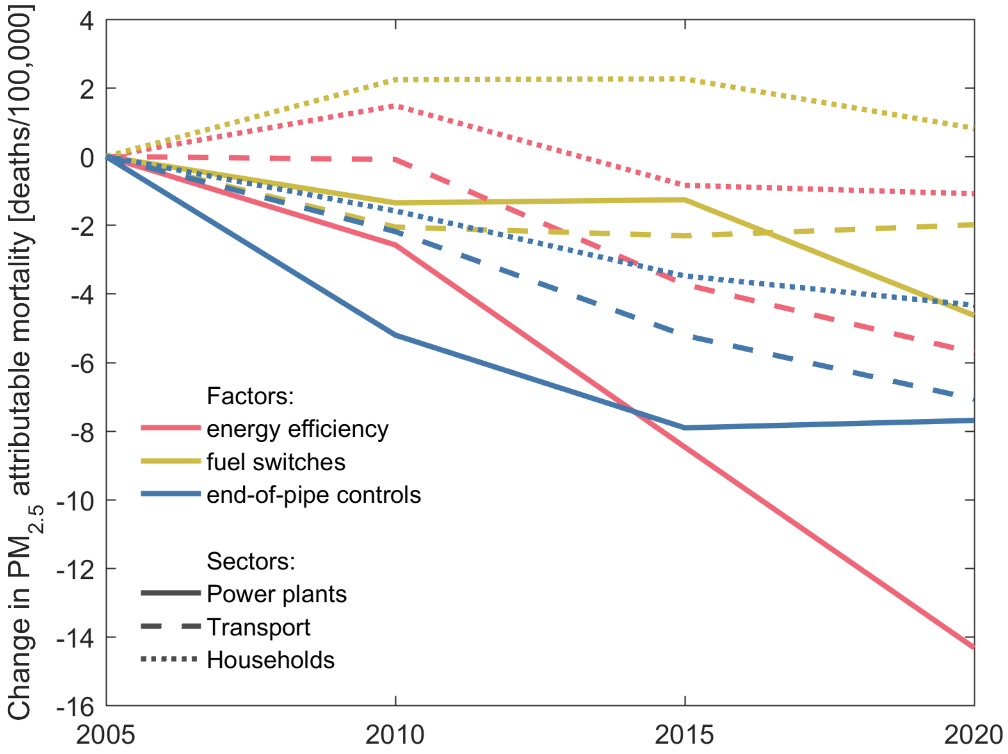

Figure 3.3 (A) $PM_{2.5}$ attributable mortality per 100,000 population in Europe between 2005 and 2020 according to sector; **(B)** change in $PM_{2.5}$ attributable mortality per 100,000 population in Europe between 2005 and 2020 according to driver of change and sector.

Indicator 3.2.2: Production-based and consumption-based attribution of CO_2 and $PM_{2.5}$ emissions

Geographic coverage of Europe

The indicator covers: Austria, Belgium, Bulgaria, Cyprus, Czechia, Germany, Denmark, Estonia, Spain, Finland, France, Greece, Croatia, Hungary, Ireland, Italy, Lithuania, Luxembourg, Latvia, Malta, Netherlands (Kingdom of the), Poland, Portugal, Romania, Sweden, Slovenia, Slovakia, United Kingdom.

Data, methods, caveats, future form of the indicator

This indicator is based on the data and methods of **indicator 4.2.5** of the global *Lancet* Countdown 2022 Report.

Romanello, M., Di Napoli, C., Drummond, P., Green, C., Kennard, H., Lampard, P., ... & Costello, A. (2022). The 2022 report of the Lancet Countdown on health and climate change: health at the mercy of fossil fuels. *The Lancet*, *400*(10363), 1619-1654.

A full description of the methods, data, caveats and future form of the indicator can be found in the Appendix of the global *Lancet* Countdown 2022 report[16]: https://www.thelancet.com/cms/10.1016/S0140-6736(22)01540-9/attachment/d63703f8-315e-4cdb-9573-1e552e1d4913/mmc5.pdf

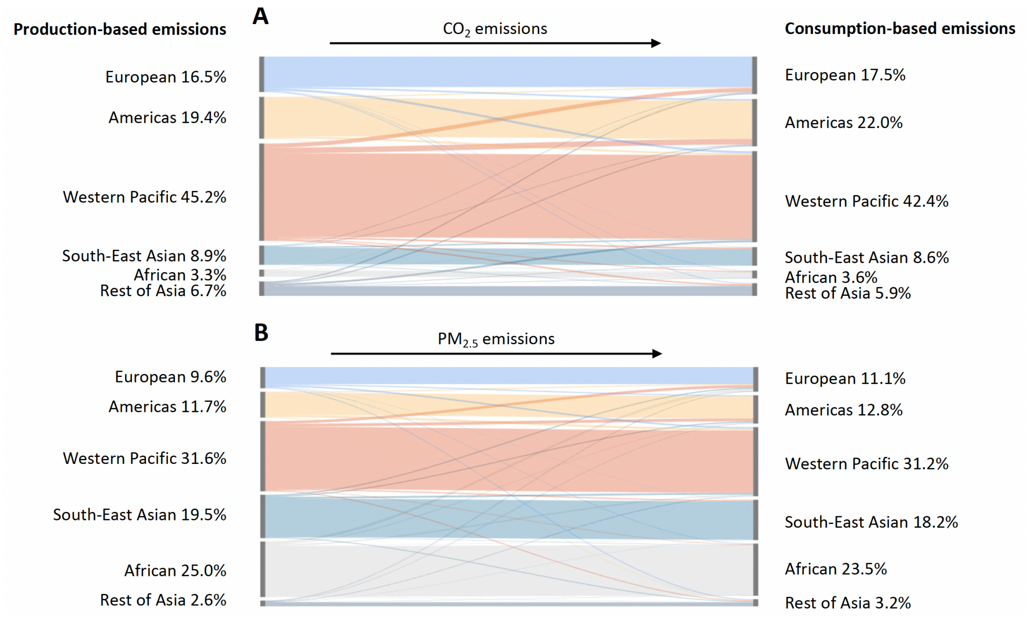

Figure 3.4 (A) CO_2 and (**B**) $PM_{2.5}$ emissions emitted in the production of goods and services traded between world regions in 2021 (i.e., production-based and consumption-based emissions.

3.3: Sustainable and healthy transport

Geographic Coverage of Europe

For this indicator, we included the European Environment Agency (EEA) member and cooperating countries plus the United Kingdom of Great Britain and Northern Ireland.

Data

1. Fuel use data is from the IEA, category road transportation from World Extended Energy Balances
2. UN Population estimates, 2019 edition
3. Modal split of passenger transport

REFS:

1) IEA World Energy Statistics and Balances 2023.[153]
2) UN population prospects 2019.[168]
3) Modal split of passenger transport, 2022, EUROSTAT[169]

Methods

This indicator is comprised of two metrics:

1. Per capita fuel use on road transport data (by fuel type) from the IEA World Extended Energy Balances are divided by corresponding population statistics from the UNDP.
2. Mode share by country over time (2010 to 2020, 2020 shown in figure) by passenger-kilometres, provided by EUROSTAT.

Inequality Context

This indicator does not examine inequality.

Caveats

Harmonised, comparable data on active transport modes (e.g., walking and cycling) are not available at the country level, limiting feasibility of tracking progress on healthy, low-carbon transport.

Metric 1 of this indicator captures change in total fuel use and type of fuel use for transport, but it does not capture shifts in modes of transport used.

Metric 2 captures mode share for passenger vehicle trips but does not capture active travel such as walking and cycling for short trips, which can yield substantial health benefits through increased physical activity.
Further methodological information about how modal share is calculated is available here https://data.europa.eu/data/datasets/ebut9gyyopgfvhrlwojq?locale=en

Future Form of the Indicator

The data are updated annually by the IEA and EUROSTAT. There is currently no expectation that sub-national figures will be available, but a possibility of including regional (NUTS 2) survey data for modal-share exists.

Analysis

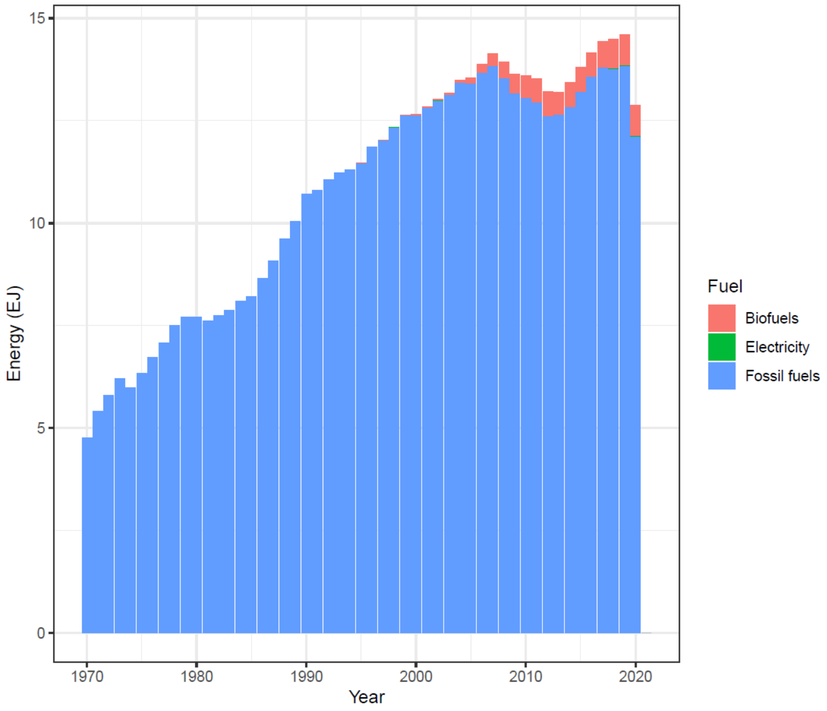

Figure 3.5 Energy use for road transport across Europe, 1970 – 2020.

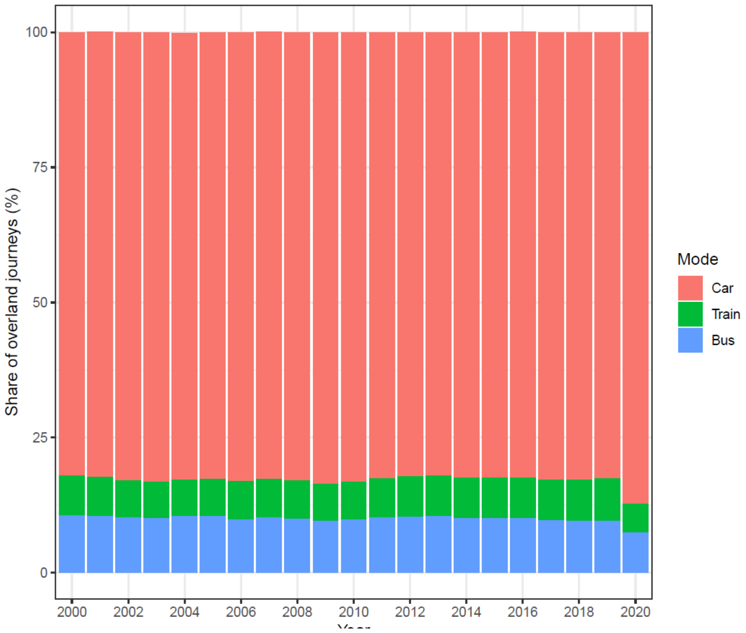

Figure 3.6 Share of journeys by mode across EUROSTAT countries, 2000 to 2020.

3.4: Food, agriculture and health

Indicator 3.4.1: Life cycle emissions from food demand, production and trade

Geographic Coverage of Europe

For this indicator, we used the M49 classification of Europe of the United Nations Statistical Division.

Data

The data sources for this indicator are:

- Dietary exposure from combination of the Global Dietary Database (GDD)[170] released by Tufts University and food availability data from Food Balance Sheets (FBS)[171] released by the Food and Agriculture Organization of the UN, adjusted for waste at the household level
    - Publicly accessible; GDD data[170] available from 1990 to 2018 with uncertain intervals for updating; FBS data[171] is updated regularly and available as far back as 1960
- Life-cycle estimates of emissions footprints of food commodities from a comprehensive meta-analysis of life-cycle assessments (Poore and Nemecek, Science 2018)[172]
    - The estimates have been regionalised by the authors and made available; they will be updated regularly as part of the HESTIA database[173] and project.

Methods

Estimates of life-cycle emissions per food group and region will be paired with estimates of food demand by food group and country. Life-cycle estimates will be taken from a comprehensive meta-analyses,[172] and estimates of food demand will be taken from FAO's Food Balance Sheets[171] which are updated annually. Additional socio-demographic detail will be adapted from the Global Dietary Database.[170]

Inequality Context

The indicator reflects inequalities in life-cycle emissions associated with food demand between, but not within countries.

Caveats

The indicator captures the full emissions footprints of food demand and therefore is more comprehensive in coverage than emissions indicators that are linked to only one step in the food chain (e.g., agricultural production) or only include a limited set of greenhouse gas species (e.g., methane and nitrous oxide).

Life-cycle estimates are highly context-dependent. The analysis will be based on a meta-analysis of life-cycle assessments to address this, but there are large uncertainties for food groups and regions for which few individual assessments are available. Aspects of improvements in farm management over time are not reflected in the estimates of life-cycle emissions, because those are provided only for one harmonised time point so far.

Future Form of the Indicator

The estimates of life-cycle footprint will be updated as new data becomes available.

Analysis

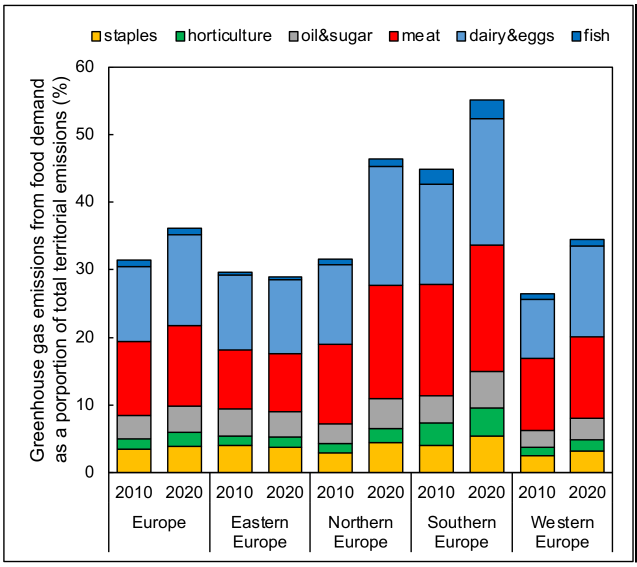

Region	Year	Total emissions (MtCO2-eq)	Food demand (MtCO2-eq)	Proportion of total (%)
Europe	2010	6,302	1,871	30%
	2019	5,417	1,855	34%
Eastern Europe	2010	2,492	680	27%
	2019	2,535	677	27%
Nothern Europe	2010	908	271	30%
	2019	639	280	44%
Southern Europe	2010	1,012	440	44%
	2019	745	400	54%
Western Europe	2010	1,890	480	25%
	2019	1,499	498	33%

Figure 3.7 Greenhouse gas emissions from food demand in absolute terms (**left**) and as a proportion of total territorial emissions (%) by region, year, and food group (**right**).

Indicator 3.4.2: Sustainable diets

Geographic Coverage of Europe

For this indicator, we used the M49 classification of Europe of the United Nations Statistical Division**.**

Data

Table 3.1. Overview of data sources.

Type	Coverage	Source
Exposure data:		
Food consumption data	Country-level	Food availability data adjusted for food waste at the household level and for age and sex-specific trends.[171,174,170] Estimates of energy intake were in line with trends in body weight across countries.[175]
Weight estimates	Country-level	Baseline data from pooled analysis of measurement studies differentiated by sex and age with global coverage.[175]
Health analysis:		
Relative risk estimates	General	Adopted from meta-analysis of prospective cohort studies.[176,177,178,179,180,181,182] The certainty of evidence for the risk-disease associations were rated as moderate to high by NutriGrade.[179,180,181]
Mortality and population data	Country-level	Adopted from the Global Burden of Disease project by country, sex, and age group.[183]

Methods

Baseline consumption data

We estimated baseline food consumption by adopting estimates of food availability from the FAO's food balance sheets, and adjusting those for the amount of food wasted at the point of consumption.[171,174] We disaggregated this proxy for food consumption by age and sex by adopting the same age and sex-specific trends as observed in dietary surveys.[170]

An alternative would have been to rely on a set of consumption estimates that has been based on a variety of data sources, including dietary surveys, household budget and expenditure surveys, and food availability data.[184,185] However, neither the exact combination of these data sources, nor the estimation model used to derive the data have been made publicly available. For some individual countries, using dietary surveys would also have been an alternative. However, underreporting is a persistent problem in dietary survey,[186,187] and regional differences in

survey methods would have meant that our results would not be comparable between countries. In contrast to dietary surveys, waste-adjusted food-availability estimates indicate levels of energy intake per region that reflect differences in the prevalence of overweight and obesity across regions.[175]

Food balance sheets report on the amount of food that is available for human consumption.[171] They reflect the quantities reaching the consumer, but do not include waste from both edible and inedible parts of the food commodity occurring in the household. As such, the amount of food actually consumed may be lower than the quantity shown in the food balance sheet depending on the degree of losses of edible food in the household, e.g. during storage, in preparation and cooking, as plate-waste, or quantities fed to domestic animals and pets, or thrown away.

We followed the waste-accounting methodology developed by the FAO to account for the amount of food wasted at the household level that was not accounted for in food availability estimates.[174] **Table 3.2** provides and overview of the parameters used in the calculation.

For each commodity and region, we estimated food consumption by multiplying food availability data with conversion factors (*cf*) that represent the amount of edible food (e.g. after peeling) and with the percentage of food wasted during consumption (*1-wp(cns)*). For roots and tubers, fruits and vegetables, and fish and seafood, we also accounted for the differences in wastage between the proportion that is utilised fresh (pct_{frsh}) and the proportion that utilised in processed form (pct_{prcd}). The equation used for each food commodity and region was:

$$Consumption = Availability \cdot \frac{pct_{frsh}}{100} \cdot cf_{frsh} \cdot \left(1 - \frac{wp(cns_{frsh})}{100}\right)$$
$$+ Availability \cdot \frac{pct_{prcd}}{100} \cdot cf_{prcd} \cdot \left(1 - \frac{wp(cns_{prcd})}{100}\right)$$

Table 3.2 Percentage of food wasted during consumption (cns), and percentage of processed utilisation (pctprcd). The percentage of fresh utilisation is calculated as 1-pctprcd. Conversion factors to edible portions of foods are provided below the table.

Food group	Item	Region						
		Europe	USA, Canada, Oceania	Indus-trialized Asia	Sub-Saharan Africa	North Africa, West and Central Asia	South and Southeast Asia	Latin America
cereals	wp(cns)	25	27	20	1	12	3	10
roots and tuber	pctprcd	73	73	15	50	19	10	80
	wp(cns)	17	30	10	2	6	3	4
	wp(cnsprcd)	12	12	12	1	3	5	2
oilseeds and pulses	cns	4	4	4	1	2	1	2
fruits and vegetables	pctprcd	60	60	4	1	50	5	50
	wp(cns)	19	28	15	5	12	7	10
	wp(cnsprcd)	15	10	8	1	1	1	1
milk and dairy	wp(cns)	7	15	5	0.1	2	1	4
eggs	wp(cns)	8	15	5	1	12	2	4
meat	wp(cns)	11	11	8	2	8	4	6
fish and seafood	pctprcd	*40% for low-income countries, and 96% for all others.*						
	wp(cns)	11	33	8	2	4	2	4
	wp(cnsprcd)	10	10	7	1	2	1	2

Conversion factors: maize, millet, sorghum: 0.69; wheat, rye, other grains: 0.78; rice: 1; roots: 0.74 (0.9 for industrial processing); nuts and seeds: 0.79; oils: 1; vegetables: 0.8 (0.75 for industrial processing); fruits: 0.8 (0.75 for industrial processing); beef: 0.715; lamb: 0.71; pork: 0.68; poultry: 0.71; other meat: 0.7; milk and dairy: 1; fish and seafood: 0.5; other crops: 0.78

Comparative risk assessment

We estimated the mortality and disease burden attributable to dietary and weight-related risk factors by calculating population impact fractions (PIFs) which represent the proportions of disease cases that would be avoided when the risk exposure was changed from a baseline situation to a counterfactual situation. For calculating PIFs, we used the general formula:[188,189,190]

$$PIF = \frac{\int RR(x)P(x)dx - \int RR(x)P'(x)dx}{\int RR(x)P(x)dx}$$

where $RR(x)$ is the relative risk of disease for risk factor level x, $P(x)$ is the number of people in the population with risk factor level x in the baseline scenario, and $P'(x)$ is the number of people in the population with risk factor level x in the counterfactual scenario. We assumed that changes in relative risks follow a dose-response relationship,[189] and that PIFs combine multiplicatively, i.e. $PIF = 1 - \prod_i (1 - PIF_i)$ where the i's denote independent risk factors.[189,191]

The number of avoided deaths due to the change in risk exposure of risk i, $\Delta deaths_i$, was calculated by multiplying the associated PIF by disease-specific death rates, DR, and by the number of people alive within a population, P:

$$\Delta deaths_i(r,s,a,d) = PIF_i(r,s,a,d) \cdot DR(r,s,a,d) \cdot P(r,s,a)$$

where PIFs are differentiated by region *r*, sex *s*, age group *a*, and disease/cause of death *d*; the death rates are differentiated by region, sex, age group, and disease; the population groups are differentiated by region, sex, and age group; and the change in the number of deaths is differentiated by region, sex, age group, and disease.

We used publicly available data sources to parameterize the comparative risk analysis. Mortality and population data were adopted from the Global Burden of Disease project.[183] Baseline data on the weight distribution in each country were adopted from a pooled analysis of population-based measurements undertaken by the NCD Risk Factor Collaboration.[175]

The relative risk estimates that relate the risk factors to the disease endpoints were adopted from meta-analyses of prospective cohort studies for dietary and weight-related risks.[176,177,178,179,180,181,182] In line with the meta-analyses, we included non-linear dose-response relationships for fruits, vegetables, and nuts and seeds, and assumed linear dose-response relationships for the remaining risk factors. As our analysis was primarily focused on mortality from chronic diseases, we focused on adults aged 20 year or older, and we adjusted the relative-risk estimates for attenuation with age based on a pooled analysis of cohort studies focussed on metabolic risk factors,[192] in line with other assessments.[190,193]

Table 3.3 provides an overview of the relative-risk parameters used. For the counterfactual scenario, we defined minimal risk exposure levels (TMRELs) as follows: 300 g/d for fruits, 500 g/d for vegetables, 100 g/d for legumes, 20 g/d for nuts and seeds, 0 g/d for red meat, and no underweight, overweight, or obesity. The TMRELs are in line with those defined by the Nutrition and Chronic Diseases Expert Group (NutriCoDE),[193] with the exception that we used a higher value for vegetables, and we used zero as minimal risk exposure for red meat, in each case based on a more comprehensive meta-analysis.[178,179]

The selection of risk-disease associations used in the health analysis was supported by available criteria used to judge the certainty of evidence, such as the Bradford-Hill criteria used by the Nutrition and Chronic Diseases Expert Group (NutriCoDE),[193] the World-Cancer-Research-Fund criteria used by the Global Burden of Disease project,[194] as well as NutriGrade (**Table 3.4**).[195] The certainty of evidence supporting the associations of dietary risks and disease outcomes as used here were graded as moderate or high with NutriGrade,[179,180,181] and/or assessed as probable or convincing by the Nutrition and Chronic Diseases Expert Group,[193] and by the World Cancer Research.[196] The certainty of evidence grading in each case relates to the general relationship between a risk factor and a health outcome, and not to a specific relative-risk value.

We did not include all available risk-disease associations that were graded as having a moderate certainty of evidence and showed statistically significant results in the meta-analyses that included NutriGrade assessments.[179,180,181] That was because for some associations, such as for milk and fish, more detailed meta-analyses (with more sensitivity analyses) were available that indicated potential confounding with other major dietary risks or health status at baseline.[197,198,199] Such sensitivity analyses were not presented in the meta-analyses that included NutriGrade assessments, but they are important for health assessments that evaluate changes in multiple risk factors.

Table 3.3 Relative risk parameters (mean and low and high values of 95% confidence intervals) for dietary risks and weight-related risks.

Food group	Endpoint	Unit	RR mean	RR low	RR high	Reference
Red meat	CHD	100 g/d	1.15	1.08	1.23	Bechthold et al (2019)
	Stroke	100 g/d	1.12	1.06	1.17	Bechthold et al (2019)
	Colorectal cancer	100 g/d	1.12	1.06	1.19	Schwingshackl et al (2018)
	Type 2 diabetes	100 g/d	1.17	1.08	1.26	Schwingshackl et al (2017)
Fruits	CHD	100 g/d	0.95	0.92	0.99	Aune et al (2017)
	Stroke	100 g/d	0.77	0.70	0.84	Aune et al (2017)
	Cancer	100 g/d	0.94	0.91	0.97	Aune et al (2017)
Vegetables	CHD	100 g/d	0.84	0.80	0.88	Aune et al (2017)
	Cancer	100 g/d	0.93	0.91	0.95	Aune et al (2017)
Legumes	CHD	57 g/d	0.86	0.78	0.94	Afshin et al (2014)
Nuts	CHD	28 g/d	0.71	0.63	0.80	Aune et al (2016)
Underweight	CHD	15<BMI<18.5	1.17	1.09	1.24	Global BMI Collab (2016)
	Stroke	15<BMI<18.5	1.37	1.23	1.53	Global BMI Collab (2016)
	Cancer	15<BMI<18.5	1.10	1.05	1.16	Global BMI Collab (2016)
	Respiratory disease	15<BMI<18.5	2.73	2.31	3.23	Global BMI Collab (2016)
Overweight	CHD	25<BMI<30	1.34	1.32	1.35	Global BMI Collab (2016)
	Stroke	25<BMI<30	1.11	1.09	1.14	Global BMI Collab (2016)
	Cancer	25<BMI<30	1.10	1.09	1.12	Global BMI Collab (2016)
	Respiratory disease	25<BMI<30	0.90	0.87	0.94	Global BMI Collab (2016)
	Type 2 diabetes	25<BMI<30	1.88	1.56	2.11	Prosp Studies Collab (2009)
Obesity (grade 1)	CHD	30<BMI<35	2.02	1.91	2.13	Global BMI Collab (2016)
	Stroke	30<BMI<35	1.46	1.39	1.54	Global BMI Collab (2016)
	Cancer	30<BMI<35	1.31	1.28	1.34	Global BMI Collab (2016)
	Respiratory disease	30<BMI<35	1.16	1.08	1.24	Global BMI Collab (2016)
	Type 2 diabetes	30<BMI<35	3.53	2.43	4.45	Prosp Studies Collab (2009)
Obesity (grade 2)	CHD	30<BMI<35	2.81	2.63	3.01	Global BMI Collab (2016)
	Stroke	30<BMI<35	2.11	1.93	2.30	Global BMI Collab (2016)
	Cancer	30<BMI<35	1.57	1.50	1.63	Global BMI Collab (2016)
	Respiratory disease	30<BMI<35	1.79	1.60	1.99	Global BMI Collab (2016)
	Type 2 diabetes	30<BMI<35	6.64	3.80	9.39	Prosp Studies Collab (2009)
Obesity (grade 3)	CHD	30<BMI<35	3.81	3.47	4.17	Global BMI Collab (2016)
	Stroke	30<BMI<35	2.33	2.05	2.65	Global BMI Collab (2016)
	Cancer	30<BMI<35	1.96	1.83	2.09	Global BMI Collab (2016)
	Respiratory disease	30<BMI<35	2.85	2.43	3.34	Global BMI Collab (2016)
	Type 2 diabetes	30<BMI<35	12.49	5.92	19.82	Prosp Studies Collab (2009)

Table 3.4 Overview of existing ratings on the certainty of evidence for a statistically significant association between a risk factor and a disease endpoint. The ratings include those of the Nutrition and Chronic Diseases Expert Group (NutriCoDE),[193] the World Cancer Research Fund,[196] and NutriGrade.[179,180,181] The ratings relate to the risk-disease associations in general, and not to the specific relative-risk factor used for those associations in this analysis.

Food group	Endpoint	Association	Certainty of evidence
Fruits	CHD	reduction	NutriCoDE: probable or convincing;<br>NutriGrade: moderate quality of meta-evidence
	Stroke	reduction	NutriCoDE: probable or convincing<br>NutriGrade: moderate quality of meta-evidence
	Cancer	reduction	WCRF: strong evidence (probable) for some cancers<br>NutriGrade: moderate quality of meta-evidence for colorectal cancer
Vegetables	CHD	reduction	NutriCoDE: probable or convincing<br>NutriGrade: moderate quality of meta-evidence
	Cancer	reduction	WCRF: strong evidence (probable) for non-starchy vegetables and some cancers<br>NutriGrade: moderate quality of meta-evidence for colorectal cancer
Legumes	CHD	reduction	NutriCoDE: probable or convincing<br>NutriGrade: moderate quality of meta-evidence
Nuts and seeds	CHD	reduction	NutriCoDE: probable or convincing<br>NutriGrade: moderate quality of meta-evidence
Red meat	CHD	increase	NutriGrade: moderate quality of meta-evidence
	Stroke	increase	NutriGrade: moderate quality of meta-evidence
	Cancer	increase	WCRF: strong evidence (probable) for colorectal cancer<br>NutriGrade: moderate quality of meta-evidence for colorectal cancer
	Type-2 diabetes	increase	NutriCoDE: probable or convincing<br>NutriGrade: high quality of meta-evidence

NutriCoDE: Nutrition and Chronic Diseases Expert Group
NutriGrade: Grading of Recommendations Assessment, Dvelopment, and Evaluation (GRADE) tailored to nutrition research
WCRF: World Cancer Research Fund

For the different diet scenarios, we calculated uncertainty intervals associated with changes in mortality based on standard methods of error propagation and the confidence intervals of the relative risk parameters. For the error propagation, we approximated the error distribution of the relative risks by a normal distribution and used that side of deviations from the mean which was largest. This method leads to conservative and potentially larger uncertainty intervals as probabilistic methods, such as Monte Carlo sampling, but it has significant computational advantages, and is justified for the magnitude of errors dealt with here (<50%) (see e.g. IPCC Uncertainty Guidelines).

Inequality Context

The indicator reflects inequalities in deaths attributable to imbalanced diets between, but not within countries.

Caveats

In the comparative risk assessment, we used relative risk factors that are subject to the caveats common in nutritional epidemiology, including small effect sizes and potential measurement error of dietary exposure, such as over and underreporting and infrequent assessment.[200] For our calculations, we assumed that the risk-disease relationships describe causal associations, an assumption supported by the existence of statistically significant dose-response relationships in meta-analyses, the existence of plausible biological pathways, and supporting evidence from experiments, e.g. on intermediate risk factors.[176,178,179,180,181,193,201,202,203,204] However, residual confounding with unaccounted risk factors cannot be ruled out in epidemiological studies. Additional aspects rarely considered in meta-analyses are the importance of substitution between food groups that are associated with risks, and the time lag between dietary exposure and disease.

To address potential confounding, we omitted risk-disease associations that became non-significant in fully adjusted models, in particular those related to milk intake,[197,198] and to fish intake.[199,205,206,207,208] The quality of evidence in meta-analyses that covered the same risk-disease associations as used here was graded with NutriGrade as moderate or high for all risk-disease pairs included in the analysis (**table 3.4**).[179,180,181] In addition, the Nutrition and Chronic Diseases Expert Group and the World Cancer Research Fund graded the evidence for a causal association of ten of the 12 risk-disease associations included in the analysis as probable or convincing.[193,196] The relative health ranking of leading risk factors found in our analysis was similar to existing rankings that relied on different relative-risk parameters and exposure data.[194,209]

As exposure data, we used a proxy of food consumption that was derived from estimates of food availability that were adjusted for the amount of food wasted at the point of consumption.[171,174] An alternative would have been to rely on a set of consumption estimates that has been based on a variety of data sources, including dietary surveys, household budget and expenditure surveys, and food availability data.[184,185] However, neither the exact combination of these data sources, nor the estimation model used to derive the data have been made publicly available. For some individual countries, using dietary surveys would also have been an alternative. However, underreporting is a persistent problem in dietary survey,[186,187] and regional differences in survey methods would have meant that our results would not be comparable between countries. In contrast to dietary surveys, waste-adjusted food-availability estimates indicate levels of energy intake per region that reflect differences in the prevalence of overweight and obesity across regions.[175]

Future form of the indicator

The estimates of diet-related disease burden will be updated annually based on new data on food intake, mortality, and population numbers.

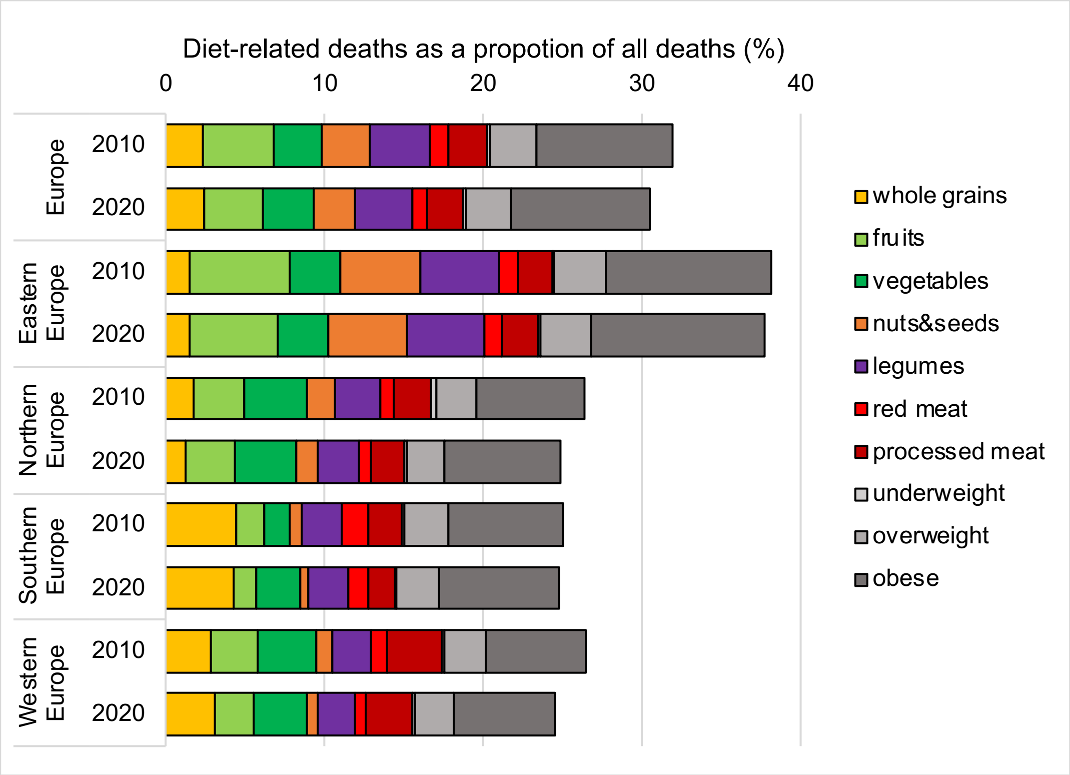

Figure 3.8 Burden of diet-related deaths in Europe by region, year, and risk factor.

3.5: Health sector emissions and harms

Geographic Coverage of Europe

For this indicator, nations in the WHO-53 region are included.

Data, methods, caveats, future form of the indicator

This indicator is based on the data and methods of **indicator 3.6** of the global *Lancet* Countdown 2022 Report.

Romanello, M., Di Napoli, C., Drummond, P., Green, C., Kennard, H., Lampard, P., ... & Costello, A. (2022). The 2022 report of the Lancet Countdown on health and climate change: health at the mercy of fossil fuels. *The Lancet*, *400*(10363), 1619-1654.

A full description of the methods, data, caveats and future form of the indicator can be found in the Appendix of the global *Lancet* Countdown 2022 report[16]: https://www.thelancet.com/cms/10.1016/S0140-6736(22)01540-9/attachment/d63703f8-315e-4cdb-9573-1e552e1d4913/mmc5.pdf

Section 4: Economics and finance

4.1: The health linked economic impacts of climate change and its mitigation

Indicator 4.1.1: Economic losses due to climate-related extreme events

Geographic Coverage of Europe

For this indicator, nations in the WHO-53 region plus Liechtenstein and Kosovo (under UNSC resolution 1244) are included. Note however, data is only reported for nations where these exceed reporting thresholds. Country data is also summed according to subregional groupings taken from the UN Geoscheme for Europe and Asia, as shown below.

Data

1. Swiss Re Institute sigma catastrophe database
2. IMF World Economic Outlook (October 2022)

This indicator is based on the data and methods of indicator 4.1.1 of the global *Lancet* Countdown 2022 Report,[16] and the method is reproduced and adapted specifically for the Europe Region as outlined below. The Swiss Re Institute provided the data for this indicator. The Swiss Re Institute sigma catastrophe database is an international commercial database recording both natural and man-made disasters from 1970 and has over 12,000 entries.

The term 'natural catastrophe' refers to an event caused by natural forces. Such an event generally results in a large number of individual losses involving many insurance policies. The scale of the losses resulting from a catastrophe depends not only on the severity of the natural forces concerned, but also on man-made factors, such as building design or the efficiency of disaster control in the afflicted region.
Natural catastrophes are categorised as shown in **Table 4.1**. For this indicator, only data for 'weather-related' events is presented.

Table 4.1 Categorisation of natural catastrophes in the data provided by the Swiss Re Institute.

Category	Peril Group	Peril
	Earthquake	Earthquake
		Tsunami
		Volcano eruption
	Weather-related	Storm
		Flood
		Hail
		Cold, frost
		Drought, bush fires, heat waves
		Other natural catastrophes

Total (insured and uninsured) economic losses reported by Swiss Re are all the financial losses directly attributable to a major event, i.e., damage to buildings, infrastructure, vehicles etc. This also includes losses due to business interruption as a direct consequence of the property damage. Insured losses are gross of any reinsurance, be it provided by commercial or government schemes. Total loss figures do not include indirect financial losses – i.e., loss of earnings by suppliers due to disabled businesses, estimated shortfalls in GDP and non-economic losses, such as loss of reputation or impaired quality of life. Insured losses refer to all insured losses except liability. To calculate uninsured losses, insured losses are subtracted from total losses.

Data are collected from a variety of sources, both internal and external. These include professional insured claims aggregators as well as insurance associations. Among the sources are also official government data, when available. Economic loss data can be estimated on the basis of Swiss Re proprietary catastrophe risk models. Also, if insured loss data are available, economic loss data are estimated on the basis of the local insurance penetration and other event-specific information (such as damages to public infrastructure, number of buildings damaged or destroyed etc.).

Minimum thresholds apply to inclusion in the database. At least one of the following must apply, for events recorded in 2022 (with economic values changing each year following changes to US CPI, and with values converted from US$ to € using average market exchange rates for 2022 from the IMF:

- **Insured losses (claims):** €23.9 million (maritime disasters), €47.9 million (aviation), €59.4 million (other)
- **Economic losses**: €114.5 million
- **Casualties**: Dead or missing: 20; Injured: 50; Homeless: 2000

Loss values are presented in US$ by Swiss Re, or if initially expressed in local currency, converted to US$ using year-end exchange rates, and converted to €. Further information on the methodology of the sigma explorer database can be found here: https://www.sigma-explorer.com/documentation/Methodology_sigma-explorer.com.pdf. Total insured and uninsured losses are then divided by total GDP for each year. GDP data are taken from the IMF's World Economic Outlook (October 2022 Edition). All values reported for this indicator are in €2022.

Currencies in this indicator are converted from dollars to euros using the International Monetary Fund (IMF) exchange rate of 0.9496 euros to the dollar (2022) (https://data.imf.org/?sk=4c514d48-b6ba-49ed-8ab9-52b0c1a0179b).

Inequality Context

Due to high year-on-year variability, it is not possible to draw meaningful quantitative insights on inequalities between countries in the Europe region, though a few general statements can be made. Forty of the 53 countries in the WHO Europe region are classified as having Very High development according to the UN's Human Development Index (HDI), and 34 belong to the High Income Group classified by the World Bank. These

groupings often report high weather-related economic losses, but this can reflect the high value of the properties affected. The Europe region has 10 countries classified in the High HDI band and 2 in the Medium, and 15 countries in the Upper Middle income group and four in the Lower Middle income group. These groups can have lower economic losses, but this can reflect lower property values and do not always reflect the level of hardship and disruption caused or the resiliency challenges faced. Their smaller numbers can also mean their challenges can be overlooked. Some natural catastrophes are also more likely to occur in some regions than others, and these can coincide with regions more affected by inequalities; for example, heatwaves can be more common in Southern Europe than in Northern Europe, and some regions are more prone to floods and storms than others. One trend that does appear to be genuine is that, although the amount of losses that are insured appear to be on an upward trend across all groups, more losses are typically insured in wealthier and more developed regions than in less wealthy and less developed regions.

Caveats

Only events with measurable economic losses above the threshold levels are included. Each natural catastrophe event recorded is assigned a direct economic loss, and where applicable, an insured loss. Where available, data is taken from official institutions, but where not, estimates are calculated. The process for estimation depends on what data is available. For example, if loss estimates from insurance market data is available, this data may be combined with data on insurance penetration and other event-specific information to estimate total economic losses. If only low-quality information is available, such as a description of the number of homes damaged or destroyed, assumptions on value and costs are made. Some data (including both losses and GDP values) may be revised compared to previous reports, due to updated information or detailed measurement approaches.

Future Form of the Indicator

No changes to the indicator are currently planned.

Findings and additional analysis

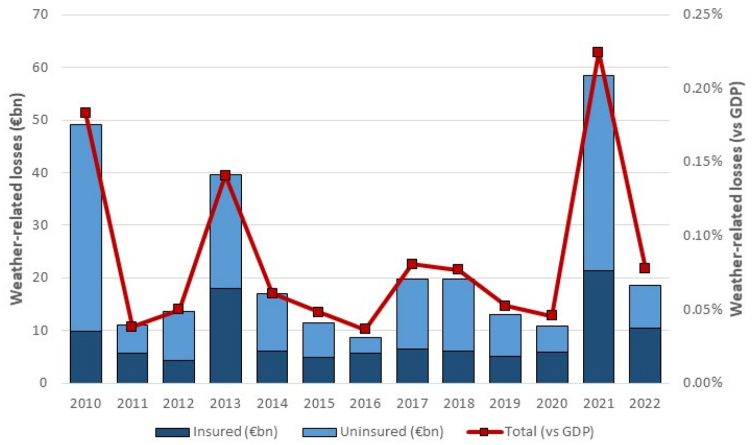

Figure 4.1 Insured and Uninsured weather-related losses in €bn and vs GDP for the WHO Europe region.

Indicator 4.1.2: Change in labour supply

Geographic Coverage of Europe

For this indicator, we included all European Union member countries.

Data

1. Labour data: Eurostat Regional Database, 1995-2020. The original labour data comes from EU Labour Force Survey (EU-LFS). This is a quarterly household sample survey conducted in all Member States of the EU, the United Kingdom of Great Britain and Northern Ireland, EFTA, and candidate countries.
2. Climate data: ERA5-Land 0.1°×0.1 spatial and hourly temporal resolution.[10]

Methods

To track the impact of climate change on labour supply in Europe, we use a panel data fixed-effects regression. We control for mean temperature and its second-degree polynomial and total precipitation and its second-degree polynomial as climatic stressors (following Dasgupta *et al.*, 2021). Our dependent variable is the log of the number of working hours at the NUTS-2 level. Our specification also controls for both NUTS-2 year fixed-effects to account for unobserved heterogeneity such as changes in labour policies. The standard errors are clustered at the country-level. Our panel data specification can be written as follows:

$$\ln(y_{it}) = f_s(T_{it}) + \boldsymbol{X}\beta_{(it)} + \alpha_{(i)} + \gamma_{(t)} + \mu_{(it)}$$

where y_{it} is the log of number of hours worked at NUTS-2 region i at year t. $f_s(T_{it})$ represents the non-linear effect of sub-national temperature on labour supply, $\boldsymbol{X}\beta_{it}$ is a vector of precipitation terms and log of working population (between 15-64). $\alpha_{(i)}$ and $\gamma_{(t)}$ are NUTS-2 and year fixed-effects to control for unobserved heterogeneity, while μ_{it} is a random error term. In the second step, we conduct a counterfactual analysis of the change in labour supply due to change in temperature from a long-term mean in Europe. We combine our econometric estimates with differences in periodic warming from a long-term mean of 1981-2010 to estimate the impact of temperature change on the number of hours worked in each NUTS-2 region. These data are presented as a percentage change in labour supply compared to 1965-1994.

Caveats

The main caveat in this indicator is that the labour supply data is available only at the annual level, as such within year heterogeneity cannot be accounted for.

Future Form of the Indicator

Future iterations of this indicator will use micro-survey data from the EU Labour Force Survey (EU-LFS) and European Union Statistics on Income and Living Conditions (EU-SILC) to account for temporal heterogeneity and various household and societal characteristics. Second, impacts on labour supply and labour productivity will be estimated separately. Third, impacts of adaptation will be explicitly accounted for.

Indicator 4.1.3: Impact of heat on economic activity

Geographic Coverage of Europe

1. South European countries: Albania, Austria, Belgium, Bulgaria, Croatia, Cyprus, Czechia, Germany, Greece, France, Hungary, Italy, Luxembourg, Malta, Netherlands (Kingdom of the), North Macedonia, Poland, Romania, Slovakia, Slovenia, Spain, and Türkiye.
2. North European countries: Estonia, Latvia, Lithuania, Denmark, Finland, Iceland, Norway, and Sweden

Data

1. GDP growth data: Eurostat Regional Database, 2001-2020.
2. Climate data: ERA5-Land 0.1°×0.1 spatial and hourly temporal resolution.[10]

Methods

To track the impact of climate change on economic activity in Europe, we use a panel data regression with coefficients that vary over time. Our measure of climate change is temperature anomaly, defined as the annual temperature difference, in °C, from a mean temperature of a 30-year period between 1981-2010. Our dependent variable is the real GDP per capita growth at the NUTS-2 level. Our specification also controls for drought measured by twelve-month Standardized Precipitation Index (SPI), and precipitation and its second-degree polynomial. To account for unobserved heterogeneity, we include both NUTS-2 year fixed-effects. The standard errors are clustered at the country-by-year level. Our panel data specification can be written as follows:

$$y_{it} = \beta_1(\tau_t) + V_{(it)} + \gamma'(\tau_t)X_{(it)} + \alpha_{(i)} + \mu_{(it)}$$

where y_{it} is the real GPD per capita growth at NUTS-2 region i at year t, $\boldsymbol{V}_{it}$ is the temperature anomaly, and $\boldsymbol{X}_{it}$ is a vector of relevant variables affecting economic activity (droughts), while $\boldsymbol{\mu}_{it}$ is a random error term. All variables are recorded for different locations with index $i = 1, \ldots, N$ and over a number of years $t = 1, \ldots, T$. Our specification also includes location (NUTS-2) and time (year) fixed-effects to control for unobserved heterogeneity and factors influencing GDP growth such as technological or policy changes from year-to-year and natural resources endowments. The time-varying coefficients allow us to examine whether the relationship between temperature anomaly economic activity has evolved over time.

Caveats

The main caveat in this indicator is that the GDP growth data is available only at the annual-level, as such temporal heterogeneity cannot be accounted for.

Future Form of the Indicator

Future iterations of this indicator will use micro-survey data from the European Union Statistics on Income and Living Conditions (EU-SILC) to account for temporal heterogeneity and various household and societal characteristics.

Findings & additional analysis

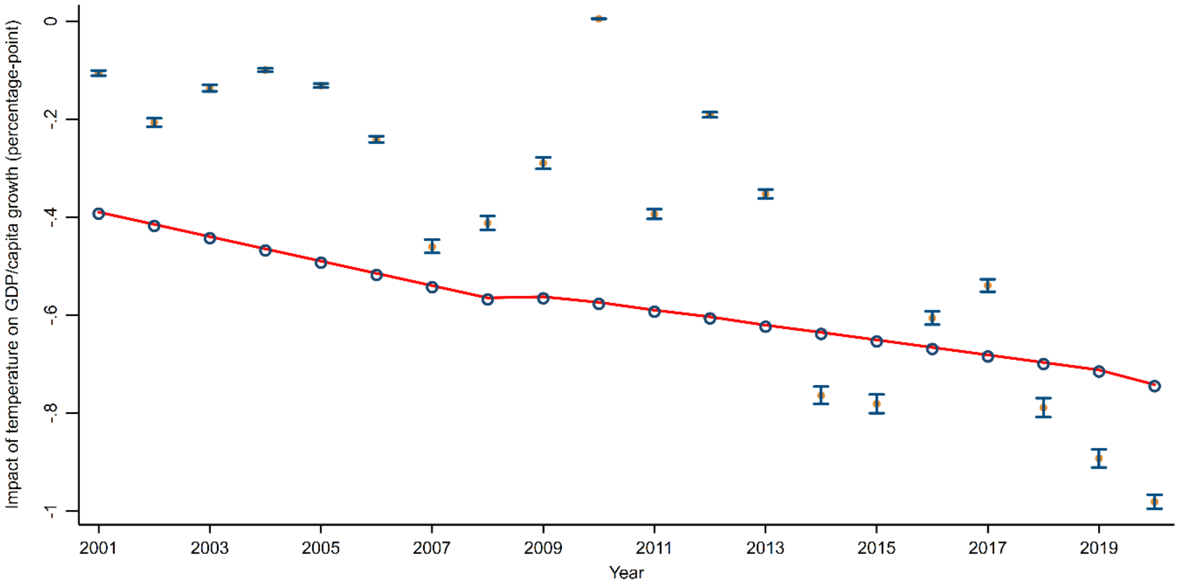

Figure 4.2 Reduction in real GDP per capita growth due to actual annual temperature anomaly from the reference period of 1981-2010 in Southern Europe. The yellow circles represent the change in GDP per capita for each year and the blue bars show the 95% confidence interval. The red line connects the regression coefficients for each year due to 1°C temperature anomaly. The estimates are generated from a time-varying coefficient regression using GDP per capita growth and ERA-5 Land temperature data at the NUTS-2 level. The specification also includes NUTS-2 and year fixed-effects. The standard errors are clustered at the country-by-year level.

Indicator 4.1.4: Monetised value of unhealthy diets

Geographic Coverage of Europe

For this indicator, we used the M49 classification of Europe of the United Nations Statistical Division.

Data

1. Values of statistical life: OECD[210]
2. Diet-related health impacts: estimates based on indicator 3.4.2

Methods

Valuing the cost of unhealthy diets is relevant for tracking the health co-benefits of dietary changes towards more sustainable diets. According to model-based analyses, dietary changes towards more sustainable diets are associated with health benefits,[211] have a significant climate-change mitigation potential,[212] and they are needed as a part of a combination of mitigation measure to stay on track to limit global warming to less than 2 degrees Celsius.[213]

This indicator uses the estimates of health co-benefits of dietary change from the previous working group as a starting point, and combines those with standard methods to value the health impacts of dietary changes.[214] The most common method for valuing health impacts in monetary terms, and the one used here, makes use of the so-called value of statistical life, a measure based on the aggregate willingness to pay for health risk reduction.
To estimate the value of health co-benefits, estimates of the diet-related mortality (from WP2) are combined with estimates of the value of statistical life. The value of statistical life (VSL) is a measure for the willingness to pay for a mortality risk reduction defined as the marginal rate of substitution between money and mortality risk in a defined time period.[210] The VSL does not represent the value of life itself, but rather the value of small risks to life which can be estimated either from market decisions that reveal the implicit values reflected in behaviour (revealed preference studies), or by using surveys which elicit respondents' willingness to pay for small reductions in mortality risks directly (stated preference studies).

The VSL values are based on a comprehensive global meta-analysis of stated preference surveys of mortality risk valuation undertaken for the Organisation for Economic Co-operation and Development (OECD).[215] Following OECD recommendations, we adopt a VSL base value for the EU-27 of USD 3.5 million (1.75-5.25 million) and use the benefit-transfer method to calculate VSLs in other regions.[210] In the benefit-transfer method, the VSL base value is adjusted by income (Y) subject to an elasticity of substitution (β):

$$VSL_r = VSL_{base}\left(\frac{Y_r}{Y_{base}}\right)^{\beta}$$

Following OECD recommendations, we use GDP per capita adjusted for purchasing power parity (PPP) as a proxy for income, and we adopted an elasticity of 0.8 for benefit transfers to high-income countries and an elasticity of 1.0 for benefit transfers to low and middle-income countries.[210] Baseline data on GDP per capita were sourced

from the World Bank Development Indicator database. In line with World Bank methodology, we defined the income classification of countries depending on their GDP per capita (adjusted for purchasing power parity).

Caveats

There are uncertainties related to both the VSL estimates and the estimates of diet-related mortality. The former relates e.g. to the benefit transfer method that adjusts an aggregate value for differences in income levels, whilst the latter related to epidemiological uncertainty related to the relative risk estimates, among others.

Future Form of the Indicator

The estimates of the value of diet-related disease burden will be updated annually based on new data estimates of the burden of diet-related diseases (see **indicator 3.4.2**) and new economic data on national income.

4.2: The economics of the transition to zero-carbon economies

Indicator 4.2.1: Net value of fossil fuel subsidies and carbon prices

Geographic Coverage of Europe

For this indicator, we included the 53 member states of the WHO European Region. However not all nations from this region had reported fossil fuel subsidy or carbon price revenue data; the 2022 report included data from 43 of these 53 countries as shown in the table below (Liechtenstein and Kosovo [under UNSC resolution 1244] were also not included).

Countries included in indicator	Armenia, Austria, Azerbaijan, Belarus, Belgium, Bulgaria, Croatia, Cyprus, Czechia, Denmark, Estonia, Finland, France, Georgia, Germany, Greece, Hungary, Iceland, Ireland, Israel, Italy, Kazakhstan, Latvia, Lithuania, Luxembourg, Malta, Netherlands (Kingdom of the), Norway, Poland, Portugal, Republic of Moldova, Romania, Russian Federation, Slovakia, Slovenia, Spain, Sweden, Switzerland, Türkiye, Turkmenistan, Ukraine, United Kingdom of Great Britain and Northern Ireland, Uzbekistan
Countries not included	Albania, Andorra, Bosnia and Herzegovina, Kosovo (under UNSC resolution 1244), Kyrgyzstan, Liechtenstein, Monaco, Montenegro, North Macedonia, San Marino, Serbia, Tajikistan

Methods

This indicator is based on the data and methods of indicator 4.2.4 of the global *Lancet* Countdown 2022 Report.

Romanello, M., Di Napoli, C., Drummond, P., Green, C., Kennard, H., Lampard, P., ... & Costello, A. (2022). The 2022 report of the Lancet Countdown on health and climate change: health at the mercy of fossil fuels. *The Lancet*, *400*(10363), 1619-1654.

A full description of the methods, data, caveats and future form of the indicator can be found in the Appendix of the global *Lancet* Countdown 2022 report[16]: https://www.thelancet.com/cms/10.1016/S0140-6736(22)01540-9/attachment/d63703f8-315e-4cdb-9573-1e552e1d4913/mmc5.pdf

Currencies in this indicator are converted from dollars to euros using the International Monetary Fund (IMF) exchange rate of 0.9496 euros to the dollar (2022) (https://data.imf.org/?sk=4c514d48-b6ba-49ed-8ab9-52b0c1a0179b).

Inequality Context

Fossil fuel subsidies are often implemented to reduce fuel costs and stimulate economies. These subsidies can be particularly needed in developing economies, which in many cases have not been able to afford to transition to clean energy, and hence end up supporting new or existing fossil fuel technologies. Implementing carbon pricing

schemes and/or reducing fossil fuel subsidies in such countries can therefore have adverse effects on their economies, unless also accompanied by clean energy investment to help them transition to low-emission technologies. On the other hand, reducing fossil fuel usage helps reduce climate change and poor air quality, which can also adversely affect poorer countries, thereby reducing the impacts that would otherwise result from climate change and poor air quality.

Findings & additional analysis

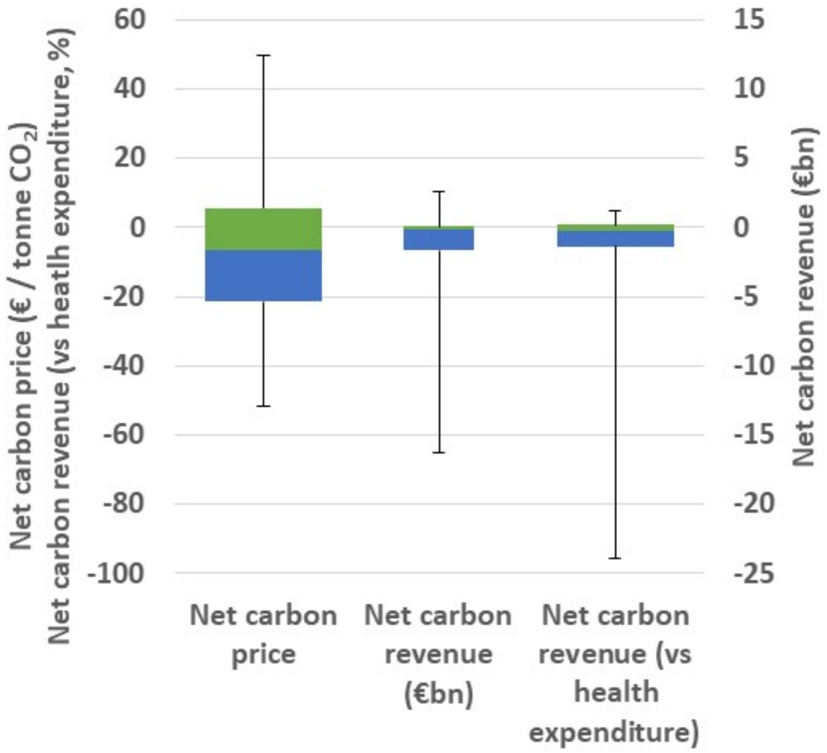

Figure 4.2 Net carbon price, net carbon revenue, and net carbon revenue as a fraction of national health expenditure for the WHO Europe region in 2020.

Indicator 4.2.2: Clean energy investment

Geographic Coverage of Europe

The data for this indicator is reported on a regional level only. The countries in the IEA report's Europe region include the EEA-38 countries plus Belarus, Gibraltar, Israel, Republic of Moldova, Ukraine and the United Kingdom of Great Britain and Northern Ireland but excluding Liechtenstein i.e a total of 43 countries.:

IEA Europe region	Albania, Austria, Belarus, Belgium, Bosnia and Herzegovina, Bulgaria, Croatia, Czechia, Denmark, Estonia, Finland, France, Germany, Gibraltar, Greece, Hungary, Iceland, Ireland, Israel, Italy, Kosovo (under UNSC resolution 1244), Latvia, Lithuania, Luxembourg, Malta, Montenegro, Netherlands (Kingdom of the), North Macedonia, Norway, Poland, Portugal, Republic of Cyprus, Republic of Moldova, Romania, Serbia, Slovakia, Slovenia, Spain, Sweden, Switzerland, Türkiye, Ukraine, United Kingdom of Great Britain and Northern Ireland

Data

IEA World Energy Investment 2023

Methods

This indicator is based on the data and methods of **indicator 4.1.1** of the global *Lancet* Countdown 2022 Report,[16] and the method is reproduced and adapted specifically for the Europe Region as outlined below.
The data for this indicator is sourced from the annual IEA World Energy Investment[216] publication. Key categories of investment are defined as follows:

Clean energy – investment in renewable power, energy efficiency, electricity networks, electric vehicles, battery storage, nuclear, low-emission fuels (modern liquid and gaseous bioenergy, low-emission hydrogen and low-emission hydrogen-based fuels), CCUS (carbon capture utilisation and storage), and other end-use (renewables for end use and electrification in the buildings, transport and industrial sectors).
Fossil fuels – investment in coal, oil and gas electricity generation capacity and fuel supply without CCS.
Power sector – investment in coal, oil, gas, nuclear and renewable electricity generation capacity, and electricity networks and battery storage. Renewables includes pumped-hydro storage.
Other supply – investment in coal, natural gas, oil and renewable energy supply for non-electricity purposes. This includes upstream mining, drilling, and pipeline infrastructure. Renewable energy includes modern liquid and gaseous bioenergy, low-carbon hydrogen, as well as hydrogen-based fuels that do not emit any CO_2 from fossil fuels directly when used and emit very little when being produced.
Energy efficiency – An energy efficiency investment is defined as the incremental spending on new energy-efficient equipment or the full cost of refurbishments that reduce energy use.

For most sectors, 'investment' is defined as ongoing capital spending on assets. For some sectors, such as power generation, this investment is spread out evenly from the year in which a new plant or upgrade of an existing one begins its construction to the year in which it becomes operational. For other sources, such as upstream oil and

gas and liquefied natural gas (LNG) projects, investment reflects the capital spending incurred over time as production from a new source ramp up or to maintain output from an existing asset. This definition and differs from the definition previously employed by the IEA before 2019, in which investment was defined as overnight capital expenditure.

Currencies in this indicator are converted from dollars to euros using the International Monetary Fund (IMF) exchange rate of 0.9496 euros to the dollar (2022) (https://data.imf.org/?sk=4c514d48-b6ba-49ed-8ab9-52b0c1a0179b).

Inequality Context

The data for this indicator is published only for the Europe region as a whole, so it is not possible to generate insights on inequalities between and within countries in Europe. However, it can be said that Europe needs to significantly increase its clean energy investment to reach net zero emission by 2050, and that Europe needs to play a leading role in delivering this transition at a global level. It is also important that, within Europe, this investment and its associated benefits including employment are fairly distributed, and perhaps even prioritised into more deprived regions, and which may otherwise resort to investing in dirtier forms of energy.

Caveats

Other areas of expenditure, including operation and maintenance, research and development, financing costs, mergers and acquisitions or public markets transactions, are not included. Investment estimates are derived from IEA data for energy demand, supply and trade, and estimates of unit capacity costs, For more information, see IEA World Energy Investment 2023.

Future Form of the Indicator

No changes to the indicator are currently planned.

Findings & additional analysis

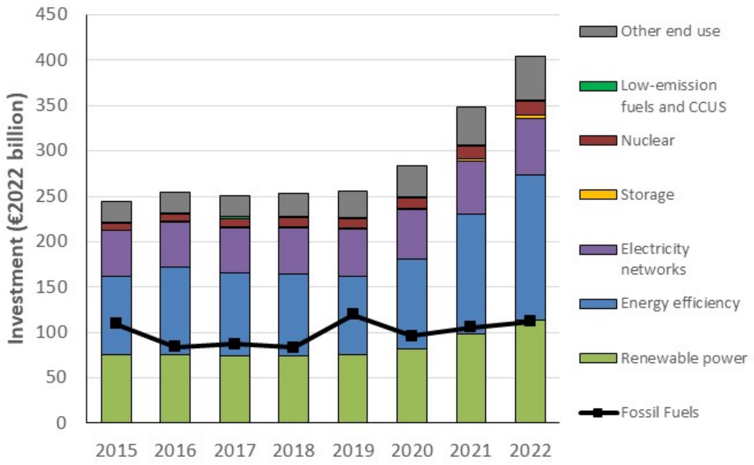

Figure 4.4 Investment in clean energy (columns) and fossil fuels (solid line) for the WHO Europe region.

Section 5: Politics and governance

5.1: Scientific engagement with health and climate change

Indicator 5.1.1: Coverage of health and climate Change in scientific articles

Geographic Coverage of Europe

The indicator covers all 53 WHO European region countries together with Liechtenstein and Kosovo (under UNSC resolution 1244).

Data

Publications are retrieved from the open access publication database OpenAlex using the query from Berrang-Ford *et al.* 2021.[217] This is a change from previous iterations, which used closed source databases. This change ensures the continued sustainability of the indicator, and increases replicability. Using Open Alex, and its predecessor Microsoft Academic Graph, has also been shown to increase coverage compared to traditional bibliographic database.

Methods

After retrieving records from Open Alex, by querying the latest snapshot, we applied the machine learning classifiers trained in Berrang-Ford *et al.* 2021.[217] These classifiers return a prediction for the relevance of a document, as well as for its membership of the categories impacts, mitigation, and adaptation. We also use a geoparser[218] to extract locations from the titles and abstracts of the texts.

Caveats

The database of studies is most likely incomplete. Some studies will be missed because they are not indexed in OpenAlex. Some studies will be missed because they do not match the query. And some studies will be missed because they are incorrectly predicted to be irrelevant. In addition, some of the included studies will have received incorrect predictions.

Analysis

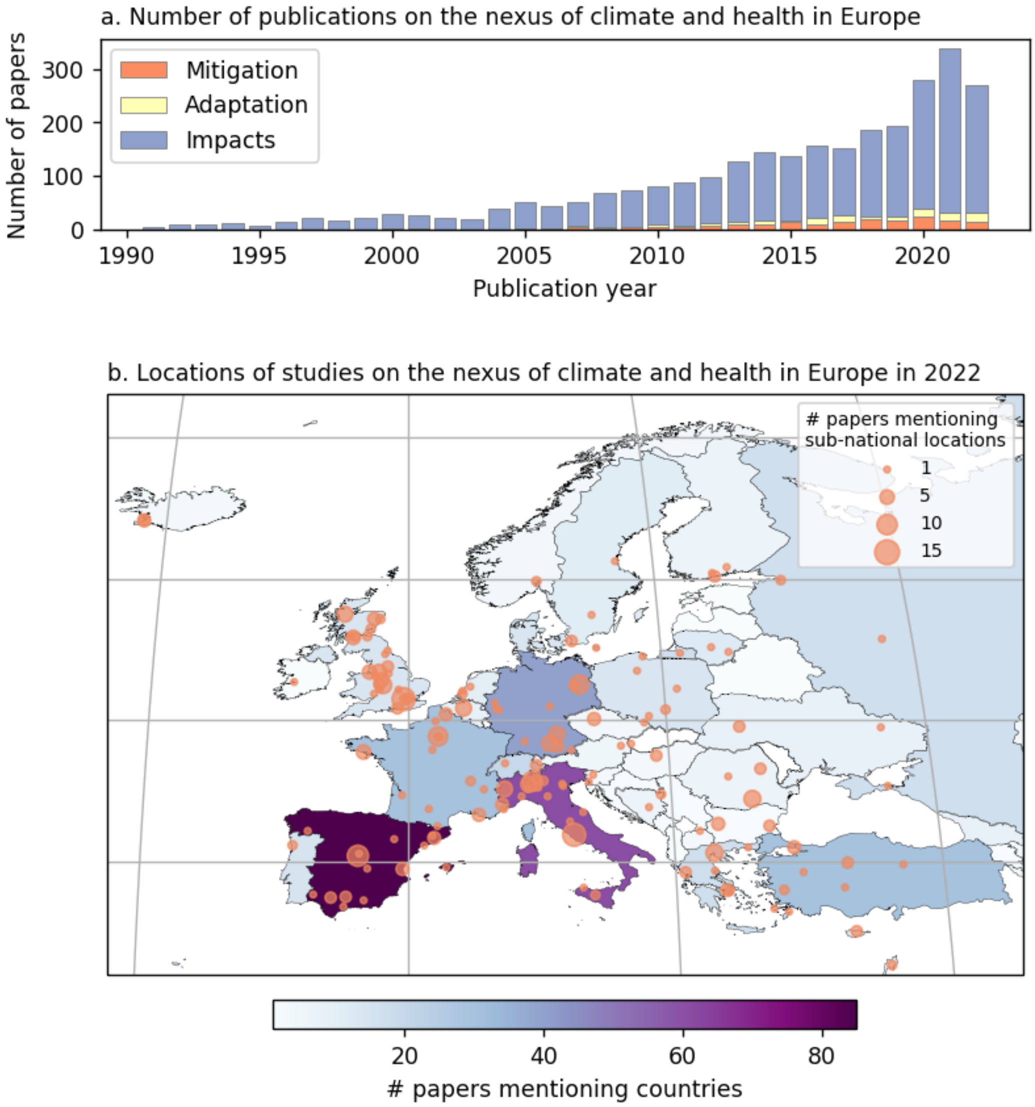

Figure 5.1 The size and distribution of papers on the nexus of climate and health that mention locations in Europe. (**A**) Number of papers published per year. (**B**) Locations mentioned in papers published in 2022. Countries with darker shading were mentioned in more papers. Dots refer to subnational locations and are scaled according to the number of locations mentioning them.

Indicator 5.1.2: Coverage of the health impacts of anthropogenic climate change in scientific articles

Geographic Coverage of Europe

The indicator covers all 53 WHO European region countries together with Liechtenstein and Kosovo (under UNSC resolution 1244).

Data

Publications come from the same source as **indicator 5.1.1**. Observational climate data come from HADCRUT4, for temperature and GPCC for precipitation. Climate model runs are from CMIP6.

Methods

The majority of studies on climate and health focus on the impacts of climate variables on health outcomes. Many studies attribute health outcomes to changes in climate variables without also attributing the changes in those variables to human influence on the climate. In this new sub-indicator, we apply a partial attribution, by extracting the locations from studies and analysing whether there we could detect trends in the climate variable investigated (limited to temperature and precipitation where long-term high-dimensional data is available) that could be attributable to human influence on the climate.

We use the same query as **indicator 5.1.1** to retrieve records. A subset of 2,000 records were labelled by hand, according to whether they presented evidence on the impacts of climate on health, as well as the type of evidence, the health outcome category, the climate driver, as well as any extreme events or climate driven increases in exposure. A machine learning model was trained to reproduce these labels, using nested cross-validation to select hyperparameters and evaluate the model. **Table 5.1** shows the performance of the different assessed models, each of which is a pre-trained language model, which we fine-tuned to our task of predicting the given category.

Category	bert-tiny	climatebert	distilroberta-base	scincl
Relevance	0.44 (0.29)	**0.85 (0.01)**	0.82 (0.02)	0.84 (0.02)
Climate driver	0.54 (0.00)	0.44 (0.23)	0.55 (0.01)	**0.61 (0.06)**
Health impact	0.21 (0.11)	0.59 (0.04)	0.58 (0.01)	**0.68 (0.06)**
Attribution type	0.13 (0.03)	0.58 (0.05)	0.50 (0.05)	**0.60 (0.02)**
Extreme event	0.01 (0.02)	**0.73 (0.07)**	0.71 (0.09)	0.66 (0.01)
Exposure	0.00 (0.00)	0.36 (0.11)	0.36 (0.11)	**0.51 (0.14)**

Table 5.1 F1 scores (mean and standard deviation across k-folds) for different models across the different prediction tasks. Where a task is multilabel, macro F1 scores are shown.

Once we had picked the best performing model, in this case scincl, we used the trained model to predict each category, for the hundreds of thousands of documents retrieved from our query which we had not labelled by hand. We also applied a geoparser, to extract the locations mentioned in each study.

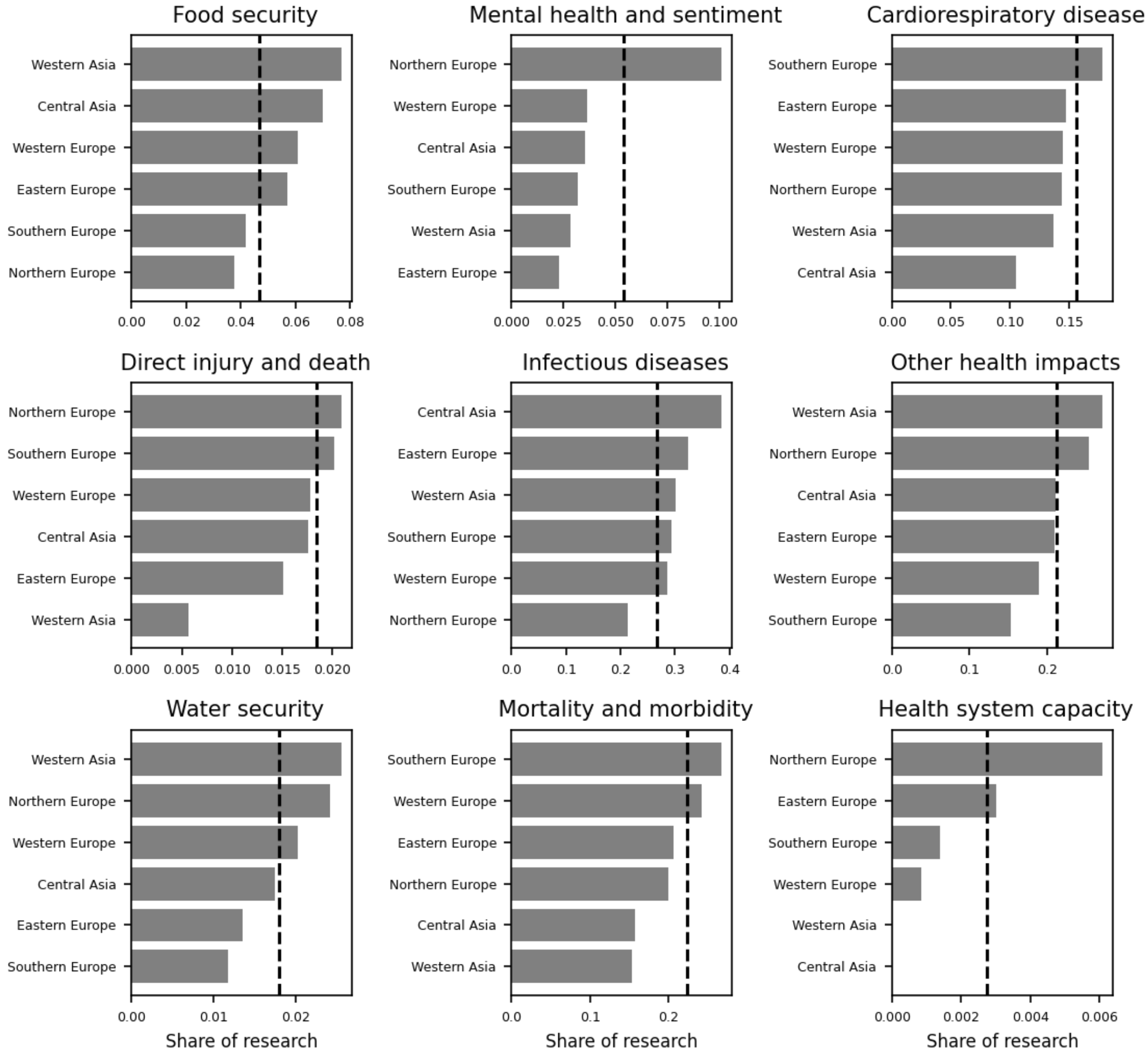

Figure 5.2 The share of research within each region on each health impact category. The share of all European research on each impact category is shown by the dashed line.

Following Callaghan *et al.* 2021,[219] we resolved each study to the set of 2.5 x 2.5 degree grid cells co-located with the locations it mentions, in order to combine the studies with evidence from the observational climate record and climate models, that tell us where trends in temperature or precipitation can be attributed to human influence on the climate. Using the same procedure as Knutson *et al.* 2013,[220] and Knutson & Zeng 2018,[221] we say that observed temperature or precipitation trends in a given grid cell are attributable to human influence on the climate where they are inconsistent with climate model runs modelling the climate system without anthropogenic forcing, and consistent with climate model runs modelling the climate system with anthropogenic forcing. All studies for which trends in the relevant climate variable are attributable to human influence for at least 50% of the area discussed are treated as partially attributable.

	Sub-category	bert-tiny	climatebert	distilroberta-base	scincl
**0**	Cardiorespiratory disease	0.27 (0.34)	**0.79 (0.044)**	0.78 (0.038)	0.74 (0.073)
**1**	Direct injury and death	0.0 (0.0)	0.36 (0.18)	0.34 (0.16)	**0.5 (0.15)**
**2**	Food security	0.11 (0.16)	0.73 (0.061)	0.65 (0.064)	**0.8 (0.12)**
**3**	Health system capacity	0.029 (0.021)	0.13 (0.19)	0.37 (0.26)	**0.51 (0.16)**
**4**	Infectious diseases	0.7 (0.15)	**0.94 (0.019)**	0.9 (0.015)	0.89 (0.036)
**5**	Mental health and sentiment	0.19 (0.12)	0.87 (0.031)	0.86 (0.018)	**0.95 (0.024)**
**6**	Mortality and morbidity	0.23 (0.33)	**0.78 (0.037)**	0.73 (0.05)	0.77 (0.053)
**7**	Other health impacts	0.34 (0.044)	**0.63 (0.029)**	0.42 (0.27)	0.55 (0.11)
**8**	Water security	0.038 (0.031)	0.095 (0.13)	0.2 (0.15)	**0.4 (0.16)**

Table 5.2 F1 scores (mean and standard deviation across k-folds) for different models across the different subcategories of health impacts.

Caveats

As with **indicator 5.1.1**, we likely miss studies due to limitations of the database, our query, and the machine learning models we train to predict inclusion. Because our classifiers do not work perfectly, a minority of our dataset will be mislabelled. Although the average F1 score across health impacts is fairly low 0.68, it is significantly higher for more common health impact categories such as mental health and sentiment (0.95) and infectious diseases (0.89) (**Table 5.2**), meaning that we have lesser uncertainty while identifying the more commonly studied health impact categories.

5.2: Individual engagement with health and climate change on social media

Social media has become a wide-spread new source of information that allows scientists and experts to reach a broader audience and allows the audience to be more engaged in the discussions about climate change and express their opinions about climate change and its impact on individual health. Existing studies show that only based on the Twitter data there can be tracked certain correlations between information about climate change and the citizen sentiment,[222] also some recent analysis of the Twitter data shows that there exists an immediate response to the climate change regulations and discussions in public tweets.[223] That is another example of social media data relevance in understanding citizen perception of climate change consequences. One of the aspects working with the Twitter data is properly identifying geolocation of such data and mapping it to country-level specifics. Here we propose an instrument to identify geographical locations of the tweets and apply multi-lingual keyword list to estimate the intensity of the rhetoric related to climate change and health relations. The findings from this study can be used to inform public health campaigns and establish policy priorities.

Geographic Coverage of Europe

Table 5.3 List of cities and countries included in this indicator.

Country	City	Language
Austria	Vienna	German, English
Bulgaria	Sofia	Bulgarian, English
Croatia	Zagreb	Croatian, English
Czechia	Prague	Czech, English
Cyprus	Nicosia<br>Limassol	English
Estonia	Tallinn	Estonian, English
Finland	Helsinki	Finnish, English
France	Paris	French, English
Germany	Berlin<br>Hamburg	German, English
Hungary	Budapest	Hungarian, English
Iceland	Reykjavik	Icelandic, English
Ireland	Dublin	English
Italy	Rome	Italian, English
Latvia	Riga	Latvian, English
Lithuanian	Vilnius	Lithuanian, English
Norway	Oslo	Norwegian, English
Poland	Warsaw	Polish, English
Portugal	Lisbon	Portuguese, English
Romania	Bucharest	Romanian, English
Slovakia	Bratislava	English
Slovenia	Ljubljana	Slovenian, English
Spain	Madrid<br>Barcelona<br>Valencia	Spanish, English
Switzerland	Zurich	German, English
United Kingdom of Great Britain and Northern Ireland	London	English

Data

To access the public rhetoric, we work with a set of the largest cities in the countries-members of the European Environment Agency (EEA) and the United Kingdom of Great Britain and Northern Ireland. We extract data based on the city-level and the list of the cities tries to represent the rhetoric that comes from at least one biggest city in each country (the full list of analyzed countries, cities, and languages is presented in **Table 5.3**). We retrieve all tweets that come from each of these locations for the period of 2022. The results yielded 28 cities with 2 490 601 English language tweets and 23 cities with 6 156 957 tweets on the official languages of corresponding countries (excluding countries with the predominant rhetoric being in English language such as Cyprus, Ireland, the UK), spanning all together 24 countries (refer to the limitations section for additional information regarding key term translations and the absence of official translations for Albania, Bosnia and Herzegovina, Montenegro, North Macedonia, Serbia, Kosovo (under UNSC resolution 1244), as well as language complexities involving Danish, Dutch, Greek, and Swedish, resulting in missing intensity scores for Belgian, Denmark, Greece, Netherlands (Kingdom of the), and Sweden; and additionally about special case of Türkiye).

Methods

First, we identify the geolocation instrument, and to do so we use a list of cities for which we query the Twitter data. The reverse geolocation tool embedded in the Twitter API system allows us to trace back the unique place IDs for as fine-grained locations as the cities. To retract such place IDs, we use the geographic coordinates unique for each city and run them through the API algorithm[1]. The returned data provides us with the place ids for city, neighbourhood, administration, and the country level. We obtain the list of place ids mapped to the cities and use them to query the twitter rhetoric.

With the dataset of multilingual geolocated tweets, we performed a search through the text of each tweet to identify if they discuss climate change and health-related topics. To do so, we first identified the list of keywords that we intended to search (both climate change and health related ones). We used the official glossary of the European Environment Agency (**Box 5.1**)[2] and excluded common words and words that could have multiple meanings unrelated to environment and health issues (**Box 5.2**). The list of excluded terms can be found at the end of the section and is presented in English, although respective translations in other languages have also been excluded.

We develop our indicator of health-climate change rhetoric engagement intensity as a monthly proportion of tweets containing at least one term from our health-related term list (see list of keywords at the end of the section) in relation to the number of tweets mentioning at least one of the key terms from the climate change term list that were coming from a given geolocated region (a city) in each language. To properly calculate the intensity, we first evaluate the number of tweets written in a specific language, and inside those we searched through the key terms about climate change and already inside the climate change specific tweets we search the health-related key terms.

---

[1] The reverse geocoding algorithm is described at the Twitter API portal: https://developer.twitter.com/en/docs/twitter-api/v1/geo/places-near-location/api-reference/get-geo-reverse_geocode

[2] European Environment Agency Glossary: https://www.eea.europa.eu/help/glossary#c4=10&c0=all&b_start=10

The language processing is quite important in our analysis and consists of two parts depending on whether language is part of Twitter supported languages or not:

- If the language is supported by the Twitter platform, it is automatically identified and returned as part of the tweets meta-data. This is valid for the following languages: Bulgarian, Czech, German, Greek, Danish, Dutch, English, French, Icelandic, Italian, Hungarian, Latvian, Lithuanian, Norwegian, Polish, Portuguese, Romanian, Swedish, Slovenian, Spanish. In this case, we first subset the tweets based on such language identifiers, and then calculate the total number of tweets written by a given media outlet in each language. And then use this total amount of tweets to search through the keywords in a corresponding language.
- If the language is not supported by Twitter (such as Croatian, Estonian), the language will automatically be flagged as unidentified. In this case, we use all the returned non-English tweets as a denominator, and search keywords translated to the predominant non-English language in this location.

We utilized an originally written computational algorithm to identify the lists of tweets containing each keyword from a given list for each city in a given language on a monthly basis throughout 2022. These lists were combined to create a master list of unique tweets, excluding any duplicates resulting from multiple keywords mentioned in the same tweet. The number of the unique tweets mentioning climate change keyword for English and non-English set of tweets serves as the denominator for our indicator. For the numerator part we used those selected climate change specific tweets and identify how many of them mention at least one key term from the health-related terms (list of which is provided at the end of the section).

The resulting indicator of *rhetoric intensity* was obtained by dividing the number of health-related tweets by the number of climate change-related tweets for each city and each language. This allows us to identify the health specific rhetoric described as part of the general climate change rhetoric expressed by the public audience.

Figure 5.3 presents the distribution of intensity across the year. On average, engagement in health rhetoric was observed in approximately 5-7% of all the public tweets that mention climate change keywords, and this is valid for both English and non-English tweets (with wider confidence intervals for the English language tweets). Notably, this percentage exhibited peaks in summer and winter months, which could be attributed to extreme warm temperatures and heightened discourse surrounding related issues. One note with respect to this plot is that the denominator varies drastically around cities and countries, which can potentially overestimate the average intensity for certain cities and as a result lead to a total overestimation of the intensity (this can explain wider confidence intervals for the English language tweets subset). That is why, we recommend emphasizing the cross-country variation.

Figures 5.4-5.5 demonstrates the monthly dynamic for each of the cities for English language tweets (Sofia were excluded due to insufficient number of observations for tracking the dynamic) and cities for the non-English language tweets (with Cyprus cities being lumped together due to the small number of the tweets present in each of the cities; Ljubljana and Sofia were excluded due to insufficient number of observations for tracking the dynamic), and additionally allows us to provide a cross-country comparison. Here we can see that for individual

cities such Bratislava, Cypriot cities, Reykjavik the confidence intervals are quite wide, which indicates that the total number of tweets in these places is generally small, and the intensity scores can be quite overestimated. However, for the rest of the cities the rhetoric intensity is quite stable over time and spins around average 5-7% for both English and non-English language tweets.

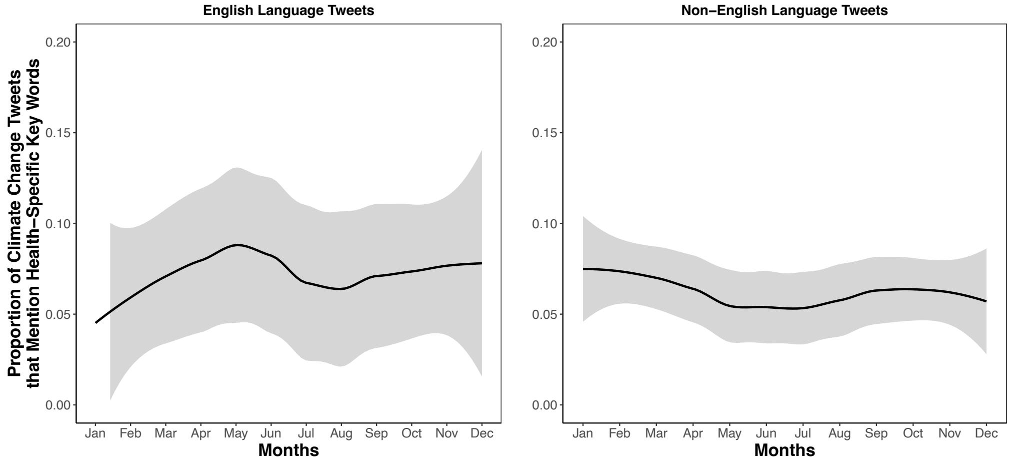

Figure 5.3 Average intensity score across all cities per month.

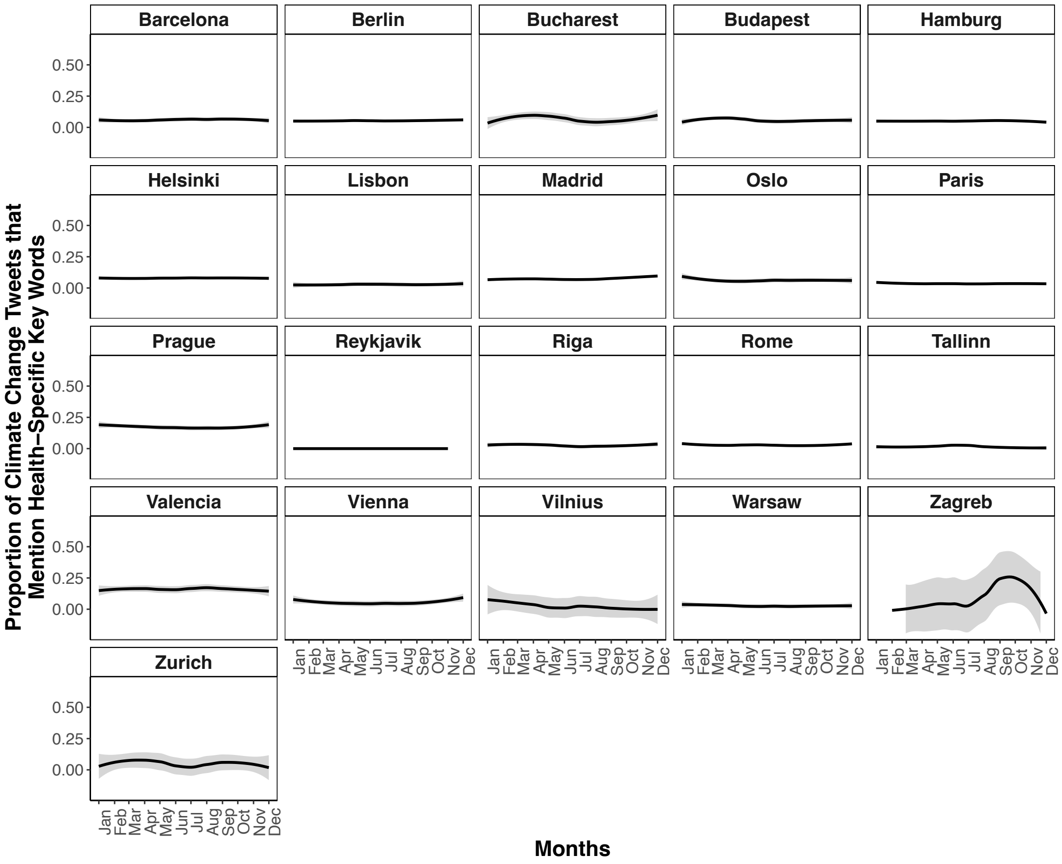

Figure 5.4 Monthly intensity scores for English language Tweets across cities.

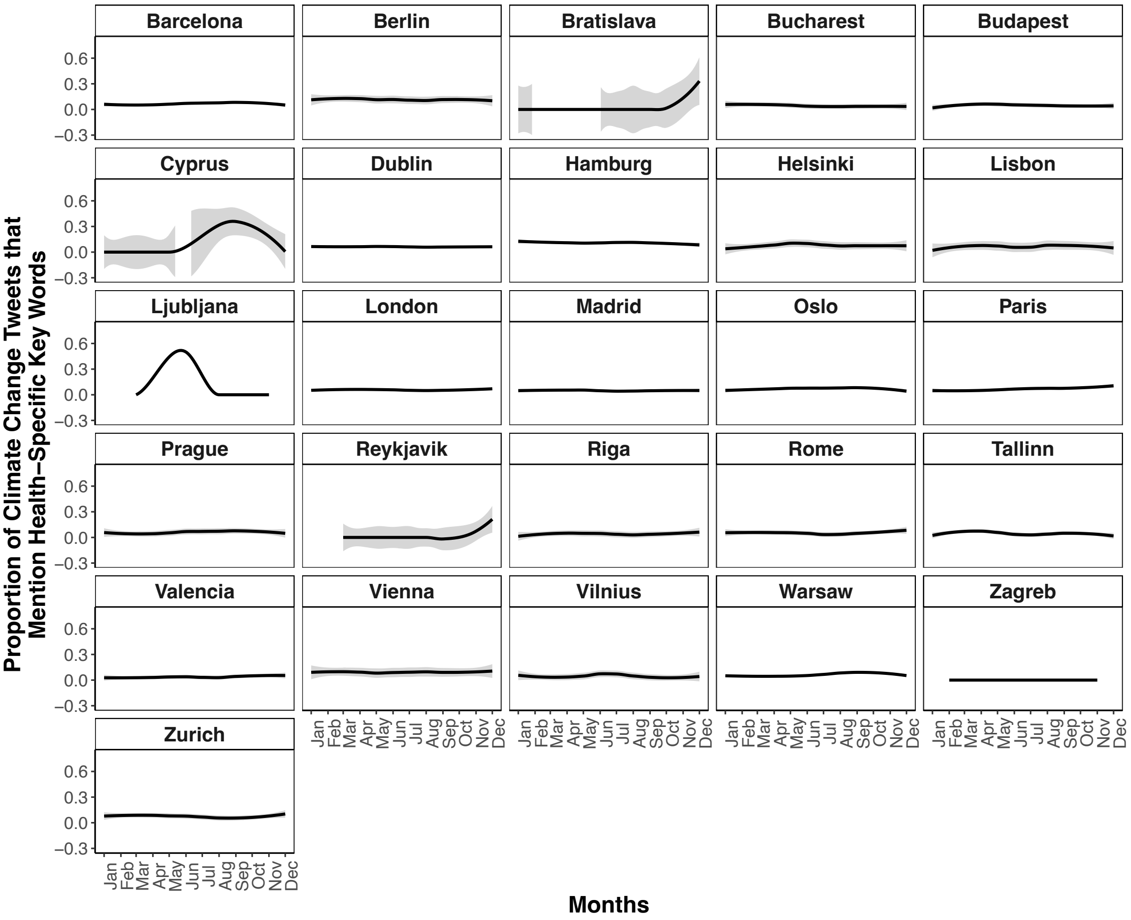

Figure 5.5 Monthly Intensity Scores for Non-English Language Tweets Across the Cities.

To explore more the cross-country comparison, we put together data at the country level, lumping together all the tweets for year 2022 and not including any potential time variation. That allows us to map the average intensity score for each country to the maps, which are presented in **Figures 5.6** and **5.7**. We emphasize the map on **Figure 5.6**, because it presents the comparison for non-English language tweets and can be perceived as more accurate in terms of how locals engage in climate change- health rhetoric. Here we see a split between Northern and Central and Southern Europe, with public in Northern Europe generally engaging slightly more with such a rhetoric. In **Figure 5.7**, we show the map for English language tweets intensity. Here it is worth noting that tweets in English that come from not English-speaking countries can indicate that these are the tweets from foreigners (for instance, tourists) or locals who aim to reach a wider audience by tweeting in English. Despite this potentially more heterogenous sample of users, we still observe a similar pattern for the English language tweets.

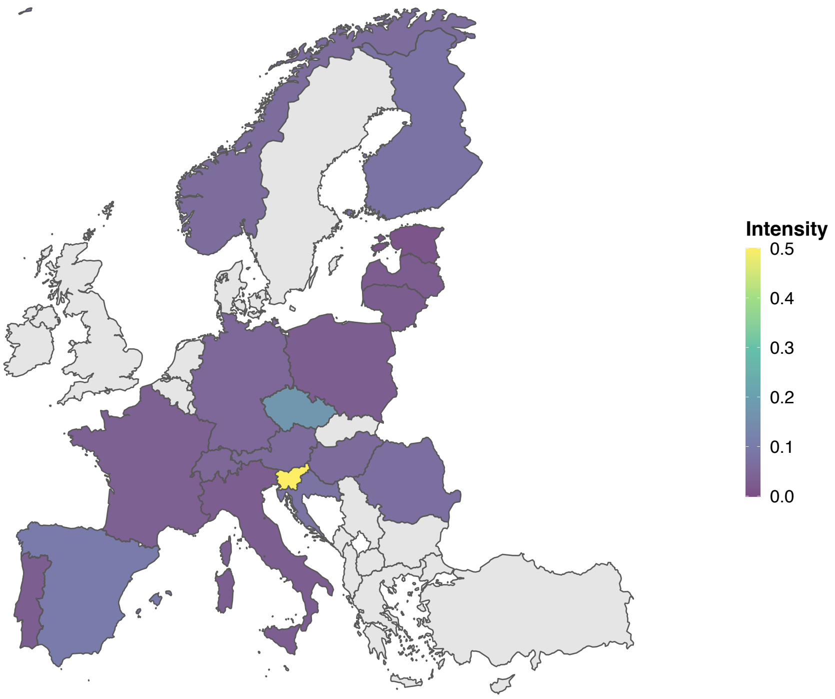

Figure 5.6 Map of the Cross-Country Comparison of Engagement Intensity with Non-English Language tweets.

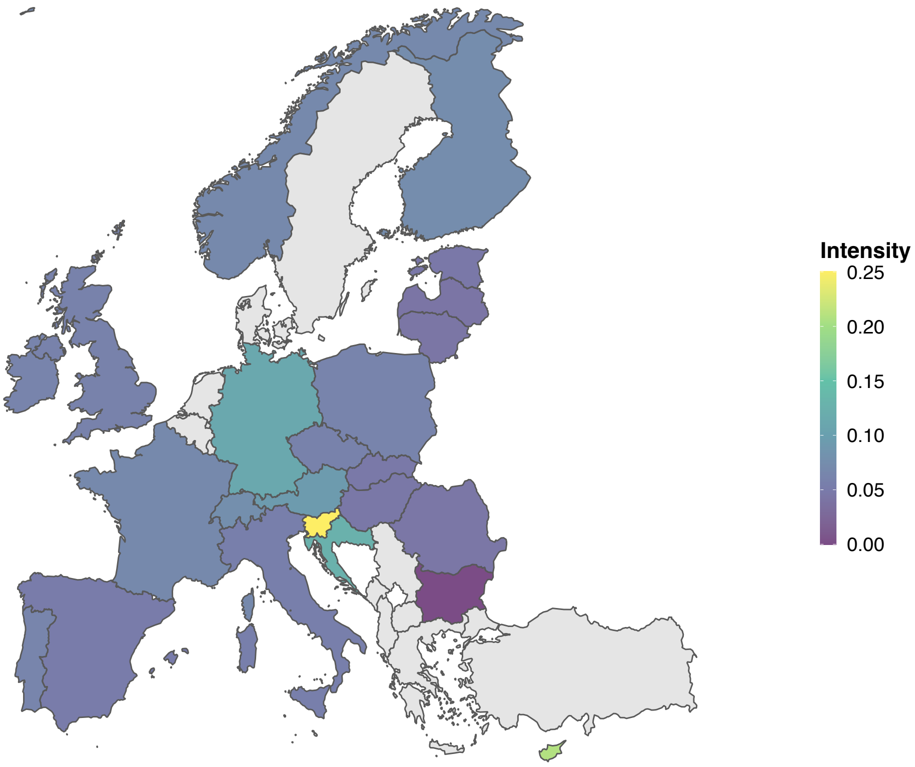

Figure 5.7 Map of the Cross-Country Comparison of Engagement Intensity with English Language tweets.

Caveats

Several limitations raised in the process of data collection and data analysis, that we aim to address in the next iterations of the report.

1. We utilize a non-random set of European cities, since Twitter data querying exceeds the permitted by Twitter API limits, we must gradually expand data collection. In this iteration of the report, we started with the largest megapolises in most of the EEA+UK countries. That presumably will allow us to catch the majority of the geolocated rhetoric that is coming from each state. In the further iterations of the report, we plan to expand the number of cities included.
2. We work only with the geolocated tweets, which presents only part of the Twitter rhetoric. On the one hand, such geographic restrictions limit and bias our data, but on the other hand, this is a precise way to map tweets to the country level and country-level specifics. We also assume that since we use the same methodology to pull the tweets for each of the locations, we can still proceed with the between-location comparisons.
3. The geolocation instrument is based not on the users' location but presumably on the location tracked through the users' devices. So even when a user self-identifies a location A, their tweets can be flagged in a location B. We suspect that such geolocation information is based on the IP addresses information and the users' geolocation settings embedded in their devices. Since Twitter does not disclose a precise procedure, there is an uncertainty about the geolocation information validity, which we partly lift with working with the multi-lingual key words.
4. There were also several pitfalls related to place IDs extraction. For some locations, such as Malta, the place ids for lower-level administrative units like cities and neighbourhoods is missing and place ids are only available for the whole country. That is precisely why Malta has been excluded from the current analysis. In such cases, we try to use the only available tier and use that place id to extract the tweets, unless that tier has corresponded to the country level. Some of the cities, such as Brussels, failed in the reverse geolocation exercise, which resulted in lacking the place id and inability to extract data for such locations.
5. One additional sample specific case is Türkiye, which has not been included in the current analysis, due to the lack of data for most of the year 2022. The lack of this data is due to the large number of geolocated tweets which was impossible to extract due to Twitter API imposed limits and restrictions. We aim to address this problem in the next iteration of the report.
6. To search through the climate change and health rhetoric, we utilize the official European Environment Agency (EEA) glossary, which is published in English and includes translations in the official languages of EEA member countries. However, it should be noted that Albania, Bosnia and Herzegovina, Kosovo (under UNSC resolution 1244), North Macedonia, Montenegro, and Serbia are not full members of the EEA, resulting in the absence of translations in the glossary for their official languages. Consequently, we exclude the rhetoric from these countries in the current iteration of our report. For future versions, we plan to address this issue by employing machine translation techniques to accurately translate all the glossary keywords and include the rhetoric from these countries in our analysis.
7. The utilized glossary includes the lemmatized version of all the terms. We exclusively utilize these lemmatized forms and did not generate all potential grammatical forms of the words for searching and

querying purposes. It should be noted that some languages, such as Spanish, French, and Bulgarian, are more sensitive to lemmatization, which can create imbalances in cross-country intensity estimates and potentially bias cross-country comparisons. However, this should not impact the within-country variation between media outlets, as the same glossary is applied to all tweets written by media outlets in each language within the country. One approach to address this issue is to employ stemming of words, which involves searching through word stems to capture a majority of grammatical forms. However, this approach may present challenges, such as the inclusion of small and potentially meaningless stems that may capture irrelevant rhetoric. For instance, using the stem "gen" to query for "genetic" may also capture words like "gender," which might be unrelated to climate change-health rhetoric. This could result in an overestimation of the intensity indicator. Therefore, we choose not to perform stemming and instead opt for potentially underestimating effects. In future iterations of this indicator, our plan is to expand the list of keywords by including all possible grammatical forms of the keywords in the search, thus providing a more comprehensive analysis.

8. Another language specific problem that we face is related to special characters that are used in Danish, Dutch, Greek, and Swedish languages. These special characters prevent the automated regular expressions querying. We aim to address this problem in the next iteration of the report.

Future Form of the Indicator

1. Expand the number of cities and tweets extracted;
2. Improve the geolocation instrument;
3. Missing keywords translations for the cooperating members of the EEA;
4. Resolve language specifics issues for Danish, Dutch, Greek, and Swedish languages;
5. Expand the time-series aspect of the data, by including media outlets rhetoric prior to 2022.

Findings & additional analysis

By extracting all rhetoric shared by the analyzed geolocated Twitter data for the year 2022, we obtained a dataset comprising 2,490,601 English language tweets and 6,156,957 non-English language multilingual tweets. Among these, 146,578 English-language tweets and 478 910 non-English language tweets contained at least one of the selected climate change specific keywords in any language, which on average accounts for approximately 5.9% and 7.8% correspondingly of the total English and non-English tweets. From the selected climate change specific tweets, we identify 10,037 English language and 30,944 non-English language health specific tweets (tweets that mention at least one of the keywords from the list of health-related key terms), which maps to 6.8% and 6.5% of climate change specific tweets explicitly mentioning health-related terms for English and non-English languages correspondingly.

In the cross-country comparison, we find that even with a relatively small and somewhat biased geolocated sample of tweets, we do observe some heterogeneity across different countries (**Figures 5.4-5.5** shows the aggregated results on the intensity of the public engagement in the rhetoric about climate change and health). This can serve as a proxy on how much attention is being paid to the climate change and health co-benefits among the public.

These results can be correlated with the economic development of the states and generally with the public engagement about social issues on Twitter.

Box 5.1 Health specific keywords from an official EEA glossary term for each language.

aerobic condition	health hazard
aerobic process	health legislation
AIDS	health protection
allergen	health regulation
allergy	health-related biotechnology
alveolus	health risks
anatomy	health service
antibiotic	hearing acuity
antibody	hearing impairment
birth control	hearing procedure
blood	hearing protection
breast milk	hearing
calcium	hearing system
cancer	hospital
cancer risk	human biology
cardiology	human body
cardiovascular disease	human disease
cardiovascular system	human health
cell	human pathology
Chagas' disease	human physiology
cleansing	human well-being
clinical symptom	hygiene
cytotoxicity	immunological disease
decontamination	immunology
detoxification	impregnation
dialysis	industrial medicine
disease	infant mortality
disease cause	infection
disinfection	infectious disease
dosage	injury
dose	insurance
drug abuse	intoxication
drug	laboratory test
effect on health	leukaemia
elderly person	lymphatic system
endocrine system	malaria
endocrinology	malnutrition
enterovirus	medical science
environmental health	medicinal plant
environmental health hazard	medicine
environmental health impact assessment	mental effect
environmental health protection	metabolism
environmental health risks	mortality
environmental medicine	muscular system
epidemic	nausea
epidemiology	necrosis
germ	nervous system
haematology	neurotoxicity
health	nutrition
health care	nutritive value of food
health-care activities waste	occupational disease
health care profession	occupational health
health effect of noise	occupational health care
health-environment relationship	occupational medicine
health facility	occupational safety

occupational safety regulation
onchocerciasis
oncology
organ
orphan disease
oxygen deficiency
pandemics
pathology
pharmaceutical industry
pharmacokinetics
pharmacology
physical treatment
physiological change
physiology
poison
poisoning
prescription
preventive health measure
primary treatment
psychic effect
psychological effect
psychological stress
psychology
psychosomatic effect
psychosomatic illness
public health
pulmonary disease
radiation sickness
rapid test
recreation
rehabilitation
respiration

respiratory disease
respiratory system
respiratory tract
salmonella
seizure
self-help programme
self-monitoring
side effect
side effects of pharmaceutical drugs
sleep disturbance
social welfare
spasmodic croup
stress
survival
teratogen
teratogenesis
teratogenesis screening
teratogenicity
teratogenic substance
therapy
toxic effect
toxicity
toxicology
tumour
ultrasound
virology
virus
vitamin
work accident
X-ray

Box 5.2 List of excluded keywords from an official EEA glossary terms for each language.

English: accident, acid, act, additive, adhesive, administration, administrative body, administrative boundary, administrative competence, administrative court (administration), administrative deed, administrative fiat, administrative instructions, administrative jurisdiction, administrative law, administrative occupation, administrative organisation, administrative procedure, administrative sanction, adult, adult education, advertisement, advice, africa, age, agreement (administrative), agreement (contract), agreement (legal), agriculture, air, airport, alarm, alcohol, alignment, allocation, allocation plan, allowance, altitude, americas, analysis, analysis programme, angling, animal, appeal, approach, approval, archaeology, architecture, art, ash, asia, assay, atlas, atmosphere, attribute, attribution, audit, authorisation, authority body, banking, bank (land), barents sea, base (chemical), battery, bay, beach, bee, beetle, behaviour, beverage, bibliography, biology, bird, black sea, boiler, book, border, breeding, brick, bridge, brook, budget, budget policy, bug, building, built-up area, bureaucratisation, bus, business, bus station, butterfly, by-catch, by-product, cable, calculation, calibration, camp, camping, canal, car, caribbean area, caspian sea, catalysis, catalyst, catastrophe, cattle, cave, cell (energy), cellulose, census survey, central africa, central america, central asia, central government, centralisation, channelling, chart (act), chart (nautical), chemical, chemistry, chestnut, child, chimney, church, citizen, city, city centre, cliff, climate action bonds, cloud, coast, coating, code, coke, cold, colour, communication, commuting, comparison, compensation, competition (biological), compression, compressor, concession, conductivity, conflict, congress, conservation, consultancy, consultation, consumption, container, continent, contract, convention, cork, corridor, cost, country lodge, county, court, court of justice, court of justice of the european communities, cove, covering, craft, credit, credit assistance, credit policy, creek, crime, criminality, criminal law, crocodile, cruising, cultivation, culture (society), curriculum, customs, cutting (forestry), cutting (vegetative propagation), dairy product, dam, damage, dating, debt, decision, decomposition, decree, deer, defence, degradation, delta, demand, democracy, demography, density, deposition, deregulation, desert, design (project), detection, detector, detergent, deterrence, deterrent, devolution, dictionary, diffusion, digester, digestion (sewage), directive, disaster, disinvestment, dispersion, dissolution, distribution, ditch, doctrine (law), document, documentary film, documentation, dog, draining, drawing, drilling, drying, dumping, dust, dye, dyke, east africa, eastern asia, eastern europe, east-west relations, east-west trade, ec council of ministers, economics, economy, ec policy, ec regulation, ec treaty, education, educational institution, educational path, educational planning, educational system, education policy, effect, egg, elasticity, electronics, emancipation, employment, encyclopaedia, enforcement, engine, engineering, enrichment, equipment, ethics, ethnology, ethology, europe, european court of justice, european monetary fund, european monetary system, european parliament, european standard, european union, evaluation, evaluation criterion, evaluation method, evaluation of technology,

evolution, exceptional tax, exchange policy, excise, executive order, exhibit, expenditure, experiment, experimental study, expert system, exploration, explosion, explosive, export, export licence, exposure, expropriation, externality, extraction, fabric, factor market, fallout, family, family law, farm, fault, federal authority, federal government, federal law, fee, feedback loop, fen, field, field experiment, field study, film, filter, finances, financial aid, financial assistance, financial compensation, financial contribution, financial fund, financial instrument, financial law, financial management, financial market, financing, fine, fire, firing, firm, fish, fitting (plumbing), flavouring, flea, flow, fog, food, forecast, forecasting, foreign economic relations, foreign policy, foreign trade, foresight, fountain, freedom, free movement of capital, free trade, frog, frost, fruit, fur, furniture, game (animals), game (play), garden, gas, gender issue, geography, glacier, glass, glaze, glossary, glue, goal of individual economic business, golf, goods, goods and services, government advisory body, government building, government (cabinet), government contracting, government liability, government policy, grain, grass, gridding, grinding, gross domestic product, gross national product, group behaviour, gulf, hail, handicraft, harbour, hardness, harvest, hazard, hazard area, haze, highway, hiking trail, hill, historical evolution, historical monument, historical research, historical site, historic centre, history, holiday, holiday camp, home garden, homepage, horse, hotel industry, hot water, housing, housing density, housing finance, housing improvement, housing legislation, housing need, housing programme, housing quality standard, humanitarian aid, hunting, ice, ideology, image classification, image enhancement, image filtering, image processing, immune system, immunity, immunoassay, impact assessment, impact minimisation, impactor, impact prevention, impact reversal, impact source, implementation law, import, import licence, impoverishment, income, income tax, incorporation, indefinite legal concept, index, indexing of documentation, indian ocean, indicator, indicator-based assessment, industrial policy, industry, infant, infiltration, informatics, information, information centre, information clearing-house, information exchange, information infrastructure, information kit, information network, information processing, information service, information source, information system, information technology, information technology industry, information transfer, infrastructure, infrastructure for spatial information in europe, inhabitant, initial training, ink, inner city, innovation, insect, inspection, inspection of records, inspection service, institutional activity, institutionalisation, institutional structure, instrumentation, insurance business, insurance coverage, interest, interest group, interim decision, interlaboratory comparison, inter-library loan, intermediate goods, intermediate product, internal european market, internal migration, international agreement, international assistance, international balance, international competitiveness, international conflict, international convention, international co-operation, international court of justice, international distribution, international division of labour, international economic law, international environmental relations, international harmonisation, international law, internationally important ecosystem, international monetary fund, international organisation, international politics, international relations, international river basin, international safety, international standardisation, international trade, international transaction, international watercourse, internet, internet search service, internet service provider, interpolation, interpretation method, intervention fund, inventory, inversion, investment, ion, island, joint implementation (rio conference), judgement (sentence), judicial assistance, judicial body, judicial system, judiciary rule, juridical act, jurisdiction, jurisprudence, justice, labelling, laboratory, laboratory experiment, laboratory research, laboratory technique, laboratory waste, labour, labour force, labour law, labour market, labour relations, lake, lamp, land, landscape, laser, latitude, laundering, law amendment, law branch, law (corpus of rules), law draft, law enforcement, law (individual), law relating to prisons, law (science), lead, leaf, leakage, lease, leather, legal basis, legal form of organisations, legally protected right, legal procedure, legal profession, legal regulation, legal remedy, legal system, legal text, legislation, legislative authority, legislative competence, legislative information, legislative procedure, legislative process, legislature, leisure activity, leisure centre, leisure time, less developed country, level of education, lexicon, liability, liability legislation, library, library service, licencing, licencing obligation, licencing procedure, life cycle, life-cycle management, lifestyle, light, lighting, lime, limit value, line, linear economy, literature, literature data bank, literature evaluation, literature study, litigation, litter, lizard, local authority, local building material, local development, local finance, local government, local government policy, local passenger service, local recreation, local traffic, location of industries, locomotive, lodging, longitude, long-term effect, long-term experiment, long-term transition, long-term trend, loss, lower house, machinery, macroeconomic goal, macroeconomics, mailing list, maintenance (technical), major accident, major risk, major risk installation, mammal, management, management accounting, management contract, management plan, management technique, mandate, manpower, man (society), manufacturing activity, manufacturing trade, manure, map, map chart, mapping, marble, marina, marine strategy framework directive, marital status, maritime law, maritime navigation, maritime transport, marker, market, market economy, market form, market gardening, marketing, market price, market research, market study, mass media, mass recreation, mass transport (physics), material, measuring, measuring instrument, measuring method, measuring programme, meat, mediterranean area, mediterranean forest, mediterranean sea, mediterranean wood, melting, mercury, metal, meteorology, method, methodology, metropolis, microwave, migrant labour, military activities, military air traffic, military aspects, military equipment, military zone, milk, mill, mine, mineral, ministry, ministry building, minority, miscellaneous product, mist, mite, mixed farming, mixing, mobile home, model, modelling, mode of transportation, moisture, monetary assessment, monetary economics, monetary relations, money market, monitoring, monitoring criterion, monitoring data, monitoring equipment, monitoring network, monitoring station, monitoring system, monitoring technique, monopoly, monument, morphology, mosaic, motorcycle, motor vehicle, motorway, mountain, mountain range, mountain refuge, mountain resort, mowing, mud (sediment), multilateral agreement, multimedia technology, multinational firm, multiple use management area, multispectral scanner, municipality, municipal law, municipal level, museum, mushroom, music, mutant, mutation, myth, national accounting, national boundary, national conservation programme, national economic costs, national economy, nationalisation, national legislation, national park, national planning, national reserve, natural area, natural capital, natural capital accounts, natural material, natural monument, natural resource, natural resource conservation, natural risk, natural risk analysis, natural risks prevention, natural scenery, natural science, natural stone, natural value, nature-based solution, navigation, navigational hazard, need, negotiable charge, neighbourhood improvement scheme, neighbourhood law, neighbourhood noise, nesting, nesting area, net resource depletion, netting policy (emissions trading), neutralisation, new community, new installation, new material,

newsgroup, newsletter, new technology, new town, noise, noise level, noise map, noise measurement, noise monitoring, noise type, nomenclature, non-metal, non-residential building, norm, normalisation, north africa, north america, north atlantic ocean, north pacific ocean, north-south relationship, notice, notification, nuisance, nursery garden, nursery (plant breeding), nutrient, oak, objection, objective well-being, obligation to inform, obligation to label, observation satellite, occupation, occupational group, occupational status, ocean, oceania, odonate, odour, office, official duty, official hearing, offset policy (emissions trading), off-site, open sea, operating data, opinion, opinion survey, order, ordinance, ore, organisation (law), organisation of teaching, organisation of the legal system, organisation of work, organism, oven, overburden, overconsumption, overcrowding, overturn (limnology), ownership, packaging, paint, painting business, paint room, paint shop, paper, parameter, parliament, parliamentary debate, parliamentary report, participation, patent, path, peat, pedagogy, penalty, permission, pest, pet, philosophy, photograph, photography, physical planning, physical process, physical property, physical science, physics, pipe, pipeline, pixel, plain, plan, planning law, planning measure, planning permission, planning-programming-budgeting system, plant (biology), planting, playground, point, police, police law, police power, policy, policy effectiveness, policy framework, policy guideline, policy implementation, policy instrument, policy integration, policy planning, political counselling, political doctrine, political geography, political ideology, political organisation, political party, political power, politics, polygon, pond, pool, post-treatment, poverty, power company, predator, premium, preserve, press, pressing, press release, pressure, price, prices policy, primary sector, primate, printing work, prior informed consent, private car, private domain, private international law, private law, private sector, private transport, privatisation, procedural law, process analysis, processing, product, product advertising, product comparison, product evaluation, product identification, product information, production policy, productivity, productivity trend, product liability, product life cycle, product standard, professional society, profit, prognostic data, programme, progress line, prohibition, project, promotion of trade and industry, propagation process, property protection, prosecution, prosperity, protocol, protozoan, province, provincial/regional authority (d), provincial/regional law (d), public, public action, public aid, public attendance, public bath, public building, public contract, public debt, public discussion, public domain, public emergency limit, public expenditure, public finance, public financing, public function, public hearing, public information, public inquiry, public institution, public international law, public law, public maritime domain, public opinion, public opinion polling, public park, public participation, public-private partnership, public procurement, public prosecutor's office, public relations, public sector, public service, public utility, public works, pulp, pump, pumping, purchase, quality assurance, quality certification, quality control, quality objective, quality of life, quality standard, racking, radar, radio, radio programme, rag, railway, rain, rape (plant), raster, rate, reactor, reasonableness, recommendation, redress, reed, reef, reference service, referral information, referral information system, reflection, refrigerator, refuge, refugee, region, registration, regulation, reintroduction, relational database, relief (land), religion, removal, rental housing, repair business, replacement, replacement cost, reporting process, report to the minister, representation, repression, reprocessing, reproduction (biological), reptile, rescue service, rescue system, research, research centre, research of the effects, research policy, research project, reserve, residential area, residential area with traffic calmings, residential building, residual risk, resilience, resin, resistance (biological), resolution (act), resolution (parameter), resource, resource use, resource utilisation, responsibility, resting form, restoration, retail trade, rice, right of access, right of property, rights, rights of future generations, rights of the individual, right to compensation, right to information, ringing (wildlife), rinsing, rising (geological), risk, risk analysis, risk assessment, risk-based change, risk-benefit analysis, risk communication, risk exposure, risk exposure plan, risk management, risk perception, risk reduction, river, road, rock, rodent, root, route, route planning, rubber, ruling, running wild, runoff, safety, safety analysis, safety measure, safety rule, safety standard, safety study, safety system, salamander, salt, salvage, sampling, sampling technique, sanction, sand, sanitation, satellite, satellite account, satellite image, saving, school, school life, school teaching, schoolwork, science, scientific and technical information, scientific committee, scientific co-operation, scientific dispute, scientific policy, scientific research, scoping procedure, sea, seagrass, sealing, seal (technical), season, secondary education, secondary sector, second-hand goods, sectoral assessment, security of installations, sediment, seed (biology), seed (product), seizure of profits, selection of technology, sensitive area, sensitivity analysis, sensor, separation, separator, service area, services, sewage, shellfish, shelter, ship, shop, shopping centre, show, shredder, shrub, silver, sizing, skiing, skin, slag, sleep, slope, sludge, small islands (political geography), smoke, smoking, snake, snow, snowslide, soaking, soap, social analysis, social behaviour, social bond, social cohesion, social condition, social cost, social development, social differentiation, social dynamics, social equity, social facility, social framework, social group, social indicator, social inequality, social medicine, social-minded behaviour, social movement, social participation, social policy, social problem, social process, social protection, social psychology, social relief, social representation, social science, social security, social service, social structure, social survey, social system, social value, society, socio-cultural group, socioeconomic factor, socioeconomics, socioeducational activity, sociological survey, sociology, sociopolitical aspect, softening, software, software development, soil, sound, sound level, sound measurement, soundproofing, south america, south atlantic ocean, southeast asia, southern africa, southern asia, south pacific ocean, space (interplanetary), space policy, space research, space transportation, space travel, spatial distribution, spatial mobility, special authorisation, specialisation (biological), special law, species, speech, speed, speed limit, spider, spillage, sponsorship, sport, sports facility, spring (hydrology, land), square, stable, stack, standard, standardisation, starch, state, state control, state of matter, state of the art, station, statistical analysis, statistical data, statistical information, statistical information system, statistical series, statistics, status of development, statutory declaration, statutory public body, statutory text, steel, sterilisation (biological), sterilisation (process), steroid, stock (biological), stocking, stock management, stocktaking, stock (trade), stone, storage (process), stove, stratification, subject, submarine, subsequent order, subsidence, subsidiary principle, subsidy, substitutability (chemistry), suburb, sugar industry, sugar (product), supervision of building works, supervision of installation, supervisory body, supply and demand, supply (trade), surplus, surveillance, survey, suspended matter, swamp, swans, geese and ducks, sweetener, swell, taking of evidence, tanker (ship), tanker (truck), tar, target group, target setting, tariff, tax, taxation, taxation policy, tax differentiation, tax law, tax on capital, tax on consumption, taxonomy, taxonomy regulation, tax system, teaching, teaching

material, teaching method, technical information, technical instruction, technical regulation, technological accident, technological change, technological development, technological hazard, technological process, technology, technology acceptance, technology assessment, technology transfer, telecommunication, teleheating, telematics, telemetry, television, television programme, terminology, termite, territorial community, territorial government, territorial policy, territory, test, test animal, testing guideline, testing method, test organism, textile, theory of money, theory of the welfare state, thermodynamics, thesaurus, thesis, thickening, threshold value, thunderstorm, tide, timber, time, time allocation, time budget, time-horizon mismatch, tin (element), tissue, tobacco, tobacco smoke, tornado, total parameter, tourism, tourist attendance, tourist facility, touristic activity management, touristic route, touristic unit, touristic zone, tradeable permit, trade activity, trade and consumption, trade barrier, trade (economic), trade impact on environment, trade policy, trade (profession), trade relations, trade restriction, trade (services), trades union, traditional culture, traditional health care, traffic, traffic accident, traffic control, traffic control measure, traffic engineering, traffic infrastructure, traffic jam, traffic monitoring, traffic on water, traffic regulation, traffic route, traffic route construction, train, training, training centre, trajectory, transition, transitional arrangement, transitional settlement, transition element, transportation, transportation business, transportation by pipeline, transportation mean, transportation policy, transport cost, transport law, transport (physics), transport planning, transport regulation, transport system, transposition of directive, travel, travel cost, treaty, tree, trend, trend of opinion, trial, tropics, tunnel, turbine, type of business, type of claim, type of management, type of tenure, united nations, unsupervised image classification, upper house, urban design, urban development, urban development document, urban development law, urban facility, urban flows (resources), urban settlement, valley, vandalism, variety collection, varnish, vector, vector of human diseases, vector to raster, vegetable, vehicle, ventilation, vermin, vibration, video, village, vocabulary, vocational training, volatility, voluntary work, voting, wadden sea, wage system, wall, war, warning system, war victim, waterfall, water (geographic), water (substance), weapon, weather, weed, weight, welding, well, west africa, western asia, western europe, whale, wind, windfall, winter sports resort, woman, woman's status, wood, wool, working condition, working hours, workplace, world, world health organization, world heritage site, world wide web, wreck, write-off, wrongful act, wrongful government act, yeast, yield (agricultural), yield (economy), young, youth, youth work, zoology
**Bulgarian**: all terms from English language translated according to EEA, and additionally - Бор, Тор, Мед
**German**: all terms from English language translated according to EEA, and additionally - Gen, Russ
**Estonian**: all terms from English language translated according to EEA, and additionally - rt, bor, dina
**Finnish**: all terms from English language translated according to EEA, and additionally - suo
**French**: all terms from English language translated according to EEA, and additionally - pré, fer, cation, cap
**Icelandic**: all terms from English language translated according to EEA, and additionally - ál, bú
**Italian**: all terms from English language translated according to EEA, and additionally - lega
**Latvian**: all terms from English language translated according to EEA, and additionally - aka
**Norwegian**: all terms from English language translated according to EEA, and additionally - for, sel, eng, ef, bor, nes
**Polish**: all terms from English language translated according to EEA, and additionally - bor, bar, tal, rak, las
**Romanian**: all terms from English language translated according to EEA, and additionally - bor, lac, zer, iod
**Slovakia**: all terms from English language translated according to EEA, and additionally - íl, jed, lom, les, mys, lak, pec
**Slovenian**: all terms from English language translated according to EEA, and additionally - rt, bor, rak, eter, gen, lak, pes, talij, dim, kolo
**Turkish**: all terms from English language translated according to EEA, and additionally - ısı, yağ, is, kil, gen, bor, eter, alg

Box 5.3 translations of the inequality key terms.

English: "inequality", "inequity", "injustice", "justice", "equity", "equality"
Bulgarian: "неравенство", "несправедливост", "несправедливост", "справедливост", "справедливост", "равенство"
Croatian: "nejednakost", "nejednakost", "nepravda", "pravda", "pravičnost", "jednakost"
Czech: "nerovnost", "nespravedlnost", "nespravedlnost", "spravedlnost", "spravedlnost", "rovnost"
Estonia: "ebavõrdsus", "ebavõrdsus", "ebaõiglus", "õiglus", "võrdsus", "võrdsus"
French: "inégalité", "inéquité", "injustice", "justice", "équité", "égalité"
Finnish: "epätasa-arvo", "epäoikeudenmukaisuus", "epäoikeudenmukaisuus", "oikeudenmukaisuus",
German: "ungleichheit", "ungerechtigkeit", "gerechtigkeit", "gleichheit"
Hungarian: "egyenlőtlenség", "egyenlőtlenség", "igazságtalanság", "igazságosság", "méltányosság", "egyenlőség"
Icelandic: "ójöfnuður", "misrétti", "óréttlæti", "réttlæti", "jafnrétti", "jafnrétti"
Italian: "disuguaglianza", "iniquità", "ingiustizia", "giustizia", "equità", "uguaglianza"
Latvian: "nevienlīdzība", "nevienlīdzība", "netaisnība", "taisnīgums", "vienlīdzība", "vienlīdzība"
Lithuanian: "nelygybė", "nelygybė", "neteisybė", "teisingumas", "teisingumas", "lygybė"
Norwegian: "ulikhet", "ulikhet", "urettferdighet", "rettferdighet", "likhet", "likhet"
Polish: „nierówność", „nierówność", „niesprawiedliwość", „sprawiedliwość", „równość", „równość"
Portuguese: "desigualdade", "injustiça", "justiça", "equidade", "igualdade"
Romanian: „inegalitate", „inechitate", „nedreptate", „dreptate", „echitate", „egalitate"
Slovak: "nerovnosť", "nerovnosť", "nespravodlivosť", "spravodlivosť", "rovnosť", "rovnosť"
Slovenian: "neenakost", "nepravičnost", "nepravičnost", "pravičnost", "pravičnost", "enakost"
Spanish: "desigualdad", "inequidad", "injusticia", "justicia", "equidad", "igualdad"
Turkish: "eşitsizlik", "eşitsizlik", "haksızlık", "adalet", "eşitlik", "eşitlik"

5.3: Political engagement with health and climate change

Indicator 5.3.1: Engagement with health and climate change in the European Parliament

Geographic Coverage of Europe

Each of the 28 EEA countires that participate in the EU parliament.

Data

We downloaded the transcripts of the EU parliament debates (https://www.europarl.europa.eu/) for 2022 and merged it with the data downloaded from previous years for 2014-2022. The 2022 debates added 10283 speeches to the containing 253,839 speeches from previous years. The data was downloaded in XML format and then converted to CSV. The speeches were enhanced with metadata of the various speakers, taken from the database of MEPs available on the same website. We pre-processed and prepared these documents for the application of natural language processing by converting the reports to plain text format as well as translating non-English documents to English texts. We used google_trans_new package (https://pypi.org/project/google-trans-new/), a python API for automatic translation. The package is free to use for academic purposes and uses state-of-the-art neural machine translation developed at google. Further preprocessing involved the removal of stopwords and regularising (lowercasing), and was performed in python with the NLTK package.

Methods

In order to produce the measure of engagement regarding climate change and health in debates of the European Union parliament, we used the publicly available transcripts of the debates. Our approach to produce the indicators is based on identifying the presence of key search terms related to health and climate change within each speech and identifying instances of intersection where terms relating to each field appeared close together, within a window of 25 words. We provide a full list of terms in **Table 5.3**.

Table 5.3 A comprehensive list of terms on health and climate change.

Health terms	Climate change terms
malaria	climate change
diarrhoea	changing climate
infection	climate emergency
disease	climate action
diseases	climate crisis
sars	climate decay
measles	global warming
pneumonia	green house
epidemic	temperature
epidemics	extreme weather
pandemic	global environmental change
pandemics	climate variability
epidemiology	greenhouse
healthcare	greenhouse-gas
health	low carbon
mortality	ghge
morbidity	ghges
nutrition	renewable energy
illness	carbon emission
illnesses	carbon emissions
ncd	carbon dioxide
ncds	carbon-dioxide
air pollution	co2 emission
nutrition	co2 emissions
malnutrition	climate pollutant
malnourishment	climate pollutants
mental disorder	decarbonization
mental disorders	decarbonisation
stunting	carbon neutral
	carbon-neutral
	carbon neutrality
	climate neutrality
	net-zero
	net zero

Inequality Context

Additionally, we searched our samples for terms relating to inequality and injustice, and report on how these terms appear in the context of the health-climate change debate. We focus on the following terms: “inequality” “inequity” “injustice” “justice” “equity” “equality”. We determine how often these terms appear in the intersection window text. We find that in total, these terms only appear twice in the same window as the intersection, in speeches from Germany and Portugal.

Additional analysis

We present some additional findings and figures in this section.

Additional analysis on health and climate Change

Figure 5.8 shows the number of references to health, climate change, and the intersection of health and climate change for the EU parliamentary debates over time. This data is not normalised by number of sessions for each year, and instead provides a general overview of the years. The figure shows that despite the variability in discussing health and climate change individually, there is still a very low engagement with the impact of climate change on health, and its implications.

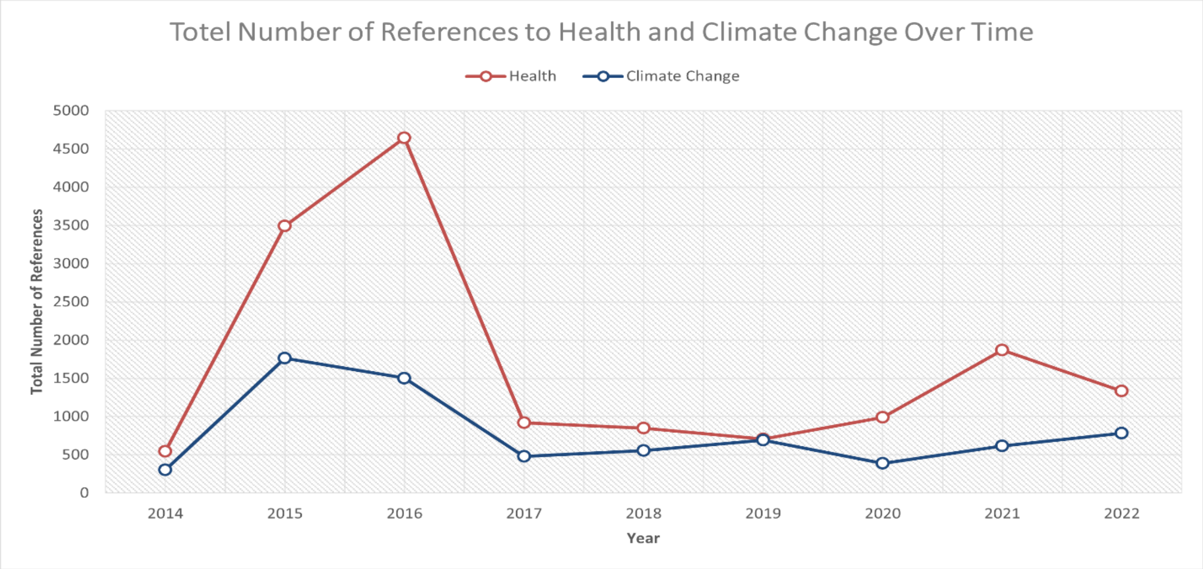

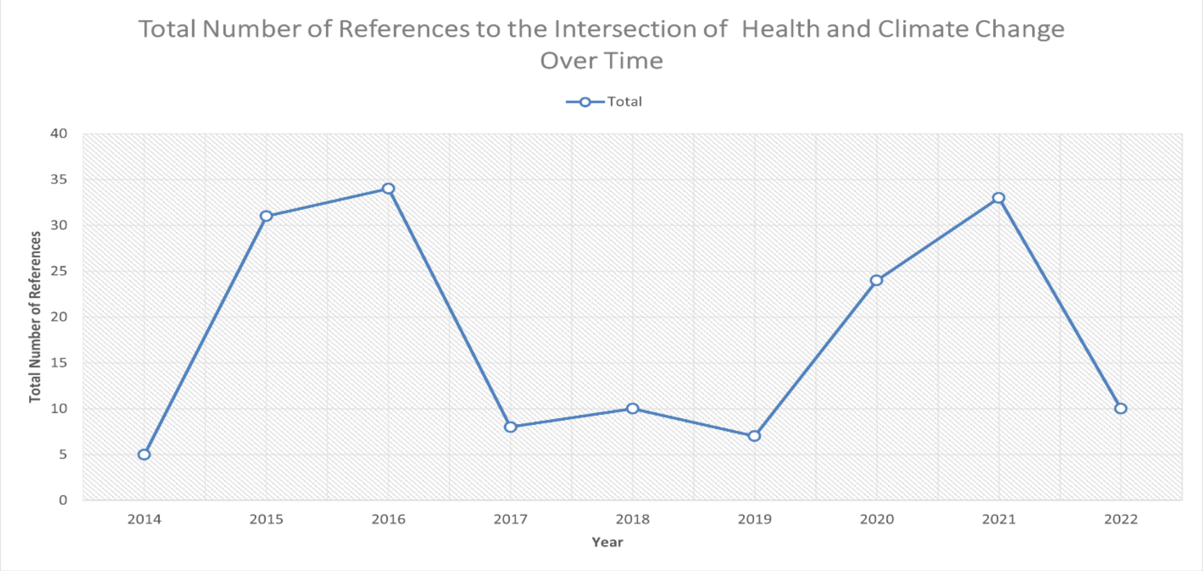

Figure 5.8 Total number of references to health, climate change, and the intersection of health and climate change over time, 2014-2021.

Figure 5.9 presents the frequency of terms related to health, climate change, and the intersection of health and climate change for each political group within the European Parliament. The figure shows relatively low engagement with the impacts of climate change on health, despite a high investment in health from at least two of the participating political groups (EPP and S&D). The health implications of climate change remain largely untouched.

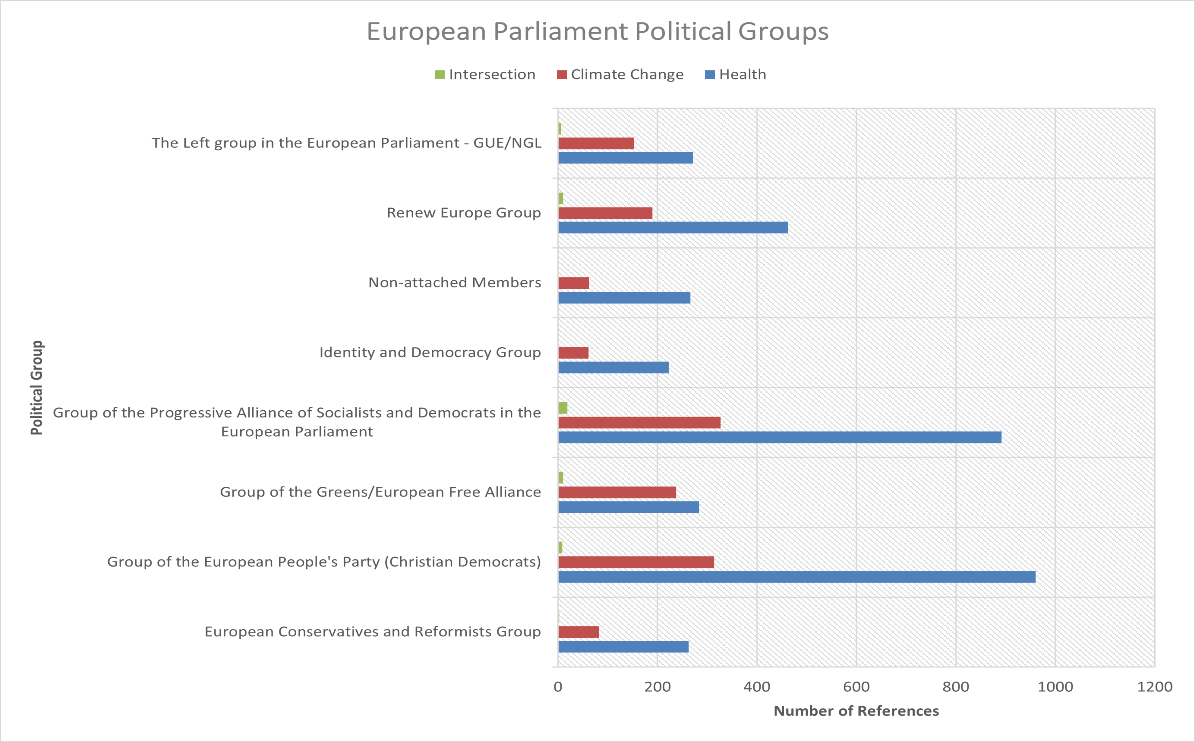

Figure 5.9 Total number of references to health, climate change, and the intersection of health and climate change by political group, 2014-2022.

Figure 5.10 shows the total number of references to health, climate change and the intersection of health and climate change broken down by each participating country. The bar chart shows that despite the high engagement in health terms at the debates, the connection to climate change remains unexplored for most countries, with only Germany, France and Sweden making a small connection.

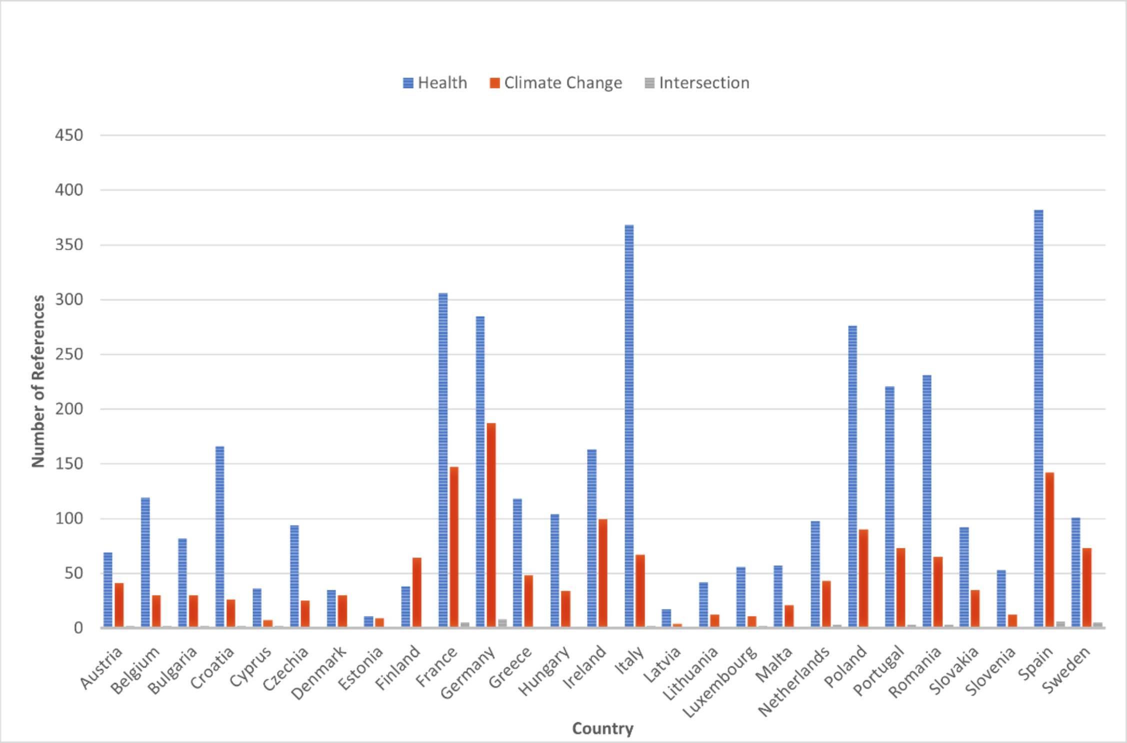

Figure 5.10 Total number of references to health, climate change, and the intersection of health and climate change by country, 2014-2022

Further analysis on health and climate change by country and political party

The figures in this section provide a comprehensive overview of each of the 28 participating EEA countries and the level of engagement of their political parties, showing the total number of references to health and climate change (and their intersection).

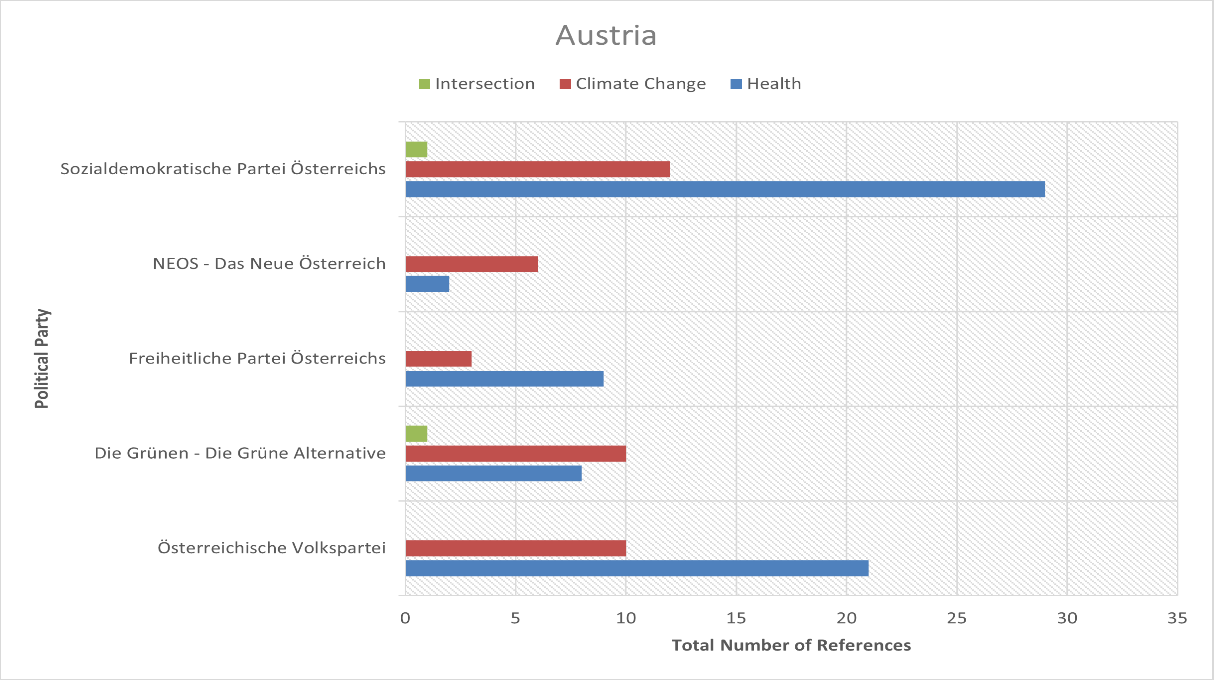

Figure 5.11 Total number of references to health, climate change, and the intersection of health and climate change in Austria, 2014-2022.

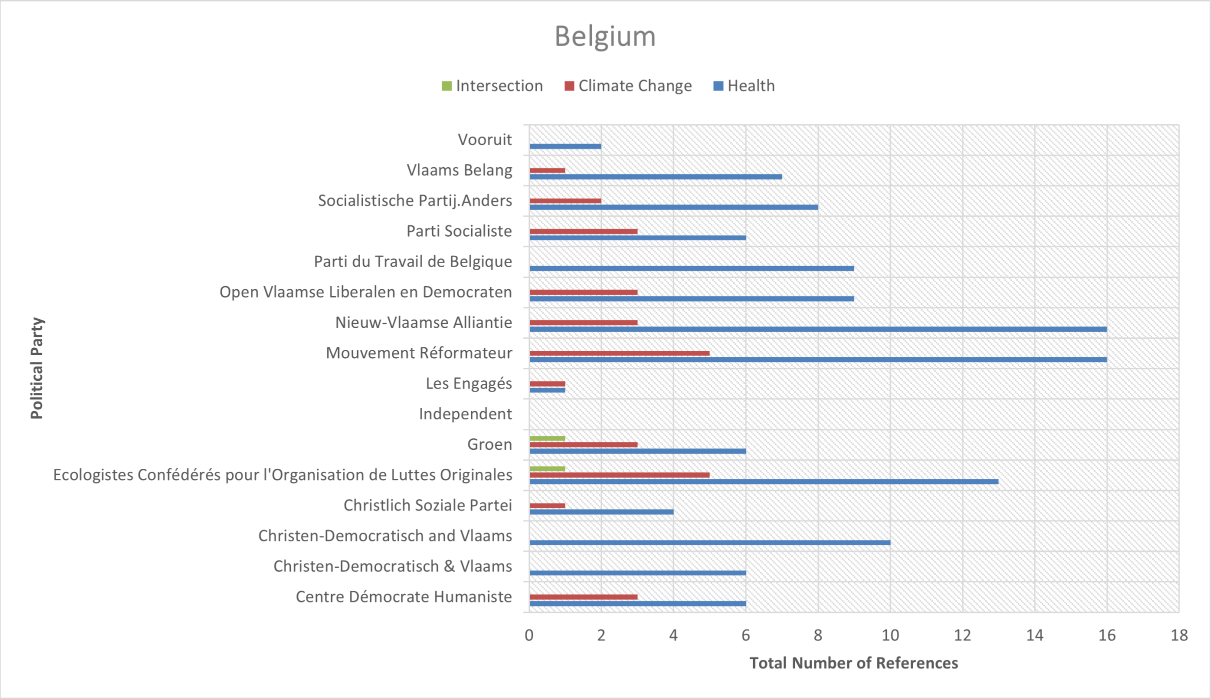

Figure 5.12 Total number of references to health, climate change, and the intersection of health and climate change in Belgium, 2014-2022.

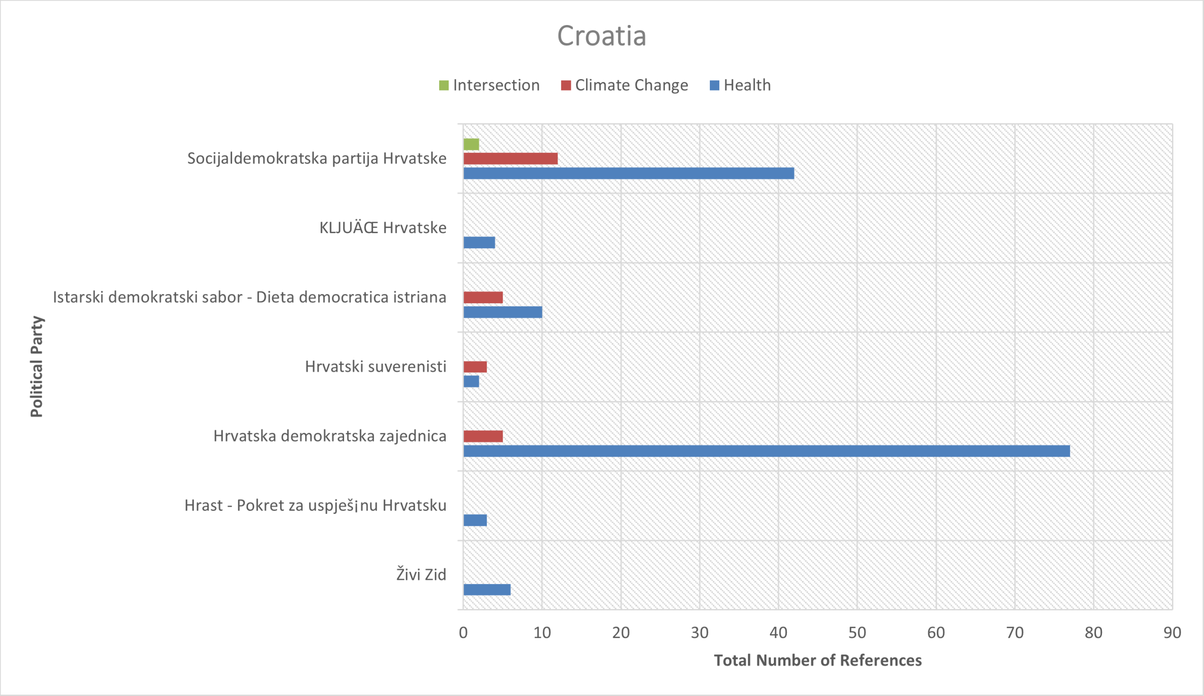

Figure 5.13 Total number of references to health, climate change, and the intersection of health and climate change in Croatia, 2014-2022.

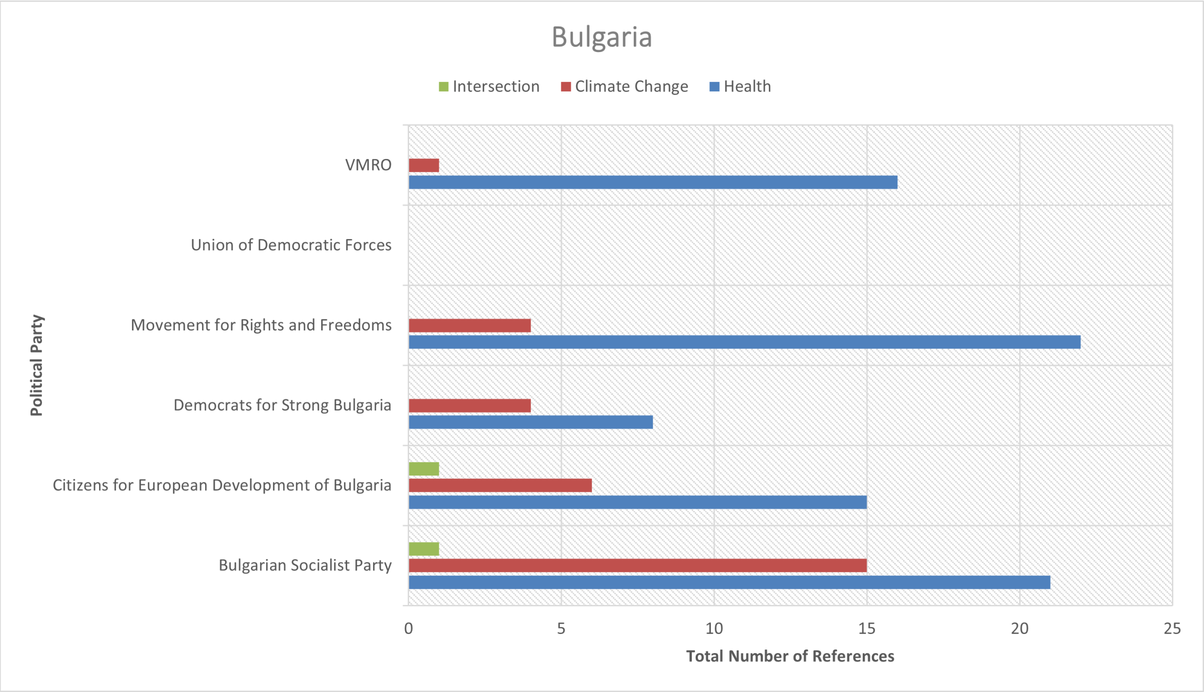

Figure 5.14 Total number of references to health, climate change, and the intersection of health and climate change in Bulgaria, 2014-2022.

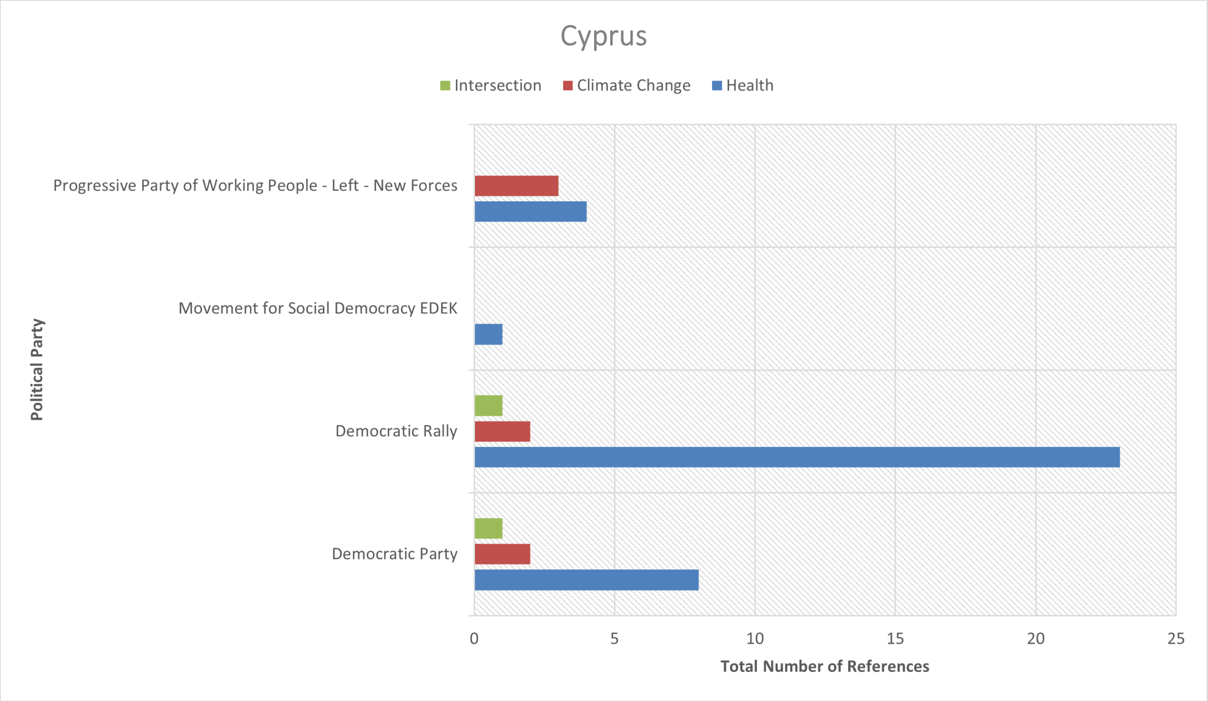

Figure 5.15 Total number of references to health, climate change, and the intersection of health and climate change in Cyprus, 2014-2022.

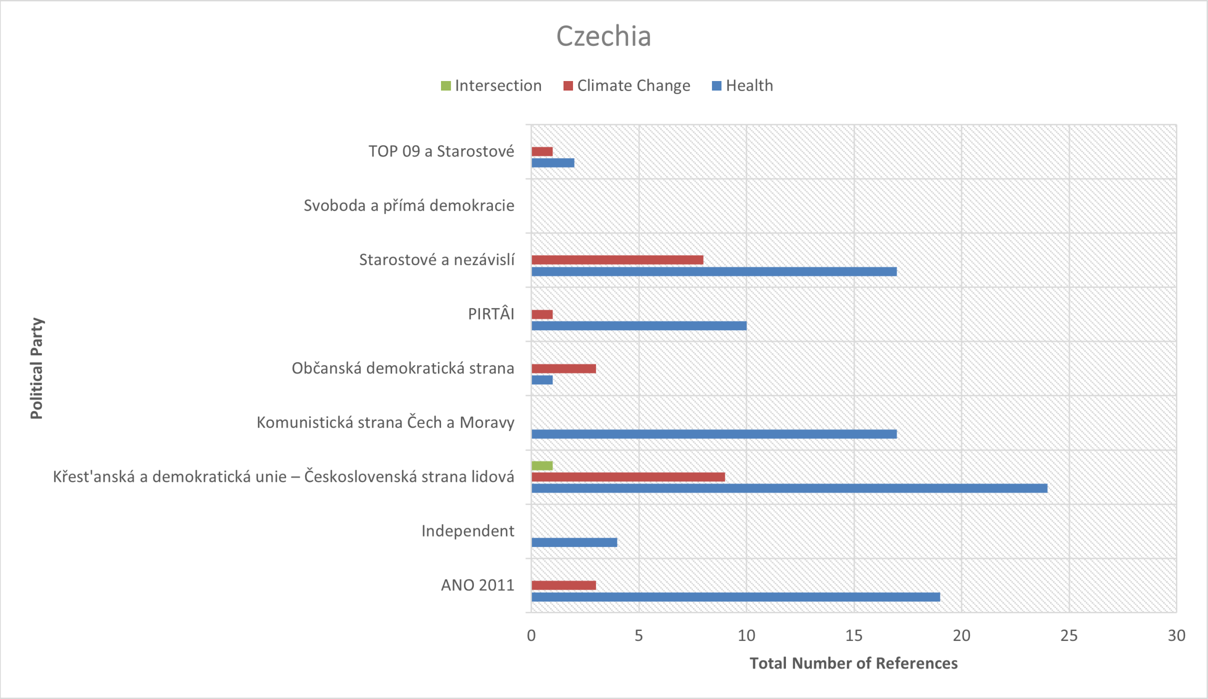

Figure 5.16 Total number of references to health, climate change, and the intersection of health and climate change in Czechia, 2014-2022.

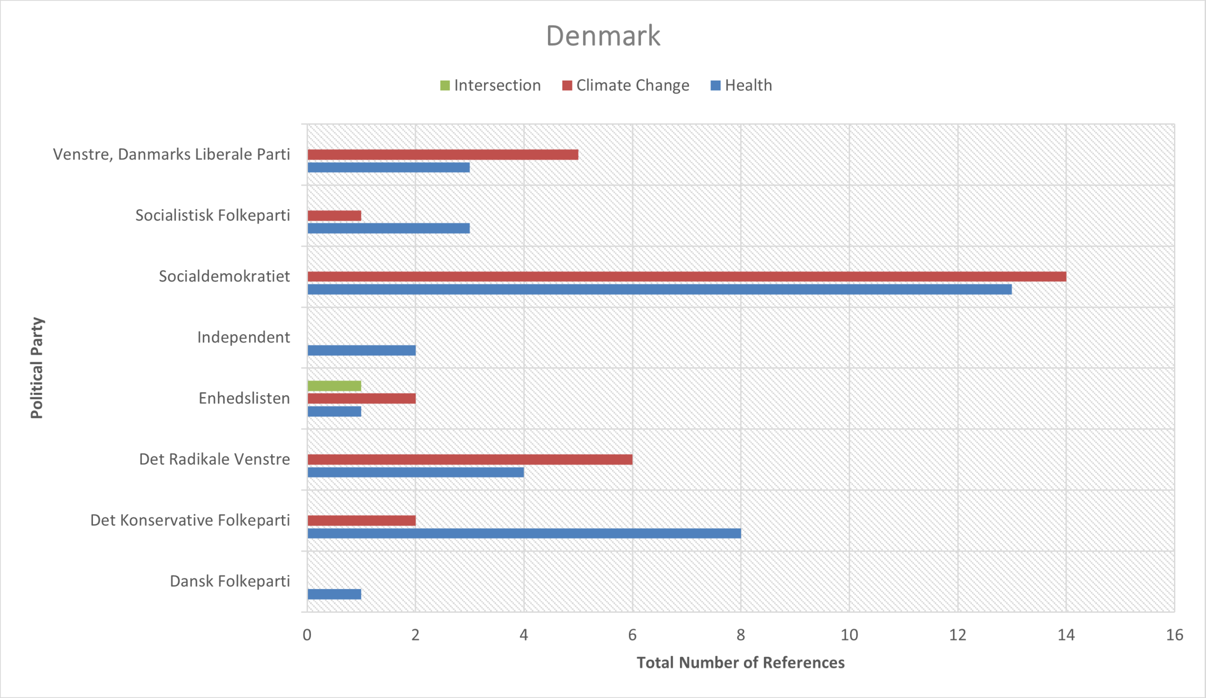

Figure 5.17 Total number of references to health, climate change, and the intersection of health and climate change in Denmark, 2014-2022.

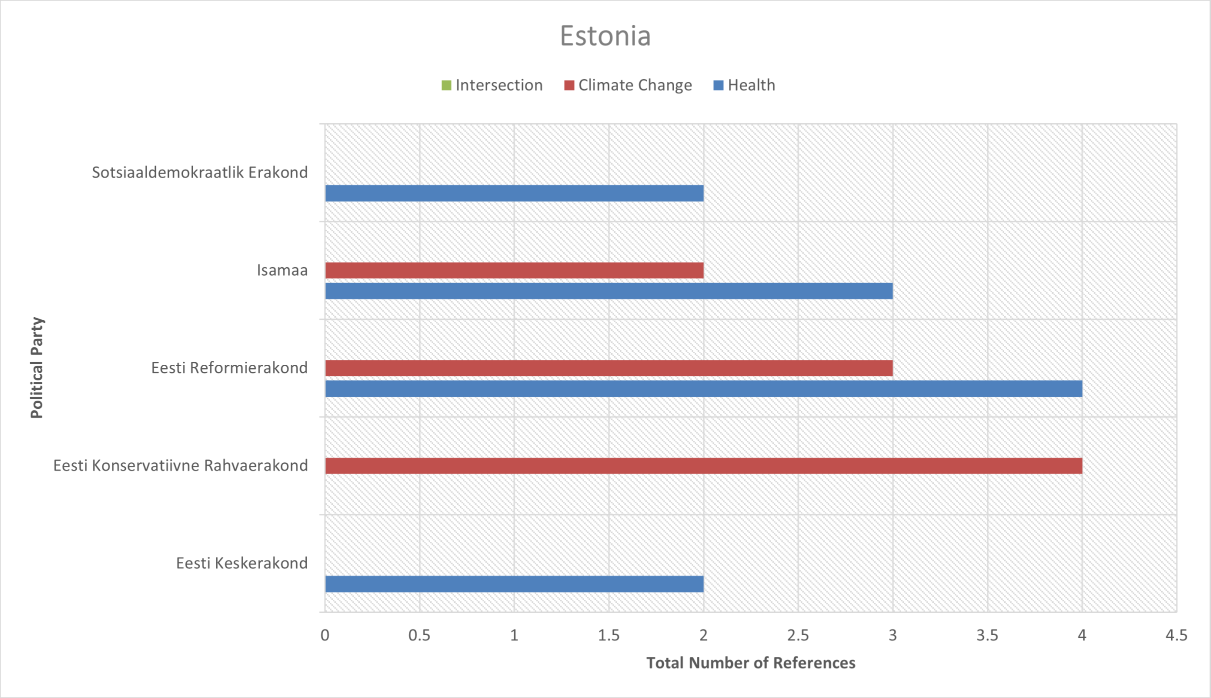

Figure 5.19 Total number of references to health, climate change, and the intersection of health and climate change in Estonia, 2014-2022**.**

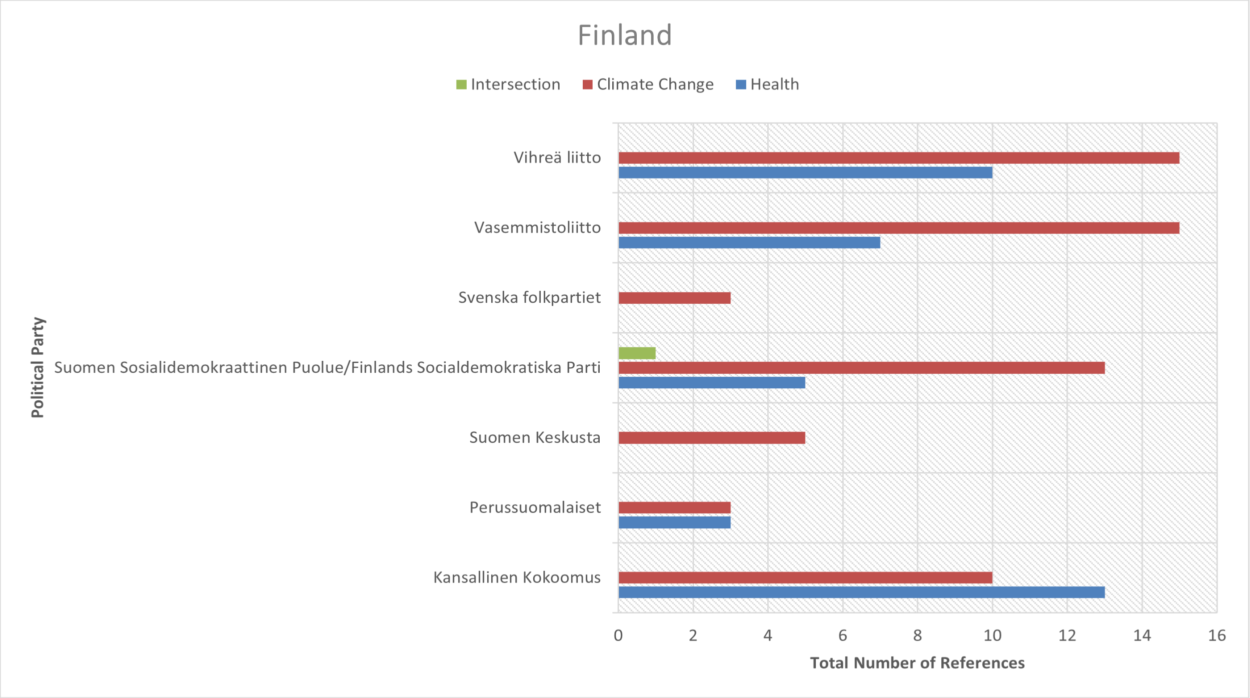

Figure 5.20 Total number of references to health, climate change, and the intersection of health and climate change in Finland, 2014-2022.

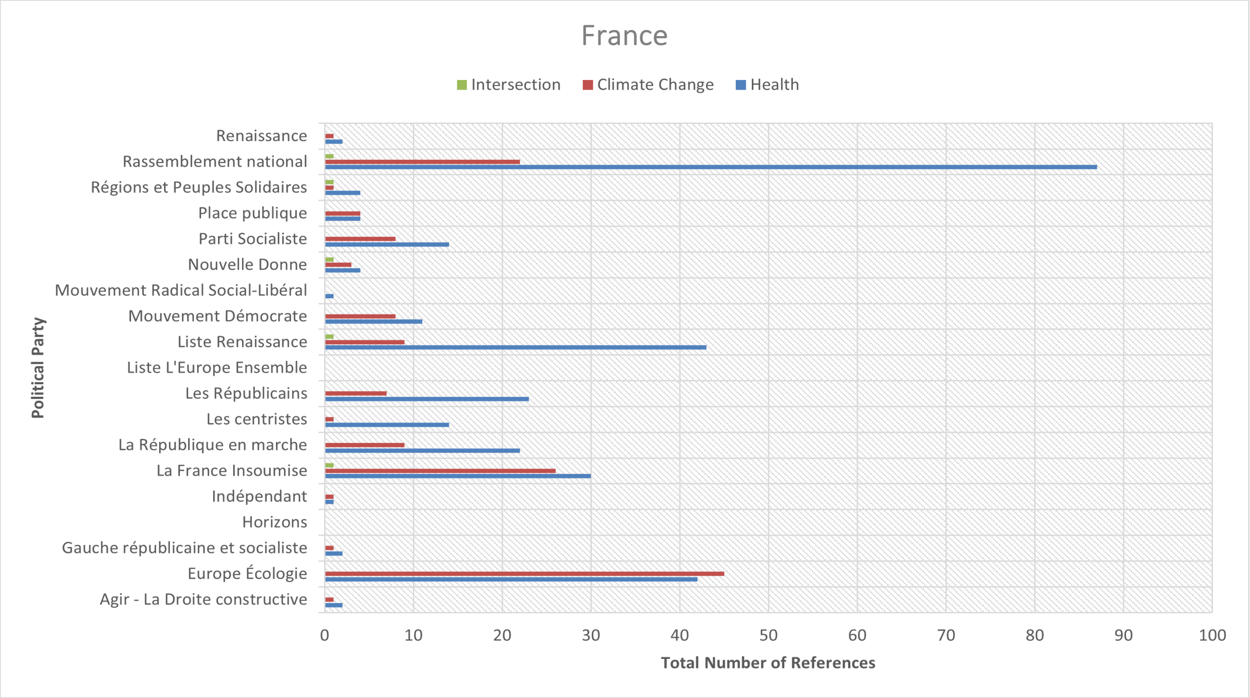

Figure 5.21 Total number of references to health, climate change, and the intersection of health and climate change in France, 2014-2022.

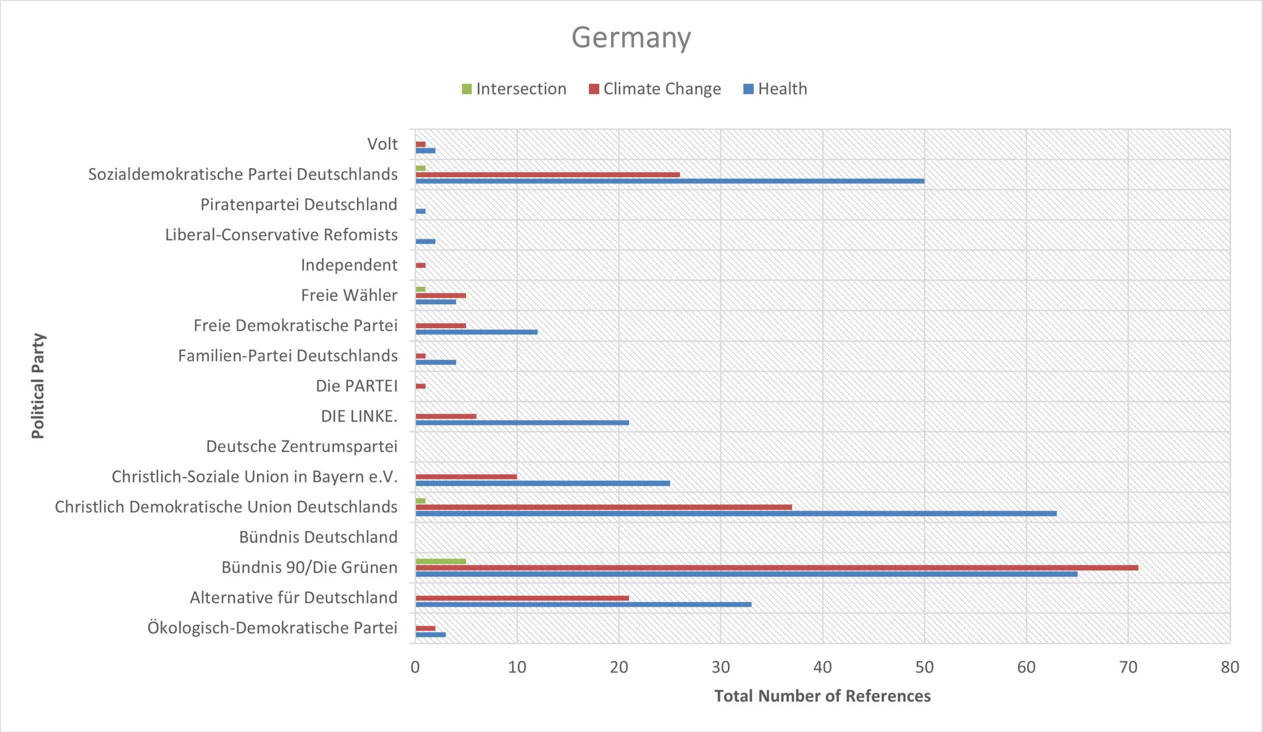

Figure 5.22 Total number of references to health, climate change, and the intersection of health and climate change in Germany, 2014-2022.

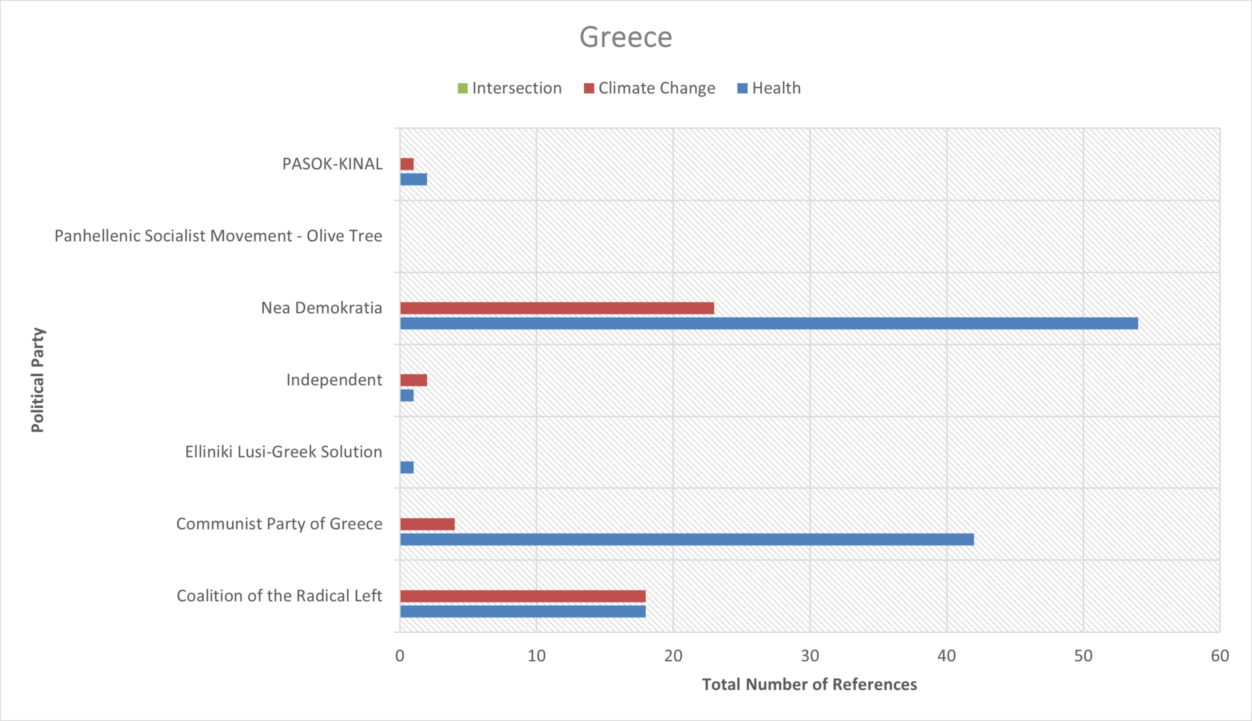

Figure 5.23 Total number of references to health, climate change, and the intersection of health and climate change in Greece, 2014-2022.

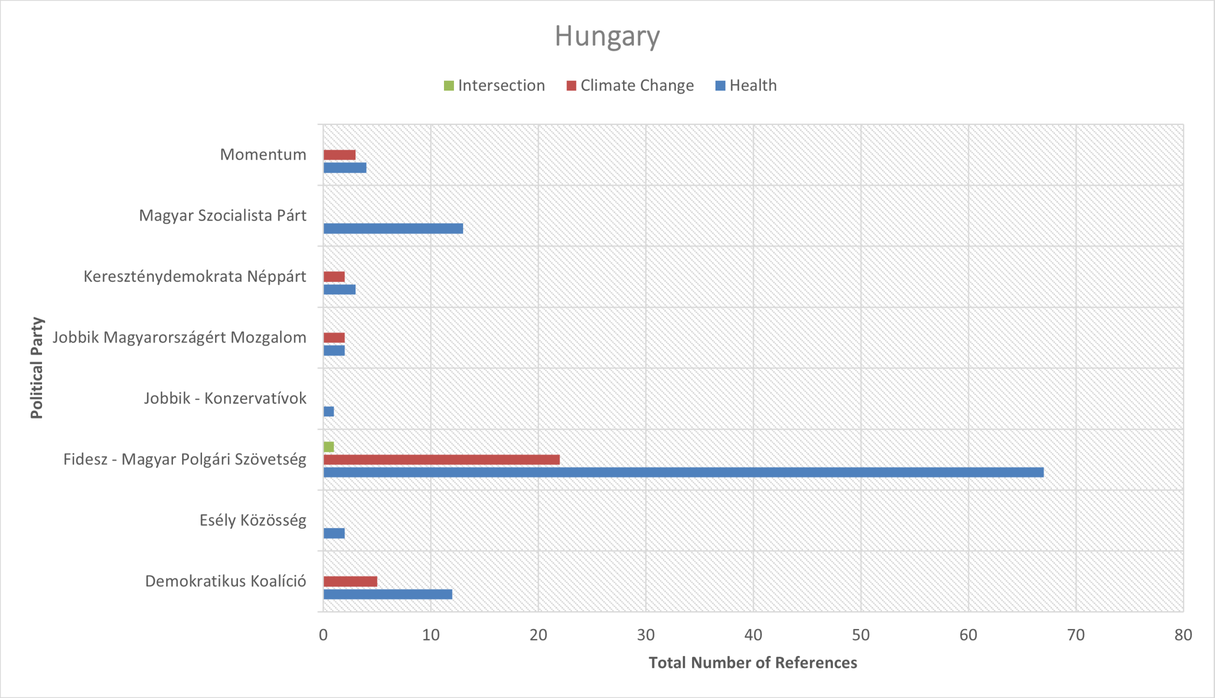

Figure 5.24 Total number of references to health, climate change, and the intersection of health and climate change in Hungary, 2014-2022.

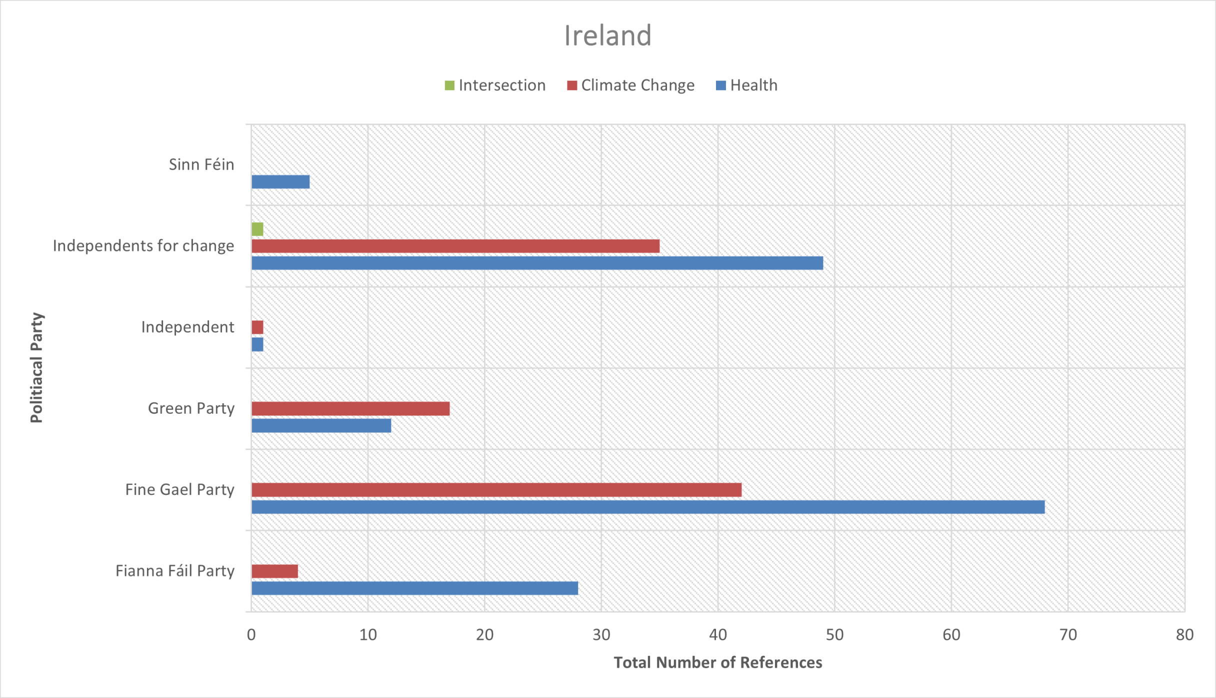

Figure 5.25 Total number of references to health, climate change, and the intersection of health and climate change in Ireland, 2014-2022.

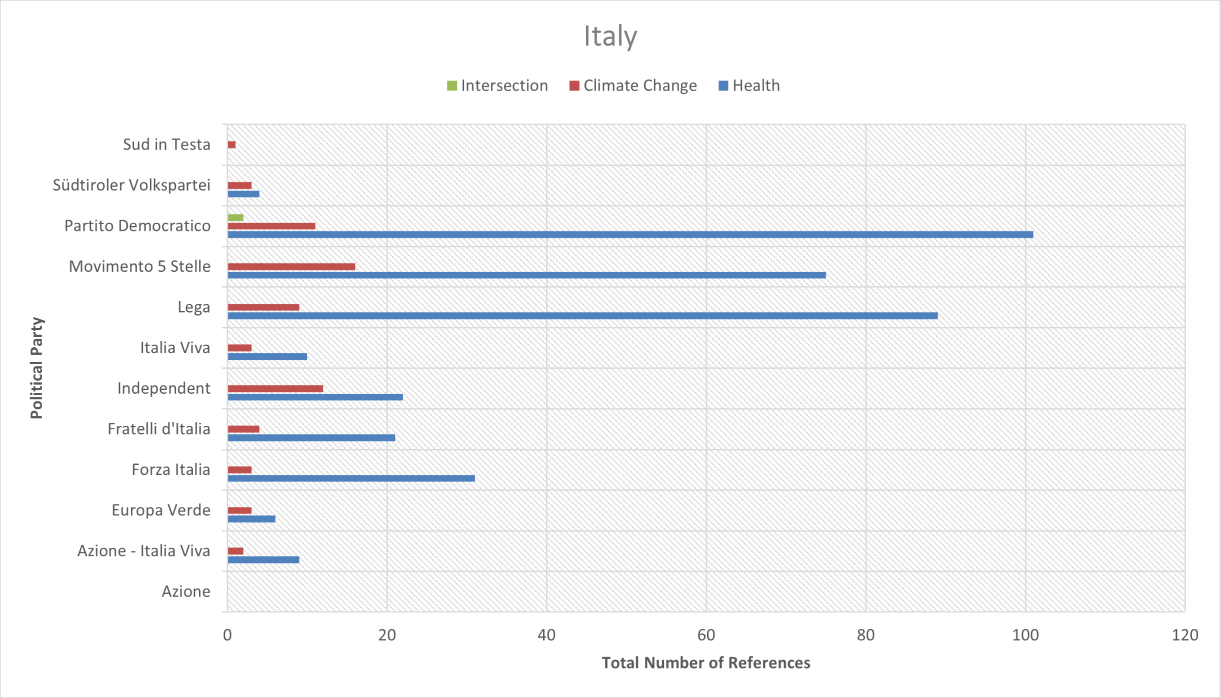

Figure 5.26 Total number of references to health, climate change, and the intersection of health and climate change in Italy, 2014-2022.

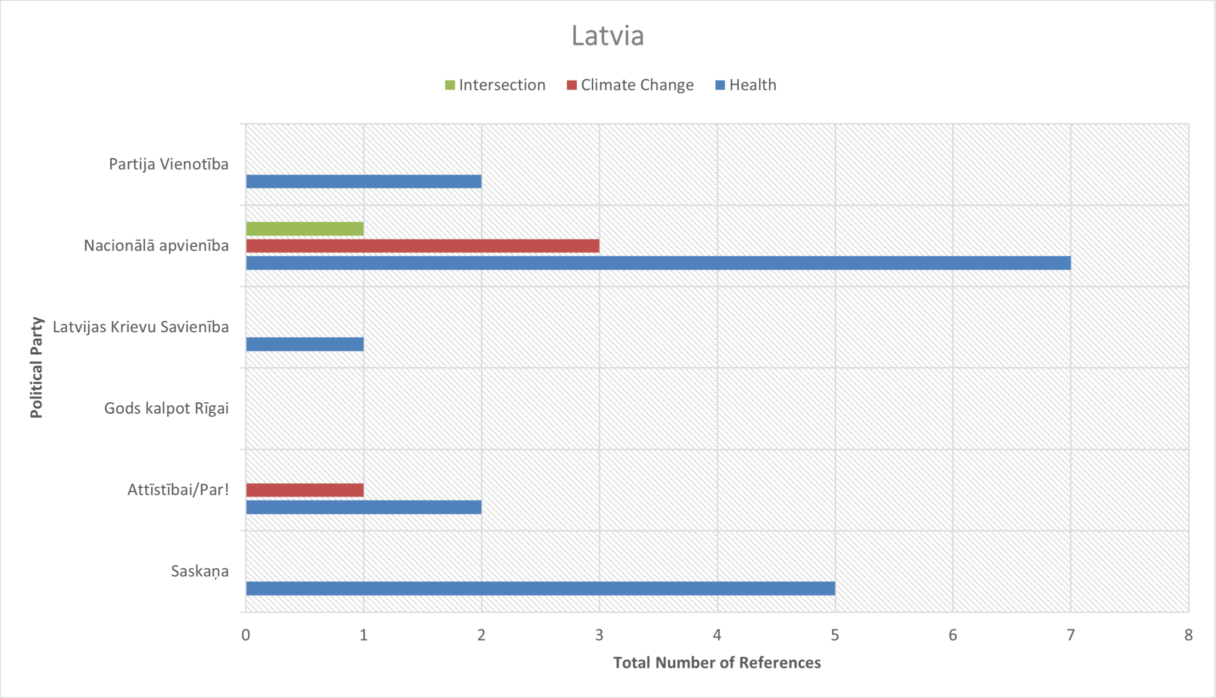

Figure 5.27 Total number of references to health, climate change, and the intersection of health and climate change in Latvia, 2014-2022.

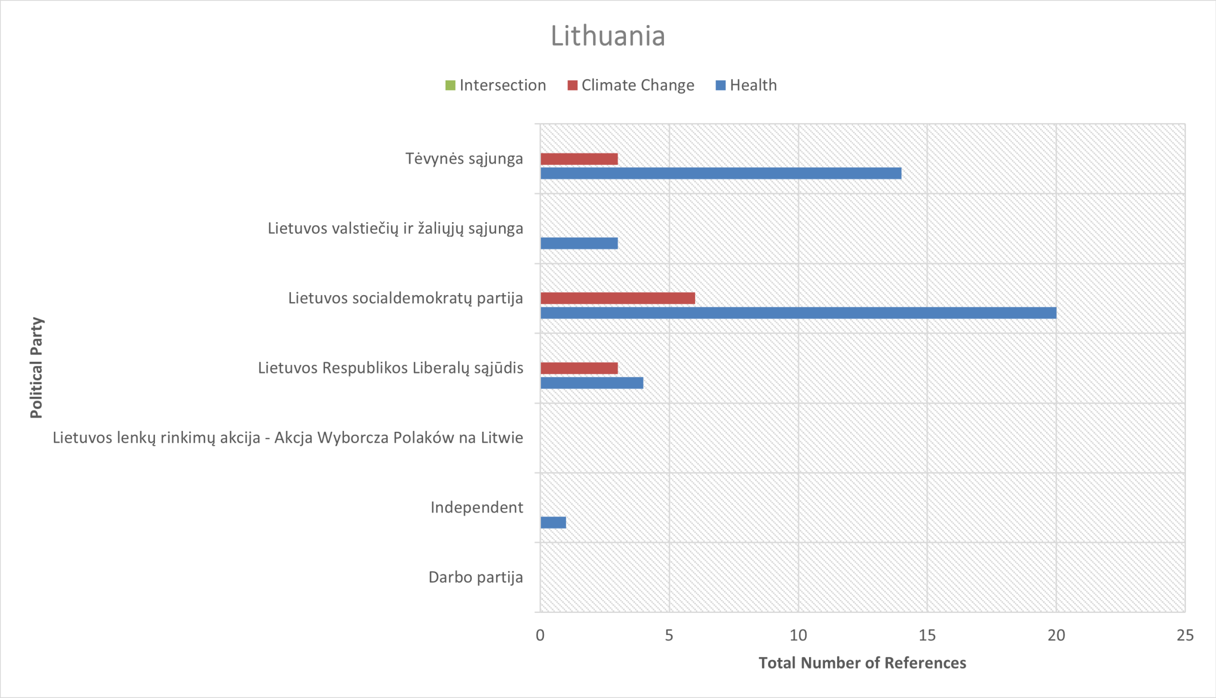

Figure 5.28 Total number of references to health, climate change, and the intersection of health and climate change in Lithuania, 2014-2022.

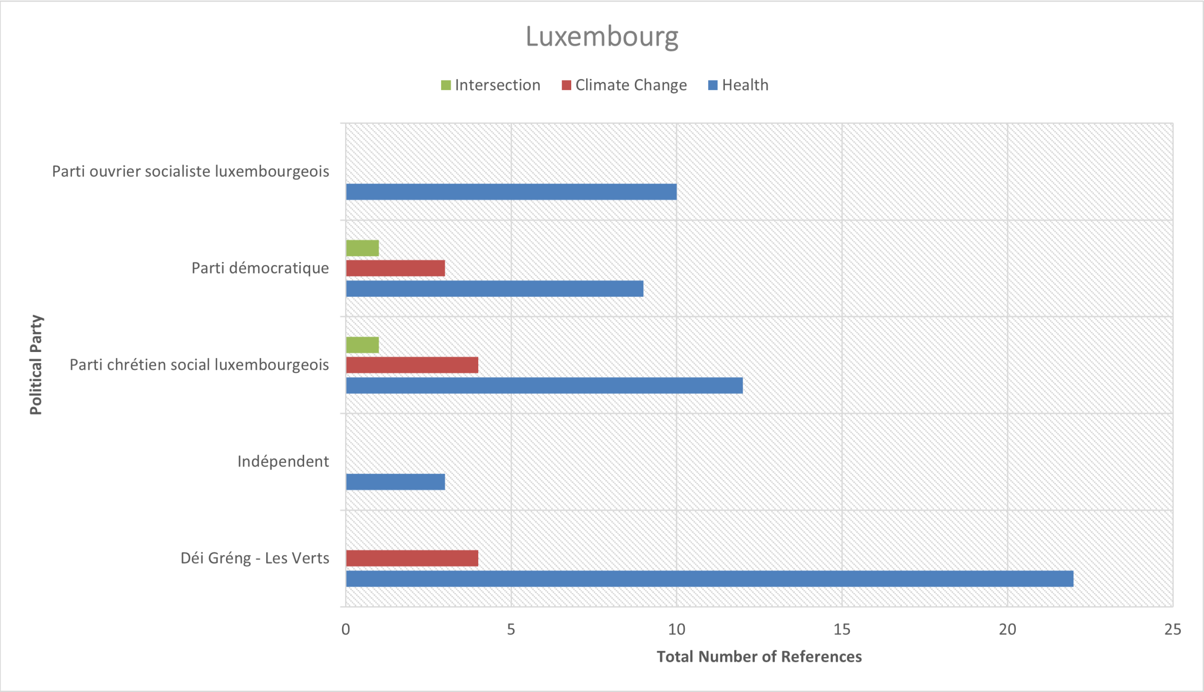

Figure 5.29 Total number of references to health, climate change, and the intersection of health and climate change in Luxembourg 2014-2022.

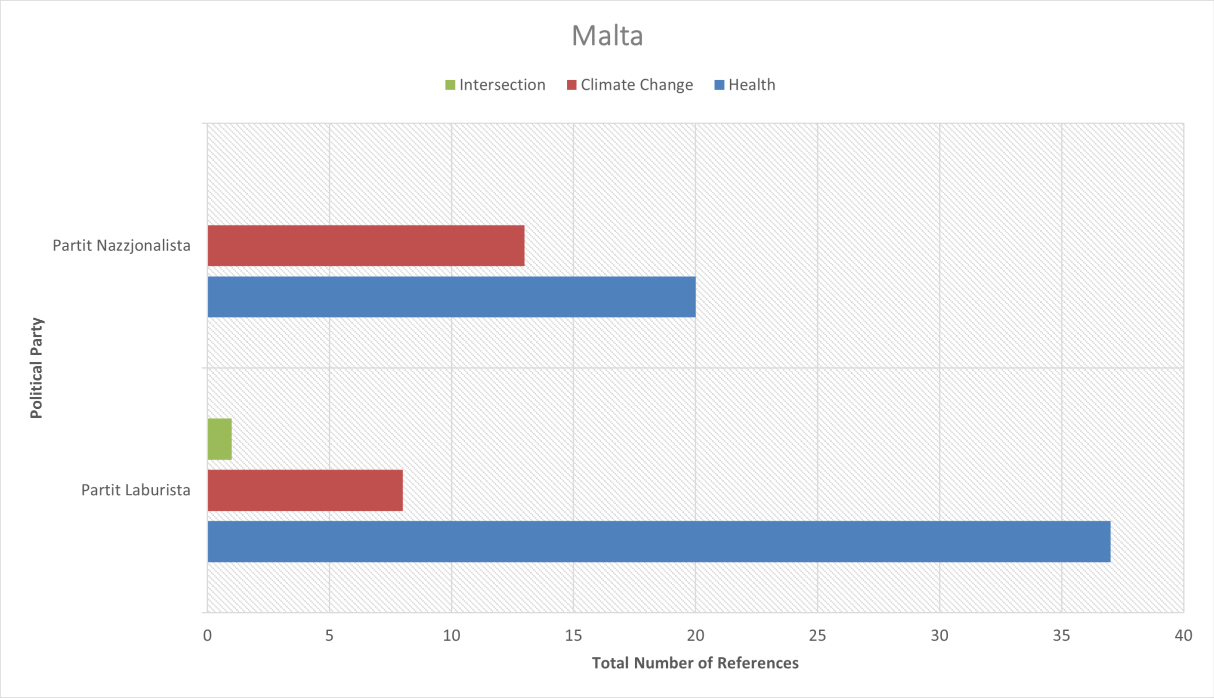

Figure 5.30 Total number of references to health, climate change, and the intersection of health and climate change in Malta, 2014-2022.

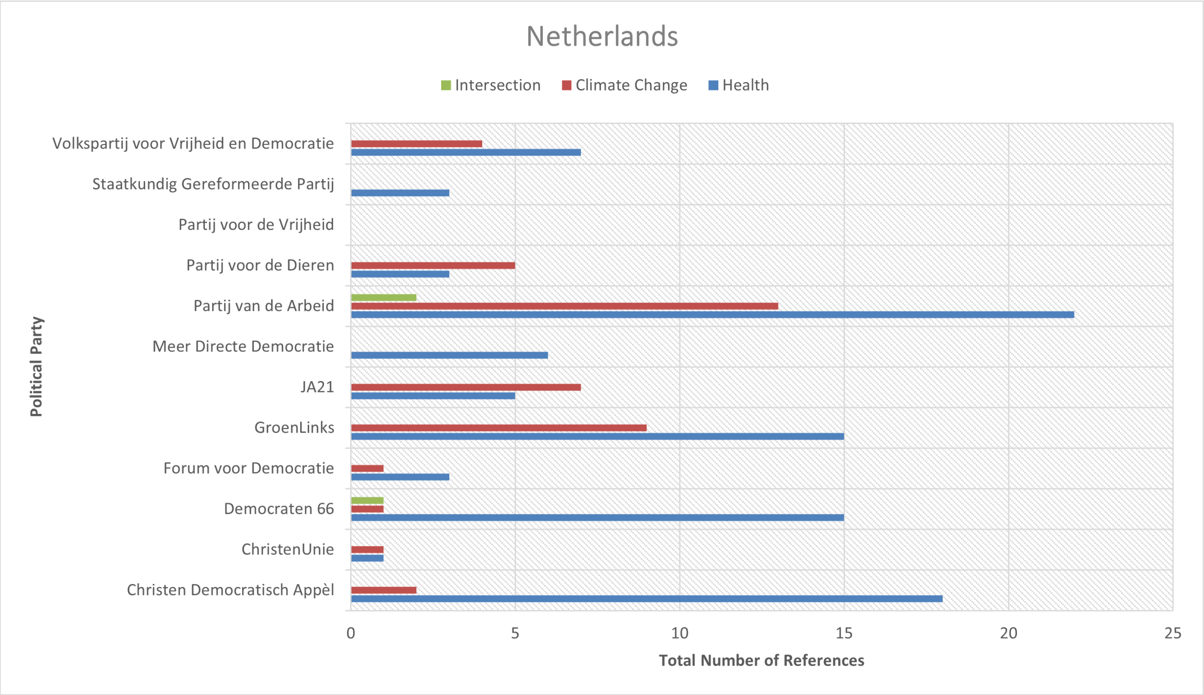

Figure 5.31 Total number of references to health, climate change, and the intersection of health and climate change in Netherlands (Kingdom of the), 2014-2022.

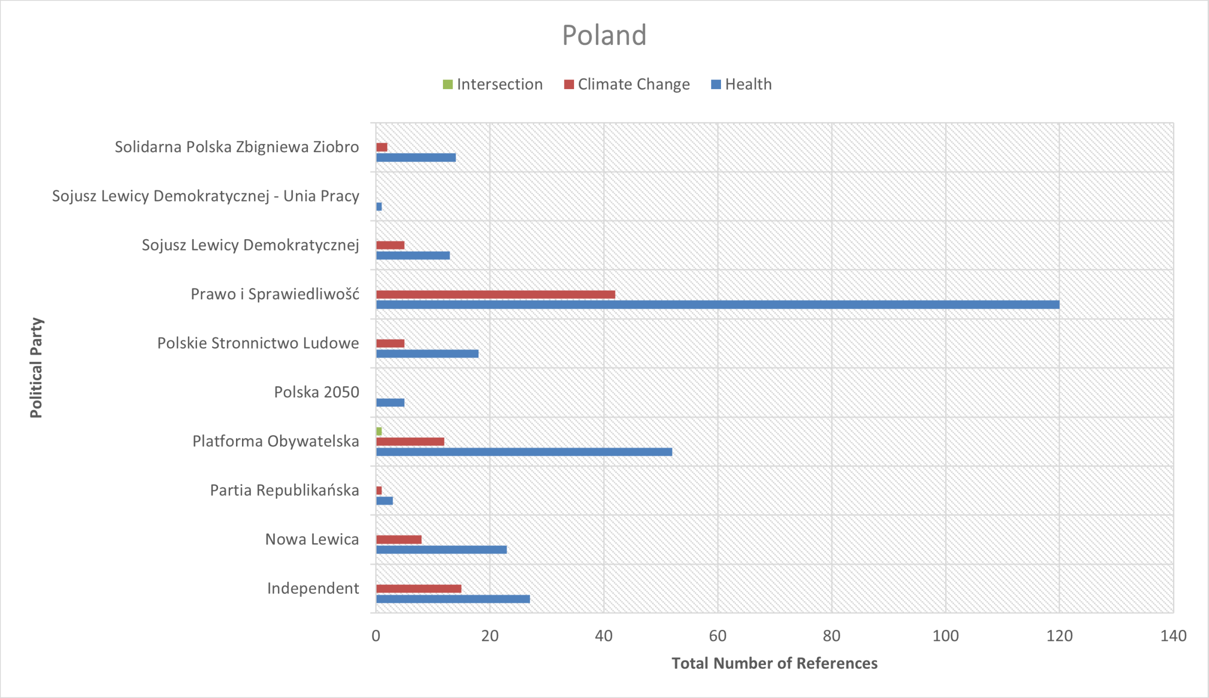

Figure 5.32 Total number of references to health, climate change, and the intersection of health and climate change in Poland, 2014-2022.

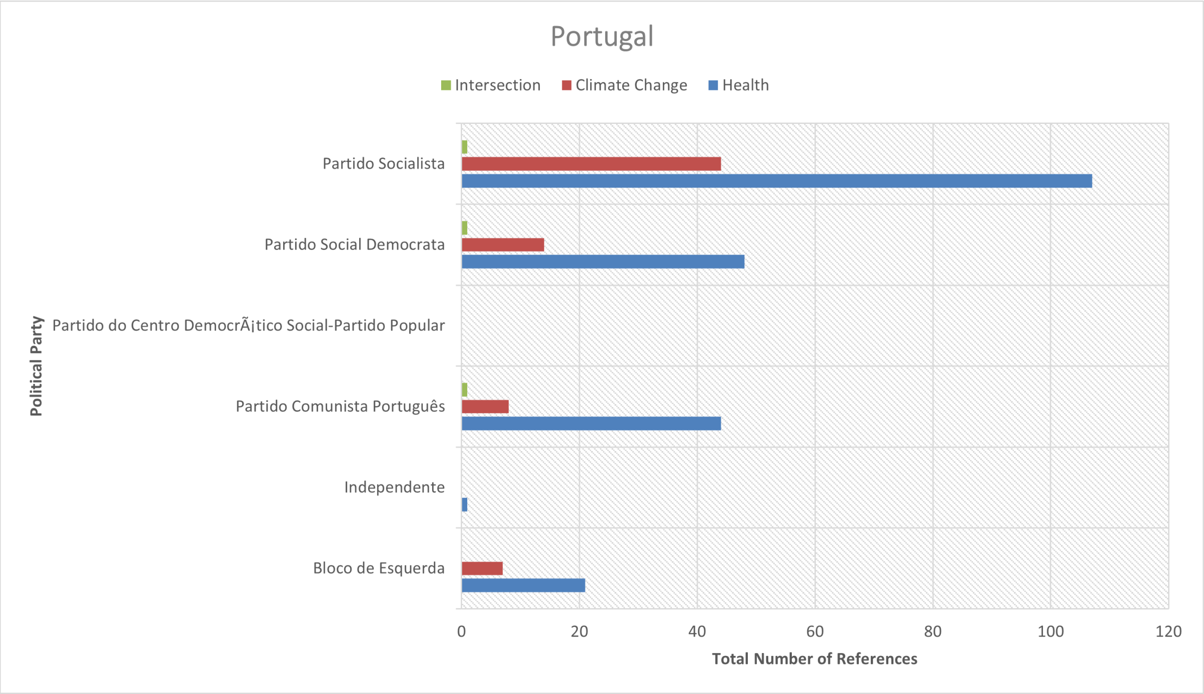

Figure 5.33 Total number of references to health, climate change, and the intersection of health and climate change in Portugal, 2014-2022.

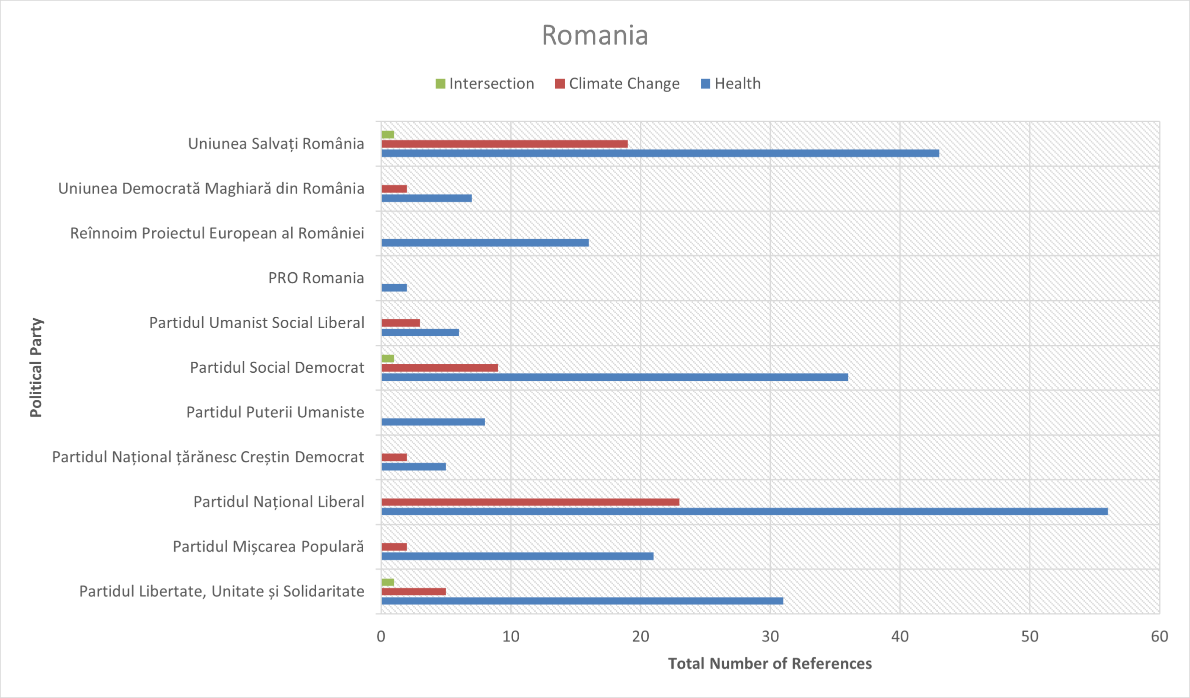

Figure 5.34 Total number of references to health, climate change, and the intersection of health and climate change in Romania, 2014-2022.

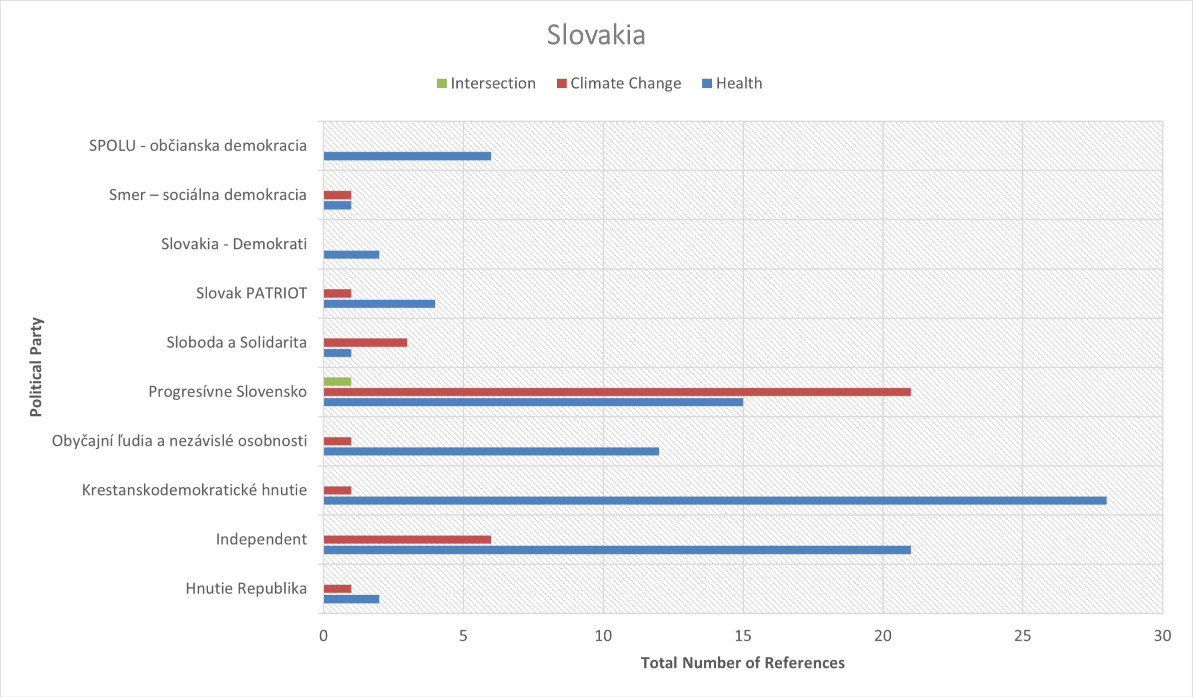

Figure 5.35 Total number of references to health, climate change, and the intersection of health and climate change in Slovakia 2014-2022.

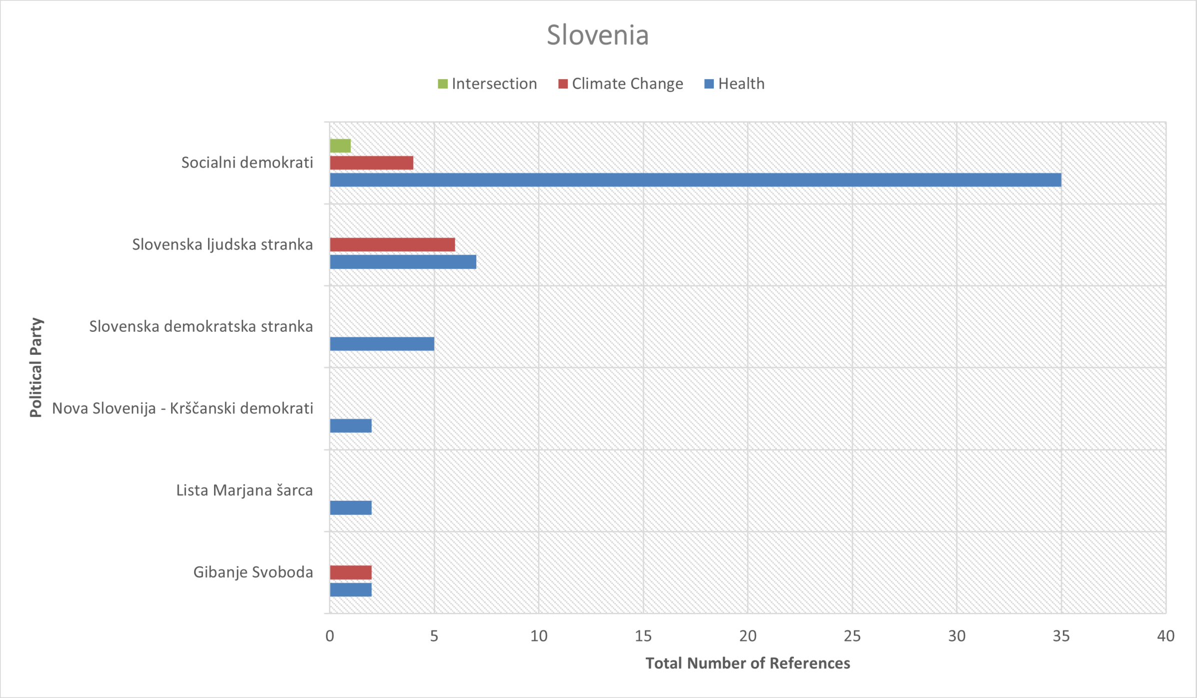

Figure 5.36 Total number of references to health, climate change, and the intersection of health and climate change in Slovenia, 2014-2022.

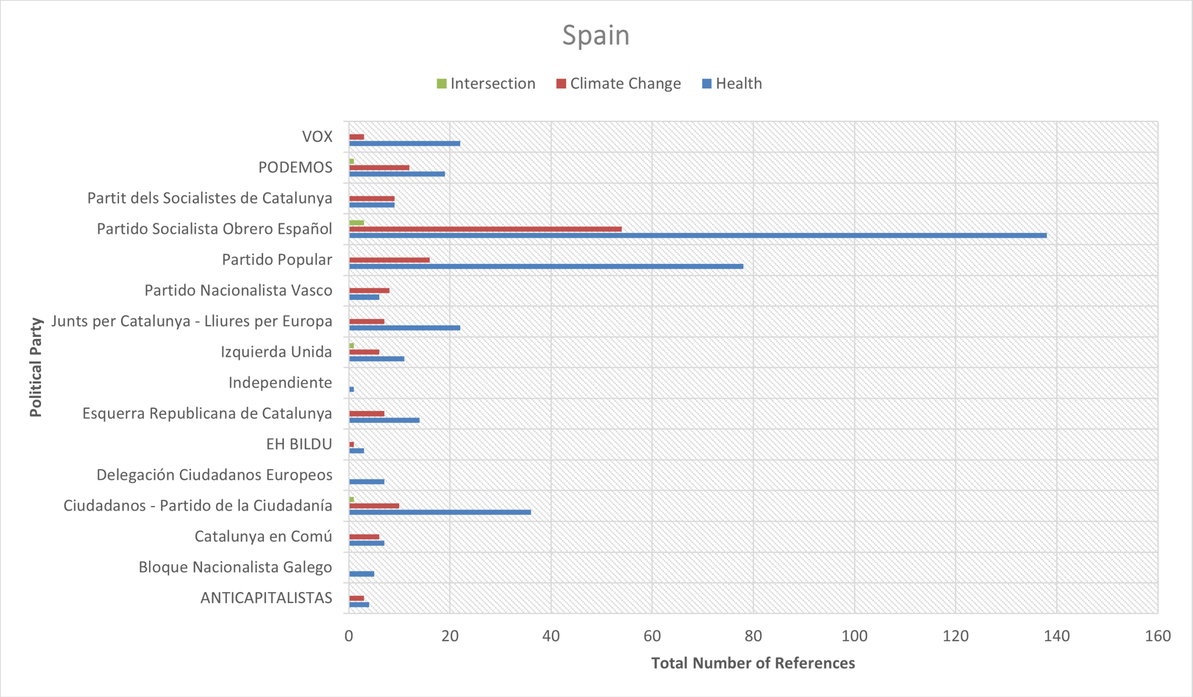

Figure 5.37 Total number of references to health, climate change, and the intersection of health and climate change in Spain, 2014-2022.

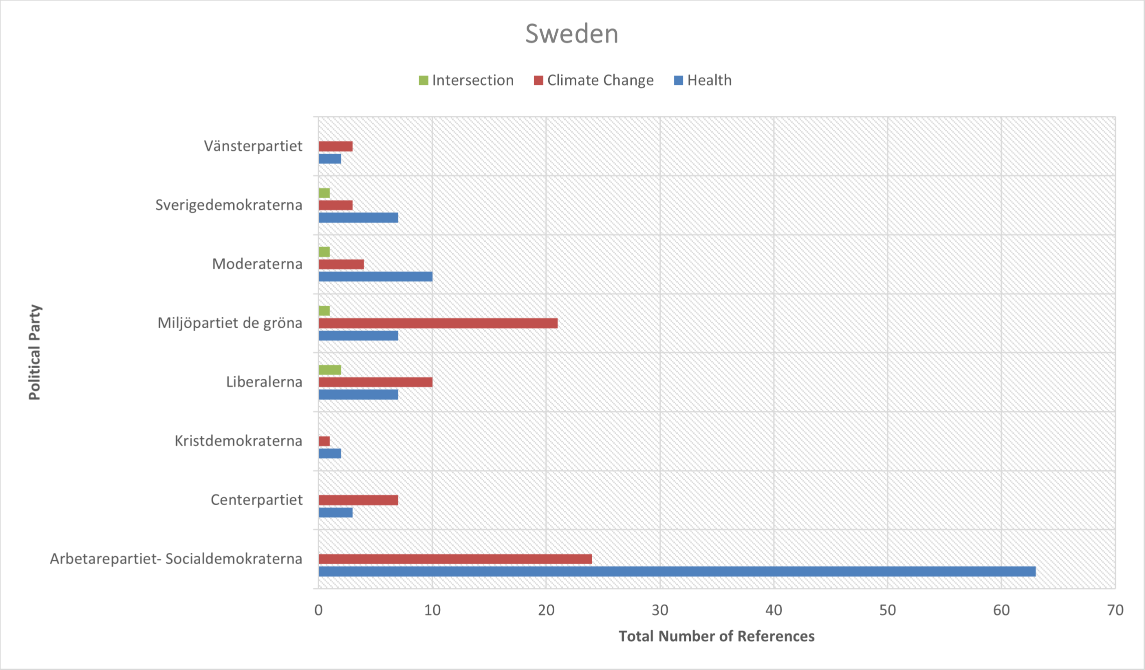

Figure 5.38 Total number of references to health, climate change, and the intersection of health and climate change in Sweden, 2014-2022.

Additional Figures

The figures in this section break down references to health, climate change, and the intersection of health and climate change between 2014 and 2022, by country. In these figures, the number of references are normalized by total number of speeches that year. Therefore, they show percentages instead of the total number of references. This can be misleading, especially in earlier years where a small number of speeches are recorded.

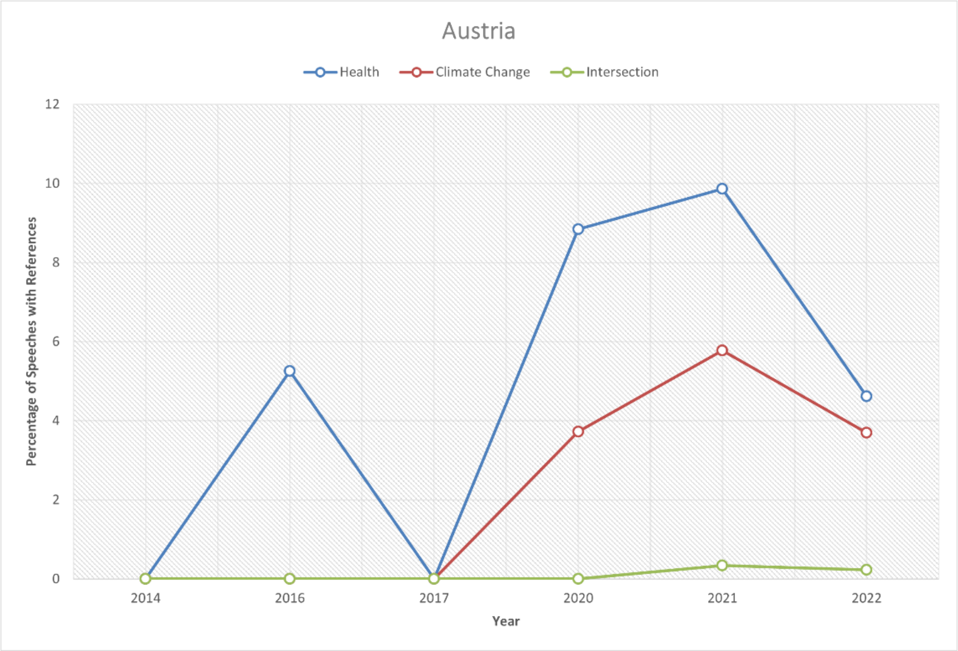

Figure 5.39 Percentage of speeches with references to health, climate change and the intersection of the terms for Austria.

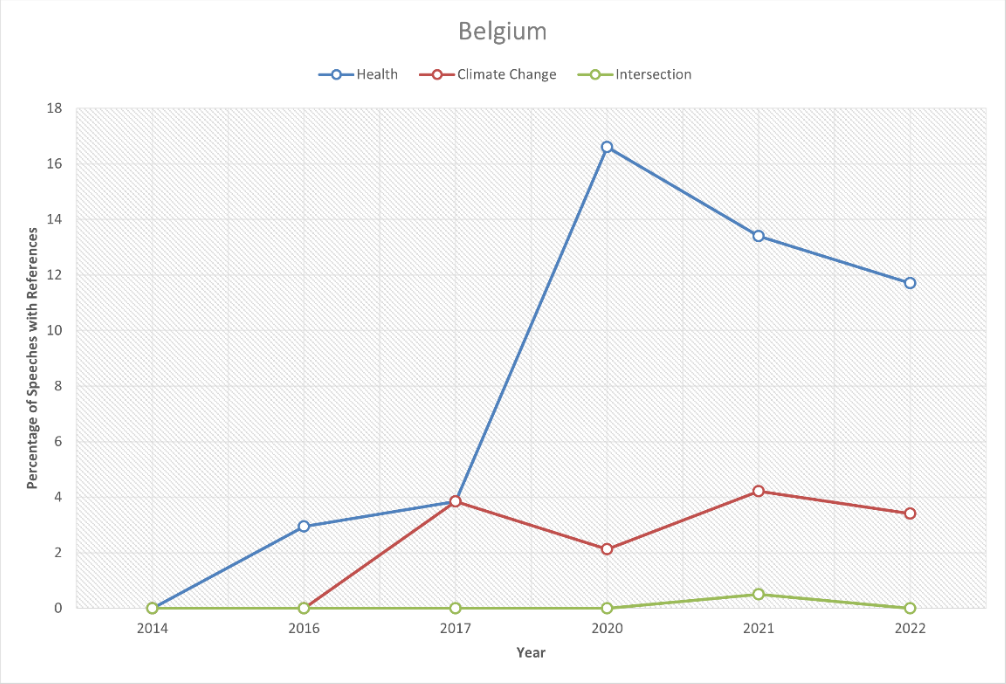

Figure 5.40 Percentage of speeches with references to health, climate change and the intersection of the terms for Belgium.

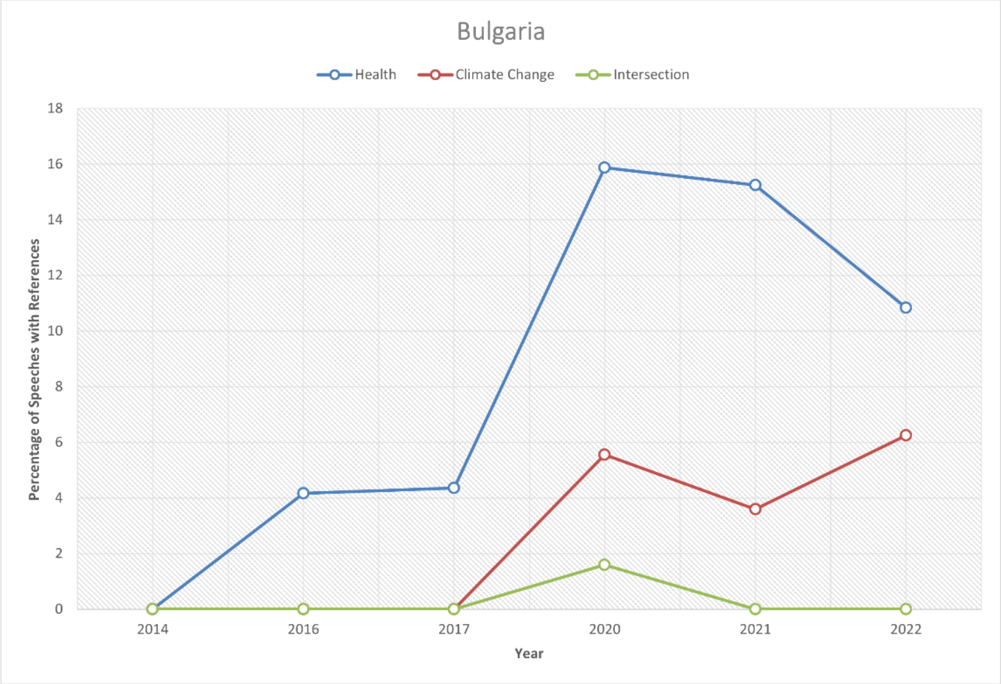

Figure 5.41 Percentage of speeches with references to health, climate change and the intersection of the terms for Bulgaria.

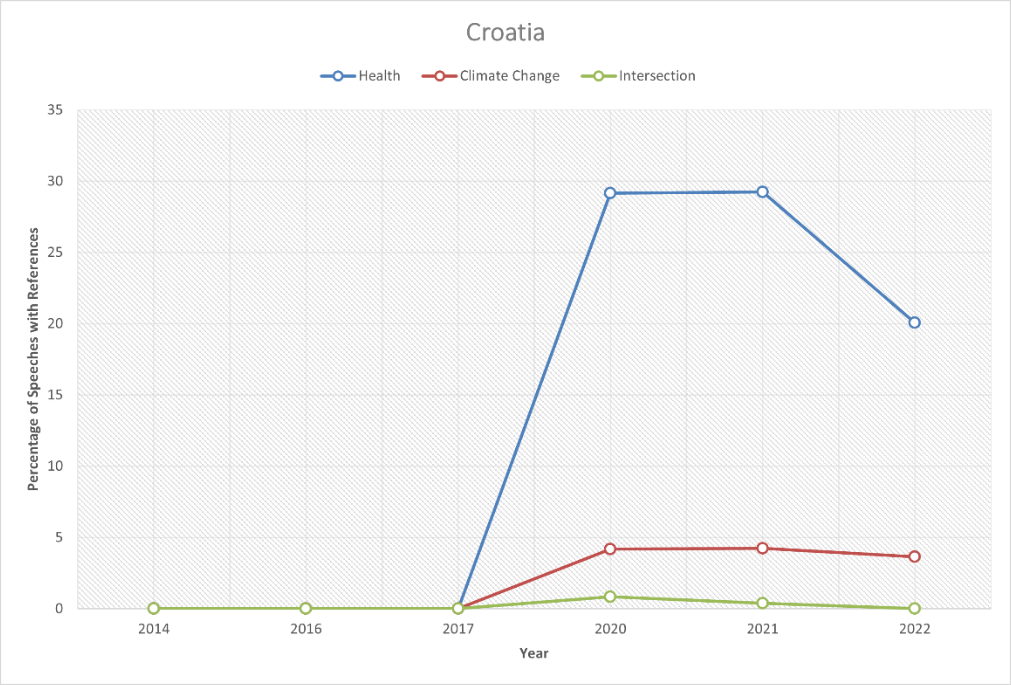

Figure 5.42 Percentage of speeches with references to health, climate change and the intersection of the terms for Croatia.

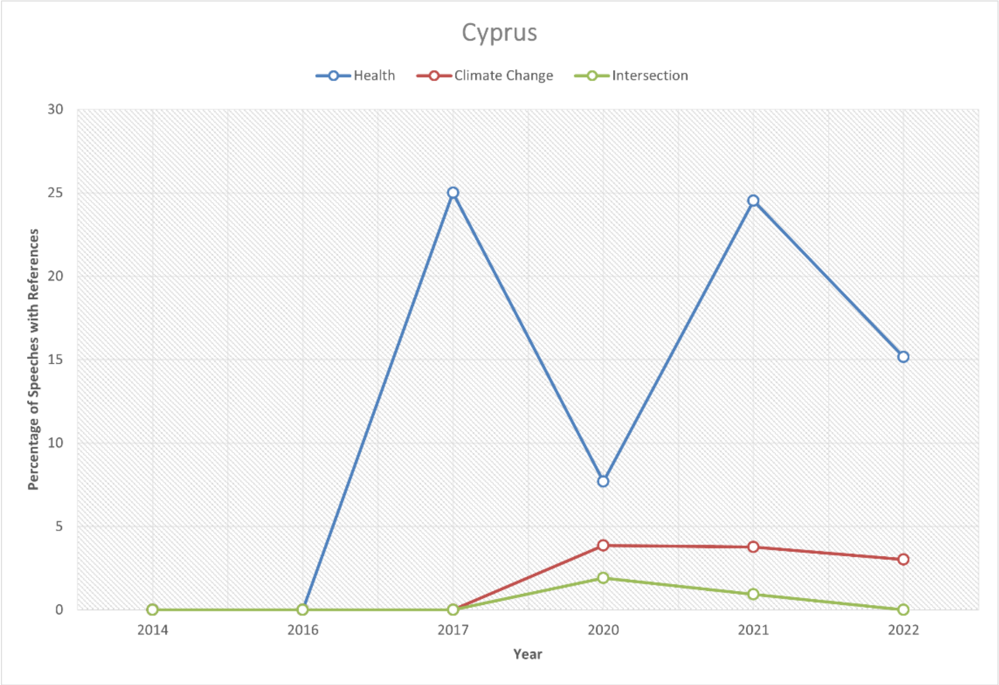

Figure 5.43 Percentage of speeches with references to health, climate change and the intersection of the terms for Cyprus.

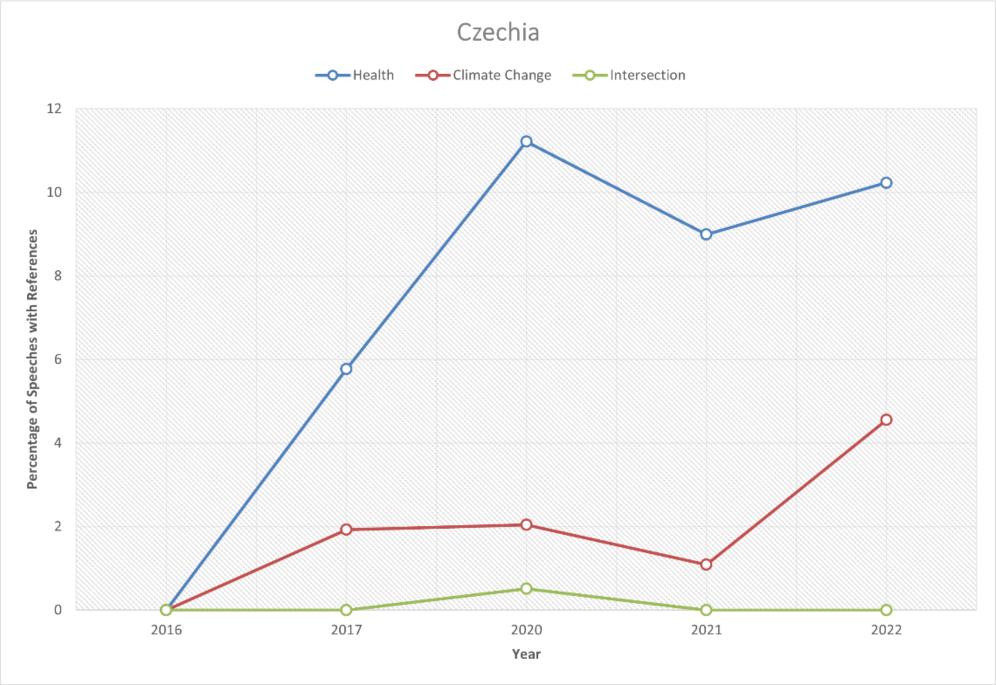

Figure 5.44 Percentage of speeches with references to health, climate change and the intersection of the terms for Czechia.

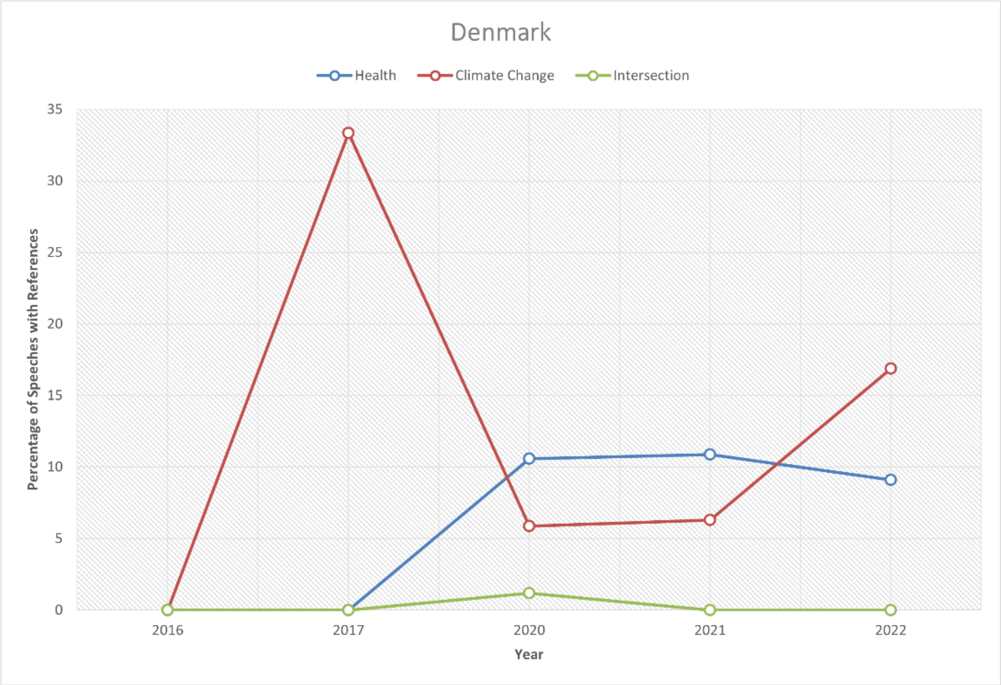

Figure 5.45 Percentage of speeches with references to health, climate change and the intersection of the terms for Denmark.

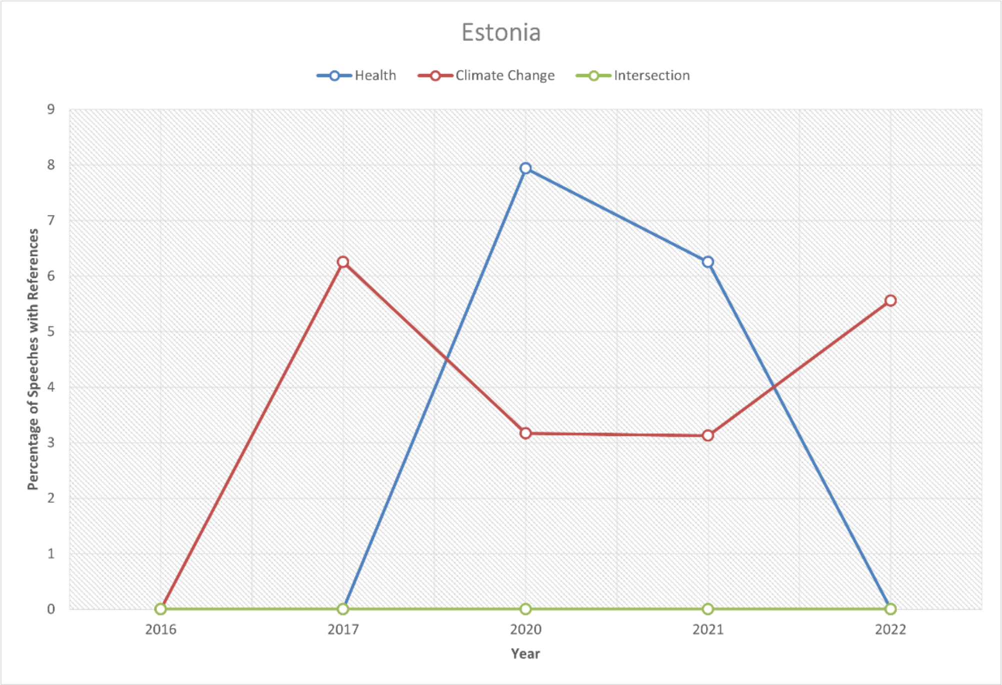

Figure 5.46 Percentage of speeches with references to health, climate change and the intersection of the terms for Estonia.

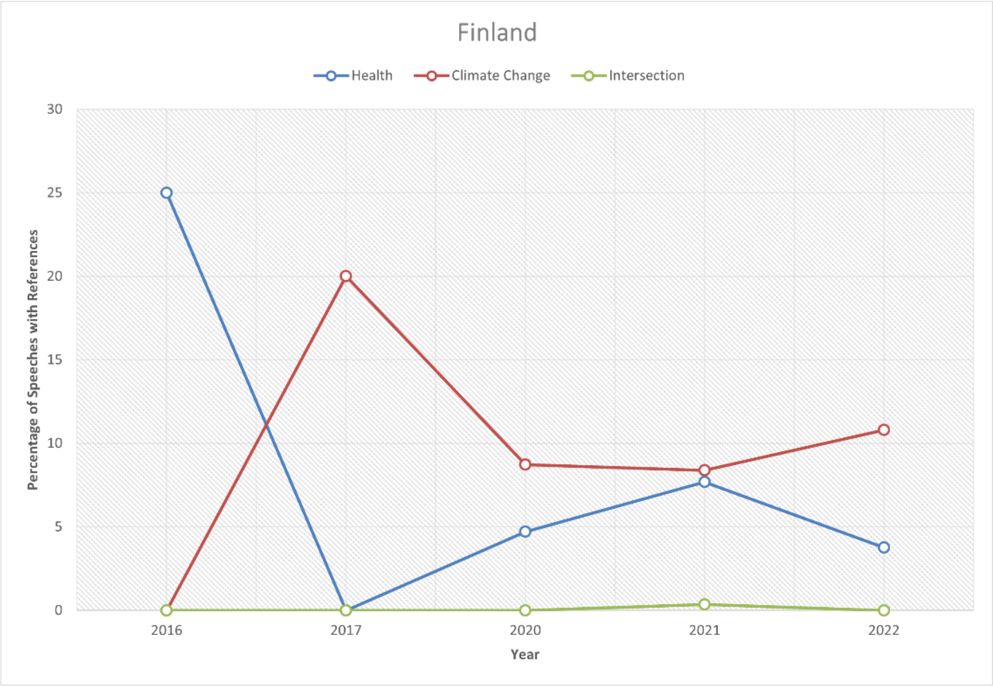

Figure 5.47 Percentage of speeches with references to health, climate change and the intersection of the terms for Finland.

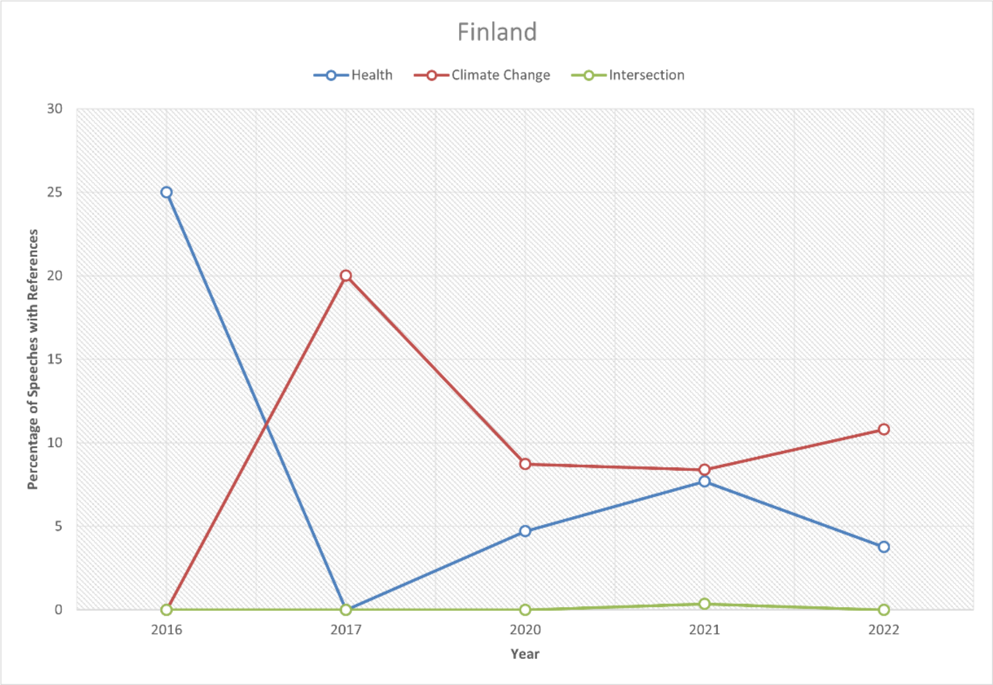

Figure 5.48 Percentage of speeches with references to health, climate change and the intersection of the terms for Finland.

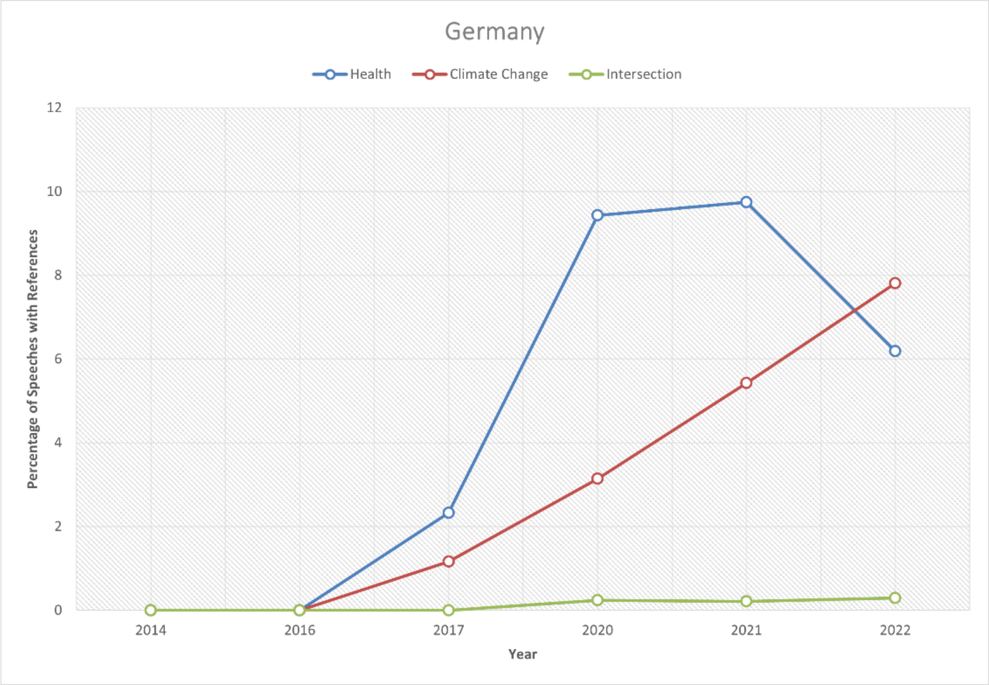

Figure 5.49 Percentage of speeches with references to health, climate change and the intersection of the terms for Germany.

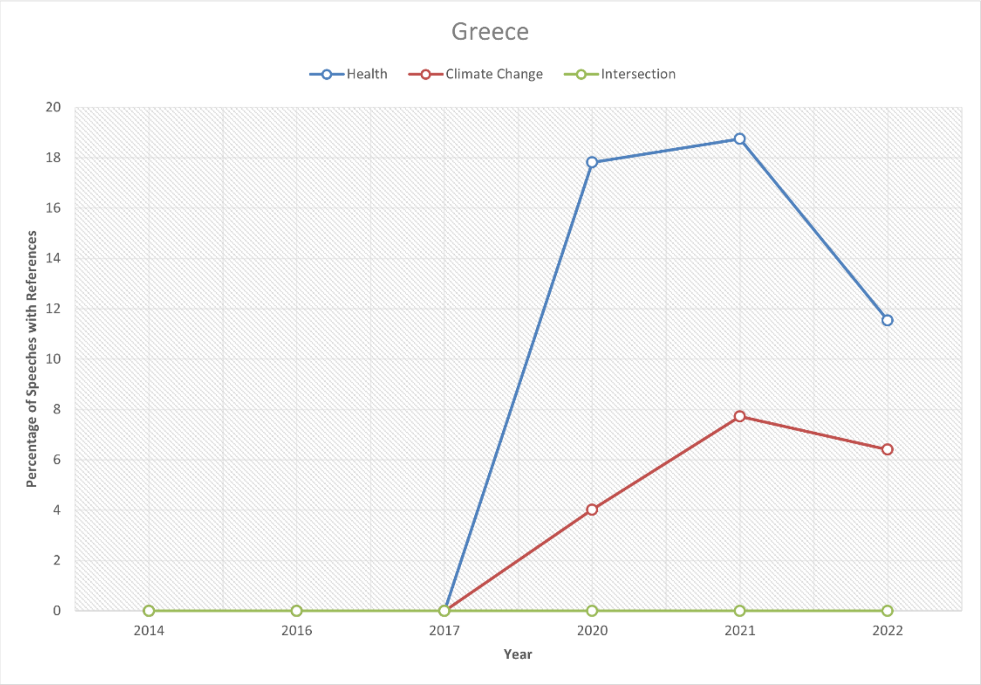

Figure 5.50 Percentage of speeches with references to health, climate change and the intersection of the terms for Greece.

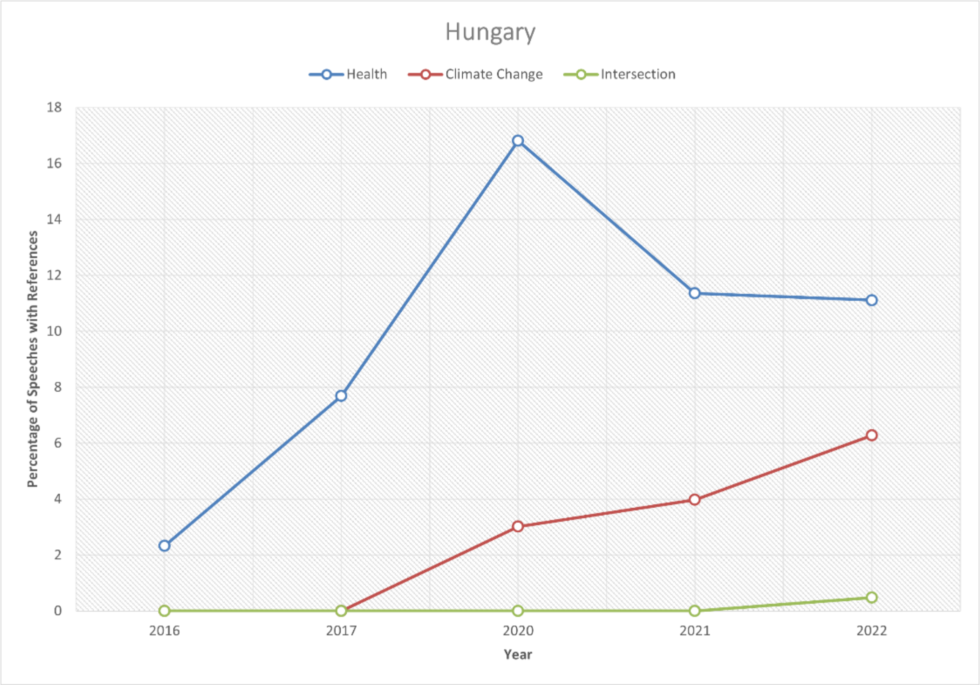

Figure 5.51 Percentage of speeches with references to health, climate change and the intersection of the terms for Hungary.

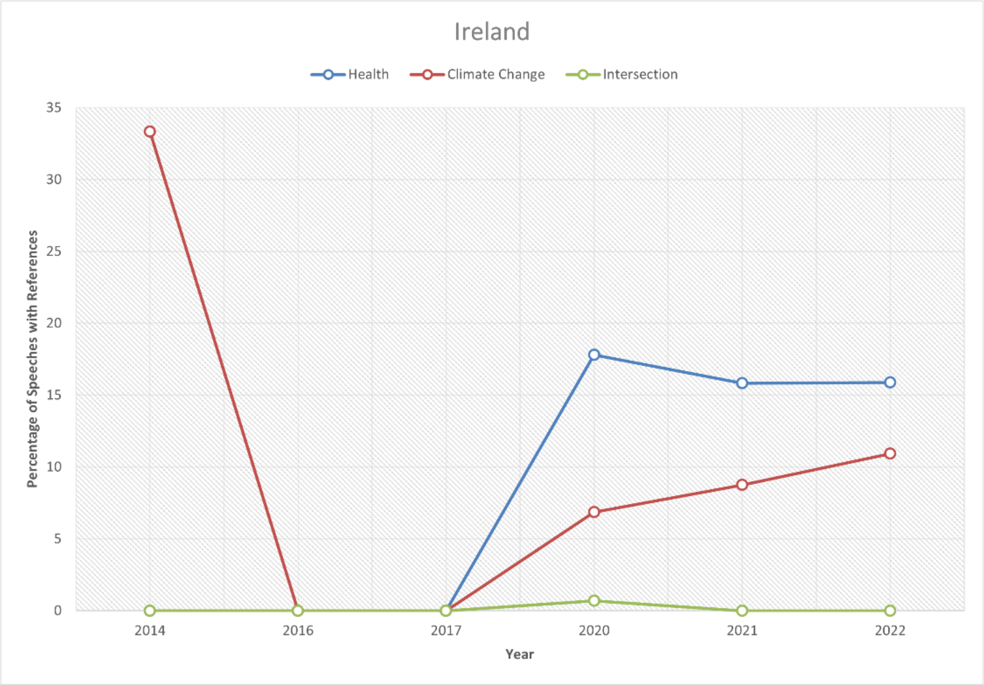

Figure 5.52 Percentage of speeches with references to health, climate change and the intersection of the terms for Ireland.

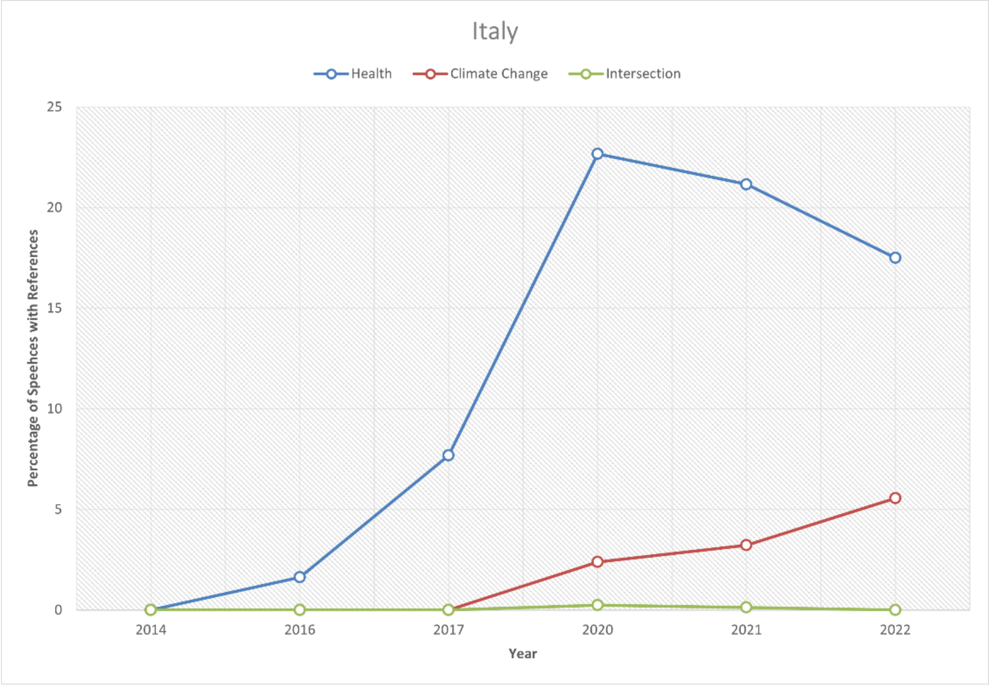

Figure 5.53 Percentage of speeches with references to health, climate change and the intersection of the terms for Italy.

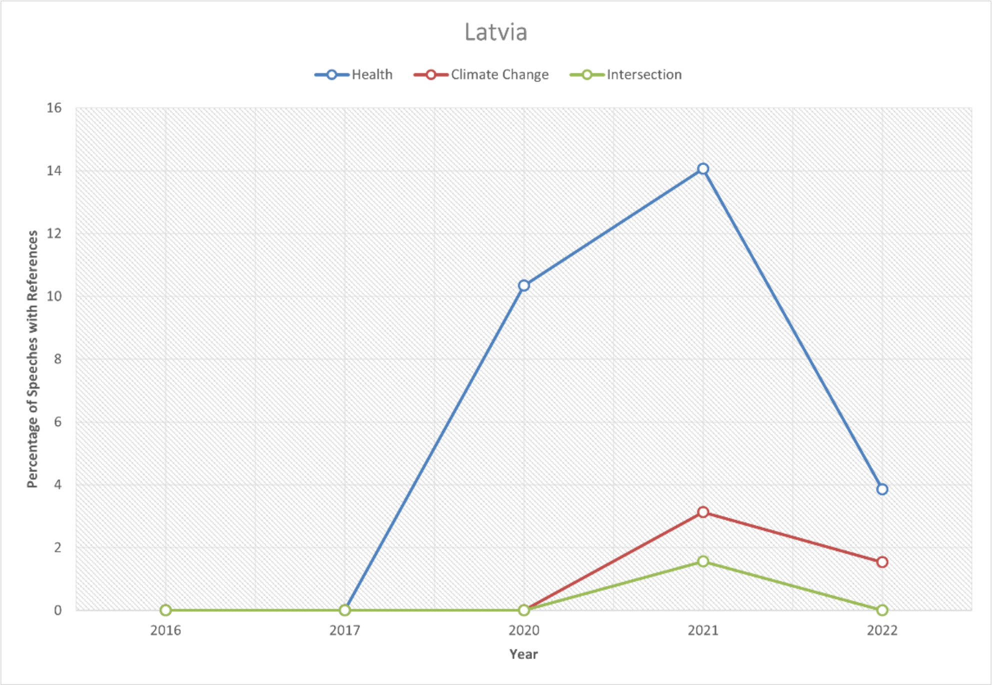

Figure 5.54 Percentage of speeches with references to health, climate change and the intersection of the terms for Latvia.

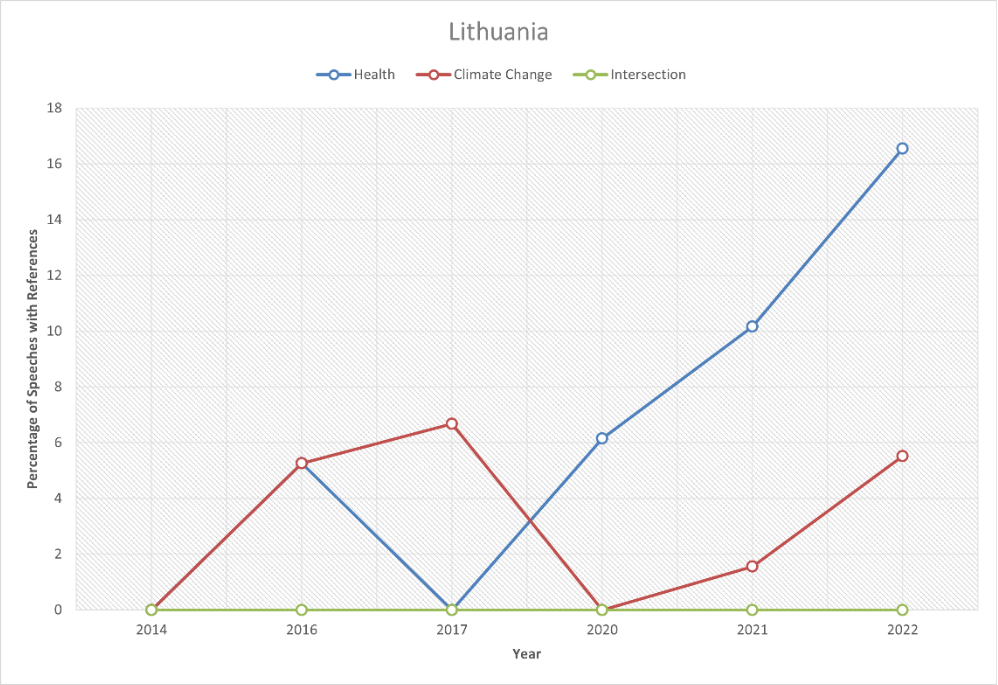

Figure 5.55 Percentage of speeches with references to health, climate change and the intersection of the terms for Lithuania.

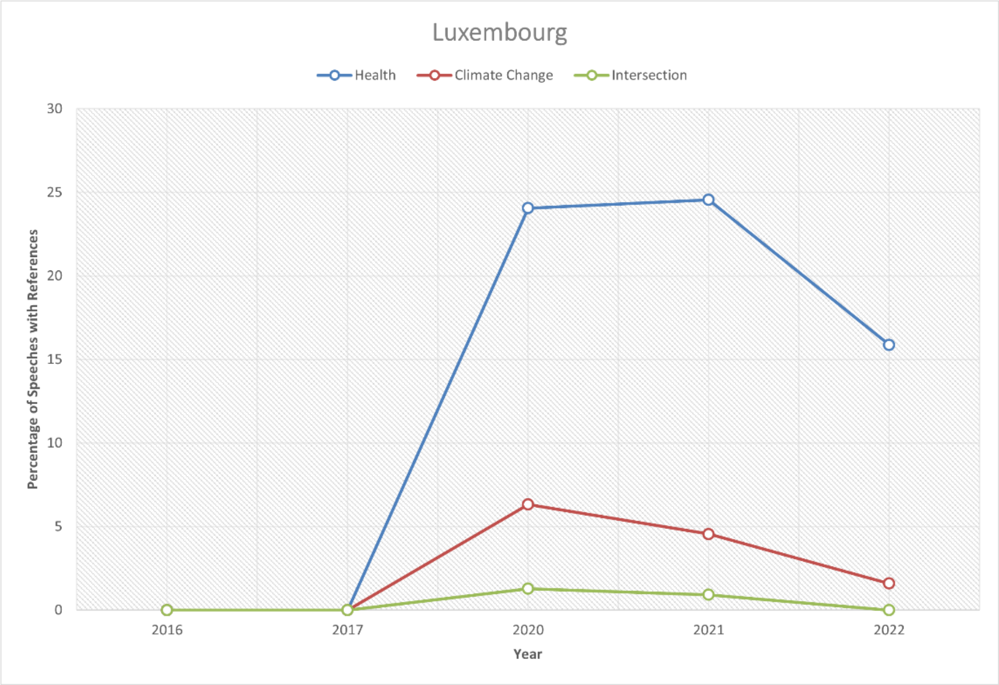

Figure 5.56 Percentage of speeches with references to health, climate change and the intersection of the terms for Luxembourg.

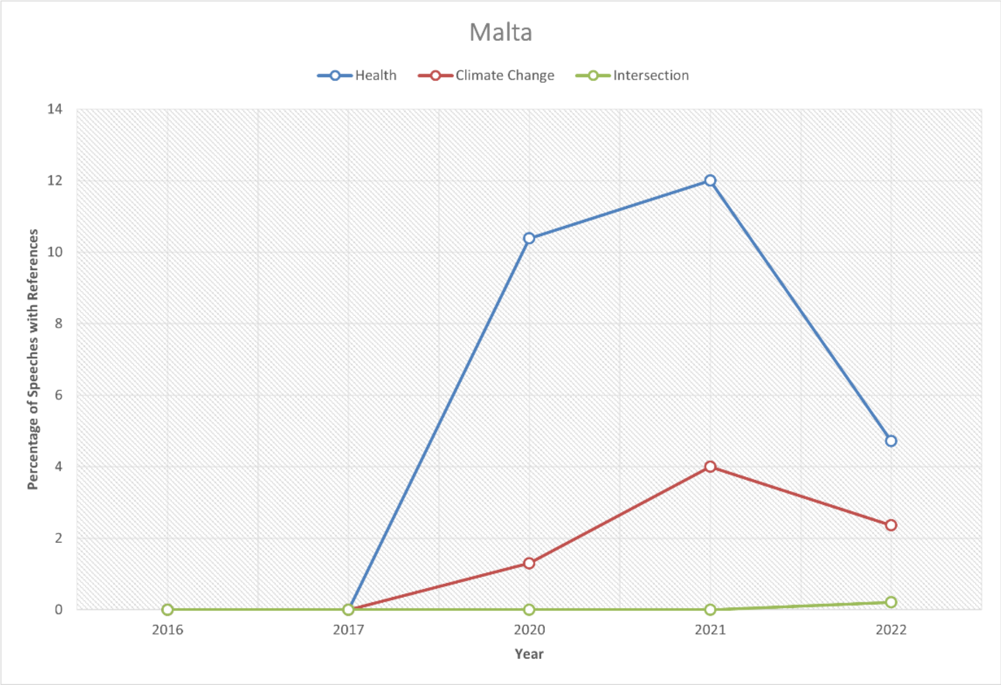

Figure 5.57 Percentage of speeches with references to health, climate change and the intersection of the terms for Malta.

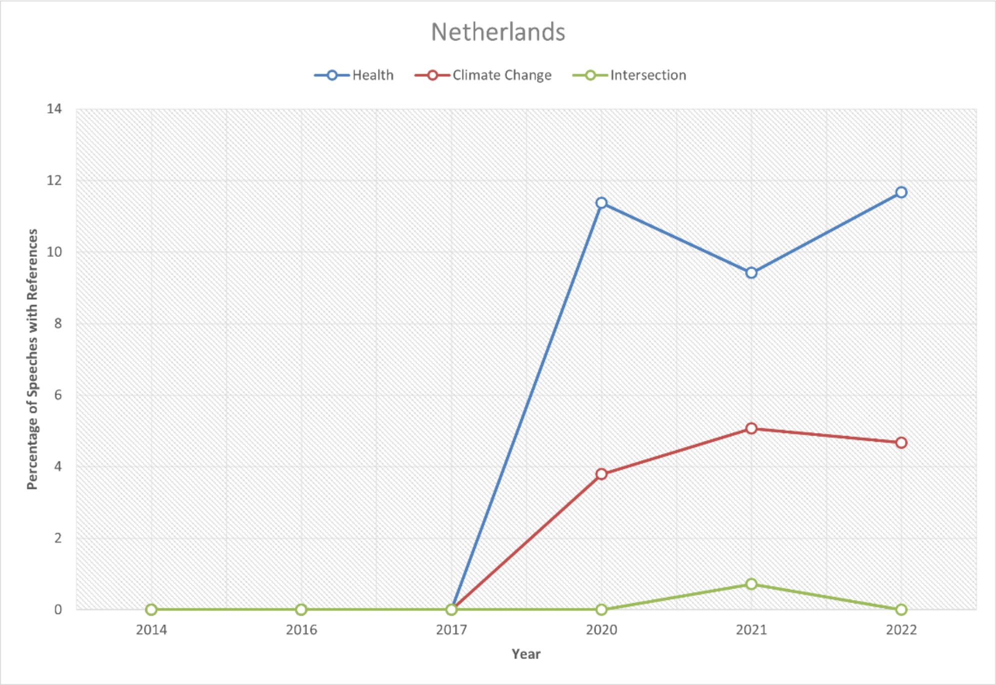

Figure 5.58 Percentage of speeches with references to health, climate change and the intersection of the terms for the Netherlands (Kingdom of the).

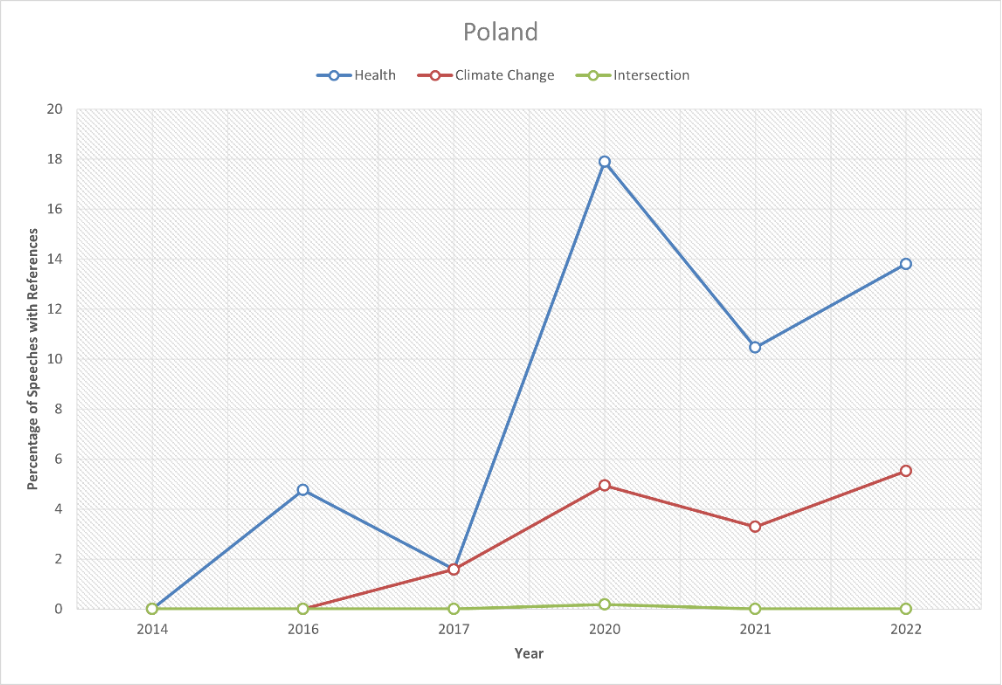

Figure 5.59 Percentage of speeches with references to health, climate change and the intersection of the terms for Poland.

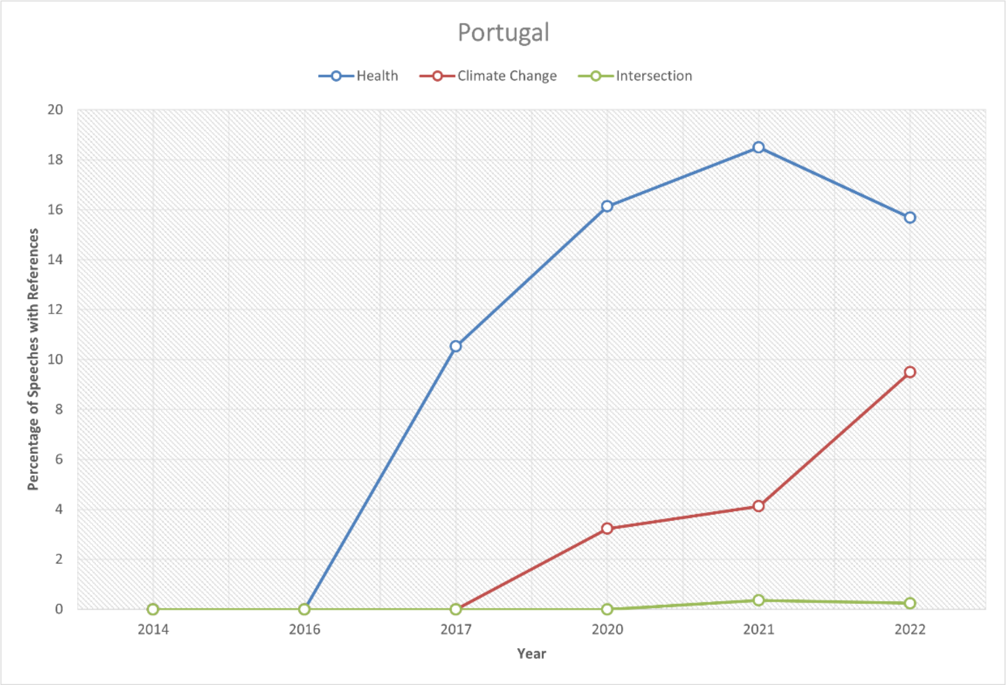

Figure 5.60 Percentage of speeches with references to health, climate change and the intersection of the terms for Portugal.

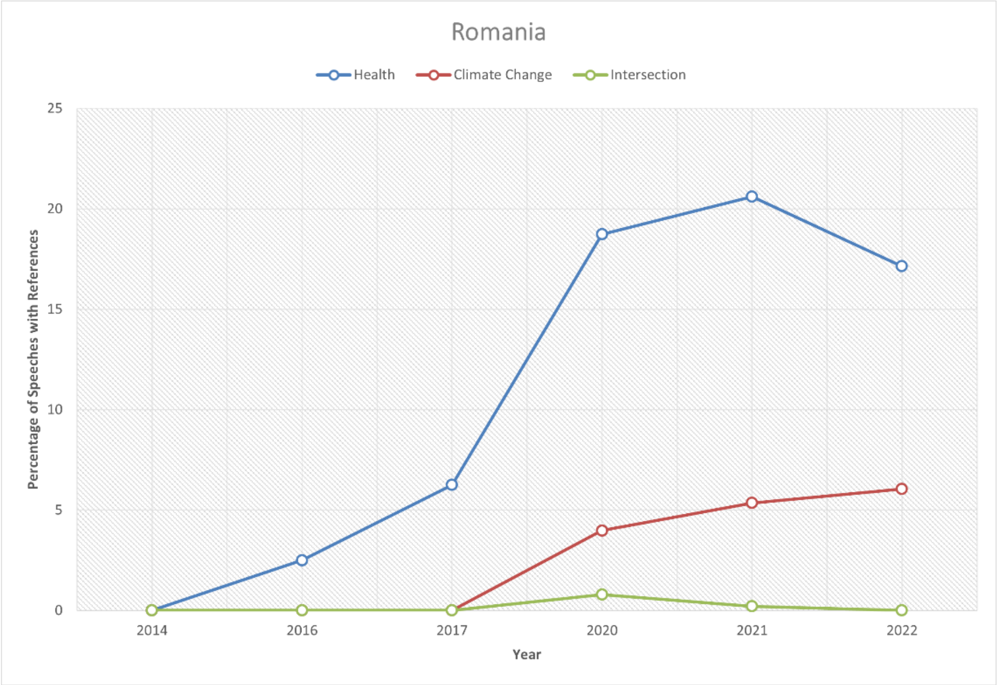

Figure 5.61 Percentage of speeches with references to health, climate change and the intersection of the terms for Romania.

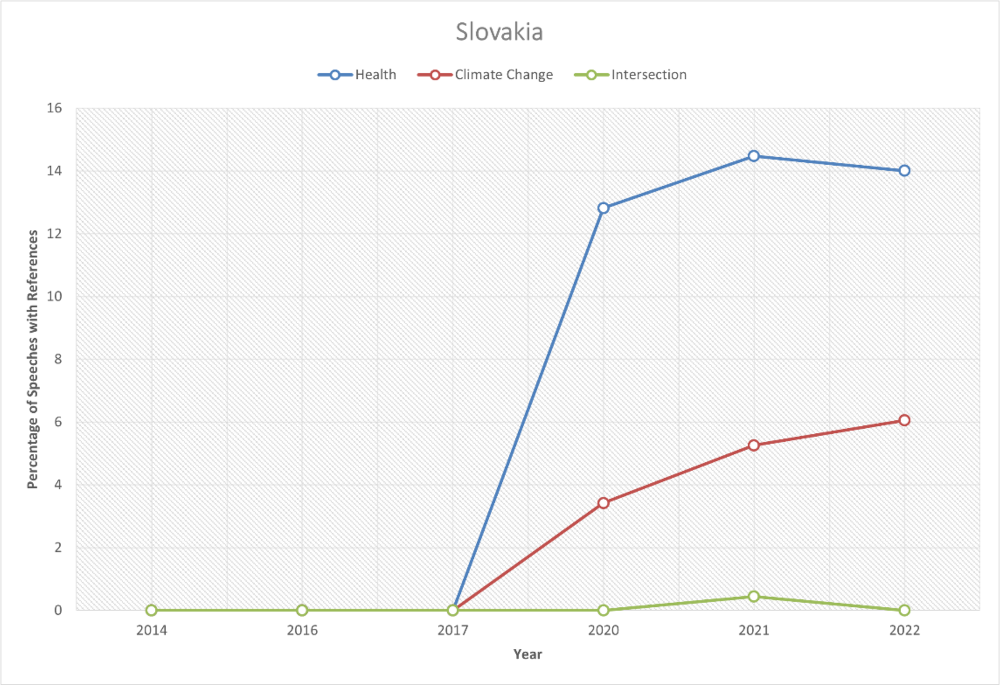

Figure 5.62 Percentage of speeches with references to health, climate change and the intersection of the terms for Slovakia.

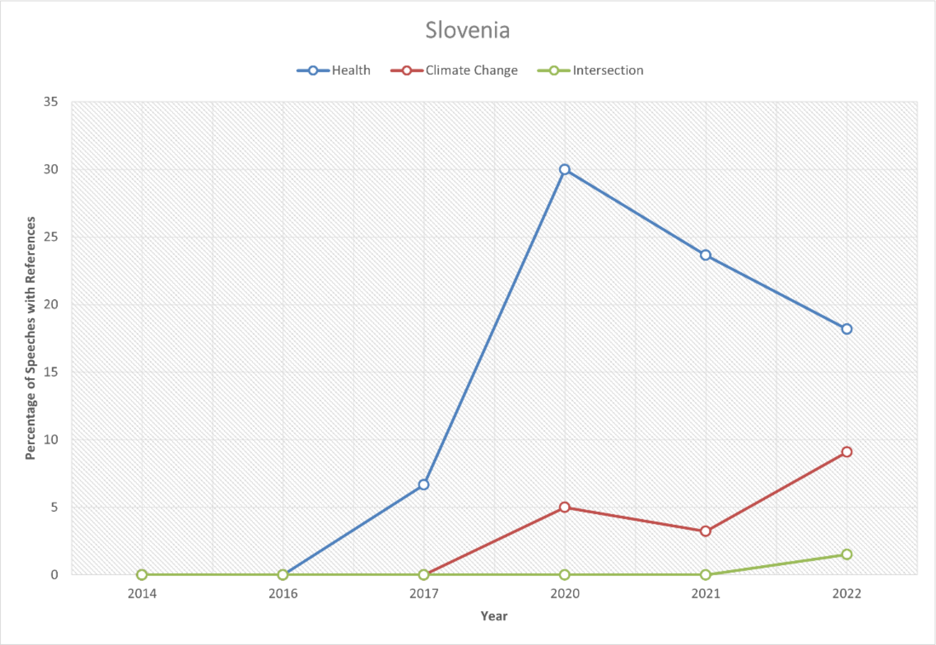

Figure 5.63 Percentage of speeches with references to health, climate change and the intersection of the terms for Slovenia.

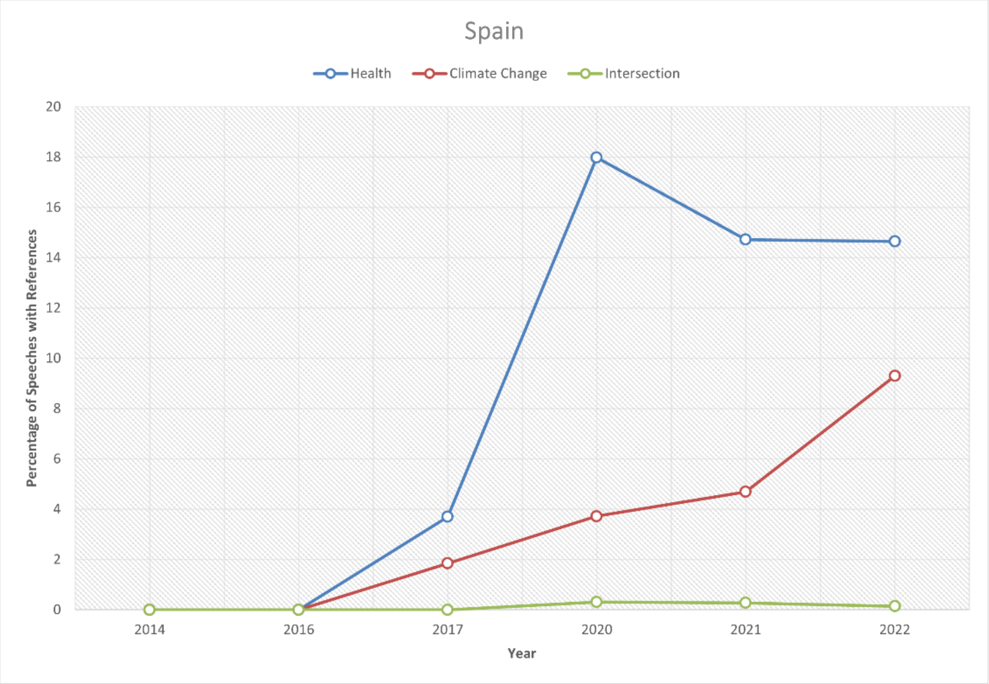

Figure 5.64 Percentage of speeches with references to health, climate change and the intersection of the terms for Spain.

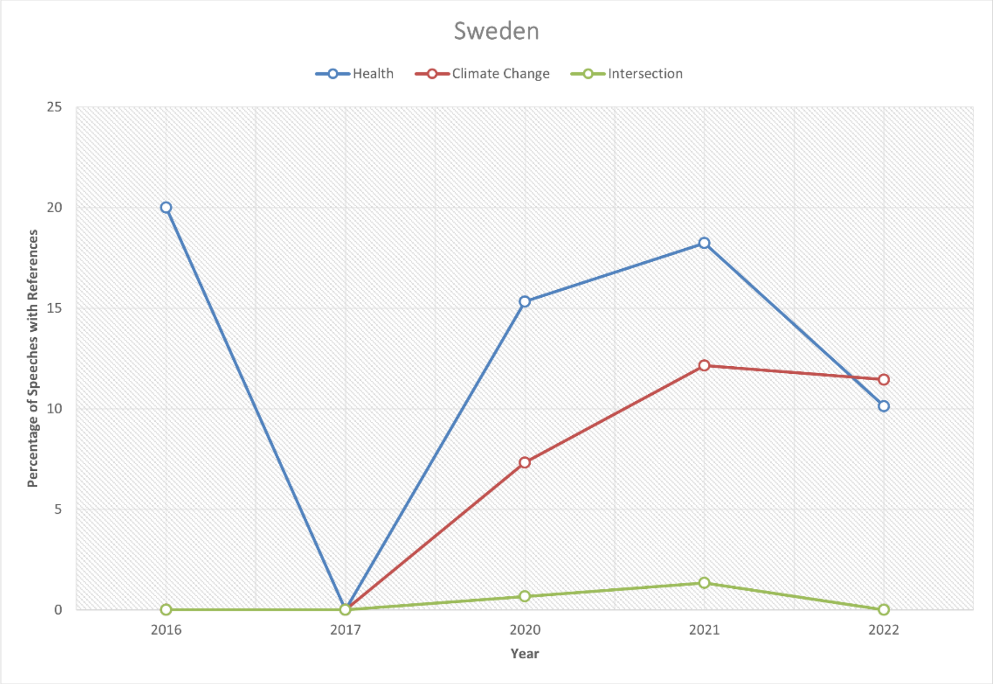

Figure 5.65 Percentage of speeches with references to health, climate change and the intersection of the terms for Sweden.

Indicator 5.3.2: Political engagement with health and climate change on social media

Geographic Coverage of Europe

This indicator includes all 38 European Environment Agency countries.

Data

The data collection process involved leveraging the open-source Python Library Tweepy, which utilizes the Twitter API V2, to gather relevant information from Twitter. Specifically, this research project utilized the "tweets/search/all" endpoint of the Twitter API, which was accessible through academic research access. The focus was on retrieving tweets from a carefully curated list of official accounts belonging to state leaders and key ministries, including Finance, Economy, Environment, and Health. In exceptional instances where an official handle was unavailable, tweets from personal accounts of relevant politicians overseeing the respective ministries were also included in the dataset.

Due to the large volume of tweets, we decided against translating them all as we did for the parliamentary speeches. Instead, we translated the terms provided in **Table 5.3**.

Methods

The second part of the indicator captures national governments' responses to the nexus of climate change and health. This part of the indicator measures governments' engagement with health and climate change through their official twitter handle. Our approach to produce the indicators is based on identifying the presence of key search terms related to health and climate change within each tweet and identifying instances of intersection where terms relating to each field appeared in the same tweet. The list of terms is the same as in **Table 5.3**.

Inequality Context

Additionally, we searched our samples for terms relating to inequality and injustice, and report on how these terms appear in the context of the health-climate tweets. We focus on the following terms: "inequality" "inequity" "injustice" "justice" "equity" "equality". We translated these terms into the relevant languages before searching the tweets that include the intersection of health and climate change. We find that in total, these terms only appear once in the same window as the intersection, in a tweet from Slovakia.

Additional analysis

We present some additional findings and figures in this section.

Figure 5.66 shows the number of references to health, climate change, and the intersection of health and climate change between 2018 and 2022. This data normalised by number of tweets for each year, and then multiplied by 100 to show the percentage of tweets that mention health, climate change, and the intersection of health and climate change. The figure shows a large spike in tweets over health in 2020, coinciding with the global pandemic. **Figure 5.67** shows the percentage of tweets by country that reference health, climate change, and the intersection of health and climate change between 2018 and 2022. The figure shows that the highest percentage of references to health by far comes from Germany, while the highest percentage of tweets that reference Climate Change come from Austria.

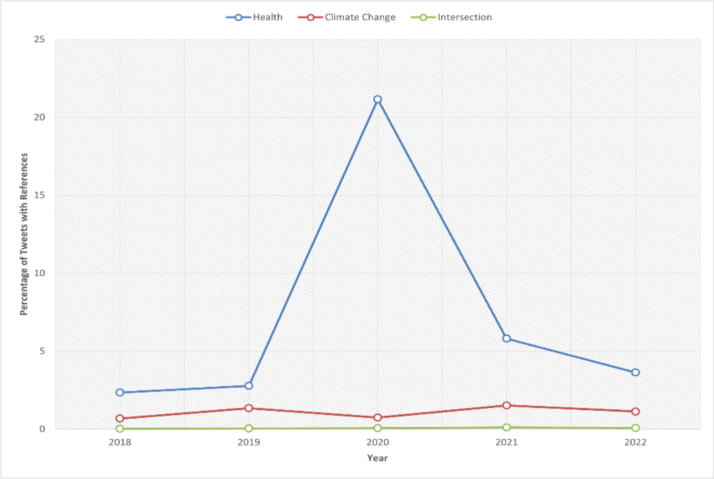

Figure 5.66 Total number of references to health, climate change, and the intersection of health and climate change over time, 2018-2022.

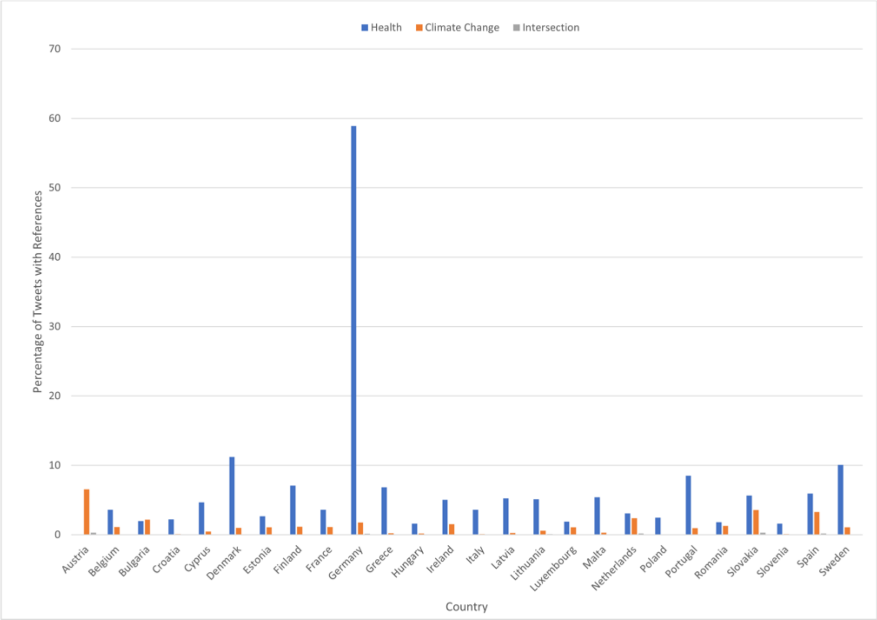

Figure 5.67 Total number of references to health, climate change, and the intersection of health and climate change by political group, 2014-2022.

Further Analysis on Health and Climate Change by Country

The figures in this section break down references to health, climate change, and the intersection of health and climate change between 2018 and 2022, by EEA country. In these figures, the number of references are normalized by total number of speeches that year. Therefore, they show percentages instead of the total number of references.

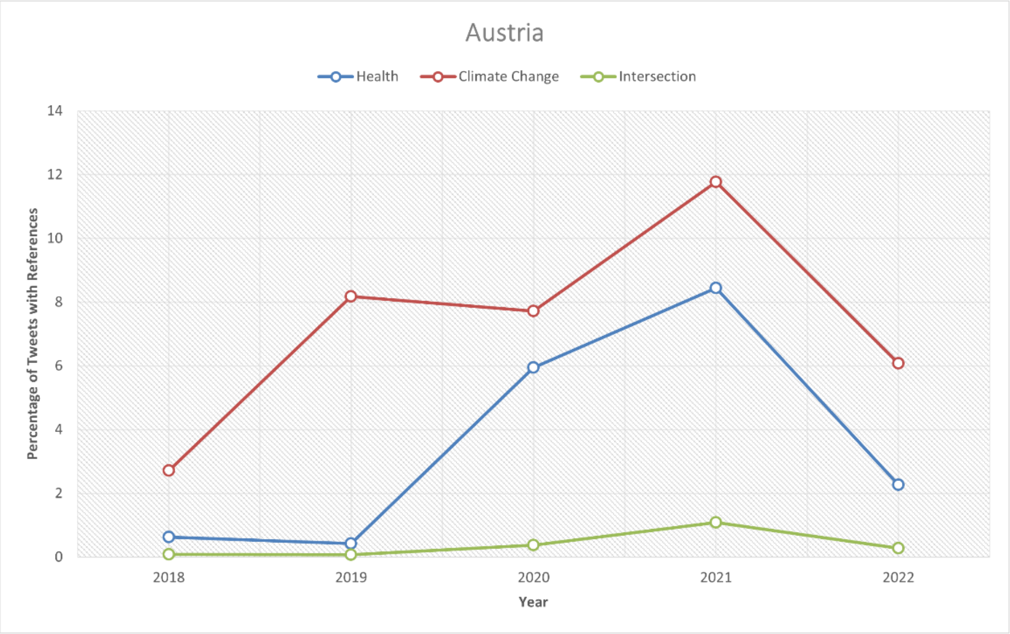

Figure 5.68 Percentage of tweets with references to health, climate change and the intersection of the terms for Austria.

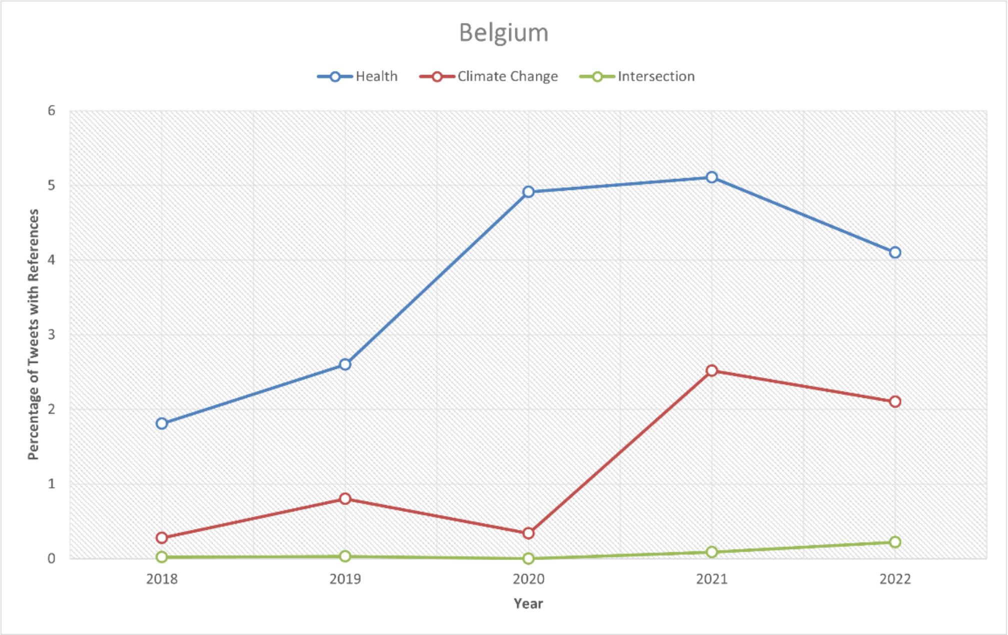

Figure 5.69 Percentage of Tweets with references to health, climate change and the intersection of the terms for Belgium.

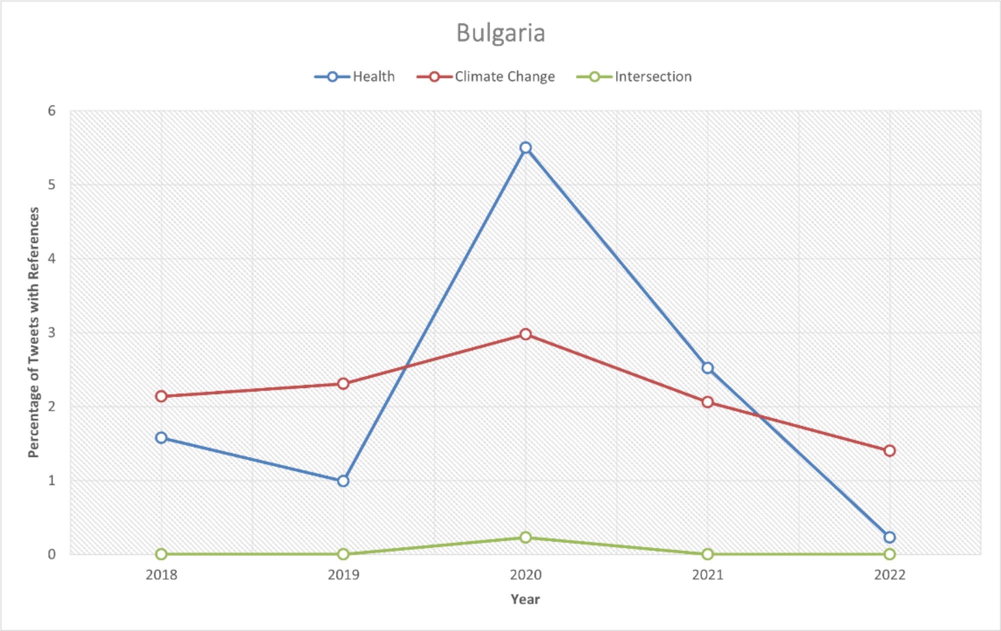

Figure 5.70 Percentage of Tweets with references to health, climate change and the intersection of the terms for Bulgaria.

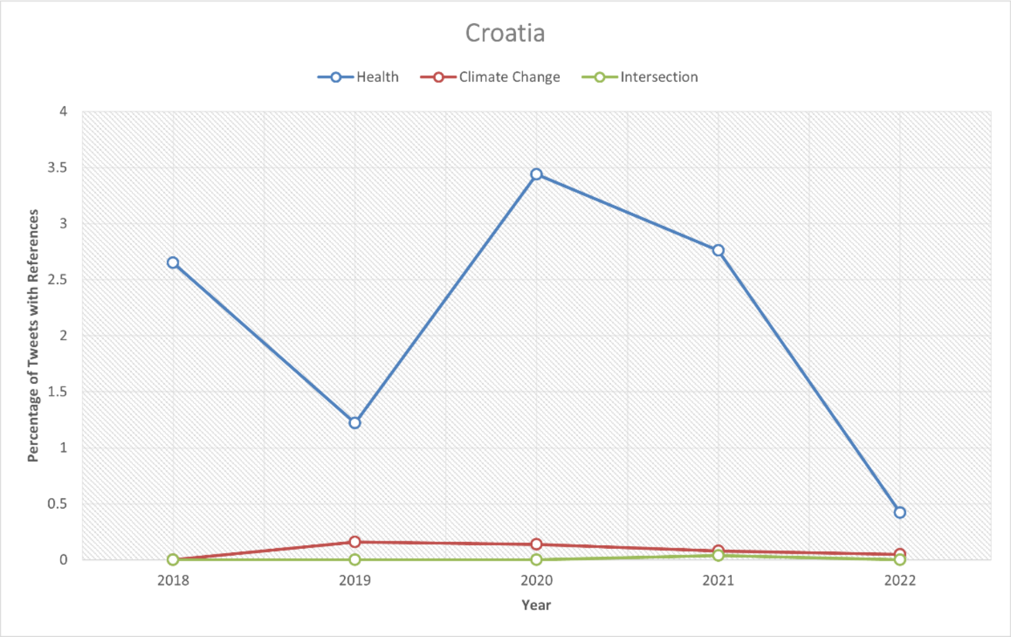

Figure 5.71 Percentage of Tweets with references to health, climate change and the intersection of the terms for Croatia.

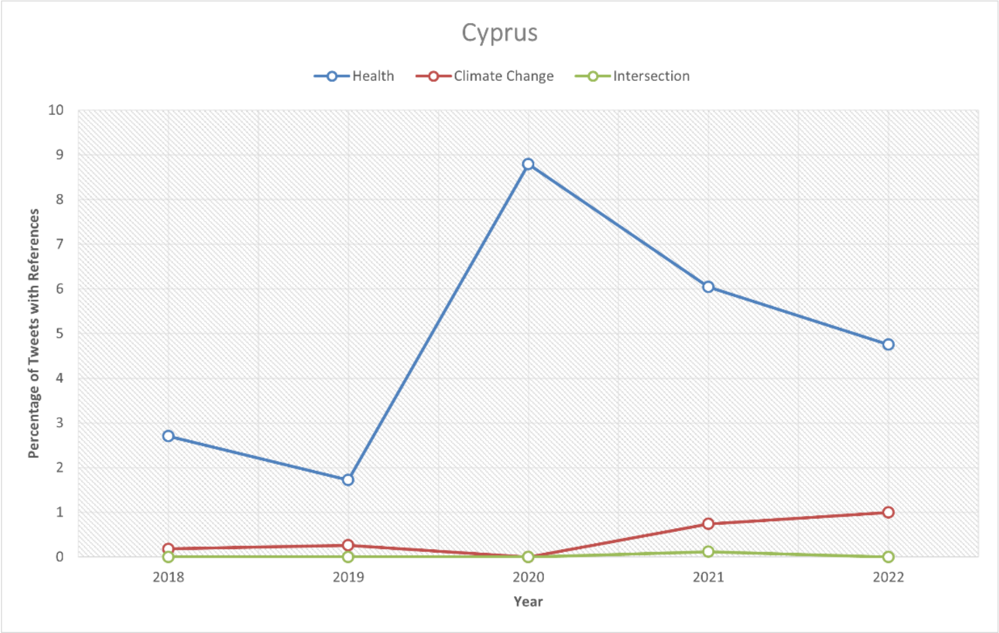

Figure 5.72 Percentage of Tweets with references to health, climate change and the intersection of the terms for Cyprus.

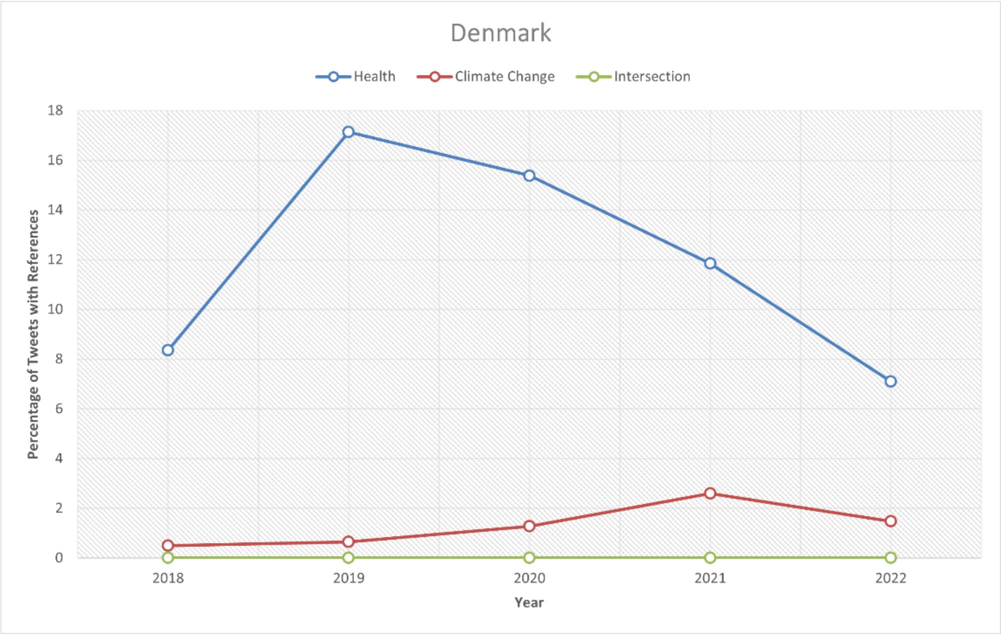

Figure 5.73 Percentage of Tweets with references to health, climate change and the intersection of the terms for Denmark.

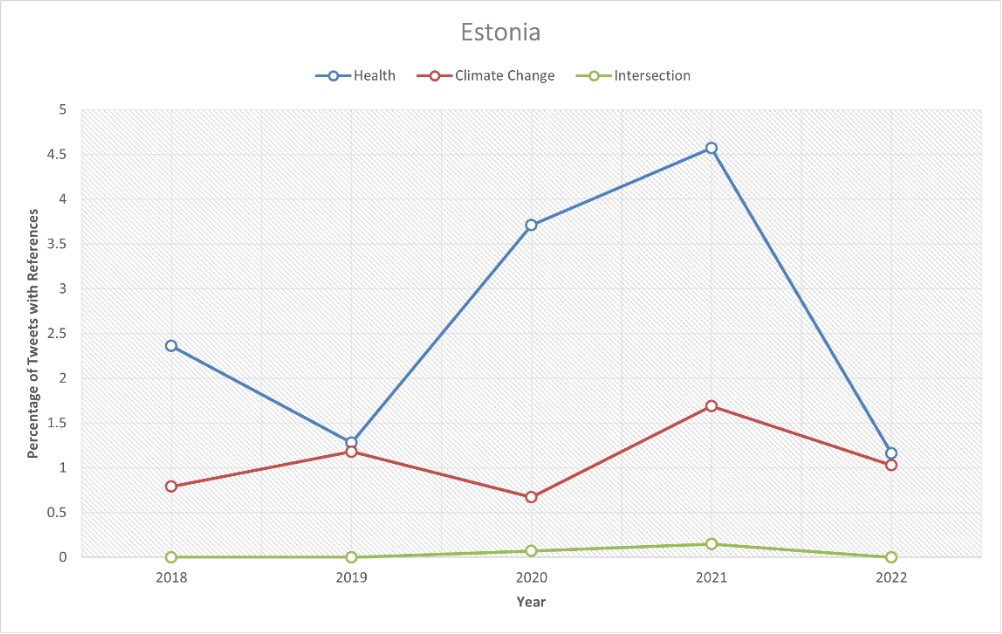

Figure 5.74 Percentage of Tweets with references to health, climate change and the intersection of the terms for Estonia.

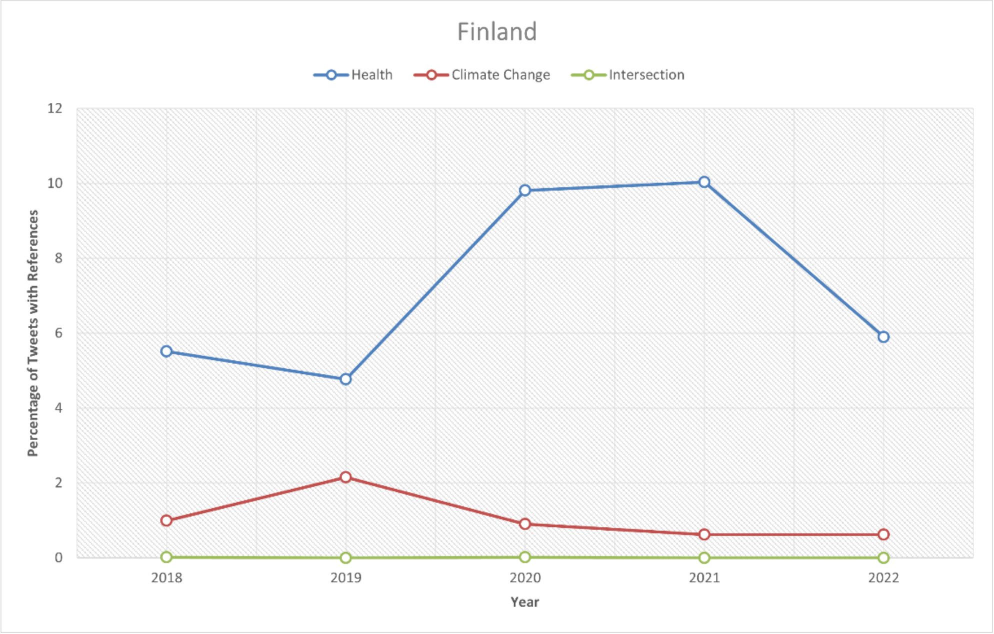

Figure 5.75 Percentage of Tweets with references to health, climate change and the intersection of the terms for Finland.

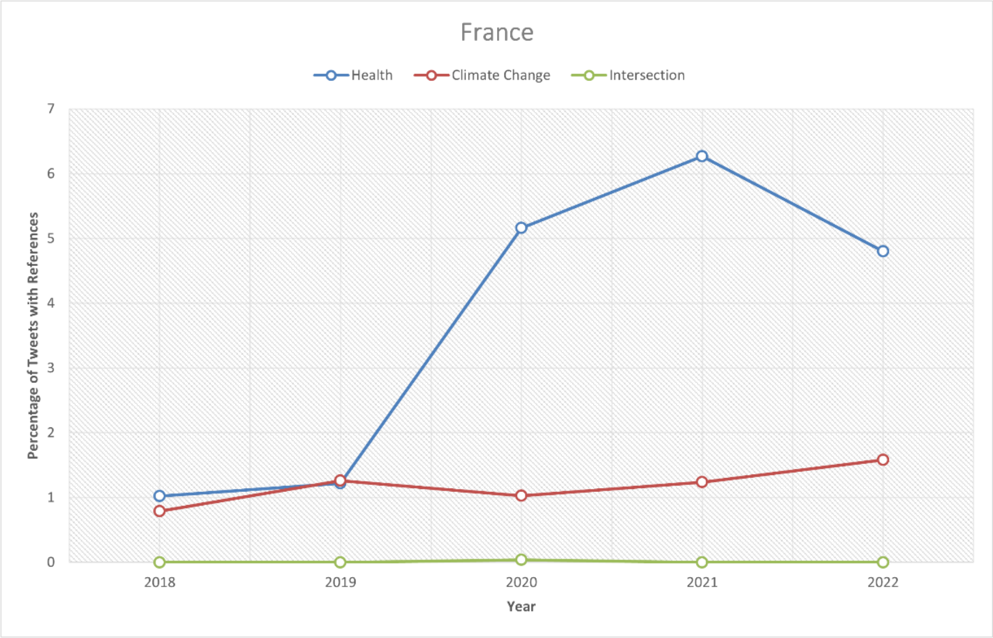

Figure 5.76 Percentage of Tweets with references to health, climate change and the intersection of the terms for France.

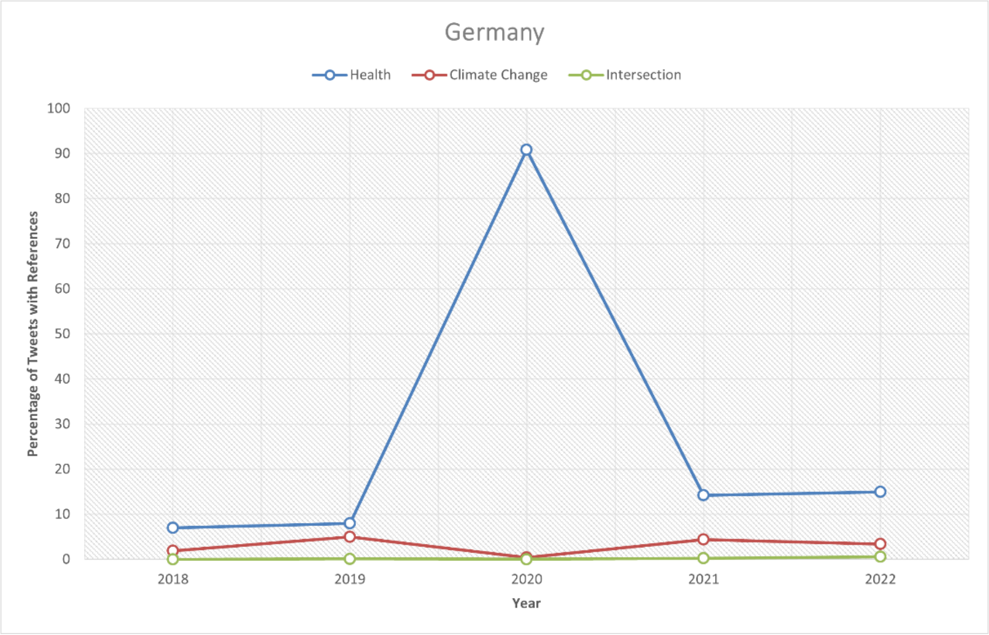

Figure 5.77 Percentage of Tweets with references to health, climate change and the intersection of the terms for Germany.

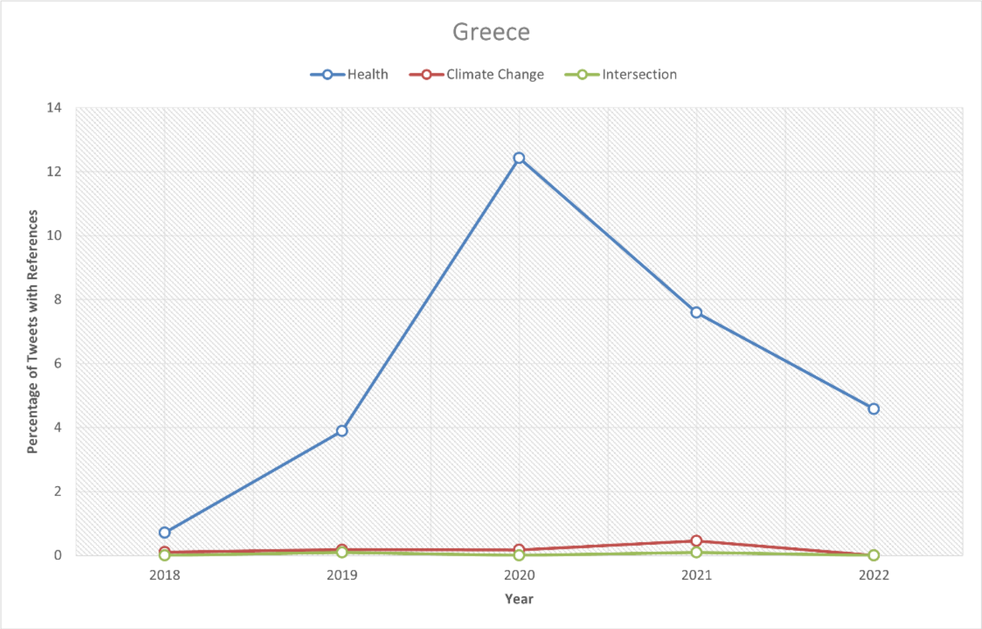

Figure 5.78 Percentage of Tweets with references to health, climate change and the intersection of the terms for Greece.

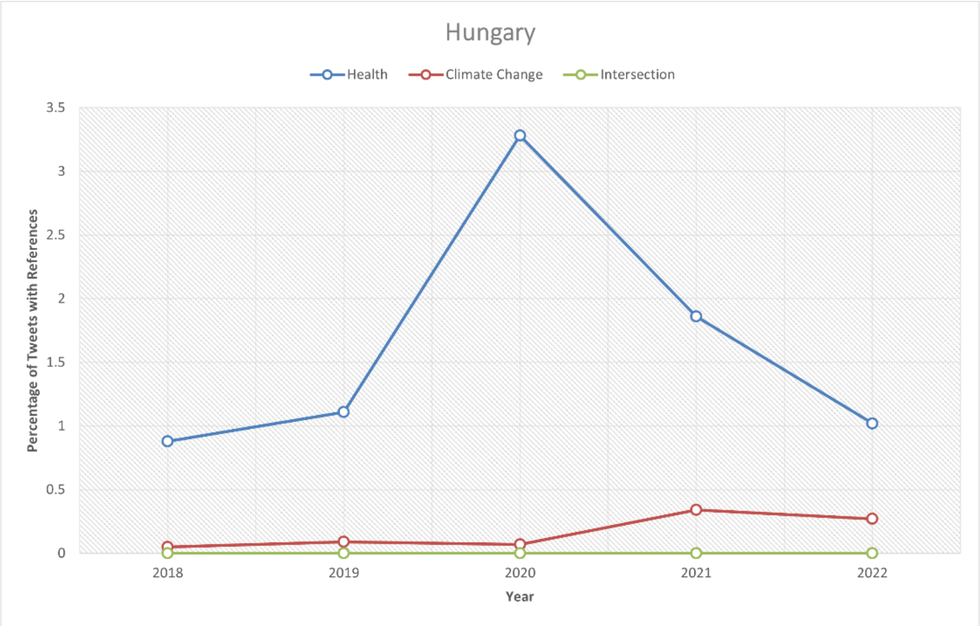

Figure 5.79 Percentage of Tweets with references to health, climate change and the intersection of the terms for Hungary.

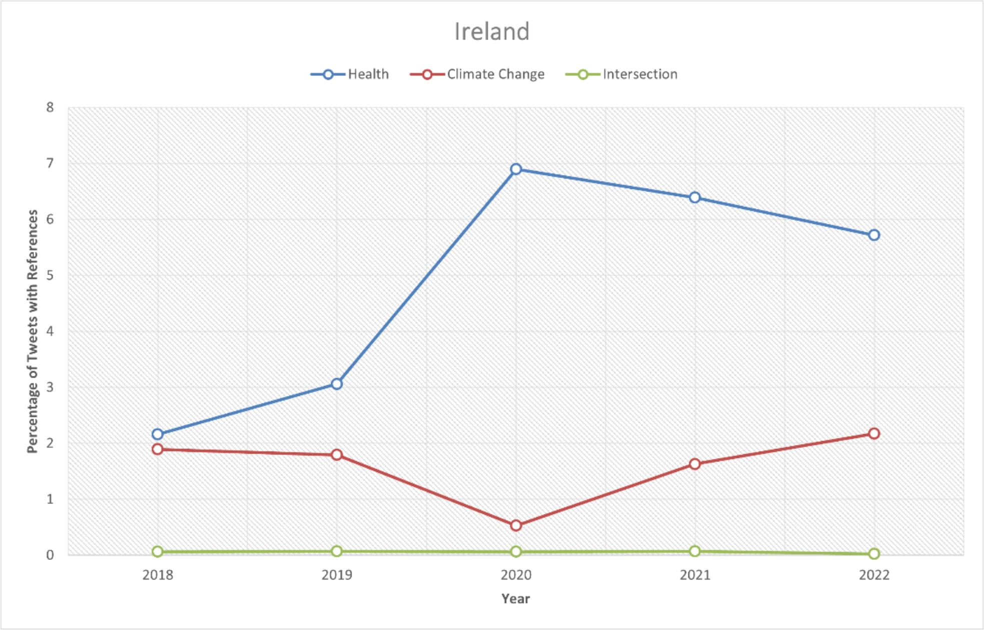

Figure 5.80 Percentage of Tweets with references to health, climate change and the intersection of the terms for Ireland.

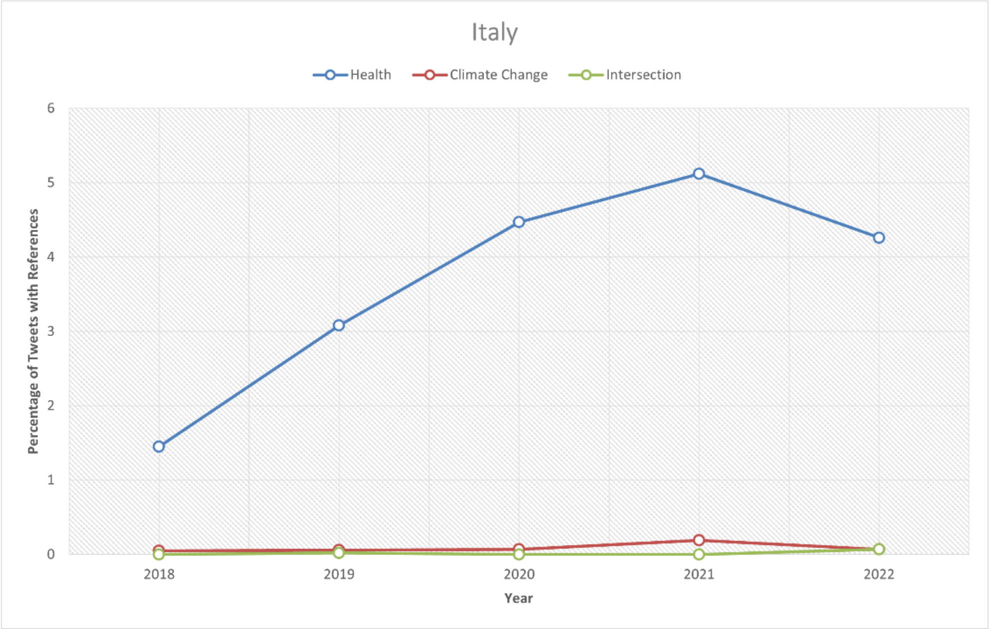

Figure 5.81 Percentage of Tweets with references to health, climate change and the intersection of the terms for Italy.

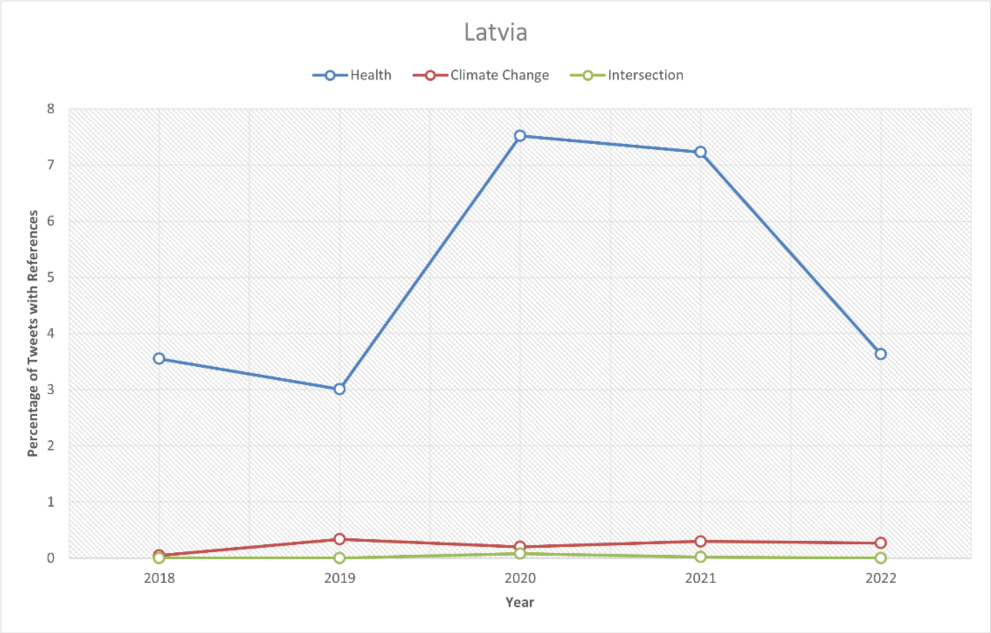

Figure 5.82 Percentage of Tweets with references to health, climate change and the intersection of the terms for Latvia.

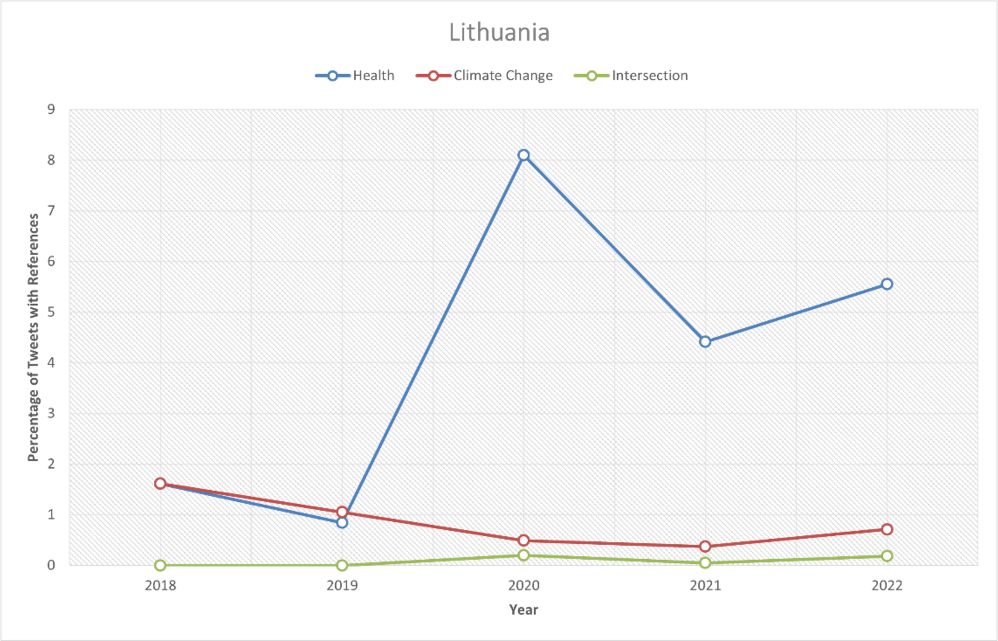

Figure 5.83 Percentage of Tweets with references to health, climate change and the intersection of the terms for Lithuania.

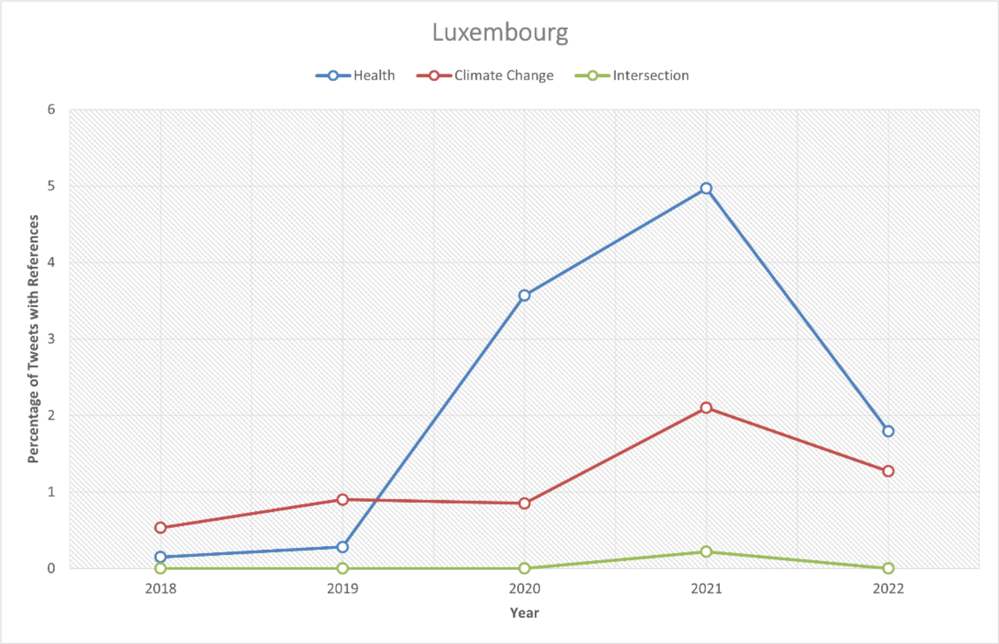

Figure 5.84 Percentage of Tweets with references to health, climate change and the intersection of the terms for Luxembourg.

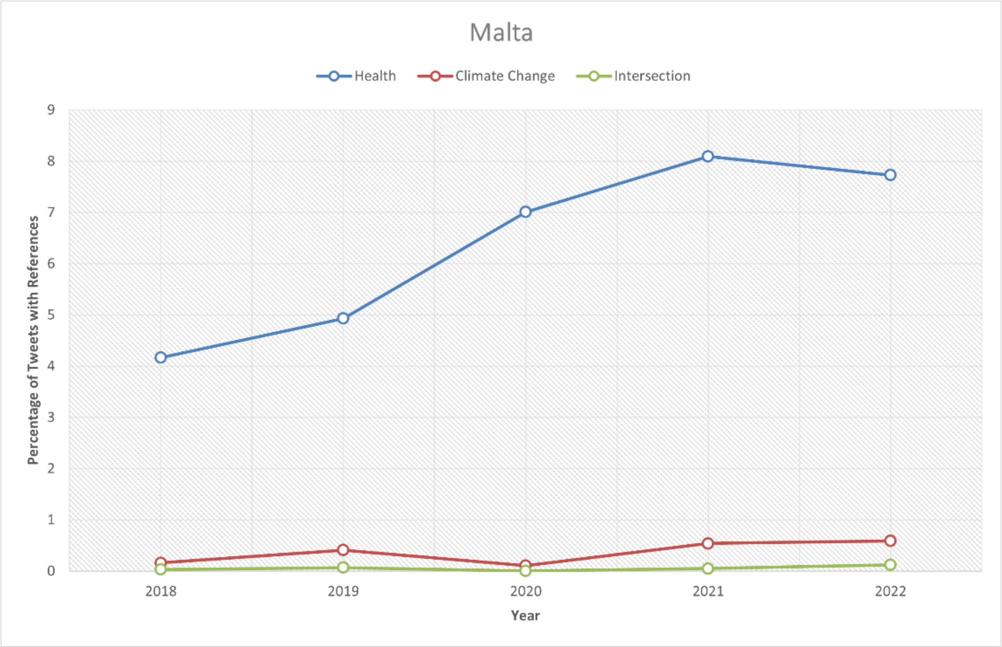

Figure 5.85 Percentage of Tweets with references to health, climate change and the intersection of the terms for Malta.

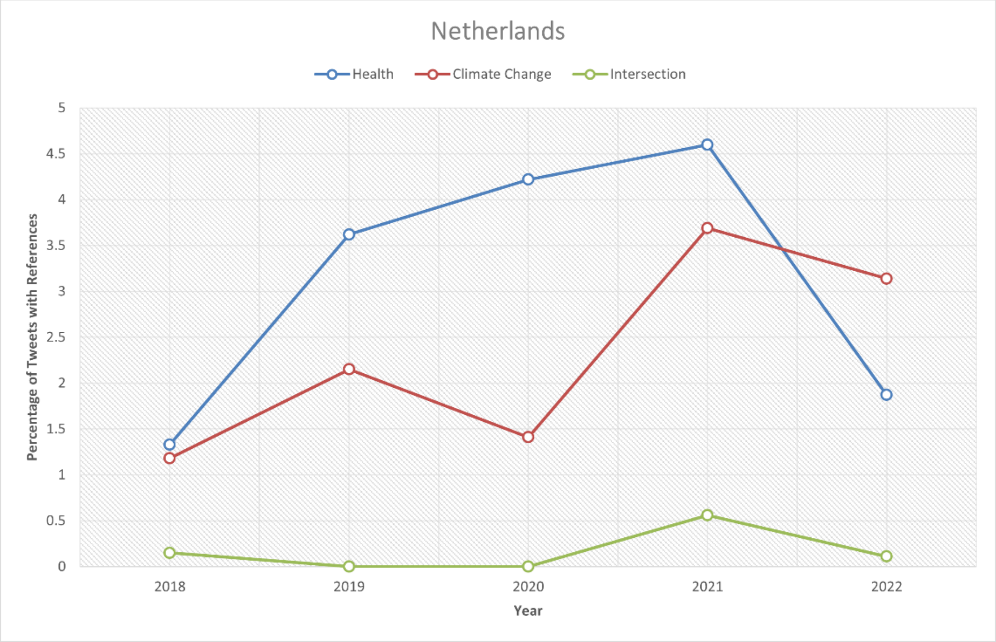

Figure 5.86 Percentage of Tweets with references to health, climate change and the intersection of the terms for the Netherlands (Kingdom of the).

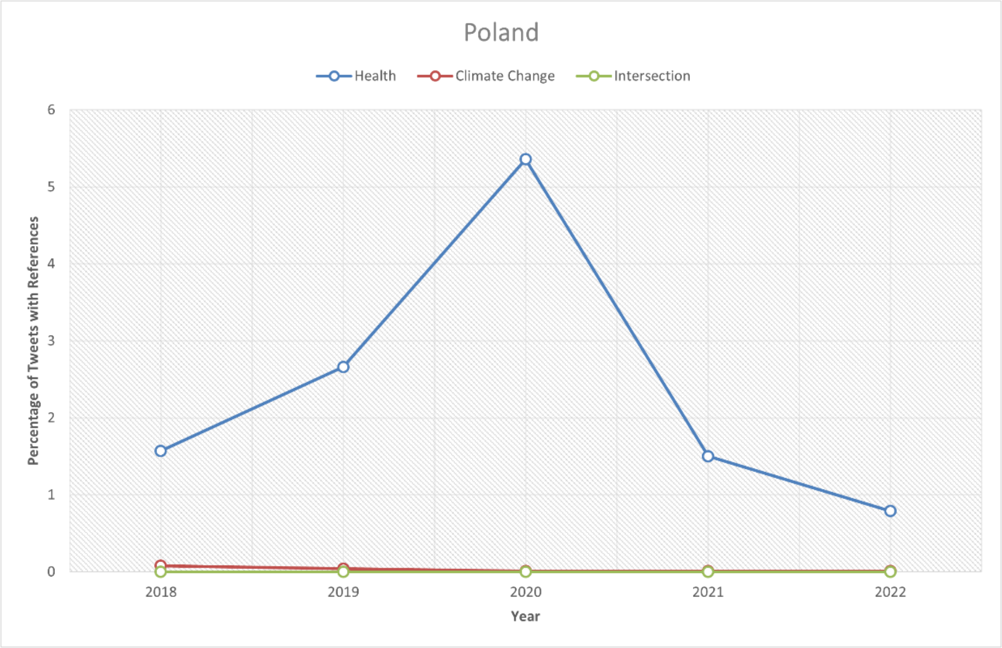

Figure 5.87 Percentage of Tweets with references to health, climate change and the intersection of the terms for Poland.

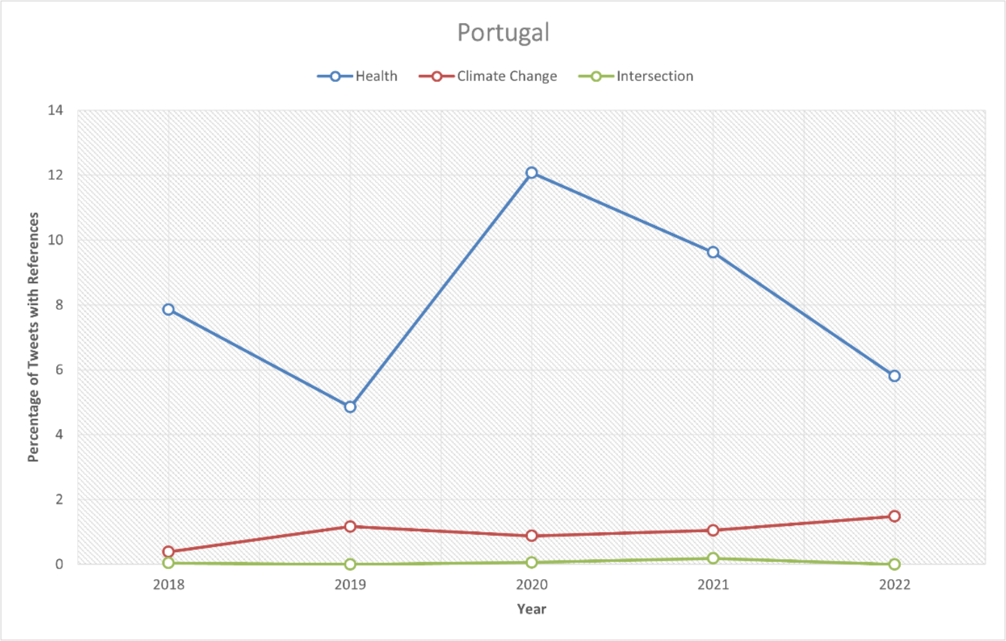

Figure 5.88 Percentage of Tweets with references to health, climate change and the intersection of the terms for Portugal.

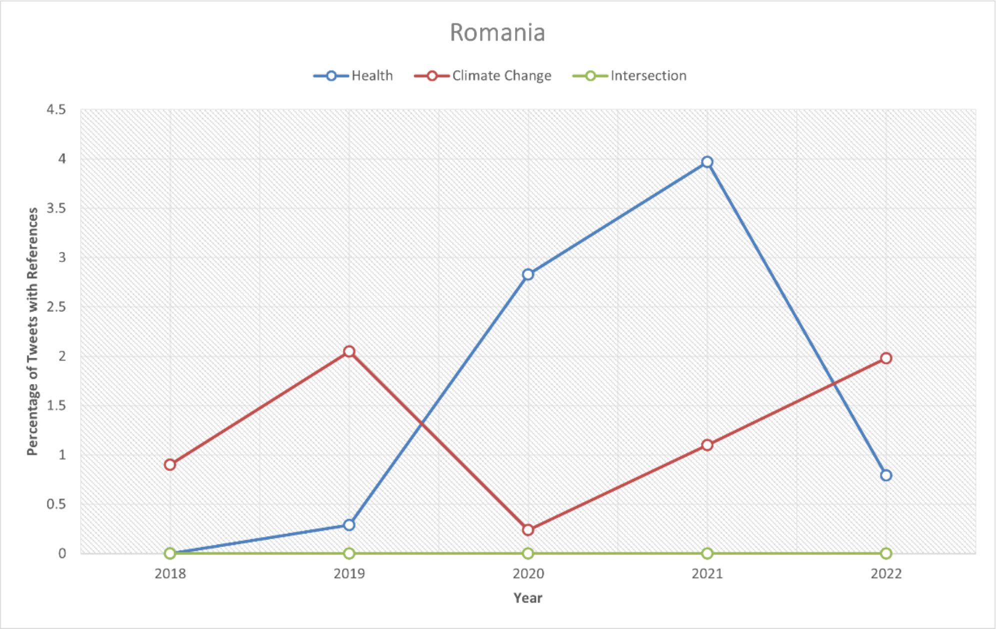

Figure 5.89 Percentage of Tweets with references to health, climate change and the intersection of the terms for Romania.

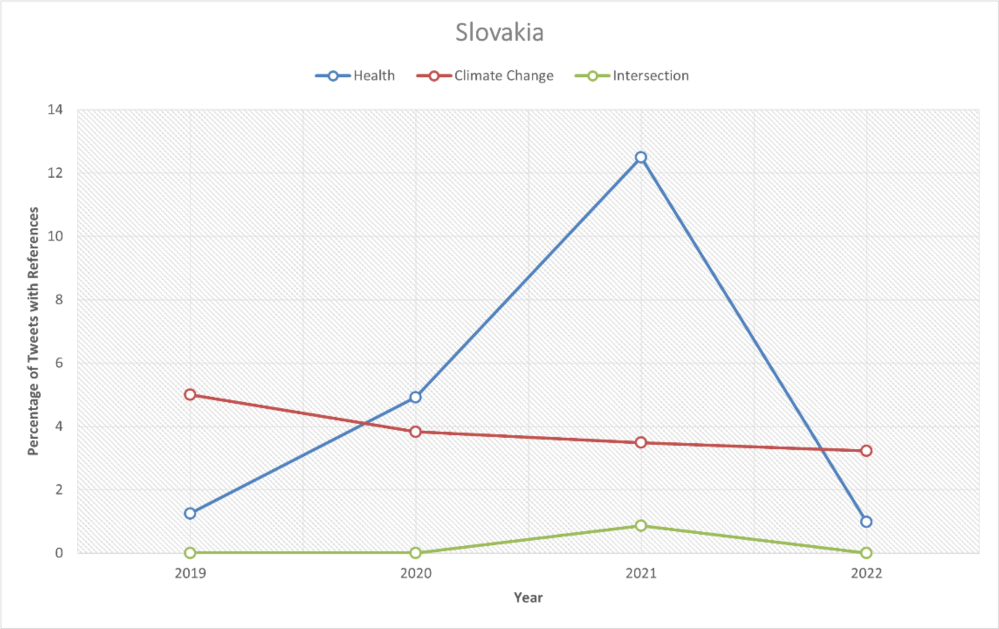

Figure 5.90 Percentage of Tweets with references to health, climate change and the intersection of the terms for Slovakia.

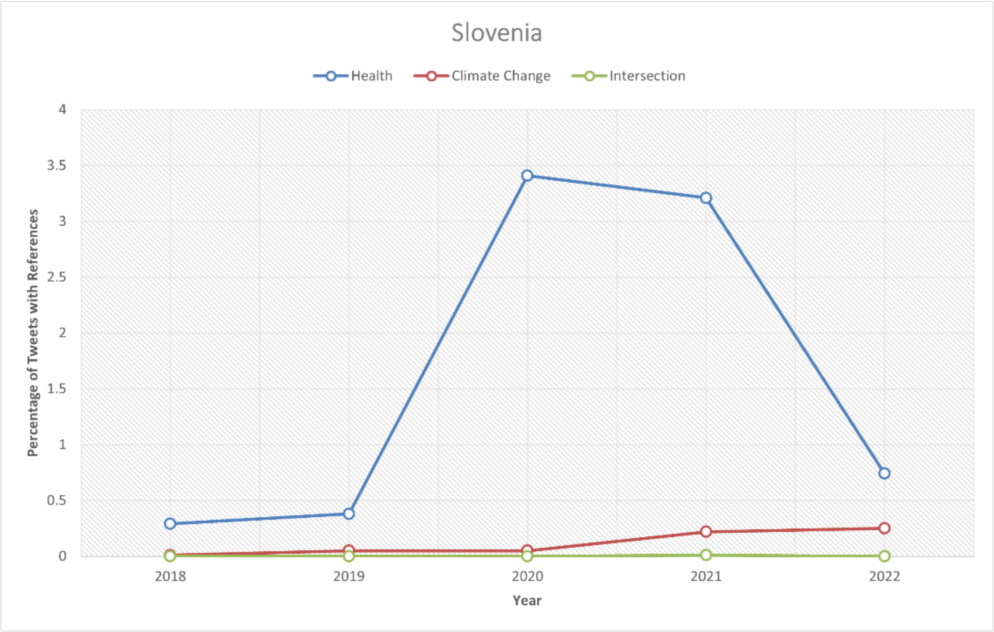

Figure 5.91 Percentage of Tweets with references to health, climate change and the intersection of the terms for Slovenia.

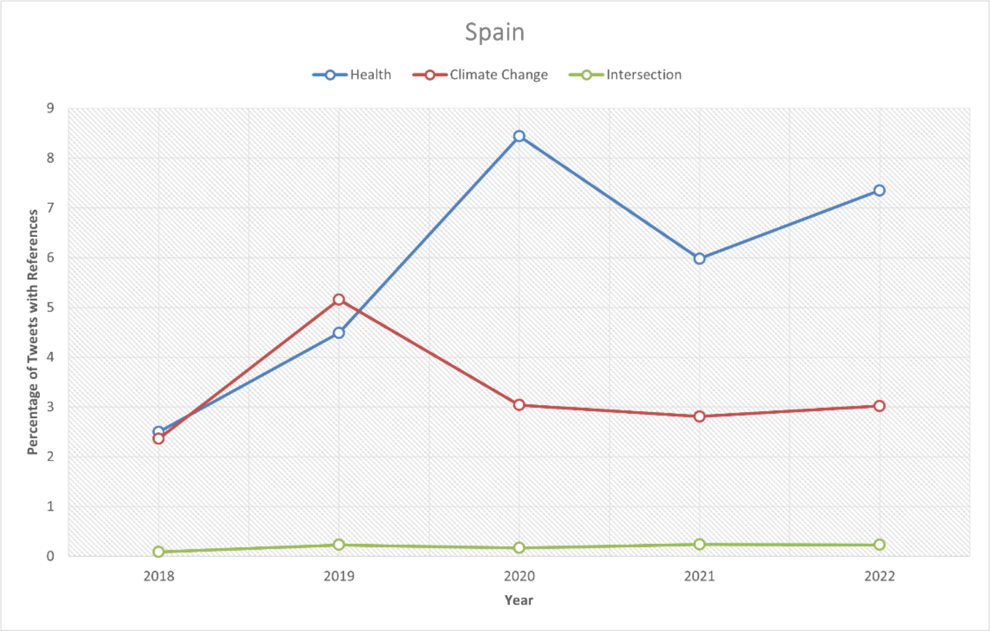

Figure 5.92 Percentage of Tweets with references to health, climate change and the intersection of the terms for Spain.

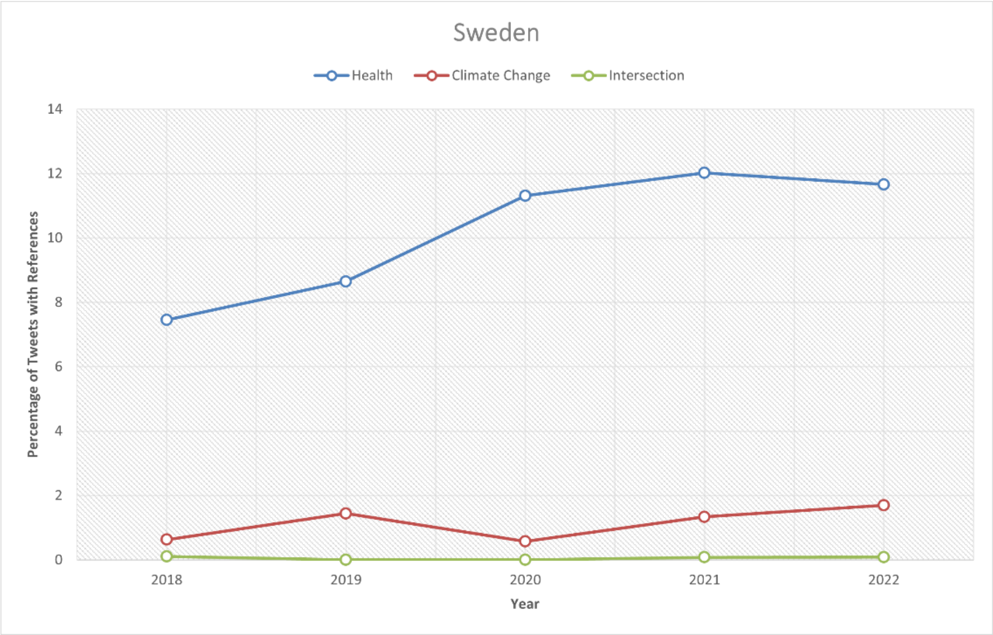

Figure 5.93 Percentage of Tweets with references to health, climate change and the intersection of the terms for Sweden.

5.4: Corporate sector engagement with health and climate change

Geographic Coverage of Europe

For this indicator, we included European Environment Agency (EEA) members and cooperating countries plus the United Kingdom of Great Britain and Northern Ireland.

Data

To produce this indicator, we draw on the publicly available UN GCCOP reports. The database consists of over 67,000 reports submitted by participants. The reports are available for companies based in more than 160 countries.

GCCOP reports are submitted in different languages. For the development of our indicator, we focused on three different groups of reports: the convertible reports available in English, the available reports from the European Environment Agency (EEA) region, and the available reports from the globe. Several files were corrupt or could not be converted into plain text format for analysis, so we only used the available ones. The distribution of available reports over time is presented in **Table 5.4.**

Table 5.4 Distribution of Available multilingual reports over time.

Year	Number of reports English	Number of reports EEA	Number of reports Global
2011	1052	1071	2057
2012	1422	1670	3015
2013	1597	1695	3231
2014	1721	1757	3186
2015	1812	1932	3472
2016	1974	1937	3573
2017	2006	2013	3724
2018	2035	2079	3751
2019	2238	2309	4060
2020	2063	2072	3554
2021	3112	3226	5737
2022	3328	3511	6089
Total	24360	25272	45449

There are only single GCCOP report submissions before 2011, thus we limit the sample of COP reports to the period 2011-2022. We pre-processed and prepared these documents for the application of natural language processing by converting the reports to plain text format as well as translating non-English documents to English texts. We used the open-source pretrained neural machine translation model Opus-MT under the HuggingFace pipeline to implement the translation task. Once the documents were translated in English, the text was pre-processed and prepared for analysis. Text processing involved removing punctuation and numbers (except 1 and 9 which are included in COVID terms); removing stop-words; and regularising (lowercasing). Ultimately the analysis was conducted in English. All pre-processing and analysis were carried out in Python using the NLTK package.

Methods

In this report, we measure companies' engagement with climate change and health in their' UN Global Compact Communication on Progress (GCCOP) reports. We use the publicly available COP reports to produce the indicators based on identifying references to key search terms on health and climate change. In order to produce these indicators, we focus on whether any of the climate change related terms appeared immediately before or after any public health terms in the COP reports. This was based on a search of the 25 words before and after a reference to a public health related term. We provide a full list of terms in **Table 5.5**.

Table 5.5 A comprehensive list of terms.

Health terms	Climate change terms	Covid terms	Gender terms	Inequality terms
malaria,	climate change,	covid-19,	gender,	inequality,
diarrhoea,	changing climate,	covid19,	male,	inequity,
infection,	climate emergency,	covid 19,	female,	injustice,
disease,	climate crisis,	corona, sars-cov-2,	man,	justice,
diseases,	climate decay,	covid,	men,	equity,
sars,	global warming,	corona virus	woman,	equality
measles,	green house,		women,	
pneumonia,	temperature,		sex	
epidemic,	extreme weather,			
epidemics,	global environmental change,			
pandemic,	climate variability,			
pandemics,	greenhouse,			
epidemiology,	greenhouse-gas,			
healthcare,	low carbon,			
health,	ghge,			
mortality,	ghges,			
morbidity,	renewable energy,			
nutrition,	carbon emission,			
illness,	carbon emissions,			
illnesses,	carbon dioxide,			
ncd,	carbon-dioxide,			
ncds,	co2 emission,			
air pollution,	co2 emissions,			
nutrition,	climate pollutant,			
malnutrition,	climate pollutants,			
malnourishment,	decarbonization, decarbonisation,			
mental disorder,	carbon neutral,			
mental disorders,	carbon-neutral,			
stunting	carbon neutrality,			
	climate neutrality,			
	climate action,			
	net-zero,			
	net zero			

Inequality Context

We also examine companies' awareness of gendered impacts of climate change and health, that of covid and that of inequality. To further produce indicators involving the COVID-19 pandemic, gender and inequality, we used additional search terms related to gender, COVID-19 and inequality to identify which of the intersection references also engaged with these issues. We provide a full list of terms in **Table 5.5**.

Analysis

We present some additional findings and breakdowns in this section. **Figure 5.94** presents the total number of references to climate change, health, and the intersection of climate change and health across for the GCCOP reports. Despite the increase in the proportion of companies engaging with the climate change-health linkages, the overall number of references remains fairly low and consistent, relative to the individual references to health and climate change – though, there has been an increase in references to the intersection of climate change and heath in the past two years.

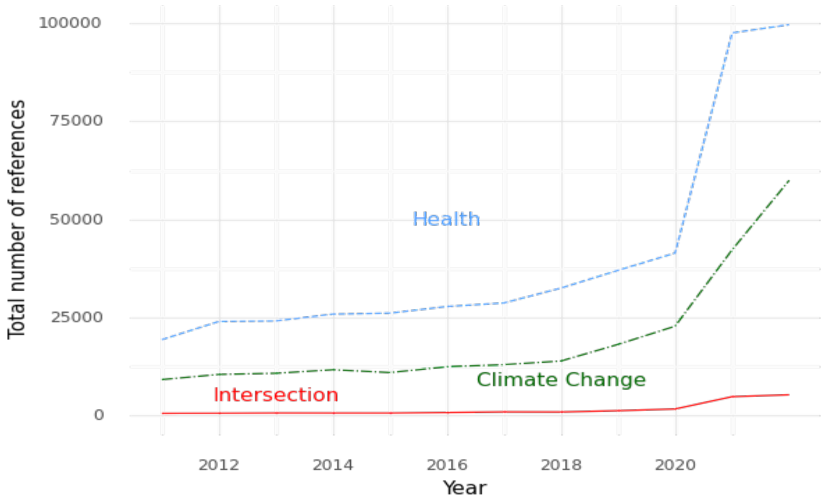

Figure 5.94 Total references to climate change, health and the intersection of climate change and health in GCCOP reports 2011-2022.

Figure 5.95 zooms in the intersection of climate change and health and shows the trend only for the references to both climate change and health. The figure shows that since 2018 – and particularly since 2020 – there has been a sharp rise in the number of references. However, the total number of references is still small.

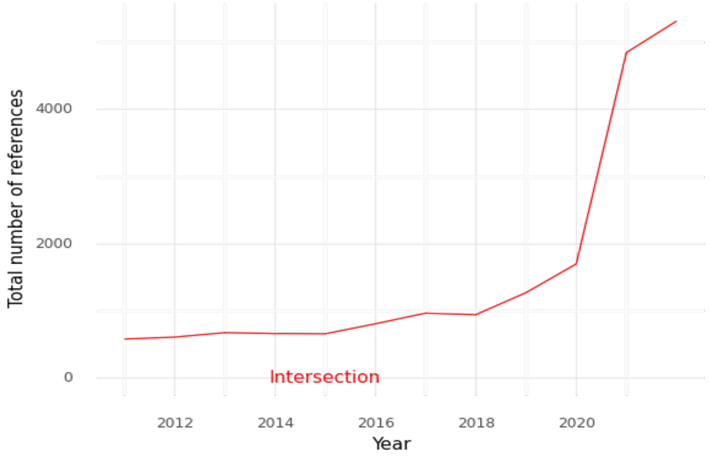

Figure 5.95 Total references to the intersection of climate change and health in GCCOP reports 2011-2022.

Figure 5.96 shows the average number of references to climate change, health and the intersections in the reports. The figure again demonstrated relatively low level of engagements with the health impacts of climate change, compared to the separate references to health and climate change.

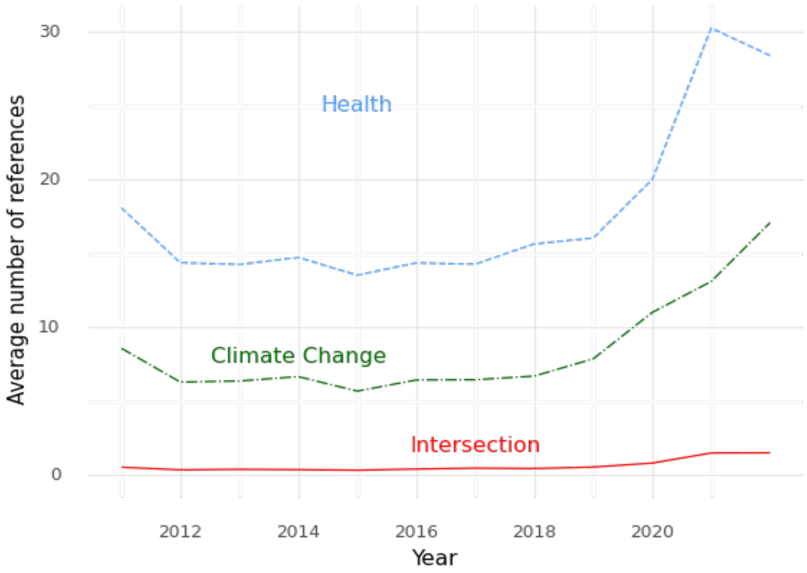

Figure 5.96 Average references to climate change, health and the intersection of climate change and health in GCCOP reports 2011-2022.

There is a growing awareness of the gendered impacts of climate change and health, thus we want to see if gendered impacts of climate change are salient among companies. We consider the extent to which references to the health dimension of climate change in companies' GCCOP reports engage with gender issues. We do this by further examining the references to the intersection of climate change and health (see Table 1 for the words used to capture gender impacts). Based on the additional search of the references to the climate change-health intersection using these gender-related keywords, we identified references to the health dimensions of climate change with a gender focus in companies' annual GCCOP reports. **Figure 5.97** presents annual references to the gender dimensions of climate change and health in UN Global Compact COP reports between 2011 and 2022. The figure shows a steady increase in engagement between 2015 and 2017. Despite small decreases in the proportion of references in 2018 and 2020, the overall trend shows a clear increase in the mentions of the gendered impacts of climate change. Although in 2011, references on the gendered impacts were below 10%, in 2022 the references to the gender dimensions are approximately 18%.

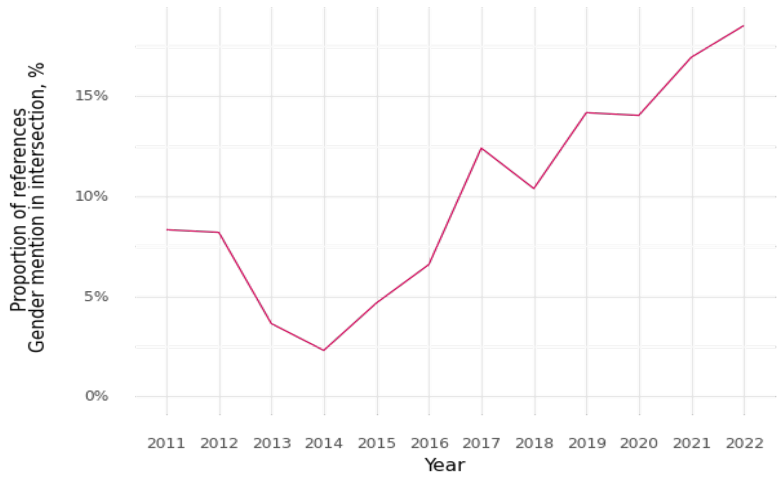

Figure 5.97 Proportion of references to the intersection of health and gender that include a reference to gender in GCCOP reports 2011-2022.

We additionally examine the impacts of inequality to the climate change-health intersection using these inequality-related keywords. **Figure 5.98** presents annual references to the inequality dimensions of climate change and health intersection between 2011 and 2022. The figure shows a steady increase in engagement between 2016 and 2022 with a decrease between 2014 and 2015. Overall, we observe a notable increase of inequality-related impacts from below 6% in 2011 to 25% in 2022.

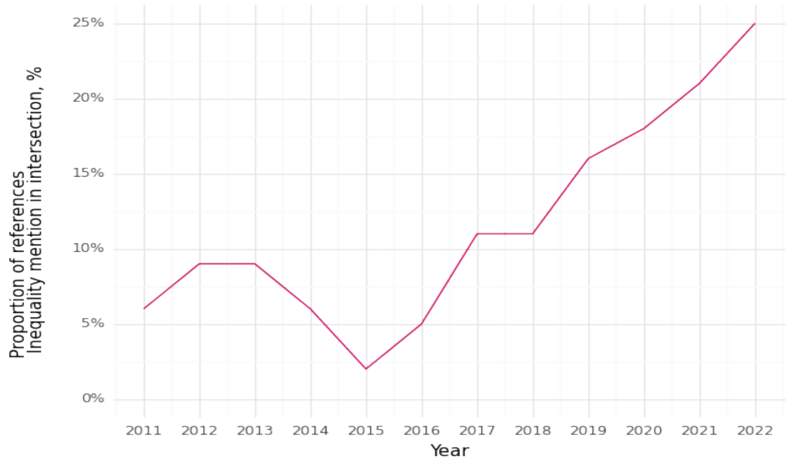

Figure 5.98 Proportion of references to the intersection of health and climate-change that include a reference to inequality in GCCOP reports 2011-2022.

We also consider engagement across different sectors. It might be the case that companies that are considered part of the polluting sectors tend to report more the intersection of climate change and health. **Figure 5.99** Shows the proportion of companies that highlight climate change (green bar), health issues (blue bar), and their intersection (red bar). We can see that companies of the real estate investment trusts sector (~ 50%), the tobacco sector (~ 48%), the alternative energy sector (~ 40%), the mining sector (~ 40%), and the oil sector (~ 38%) are the ones that mostly engage with the health impacts of climate change.

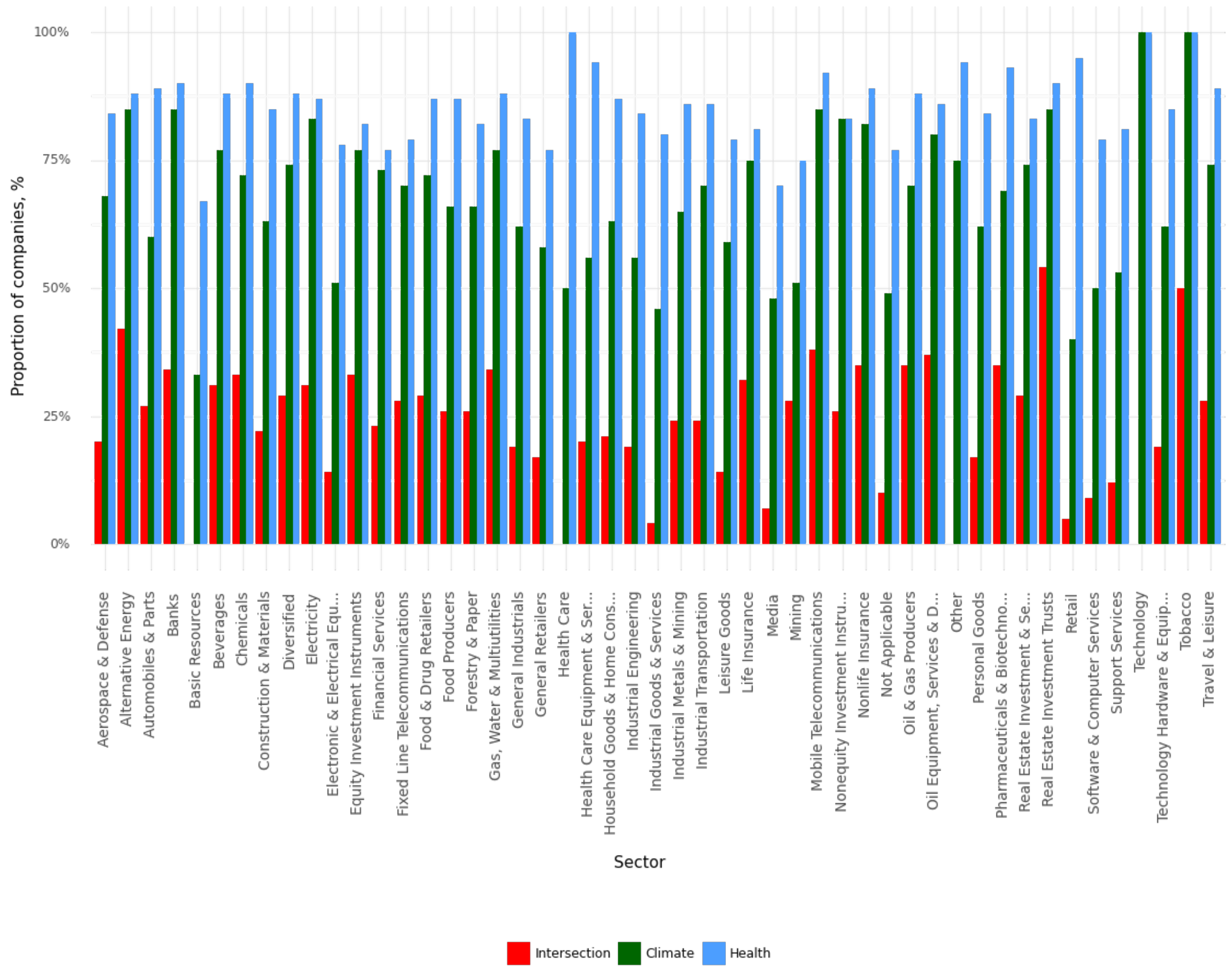

Figure 5.99 Proportion of companies by sector that mention health, climate change, and the intersection of health and climate change since 2011.

Lastly, we consider the changes on the references to climate change, health issues, and their intersection of companies within the health sector. **Figure 5.100** shows the proportion of companies focusing on health, climate change, and their intersection. Unsurprisingly, companies within the health sector constantly highlight health issues and throughout the year more than 80% of companies mention health issues in their reports. In addition, climate change issues started gaining momentum especially after 2016, and companies references to climate change increases since then. In 2022 more than 75% of companies mention climate change in their reports. As for their intersection, although we observe an increase – especially after 2016 –, the proportion of companies that highlight health related issues of climate change is still small (~ 30%).

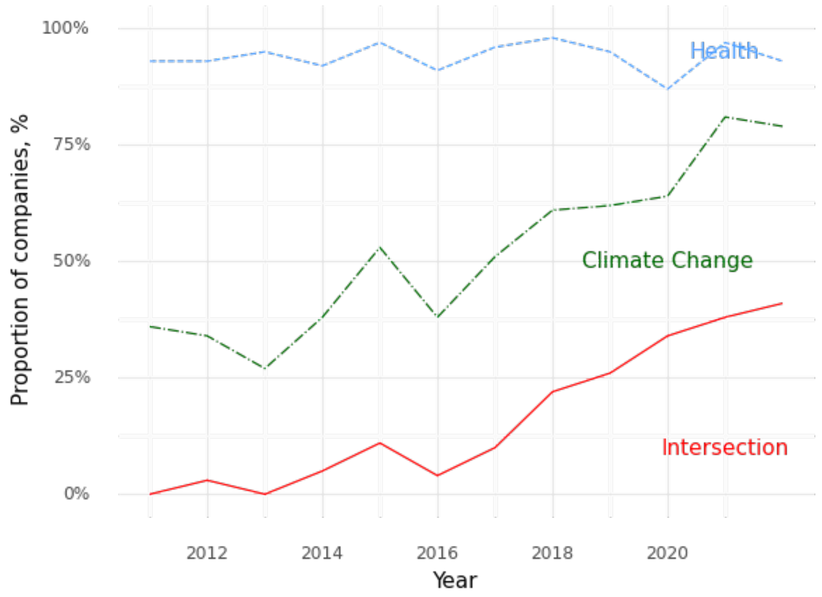

Figure 5.100 Proportion of companies within the health sector that mention health, climate change, and the intersection of health and climate change in GCCOP reports 2011-2022

Figure 5.101 presents the total number of references to climate change, health, and the intersection of climate change and health across for the GCCOP reports from companies within the health sector. Despite the increase in the proportion of companies engaging with the climate change-health linkages, the overall number of references remains fairly low and consistent, relative to the individual references to health and climate change – though, there has been an increase in references to the intersection of climate change and heath in the past two years.

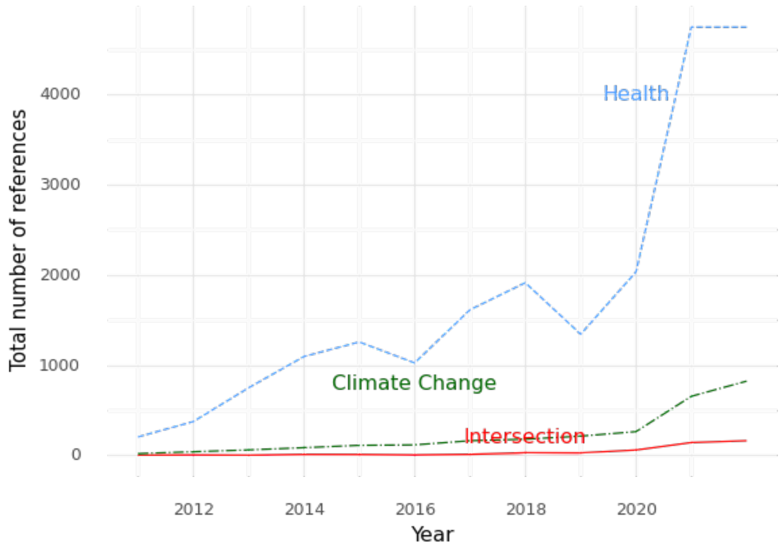

Figure 5.101 Total references to climate change, health, and the intersection of climate change and health, 2011-2022.

5.5: Media engagement with health and climate change

The media plays a significant role in shaping public perception and facilitating communication between the public and policy makers, including in the areas related to health and climate change. One crucial aspect of this influence is the media's ability to frame policy-related issues, deciding what aspects to highlight or downplay. Understanding the dynamics of this media-policy-public link is essential for gaining insight into the shortcomings and distortions that currently hinder efficient information dissemination from policy makers to the public. Here we work with the prominent European media outlets and use textual analysis techniques to track the engagement in climate change and health rhetoric in their discourse on social media. The findings from this study can be used to inform public health campaigns, resource allocations, and policy priorities for improving public policy media communication.

Studying the frequency of mentioning a particular topic that holds important policy relevance is crucial for several reasons. It provides insights into the circulation of the issue within the media and helps assess the expected level of public attention directed towards that specific issue.[224,225,226] The practices employed by mass media have a far-reaching impact on translating complex scientific concepts into policy discussions and shaping public perceptions regarding environmental issues, technology, and risk. However, it is crucial to assess the frequency of media coverage on health issues to determine whether these topics receive adequate attention and public attention is one of the best predictors of vital health-related knowledge: proper communication of environmental and health-related news could be lifesaving in certain situations.[225]

The indicator is based on the frequency of health-related and climate change-related issues appearing in media coverage, and is therefore a reliable measure of media-sponsored public awareness of the issues for the following reasons:

First, analyzing tweets from these outlets helps us understand the issues they prioritize, amplify, or downplay, giving insights into the media's influence on public attention to climate change and health topics.[227] Importantly, by incorporating media outlets from various EU countries, we provide a cross-country perspective that allows for a comprehensive picture of health-related issue mentions across the European Union.[228] This approach helps to capture the diverse discussions and variations in media-sponsored public attention to health topics within different European states. By considering media outlets from multiple countries, we enhance our understanding of the broader European context and gain insights into the similarities and differences in public attention and media coverage of health-related issues across the European states.[229]

Second, we gain access to discussions and representations of climate change and health topics by analyzing tweets from media outlets. This allows us to capture the immediate attention and ongoing dialogue surrounding these issues, providing a more up-to-date approximation of public attention.[230,231]

Third, social media platforms like Twitter have a broad user base, making them a valuable source for approximating public attention.[232] Analyzing multilingual tweets allows us to capture discussions from diverse

regions and languages, providing a more comprehensive understanding of global or localized attention to climate change and health on social media.[233] Because social media platforms like Twitter are designed as interactive and encourage public engagement in co-production of news content, allowing users to share, retweet, and comment on specific topics, health related topics discussed there provide an insight into how much attention these critical issues receive.

Geographic Coverage of Europe

We collected data on media outlets on all the 38 countries of EEA + United Kingdom of Great Britain and Northern Ireland. However, due to the language specifics restrictions described in the limitations, we only present the results for 29 counties and 172 total number of outlets.

Data

We selected the most prominent media outlets across 38 member and associate member countries of the European Environment Agency (**Table 5.6**). To compile the list and assign ideological labels to the outlets, we utilized the predictive capabilities of the GPT-4 model, based on the GPT chat. To access the media rhetoric, we retrieved all content posted on the official Twitter accounts of these outlets for the period of 2022. The results yielded 169 media outlets with valid Twitter accounts spanning 29 countries (refer to the limitations section for additional information regarding key term translations and the absence of official translations for Albania, Bosnia and Herzegovina, Montenegro, North Macedonia, Serbia, Kosovo (under UNSC resolution 1244), as well as language complexities involving Danish, Dutch, Greek, and Swedish, resulting in missing intensity scores for Denmark, Netherlands (Kingdom of the), Dutch-speaking Belgian outlets, Sweden, Greece, and Greek-speaking Cypriot outlets). The utilized media outlets are known to have valid and active Twitter accounts.

Methods

With the dataset of multilingual tweets written by media outlets, we performed a search through the text of each tweet to identify if they discuss climate change and health-related topics. To do so, we first identified the list of climate change and health-related keywords that we intended to search. We used the official glossary of the European Environment Agency and excluded common words and words that could have multiple meanings unrelated to environment and health issues. The list of excluded terms can be found in **Box 5.5** and is presented in English, although respective translations in other languages have also been excluded.

We develop our indicator of health-climate change rhetoric engagement intensity as a monthly proportion of tweets containing at least one term from our health-related term list (**Box 5.4**) in relation to the number of tweets mentioning at least one of the key terms from the climate change term list written by each media outlet in a certain language. To properly calculate the intensity, we first evaluate the number of tweets written in a specific language (usually the official language of the country in which media outlet is operating), and inside those we searched

through the key terms about climate change. The number of the unique tweets with at least one of such key terms mentions we use as a denominator for our indicator.

The language processing is quite important in our analysis and consists of two parts depending on whether language is part of Twitter supported languages or not:

- If the language is supported by the Twitter platform, it is automatically identified and returned as part of the tweets meta-data. This is valid for the following languages: Bulgarian, Czech, German, Greek, Danish, Dutch, English, French, Icelandic, Italian, Hungarian, Latvian, Norwegian, Polish, Portuguese, Romanian, Swedish, Slovenian, Spanish, Turkish. In this case, we first subset the tweets based on such language identifiers, and then calculate the total number of tweets written by a given media outlet in each language. And then use this total amount of tweets to search through the keywords in a corresponding language.
- If the language is not supported by Twitter (such as Croatian, Estonian, Irish, Lithuanian), the language will automatically be flagged as unidentified. In this case, we use all the returned tweets as a denominator, and search keywords translated to the language that such media outlets use.

We utilized an originally written computational algorithm to identify the lists of tweets containing each keyword from a given list for each media outlet on a monthly basis throughout 2022. These lists were combined to create a master list of unique tweets, excluding any duplicates resulting from multiple keywords mentioned in the same tweet. The number of unique tweets represented the total count of climate change keyword-mentioned tweets, which served as the denominator for our indicator. For the numerator part we used those selected tweets that mention at least one key term from the climate change rhetoric, and in that search through the health-related terms (**Box 5.4**). The number of health-related tweets in those climate change tweets results in our numerator.

The resulting indicator of rhetoric intensity was obtained by dividing the number of health-related tweets with the mentioned keywords by the number of climate change-related tweets written by the media outlet. This allows us to identify the health specific rhetoric described as part of the general climate change rhetoric.

Figure 5.102 presents the distribution of intensity across the year. On average, engagement in climate change and health co-benefits rhetoric was observed in approximately 8% of the overall media textual corpus. Notably, this percentage exhibited a slight peak over the summer and even larger peaks over the winter months, which could be attributed to abnormal summer and winter temperatures and heightened discourse surrounding related issues.

Figure 5.103 demonstrates the monthly dynamic for each of the 29 countries, and additionally allows us to provide a cross-country comparison. Here we can see that on average the intensity varies around 8-10% with individual outliers such as Hungary or Malta due to overinflated confidence intervals.

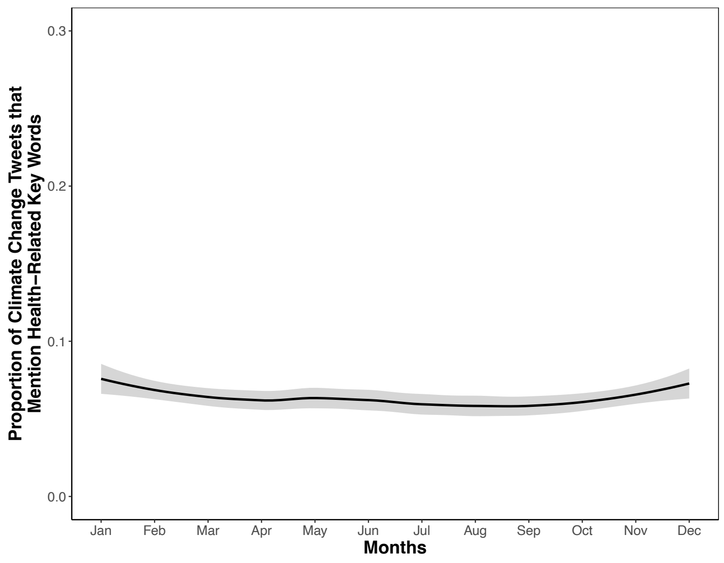

Figure 5.102 Average Health Intensity Score across All the Media Outlets Per Months.

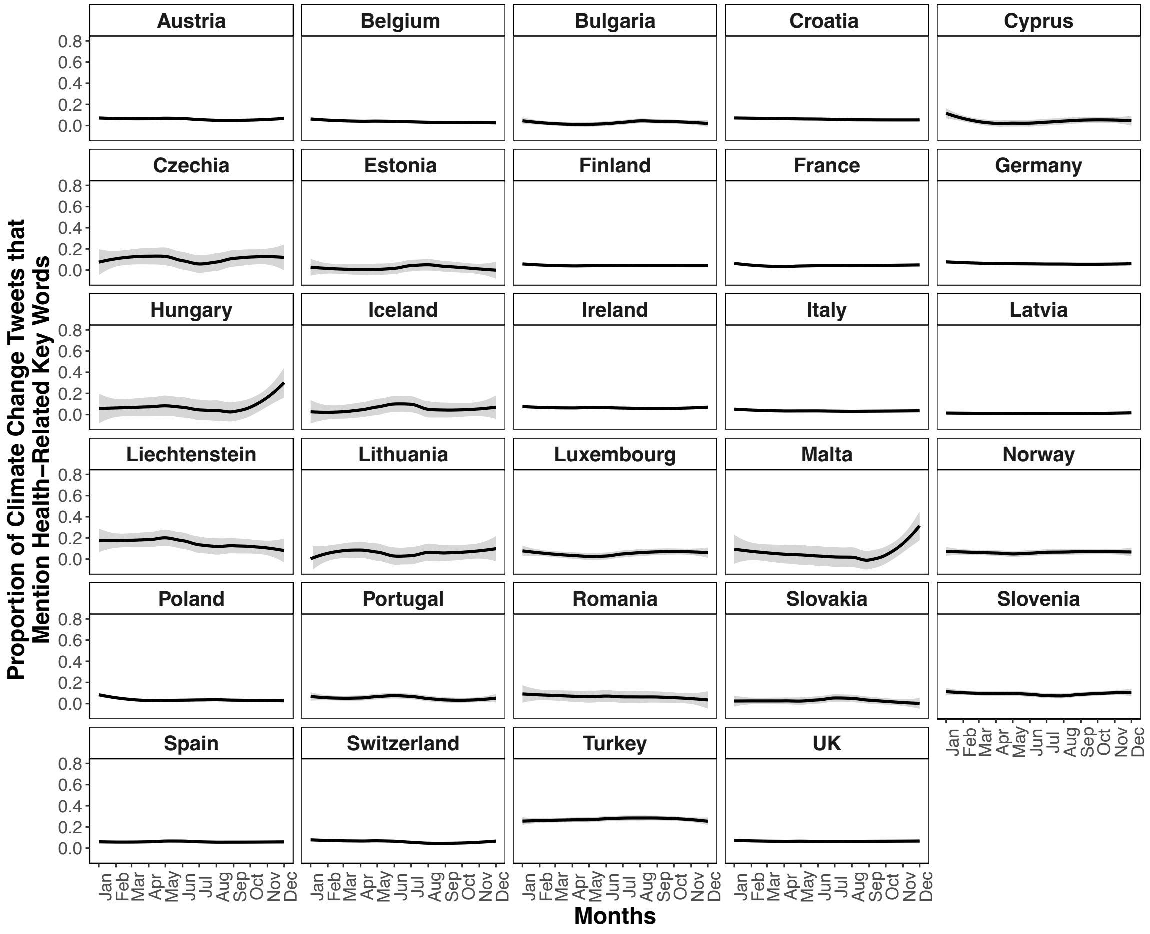

Figure 5.103 Monthly Health Intensity Scores Across the States.

Caveats

Several limitations raised in the process of data collection and data analysis, that we aim to address in the next iterations of the report.

1. We utilize a non-random list of media outlets for our study. We obtain the data from open sources provided by the GPT 4 Chat platform and select the largest and most prominent media outlets in each country. However, it's important to note that our list of media outlets is not exhaustive, and we primarily captured centrist or populist ideological views with this selection. In future iterations of our report, we plan to expand the list of media outlets to include a more diverse range of sources. This will allow for a broader exploration of rhetoric presented by different media outlets, providing a more comprehensive understanding of the landscape.

2. To search through the climate change and health rhetoric, we utilize the official European Environment Agency (EEA) glossary, which is published in English and includes translations in the official languages of EEA member countries. However, it should be noted that Albania, Bosnia and Herzegovina, (under UNSC resolution 1244), North Macedonia, Montenegro, and Serbia are not full members of the EEA, resulting in the absence of translations in the glossary for their official languages. Consequently, we exclude the rhetoric from these countries in the current iteration of our report. For future versions, we plan to address this issue by employing machine translation techniques to accurately translate all the glossary keywords and include the rhetoric from these countries in our analysis.

3. The utilized glossary includes the lemmatized version of all the terms. We exclusively utilize these lemmatized forms and did not generate all potential grammatical forms of the words for searching and querying purposes. It should be noted that some languages, such as Spanish, French, and Bulgarian, are more sensitive to lemmatization, which can create imbalances in cross-country intensity estimates and potentially bias cross-country comparisons. However, this should not impact the within-country variation between media outlets, as the same glossary is applied to all tweets written by media outlets in each language within the country. One approach to address this issue is to employ stemming of words, which involves searching through word stems to capture a majority of grammatical forms. However, this approach may present challenges, such as the inclusion of small and potentially meaningless stems that may capture irrelevant rhetoric. For instance, using the stem "gen" to query for "genetic" may also capture words like "gender," which might be unrelated to climate change-health rhetoric. This could result in an overestimation of the intensity indicator. Therefore, we choose not to perform stemming and instead opt for potentially underestimating effects. In future iterations of this indicator, our plan is to expand the list of keywords by including all possible grammatical forms of the keywords in the search, thus providing a more comprehensive analysis.

4. Another language specific problem that we face is related to special characters that are used in Danish, Dutch, Greek, and Swedish languages. These special characters prevent the automated regular expressions querying. We aim to address this problem in the next iteration of the report.

5. In the current iteration of the report, we only explore textual rhetoric and build an indicator of engagement in climate change and health textual discourse expressed by media outlets. In the continuation of this report, we plan to explore a visual representation that media outlets use to address such climate change and health rhetoric.

Future Form of the Indicator

6. Missing keywords translations for the cooperating members of the EEA;
7. Resolve language specifics issues for Danish, Dutch, Greek, and Swedish languages;
8. Expand the time-series aspect of the data, by including media outlets rhetoric prior to 2022.

Analysis

By extracting all rhetoric shared by the analyzed media outlets throughout the year 2022, we obtained a dataset comprising 3,727,118 multilingual tweets. Among these, 547,786 tweets contained at least one of the selected keywords from the list of climate change in any language, which on average accounts for approximately 14.7% of the total tweets. And from the tweets with the climate change keywords mentions 44,766 mention health related keywords (**Box 5.4**) which means that on average about 8.2% of the general climate change specific rhetoric mentions health-specific issues.

In the cross-country comparison, we find that even with a relatively small sample of media outlets that represent the most prominent news suppliers with large coverage in each country, we do not observe that much of heterogeneity across different countries (**Figure 5.104** shows the aggregated results on the intensity of the media outlets' engagement in the rhetoric about health-climate change). This can serve as a proxy on how much attention is being paid to the climate change and health co-benefits in policy agenda and in the news opinions.

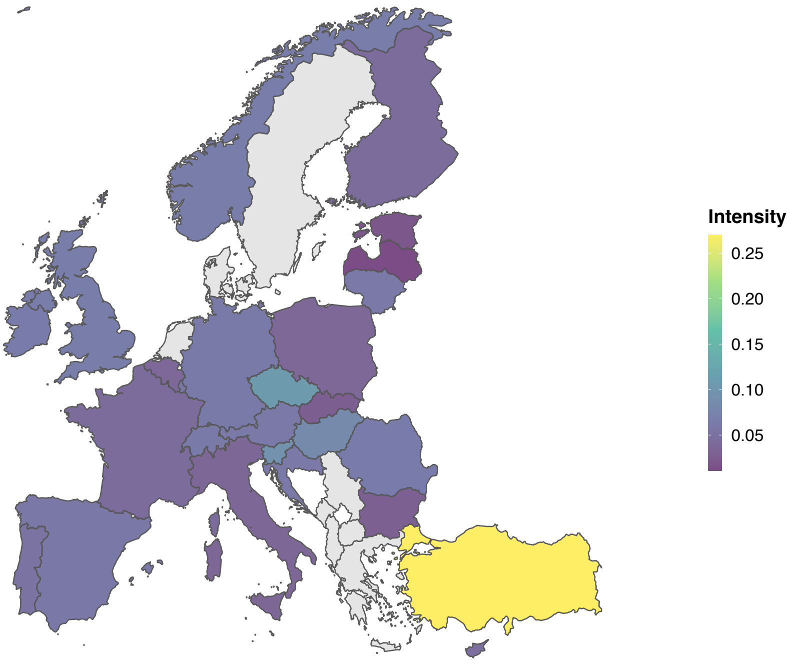

Figure 5.104 Map of the Cross-Country Comparison of Engagement Intensity.

Table 5.6 List of the countries with the media outlets, their handles and the languages used to search through the key words.

Country	Media Outlet
Austria	derStandardat
Austria	DiePressecom
Austria	Heute_at
Austria	kleinezeitung
Austria	krone_at
Austria	KURIERat
Austria	ORF
Austria	sn_aktuell
Austria	WienerZeitung
Belgium	ladh
Belgium	lalibrebe
Belgium	lecho
Belgium	lesoir
Belgium	RTBFinfo
Bulgaria	bg_eurovision
Bulgaria	CapitalBg
Bulgaria	dnevnik
Bulgaria	SegaBG
Bulgaria	www24chasa
Croatia	24sata_HR
Croatia	hrtvijesti
Croatia	jutarnjihr
Croatia	N1infoZG
Croatia	novilisthr
Croatia	vecernji_list
Cyprus	cyprusmail
Czechia	Aktualnecz
Czechia	CRozhlas
Czechia	CzechTV
Czechia	mfdnes
Czechia	novinkycz

Estonia	aripaev_ee
Estonia	DelfiEE
Estonia	errnews
Estonia	postimees
Finland	hsfi
Finland	iltalehti_fi
Finland	iltasanomat
Finland	KauppalehtiFi
Finland	talouselama
Finland	turunsanomat
Finland	yleuutiset
France	BFMTV
France	FRANCE24
France	franceinfo
France	Le_Figaro
France	le_Parisien
France	lemondefr
France	LesEchos
France	LEXPRESS
France	libe
France	lobs
Germany	ARD_Presse
Germany	BILD
Germany	derspiegel
Germany	faznet
Germany	handelsblatt
Germany	SZ
Germany	welt
Germany	ZDF
Germany	zeitonline
Hungary	444hu
Hungary	hvg_hu
Hungary	indexhu
Hungary	nepszava
Iceland	Iceland_Review
Iceland	mblfrettir
Iceland	RUVfrettir
Ireland	Independent_ie
Ireland	irishexaminer
Ireland	IrishMirror
Ireland	IrishSunOnline
Ireland	IrishTimes
Ireland	rtenews
Ireland	thejournal_ie
Italy	Corriere
Italy	fattoquotidiano
Italy	ilgiornale
Italy	ilmessaggeroit
Italy	LaStampa
Italy	RaiNews
Italy	repubblica
Italy	SkyTG24
Italy	sole24ore
Latvia	DelfiLV
Latvia	DienaLv
Latvia	LatvijasAvize
Latvia	nralv
Latvia	TVNET_zinas
Liechtenstein	Volksblatt
Lithuania	DELFILietuva
Lithuania	LRTenglish

Luxembourg	le_quotidien_lu
Luxembourg	lessentiel
Luxembourg	RTLlu
Luxembourg	tageblatt_lu
Luxembourg	Wort_LU
Malta	LovinMalta
Malta	maltainde
Malta	maltatoday
Malta	TelevisionMalta
Malta	TheTimesofMalta
Norway	Aftenposten
Norway	dagbladet
Norway	DN_no
Norway	Klassekampen
Norway	NRKno
Norway	vgnett
Poland	DGPrawna
Poland	gazeta_wyborcza
Poland	OnetWiadomosci
Poland	PAPinformacje
Poland	PolsatNewsPL
Poland	rzeczpospolita
Poland	tvn24
Poland	tvp_info
Portugal	dntwit
Portugal	expresso
Portugal	JNegocios
Portugal	JornalNoticias
Portugal	Publico
Portugal	rtppt
Portugal	SICNoticias
Romania	Digi24_HD
Romania	HotNews_ro
Romania	libertatea
Romania	romania_insider
Slovakia	Pravdask
Slovenia	24ur_com
Slovenia	Delo
Slovenia	Dnevnik_si
Slovenia	rtvslo
Slovenia	STA_English
Slovenia	vecer
Spain	abc_es
Spain	el_pais
Spain	elconfidencial
Spain	eldiarioes
Spain	elmundoes
Spain	elperiodico
Spain	larazon_es
Spain	LaVanguardia
Spain	rtve
Switzerland	24heuresch
Switzerland	Blickch
Switzerland	NZZ
Switzerland	RTSinfo
Switzerland	srfnews
Switzerland	swissinfo_en
Switzerland	tagesanzeiger
Türkiye	anadoluagency
Türkiye	cumhuriyetgzt
Türkiye	gazetesozcu

Türkiye	Hurriyet
Türkiye	milliyet
Türkiye	t24comtr
Türkiye	yenisafak
UK	BBCNews
UK	Channel4News
UK	DailyMailUK
UK	DailyMirror
UK	FT
UK	guardian
UK	Independent
UK	itvnews
UK	SkyNews
UK	Telegraph
UK	TheSun
UK	thetimes

Box 5.4 Health Specific keywords from an official EEA glossary term for each language.

aerobic condition
aerobic process
AIDS
allergen
allergy
alveolus
anatomy
antibiotic
antibody
birth control
blood
breast milk
calcium
cancer
cancer risk
cardiology
cardiovascular disease
cardiovascular system
cell
Chagas' disease
cleansing
clinical symptom
cytotoxicity
decontamination
detoxification
dialysis
disease
disease cause
disinfection

dosage
dose
drug abuse
drug
effect on health
elderly person
endocrine system
endocrinology
enterovirus
environmental health
environmental health hazard
environmental health impact assessment
environmental health protection
environmental health risks
environmental medicine
epidemic
epidemiology
germ
haematology
health
health care
health-care activities waste
health care profession
health effect of noise
health-environment relationship
health facility
health hazard
health legislation
health protection

health regulation
health-related biotechnology
health risks
health service
hearing acuity
hearing impairment
hearing procedure
hearing protection
hearing
hearing system
hospital
human biology
human body
human disease
human health
human pathology
human physiology
human well-being
hygiene
immunological disease
immunology
impregnation
industrial medicine
infant mortality
infection
infectious disease
injury
insurance
intoxication
laboratory test
leukaemia
lymphatic system
malaria
malnutrition
medical science
medicinal plant
medicine
mental effect
metabolism
mortality
muscular system
nausea
necrosis

nervous system
neurotoxicity
nutrition
nutritive value of food
occupational disease
occupational health
occupational health care
occupational medicine
occupational safety
occupational safety regulation
onchocerciasis
oncology
organ
orphan disease
oxygen deficiency
pandemics
pathology
pharmaceutical industry
pharmacokinetics
pharmacology
physical treatment
physiological change
physiology
poison
poisoning
prescription
preventive health measure
primary treatment
psychic effect
psychological effect
psychological stress
psychology
psychosomatic effect
psychosomatic illness
public health
pulmonary disease
radiation sickness
rapid test
recreation
rehabilitation
respiration
respiratory disease
respiratory system

respiratory tract
salmonella
seizure
self-help programme
self-monitoring
side effect
side effects of pharmaceutical drugs
sleep disturbance
social welfare
spasmodic croup
stress
survival
teratogen
teratogenesis
teratogenesis screening
teratogenicity
teratogenic substance
therapy
toxic effect
toxicity
toxicology
tumour
ultrasound
virology
virus
vitamin
work accident
X-ray

Box 5.5 List of excluded keywords from an official EEA glossary terms for each language.

English: accident, acid, act, additive, adhesive, administration, administrative body, administrative boundary, administrative competence, administrative court (administration), administrative deed, administrative fiat, administrative instructions, administrative jurisdiction, administrative law, administrative occupation, administrative organisation, administrative procedure, administrative sanction, adult, adult education, advertisement, advice, africa, age, agreement (administrative), agreement (contract), agreement (legal), agriculture, air, airport, alarm, alcohol, alignment, allocation, allocation plan, allowance, altitude, americas, analysis, analysis programme, angling, animal, appeal, approach, approval, archaeology, architecture, art, ash, asia, assay, atlas, atmosphere, attribute, attribution, audit, authorisation, authority body, banking, bank (land), barents sea, base (chemical), battery, bay, beach, bee, beetle, behaviour, beverage, bibliography, biology, bird, black sea, boiler, book, border, breeding, brick, bridge, brook, budget, budget policy, bug, building, built-up area, bureaucratisation, bus, business, bus station, butterfly, by-catch, by-product, cable, calculation, calibration, camp, camping, canal, car, caribbean area, caspian sea, catalysis, catalyst, catastrophe, cattle, cave, cell (energy), cellulose, census survey, central africa, central america, central asia, central government, centralisation, channelling, chart (act), chart (nautical), chemical, chemistry, chestnut, child, chimney, church, citizen, city, city centre, cliff, climate action bonds, cloud, coast, coating, code, coke, cold, colour, communication, commuting, comparison, compensation, competition (biological), compression, compressor, concession, conductivity, conflict, congress, conservation, consultancy, consultation, consumption, container, continent, contract, convention, cork, corridor, cost, country lodge, county, court, court of justice, court of justice of the european communities, cove, covering, craft, credit, credit assistance, credit policy, creek, crime, criminality, criminal law, crocodile, cruising, cultivation, culture (society), curriculum, customs, cutting (forestry), cutting (vegetative propagation), dairy product, dam, damage, dating, debt, decision, decomposition, decree, deer, defence, degradation, delta, demand, democracy, demography, density, deposition, deregulation, desert, design (project), detection, detector, detergent, deterrence, deterrent, devolution, dictionary, diffusion, digester, digestion (sewage), directive, disaster, disinvestment, dispersion, dissolution, distribution, ditch, doctrine (law), document, documentary film, documentation, dog, draining, drawing, drilling, drying, dumping, dust, dye, dyke, east africa, eastern asia, eastern europe, east-west relations, east-west trade, ec council of ministers, economics, economy, ec policy, ec regulation, ec treaty, education, educational institution, educational path, educational planning, educational system, education policy, effect, egg, elasticity, electronics, emancipation, employment, encyclopaedia, enforcement, engine, engineering, enrichment, equipment, ethics, ethnology, ethology, europe, european court of justice, european monetary fund, european monetary system, european parliament, european standard, european union, evaluation, evaluation criterion, evaluation method, evaluation of technology, evolution, exceptional tax, exchange policy, excise, executive order, exhibit, expenditure, experiment, experimental study, expert system, exploration, explosion, explosive, export, export licence, exposure, expropriation, externality, extraction, fabric, factor market, fallout, family, family law, farm, fault, federal authority, federal government, federal law, fee, feedback loop, fen, field, field experiment, field study, film, filter, finances, financial aid, financial assistance, financial compensation, financial contribution, financial fund, financial instrument, financial law, financial management, financial market, financing, fine, fire, firing, firm, fish, fitting (plumbing), flavouring, flea, flow, fog, food, forecast, forecasting, foreign economic relations, foreign policy, foreign trade, foresight, fountain, freedom, free movement of capital, free trade, frog, frost, fruit, fur, furniture, game (animals), game (play), garden, gas, gender issue, geography, glacier, glass, glaze, glossary, glue, goal of individual economic business, golf, goods, goods and services, government advisory body, government building, government (cabinet), government contracting, government liability, government policy, grain, grass, gridding, grinding, gross domestic product, gross national product, group behaviour, gulf, hail, handicraft, harbour, hardness, harvest, hazard, hazard area, haze, highway, hiking trail, hill, historical evolution, historical monument, historical research, historical site, historic centre, history, holiday, holiday camp, home garden, homepage, horse, hotel industry, hot water, housing, housing density, housing finance, housing improvement, housing legislation, housing need, housing programme, housing quality standard, humanitarian aid, hunting, ice, ideology, image classification, image enhancement, image filtering, image processing, immune system, immunity, immunoassay, impact assessment, impact minimisation, impactor, impact prevention, impact reversal, impact source, implementation law, import, import licence, impoverishment, income, income tax, incorporation, indefinite legal concept, index, indexing of documentation, indian ocean, indicator, indicator-based assessment, industrial policy, industry, infant, infiltration, informatics, information, information centre, information clearing-house, information exchange, information infrastructure, information kit, information network, information processing, information service, information source, information system, information technology, information technology industry, information transfer, infrastructure, infrastructure for spatial information in europe, inhabitant, initial training, ink, inner city, innovation, insect, inspection, inspection of records, inspection service, institutional activity, institutionalisation, institutional structure, instrumentation, insurance business, insurance coverage, interest, interest group, interim decision, interlaboratory comparison, inter-library loan, intermediate goods, intermediate product, internal european market,

internal migration, international agreement, international assistance, international balance, international competitiveness, international conflict, international convention, international co-operation, international court of justice, international distribution, international division of labour, international economic law, international environmental relations, international harmonisation, international law, internationally important ecosystem, international monetary fund, international organisation, international politics, international relations, international river basin, international safety, international standardisation, international trade, international transaction, international watercourse, internet, internet search service, internet service provider, interpolation, interpretation method, intervention fund, inventory, inversion, investment, ion, island, joint implementation (rio conference), judgement (sentence), judicial assistance, judicial body, judicial system, judiciary rule, juridical act, jurisdiction, jurisprudence, justice, labelling, laboratory, laboratory experiment, laboratory research, laboratory technique, laboratory waste, labour, labour force, labour law, labour market, labour relations, lake, lamp, land, landscape, laser, latitude, laundering, law amendment, law branch, law (corpus of rules), law draft, law enforcement, law (individual), law relating to prisons, law (science), lead, leaf, leakage, lease, leather, legal basis, legal form of organisations, legally protected right, legal procedure, legal profession, legal regulation, legal remedy, legal system, legal text, legislation, legislative authority, legislative competence, legislative information, legislative procedure, legislative process, legislature, leisure activity, leisure centre, leisure time, less developed country, level of education, lexicon, liability, liability legislation, library, library service, licencing, licencing obligation, licencing procedure, life cycle, life-cycle management, lifestyle, light, lighting, lime, limit value, line, linear economy, literature, literature data bank, literature evaluation, literature study, litigation, litter, lizard, local authority, local building material, local development, local finance, local government, local government policy, local passenger service, local recreation, local traffic, location of industries, locomotive, lodging, longitude, long-term effect, long-term experiment, long-term transition, long-term trend, loss, lower house, machinery, macroeconomic goal, macroeconomics, mailing list, maintenance (technical), major accident, major risk, major risk installation, mammal, management, management accounting, management contract, management plan, management technique, mandate, manpower, man (society), manufacturing activity, manufacturing trade, manure, map, map chart, mapping, marble, marina, marine strategy framework directive, marital status, maritime law, maritime navigation, maritime transport, marker, market, market economy, market form, market gardening, marketing, market price, market research, market study, mass media, mass recreation, mass transport (physics), material, measuring, measuring instrument, measuring method, measuring programme, meat, mediterranean area, mediterranean forest, mediterranean sea, mediterranean wood, melting, mercury, metal, meteorology, method, methodology, metropolis, microwave, migrant labour, military activities, military air traffic, military aspects, military equipment, military zone, milk, mill, mine, mineral, ministry, ministry building, minority, miscellaneous product, mist, mite, mixed farming, mixing, mobile home, model, modelling, mode of transportation, moisture, monetary assessment, monetary economics, monetary relations, money market, monitoring, monitoring criterion, monitoring data, monitoring equipment, monitoring network, monitoring station, monitoring system, monitoring technique, monopoly, monument, morphology, mosaic, motorcycle, motor vehicle, motorway, mountain, mountain range, mountain refuge, mountain resort, mowing, mud (sediment), multilateral agreement, multimedia technology, multinational firm, multiple use management area, multispectral scanner, municipality, municipal law, municipal level, museum, mushroom, music, mutant, mutation, myth, national accounting, national boundary, national conservation programme, national economic costs, national economy, nationalisation, national legislation, national park, national planning, national reserve, natural area, natural capital, natural capital accounts, natural material, natural monument, natural resource, natural resource conservation, natural risk, natural risk analysis, natural risks prevention, natural scenery, natural science, natural stone, natural value, nature-based solution, navigation, navigational hazard, need, negotiable charge, neighbourhood improvement scheme, neighbourhood law, neighbourhood noise, nesting, nesting area, net resource depletion, netting policy (emissions trading), neutralisation, new community, new installation, new material, newsgroup, newsletter, new technology, new town, noise, noise level, noise map, noise measurement, noise monitoring, noise type, nomenclature, non-metal, non-residential building, norm, normalisation, north africa, north america, north atlantic ocean, north pacific ocean, north-south relationship, notice, notification, nuisance, nursery garden, nursery (plant breeding), nutrient, oak, objection, objective well-being, obligation to inform, obligation to label, observation satellite, occupation, occupational group, occupational status, ocean, oceania, odonate, odour, office, official duty, official hearing, offset policy (emissions trading), off-site, open sea, operating data, opinion, opinion survey, order, ordinance, ore, organisation (law), organisation of teaching, organisation of the legal system, organisation of work, organism, oven, overburden, overconsumption, overcrowding, overturn (limnology), ownership, packaging, paint, painting business, paint room, paint shop, paper, parameter, parliament, parliamentary debate, parliamentary report, participation, patent, path, peat, pedagogy, penalty, permission, pest, pet, philosophy, photograph, photography, physical planning, physical process, physical property, physical science, physics, pipe, pipeline, pixel, plain, plan, planning law, planning measure, planning permission, planning-programming-budgeting system, plant (biology), planting, playground, point, police, police law, police power, policy, policy effectiveness, policy framework, policy guideline, policy implementation, policy instrument, policy integration, policy planning, political counselling, political doctrine, political geography, political ideology, political organisation, political party, political power, politics, polygon, pond, pool, post-treatment, poverty, power company, predator, premium, preserve, press, pressing, press release, pressure, price, prices policy, primary sector, primate, printing work, prior informed consent, private car, private domain, private international law, private law, private sector, private transport, privatisation, procedural law, process analysis, processing, product, product advertising, product comparison, product evaluation, product identification, product information, production policy, productivity, productivity trend, product liability, product life cycle, product standard, professional society, profit, prognostic data, programme, progress line, prohibition, project, promotion of trade and industry, propagation process, property protection, prosecution, prosperity, protocol, protozoan, province, provincial/regional authority (d), provincial/regional law (d), public, public action, public aid, public attendance, public bath, public building, public contract, public debt, public discussion, public domain, public emergency limit, public expenditure, public finance, public financing, public function, public hearing, public information, public inquiry, public institution, public international law, public law, public maritime domain, public opinion, public opinion polling, public park, public participation, public-private partnership, public procurement, public prosecutor's office, public relations, public sector, public service, public utility, public works, pulp, pump, pumping, purchase, quality assurance, quality certification, quality control, quality objective, quality of life, quality standard, racking, radar, radio, radio programme, rag, railway, rain, rape (plant), raster, rate, reactor, reasonableness, recommendation, redress, reed, reef, reference service, referral information, referral information system, reflection, refrigerator, refuge, refugee, region, registration, regulation, reintroduction, relational database, relief (land), religion, removal, rental housing, repair business, replacement, replacement cost, reporting process, report to the minister, representation, repression, reprocessing, reproduction (biological), reptile, rescue service, rescue system, research, research centre, research of the effects, research policy, research project, reserve, residential area, residential area with traffic calmings, residential building, residual risk, resilience, resin, resistance (biological), resolution (act), resolution (parameter), resource, resource use, resource utilisation, responsibility, resting form, restoration, retail trade, rice, right of access, right of property, rights, rights of future generations, rights of the individual, right to compensation, right to information, ringing (wildlife), rinsing, rising (geological), risk, risk analysis, risk assessment, risk-based change, risk-benefit analysis, risk communication, risk exposure, risk exposure plan, risk management, risk perception, risk reduction, river, road, rock, rodent, root, route, route planning, rubber, ruling, running wild, runoff, safety, safety analysis, safety measure, safety rule, safety standard, safety study, safety system, salamander, salt, salvage, sampling, sampling technique, sanction, sand, sanitation, satellite, satellite account, satellite image, saving, school, school life, school teaching, schoolwork, science, scientific and technical information, scientific committee, scientific co-operation, scientific dispute, scientific policy, scientific research, scoping

procedure, sea, seagrass, sealing, seal (technical), season, secondary education, secondary sector, second-hand goods, sectoral assessment, security of installations, sediment, seed (biology), seed (product), seizure of profits, selection of technology, sensitive area, sensitivity analysis, sensor, separation, separator, service area, services, sewage, shellfish, shelter, ship, shop, shopping centre, show, shredder, shrub, silver, sizing, skiing, skin, slag, sleep, slope, sludge, small islands (political geography), smoke, smoking, snake, snow, snowslide, soaking, soap, social analysis, social behaviour, social bond, social cohesion, social condition, social cost, social development, social differentiation, social dynamics, social equity, social facility, social framework, social group, social indicator, social inequality, social medicine, social-minded behaviour, social movement, social participation, social policy, social problem, social process, social protection, social psychology, social relief, social representation, social science, social security, social service, social structure, social survey, social system, social value, society, socio-cultural group, socioeconomic factor, socioeconomics, socioeducational activity, sociological survey, sociology, sociopolitical aspect, softening, software, software development, soil, sound, sound level, sound measurement, soundproofing, south america, south atlantic ocean, southeast asia, southern africa, southern asia, south pacific ocean, space (interplanetary), space policy, space research, space transportation, space travel, spatial distribution, spatial mobility, special authorisation, specialisation (biological), special law, species, speech, speed, speed limit, spider, spillage, sponsorship, sport, sports facility, spring (hydrology, land), square, stable, stack, standard, standardisation, starch, state, state control, state of matter, state of the art, station, statistical analysis, statistical data, statistical information, statistical information system, statistical series, statistics, status of development, statutory declaration, statutory public body, statutory text, steel, sterilisation (biological), sterilisation (process), steroid, stock (biological), stocking, stock management, stocktaking, stock (trade), stone, storage (process), stove, stratification, subject, submarine, subsequent order, subsidence, subsidiary principle, subsidy, substitutability (chemistry), suburb, sugar industry, sugar (product), supervision of building works, supervision of installation, supervisory body, supply and demand, supply (trade), surplus, surveillance, survey, suspended matter, swamp, swans, geese and ducks, sweetener, swell, taking of evidence, tanker (ship), tanker (truck), tar, target group, target setting, tariff, tax, taxation, taxation policy, tax differentiation, tax law, tax on capital, tax on consumption, taxonomy, taxonomy regulation, tax system, teaching, teaching material, teaching method, technical information, technical instruction, technical regulation, technological accident, technological change, technological development, technological hazard, technological process, technology, technology acceptance, technology assessment, technology transfer, telecommunication, teleheating, telematics, telemetry, television, television programme, terminology, termite, territorial community, territorial government, territorial policy, territory, test, test animal, testing guideline, testing method, test organism, textile, theory of money, theory of the welfare state, thermodynamics, thesaurus, thesis, thickening, threshold value, thunderstorm, tide, timber, time, time allocation, time budget, time-horizon mismatch, tin (element), tissue, tobacco, tobacco smoke, tornado, total parameter, tourism, tourist attendance, tourist facility, touristic activity management, touristic route, touristic unit, touristic zone, tradeable permit, trade activity, trade and consumption, trade barrier, trade (economic), trade impact on environment, trade policy, trade (profession), trade relations, trade restriction, trade (services), trades union, traditional culture, traditional health care, traffic, traffic accident, traffic control, traffic control measure, traffic engineering, traffic infrastructure, traffic jam, traffic monitoring, traffic on water, traffic regulation, traffic route, traffic route construction, train, training, training centre, trajectory, transition, transitional arrangement, transitional settlement, transition element, transportation, transportation business, transportation by pipeline, transportation mean, transportation policy, transport cost, transport law, transport (physics), transport planning, transport regulation, transport system, transposition of directive, travel, travel cost, treaty, tree, trend, trend of opinion, trial, tropics, tunnel, turbine, type of business, type of claim, type of management, type of tenure, united nations, unsupervised image classification, upper house, urban design, urban development, urban development document, urban development law, urban facility, urban flows (resources), urban settlement, valley, vandalism, variety collection, varnish, vector, vector of human diseases, vector to raster, vegetable, vehicle, ventilation, vermin, vibration, video, village, vocabulary, vocational training, volatility, voluntary work, voting, wadden sea, wage system, wall, war, warning system, war victim, waterfall, water (geographic), water (substance), weapon, weather, weed, weight, welding, well, west africa, western asia, western europe, whale, wind, windfall, winter sports resort, woman, woman's status, wood, wool, working condition, working hours, workplace, world, world health organization, world heritage site, world wide web, wreck, write-off, wrongful act, wrongful government act, yeast, yield (agricultural), yield (economy), young, youth, youth work, zoology
**Bulgarian**: all terms from English language translated according to EEA, and additionally - Бор, Тор, Мед
**German**: all terms from English language translated according to EEA, and additionally - Gen, Russ
**Estonian**: all terms from English language translated according to EEA, and additionally - rt, bor, dina
**Finnish**: all terms from English language translated according to EEA, and additionally - suo
**French**: all terms from English language translated according to EEA, and additionally - pré, fer, cation, cap
**Icelandic**: all terms from English language translated according to EEA, and additionally - ál, bú
**Italian**: all terms from English language translated according to EEA, and additionally - lega
**Latvian**: all terms from English language translated according to EEA, and additionally - aka
**Norwegian**: all terms from English language translated according to EEA, and additionally - for, sel, eng, ef, bor, nes
**Polish**: all terms from English language translated according to EEA, and additionally - bor, bar, tal, rak, las
**Romanian**: all terms from English language translated according to EEA, and additionally - bor, lac, zer, iod
**Slovakia**: all terms from English language translated according to EEA, and additionally - íl, jed, lom, les, mys, lak, pec
**Slovenian**: all terms from English language translated according to EEA, and additionally - rt, bor, rak, eter, gen, lak, pes, talij, dim, kolo
**Turkish**: all terms from English language translated according to EEA, and additionally - ısı, yağ, is, kil, gen, bor, eter, alg

Inequality Context

We used the following translations of the inequality key terms (see **box 5.6**).

**English:** "inequality", "inequity", "injustice", "justice", "equity", "equality"
**Bulgarian:** "неравенство", "несправедливост", "несправедливост", "справедливост", "справедливост", "равенство"
**Croatian:** "nejednakost", "nejednakost", "nepravda", "pravda", "pravičnost", "jednakost"
**Czech:** "nerovnost", "nespravedlnost", "nespravedlnost", "spravedlnost", "spravedlnost", "rovnost"
**Estonia:** "ebavõrdsus", "ebavõrdsus", "ebaõiglus", "õiglus", "võrdsus", "võrdsus"
**French:** "inégalité", "inéquité", "injustice", "justice", "équité", "égalité"
**Finnish:** "epätasa-arvo", "epäoikeudenmukaisuus", "epäoikeudenmukaisuus", "oikeudenmukaisuus", "oikeudenmukaisuus", "tasa-arvo"
**German:** "ungleichheit", "ungerechtigkeit", "gerechtigkeit", "gleichheit"

**Hungarian:** "egyenlőtlenség", "egyenlőtlenség", "igazságtalanság", "igazságosság", "méltányosság", "egyenlőség"
**Icelandic:** "ójöfnuður", "misrétti", "óréttlæti", "réttlæti", "jafnrétti", "jafnrétti"
**Italian:** "disuguaglianza", "iniquità", "ingiustizia", "giustizia", "equità", "uguaglianza"
**Latvian:** "nevienlīdzība", "nevienlīdzība", "netaisnība", "taisnīgums", "vienlīdzība", "vienlīdzība"
**Norwegian:** "ulikhet", "ulikhet", "urettferdighet", "rettferdighet", "likhet", "likhet"
**Polish:** „nierówność", „nierówność", „niesprawiedliwość", „sprawiedliwość", „równość", „równość"
**Portuguese:** "desigualdade", "injustiça", "justiça", "equidade", "igualdade"
**Romanian:** „inegalitate", „inechitate", „nedreptate", „dreptate", „echitate", „egalitate"
**Slovak:** "nerovnosť", "nerovnosť", "nespravodlivosť", "spravodlivosť", "rovnosť", "rovnosť"
**Slovenian:** "neenakost", "nepravičnost", "nepravičnost", "pravičnost", "pravičnost", "enakost"
**Spanish:** "desigualdad", "inequidad", "injusticia", "justicia", "equidad", "igualdad"
**Turkish:** "eşitsizlik", "eşitsizlik", "haksızlık", "adalet", "eşitlik", "eşitlik"

References

1. Countries — European Environment Agency. Accessed March 19, 2022. https://www.eea.europa.eu/countries-and-regions
2. WHO Europe | Countries. Accessed March 19, 2022. https://www.euro.who.int/en/countries
3. Country profiles European Union . Accessed March 19, 2022. https://european-union.europa.eu/principles-countries-history/country-profiles_en
4. Negotiations status. Accessed March 19, 2022. https://ec.europa.eu/neighbourhood-enlargement/enlargement-policy/negotiations-status_en
5. The EFTA States | European Free Trade Association. Accessed March 19, 2022. https://www.efta.int/about-efta/the-efta-states
6. Global Burden of Disease Study 2019 (GBD 2019) Population Estimates 1950-2019 | GHDx. Accessed March 18, 2022. https://ghdx.healthdata.org/record/ihme-data/gbd-2019-population-estimates-1950-2019
7. Global Burden of Disease Collaborative Network. Global Burden of Disease Study 2019 (GBD 2019) Results. Seattle, United States: Institute for Health Metrics and Evaluation (IHME). Published 2020. Accessed March 18, 2022. https://ghdx.healthdata.org/gbd-results-tool.
8. World Bank. United Nations Population Division's World Urbanization Prospects: 2018 Revision. Accessed March 18, 2022. https://data.worldbank.org/indicator/SP.URB.TOTL
9. Chambers J. Global and cross-country analysis of exposure of vulnerable populations to heatwaves from 1980 to 2018. *Clim Change*. 2020;163(1):539-558. doi:10.1007/S10584-020-02884-2/FIGURES/8
10. ERA5-Land hourly data from 1950 to present. Accessed March 8, 2022. https://cds.climate.copernicus.eu/cdsapp#!/dataset/reanalysis-era5-land?tab=overview
11. GEOSTAT - GISCO - Eurostat. Accessed December 3, 2021. https://ec.europa.eu/eurostat/web/gisco/geodata/reference-data/population-distribution-demography/geostat
12. Hernangómez D. giscoR: Download Map Data from GISCO API - Eurostat. doi:10.5281/ZENODO.4317947
13. Jay O. *Extreme Heat Policy*.; 2021.
14. Beggs PJ, Zhang Y, McGushin A, et al. The 2021 report of the MJA–Lancet Countdown on health and climate change: Australia increasingly out on a limb. *Med J Aust*. 2021;215(9):390-392.e22. doi:10.5694/MJA2.51302
15. Sydney research informs Sports Medicine Australia's new heat policy - The University of Sydney. Accessed November 10, 2023. https://www.sydney.edu.au/news-opinion/news/2021/02/24/sydney-research-informs-sports-medicine-australia-s-new-heat-pol.html
16. Romanello M, Di Napoli C, Drummond P, et al. The 2022 report of the Lancet Countdown on health and climate change: health at the mercy of fossil fuels. *Lancet*. 2022;400(10363):1619-1654. doi:10.1016/S0140-6736(22)01540-9/ATTACHMENT/D63703F8-315E-4CDB-9573-1E552E1D4913/MMC5.PDF
17. Barthelme S. imager: Image Processing Library Based on 'CImg.' Published online 2021.
18. May RM, Arms SC, Marsh P. MetPy: A Python Package for Meteorological Data. Published 2022.

Accessed March 18, 2022. doi:doi:10.5065/D6WW7G29
19. GISCO - Eurostat. Published 2022. Accessed March 18, 2022. https://ec.europa.eu/eurostat/web/gisco
20. Fan Y, Wang J, Obradovich N, Zheng S. Intraday adaptation to extreme temperatures in outdoor activity. *Sci Rep*. 2023;13(1):473. doi:10.1038/S41598-022-26928-Y
21. Nikitara K, Odani S, Demenagas N, Rachiotis G, Symvoulakis E, Vardavas C. Prevalence and correlates of physical inactivity in adults across 28 European countries. *Eur J Public Health*. 2021;31(4):840-845. doi:10.1093/EURPUB/CKAB067
22. Phelan JC, Link BG, Tehranifar P. Social Conditions as Fundamental Causes of Health Inequalities: Theory, Evidence, and Policy Implications. *http://dx.doi.org/101177/0022146510383498*. 2010;51(1_suppl):S28-S40. doi:10.1177/0022146510383498
23. Weekly death statistics - Eurostat. Accessed March 14, 2022. https://ec.europa.eu/eurostat/statistics-explained/index.php?title=Weekly_death_statistics&stable
24. Muñoz-Sabater J, Dutra E, Agustí-Panareda A, et al. ERA5-Land: A state-of-the-art global reanalysis dataset for land applications. *Earth Syst Sci Data*. 2021;13(9):4349-4383. doi:10.5194/ESSD-13-4349-2021
25. Eurostat. Regional demographic statistics. Accessed March 18, 2022. https://ec.europa.eu/eurostat/data/database?node_code=demo_r_pjangrp3
26. Library WO, Gasparrini A. Modeling exposure–lag–response associations with distributed lag non-linear models. *Stat Med*. 2014;33(5):881-899. doi:10.1002/SIM.5963
27. Martínez-Solanas È, Quijal-Zamorano M, Achebak H, et al. Projections of temperature-attributable mortality in Europe: a time series analysis of 147 contiguous regions in 16 countries. *Lancet Planet Heal*. 2021;5(7):e446-e454. doi:10.1016/S2542-5196(21)00150-9/ATTACHMENT/CF8FBC2A-0B57-4F32-99B4-3395CAE5A955/MMC1.PDF
28. Ballester J, van Daalen KR, Chen Z, et al. Effect of temporal data aggregation in timeseries epidemiological studies of temperature related mortality. *Lancet Reg Heal - Eur*.
29. Sera F, Armstrong B, Blangiardo M, Gasparrini A. An extended mixed-effects framework for meta-analysis. *Stat Med*. 2019;38(29):5429-5444. doi:10.1002/SIM.8362
30. Gasparrini A, Leone M. Attributable risk from distributed lag models. *BMC Med Res Methodol*. 2014;14(1):1-8. doi:10.1186/1471-2288-14-55/FIGURES/3
31. Vicedo-Cabrera AM, Sera F, Gasparrini A. Hands-on Tutorial on a Modeling Framework for Projections of Climate Change Impacts on Health. *Epidemiology*. 2019;30(3):321-329. doi:10.1097/EDE.0000000000000982
32. Liu C, Yavar Z, Sun Q. Cardiovascular response to thermoregulatory challenges. *Am J Physiol - Hear Circ Physiol*. 2015;309(11):H1793-H1812. doi:10.1152/AJPHEART.00199.2015/ASSET/IMAGES/LARGE/ZH40211517280004.JPEG
33. Achebak H, Devolder D, Ballester J. Heat-related mortality trends under recent climate warming in Spain: A 36-year observational study. *PLOS Med*. 2018;15(7):e1002617. doi:10.1371/JOURNAL.PMED.1002617
34. Saucy A, Ragettli MS, Vienneau D, et al. The role of extreme temperature in cause-specific acute cardiovascular mortality in Switzerland: A case-crossover study. *Sci Total Environ*. 2021;790:147958.

doi:10.1016/J.SCITOTENV.2021.147958
35. Fouillet A, Rey G, Laurent F, et al. Excess mortality related to the August 2003 heat wave in France. *Int Arch Occup Environ Health*. 2006;80(1):16. doi:10.1007/S00420-006-0089-4
36. Conte Keivabu R. Extreme Temperature and Mortality by Educational Attainment in Spain, 2012–2018. *Eur J Popul*. 2022;38(5):1145-1182. doi:10.1007/S10680-022-09641-4/TABLES/13
37. ISGlobal. EARLY-ADAPT. doi:https://early-adapt.eu.
38. Weekly death statistics - Eurostat. Accessed March 18, 2022. https://ec.europa.eu/eurostat/statistics-explained/index.php?title=Weekly_death_statistics&stable
39. NOAA. NOAA Merged Land Ocean Global Surface Temperature Analysis (NOAAGlobalTemp). 2023. https://www.ncei.noaa.gov/products/land-based-station/noaa-global-temp
40. Ballester J, Quijal-Zamorano M, Fernando R, et al. Heat-related mortality in Europe during the summer of 2022. *Nat Med 2023 297*. 2023;29(7):1857-1866. doi:10.1038/s41591-023-02419-z
41. Gasparrini A, Armstrong B, Kenward MG. Multivariate meta-analysis for non-linear and other multi-parameter associations. *Stat Med*. 2012;31(29):3821-3839. doi:10.1002/SIM.5471
42. Philip S, Kew S, van Oldenborgh GJ, et al. A protocol for probabilistic extreme event attribution analyses. *Adv Stat Climatol Meteorol Oceanogr*. 2020;6(2):177-203. doi:10.5194/ASCMO-6-177-2020
43. Semenza JC, Rubin CH, Falter KH, et al. Heat-Related Deaths during the July 1995 Heat Wave in Chicago. *N Engl J Med*. 1996;335(2):84-90. doi:10.1056/nejm199607113350203
44. Early-Adapt. Signs of Early Adaptation to Climate Change.
45. Orellano P, Reynoso J, Quaranta N, Bardach A, Ciapponi A. Short-term exposure to particulate matter (PM10 and PM2.5), nitrogen dioxide (NO2), and ozone (O3) and all-cause and cause-specific mortality: Systematic review and meta-analysis. *Environ Int*. 2020;142:105876. doi:10.1016/J.ENVINT.2020.105876
46. Sofiev M, Vankevich R, Lotjonen M, et al. An operational system for the assimilation of the satellite information on wild-land fires for the needs of air quality modelling and forecasting. *Atmos Chem Phys*. 2009;9(18):6833-6847. doi:10.5194/ACP-9-6833-2009
47. Soares J, Sofiev M, Hakkarainen J. Uncertainties of wild-land fires emission in AQMEII phase 2 case study. *Atmos Environ*. 2015;115:361-370. doi:10.1016/J.ATMOSENV.2015.01.068
48. Hänninen R, Sofiev M, Uppstu A, Kouznetsov R. Daily surface concentration of fire related PM2.5 for 2003-2021, modelled by SILAM CTM when using the MODIS satellite data for the fire radiative power [Dataset]. Finnish Meteorological Institute. Published 2022. https://doi.org/10.23728/FMI-B2SHARE.A006840CCE9340E8BF11E562BB8D396E
49. Kollanus V, Prank M, Gens A, et al. Mortality due to Vegetation Fire–Originated PM2.5 Exposure in Europe—Assessment for the Years 2005 and 2008. *Environ Health Perspect*. 2017;125(1):30. doi:10.1289/EHP194
50. Zapata-Diomedi B, Barendregt JJ, Veerman JL. Population attributable fraction: names, types and issues with incorrect interpretation of relative risks. *Br J Sports Med*. 2018;52(4):212-213. doi:10.1136/BJSPORTS-2015-095531
51. Khomenko S, Cirach M, Pereira-Barboza E, et al. Premature mortality due to air pollution in European cities: a health impact assessment. *Lancet Planet Heal*. 2021;5(3):e121-e134. doi:10.1016/S2542-

5196(20)30272-2/ATTACHMENT/EA6D2A19-0992-4ED1-AC46-9FF42EA6DB7B/MMC1.PDF

52. Aguilera R, Corringham T, Gershunov A, Benmarhnia T. Wildfire smoke impacts respiratory health more than fine particles from other sources: observational evidence from Southern California. *Nat Commun 2021 121*. 2021;12(1):1-8. doi:10.1038/s41467-021-21708-0

53. Chen G, Guo Y, Yue X, et al. Mortality risk attributable to wildfire-related PM2·5 pollution: a global time series study in 749 locations. *Lancet Planet Heal*. 2021;5(9):e579-e587. doi:10.1016/S2542-5196(21)00200-X/ATTACHMENT/E841C89B-3E49-4AB5-AFB7-E93EA966CFB8/MMC1.PDF

54. Maier SW, Russell-Smith J, Edwards AC, Yates C. Sensitivity of the MODIS fire detection algorithm (MOD14) in the savanna region of the Northern Territory, Australia. *ISPRS J Photogramm Remote Sens*. 2013;76:11-16. doi:10.1016/J.ISPRSJPRS.2012.11.005

55. EEA. *Use of Freshwater Resources (CSI 018/WAT 001)*.; 2018.

56. Allen RG, Pereira LS, Raes D, Smith M. *Crop Evapotranspiration - Guidelines for Computing Crop Water Requirements - FAO Irrigation and Drainage Paper 56*.; 1998.

57. Vicente-Serrano SM, Beguería S, López-Moreno JI. A Multiscalar Drought Index Sensitive to Global Warming: The Standardized Precipitation Evapotranspiration Index. *J Clim*. 2010;23(7):1696-1718. doi:10.1175/2009JCLI2909.1

58. MeteoSwiss. SPI and SPEI.

59. WMO. *2021 Edition of the State of Climate Services Report (WMO-No. 1278)*.; 2021.

60. Eyring V, Bony S, Meehl GA, et al. Overview of the Coupled Model Intercomparison Project Phase 6 (CMIP6) experimental design and organization. *Geosci Model Dev*. 2016;9(5):1937-1958. doi:10.5194/GMD-9-1937-2016

61. Warszawski L, Frieler K, Huber V, Piontek F, Serdeczny O, Schewe J. The inter-sectoral impact model intercomparison project (ISI-MIP): Project framework. *Proc Natl Acad Sci U S A*. 2014;111(9):3228-3232. doi:10.1073/PNAS.1312330110/ASSET/E112A168-8F78-4223-B2BE-E0553A3C432C/ASSETS/GRAPHIC/PNAS.1312330110I1.GIF

62. Good S, Fiedler E, Mao C, et al. The Current Configuration of the OSTIA System for Operational Production of Foundation Sea Surface Temperature and Ice Concentration Analyses. *Remote Sens 2020, Vol 12, Page 720*. 2020;12(4):720. doi:10.3390/RS12040720

63. Copernicus In Situ - Marine. Accessed November 11, 2023. https://insitu.copernicus.eu/FactSheets/CMEMS/

64. Center For International Earth Science Information Network-CIESIN-Columbia University. Gridded Population of the World, Version 4 (GPWv4): Population Count, Revision 11. 2018. doi:10.7927/H4JW8BX5

65. Baker-Austin C, Trinanes JA, Taylor NGH, Hartnell R, Siitonen A, Martinez-Urtaza J. Emerging Vibrio risk at high latitudes in response to ocean warming. *Nat Clim Chang 2012 31*. 2012;3(1):73-77. doi:10.1038/nclimate1628

66. Martinez-Urtaza J, Van Aerle R, Abanto M, et al. Genomic Variation and Evolution of Vibrio parahaemolyticus ST36 over the Course of a Transcontinental Epidemic Expansion. *MBio*. 2017;8(6):1425-1442. doi:10.1128/MBIO.01425-17

67. Parveen S, Hettiarachchi KA, Bowers JC, et al. Seasonal distribution of total and pathogenic Vibrio

parahaemolyticus in Chesapeake Bay oysters and waters. *Int J Food Microbiol*. 2008;128(2):354-361. doi:10.1016/J.IJFOODMICRO.2008.09.019

68. Baker-Austin C, Oliver JD, Alam M, et al. Vibrio spp. infections. *Nat Rev Dis Prim 2018 41*. 2018;4(1):1-19. doi:10.1038/s41572-018-0005-8
69. Newton A, Kendall M, Vugia DJ, Henao OL, Mahon BE. Increasing Rates of Vibriosis in the United States, 1996–2010: Review of Surveillance Data From 2 Systems. *Clin Infect Dis*. 2012;54(0 5):S391. doi:10.1093/CID/CIS243
70. Ralston EP, Kite-Powell H, Beet A. An estimate of the cost of acute food and water borne health effects from marine pathogens and toxins in the United States. *J Water Health*. 2011;9(4):680. doi:10.2166/WH.2011.157
71. Hersbach H, Bell B, Berrisford P, et al. The ERA5 global reanalysis. *Q J R Meteorol Soc*. 2020;146(730):1999-2049. doi:10.1002/QJ.3803
72. Kriticos DJ, Jarošik V, Ota N. Extending the suite of bioclim variables: a proposed registry system and case study using principal components analysis. *Methods Ecol Evol*. 2014;5(9):956-960. doi:10.1111/2041-210X.12244
73. Farooq Z, Sjödin H, Semenza JC, et al. European projections of West Nile virus transmission under climate change scenarios. *One Heal*. 2023;16:100509. doi:10.1016/J.ONEHLT.2023.100509
74. Homepage | European Centre for Disease Prevention and Control. Accessed November 11, 2023. https://www.ecdc.europa.eu/en
75. Farooq Z, Rocklöv J, Wallin J, et al. Artificial intelligence to predict West Nile virus outbreaks with eco-climatic drivers. *Lancet Reg Heal - Eur*. 2022;17:100370. doi:10.1016/J.LANEPE.2022.100370
76. Chen T, Guestrin C. XGBoost: A Scalable Tree Boosting System. *Proc 22nd ACM SIGKDD Int Conf Knowl Discov Data Min*. doi:10.1145/2939672
77. XGBoost Documentation — xgboost 1.7.6 documentation. https://xgboost.readthedocs.io/en/stable/
78. van Daalen KR, Romanello M, Rocklöv J, et al. The 2022 Europe report of the Lancet Countdown on health and climate change: towards a climate resilient future. *Lancet Public Heal*. 2022;7(11):e942-e965. doi:10.1016/S2468-2667(22)00197-9/ATTACHMENT/817725A3-ED43-419E-8610-C286BFD45546/MMC1.PDF
79. Chambers J. Hybrid gridded demographic data for the world, 1950-2020. Published online April 27, 2020. doi:10.5281/ZENODO.3768003
80. DiSera L, Sjödin H, Rocklöv J, et al. The Mosquito, the Virus, the Climate: An Unforeseen Réunion in 2018. *GeoHealth*. 2020;4(8). doi:10.1029/2020GH000253
81. Colón-González FJ, Sewe MO, Tompkins AM, et al. Projecting the risk of mosquito-borne diseases in a warmer and more populated world: a multi-model, multi-scenario intercomparison modelling study. *Lancet Planet Heal*. 2021;5(7):e404-e414. doi:10.1016/S2542-5196(21)00132-7/ATTACHMENT/F6794FC7-A9E6-410F-B0FB-86D5C90BA907/MMC1.PDF
82. Liu-Helmersson J, Brännström Å, Sewe MO, Semenza JC, Rocklöv J. Estimating past, present, and future trends in the global distribution and abundance of the arbovirus vector Aedes aegypti under climate change scenarios. *Front Public Heal*. 2019;7(JUN):148. doi:10.3389/FPUBH.2019.00148/BIBTEX

83. Rocklöv J, Quam MB, Sudre B, et al. Assessing Seasonal Risks for the Introduction and Mosquito-borne Spread of Zika Virus in Europe. *eBioMedicine*. 2016;9:250-256. doi:10.1016/J.EBIOM.2016.06.009
84. Clark JS. Models for ecological data : an introduction. Published online 2007:617.
85. Johnson LR, Ben-Horin T, Lafferty KD, et al. Understanding uncertainty in temperature effects on vector-borne disease: a Bayesian approach. *Ecology*. 2015;96(1):203-213. doi:10.1890/13-1964.1
86. Rocklöv J, Tozan Y. Climate change and the rising infectiousness of dengue. *Emerg Top Life Sci*. 2019;3(2):133. doi:10.1042/ETLS20180123
87. Goldewijk KK, Beusen A, Doelman J, Stehfest E. Anthropogenic land use estimates for the Holocene - HYDE 3.2. *Earth Syst Sci Data*. 2017;9(2):927-953. doi:10.5194/ESSD-9-927-2017
88. Liu-Helmersson J, Stenlund H, Wilder-Smith A, Rocklöv J. Vectorial Capacity of Aedes aegypti: Effects of Temperature and Implications for Global Dengue Epidemic Potential. *PLoS One*. 2014;9(3). doi:10.1371/JOURNAL.PONE.0089783
89. Rocklöv J, Tozan Y, Ramadona A, et al. Using Big Data to Monitor the Introduction and Spread of Chikungunya, Europe, 2017 . *Emerg Infect Dis*. 2019;25(6):1041-1049. doi:10.3201/EID2506.180138
90. Ramadona AL, Tozan Y, Lazuardi L, Rocklöv J. A combination of incidence data and mobility proxies from social media predicts the intra-urban spread of dengue in Yogyakarta, Indonesia. *PLoS Negl Trop Dis*. 2019;13(4):e0007298. doi:10.1371/JOURNAL.PNTD.0007298
91. Simini F, González MC, Maritan A, Barabási AL. A universal model for mobility and migration patterns. *Nat 2012 4847392*. 2012;484(7392):96-100. doi:10.1038/nature10856
92. Stefanouli M, Polyzos S. Gravity vs radiation model: two approaches on commuting in Greece. *Transp Res Procedia*. 2017;24:65-72. doi:10.1016/J.TRPRO.2017.05.069
93. Boualam MA, Pradines B, Drancourt M, Barbieri R. Malaria in Europe: A Historical Perspective. *Front Med*. 2021;8:876. doi:10.3389/FMED.2021.691095/BIBTEX
94. Krüger A, Rech A, Su XZ, Tannich E. Two cases of autochthonous Plasmodium falciparum malaria in Germany with evidence for local transmission by indigenous Anopheles plumbeus. *Trop Med Int Heal*. 2001;6(12):983-985. doi:10.1046/J.1365-3156.2001.00816.X
95. Arends JE, Oosterheert JJ, Kraaij-Dirkzwager MM, et al. Two Cases of Plasmodium falciparum Malaria in the Netherlands without Recent Travel to a Malaria-Endemic Country. *Am J Trop Med Hyg*. 2013;89(3):527-530. doi:10.4269/AJTMH.13-0213
96. Alduchov O. Improved Magnus Form Approximation of Saturation Vapor Pressure. *J Appl Meteorol Climatol*. 1996;33(4). Accessed March 18, 2022. https://journals.ametsoc.org/view/journals/apme/35/4/1520-0450_1996_035_0601_imfaos_2_0_co_2.xml
97. Patz JA, Graczyk TK, Geller N, Vittor AY. Effects of environmental change on emerging parasitic diseases. *Int J Parasitol*. 2000;30(12-13):1395-1405. doi:10.1016/S0020-7519(00)00141-7
98. Grover-Kopec EK, Blumenthal MB, Ceccato P, Dinku T, Omumbo JA, Connor SJ. Web-based climate information resources for malaria control in Africa. *Malar J*. 2006;5(1):1-9. doi:10.1186/1475-2875-5-38/FIGURES/5
99. State of inequality HIV, tuberculosis and malaria. Accessed November 11, 2023.

https://www.who.int/data/inequality-monitor/publications/report_2021_hiv_tb_malaria
100. Berriatua E, Maia C, Conceição C, et al. Leishmaniases in the European Union and Neighboring Countries. *Emerg Infect Dis*. 2021;27(6):1723-1727. doi:10.3201/EID2706.210239
101. European Centre for Disease Prevention and Control. Surveillance, prevention and control of leishmaniases in the European Union and its neighbouring countries. Published 2022. https://www.ecdc.europa.eu/en/publications-data/surveillance-prevention-control-leishmaniases-European-Union-and-neighbouring-countries
102. WHO. The Global Health Observatory: Leishmaniasis. Accessed November 11, 2023. https://www.who.int/data/gho/data/themes/topics/indicator-groups/indicator-group-details/GHO/leishmaniasis
103. Fick SE, Hijmans RJ. WorldClim 2: new 1-km spatial resolution climate surfaces for global land areas. *Int J Climatol*. 2017;37(12):4302-4315. doi:10.1002/JOC.5086
104. Chen T, Guestrin C. XGBoost: A scalable tree boosting system. *Proc ACM SIGKDD Int Conf Knowl Discov Data Min*. 2016;13-17-August-2016:785-794. doi:10.1145/2939672.2939785
105. Chen T, He T. xgboost: eXtreme Gradient Boosting. Published 2023. Accessed November 11, 2023. https://cran.r-project.org/package=xgboost
106. Robin X, Turck N, Hainard A, et al. pROC: An open-source package for R and S+ to analyze and compare ROC curves. *BMC Bioinformatics*. 2011;12(1):1-8. doi:10.1186/1471-2105-12-77/TABLES/3
107. Ready PD. Leishmaniasis emergence in Europe. *Eurosurveillance*. 2010;15(10):29-39. doi:10.2807/ESE.15.10.19505-EN/CITE/PLAINTEXT
108. Rochlin I, Toledo A. Emerging tick-borne pathogens of public health importance: A mini-review. *J Med Microbiol*. 2020;69(6):781-791. doi:10.1099/JMM.0.001206/CITE/REFWORKS
109. Marques AR, Strle F, Wormser GP. Comparison of Lyme Disease in the United States and Europe. *Emerg Infect Dis*. 2021;27(8):2017-2024. doi:10.3201/EID2708.204763
110. Wondim MA, Czupryna P, Pancewicz S, Kruszewska E, Groth M, Moniuszko-Malinowska A. Epidemiological Trends of Trans-Boundary Tick-Borne Encephalitis in Europe, 2000–2019. *Pathog 2022, Vol 11, Page 704*. 2022;11(6):704. doi:10.3390/PATHOGENS11060704
111. Jenkins VA, Silbernagl G, Baer LR, Hoet B. The epidemiology of infectious diseases in Europe in 2020 versus 2017–2019 and the rise of tick-borne encephalitis (1995–2020). *Ticks Tick Borne Dis*. 2022;13(5):101972. doi:10.1016/J.TTBDIS.2022.101972
112. Vandekerckhove O, De Buck E, Van Wijngaerden E. Lyme disease in Western Europe: an emerging problem? A systematic review. *Acta Clin Belg*. 2021;76(3):244-252. doi:10.1080/17843286.2019.1694293
113. Mysterud A, Jore S, Østerås O, Viljugrein H. Emergence of tick-borne diseases at northern latitudes in Europe: a comparative approach. *Sci Reports 2017 71*. 2017;7(1):1-12. doi:10.1038/s41598-017-15742-6
114. Randolph SE. Tick ecology: processes and patterns behind the epidemiological risk posed by ixodid ticks as vectors. *Parasitology*. 2004;129 Suppl(SUPPL.). doi:10.1017/S0031182004004925
115. Nolzen H, Brugger K, Reichold A, Brock J, Lange M, Thulke HH. Model-based extrapolation of ecological systems under future climate scenarios: The example of Ixodes ricinus ticks. *PLoS One*.

2022;17(4):e0267196. doi:10.1371/JOURNAL.PONE.0267196

116. The Global Biodiversity Information Facility. Published 2023. Accessed November 11, 2023. https://www.gbif.org/occurrence/download/0035929-230530130749713

117. Faroux S, Kaptué Tchuenté AT, Roujean J-L, Masson V, Martin E, Le Moigne P. ECOCLIMAP-II/Europe: a twofold database of ecosystems and surface parameters at 1 km resolution based on satellite information for use in land surface, meteorological and climate models. *Geosci Model Dev*. 2013;6:563-582. doi:10.5194/gmd-6-563-2013

118. Köble R, Seufert G. Novel maps for forest tree species in Europe, in: European Symp. on the Physico-Chemical Behaviour of Air Pollutants: " A Changing Atmosphere ". Presented at the European Symp. on the Physico-Chemical Behaviour of Air Pollutants: " A Changing Atmosphere ", Torino.

119. Earth Resources Observation And Science (EROS) Center. Global Land Cover Characterization (GLCC). Published 2017. Accessed March 18, 2022. https://doi.org/10.5066/F7GB230D

120. Prank M, Chapman DS, Bullock JM, et al. An operational model for forecasting ragweed pollen release and dispersion in Europe. *Agric For Meteorol*. 2013;182-183:43-53. doi:10.1016/J.AGRFORMET.2013.08.003

121. Sofiev M, Palamarchuk J, Kouznetsov R, et al. European pollen reanalysis, 1980-2022, for alder, birch, and olive, v.1.0 (Version 1.0) [Data set]. *Finnish Meteorol Inst*. Published online 2023. doi:https://doi.org/10.57707/FMI-B2SHARE.980BC5264C6848859A3AB542A88979F9

122. Sofiev M, Palamarchuk J, Kouznetsov R. European pollen reanalysis, 1980-2022, for alder, birch, and olive, v.1.0 (Version 1.0). *Sci Data*.

123. Sofiev M, Vira J, Kouznetsov R, Prank M, Soares J, Genikhovich E. Construction of the SILAM Eulerian atmospheric dispersion model based on the advection algorithm of Michael Galperin. *Geosci Model Dev*. 2015;8(11):3497-3522. doi:10.5194/GMD-8-3497-2015

124. Sofiev M, Siljamo P, Ranta H, et al. A numerical model of birch pollen emission and dispersion in the atmosphere. Description of the emission module. *Int J Biometeorol*. 2013;57(1):45-58. doi:10.1007/S00484-012-0532-Z/FIGURES/4

125. Linkosalo T, Ranta H, Oksanen A, et al. A double-threshold temperature sum model for predicting the flowering duration and relative intensity of Betula pendula and B. pubescens. *Agric For Meteorol*. 2010;150(12):1579-1584. doi:10.1016/J.AGRFORMET.2010.08.007

126. Sofiev M, Siljamo P, Ranta H, Rantio-Lehtimäki A. Towards numerical forecasting of long-range air transport of birch pollen: Theoretical considerations and a feasibility study. *Int J Biometeorol*. 2006;50(6):392-402. doi:10.1007/S00484-006-0027-X/FIGURES/5

127. Sofiev M, Berger U, Prank M, et al. MACC regional multi-model ensemble simulations of birch pollen dispersion in Europe. *Atmos Chem Phys*. 2015;15(14):8115-8130. doi:10.5194/ACP-15-8115-2015

128. Sofiev M, Ritenberga O, Albertini R, et al. Multi-model ensemble simulations of olive pollen distribution in Europe in 2014: Current status and outlook. *Atmos Chem Phys*. 2017;17(20):12341-12360. doi:10.5194/ACP-17-12341-2017

129. Pfaar O, Bastl K, Berger U, et al. Defining pollen exposure times for clinical trials of allergen immunotherapy for pollen-induced rhinoconjunctivitis - an EAACI position paper. *Allergy*. 2017;72(5):713-722. doi:10.1111/ALL.13092

130. Ritenberga O, Sofiev M, Siljamo P, et al. A statistical model for predicting the inter-annual variability of birch pollen abundance in Northern and North-Eastern Europe. *Sci Total Environ*. 2018;615:228-239. doi:10.1016/J.SCITOTENV.2017.09.061

131. Cafiero C, Viviani S, Nord M. Food security measurement in a global context: The food insecurity experience scale. *Measurement*. 2018;116:146-152. doi:10.1016/J.MEASUREMENT.2017.10.065

132. Ballard TJ, Kepple AW, Cafiero C, Schmidhuber J, Italy R/. Better measurement of food insecurity in the context of enhancing nutrition 1 The "Voices of the Hungry" project. doi:10.4455/eu.2014.007

133. Dasgupta S, Robinson EJZ. Attributing changes in food insecurity to a changing climate. *Sci Reports 2022 121*. 2022;12(1):1-11. doi:10.1038/s41598-022-08696-x

134. Dasgupta S, Robinson EJZ. Improving Food Policies for a Climate Insecure World: Evidence from Ethiopia. *Natl Inst Econ Rev* . 2021;258:66-82. doi:10.1017/NIE.2021.35

135. Dasgupta S, van Maanen N, Gosling SN, Piontek F, Otto C, Schleussner CF. Effects of climate change on combined labour productivity and supply: an empirical, multi-model study. *Lancet Planet Heal*. 2021;5(7):e455-e465. doi:10.1016/S2542-5196(21)00170-4/ATTACHMENT/F9ABD22F-AA15-40B1-A694-ACA36E0FA68F/MMC1.PDF

136. Cohen-Shacham E, Walters. G, Janzen C, Maginnis S. *Nature-Based Solutions to Address Global Societal Challenges.*

137. Goodwin S, Olazabal M, Castro AJ, Pascual U. Global mapping of urban nature-based solutions for climate change adaptation. *Nat Sustain 2023 64*. 2023;6(4):458-469. doi:10.1038/s41893-022-01036-x

138. Semenza JC. Lateral public health: Advancing systemic resilience to climate change. *Lancet Reg Heal – Eur*. 2021;9. doi:10.1016/J.LANEPE.2021.100231

139. Kabisch N, van den Bosch M, Lafortezza R. The health benefits of nature-based solutions to urbanization challenges for children and the elderly - A systematic review. *Environ Res*. 2017;159:362-373. doi:10.1016/J.ENVRES.2017.08.004

140. Kolokotsa D, Lilli A, Lilli MA, Nikolaidis NP. On the impact of nature-based solutions on citizens' health & well being. *Energy Build*. 2020;229:110527. doi:10.1016/J.ENBUILD.2020.110527

141. Rhodes CG, Scavo NA, Finney M, et al. Meta-Analysis of the Relative Abundance of Nuisance and Vector Mosquitoes in Urban and Blue-Green Spaces. *Insects*. 2022;13(3). doi:10.3390/INSECTS13030271

142. Schüle SA, Gabriel KMA, Bolte G. Relationship between neighbourhood socioeconomic position and neighbourhood public green space availability: An environmental inequality analysis in a large German city applying generalized linear models. *Int J Hyg Environ Health*. 2017;220(4):711-718. doi:10.1016/J.IJHEH.2017.02.006

143. Chen Y, Yue W, La Rosa D. Which communities have better accessibility to green space? An investigation into environmental inequality using big data. *Landsc Urban Plan*. 2020;204:103919. doi:10.1016/J.LANDURBPLAN.2020.103919

144. Anguelovski I, Connolly JJT, Cole H, et al. Green gentrification in European and North American cities. *Nat Commun 2022 131*. 2022;13(1):1-13. doi:10.1038/s41467-022-31572-1

145. Oscilowicz E, Anguelovski I, Triguero-Mas M, García-Lamarca M, Baró F, Cole HVS. Green justice through policy and practice: a call for further research into tools that foster healthy green cities for all.

Cities Heal. 2022;6(5):878-893. doi:10.1080/23748834.2022.2072057

146. Triguero-Mas M, Anguelovski I, García-Lamarca M, et al. Natural outdoor environments' health effects in gentrifying neighborhoods: Disruptive green landscapes for underprivileged neighborhood residents. *Soc Sci Med*. 2021;279:113964. doi:10.1016/J.SOCSCIMED.2021.113964
147. Paciência I, Moreira A, Moreira C, et al. Neighbourhood green and blue spaces and allergic sensitization in children: A longitudinal study based on repeated measures from the Generation XXI cohort. *Sci Total Environ*. 2021;772:145394. doi:10.1016/J.SCITOTENV.2021.145394
148. World Health Organization. 2021 WHO health and climate change global survey report. Published online 2021:1-79. Accessed March 18, 2022. https://www.who.int/health-topics/climate-change#tab=tab_1
149. Didan K, Barreto Munoz A, Solano R, Huete A. *MODIS Vegetation Index User's Guide (MOD13 Series).*; 2015.
150. Didan K. MOD13Q1 MODIS/Terra Vegetation Indices 16-Day L3 Global 250m SIN Grid V006. NASA EOSDIS Land Processes DAAC. *USGS*. 2015;5:2002-2015. doi:10.5067/MODIS
151. Pekel JF, Cottam A, Gorelick N, Belward AS. High-resolution mapping of global surface water and its long-term changes. *Nat 2016 5407633*. 2016;540(7633):418-422. doi:10.1038/nature20584
152. IEA. *CO2 Emissions from Fuel Combustion Statistics: Greenhouse Gas Emissions from Energy 2023*. doi:https://doi.org/10.1787/co2-data-en
153. IEA. *World Energy Statistics and Balances 2023.* doi:https://doi.org/10.1787/enestats-data-en
154. EUROSTAT. Energy statistics - an overview. Published 2023. Accessed July 25, 2023. https://ec.europa.eu/eurostat/statistics-explained/index.php?title=Energy_statistics_-_an_overview#Final_energy_consumption
155. International Environment Agency. World Extended Energy Balances (2021 edition). Accessed March 18, 2022. https://www.oecd-ilibrary.org/energy/data/iea-world-energy-statistics-and-balances/extended-world-energy-balances-edition-2021_cac5fa90-en
156. Food and Agriculture Organization of the United Nations. FAOSTAT Statistical Database. Published 2022. Accessed March 18, 2022. https://www.fao.org/faostat/en/#home
157. IFASTAT. Accessed March 18, 2022. https://www.fertilizer.org/Public/Market_Intelligence/IFASTAT/Public/IFA_Stat/IFASTAT.aspx?hkey=16e71efa-7bd3-421e-8344-3a191a46078f
158. Amann M, Bertok I, Borken-Kleefeld J, et al. Cost-effective control of air quality and greenhouse gases in Europe: Modeling and policy applications. *Environ Model Softw*. 2011;26(12):1489-1501. doi:10.1016/J.ENVSOFT.2011.07.012
159. Simpson D, Benedictow A, Berge H, et al. The EMEP MSC-W chemical transport model: technical description. *Atmos Chem Phys*. 2012;12(16):7825-7865. doi:10.5194/ACP-12-7825-2012
160. Menut L, Bessagnet B, Khvorostyanov D, et al. CHIMERE 2013: a model for regional atmospheric composition modelling. *Geosci Model Dev*. 2013;6(4):981-1028. doi:10.5194/GMD-6-981-2013
161. Chen J, Hoek G. Long-term exposure to PM and all-cause and cause-specific mortality: A systematic review and meta-analysis. *Environ Int*. 2020;143:105974. doi:10.1016/J.ENVINT.2020.105974
162. Rafaj P, Amann M, Siri JG. Factorization of air pollutant emissions: Projections versus observed trends

in Europe. *Sci Total Environ*. 2014;494-495:272-282. doi:10.1016/J.SCITOTENV.2014.07.013
163. Commission E. *The Third Clean Air Outlook. COM*.; 2022. https://eur-lex.europa.eu/legal-content/EN/TXT/HTML/?uri=CELEX:52022DC0673
164. Commission E. *Impact Assessment Report Accompanying the Document Proposal for a Directive of the European Parliament and of the Council on Ambient Air Quality and Cleaner Air for Europe*.; 2022. https://eur-lex.europa.eu/resource.html?uri=cellar:a5235624-55f8-11ed-92ed-01aa75ed71a1.0001.02/DOC_1&format=PDF
165. European Environment Agency. *Air Quality in Europe 2022*.; 2022. https://www.eea.europa.eu/publications/air-quality-in-europe-2022
166. Hoffmann B, Brunekreef B, Andersen ZJ, Forastiere F, Boogaard H. Benefits of future clean air policies in Europe: Proposed analyses of the mortality impacts of PM2.5and NO2. *Environ Epidemiol*. 2022;6(5):E221. doi:10.1097/EE9.0000000000000221
167. Brunekreef B, Strak M, Chen J, et al. *Mortality and Morbidity Effects of Long-Term Exposure to Low-Level PM2.5, BC, NO2, and O3: An Analysis of European Cohorts in the ELAPSE Project*. Vol 2021. Health Effects Institute; 2021. Accessed November 10, 2023. /pmc/articles/PMC9476567/
168. World Population Prospects - Population Division - United Nations. Accessed March 18, 2022. https://population.un.org/wpp/
169. Modal split of passenger transport. Accessed March 18, 2022. http://appsso.eurostat.ec.europa.eu/nui/show.do?dataset=tran_hv_psmod
170. Miller V, Singh GM, Onopa J, et al. Global Dietary Database 2017: data availability and gaps on 54 major foods, beverages and nutrients among 5.6 million children and adults from 1220 surveys worldwide. *BMJ Glob Heal*. 2021;6(2):e003585. doi:10.1136/BMJGH-2020-003585
171. Food and Agriculture Organization of the United Nations. Food balance sheets: a handbook. Accessed March 18, 2022. https://www.fao.org/3/x9892e/X9892e.htm
172. Poore J, Nemecek T. Reducing food's environmental impacts through producers and consumers. *Science (80-)*. 2018;360(6392):987-992. doi:10.1126/SCIENCE.AAQ0216/SUPPL_FILE/AAQ0216_DATAS2.XLS
173. Achieve more sustainable agriculture | Hestia. Accessed November 10, 2023. https://www.hestia.earth/
174. Gustavsson J, Cederberg C, Sonesson U, Van Otterdijk R, Meybeck A, Rome F. Global food losses and food waste: extent, causes and prevention. *Food Agric Organ*. Published online 2011:1-37. Accessed March 18, 2022. http://www.fao.org/news/story/en/item/74192/icode/
175. Di Cesare M, Bentham J, Stevens GA, et al. Trends in adult body-mass index in 200 countries from 1975 to 2014: A pooled analysis of 1698 population-based measurement studies with 19.2 million participants. *Lancet*. 2016;387(10026):1377-1396. doi:10.1016/S0140-6736(16)30054-X/ATTACHMENT/50E15529-B93E-4149-B990-695FF383D537/MMC1.PDF
176. Afshin A, Micha R, Khatibzadeh S, Mozaffarian D. Consumption of nuts and legumes and risk of incident ischemic heart disease, stroke, and diabetes: a systematic review and meta-analysis. *Am J Clin Nutr*. 2014;100(1):278-288. doi:10.3945/AJCN.113.076901
177. Aune D, Keum NN, Giovannucci E, et al. Nut consumption and risk of cardiovascular disease, total cancer, all-cause and cause-specific mortality: A systematic review and dose-response meta-analysis of

prospective studies. *BMC Med*. 2016;14(1):1-14. doi:10.1186/S12916-016-0730-3/TABLES/1

178. Aune D, Giovannucci E, Boffetta P, et al. Fruit and vegetable intake and the risk of cardiovascular disease, total cancer and all-cause mortality—a systematic review and dose-response meta-analysis of prospective studies. *Int J Epidemiol*. 2017;46(3):1029-1056. doi:10.1093/IJE/DYW319

179. Bechthold A, Boeing H, Schwedhelm C, et al. Food groups and risk of coronary heart disease, stroke and heart failure: A systematic review and dose-response meta-analysis of prospective studies. *https://doi.org/101080/1040839820171392288*. 2017;59(7):1071-1090. doi:10.1080/10408398.2017.1392288

180. Schwingshackl L, Hoffmann G, Lampousi AM, et al. Food groups and risk of type 2 diabetes mellitus: a systematic review and meta-analysis of prospective studies. *Eur J Epidemiol*. 2017;32(5):363-375. doi:10.1007/S10654-017-0246-Y/TABLES/1

181. Schwingshackl L, Schwedhelm C, Hoffmann G, et al. Food groups and risk of colorectal cancer. *Int J Cancer*. 2018;142(9):1748-1758. doi:10.1002/IJC.31198

182. Di Angelantonio E, Bhupathiraju SN, Wormser D, et al. Body-mass index and all-cause mortality: individual-participant-data meta-analysis of 239 prospective studies in four continents. *Lancet*. 2016;388(10046):776-786. doi:10.1016/S0140-6736(16)30175-1/ATTACHMENT/E2A32D35-AEAE-445A-8FEB-FE6D9C03DA38/MMC1.PDF

183. Abbafati C, Machado DB, Cislaghi B, et al. Global age-sex-specific fertility, mortality, healthy life expectancy (HALE), and population estimates in 204 countries and territories, 1950–2019: a comprehensive demographic analysis for the Global Burden of Disease Study 2019. *Lancet*. 2020;396(10258):1160-1203. doi:10.1016/S0140-6736(20)30977-6/ATTACHMENT/4E76F1CB-0626-4875-9CF1-96DD065CD148/MMC2.PDF

184. Del Gobbo LC, Khatibzadeh S, Imamura F, et al. Assessing global dietary habits: a comparison of national estimates from the FAO and the Global Dietary Database. *Am J Clin Nutr*. 2015;101(5):1038-1046. doi:10.3945/AJCN.114.087403

185. Micha R, Khatibzadeh S, Shi P, Andrews KG, Engell RE, Mozaffarian D. Global, regional and national consumption of major food groups in 1990 and 2010: a systematic analysis including 266 country-specific nutrition surveys worldwide. *BMJ Open*. 2015;5(9):e008705. doi:10.1136/BMJOPEN-2015-008705

186. Freedman LS, Commins JM, Moler JE, et al. Pooled Results From 5 Validation Studies of Dietary Self-Report Instruments Using Recovery Biomarkers for Energy and Protein Intake. *Am J Epidemiol*. 2014;180(2):172-188. doi:10.1093/AJE/KWU116

187. Rennie KL, Coward A, Jebb SA. Estimating under-reporting of energy intake in dietary surveys using an individualised method. *Br J Nutr*. 2007;97(6):1169-1176. doi:10.1017/S0007114507433086

188. Murray CJL, Ezzati M, Lopez AD, Rodgers A, Vander Hoorn S. Comparative quantification of health risks: Conceptual framework and methodological issues. *Popul Health Metr*. 2003;1(1):1-20. doi:10.1186/1478-7954-1-1/FIGURES/4

189. S.S. L, T. V, A.D. F, et al. A comparative risk assessment of burden of disease and injury attributable to 67 risk factors and risk factor clusters in 21 regions, 1990-2010: A systematic analysis for the Global Burden of Disease Study 2010. *Lancet*. 2012;380(9859):2224-2260.

doi:http://dx.doi.org/10.1016/S0140-6736%2812%2961766-8
190. Forouzanfar MH, Alexander L, Bachman VF, et al. Global, regional, and national comparative risk assessment of 79 behavioural, environmental and occupational, and metabolic risks or clusters of risks in 188 countries, 1990-2013: A systematic analysis for the Global Burden of Disease Study 2013. *Lancet*. 2015;386(10010):2287-2323. doi:10.1016/S0140-6736(15)00128-2/ATTACHMENT/8ECECF19-44E8-4A5A-843A-B063BA3F32E2/MMC1.PDF
191. Murray CJL, Ezzati M, Flaxman AD, et al. GBD 2010: Design, definitions, and metrics. *Lancet*. 2012;380(9859):2063-2066. doi:10.1016/S0140-6736(12)61899-6/ATTACHMENT/40CF69A5-C53F-4177-85F3-F2177FA6EC29/MMC1.PDF
192. Singh GM, Danaei G, Farzadfar F, et al. The Age-Specific Quantitative Effects of Metabolic Risk Factors on Cardiovascular Diseases and Diabetes: A Pooled Analysis. *PLoS One*. 2013;8(7):e65174. doi:10.1371/JOURNAL.PONE.0065174
193. Micha R, Shulkin ML, Peñalvo JL, et al. Etiologic effects and optimal intakes of foods and nutrients for risk of cardiovascular diseases and diabetes: Systematic reviews and meta-analyses from the Nutrition and Chronic Diseases Expert Group (NutriCoDE). *PLoS One*. 2017;12(4):e0175149. doi:10.1371/JOURNAL.PONE.0175149
194. Afshin A, Sur PJ, Fay KA, et al. Health effects of dietary risks in 195 countries, 1990–2017: a systematic analysis for the Global Burden of Disease Study 2017. *Lancet*. 2019;393(10184):1958-1972. doi:10.1016/S0140-6736(19)30041-8/ATTACHMENT/89650CE3-EC1A-450B-AD88-3B2DB16AD72D/MMC1.PDF
195. Schwingshackl L, Knüppel S, Schwedhelm C, et al. Perspective: NutriGrade: A Scoring System to Assess and Judge the Meta-Evidence of Randomized Controlled Trials and Cohort Studies in Nutrition Research. *Adv Nutr*. 2016;7(6):994-1004. doi:10.3945/AN.116.013052
196. World Cancer Research Fund/American Institute for Cancer Research. Diet, Nutrition, Physical Activity and Cancer: A Global Perspective. Accessed March 19, 2022. https://www.wcrf.org/diet-and-cancer/
197. Aune D, Norat T, Romundstad P, Vatten LJ. Dairy products and the risk of type 2 diabetes: a systematic review and dose-response meta-analysis of cohort studies. *Am J Clin Nutr*. 2013;98(4):1066-1083. doi:10.3945/AJCN.113.059030
198. Aune D, Lau R, Chan DSM, et al. Dairy products and colorectal cancer risk: a systematic review and meta-analysis of cohort studies. *Ann Oncol*. 2012;23(1):37-45. doi:10.1093/ANNONC/MDR269
199. Mohan D, Mente A, Dehghan M, et al. Associations of Fish Consumption With Risk of Cardiovascular Disease and Mortality Among Individuals With or Without Vascular Disease From 58 Countries. *JAMA Intern Med*. 2021;181(5):631-649. doi:10.1001/JAMAINTERNMED.2021.0036
200. Satija A, Yu E, Willett WC, Hu FB. Understanding Nutritional Epidemiology and Its Role in Policy. *Adv Nutr*. 2015;6(1):5-18. doi:10.3945/AN.114.007492
201. Zheng J, Huang T, Yu Y, Hu X, Yang B, Li D. Fish consumption and CHD mortality: an updated meta-analysis of seventeen cohort studies. *Public Health Nutr*. 2012;15(4):725-737. doi:10.1017/S1368980011002254
202. Aune D, Keum N, Giovannucci E, et al. Whole grain consumption and risk of cardiovascular disease, cancer, and all cause and cause specific mortality: systematic review and dose-response meta-analysis of

prospective studies. *BMJ*. 2016;353. doi:10.1136/BMJ.I2716

203. MacMahon S, Baigent C, Duffy S, et al. Body-mass index and cause-specific mortality in 900 000 adults: Collaborative analyses of 57 prospective studies. *Lancet*. 2009;373(9669):1083-1096. doi:10.1016/S0140-6736(09)60318-4/ATTACHMENT/43FF481A-61DA-4EDE-9FC5-F7D72B899318/MMC1.PDF

204. Schwingshackl L, Hoffmann G, Iqbal K, Schwedhelm C, Boeing H. Food groups and intermediate disease markers: a systematic review and network meta-analysis of randomized trials. *Am J Clin Nutr*. 2018;108(3):576-586. doi:10.1093/AJCN/NQY151

205. Xun P, Qin B, Song Y, et al. Fish consumption and risk of stroke and its subtypes: accumulative evidence from a meta-analysis of prospective cohort studies. *Eur J Clin Nutr 2012 6611*. 2012;66(11):1199-1207. doi:10.1038/ejcn.2012.133

206. Jayedi A, Shab-Bidar S, Eimeri S, Djafarian K. Fish consumption and risk of all-cause and cardiovascular mortality: a dose–response meta-analysis of prospective observational studies. *Public Health Nutr*. 2018;21(7):1297-1306. doi:10.1017/S1368980017003834

207. Zhao LG, Sun JW, Yang Y, Ma X, Wang YY, Xiang YB. Fish consumption and all-cause mortality: a meta-analysis of cohort studies. *Eur J Clin Nutr 2016 702*. 2015;70(2):155-161. doi:10.1038/ejcn.2015.72

208. Guasch-Ferré M, Satija A, Blondin SA, et al. Meta-Analysis of Randomized Controlled Trials of Red Meat Consumption in Comparison with Various Comparison Diets on Cardiovascular Risk Factors. *Circulation*. 2019;139(15):1828-1845. doi:10.1161/CIRCULATIONAHA.118.035225

209. Schwingshackl L, Knüppel S, Michels N, et al. Intake of 12 food groups and disability-adjusted life years from coronary heart disease, stroke, type 2 diabetes, and colorectal cancer in 16 European countries. *Eur J Epidemiol*. 2019;34(8):765-775. doi:10.1007/S10654-019-00523-4/TABLES/3

210. Mortality Risk Valuation in Environment, Health and Transport Policies - OECD. Accessed March 19, 2022. https://www.oecd.org/environment/mortalityriskvaluationinenvironmenthealthandtransportpolicies.htm

211. Springmann M, Wiebe K, Mason-D’Croz D, Sulser TB, Rayner M, Scarborough P. Health and nutritional aspects of sustainable diet strategies and their association with environmental impacts: a global modelling analysis with country-level detail. *Lancet Planet Heal*. 2018;2(10):e451-e461. doi:10.1016/S2542-5196(18)30206-7

212. Poore J, Nemecek T. Reducing food’s environmental impacts through producers and consumers. *Science (80-)*. 2018;360(6392):987-992. doi:10.1126/science.aaq0216

213. Springmann M, Clark M, Mason-D’Croz D, et al. Options for keeping the food system within environmental limits. *Nat 2018 5627728*. 2018;562(7728):519-525. doi:10.1038/s41586-018-0594-0

214. Springmann M, Godfray HCJ, Rayner M, Scarborough P. Analysis and valuation of the health and climate change cobenefits of dietary change. *Proc Natl Acad Sci U S A*. 2016;113(15):4146-4151. doi:10.1073/PNAS.1523119113/SUPPL_FILE/PNAS.1523119113.SAPP.PDF

215. Lindhjem H, Navrud S, Braathen NA, Biausque V. Valuing Mortality Risk Reductions from Environmental, Transport, and Health Policies: A Global Meta-Analysis of Stated Preference Studies. *Risk Anal*. 2011;31(9):1381-1407. doi:10.1111/J.1539-6924.2011.01694.X

216. International Energy Agency. *World Energy Investment 2023.*; 2023. https://www.iea.org/reports/world-energy-investment-2023

217. Berrang-Ford L, Sietsma AJ, Callaghan M, et al. Systematic mapping of global research on climate and health: a machine learning review. *Lancet Planet Heal*. 2021;5(8):e514-e525. doi:10.1016/S2542-5196(21)00179-0/ATTACHMENT/6A529F7F-DF6B-4C1B-B5AB-258A524E6205/MMC2.XLSX

218. Halterman A. Mordecai: Full Text Geoparsing and Event Geocoding. *J Open Source Softw*. 2017;2(9):91. doi:10.21105/JOSS.00091

219. Callaghan M, Schleussner CF, Nath S, et al. Machine-learning-based evidence and attribution mapping of 100,000 climate impact studies. *Nat Clim Chang 2021 1111*. 2021;11(11):966-972. doi:10.1038/s41558-021-01168-6

220. Knutson TR, Zeng F, Wittenberg AT. Multimodel Assessment of Regional Surface Temperature Trends: CMIP3 and CMIP5 Twentieth-Century Simulations. *J Clim*. 2013;26(22):8709-8743. doi:10.1175/JCLI-D-12-00567.1

221. Knutson TR, Zeng F. Model Assessment of Observed Precipitation Trends over Land Regions: Detectable Human Influences and Possible Low Bias in Model Trends. *J Clim*. 2018;31(12):4617-4637. doi:10.1175/JCLI-D-17-0672.1

222. Cody EM, Reagan AJ, Mitchell L, Dodds PS, Danforth CM. Climate Change Sentiment on Twitter: An Unsolicited Public Opinion Poll. *PLoS One*. 2015;10(8):e0136092. doi:10.1371/JOURNAL.PONE.0136092

223. Sanford M, Painter J, Yasseri T, Lorimer J. Controversy around climate change reports: a case study of Twitter responses to the 2019 IPCC report on land. *Clim Change*. 2021;167(3-4):1-25. doi:10.1007/S10584-021-03182-1/TABLES/5

224. Boykoff MT, Roberts JT, Timmons J, James R, Fellow MR, Martin J. Media coverage of climate change: current trends, strengths, weaknesses. *Hum Dev Rep*. Published online 2007.

225. Stryker JE, Moriarty CM, Jensen JD. Effects of Newspaper Coverage on Public Knowledge About Modifiable Cancer Risks. *Health Commun*. 2008;23(4):380-390. doi:10.1080/10410230802229894

226. Sampei Y, Aoyagi-Usui M. Mass-media coverage, its influence on public awareness of climate-change issues, and implications for Japan’s national campaign to reduce greenhouse gas emissions. *Glob Environ Chang*. 2009;19(2):203-212. doi:10.1016/J.GLOENVCHA.2008.10.005

227. Culotta A. Towards detecting influenza epidemics by analyzing Twitter messages. *SOMA 2010 - Proc 1st Work Soc Media Anal*. Published online 2010:115-122. doi:10.1145/1964858.1964874

228. Fischhoff B, Brewer NT, Downs JS. *Communicating Risks and Benefits : An Evidence-Based User’s Guide*. Food and Drug Administration - Department of Health and Human Services; 2012.

229. Schmidt A, Ivanova A, Schäfer MS. Media attention for climate change around the world: A comparative analysis of newspaper coverage in 27 countries. *Glob Environ Chang*. 2013;23(5):1233-1248. doi:10.1016/J.GLOENVCHA.2013.07.020

230. Park CS, Kaye BK. The tweet goes on: Interconnection of Twitter opinion leadership, network size, and civic engagement. *Comput Human Behav*. 2017;69:174-180. doi:10.1016/J.CHB.2016.12.021

231. Park CS. Does Twitter motivate involvement in politics? Tweeting, opinion leadership, and political engagement. *Comput Human Behav*. 2013;29(4):1641-1648. doi:10.1016/J.CHB.2013.01.044

232. Carley KM, Malik M, Landwehr PM, Pfeffer J, Kowalchuck M. Crowd sourcing disaster management: The complex nature of Twitter usage in Padang Indonesia. *Saf Sci*. 2016;90:48-61. doi:10.1016/J.SSCI.2016.04.002

233. Chew C, Eysenbach G. Pandemics in the Age of Twitter: Content Analysis of Tweets during the 2009 H1N1 Outbreak. *PLoS One*. 2010;5(11):e14118. doi:10.1371/JOURNAL.PONE.0014118
